# Optimizing integration of community-based management of possible serious bacterial infection (PSBI) in young infants into primary healthcare systems in Ethiopia and Kenya: successes and challenges

**DOI:** 10.1186/s12913-024-10679-9

**Published:** 2024-03-05

**Authors:** Gizachew Tadele Tiruneh, George Odwe, Alexandra Haake Kamberos, Kezia K’Oduol, Nebreed Fesseha, Zipporah Moraa, Hellen Gwaro, Dessalew Emaway, Hema Magge, Yasir Bin Nisar, Lisa R. Hirschhorn

**Affiliations:** 1 The Last Ten Kilometers (L10K) Project, JSI Research & Training Institute, Inc, Addis Ababa, Ethiopia; 2 Population Council, Nairobi, Kenya; 3 Feinberg School of Medicine and Havey Institute of Global Health, Northwestern University, Chicago, IL USA; 4 Living Goods, Nairobi, Kenya; 5 Lwala Community Alliance, Nairobi, Kenya; 6 Bill & Melinda Gates Foundation, Seattle, USA; 7 Department of Maternal, Newborn, Child and Adolescent Health and Ageing, World Health Organization, Geneva, Switzerland

**Keywords:** Community-based integrated community case management of common childhood illnesses, COVID-19, Ethiopia, Implementation research, Integration, Kenya, Neonatal mortality, Possible serious bacterial infection, Sustainability

## Abstract

**Background:**

Ethiopia and Kenya have adopted the community-based integrated community case management (iCCM) of common childhood illnesses and newborn care strategy to improve access to treatment of infections in newborns and young infants since 2012 and 2018, respectively. However, the iCCM strategy implementation has not been fully integrated into the health system in both countries. This paper describes the extent of integration of iCCM program at the district/county health system level, related barriers to optimal integration and implementation of strategies.

**Methods:**

From November 2020 to August 2021, Ethiopia and Kenya implemented the community-based treatment of possible serious bacterial infection (PSBI) when referral to a higher facility is not possible using embedded implementation research (eIR) to mitigate the impact of COVID-19 on the delivery of this life-saving intervention. Both projects conducted mixed methods research from April-May 2021 to identify barriers and facilitators and inform strategies and summative evaluations from June-July 2022 to monitor the effectiveness of implementation outcomes including integration of strategies.

**Results:**

Strategies identified as needed for successful implementation and sustainability of the management of PSBI integrated at the primary care level included continued coaching and support systems for frontline health workers, technical oversight from the district/county health system, and ensuring adequate supply of commodities. As a result, support and technical oversight capacity and collaborative learning were strengthened between primary care facilities and community health workers, resulting in improved bidirectional linkages. Improvement of PSBI treatment was seen with over 85% and 81% of estimated sick young infants identified and treated in Ethiopia and Kenya, respectively. However, perceived low quality of service, lack of community trust, and shortage of supplies remained barriers impeding optimal PSBI services access and delivery.

**Conclusion:**

Pragmatic eIR identified shared and unique contextual challenges between and across the two countries which informed the design and implementation of strategies to optimize the integration of PSBI management into the health system during the COVID-19 pandemic. The eIR participatory design also strengthened ownership to operationalize the implementation of identified strategies needed to improve the health system’s capacity for PSBI treatment.

**Supplementary Information:**

The online version contains supplementary material available at 10.1186/s12913-024-10679-9.

## Background

Sub-Saharan Africa (SSA) has the highest neonatal mortality rate (NMR) in the world, with 27 deaths per 1,000 live births in 2021. While childhood mortality has decreased significantly there over the last two decades, the reduction in NMR has not been as substantial in Ethiopia and Kenya. In 2021, the NMR in Ethiopia and Kenya was estimated at 26 and 18 deaths per 1,000 live births, respectively [1]. In SSA, possible serious bacterial infection (PSBI) is a leading cause of mortality in infants (aged 0–59 days), accounting for 37% of the neonatal deaths [2].

It is recommended that sick young infants (SYIs) with any clinical sign of PSBI receive treatment in a hospital setting [3, 4]. However, in resource-limited settings, many infants with signs of infection do not receive the recommended inpatient care due to limited affordable and accessible healthcare services [5, 6]. In 2015, the World Health Organization (WHO) released guidelines for the outpatient management of PSBI, which included simplified treatment regimens involving injectable and oral antibiotics when referral is not feasible [3]. Both Ethiopia and Kenya adopted the WHO’s clinical and programmatic guidance by incorporating PSBI management guidelines into their integrated- management of childhood illnesses (IMNCI) and community case management (iCCM) strategies in the year 2012 and 2018, respectively [7, 8].

In Ethiopia, the Ministry of Health (MOH) guides the community-based treatment of PSBI through its flagship Health Extension Program, which follows the continuum of the maternal, newborn, and child health care framework from pregnancy through childhood, encompassing households and health facilities [9]. Health Extension Workers (HEWs) are trained to identify newborn infections and provide treatment when referral is not feasible. In Kenya, under the iCCM strategy, the Community Health Workers, known as Community Health Volunteers (CHVs), are responsible for assessing danger signs in newborns during postnatal home visits, referring them to the nearest facility if any danger sign is present, and conducting follow-ups at home to ensure adherence to PSBI treatment for SYIs. The community healthcare workers in both countries are required to educate mothers about infection prevention measures, early recognition of illness in newborns, and prompt care-seeking [7, 8].

Despite these efforts, the uptake of sick newborn care remains low, and the full operational integration of iCCM/PSBI strategy into the health system has not been achieved in either country [10–12]. The emergence of COVID-19 in 2020 compromised the system’s capacity in both countries to deliver essential health care due to the diversion of resources, fear, stigma, and movement restrictions that resulted in decreased client demand for maternal and child health services [13, 14]. The formative assessment findings conducted in early 2021 showed that COVID-19 pandemic and related control measures affected the uptake and delivery of essential maternal and newborn health including PSBI treatment, in the first 2–3 months of the pandemic and continued to affect activities related to generating awareness of PSBI, such as interrupting social gatherings, community meetings, and community sensitization programs [13]. Factors such as inconsistent essential drug supplies, inadequate supportive and referral linkages, limited awareness of available PSBI services, and a lack of training and clinical mentoring of health workers contribute to weak implementation of PSBI management guidelines [10, 11, 15–17]. At the household or community level, many caregivers and decision-makers often fail to recognize signs and symptoms of illness in newborns, leading to delays in seeking care for SYIs [18, 19].

To respond to COVID-19, health system actors built the capacity of the health workforce to prevent transmission of COVID-19, enhanced risk communication and community engagement (RCCE) for community surveillance, and strengthened coordination and support for health facilities [20]. To further strengthen government’s effort and mitigate the impact of the COVID-19 pandemic on neonatal mortality, we implemented the “COVID-19: Mitigating Neonatal Mortality Project”—an embedded implementation research (eIR) project in Ethiopia, Kenya, and India. The goal of the three-country project was to strengthen community health systems and the linkages between community and health facilities to improve the management of SYIs with PSBI when referral is not feasible during the pandemic. In Ethiopia, the project was implemented by JSI Research & Training Institute Inc. (JSI) between November 2020 and June 2022. In Kenya, it was implemented by a consortium led by Living Goods, Population Council Kenya, and Lwala Community Alliance organizations in close collaboration with the Busia and Migori county and sub-county health management teams (CHMTs/SCHMTs) from December 2020 through August 2022.

Previous publications from this eIR in Ethiopia and Kenya described the factors affecting healthcare-seeking behaviors of mothers to their sick young infants and facilitators and barriers of community-facility linkages in implementing PSBI treatment [13, 21] and application of implementation research frameworks in designing, implementing, and evaluating strategies to improve community-based treatment of PSBI during the COVID-19 pandemic [10]. In this paper, we assessed the integration of adaptive strategies into the health system in two of the three countries where community health system integration was a core strategy employed to strengthen PSBI treatment, and the program was completed. We describe the extent to which PSBI management has been integrated into the iCCM programs at the district/county health system level at the start of the project and the application of eIR to identify barriers and strategies needed to strengthen the integration and implementation of PSBI management into community health systems in Ethiopia and Kenya. Specifically, we (1) examined the integration of PSBI management through a participatory design and implementation process; (2) evaluated the health system’s response in terms of the provision of necessary supplies and supportive supervision, as well as the establishment of linkages between communities and primary healthcare systems. Finally, (3) we assessed program performance.

## Methods

### Context

Ethiopia’s health system is structured into three levels of care: primary-level care, which encompasses a primary hospital, health centers, and health posts; secondary-level care; and tertiary-level care [22]. Primary health care focuses on providing preventive and promotive community and outreach services through the expansive Health Extension Program and the involvement of community volunteers [22]. Health posts are staffed with two female HEWs, who are government employees with a one-year certificate of education after secondary school. They serve as frontline health workers. In 2010, Ethiopia introduced iCCM by HEWs. The management of community-based newborn sepsis was subsequently incorporated within the iCCM strategy in 2012 to improve the utilization of iCCM services for newborns and young infants at the community level when referral is not feasible [23]. As part of the iCCM strategy, Ethiopia tested and adapted the simplified regimen (i.e., two days gentamicin injection and seven days oral amoxicillin) for sick newborns with suspected bacterial infection in 2018 [7]. The outpatient treatment of PSBI is provided at both health posts and health centers.

Kenya’s health system is comprised of five tiers: national referral and teaching hospitals (level 5), county and sub-county hospitals (level 4), health centers (level 3), dispensaries (level 2), and community health structures (level 1). Hospitals represent secondary and tertiary health facilities, while health centers and dispensaries serve as primary health care (PHC) facilities. The community health service, which constitutes the first level of the health system, includes Community Health Committees, Community Health Assistants or Community Health Officers (CHO), and Community Health Volunteers (CHVs). CHVs are deployed at level 1 to deliver preventive and promotive health services in the community. Each Community Health Assistant or Community Health Officer oversees 10 CHVs, who are responsible for overseeing up to 5,000 people (500-1,000 households), with the Community Health Committee serving as the governing body for the unit. In Kenya, WHO guidelines for the management of PSBI when referral is not feasible were integrated into IMNCI in 2018 [24]. At the community level, a simplified version of IMNCI is incorporated into the iCCM module, including strategies such as postnatal home visits by CHVs within 48 h of birth to assess for danger signs in the newborn and mother and referral to a nearby health facility, if necessary. CHVs visit homes within 48 h and on days three and seven following births, and for newborns referred to the health facility, CHVs conduct follow-up visits for PSBI cases [25]. PSBI outpatient treatment is provided at health centers and dispensaries.

This eIR in Ethiopia was conducted in two districts, which included 11 health centers and 66 health posts, serving an estimated 250,000 people. In Kenya, the eIR took place in three sub-counties (Teso North and Bunyala sub-counties of Busia county and Rongo sub-county of Migori county), involving 39 public and private primary care facilities, as well as the surrounding community health units, which served an estimated population of 348,598.

### Design

We employed a pragmatic embedded implementation research (eIR) to explore contextual challenges, and design and implement strategies to optimize the integration of PSBI management into the health system during the COVID-19 pandemic. Embedded implementation research is a systematic method of translating evidence-based interventions into routine health system functions through meaningful engagement of program managers and researchers in the co-production and use of knowledge for continuous program improvement [26, 27]. The success of integration is dependent on the well-thought-out design, implementation, and performance monitoring system. Accordingly, we facilitated a participatory exploration of challenges, the design of strategies to address these challenges, implementation and monitoring of strategies.

We utilized the WHO’s functionality model of community health programs [28] to assess the identification and integration of adaptive strategies into the health system: (1) participatory design and implementation, (2) health system response, and (3) performance perspectives. The participatory design and implementation were assessed in terms of joint problem identification, design of strategies, and continuous tracking of ongoing implementation challenges and adaptations of mitigating strategies for quality program implementation. The health system response was assessed through the changes in system support, technical oversight, and functional linkages between primary care facilities and community health workers, and integration of strategies into the district/county health systems work stream. While the performance of PHC facilities is measured in terms of identification and management of PSBI cases.

### Data and analysis

Both projects in Ethiopia and Kenya utilized the Reach, Effectiveness, Adoption, Implementation, and Maintenance (RE-AIM) [29] framework to measure the implementation and effectiveness outcomes of the projects and the Consolidated Framework for Implementation Research (CFIR) [30] to guide identification of barriers and facilitators to implementation and effectiveness. These frameworks were also used to design and guide COVID-19 adaptive COVID-19 strategies, identify implementation challenges, and make adaptations to improve the reach, adoption, fidelity, and uptake of community-based management of PSBI services throughout the project (Table 1). The project hypothesized that by incorporating strategies to increase PSBI treatment integration into routine newborn care at the community and facility levels through the eIR process, the quality of newborn care would improve. Table 1 below shows the definitions of the eIR outcomes.

**Table 1:** Implementation outcome measurements used in both Kenya and Ethiopia projects

Outcome	Definition	Metrics	Data source
Reach	The degree to which an intervention-eligible population receives it	• Number of sick young infants managed for PSBI (% infants eligible who receive PSBI care) • Proportion of SYI referred	Facility iCCM/PSBI register review
Effectiveness	The impact of an intervention on the uptake of PSBI treatment	• Improvement in percent of mothers/caretakers of SYIs who seek care from an appropriate provider	Before-after household surveys
Adoption	The extent to which project innovations are employed	• Number of facilities providing PSBI services when a referral is not feasible • Number of facilities and community-based providers trained in PSBI intervention • % of supplies in stock and percent of re-supply	Chart reviews and facility assessment and program monitoring data
Implementation	*Fidelity*: intervention or strategy was implemented as it was designed in an original protocol, plan, or policy or as adapted	• Implementation strength score: − % of HEWs/CHVs trained/mentored on PSBI − Mean percentage of materials/ equipment available − Mean percentage of supplies available − % of HEWs/CHVs supervised on PSBI − % of health posts /PHCs facilitated awareness creation meetings at the community level	Process evaluation (facility assessment, interview with service providers and program managers, facility iCCM/PSBI register review) and program monitoring data
	*Acceptability*: Perception of stakeholders (county and sub-county) about the contribution of the interventions to project results	• CHVs/Facility-based providers’ acceptability of introducing and implementing the IMNCI/PSBI intervention package in the context of COVID-19 • Barriers and facilitators to uptake of PSBI guidelines in the context of COVID-19	Process evaluation, interview with service providers, Before-after household surveys
Maintenance	The extent to which the intervention is institutionalized in both facilities and communities	• Trends in service uptake and rebound • Functionality of CHVs • Implementation strategies incorporated with the district work stream and integration • Feasibility of the strategies for national scale-up	Process evaluation (facility assessment, interview with service providers and program managers, facility iCCM/PSBI register review)

CHVs: Community Health Volunteers; iCCM: integrated community-based management of common childhood illnesses and newborn care; IMNCI: integrated management of common childhood illnesses; HEWs: Health Extension Workers; PSBI: possible serious bacterial infection; PHC: primary health care; SYI: sick young infant

We had a monthly cross-learning community of practice sessions between implementation research teams to provide opportunities for harmonizing research tools and methodologies between the countries. Accordingly, guided by these implementation research frameworks, both the Ethiopia and Kenya projects conducted mixed formative quantitative and explorative research from April-May 2021 and summative evaluations from June-July 2022, which involved facility assessment and interviews with community health providers and program managers. The end-line health facility assessment covered 65 health posts in Ethiopia [10, 13] and 38 primary healthcare facilities in Kenya. The qualitative component of the end-line study in Ethiopia involved in-depth programmatic interviews with 41 HEWs and their supervisors (Additional file 1). In Kenya, six focus group discussions with CHVs comprised 10–12 participants, along with 18 in-depth interviews with healthcare providers, and 12 key informant interviews (KII) with Community Health Assistants, community health focal persons, and IMNCI-focal persons.

For quantitative data, survey reports of the formative and end-line data were accessed from each project. Facility assessment and service statistics data were descriptively analyzed using Stata 15. In Ethiopia, an interrupted time-series analysis [31] was conducted to examine the rate of PSBI cases treated over time per 100 young infants (0–59 days old) within the catchment area of each health post. The monthly rate of PSBI cases treated was calculated as the number of sick young infants treated at health posts per month divided by the expected number of sick young infants. The expected PSBI cases were estimated using 3% live births and 10% prevalence of newborn infection in the catchment population. The pre-intervention period was from September 2020 to March 2021, while the intervention period was from April 2021 to May 2022. Time series models were fit (adjusted with the Cumby-Huizinga test for lags of autocorrelation) to examine the differences between pre-intervention and intervention periods in the rate of PSBI cases treated attributable to the intervention with counterfactual estimates of what would have happened without the intervention.

Qualitative data were analyzed separately using NVivo 11 and ATLAS.ti 7 software for Kenya and Ethiopia context, respectively, guided by the CFIR framework using a priori codes derived from the interview guides and the research questions related to fidelity, adoption, acceptability, and sustainability of the PSBI guidelines. Reports of quotations, themes, and patterns of interrelationships between the themes were identified from each project and compared across projects.

## Results

The results are presented according to the order of the WHO’s functionality model of community health programs framework: participatory design and implementation, health system response, and performance.

### Participatory design and implementation

In both countries, the formative assessment identified potential and active contextual factors that informed the implementation strategies including challenges and opportunities unrelated and related to the COVID-19 pandemic preventive measures. Common barriers to the effective referral of cases from the community to the facilities and delivery of PSBI treatment were identified (Table 2). The integration of commodity supply, community-to-facility referral, support systems, and technical oversight from district/sub-county health management offices to community health systems was found to be weak within routine newborn care in both Ethiopia and Kenya including PSBI diagnosis and management. In Ethiopia, when partner support phased out the iCCM program, particularly the PSBI component, PSBI management was not fully integrated into the health system. Additionally, Gentamicin 20 mg/ml, a critical antibiotic for PSBI treatment, was not included on Ethiopia’s national drug and commodity procurement list, presenting supply procurement issues.

**Table 2:** Implementation challenges identified and strategies co-designed in both countries

Challenges	Strategies
Mother’s fear of COVID-19 infection at health facilities and COVID-19 response measures (restricted mass gatherings, limited mobility, and restricted public transportation)	• Community mobilization • Integrate COVID-19 and routine services • Strengthen facility-level infection prevention and control practices
Stock-out of essential drugs and supplies	• Strengthen supply chain management • Redistribution of supplies, and • Advocacy for iCCM/PSBI supply integration
Weak support system	• Supportive supervision and coaching • Technical oversight from district/sub-county health management offices • Establish a technical support unit (Ethiopia only) • Adaptation of digital platforms to support data management and tracking of PSBI cases (Kenya only)
Weak referral systems between facilities and frontline health workers	• Strengthen community-facility referral link
Weak integration of support system, commodity supply, and community-to-facility referral within routine newborn care	• Advocacy for iCCM/PSBI supply integration • Advocacy for procurement of gentamicin (Ethiopia only) • Integrate PSBI management strategies into the district/county work stream (performance review, supervision, and annual planning sessions)

Following the formative work, each country project facilitated a participatory design of implementation strategies employing the adapted Expert Recommendations for Implementing Change (ERIC) protocol [32]. Through this process, we systematically engaged relevant stakeholders at both the national and intervention districts/county levels to contextualize and validate the preliminary implementation strategies. We selected priority implementation strategies, adapted existing implementation strategies, and developed micro-plans to improve the quality and delivery of PSBI treatment. Throughout the program implementation, we provided technical support to intervention districts/counties, assisting them in implementing and monitoring the adapted strategies. This support created opportunities for identifying solutions to critical gaps, enabled the development of work plans to address the gaps and to track progress.

During the implementation phase in both countries, we continuously documented ongoing implementation challenges and adaptations of mitigating strategies [10]. Additionally, we provided regular feedback to the program based on real-time data collected during routine monitoring and supervision activities. We conducted monthly data reviews and cross-learning community of practice sessions between the teams from Ethiopia, Kenya, India, the World Health Organization, Northwestern University, the Bill and Melinda Gates Foundation, and other stakeholders. These sessions, embedded within the implementation process, facilitated the sharing of experiences among implementing partners, researchers, and stakeholders.

### Improved health system response

These participatory design and implementation processes improved the PHC system capacity and ownership and facilitated the high-fidelity implementation of strategies. The PSBI treatment services received significant attention and engagement from various levels of the healthcare system in both Ethiopia and Kenya as a result of this eIR. Health Extension Workers (HEWs) in Ethiopia and Community Health Volunteers (CHVs) in Kenya played active roles in raising awareness, conducting surveillance, and providing care for sick newborns. District/county health offices, sub-county health teams, and PHC facilities collaborated closely to support the implementation of these services. This collaborative approach reported improved community awareness, strengthened surveillance, and better care for sick newborns. Overall, the involvement of multiple stakeholders is associated with the success of the iCCM/PSBI implementation efforts and the improvement of newborn health outcomes.

*“… during the last two years, we did not give priority to iCCM services. Even the register was left [empty]. We pushed or referred sick children to the health center. But now, we share [iCCM] service availability with the community and provide the service. For instance, in the last six months, I identified 17 young infants and assessed and treated five of them.”*–HEW respondent, Ethiopia.

“*We have IMNCI and now PSBI management. Most of these cases are done without hospitalization, they are done without injectables. Before that, somebody would be admitted to a ward and put on a dose of injectable. But now like pneumonia plus other conditions, we just use amoxicillin and they can respond to that. Now that is a policy change.”*– KII sub-County Focal Person, Kenya.

During the formative assessment, both projects identified gaps in the availability of essential antibiotics for managing PSBI. In response, the project implementing partners in Ethiopia developed strategies for supply chain strengthening. This included advocating for procuring necessary commodities and facilitating the redistribution of gentamicin 20 mg/ml across primary health care units in the districts as a critical supply item. In addition, during implementation, we ensured the availability and appropriate use of purchase request forms and bin cards at facilities and strengthened the capacity of health facilities to forecast, quantify and manage commodities and supplies levels.

In Kenya, the project team implemented similar strategies, advocating for a continuous supply of essential commodities for PSBI management within the project catchment area. County pharmacists were engaged and they committed to providing necessary drugs to support the management of sick young infants the project also focused on strengthening the capacity of health facility personnel in charge to effectively utilize existing commodities tracking tools more effectively.

**Fig. 1:**
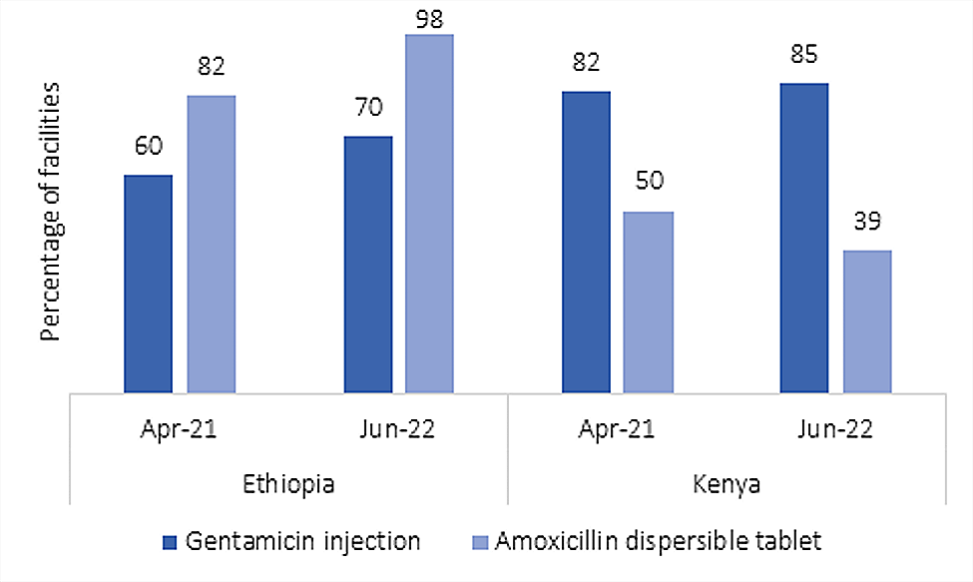
Availability of gentamicin injection and amoxicillin dispersible table

Despite the efforts, a significant number of health posts in Ethiopia and primary care facilities in Kenya experienced stock outs of injection gentamicin and oral amoxicillin, respectively, in the last three months preceding the end-line survey (Fig. 1).

In contrast, all health posts in Ethiopia and the majority of primary care facilities (health centers and dispensaries/clinics) in Kenya had iCCM clinical guides, chart booklets, and registers on the day of the end-line survey, demonstrating that implemented education and training strategies were effective at helping to sustain PSBI management guidelines.

#### Strengthened health system linkages, support systems, and technical oversigh**t**

In Ethiopia, a Technical Support Unit (TSU) comprised of trained personnel from health centers and district health offices played a crucial role in providing on-site training and coaching to HEWs for the implementation of iCCM/PSBI services. They conducted supportive supervision visits, engaged in discussions, provided feedback, and facilitated performance reviews and referrals. The project staff also ensured the availability and proper use of essential forms and cards at health posts. The HEWs acknowledged the support received from the project and health center staff, highlighting the positive impact of on-site coaching and supervision in improving their skills. One HEW from one district said, *“On-site coaching is quite helpful to demonstrate clinical skills on real clients.”*

Accordingly, HEWs preferred on-site coaching to offsite training, which is often conducted in a very short time to retain essential skills.

A district iCCM focal person said, *“HEWs were very happy. They accepted the support we provided to them. Most of them also recommend that we make a more frequent visit, and some of them call us when they encounter any challenges related to understanding the chart booklet.”*

In Ethiopia, the establishment of TSU also strengthened the existing health post-health center technical and administrative linkages.

In Kenya, the project took measures to ensure the performance and sustainability of IMNCI/PSBI initiatives by creating a pool of trainers who could continue imparting skills beyond the project duration. Refresher training on IMNCI/PSBI with a specific focus on PSBI and COVID-19 management and on-the-job mentorship was provided to providers. Additionally, training sessions were conducted for Community Health Assistants and CHVs to enhance their capacity to deliver quality essential newborn services within communities.

CHVs were trained to recognize danger signs, counsel families, and refer identified PSBI cases to the nearest health facility in a timely manner. The training covered various aspects, such as recognizing danger signs, facilitating referrals, ensuring treatment adherence, and understanding the appropriate dosage for PSBI treatment.

Feedback from focus group discussions with CHVs highlighted that most of them had received training on PSBI management as part of the iCCM/PSBI strategy for identifying and managing SYIs 0–59 days old in the community.

*“I was trained on iCCM and [PSBI]; I learned that pneumonia is an acute respiratory infection that affects children. We learned that if we visit a household, we should assess if a child has a cough, fast breathing, or chest in-drawing, which are possible signs of pneumonia. We were taught that if we visit a household and get a baby with chest in-drawing, we should refer them to a facility.” —* Focus group discussions CHV discussants, Kenya.

Community Health Assistants played a crucial role in linking communities to the formal health system, ensuring that every sick child and their caregiver had access to a CHV for support.

#### Implementation strategies integrated into the district/county health systems

During the PSBI eIR implementation in both Ethiopia and Kenya, we facilitated the integration of a support system into the district/county and health center workflow, which included performance review sessions, supervision activities, and work planning sessions. We advocated for the inclusion of gentamicin 20 mg/ml into the supply and logistics system. Based on key informant interviews conducted during the end-line evaluation, participants observed that implementation strategies were successfully incorporated into the community and PHC system, sub-county, and district operations, budgets, and procurement processes. Furthermore, these strategies were integrated into the weekly and monthly reports, regular checklists for supportive supervision, and performance reviews. These observations are promising and indicate that these strategies have the potential to be sustained and fully integrated with the existing health system, especially with continued supervision. Moreover, they can be scaled up with appropriate planning.

*“iCCM is integrated into our routine activities,… included in the annual plan and quarterly performance reviews and supportive supervision activities…it is included in the supervision checklist.”*–District Health Office Child Health Officer, Ethiopia)

*“We do as a sub-county. We do quarterly supervision of all the facilities. When we go out, we integrate all those services. There is a team looking at IMNCI/PSBI. There is a team looking at other things. We have checklists on personnel and their qualifications, commodities, equipment, and referrals. So, within these checklists, we have got PSBI items that we address.”*–KII sub-county Focal Person, Kenya.

Most key informants indicated that the project is sustainable without relying on external support and incentives as the existing health system took full ownership of its implementation with modest technical support from the implementing partners. Respondents, including healthcare providers and district/county health offices, expressed their commitment to continue their support. This commitment demonstrates their dedication to the project’s long-term success and the sustainability of its impact.

*“Our support will be sustained because it has already been integrated with the PHC support system. iCCM has been integrated with the [supervision] checklist, and support is being provided accordingly so that it will be sustained from now on, and we will continue this way.”* —Health center director, Ethiopia.

*“We have a plan. The plan was developed at the time of IMNCI. So, when PSBI came, it was a matter of strengthening it. One of the things we realized during the support supervision visits is the gaps we needed to address for that plan to succeed.”*– KII sub-county Focal Person, Kenya.

Key informants also expressed their confidence that the implemented strategies could seamlessly integrate within the existing health system, allowing for smooth and efficient adoption at a larger scale. Their belief in the compatibility of the implemented strategies with the current system suggests that the eIR project’s approaches are not only feasible but also have the potential to be widely replicated and expanded across a broader spectrum of healthcare facilities and regions. This positive outlook reinforces the project’s potential for sustainable impact and the possibility of reaching a larger population with improved healthcare services.

*“I think it is possible to scale up to other [districts]. It can also easily be integrated with the existing health system. There is also an established system regarding the health center- health post linkage and policy; if we can strengthen it, we can get better outcomes.”*–iCCM Focal Person, Ethiopia.

*“We realized there was a weak link between the community and the link facility, and it was cutting across the whole sub-county. We realized this gap and reviewed our plan on the need to strengthen that link”*– KII sub-county Focal Person, Kenya.

Both the projects in Ethiopia and Kenya leveraged national learning platforms, including technical working groups focused on child health, to disseminate and scale-up lessons from this implementation research. In Ethiopia, the MOH utilized the survey findings to inform their annual planning and forecasting for their 2014 and 2015 Ethiopian fiscal year (EFY) annual planning and forecasting of iCCM/PSBI commodities.

Similarly, in Kenya, the project adapted indicators for PSBI management into national tools and health management information systems (HMIS). For instance, the under-five supervision checklist was adapted to include newborn details. In addition, as part of the policy response, the MOH has incorporated management of SYI with PSBI where referral is not feasible into IMNCI and iCCM guidelines and in the Newborn and Child Health Strategy 2021–2025. Similarly, the MOH revised reporting tools for under-five children to include IMNCI/PSBI indicators in the DHIS2 platform. IMNCI programs and activities have also been incorporated into the Division of Neonatal and Child Health annual work plan. As a result, tools are available for tracking and monitoring PSBI management.

### Improved performance of PHC facilities in identification and management of PSBI

**Fig. 2:**
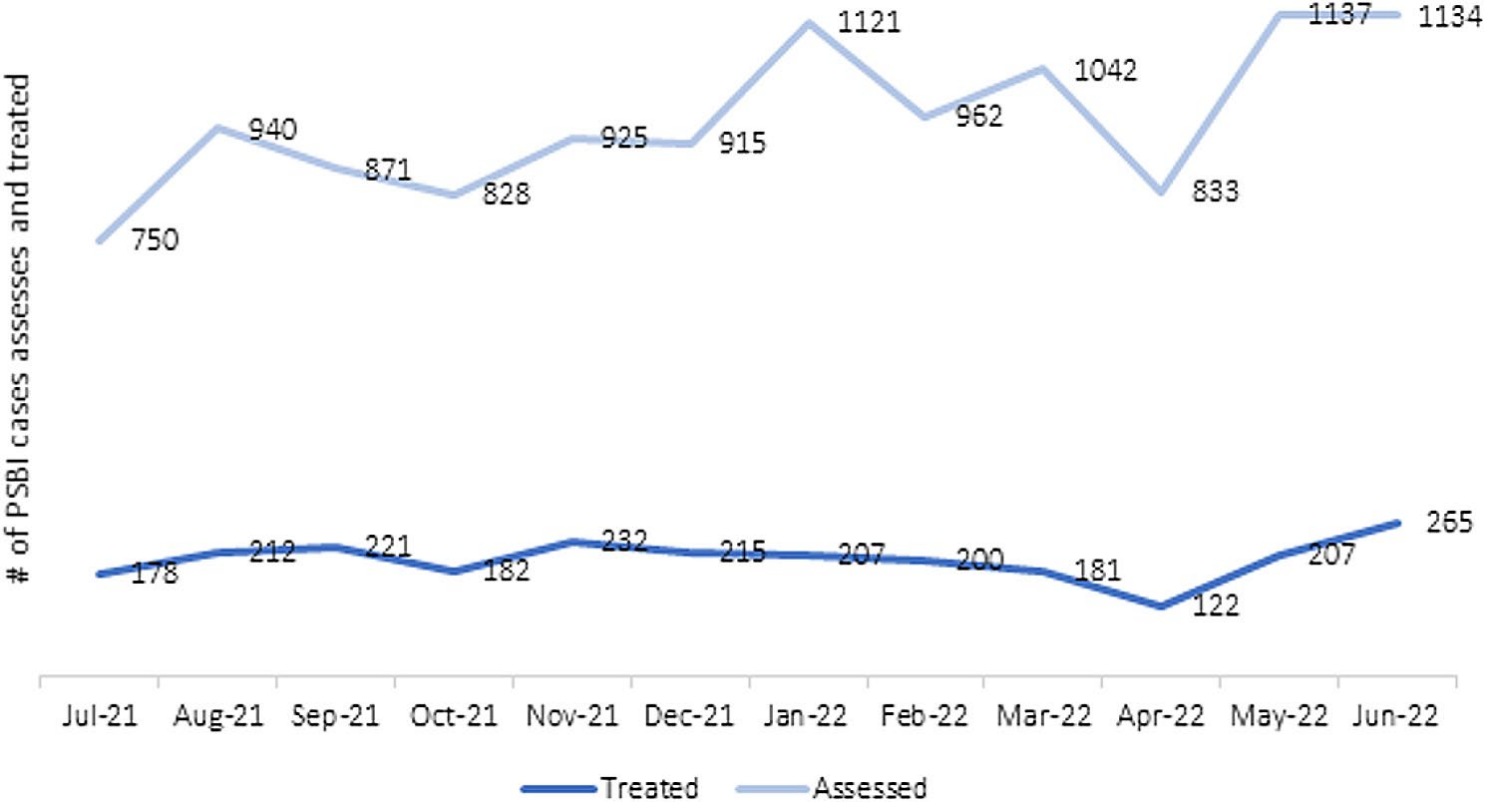
Number of PSBI cases assessed and treated, July 2021-June 2022, Kenya

Between April 2021 and May 2022, HEWs in Ethiopia made 32,428 home visits, about 35 per health post per month, and identified 3,099 newborn infants, equating to approximately three infants per health post per month. Accordingly, 84% of health posts treated at least one sick young infant in the previous six months. From April 2021 to May 2022, a total of 1,335 SYI cases were assessed by HEWs, representing an 85% coverage rate. Of which 230 PSBI and 223 local bacterial infection cases were treated by HEWs. In Kenya, CHVs assessed 10,187 newborns (0–2 months) for danger signs during July 2021-June 2022, representing an 81% coverage rate. Out of the 10,187 newborns assessed for danger signs by CHVs, 1,176 (12%) were confirmed with signs of PSBI and referred to the nearest PHCs. Data from service statistics showed an increasing trend in the number of sick young infants treated at PHCs over time, which suggests improved demand for SYI services in the community during the intervention period, July 2021-June 2022 (Fig. 2).

**Fig. 3:**
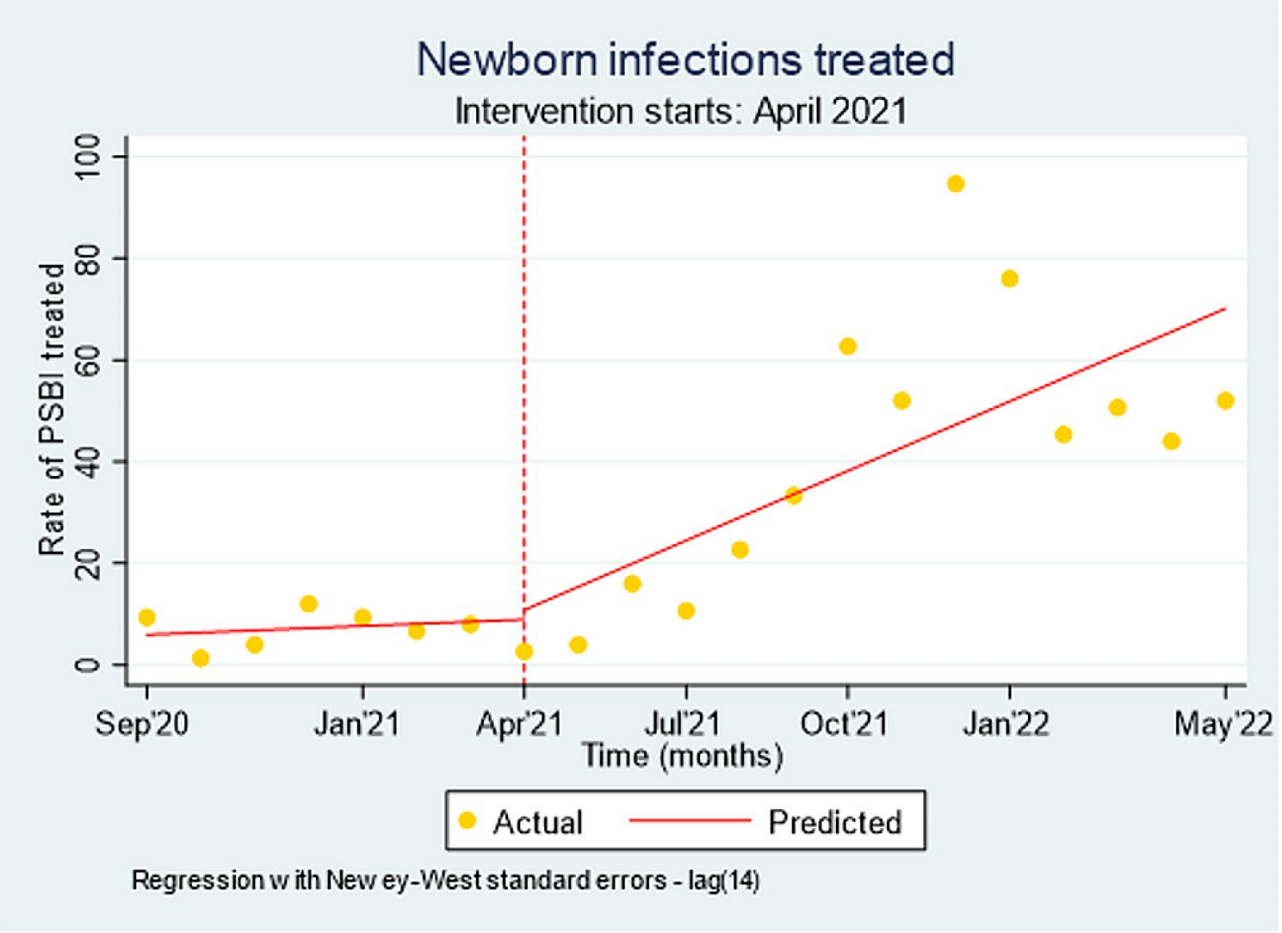
Interrupted time series analysis of the monthly rate of PSBI cases treated over time, September 2020-May 2022, Ethiopia

Interrupted time series analysis of the monthly rate of PSBI cases treated over time in Ethiopia showed that after project support commenced in April 2021, there was a 4.1% (95% CI: 1.36–6.90; p-value < 0.01) monthly rate of the trend of increase in PSBI cases treated by HEWs. As depicted in Fig. 3, there was a 4.6% (95% CI: 1.80–7.32; p-value < 0.01) annual rate of increase in PSBI treatment after April 2021, which is more than expected if the PSBI treatment strategies were not in place.

According to in-depth interview respondents in Ethiopia, the implementation of the adaptive strategies was helpful to (1) recover services interrupted due to the COVID-19 pandemic; (2) enhance the capacity of HEWs; (3) improve community awareness about the iCCM/PSBI service availability; (4) strengthen the PHC capacity to mentor HEWs; and (5) improve the care-seeking behavior of mothers for their SYIs.

Respondents mentioned that one of the perceived effects of this eIR was a recovery of routine services from the COVID-19 pandemic.

*“… the community, at some point, has stopped the service due to COVID-19. But then, we persuaded the community to resume using the service after a temporary halt.”* —iCCM focal person, Ethiopia.

*“To our delight, now everything is turning out well or having the intended result, and most community members are using the [iCCM] services at the health posts.”* —iCCM focal person, Ethiopia.

Other effects of this investment mentioned were enhancing the capacity of HEWs and strengthening the primary health care unit’s ability to support HEWs:

*“The success achieved in boosting the capacity of HEWs is worth mentioning. Generally, the support from JSI was immense in many aspects, including the training provided for our health professionals, which was unthinkable during the COVID-19 era. More importantly, the support we received equipped us with the means to improve the capacity of HEWs.”* —iCCM focal person, Ethiopia.

### Barriers to optimal integration of PSBI management guidelines

Despite the implementation of the strategies to enhance and generate demand for PSBI services, qualitative study respondents in both countries raised concerns about persisting barriers that hinder the optimal delivery of these services. These barriers include the inadequate supply of essential commodities, poor infrastructure, and heavy workloads attributed to the shortage of health workers. In Ethiopia, additional barriers identified were perceived low quality of service, lack of community trust, and challenges posed by civil conflict and regional state of emergency.

*“The community members usually have limited trust in the services at health posts.”***—**HEW respondent, Ethiopia.

*“… the first challenge is related to the shortage of supplies and medicines. Especially the shortage of drugs has been a serious challenge for us to provide the iCCM service.”* —iCCM focal person, Ethiopia.

In Kenya, negative community perceptions, geographic inaccessibility, and lack of transportation were cited as significant obstacles to the optimal integration of iCCM/PSBI treatment. Lack of supplies and tools such as the referral form, malaria test kits, and thermometers were also mentioned as major challenges to the community-facility referral. CHVs obtain supplies from the facility they are linked to, however, sometimes, facilities experienced stockouts of supplies for up to three months.

*“Yeah, distance is also a challenge, for example, a client from the Igigo area, which is in the interior, sometimes there is a lot of water in the middle so, for them to pass through the water and get here, sometimes it becomes difficult. From the perspective of the community, a CHV can get a sick child and refer them then sometimes this client may not get there as they will tell you they do not have transport, and yet you have already written them a referral. That is already a challenge. So, you get such a client and refer them, and at times, before they walk from there to here, it is a challenge for them, and so you will find that maybe they will keep postponing the visit, saying they will go the next day.”* —KII, Kenya.

#### Shortage of health workers and workload

Shortage of health workers and heavy workloads were also reported as significant challenges for delivering iCCM/PSBI services in both Ethiopia and Kenya. The most commonly occurring challenge to human resources was staff attrition– where trained staff are transferred, hence the need for constant training of new staff on PSBI management. While in Ethiopia, the health posts often closed when HEWs conducted community and outreach-based services. One respondent (HEW respondent in Ethiopia) said.

For instance, in this health post, as you can see, I am alone, and when there are campaigns like for the trachoma treatment, I stay on the field for ten consecutive days. So, the health post will be closed during campaigns and other activities. Closure of health posts affects the health service utilization of community members as they become upset when they get the health post closed.

#### Civil conflict and other competing priorities

District health office respondents reported that the regional state of emergency and conflict in the northern part of the country indirectly affected iCCM/PSBI service delivery.

*“…if I mention the war, nobody was concerned about the community’s health. Many health workers from our health center were sent to the war front to care for the injured fighters and civilians. The health posts were closed since the HEWs were participating in collecting food items from the community. Therefore, the war had disastrous effects as far the provision of medical care to the community was concerned.”* —iCCM focal person, Ethiopia.

During implementation, both countries continuously tracked implementation challenges and developed mitigation strategies including intensified support systems and organized additional need-based training for frontline health workers and their supervisors.

## Discussion

This embedded implementation research employed a systematic participatory design to identify barriers and strategies, implementing these strategies and monitoring processes to expand coverage of PSBI management to the outpatient level in the context of the COVID-19 pandemic in Ethiopia and Kenya. We found that this research was associated with strengthening the oversight of district and county health systems over community and primary health care systems, fostering collaborative learning across primary care facilities and community health workers. We saw improved bidirectional linkages between communities and health facilities, and the overall performance of the health system in delivering PSBI treatment.

Our study highlights the importance of meaningful engagement of stakeholders in planning, contextualizing, and prioritizing implementation strategies to translate evidence-based interventions into practice, and to inform policy recommendations for sustained health benefits as highlighted in the field of implementation science research [32, 33]. Both the Ethiopia and Kenya projects engaged various stakeholders throughout the design and implementation process to optimize the integration of PSBI management into the community health system [26, 32]. This involved a rigorous and thoughtful development process, including a national stakeholder consultation using an adapted ERIC protocol and a modified Delphi approach [32] as well as co-creation at district and county levels. This participatory approach facilitated the uptake of research findings for quality programming, ongoing adaptation of the program, integration of strategies into the district/county work stream (performance review, supervision, and annual planning sessions), and the supply and logistics system.

The participatory design and implementation of strategies resulted in positive, tangible aspects of delivery and uptake of PSBI treatment during the pandemic. This approach effectively engaged community volunteers, front-line health workers, and the primary health care system, leading to improved integration of PSBI management and facilitating sustained implementation. Previous studies have demonstrated that integration increases coverage and access to essential health services [34]. Similarly, implementation research in low-income countries has shown increased coverage of PSBI management through use of strategies such as front-line healthcare worker training [35], community engagement [16, 35], strengthened supply chain management [35], and provision of technical oversight [16, 35].

Both projects in Ethiopia and Kenya supported and strengthened the integration of PSBI management across the continuum of community and primary healthcare facilities. During implementation in both countries, strategies were implemented to strengthen the supply system and technical oversight of district/county and primary healthcare facilities’ work streams. As the community health system serves as an intermediary unit between the community and primary care facilities within the district or county health system, establishing strong relationships with stakeholders from both the community and the health system is critical for optimal service delivery. Strong technical oversight and accountability mechanisms were found to foster community trust, effective communication, and positive relationships; all of which contribute to improved health system performance [36]. In Ethiopia, the established technical support unit, (consisting of iCCM-trained personnel from the under-five clinic of health centers, health center heads, and district child health officers), played a key role in coordinating referrals, providing feedback to HEWs, and ensuring technical and managerial oversight to bridge the fragmented care continuum. Similarly, in Kenya, the county and sub-county health management teams, along with health workers, coordinated referrals and provided a technical support system between community volunteers and primary healthcare facilities.

To ensure successful implementation and optimal integration of management of PSBI within the health system, several critical factors need to be addressed. These include providing continued coaching and support systems for frontline health workers, ensuring technical oversight from the district/county health teams, and maintaining an adequate supply of essential commodities. Supportive supervision has the potential to improve the performance of community health workers [37, 38]. However, to overcome the remaining implementation challenges, certain priorities should be given attention. These include introducing a participatory, community-health system continuous audit and feedback system to assess the quality of PSBI management, continuous supply of essential drugs, and implemented strategies to alleviate the workload of front-line health workers, thus enabling them to deliver quality services.

Our study had several limitations inherent to the post-hoc multiple case study design and cross-country comparisons [39]. There were differences in methods and scope of data collection and analysis between Ethiopia and Kenya. The synthesis and interpretations of findings were also limited to certain common variables and contextual factors. This limits the comparability of the findings across countries. The cross-learning community of practice sessions helped standardize the methods, process of data collection, and analysis across contexts to compare variations in implementation processes and influences across contexts which partly mitigates these limitations. Lack of comparison group and pre-COVID-19 data are additional limitations of the study so we are unable to determine causality rather than association.

This participatory and pragmatic implementation research application in Ethiopia and Kenya proved beneficial in identifying contextual implementation challenges within and across the two settings and in designing and implementing effective strategies. It also facilitated the standardization of processes, approaches, methods, and metrics, as well as the extraction of lessons learned to optimize the integration of PSBI management during the COVID-19 pandemic when referral was not feasible across country contexts. The lessons learned from these two projects were valuable in capturing generalizable knowledge to support PSBI management scale-up in the respective countries and to inform implementation efforts in similar resource-constrained contexts to reduce neonatal mortality rates, particularly in times of health shocks like those observed during the COVID-19 pandemic. However, evidence regarding the effective integration and sustainment of evidence-based interventions and policies into the health system is limited [37, 40]. This research provides insights into this body of literature. Nonetheless, it is important to consider economic factors that influence the utilization of implementation strategies for delivering and sustaining evidence-based practices [41]. Further economic evaluation and sustainability research on these implementation strategies is highly recommended.

### Electronic supplementary material

Below is the link to the electronic supplementary material.

Additional file 1: In-depth interview guides.docx. These are interview guides for service providers.

Additional file 2: End-line qualitative survey dataset.pdf.

## Data Availability

The quantitative data used for this study are available as supplementary information from previously published articles [10, 13]. The qualitative data from the end-line is presented as supplementary information (Additional file 2). Program reports used for this study would be available at request from the corresponding author.

## List of abbreviations

BMGF: Bill & Melinda Gates Foundation
CFIR: Consolidated Framework for Implementation Research
CHV: Community health volunteer
eIR: Embedded implementation research
COVID-19: Coronavirus
ERIC: Expert Recommendations for Implementation Change
HEW: Health Extension Worker
iCCM: Integrated community case management of childhood illnesses and newborn care
IMNCI: Integrated management of neonatal and childhood illnesses
JSI: JSI Research & Training Institute Inc.
KII: Key informant interview
PHC: Primary health care
NMR: Neonatal mortality rate
PSBI: Possible serious bacterial infection
RE-AIM: Reach, Effectiveness, Adoption, Implementation, and Maintenance
SYI: Sick young infants
WHO: World Health Organization
