# Supplementary material for: Optimizing integration of community-based management of possible serious bacterial infection (PSBI) in young infants into primary healthcare systems in Ethiopia and Kenya: successes and challenges
Source: BMC Health Serv Res. 2024 Mar 5;24:280. doi: 10.1186/s12913-024-10679-9 (PMC10916061; doi:10.1186/s12913-024-10679-9)
Supplement: Supplementary file 1 — Additional file 1: In-depth interview guides.docx. These are interview guides for service providers. [file 12913_2024_10679_MOESM1_ESM.docx]

### In-depth interview guides for service providers (Health center staff and HEWs)

**End line evaluation of the PSBI implementation research (IR), qualitative component**

| **I** | **Identification** | |
| --- | --- | --- |
| 1 | Questionnaire ID |  |
| 2 | Area Identification |  |
| 3 | Name of Woreda/Zone/Region |  |
| 4 | Name of facility |  |
| 5 | Name of moderator |  |
| 6 | Name of a note taker |  |
| 7 | Date of discussion |  |
| 8 | Participant # |  |
| 9 | Audio File # |  |
| 10 | Start time: | **______:________** |
| 11 | End time: | **____:______** |
| 12 | Transcriber |  |

| **SN** | **Questions** | **Probe** |
| --- | --- | --- |
|  | **Fidelity** |  |
| 1 | Can you please describe the demand generation/SBCC activities you are doing on iCCM/ newborn health? What are the strategies used to raise awareness? If none, please explain why.  How is the engagement of communities/WDA networks in the implementation of integrated community-based case management (iCCM) of common childhood illnesses/possible serious bacterial infection (PSBI)? | What are some of the biggest challenges about SBCC activities for newborn care? |
| 2 | How do you describe the strength of the support you got from the PHC and the project as well as your efforts to address barriers to deliver iCCM/PSBI during COVID-19? | What was changed?  Was the support you got from the project and PHC helpful?  What could have been done differently? |
| 3 | Did do you get the support system helpful (mentorship and on-site coaching methods, supervision, technical support, PHCU level PRCMM, etc.) to enhance your skills [that is HEWs’ skills] over the traditional off-site training and woreda level review meetings? | **Probe** for acceptability of the strategies (**for HEWs only)**  How could the support system be improved? |
| 4 | How eCHIS implementation helps you with iCCM service delivery? | **Probe for advantages:** case identification? quality of iCCM case management? retrieval of client records? data quality? client appointment scheduling and/or defaulter tracing?  **Ask why?**  **Probe for areas of strengthening?** |
| 5 | How was the introduction of the iCCM/CBNC module into eCHIS affected the iCCM/CBNC service delivery?  **For Lume woreda only** | **Probe for advantages:** identification and registration of sick newborns; clinical and referral decision support; follow-up of sick child treatment and/or defaulter tracing; retrieval of client records; appointment scheduling; client satisfaction  **Ask why?**  **Probe for areas of strengthening?** |
|  | **Implementation challenges** |  |
| 6 | Describe the main issues faced by the health system to identify and treat neonatal infections in the community. What are the critical factors affecting the **delivery of iCCM** to clients? | **Probe**; lack of competence of HEWs, shortage of supplies and commodities; weak support system; low community demand?  **Regional/national state of emergency and conflict in the northern part of the country?**  How has that changed during COVID? |
| 7 | How do you think the COVID-19 pandemic and/or COVID-19 response measures affect the uptake and/or delivery of iCCM services? | **Probe:** Has COVID-19 affected your daily routines; your work on newborn; the community in terms of livelihood and vulnerability for newborn care-seeking  How has that changed over time |
|  | **Adoption and reach/effectiveness** |  |
| 8 | Explain to us any changes you observed as the result of the implementation of the COVID-19 adaptive iCCM implementation strategies? – What impact has the strategies had? What are the particular features of the strategies that made a difference? | **Probe** for support system and linkages, motivation and competence of HEWs, community engagement and awareness creation?  What do you think are the reasons for non-significant changes? |
|  | **Maintenance and sustainability** |  |
| 9 | What are the high-level benefits that are attributable to this support/IR? |  |
| 10 | Please explain to us the feasibility of this support/ IR for national scale-up? | What features could easily be integrated into the existing system? Which not? |
| 11 | How are the activities/efforts embedded in the PHC and woreda routines/work streams? What implementation strategies are incorporated with the PHC and woreda annual work plan? |  |
| 12 | Do you have anything think is important to tell us that we have not asked you? |  |

**Thanks!!**
