# Supplementary material for: Optimizing integration of community-based management of possible serious bacterial infection (PSBI) in young infants into primary healthcare systems in Ethiopia and Kenya: successes and challenges
Source: BMC Health Serv Res. 2024 Mar 5;24:280. doi: 10.1186/s12913-024-10679-9 (PMC10916061; doi:10.1186/s12913-024-10679-9)
Supplement: Supplementary file 2 — Additional file 2: End-line qualitative survey dataset.pdf. [file 12913_2024_10679_MOESM2_ESM.pdf]

End-line evaluation of the PSBI implementation research, qualitative interview transcripts

Area Identification Amhara region

Name of facility HP

Date of discussion 16/6/22

Start time: 11:00 AM

End time: 12: 21 AM

Can you please describe the demand generation/SBCC activities you are doing on ICCM/ newborn health?

We pass on information to the community during religious service in a church. We also go from house to house to communicate with the community about the neonatal health care activities underway in the health post.

We have communicated with the kebele leaders and some of the Women Development armies/ WDAs/ about the presence of the neonatal health care service in the health post. However, the commitment of WDAs is not the same as before.

What kind of message did you transmit to the community in the church during religious service?

We have informed members of the community of the presence of treatment for newborn and under-five children and to bring newborns and children under-five to the health post to get treatment. And we told them the type of services and medication which are available in our health post and those children who are affected by severe illness are referred to the health center. When we go for PNC we tell the mother about danger signs of diseases, how to carry/handle/a newborn baby, and how to feed breast.

Why is WDA's commitment not the same as the previous time?

In our Kebele WDAS and the Kebele leaders don't work actively because the high turnover of Kebele leaders has affected our activities to some extent. WDAs are not working at the moment because members of the group spend much of their time engaging in their private matters and associate every activity with incentives. They say we worked for many years so we are no longer interested in giving service to the community.

How is the engagement of communities in the implementation of integrated community-based case management (iCCM) of common childhood illnesses/possible serious bacterial infection (PSBI)?

The community knows about the services being rendered/ICCM service/. But, they need additional information about the newly started new born health care. In past the health post did not apply medical care to cure new borne illness. We used to refer the majority of neonatestoo the health center because we were not provided with inputs to be used by new born child such as Gentamicin and amoxicillin. These medication were not allowed to be given in the health post. But now we are capable to treat new born health problem and we get all required supply and the community brings their newborn baby when sick to get the service. Greater number of people get child care service here in the health post. Currently, there is no scarcity of in inputs in the health post.

Do you think members of the community are feeling pleasure to obtain child care service in the health post?

Yes, they are. They devote their time, energy and money to go to the health center. People who have no money are not able to meet the cost of transportation to go to the health center and back home. Additionally, the community is getting treatment in the health post for free.

There are community members who have no health insurance. These people are please of accessing the service for free in their nearby.

People who live a great distance away from the health center were facing a lot of problems. After we started to give treatment to a considerable number of people here, we managed to make their hardship (wastage of time, money, their power) or pain more bearable or less severe.

Did you receive any support from other people to aware the community or to bring SBCC?

The health workers at the health center inform the community to acquire and use child health care service in the health post. They are telling them that a number of illnesses that used to be referred to the health center are given by the health post now. Also kebele leaders and WDGs give us assistance to transmit information to the community. Health workers check the registration book to evaluate how we treated neonates and children above 2 months of age.

Please elaborate the kind of supports given by kebele leaders and WDGs

Members of WDGs group make a report about the number of women who are pregnant and delivered a child. The kebele leaders give the latest information to the community relating to child health service given by the health post.

Was there any setback that delays progress in the transmission of information to the community?

Not all WDGs were having dedication to duty. So, not all women who delivered a child were known especially catchment area which are far from the health post. WDGs would let us know the condition if they were fully involved in the activity. WDGs and kebele leaders did not do a satisfactory job to that effect. Because of this there might be mothers and their baby who didn't get the necessary health service.

But, we have tried to educate the community and to give the service to the best of our capacity despite heavy workload. We aren't able to reach each individual in the community and to provide them each service because of the work load. We get information about the condition of the women late if they live a great distance away from the health post. Owing to these circumstances, we are not able to give care service timely. Sometimes we heard about the mother/baby who was needed health care service after long period of time.

Members of WDGs are not willing to do their job without incentive in return for the service rendered to the community. They give their own activities priority treatment. I think it is important to give them some money that encourages them to do the job. Previously they were initiated getting some amount of money due to different activities like vaccine campaign and the like. Now rather than providing training and some incentives to HEWs it is better to give and strengthen WDAs. Birr 50 or Birr 100 has a great value for members of WDAs and they will be willing to participate in any activities.

You mentioned that not all members of the community have the opportunity to make use of the services offered by the health post/HEWs. What were the obstacles to make the community gain access to the service?

Topography and geographical condition of most areas are mountainous. Besides, there is too much work to be done at same time. This year we are implementing a number of campaigns (Polio, COVID) and regular and common package activities to achieve a goal. Our responsibility is not only ICCM and CBNC. There are many other activities in addition to it. The vaccination of COVID negatively affects other routine activities. The community associates other health care services with COVID vaccine.

Addressing the challenges posed by coronavirus pandemic is one of those actions. Also, many people attribute COVID-19 to punishment of divine power.

This way of thinking has made progress difficult. Some people refused to get their child vaccinated against the polio virus. In addition last year the political situation in the country has prevented progress. Every member of the community focused on resource mobilization

Everybody used to talk about the deteriorating security situation of the country with others. But now the situation has returned to normal.

As you said your focus was on other issues (resource mobilization) related to the conflict and political situation. How did this affect ICCM/neonatal health care service delivery?

The conflict took our attention away from what we were doing or thinking. Therefore, it was difficult to give focus to our routine including ICCM and neonatal care and treatment in 2012. But, this year everything is oaky. Our movement was restricted by the conflict. Everybody was prone to psychological problems. Feeling of instability and insecurity made us not to be motivated.

How did vaccination given against COVID-19 affect delivery of ICCM and newborn health care service?

Vaccination against polio virus, vitamin A supplement and antihelimenthic for dewarming was given to children under-five. But the community is doubtful of vitamins, nutrients, vaccine, drug, or other fluid introduced into the body using a syringe or those administered by mouth. Great number of people refused to get their children inoculated against polio. They assumed that the COVID vaccine would come in the form of oral droplet.

What other challenges did you face to increase the level of awareness of the community? WDAs are not strong enough to carry out their duty properly. Also lack of devotion on the part of kebele leaders and high turnover / change in kebele leaders / have adversely affected our activity. The kebele leadership should be exemplary / model for other people.

Occasionally, we are ordered to do something additional or unexpected work from the Woreda.

How do you describe the strength of the support you got from the PHC and the project as well as your efforts to address barriers to deliver ICCM/PSBI during COVID-19?

The heath center gives us every kind of support. Health workers that work for the health center are going to the community together with our HEWs to teach them not to connect protection of newborn health and similar other activities with coronavirus pandemic.

We are providing ICCM and neonatal health service while strengthening house to house visit during COVID pandemic.

To get rid of worms from a child/de-worming/, inoculate a child against polio or measles and provision of vitamins to a child are part of our usual activity.

We always give knowledge to the community about these issues of concern. We also teach the community about COVID-19. But most people associate the pandemic with politics or religion.

How did you implement ICCM after the emergence of COVID-19?

A number of institutions discontinued their activities following the onset of COVID-19. But, we kept on carrying out our usual / normal activity. Coronavirus pandemic did not have an immediate and strong effect on our routine. But, we were very busy giving vaccine to children.

Likewise, the community was suspicious of any drug whether it was given by a syringe or taken by mouth. They believed that all types of drugs contain coronavirus. However, they continued to obtain medical treatment except inoculation.

What action did you take to change the way of thinking of the community?

We have told them that the vaccine has nothing to do with coronavirus. We have informed them that the drug is administered to stimulate the body's ability to resist a disease.

Did PHC or the project apply any other method to support you regarding the use of ICCM/neonatal health care services to motivate or inform the community to get for their child during COVID?

The health center promised to provide us with display board that contains all services which is given for children less than 5 years. But, their promise did not become real and I don't know why they didn't able to make it.

Did do you get the support system, mentorship and on-site coaching methods, supervision, technical support, PHCU level PRCMM, etc. helpful to enhance your skills when you compared with traditional off-site training and woreda level review meetings?

Newborn / less than two-months old/ and children under-five get different types of services. Health professionals from the project and PHC critically looked at the guide booklet how we classify and treated. If we committed any mistake, they would tell us to remove same. They showed us how to correct the mistake.

Gashe Zemed from JSI came here two or three times to give us essential support at each health post. Besides he conducted a discussion with all the health posts under the health center having the guide booklet. He made us to exchange the booklet to each other. Then assessed and evaluated whether the classification and the treatment were done following the right way or not. He gave us a case scenario of age less than 2 months old, the temperature and breathing of the baby, and observed while we were classifying the type of health problem that the child had and its treatment correctly or not. He did the same way at the health post.

Were people coming here from the health center to give exclusive attention to ICCM and neonatal care?

No, they were not. They were coming to show us a lot of things including but not limited to ICCM and neonatal care. They look focusing on the guide booklet displaying detailed information about neonate and children less than 5 years obtaining the type of treatment, dose and how we classified their problem.

Do you think the support you were given by them has improved your capacity / skill to carry out productive work?

Yes it has.

How?

People have different level of knowledge. The general awareness or possession of information and facts vary from one person to the other. Therefore, when I make a mistake, anyone with a better understanding of the issue can help me remove my errors.

We started to record a newborn in writing after Gashe Zemede came here but not before. We were thinking that information about children who were less than two months old was to be recorded only when they got sick. But, recently we write each information about new born baby less than 2 month. Ato Zemede made everything easier or possible for us all by removing the errors from our data.

What is the use of keeping data for a new born child who is not sick?

Firstly, we can easily learn whether the baby is sick or healthy. Keeping the data of the new born baby during postnatal visit helps to follow and make further investigation about the condition of the child and to provide the necessary vaccine and other supply.

The child might get sick after we start to keep all information about him/ her. If he gets sick, I will give him medication to restore his / her health.

What is your reaction / response to the support you have been getting from PHC and the project for the last one year? Did you feel happy?

I feel very happy. But I have no information about the feelings of my workmates.

What makes you happy about the support?

Because the support will help us carry out our task properly and remove our errors. If there is no any follow up or support, we are not able to be aware of our mistake. We will act as if everything we do is correct or true. A follow up is more useful than a training given to a great number of people in a big room. During training we may not be able to grasp the meaning of the information being communicated. A follow up can easily reactivate or refresh my memory.

There were times that we didn’t change the training into practice. This made us to forget the content of the training and results in doing a mistake in the life of service user. The occurrences of such mistakes are resolved when supported by mentorship and on job coaching. This way of support helps to remember content of the training and lead us to perfection while getting correction.

In your opinion what needs to be improved when the experts watch over your activity and give you support?

Currently Gashe Zemed has stopped to give us support. The support should be continued as before from the health center and the woreda health office. We shouldn’t expect support from another organization regularly/permanently.

The project does not have sole responsibility to assist us. PHC/Woreda health office should organize a review meeting on a quarterly basis. To upgrade our knowledge and skill, the period of time between one event / review meeting/ and the next should not be long.

How does ECHIS implementation help you with ICCM service delivery?

ECHIS implementation on ICCM and newborn treatment has not started yet. Last time Gashe Zemed promised to choose the right drug from others after we are trained and the program / ECHIS/ is loaded.

We will gain a great deal of benefit if they insert or load the ICCM/new born treatment guide into our mobile phone/tablet/. We need not look into the guide booklet and use our time ineffectively to choice the medication to be recommended for a sick child.

The mobile phone will by itself choose the necessary drug to be administered to the child when we fill the sign and symptoms the child has and make the arrangement to meet same. No need of revising the guide booklet to search and find the right treatment. The computer identifies the disease of the child, its treatment and appointment when inserted/filled the required information. As Gash Zemed said, “It will be implemented in near future and makes your work easy”. But we have not seen it by our eyes because it is not inserted in our mobile phone.

Currently you are implementing ECHIS. How does recording of the information facilitate/contribute for the implementation of ICCM?

It lets us easily know the size of target population / number of pregnant women, number of children born recently / less than two-months old/ and number of children under-five.

Implementation of ECHIS will help obtain the necessary data without any difficulty. It makes to access individual data easily compared to finding their data from a chart. We can get information back even if our mobile phone is lost or stolen. But, we cannot get back information contained by a paper. The information registered at health post level is connected at Federal, Woreda and PHC level so possible to restore back the data from them.

How ECHIS system helps to meet the patient or service seeker at the appointed time and identify a defaulter/ non-attendant/ and to come back to the health post?

Let me cite child vaccination as an example. If the child is not inoculated today, not registered after giving the vaccination, and default the vaccine, the system put in place will give out a red color. Therefore, the system will remind me to give vaccine to the child if he/ she was not vaccinated before. It also informs that the appointment date is approaching by showing yellow light one week before the appointment. Green,yellow and red light help us to identify those who take health care service appropriately and missed or default their appointments.

We may find to apply the technology difficult until we acquire a complete understanding of it. But, after we successfully mastered the technology, we can easily find out our information. After that we are not required to submit a report to the health center. They can get every data from the mobile phone without any difficulty. But, it will take some time to learn the skill. But communication link between telephones is weak and takes long time to

save the data. Thus, information may not be mutually accessed or exchanged easily. It takes a lot of time to write the name of one household head.

Describe the main issues faced by the health system to identify and treat child/neonatal health problem?

WDAs are not fully involved in their activity. Because of this reason it may not be easy for HEWs to identify and treat neonatal infections without delay. The child may be at a risk of losing his/ her life if we fail to give treatment on time. We will be able to do a lot of work if WDAs are strengthened and fully involved in the activity. They can provide us with useful information about the general wellbeing of the community.

The number of HEWs and children seeking medical care are not having the correct proportion in quantity. So our superiors should consider of hiring additional HEWs to provide quality service.

What other factors affecting the delivery of ICCM to clients?

The mountainous nature of the area is also a source of problem. There is also scattered pattern of settlement of the population. The households are located a great distance away from each other. We have to climb up and go down a hill or a mountain this makes difficult to reach each community besides the aforementioned problem.

Did you face any problem to identify a sick child/ newborn who is less than two months old /and give it treatment?

We have no problem to identify health problem of a two-month child. But, as regards treatment, we may confuse in identification of cases. We mistakenly identify one case as the other. For example, we may fail to refer to the health center a child affected by severe illness and treat it in the health post.

Which sign that indicates the presence/absence of server disease do you find difficult to identify when you examine a child?

It is difficult to identify whether the baby has convulsion or not. In the guide there is a question and it says does the baby have convulsion? Identifying and answering this question is confusing.

What other problems did you face when examine and treat a child?

Gentamycin 10 ml preparation was easy to give the right dose but not available anywhere and substituted with 80 and 40 mg preparation. They told to us how to dilute and calculate but confusing and difficult to calculate the required dose to each baby.

Were the state of emergency and armed fighting between the government and the rebels having an effect for the delivery of ICCM service and identification and treatment of neonatal cases?

It was difficult to refer mothers to other places because of lack of transport. Everybody's movement was restricted by the state of emergency. Only ambulance was allowed for carrying people to and from the hospital. But, not all people requiring treatment gained access to the service provided by ambulance.

We were not able to go to the market to buy foodstuff or edible substances. Lack of transportation has remained a problem even after the state of emergency was lifted.

Was there shortage of supply of inputs to carry out your task?

No

Was there any lack of interest by the community to obtain service?

Members of the community are very slow to understand the message delivered by HEWs. Some households are not willing to bring their sick child to health facility. They believe God will cure their child from his/her illness.

Did you face any other problem?

No.

How do you think the COVID-19 pandemic and/or COVID-19 response measures affect the uptake and/or delivery of ICCM services?

The majority of the people argued that there was no COVID-19. They were not scared of being infected by the pandemic. It was business as usual for the community. They used to come to health facility to get service.

How did COVID-19 affect your routine?

We were scared to come to health post to carry out our task because of COVID-19. It was difficult to go to the community because the people believed that we were infected by the virus.

What did you do to make changes?

We tried hard to impart knowledge to the community about the mode of transmission of COVID-19 from one person to the other. We also took the necessary precaution to protect ourselves from infection by COVID-19 by wearing a face mask and using sanitizer and practicing physical and social distancing.

Did COVID-19 have any effect on the delivery of medical help for newborn and children under-five?

No, We managed to organize pregnant mothers' conference by allowing space between participants. In addition, when they came to the health post to get service, only two or three people were allowed to enter a room at same time.

What are the high-level benefits brought about by the support /IR?

In past or before the support there was a likelihood of ordering the wrong type of drug to a patient. Actually, we were making a lot of mistakes to that effect. But, the support has provided us with a means to go to the community to offer appropriate service.

This condition gave us the confidence or courage to do our job successfully. There is a change now. The support has created us every opportunity to deliver a good quality service for the community.

The support has provided us a means to diagnose or identify illness in a patient and its treatment. But, our achievement rate is not one hundred percent because not all newly born have gained access to treatment.

Please explain to us the feasibility of this support/ IR for national scale-up?

The support must to be given at a national level uniformly. No region must be left out from the program.

Which activities are easy and which ones are difficult to implement at a national level?

Examining and treating neonate and children less than five is easy to implement if it is possible to address different barriers that I mentioned before.

If the Woreda health office and PHC are committed to conduct the mentorship and onsite coaching in the absence of other body, it is possible to provide ICCM and neonatal health care services. Empowering the HEWs in knowledge and skill through on job training and supportive technical supervision is a prerequisite for quality service provision.

It is difficult to implement the activities in areas where there is a conflict. Nobody is willing to be assigned in an area where armed fighting takes place. It is possible to perform productive job if the activities are implemented in an area which is peaceful / nonviolent.

Our area is very peaceful. In areas bordering Tigray region, we cannot think of implementing the program. Nobody is willing to be assigned in an area disrupted by war.

How are the activities/efforts embedded in the health post work streams?

When we go to the community we take guide booklet and other kinds of items with us. If we find a newborn, we will give him treatment. We take thermometer, weighing scale, evidential or reference documents and guide booklet etc. we also take essential drugs such as gentamicin, amoxicillin, zinc and ORS with us and provide accordingly.

What implementation strategies are incorporated with the PHC and woreda annual work plan?

We communicate information to the community about newborn, COVID-19 etc. We incorporate ICCM and neonatal care with other activities. They are made part of our quarterly and annual plan of action. We give

exclusive attention to neonatal care. We also treat children affected by pneumonia. There are many things that are incorporated with our routine.

Do you have anything which is important to tell us that we have not asked you?

No.

Which kind of support have you found very important to carry out your task successfully?

Gashe Zemed is never gets tired to give us every kind of support. He goes down to the village to give support to the community.

He organizes a meeting during which mothers are asked questions to find out their awareness about the kinds of services offered by health facilities. He is watching carefully how we give medical care to children and make an appointment to meet a patient. He perceives with his eyes whether or not we give good quality treatment to our patients. He wants us to know everything.

Area Identification Amhara region

Name of facility: HC head

Date of discussion 16/6/22

Start time: 2:05 PM

End time: 3: 43PM

Can you please describe the demand generation/SBCC activities you are doing on ICCM/ newborn health?

JSI have been implementing activities pertaining to newborn and children under-five. JSI with health center and Woreda health office conducted a baseline survey to collect factual information about child health service before the project started to implement the activities. Actually, we have been giving services for children because it is part of our routine. In addition Generally, we identify newborn that are less than two-months old to find out their well-being.

Health care services are being given for prenatal and postnatal mothers, children under-five classifying into two categories i.e less than 2 months and 2 month -59 months.. We are working all these activities in collaboration with JSI L10K.

The results of the baseline survey have shown that low performance in providing health care services for children. Because of this study result we are working focusing on services provided for children with JSI. We are facing a lot of challenges when we carry out these activities because the community is scattered in different directions. The topography of Woreda is very challenging. It is characterized by many mountains. Owing to these circumstances, it was not so easy for members of the community to bring their young children affected by illness to the health facility to get medical care with a short time. Because of this we go to house to house to give the service.

Though we informed the community about the service, the geographic feature of the area has caused serious delay in our activity. When we do our job house to house we got OTP but not neonatal tetanus/ sepsis. Home delivery has not been abandoned completely in our neighborhood yet but we couldn’t access those delivered at home because of the challenging geographical structure of the area. In our health center we investigate and treat clients doing differential diagnosis. Unlike to other health centers we have not gotten sepsis. This might be because of our methods of investigation and diagnosis. There are health centers that reported local bacterial infection saying Tetanus. They have been doing this to increase their report. I and other health staff of this health center don’t allow such type of report.

During follow up of health posts that we got a great proportion of children under-five who are brought to the health center to get medical care are those affected by diarrhea and pneumonia. And HEWs treated these cases

accordingly and in a better way. Majority of the HEWs has many years work experience except few who are new. We sometimes give comments for mistakes which were done by new HEWs.

In general the report of our health center regarding neonatal sepsis and tetanus is low when compared to others.

You have mentioned that you have conducted a baseline survey before you started to start implementing activities? What was the main focus of the survey?

JSI has conducted its own baseline survey. The survey has indicated our limitation. The survey has tried to identify whether or not prenatal care was given properly.

We wanted to know if the pregnant women started follow up before 16 weeks or not and did the pregnant mother attend prenatal care in a sequence of events as per the arranged time. We also wanted to know whether the women have delivered their child in health facility. But, baseline survey has verified the condition to be different from our report and speech.

Can you please describe the demand generation/SBCC activities you are doing on ICCM/ newborn health?

We go to a church to communicate information to the community when they take up religious service. We discuss newborn and maternal health with religious fathers who have a feeling of worry or concern for same.

We also pass on information to the community whenever they are invited by kebele administration to attend a meeting. We also make use of women's conference to pass on a message pertaining to newborn and maternal health including, prenatal and postnatal care, how to do breastfeed and to deliver their baby in the health facility. In addition, we offer them advice about the benefit of getting their child vaccinated. We made clear what will be the impact on their health if they don't use/get the service.

How often do you organize women's conference?

The conference takes place every month. HEWs communicate a message to pregnant mothers during their conference and whenever they go from one village to the other to observe and direct the execution of an activity.

Women's conference is organized in the health post. But, if the women live a great distance away from the health post, it will take place in an area of their choice.

What other strategies did you use to raise awareness?

We also use mini-media to give information to clients and for the community who are living near to the health center. Besides, we go to the community living far away from the health facilities by ambulance to make the service known by the public through microphone.

Pertaining to ICCM, we offer advice the woman to give proper attention to newborn health and to bring the new born to health facility when they see a a sign of health problem.

The women are informed to give information to HEWs about anything that has happened to the well-being of the newborn. Sometimes, the mothers may not be aware whether their child is in a poor state of general health.

They bring their child to heath facility when the new born refuses to suck breast milk .We inform the woman at what time of interval the neonate urinate and defecate and to observe these conditions carefully.

What kind of message do you transmit to the community about ICCM by using mini-media?

We transmit information about the importance of child vaccination, attending prenatal and postnatal care and institutional delivery and breast-feeding. We also educate the women to feel comfort or ease before and after they delivered their child.

Who did you establish partnership with to create awareness for the community?

We establish special association with mothers who are excellent example that deserve to be imitated by the community at large. We use as an example a woman who has taken children vaccination card after finishing all vaccination properly. We show the child's vaccination card for the community.

We give them the necessary information based on fact. We give advice to a pregnant woman to attend prenatal care, and breast-feed her child eight to twelve times. We give them information about the benefit of feeding the baby for 1000 days properly based on firm or scientific evidence. If a mother does not breast-feed her child as suggested by a health worker, the baby's ears will produce pus by infection. Besides, his ability to learn will be low. But, the child's parents may not have awareness about the reason for the illness.

Where is the venue in which information dissemination takes place?

We give them information in the health center and health post. We also go to every household to pass on a message. We ask them to show us their vaccination card.

We also show them a chart containing illustrations or pictures and ask them to explain for us about the messages they are intending/ planning to convey. Then, they give ideas that result from supposition as opposed to ideas based on facts. If the mother is not literate we tell any family member who can read and write to read and explain the message on the leaflet.

How do you describe the participation/network of Women Development Armies in the implementation of ICCM/CBNC?

Our relationship with WDAs is becoming increasingly tricky/ problematic. The community does not want to move forward.

Besides, senior members of the community who have influence and authority within same show no care or interest to the well-being of the public.

The community now feels anxious about the deteriorating security situations of the country as a whole. They give priority for negative impact of the conflict/war/.They don't give focus for the prevention and treatment of specific disorder/ illness.

A number of women are facing hardship after either their couples or son have joined the fighting. The community is now less interested to hear us discuss coronavirus pandemic or any other health related issue.

The fighting which has been going on between government forces and the rebels for the last two years made the community feel disappointed. Currently, the community is showing us support that does not appear to be sincere. We are able to find WGAs only with difficulty.

What are the other problems that made WGAs less motivated to mobilize the community?

Some Members of WDAs demand financial incentive as entitlement to get their farmer work done in their house or field. Unless we can participate on religious days when farming work is prohibited.

Others say that they are not in good health. Still some of them hide their feeling or reason. We try to persuade them to keep on providing service for the community by giving reasons. We inform them that the government is planning to transfer the duty to the community to manage health service.

We tell them that the government does not have adequate resources to make payment. We inform that the community has to keep their health by themselves. HEWs have started to take part health activity some thirteen years ago. In the past some incentives was given to have understanding and knowledge on the importance of participation. But now there is a structure called one to five and one to thirty. The leader of the group has a responsibility to empower the other group member. But, only limited numbers of members of WDA were convinced to continue to invest their time and energy in such a scheme. They talked to the administrators about their complaints.

How do you describe community's response pertaining to ICCM/newborn service utilization and participation?

The majority of the people are showing interest to take part in the program and utilize the service. For example, the number of children who are vaccinated is increasing. Mothers are bringing their child for vaccination unless they had some social problem.

But, some households bring their child to health facility after an arranged time. They give some of their time to discharge social obligations.

The community is now well aware of a direct consequence of failing not to get health care service when their children are sick. They are able to perceive about a good effect of nourishing their children minerals, vitamins and other substances that promote health. Their general awareness or possession of information about the advantage of a toilet and a kitchen is extensive.

But limited numbers of households are slow to get their child treated in the health facility because of uncertainty or reluctance. They claim that their child's illness has connection with a spiritual entity / demon or a ghost/.

For example, at particular occasion a woman came to our health center to get treatment for her sick child. He was a seven- month old child. But, he was not vaccinated. And the woman said that she delivered her child in her home. She said that she did not have any awareness about institutional delivery.

Then, I gave treatment to the child. Then, he was completely cured. After that she brought the child to the health center on many occasions to get him treated. This is how lack of information affects our well-being.

What are the main challenges you faced / if any/ when you strive to bring behavioral change of the community to make use of ICCM/neonatal health care service?

There is a great deal of disagreement or dispute among many stakeholders such as kebele leaders or managers. We cannot bring about the desired result unless they take part in the ongoing process.

These entities believe that giving medical help to the community is the sole duty or responsibility of health workers. We know for sure that HEWs are required to discharge their own duty.

But, it requires the involvement of every entity with a direct interest in the program. as far as I know we used to give proper health service to the community. But now I am seeing different things. In past they would assign one person to assess the work done by the health workers every week. We are seeing a problem with kebele administrators at all levels.

We are showing certainty Health workers are firmly determined to do their job. But other entities that have a stake show little concern for the project. Health workers have no sole responsibility to do the job.

Previously, we would do our job together with other sector offices such as agriculture. But, currently that is not the case. This year the board or the group chosen to make decision has not gathered us for discussion.

We were told that the chairman of the board was replaced by another person. I hope he will evaluate our last year's quarter performance.

Why are you not able to organize and make them participate in the activity?

It was a period of armed fighting between government forces and the rebels. They were busy making the community return to a previous state of mind.

This year we have devoted much of our time on resource mobilization because of the war and different types of campaign. Therefore, we are required to do better to bring about a desired change.

How do you describe the strength of the support you got from the PHC and the project as well as your efforts to address barriers to deliver ICCM/PSBI during COVID-19?

I don’t think the HEWs have any capacity and skill gap to carry out their task. However JSI has been organizing a number of on job training sessions /coaching for HEWs. Every month we examine HEW’s registration book to evaluate their implementation performance and to make sure that everything is on a right track.

Then we bring HEWs together and we make comments in the form of opinions to remove errors if any. We tell them to bring the register with them. Then they exchange their registration book to one another and evaluate. Accordingly make a discussion on the right and wrong activities. Or we will go to the health post if they are not able to come here / health center.

After that we will give them a case or an imagined subject of investigation. They will be asked to find a solution to an imagined sequence of possible events alone.

Then, they will submit the result of their investigation on the basis of which they are evaluated. This was what we were doing.

Coronavirus pandemic posed a lot of challenges. We believed that the vaccine would not bring any problem to the community. However, our assumption did not come true.

We have tried hard to prevent the community from being infected by the virus because the community was not willing to take COVID vaccine after they saw some people become sick after taking it.

The community was suspicious even for other vaccine thinking that the vaccine might be COVID vaccine. We don’t believe all health professionals except some who worked here for long time. So, the community refuses to take children vaccination. We taught them about the vaccine and told it is not COVID vaccine.

A lot of people reported that the vaccine was having secondary medical effect. Owing to these circumstances, the community was tending to believe that those health workers are not sincere or trustworthy.

They started to believe that we will inoculate their children with lethal drug/ certain to or intending to cause death/. They refused to put any trust on health workers who joined the organization recently..

We managed to change the negative attitude pertaining to the vaccine and health workers. But, there were a number of unfounded reports concerning coronavirus pandemic. The majority of community members believed that COVID-19 was caused by “666”.

They assumed that they can prevent the transmission of the pandemic by performing ceremony of baptism. They believed that COVID-19 was caused by transgression of God’s commandment.

What kind of support did you get from the project to deal with COVID-19 to successfully implement activities pertaining to ICCM newborn health?

Currently, the project is decreasing its support in our catchment though I don't know the reason. Especially now we are giving COVID vaccine so it was good if JSI are with us.

Previously, JSI go to the health post and the villages every week or every two weeks along with the focal person or me to give the necessary support. We went to not only to the health post but also to village. Having the check list we evaluated what the HEWs done for the community. We asked the community based on the check list about the service they got.

Some of the mothers may not get inoculation for their child against measles or any other disease at the appointed time due to their understanding and awareness gap as I mentioned before. At a time when this happens, we tell the mothers to get their child vaccinated, inform about the benefit of child health care service and the availability of ICCM/CBNC in the nearby health post.

How do you describe the strength of your efforts to address barriers to deliver ICCM/PSBI during COVID-19?

We pay due attention to children affected by illness. The HEWs treat using guide booklet unless if the case is sever they refer to HC. As I mentioned before they are doing the diagnosis and treatment accordingly. The on job coaching/ mentor ship helped them to develop their capacity, skill and confidence.

For a sick child who has vaccine appointment, we carry out detailed examination to find out the cause of the illness before vaccinating. And the child must be completely cured in order to get vaccinated.

Let me cite an example of a particular instance or event in which a child has died in Dembecha two years ago. The community attributed the child's death to the vaccine. Therefore, we consider every potential reaction of the community carefully whenever we give vaccine to the child.

You told me how the support system is useful. Please tell me the drawback or importance of the support for the HEWs comparing with the traditional off-site training and woreda level review meetings?

We have found the support given by the health center and JSI extremely very important. When Gashe Zemed or health person from health center go to the health post to provide support, they also do other health related activities. Besides the support given for HEWs, they go to the community and mobilize them to maximize their health seeking behavior and at the same time evaluate what HEWs have been providing to them.

On a journey to the community regarding ICCM/neonatal health care service, they carry out a lot of other activities. Besides ICCM and neonatal support they have done pleasing job relating to OTP. The mothers may not bring their child to health facility not merely because he / she is very thin. When they conducted house-to- house visit, the likelihood of finding children having poor or inadequate diet was high. The chance of getting children before developing OTP (malnourished children who are stunted) was high..

How did the support that the project and you have given for HEWs help to improve their capacity and skill?

It is having a great deal of benefit. When health workers from here are assigned to offer support, they may not be able to address every problem unless they have been trained and have adequate knowledge about ICCM. Unless they are shown a gap in their performance, they will not be able to give high quality service to the community. Currently, Gashe Zemed and others take a look at everything carefully in order to find out something unintentionally done wrong.

In addition, any kind of support given to us by an experienced or knowledgeable person will reactivate our memory with a piece of information and they have the capacity to observe each steps of service provision. We have observed from Gash Zemed how to check the performance of HEWs. JSI wants us to carry out our duty according to our plan of action and objective toward which our effort is directed. Also we have found the support offered to the health center very important. HEWs learned and improved their skill and knowledge because of the support they got.

A mother who is pregnant and her delivery is estimated to be this month , HEWs plan for her postnatal follow up including assessing the health condition of the baby. Gash zemed and health professional from HC checked whether HEWs do this planning properly and then followed the mother and the baby after birth. The mentor ship is conducted by skillful person based on the target.

Did HEWs express a feeling of approval for the support given by JSI?

Yes they did. They said the support is important because they were able to get feedback or response regularly to remove errors and improve their working effectiveness. HEWs have claimed that the support given to them is instrumental in achieving a result or accomplishing a purpose. His support was also important to us.

Do you think the support should have been given differently to do a job better than the one you have accomplished so far?

The support given to direct the execution of ICCM/CBNC was very good. I don’t have to say more which was being done differently. They were using each opportunity. When we and HEWs were busy because of campaign, Gash Zemed didn’t want to miss or cancel his schedule so went with us and did what he planned on child care and also supported our campaign activity. They were not frightened to go to the community to give assistance. They have worked hard without mental and physical exhaustion. But, there support is decreased so we must make effective use of active assistance and encouragement given by JSI now onwards. I could say that there is no organization that worked like JSI. Thank you for their hard work.

How ECHIS implementation helps you with ICCM service delivery?

First of all, the electronic system is functioning by itself to give a report about what has happened. It has its own format. It let us know everything via the link or connection.

They can store the information into a mobile phone and then they can have data from a computer printed out on a paper. If they fail to store the data onto a computer disk, they cannot get from anything to write and submit a report. They need to have data stored on a computer disk and printed-out data to write and submit a report.

The computer is adjusted with start and end date for each service. If a woman did not attend ANC,PNC, it will produce a red light. Likewise, if a child was not assessed/examined during postnatal and not vaccinated, it will produce a red light. And the system does not allow doing on it if a child who missed vaccination services comes after a year because it does not do similar things for children after one year.

They cannot take the errors out of the system just like they do on a hard copy. The system does not allow them to cheat or mislead somebody. Everything is online or connected via computer. Therefore, the HEW writes and submits meaningless or very accurate report. It helps to prevent fabrication of false data.

Please describe its advantage to improve the quality of ICCM?

I don't think I have no the required skill to give any comment on this issue. As I mentioned before and as far as I know it helps to detect those who has appointment for maternal and child health service.

If the ICCM/CBNC guide is available on the system, it helps to detect the case and its treatment easily. They cannot order the use of the wrong medication. If the HEWs fills the symptom and sign they hear and see on the child (Temprature, respiration and others), the computer shows HEWs exact diagnose and treatment of the baby accordingly.

Therefore, the system will provide the health worker with the means to make identification of illness correctly. But treating a child using ESHIS has not been started yet.

Do you think it is helpful to retrieve or manipulate documents easily?

Yes, it is very helpful unless there is frequent interruption of power. It is a systematic and methodical way of doing things. For example, I keep all information in my mobile phone.

It saves all the information about the number of members in the family in each locality. So when they insert the head of the family it displays all other family member in that household. As I told you before the system is connected so I give feedback about the number of registered household and family member looking on the document they sent. It makes finding of documents, service provision and our communication easy.

What should be done on ECHIS to make ICCM service delivery better?

The connection needs to be updated(upgrading the GB). Because of connection problem there is a challenge to register one family member and to sink the data. The communication link between the mobile phone must be updated so that information can be mutually accessed or exchanged.

Sometimes the sim card inserted into a cellphone is closed due to a failure to settle a debt or other obligation.

I suggest the provision to the health posts of a device operating using energy from the sun as a good idea. Lack of electricity has caused a serious delay in action or progress. We are facing a problem when electric power goes off for about two days and more.

The focal person has taken in part in a training program and learned a skill. But barring of access to information by a mobile phone and interruption of electric power have remained main problems.

What are the challenges that HEWs faced to diagnose or identify neonatal infection ?

Sometimes we discuss on identifying neonatal infections. Some health workers that didn’t take ICCM/CBNC training may fail to diagnose an illness through an interview and or using the guide booklet. So, we are expected to communicate and give on job training how to diagnose, identify and treat. (whether the problem is caused by neonatal infection, measles, or polio etc). When the person trained in ICCM is absent from the health center due to different reasons we replaced by others who didn’t take the training. On such cases we have observed that even the staffs in the health center have capacity / skill gap. Except those who were trained we have seen skill gap. They don’t go through each page of the guide booklet to identify and classify the disease.

What problems did you observe in identifying and treating neonatal infection when you conduct mentoring/onsite coaching for HEWs?

There is no gap neither identifying nor treating neonatal infection and under five children health problems. The problem is not because of lack of skill. Usually, we and HEWs identify local bacterial infection in a child and treated as local bacterial infection. We don’t want to classify it as neonatal infection/sepsis for the purpose of report. I don’t mind somebody, who evaluated this health center and its catchment health posts, criticize or blame for not getting neonatal infection/sepsis want.

But I knew in some places a small number of health workers act as if they have identified an illness or disorder (neonatal infection/sepsis) just for the purpose of submitting a report.

Did you face any problem regarding supply of inputs?

At an earlier period, we were having a problem regarding supply of inputs. For example, it was somewhat difficult to get supply of plum peanut. After we identified malnourished cases, we did not get positive response from the woreda health office when we made a request to get plum peanut. Even the woreda health office was facing a problem to get it from the zonal health office while the supply is available in Zonal health office.

As a result, there was a lot of misunderstanding between us and many children were suffered. But, the problem was resolved through discussion. Now they give us plum peanut whenever we make a request.

There was also scarcity of Ampicillin which is used for under five treatments and Gentamicin. JSI stopped to distribute the drugs for some time while we were waiting the distribution from it as before because of this we didn't requisite and faced the problem.

How did you solve the temporary problem you have been facing?

We tried to borrow the substances from other health center. That is what we do whenever we face scarcity of drugs or any other material. But, after a particular period of time we have requested and purchased the drugs like other medicine and distributed for health posts and also health centers(Yezeleka HC).

Gashe Zemed was displeased when he saw the condition of having no stock left. He told us a lot of drugs have already arrived on the port. We told him that there is a plan of action to purchase and distribute the drugs to the health centers.

Did you observe low community demand/ lack of interest to use ICCM/CBNC service?

Many people lack the knowledge to make use of our service properly. They like to use traditional healing practices. They use medicinal plant to treat their children affected by illness. They do it as a custom whether or not they bring their child to health facility to get treatment especially up to one year old age. The issue of traditional healers has been becoming well-known since three or four years on wards.

I have tried hard to convince them to give up the practice. I tell the community that the methods that traditional healers are using are not compatible with the methods and principles of science. Because of this effort they brought to us and treated accordingly.

We give information to the community to free them from ignorance or superstition about child neonatal and local bacterial infection. I am using every opportunity to give clarifying information to the community.

How did the state of emergency and the war between opposing forces affect your activities?

There was no immediate threat happened because of the state of emergency in our area. But, one day some young men tried to create a problem on Gashe Zemed. But it was not done on purpose. During that time the community was informed to be alert for any strangers. They did not have any information about Gashe Zemed. They called to me and cleared the ambiguity.

Additionally, we faced some problems when we conducted the baseline survey. The problem was related with the type of question raised on the questionnaire (asks about religion).

We did not face any other challenge related to state of emergency except those related with meeting community's requirement and organizing women's conference. But, the conflict that claimed many lives and caused destruction of property was a subject of concern that was and is still causing worry for the community at large.

The community was raising problems they faced because of the conflict and war. They were having little concern for their wellbeing. They were having a feeling of apprehension when they saw displaced person. Many of them have lost their children and loved ones to the war. They mention a lot of disheartening issues for us.

How do you think the occurrence of COVID-19 pandemic and its response measures affect the uptake and delivery of ICCM/CBNC services?

Members of the community were feeing worry of COVID-19. The communities dislikes COVID vaccine therefore the they didn't ask to get services when we go house to house to provide any service. In the past they were alert and follow actively not to miss any services.

Generally COVID did not affect our routine. We used to go to the community to take care of newborn, children under-five and pregnant mothers without any difficulty. But, a lot of people were eager to take treatment against trachoma but after COVID many people don't take considering it as COVID vaccine.

COVID-19 did not cause any problem regarding delivery of ICCM services. Neither did it affect our children vaccination program and provision of neonatal care.

How did COVID-19 affect income of the community to pay for transportation to bring their children to health facility?

A lot of people were forced to move on foot to the health facility and back home because of lack of transportation. We told them to come to the health center or health post by wearing a face mask and using disinfectant.

Accordingly, many of them who have no protective mask covering the nose and the mouth and sanitizer because of this they were afraid to come here. Also the persons who have not been vaccinated against COVID0-19 thought that we would not let them in the health facility. Similarly, we are telling them about the consequences of not taking the vaccine. At particular occasion we discussed not to let in people who have not been vaccinated. But, after short time we have abandoned the idea.

We have decided to take the necessary precaution against the virus by wearing a face mask and using sanitizer. Prohibiting people from entering into the premises was not done practically though the community communicated like that.

Explain to us any changes you observed as the result of the implementation of the COVID-19 adaptive ICCM implementation strategies?

We used to give treatment to anyone affected by illness despite coronavirus pandemic. But, many people showed signs of fear to come to health facility by the presence or anticipation of danger/ infection. And facility delivery was decreased.

They thought that practicing physical or social distancing was an indication of disapproval. They thought that any act or sound of coughing was a sign of infection by COVID-19. The message being communicated made the people feel anxious. Anyway, we used to give the necessary service to everybody despite a situation causing fear or alarm.

What changes did you observe as the result of the implementation of the COVID-19 adaptive ICCM implementation strategies?

We start to control everything at the gate. We offer them advice to practice physical distancing and wash their hands with water and soap or detergent. We arranged water and soap at the gate.

After that, everyone seeking service would be taken to separate room. Persons who are showing an act of coughing would be isolated from others. The watchman used to offer welcoming and generous treatment to visitors.

What kind of support did you give to HEWs?

We offered them advice to communicate vital information to the community to take the necessary precaution against COVID-19 by using protective face covering and sanitizer. HEWs have offered advice to the community to hold themselves back from going to main urban centers to minimize the risk of being infected by the virus.

House to house service provision was strengthened to access those who didn't come due to fear of COVID.

What are the basic results/benefits achieved because of the support you and the project gave to HEWs ?

The support made access to newborn easier because of the house to house service provision. And HEWs used to refer the newborn to the health center whenever the case is beyond their ability to treat.

In the past because of zero report on neonatal infection we used to believe neither a newborn nor the mother was having access to care and treatment. We were inclined to believe that HEWs used to submit false report without offering the necessary service to postnatal mothers and their newborn babies. But, our suspicion has turned out to be incorrect. We have discovered that every child and mothers have been getting the service.

But, not all members of households visited are given treatment. Some of them receive health care advice. Members of the community are advised to visit health facilities if they face any problem related to their well-being.

During our visit to the community we make sure that mothers and their newborn were given medical help. The community provides us with information.

Because of the support HEWs developed their capacity and skill. Assessment and follow up of newborn have been strengthened. As a result they manage to rescue a lot of child from sever morbidity and mortality. Generally before

the support we heard about death of a new born or mother who gives birth after 1- 2 weeks but now such type of cases is more or less addressed properly.

Please explain to us the feasibility of this support/ IR for national scale-up?

This is visible and could be witnessed by all health professionals and HEWs. The service and care given to newborn and their mothers is beneficial enough to justify the time taken, resources devoted and efforts made. And we can keep on implementing ECHIS because it helps to save time, and hasten to do activities in an organized way. As we have seen it is convenient and possible to continue treating child using guide booklet. The main thing is giving training to HEWs and mentoring them until they have been capacitated and skilful.

But, a small number of employees are not able or willing to keep their data properly using ECHIS. And some dislikes following HEWs regularly. Regardless, I think we are on a right track.

Assessment made by JSI every week/two week was very useful. Any staff member can provide child health care service if he / she has the necessary skill/ capacity. We should not remain dependent on JSI for an extended period.JSI gave the support to capacitate HEWs and to make health professionals qualified in conducting mentoring and onsite coaching. And JSI achieved its plan. Here after it is possible to continue every activity that we have learned from them.

What kind of challenge do you foresee to continue to give support to implement the project?

I don’t think something bad will happen provided we have the necessary devotion to duty. But, the problem related to communication link between telephones should be resolved.

Also, WDAs must provide HEWs with the latest information concerning people affected by illness, and about number of newborn in the respective localities. WDAs and stakeholders should be motivated to overcome their responsibilities and accountability.

Everybody / entity that has direct interest in the program must contribute something in the form of time or energy to make it successful. But, HEWs cannot do everything alone. They will face psychological tiredness and reduced efficiency resulting from overwork or elongated exposure to stress.

How the activities are embedded in your routines/work streams?

We exchange a report on a daily basis. If we think that HEWs have failed to discharge their duty efficiently, the supervisor or HEWs coordinator will go to a place/ site to oversee and guide the work.

He watches over the entire activities about newborn, postnatal mother and the community. After that we receive a report about his findings or any other issue. We have different types of reporting mechanism such as maternal and child health report, hygiene and etc.

We receive daily and weekly report on ECHIS and other issues as deemed necessary. We asked HEWs how many household and family members they registered in ECHIS on daily bases. And then we submit weekly report to the woreda health office. The supervisor or any other person discus with HEWs for lack of consistency in service provision based on the report / if any/.

I must ask HEWs to make sure whether or not all pregnant women have attended prenatal and postnatal care and whether every child is vaccinated against polio, measles and any other infection. Similarly we check whether newborn and under five children are getting health care service accordingly but I don't focus on the number of pneumonia, diarrhea, sepsis and other illness. We don't need the number of morbidity to increase but to provide appropriate service for the community to make them healthy

What implementation strategies are incorporated with the PHC and woreda annual work plan?

We prepare a plan of action together with the zonal health office to implement ICCM and CBNC. The woreda health office can make changes or alteration to the plan accordingly. We have already submitted 2015 E. C. budget year plan of action for the woreda health office. On the plan action ICCM, neonatal sepsis, diarrhea, malaria and others are calculated based on co relational factors.

The plan of action will be distributed to every health post. Then, HEWs will prepare separate action plan for the villages and the kebeles.

How is the participation of Village health leaders in your community?

We have recruited members and reported for woreda health office and now we are on the way to give training to them.

Area Identification Amhara region

Name of facility: Lejet HP

Date of discussion: 13/6/22

Start time: 10:05 AM

End time: 11:34 AM

Can you please describe the demand generation/SBCC activities you are doing on ICCM/ newborn health?

We have participated in a training program concerning provision of care to newly born that is less than two months old. The training dealt with a number of issues concerning new born health. After the training we managed to inform/show to the community clearly and convincingly our ability to deal with all forms of new born health problems.

Next the leadership and VHLs (were formed by JSI) have been made aware of about the process of managing neonatal health problems in the health post. We managed to make available the necessary inputs to meet a standard or requirements to keep newly born healthy. At an earlier period the community was not having trust in our ability to apply medical care to cure neonatal diseases. But this state of mind has changed for the better and now days the community is coming to our health post seeking care to their newly born child. Generally, we provide medical care to all children under-five including neonate.

What other strategies did you use to raise awareness of the community about ICCM/PSBI? What activities did you do to aware the community?

We have applied different strategies to improve community's awareness about ICCM and neonatal health care. We communicate information to the community in the place where large numbers of mothers gather together such as when they go to church and during child vaccination. We informed the community about all the services that we provide in the health post when they gather together.

Firstly, in health center and other health facilities mothers may be queuing for a long time to get medical services for their child. In addition, they may be asked to pay for the services rendered by the health center and private sector but not in our health post.

Because of this reason, we have educated the community to get the necessary medical care for their kids, which is similar to the service given by the heath center, free of charge and without any difficulty in the health post which is near to their living area. Members of the community have been told that in case they are not able to bring their children to the health post for different reasons, HEWs are going from house to house to give them treatment.

Generally, we have made effective use of available opportunities to increase the level of awareness of the community and community leaders regarding child and neonatal health and vaccination.

How do you describe the strength of the support you got from the PHC and the project as well as your efforts to address barriers to deliver iCCM/PSBI during COVID-?

The project has given us active help and encouragement. Specially the training which was given to the community leaders by GSI strengthen the link among HEWs, development army and health leaders. Besides, GSI has helped us build the level of knowledge of our stakeholders. They have been made aware of child and neonatal health services. It provided us with material support. It has exerted the utmost effort to fill our skill and knowledge gap. Generally the support that we got from JSI is admirable.

What are the major challenges you faced about awareness creation on ICCM/new born health?

At first we have been experiencing lots of challenges. The community did not trust that we are capable of providing medical care to sick neonates . They have underestimated or misjudged our capacity to treat child illness successfully.

They thought that the quality of services offered by the health post is inferior to those of the health center. Owing to these facts and circumstances, lots of parents used to get their children treated in the health center. But, this way of thinking has already changed significantly through time because of the intervention and they saw when sick child get better after getting treatment at the health post. The community has now put the wellbeing of their children in the trust of HEWs.

What other means did you use to build trust of the community on the services that you give to child and neonate?

The health center has assigned one health worker to give the necessary support to our health post. Therefore, HEWs together with the health worker pass on vital information to the community. We make effective use of public gatherings such as edir or religious service to give knowledge to the community.

We also conduct house to- house visit to communicate information to the community. There was a meeting which was conducted every 2 weeks by the command post. Key stakeholders, woreda sector offices / agriculture office, health office and education office/ are members of the command post. Members of the command post are powerful and have the ability to pass on decision. They evaluate each sector's performance. We discussed with them and made child health one of the agenda on their minutes and introduced to all participants the availability of child/neonate health services in the health post. They pass on a message for the community on behalf of us about the availability of the service when we are together with them or not. The size of the area of the kebele is very big. Because of this we couldn't reach the whole community alone. Therefore, we asked them to help us

whenever they can. Accordingly, they communicate child health information to the community using each opportunity. The whole idea is to encourage the community to play a role to child health.

Generally, it is possible to say that important personalities are in attendance of the meeting. A formal list of child health issues to be discussed was put in specific order.

In collaboration with JSI we have organized a conference / meeting in which the community discussed the health of child. People from JSI discussed child health with a great deal of knowledge to the community. They have expressed their thoughts or feelings about the services being offered to the community.

What is your opinion / thought about the participation of the community?

Currently the community is ready and willing in accepting child health services. Unlike in the past, the community is now highly responsive and is quick to take in new information. Formerly, the community was very much suspicious of our capacity to give proper treatment / medical care. Because they understood that HEWs only provide package services but not treatment.

Mothers now speak remarkably and frankly about our capacity or effectiveness to give appropriate medical care to the community. The community is now having faith or confidence in our capacity when they saw cured child after getting the treatment at the health post. Mothers discussed each other about the children who get cured. Mothers became witness for our services.

Likewise, the number of people coming to our health post seeking child care is on the rise. Now they only go to the health center when we went for vaccination closing the health post. Generally speaking the number of children who get child health care is increasing during the last one year.

Who else has participated in the implementation of SBCC activities?

The woreda health office was able to show a high level of participation to that effect. Also the involvement of health workers in building the level of knowledge of the community was remarkable.

The participation of village health leaders and development groups was worthy of notice. The involvement of the village health leaders was indispensable to the continuing effectiveness of the program. There are 25 village health leaders in total. They are extremely important or necessary.

I saw VHL pictures posted on the gate of the health post.

What were their roles and responsibilities?

Initially there were development armies. They were all females. But now these VHLs are serving HEWs and development army as a bridge. They have attended formal schooling above six grades. So able to read and write. The community has a feeling of respect or attitude of admiration to them. They are capable to implement the

short time training that we gave them into practice. They are in charge of managing 80-100 households. They organize their community in order to be ready for action. Currently they are the one who perform noticeable job. It is now almost two years since VHLs were assigned.

How do you describe the strength of the support you got from the PHC and the project as well as your efforts to address barriers to deliver ICCM/PSBI during COVID-19?

We have attended training program and learned the skills necessary to do a job and have friendly relationship with the community. After the outbreak of COVID pandemic JSI gave us direction to strengthen house to house service delivery and created enough awareness to us how to create strong link with the community.

What makes you to say JSI created enough awareness? What did JSI do?

They developed our skills how to create strong link with the community. JSI aware and gave training to religious leaders, members of the kebele leadership, school principals and other community members who has acceptance in the community. By doing this they developed integrated service provision. Additionally, JSI has organized seven-day training to village health leaders concerning general Maternal and Child Health service.

How do you describe the support you got from PHC?

The health center arranges a meeting every two weeks or every month in order to evaluate our performance and address our capacity gaps on child case management. They provide us with the necessary inputs. We take a chart with us to the meeting. It is displaying detailed information about our activities on ICCM and neonatal health care. We exchange our guide booklet to each other and evaluate how it is done and comment accordingly. Health workers mention and discuss scenario or imagined sequences of possible events and their appropriate treatment during the meeting. Not to face with shortage of supplies they supported us with the necessary supplies.

How do you describe your efforts to address barriers to deliver ICCM/PSBI during COVID-19?

Each of us was striving to make the activity continue despite the challenges posed by the onset of coronavirus pandemic. We used to conduct a visit to each household to maintain the service despite discouraging crisis caused by COVID-19 across the whole world.

We were afraid mothers may fail to bring their children to health facility to get health services due to fear of corona virus pandemic. COVID-19 caused delays the community in seeking health care services. That was why we started to conduct a visit to every household to give advice to the community to bring their kids to the health post to get child care health services.

Providing services house to house was just the intention of the project to significantly reduce child morbidity and mortality. JSI wants us to carry out our task successfully regardless of coronavirus pandemic. The project was planning to fill our capacity gap.

They gave us support to share practical experiences among different kebeles. JSI has done commendable job reaching mothers at household level to enhance awareness of the community. It has built our capacity to efficiently carry out our task.

What were the barriers you faced to deliver ICCM/neonatal health care service during COVID pandemic and what else did you say about the support you got to address these barriers?

After the beginning of COVID-19, members of the community stopped to bring their children to health post because they were scared of being infected by the pandemic. Pregnant women were not willing to come to health facility to get health care service. However, we offered them advice to get treatment using preventive measures such as keeping distance, a facemask and sanitizer to avoid infection by corona virus pandemic.

We were given a face mask and sanitizer to prevent infection by corona virus pandemic for us and for the community while providing services. Likewise, we managed to conduct extensive house-to- house visit to enhance the level of awareness of the community. In addition we strengthened and improved the service we deliver at household level to make effective use of services that we provide

What could have been done differently to address barriers of ICCM/newborn health care service delivery during COVID pandemic?

We managed to efficiently carry out our task despite the onset of coronavirus pandemic. The pandemic did not cause a delay in action or progress. I can honestly say that we have done satisfying job by strengthening house to house visit after the onset of coronavirus pandemic. Reports were submitted to the health center on time.

Committee has been set up at kebele level. We used to receive a report from the committee on a daily basis. There are eleven goots. Members of the committee are reliable owing to qualities of conscientiousness and trustworthiness in carrying out a task or role. Members of the committee were selected by the community.

The committee is required to provide us with the latest information regarding the community. The committee will give us information about newcomer to the place in order to reduce spread of coronavirus pandemic to a minimum.

How could the support system be improved?

I don't think anything could have been done better We have done in a better way during the pandemic of COVID in coordination with the PHC and the Woreda health post and we report the activity that we did every day. Besides as I told you before the committee who were selected by community provide us daily report at each village.

Overall, the support during the CVD was excellent. They encouraged us to go home and work without a break

Generally, the support given to us was good enough to meet a requirement or to be considered acceptable. We did not have any leisure time during which we were able to engage in enjoyable activities. We tried hard to persuade to the community to use face mask and sanitizer and to practice physical distancing to avoid infection by COVID-19.

Did do you get the support system mentorship and on-site coaching methods, supervision, technical support, PHCU level PRCMM, etc helpful to enhance your skills over the traditional off-site training and woreda level review meetings?

We are thankful of the support given to us to improve children's medical service. They were frequently coming to our health post to offer us the necessary support. They were watching carefully the act of providing service to our clients. They have observed duration of our stay in the health post.

They have noticed whether the right type of drugs or other medications are prescribed by the health worker. They used to come here to make a fair assessment of the quality of services being offered to the community. They went on a journey to the villages to examine and judge whether services of the highest quality are provided to the community.

What additional thoughts did you have on the mentorship, on-site coaching methods and other supports? Please clarify how it helped you to improve your skill?

The support given to us was important to deal with problems successfully. In my view JSI should keep on providing the necessary support. We know that error is human. We can make unintentional mistake. JSI did timely supply provision and technical support and gave a chance to ask any unclear things and gave us timely clarification while doing on job mentoring.

The health professionals are very supportive of us coming from the pediatric department of PHC and JSI. They look at our treatment regimen from the chart. They check how many times a day we provide services and whether the health post would be closed or not. They check the record of those who we have treated and see if the treatment is correct or not.When we have lack of skill, Health workers from PHC and the project, who is knowledgeable, can teach us the ability to do the job well. We take a diagram or table displaying detailed factual information. They mention an imagined set of circumstances / worst case-scenario/ for which a method of dealing with same will be sought. In fact, they are showing us a way of resolving the difficulty immediately and without delay.

Medical profession demands perfection. An arbitrary decision or anything based on random choice will claim human life. The child's body temperature must be measured and his / her weight determined before we order use of medication.

It is strictly prohibited to give treatment to anybody without referring to guide bookle. We must take the age of the child, temperature, breaths per minute and others into account to order a course of treatment. Accordingly, our supporters make sure that the drug fits the age of the child. They gave us a comment when we wouldn't do correctly.

In past, there was lack of clarity to order (the use of) gentamicin. We used to order or prescribe 40 MG and 80 MG gentamicin. In fact, we were scared to order the required dose of gentamicin for children. Because of this we used to refer seriously sick patients to the health center to get the service for those ill and critically ill children..

But Gash Zemed showed how to calculate and prepare the required amount of Gentamycin. We have already acquired the skill how to give the required amount to a children who are seriously ill. Because of this reason, we are no longer referring patients to the health center.

How do you compare the trainings which were given in the past and the mentor ship/onsite coaching which have been given for the last one year?

Currently, we are compelled to make effective use of the knowledge gained through trainings. We are accountable for successfully carrying out of duty by applying the skills gained from the training. We prepare plan of action at the end of the training. But in the past after taking the training no accountability how we were doing.

They make a visit to our health post in order to find out how the work is progressing. They want to make sure that activities are implemented in accordance with the plan of action.

Currently, we are in a better position as far as our capacity to carry out our task is concerned. We are already aware of many things. This methods is accepted by HEWs. If we are not accountable, we tend to forget the skills gained from the training. Additionally, the community will not trust us. But these, mentorship, on job coaching and other methods of supervision, are very important and acceptable because it helps us to improve our skill and not to forget our knowledge and

In your opinion what supports need to be improved to make service delivery more pleasing or acceptable by the community?

I think we must be given the support at an appropriate time to achieve the desired result. Sometimes we are given support without interruption or break. But at another time we are given support in an interrupted sequence. Whether the follow up is conducted quarterly or monthly, shall better to make timely to improve our skill.

What is your opinion about the support given by the health center?

They make a visit to our health post three times in a week. They give us the necessary support. They come with checklist or list of items to be delivered to the health post. If we didn't ask them, they would give anything necessary based on the checklist they have.

What type of support do they give to you/ health post?

They give us all types of support. They give us on job training to fill our capacity/skill gap on each service such as how we gave treatment, how we made report. Besides we invite them to work on awareness creation on the community.

You have a systematically arranged means of collecting community information. How does the implementation of this system help you for ICCM service delivery to the community? How important is the database to efficiently carry out your task?

The database is very important because it can be automatically retrieved or manipulated. We have not finished entering information about male partner into the database yet. Anyway we have found the database very useful. Any Information recorded in writing / on a paper/ can be spoiled easily. I recommend that this system of keeping our data must be strengthened.

Does it have any other importance?

We have already started to use a computer to keep official record of mothers who come to the health post to attend antenatal care. But we didn’t finish registering electronically and not possible to register all services that we provide.

When we register a mother who gives birth, the computer helps us showing the newly born baby who needs vaccination. We are using similar method to keep records of newly born who got vaccinated. We might lose information recorded on paper but not in computer.

This way we can easily identify those who have appointment for vaccination, antenatal follow up ,postnatal follow up and defaulters / if any/. We have what is referred to as action card. It is producing light / brightness. When the child is not vaccinated, the card will produce red light and green when completed either follow up or vaccination. This is how we identify newly born who are and who are not vaccinated. The registration is done on each catchment accordingly it reminds us to visit those who have an appointment listing their name and catchment. It show red light when we don’t give the service.

Generally ECHIS makes our work easier and improve the quality of work by showing those who has an appointment for health care service.

What does it indicate / show about ICCM?

We have not done anything on ICCM treatment. Therefore, I have no any idea on this issue. Only we started registering household head and their family. Entering ICCM service into the electronic registration is started but not completed. So I couldn’t say detail about ICCM.

What is your opinion about strengthening ECHIS?

We are submitting report writing on paper. I recommend to change paper based reporting system into electronic. This will make our work easy..

In your view what do you think should be done to strengthen ECHIS and ICCM service delivery?

We need to be given training to build our capacity. We haven't been given any training about implementation of ICCM in ECHIS.

Describe the main issues faced by the health system to identify and treat neonatal infections in the community. What are the critical factors affecting the delivery of ICCM to clients?

We are facing a lot of challenges to give medical aid to children. Firstly, the number of people seeking medical aid is on the rise. We don't have sufficient space and resources to give the necessary services to all people.

It is difficult to learn the causes of illness of the children. But we have no capacity gap. We are four HEWs working for the health post. But we have heavy workload.

There is no problem regarding supply of inputs. But at on one occasion there was shortage of gentamicin. The problem has already been solved. Every month the health center provides us with the necessary input. Therefore, there is no shortage of inputs that are needed or required.

It is not possible to say that the health center gives the necessary attention regarding ICCM and CBNC service delivery to clients. The area of the kebele is wide. Therefore, it may not be easy for the health workers in the health center to travel to every place / household / to engage in ICCM and CBNC service delivery. There is a gap in service delivery. Everyone one of us has a responsibility. we carry out our task jointly. However, we have not exerted the utmost effort to deliver the necessary service for children.

How do you assess the interest of the community to make effective use of the service provided by the health post?

The community has very high interest to use the service. Our health center is situated near the town. Accordingly, parents are quick to bring their kids to the health post to get medical aid. They are also taking their kids to the health center whenever they get sick. Their mindset has changed remarkably.

For example sometimes a state of emergency is declared in the region. There is also a time at which curfew takes effect to restrict people's movement. Do they hamper your activities?

We keep on doing our job despite a state of emergency or curfew. It does not disturb delivery of medical help to a baby. we also give delivery service despite state of emergency and curfew. We are allowed or entitled to provide medical help to patients regardless of the state of emergency.

Has the conflict / war any effect on service delivery?

There was no armed fighting in this neighborhood. So, there was business as usual in this area as far as provision of medical help to patients concerned.

How do you think the COVID-19 pandemic and/or COVID-19 response measures affect the uptake and/or delivery of iCCM services?

The number of people seeking ICCM and other types of health services was decreasing subsequent to the onset of coronavirus pandemic. They were scared of contracting the virus. However, the problem did not last long. After sometime, people began to come to health facility by wearing a face mask and using sanitizer.

We used all available opportunities such as religious service, edir and funeral processions to educate the community not to stop going health facility to get medical help. In fact, we used to conduct house-to- house visit to educate the community about COVID-19 and provide medical aid to pregnant and lactating mothers and their kids.

When you travel to the community, were they willing to use the service?

The community is not scared of health workers. The community becomes less anxious when it meets health workers.

As we know people's movement was restricted by curfew or the state of emergency. Do you think people have stopped coming to the health point due to lack of income?

People were required to pay a large sum of money for transportation due of lack of means of travelling. A Bajaj / small vehicle / was not allowed to carry more than one person. an individual was obliged to pay birr 40.00 for a single trip / to come to the health post from the nearby village.

Daily laborers were particularly hard hit by COVID-19. People who have no insurance against ill health were facing a lots of challenges to cover the costs of their treatment.

They were not coming to health facility due lack of money. People / mothers / lacking money or material possessions were coming here on foot. Anyway we were not involved in busy activity because of coronavirus pandemic. But gradually the situation began to improve?

How was it improved?

The community was offered advice to get medical aid by using a facemask and sanitizer. They were told to wash their hands with water and soap to avoid infected by coronavirus pandemic. They were also advised to practice physical and social distancing. Majority of the people have health insurance. Therefore, they have no problem to that effect. Members of the community were told that the health facility is always open to all.

Facemask and sanitizer were sold to clients at a point not far away from the gate of the health center. They were not expensive. Buyers were able to meet the cost of a face mask without any difficulty. They bought a single face mask for ten birr.

Explain to us any changes you observed as the result of the implementation of the COVID-19 adaptive ICCM implementation strategies?

The community was having access to medical help despite coronavirus pandemic. We used to inspect condition of the patients including children. we gave treatment to children who were sick of severe pneumonia. Currently good number of children under-five are given treatment in our health post.

What is the reason for the improvement in service delivery?

It is because the community was given education to fully appreciate the importance of the application of medical care to their kids. Children under-five can get the necessary medical help in our health post.

Therefore, parents are not required to go on a journey to health center to get their children treated. This way they will not pay a large sum of money go to the health center and back home. They get every service for free. They will not spend their precious time while waiting to get the service. Additionally, when we go to the community to give treatment, we take the necessary inputs with us. We may come across some people who got sick of pneumonia. So, we take the necessary medicines to treat or prevent illness.

Which of the strategies applied by the project was effective? Which of these strategies has brought significant difference for improving the general wellbeing of the community?

In past our primary duty was to give treatment to children. But currently, we go to each family unit and give medical help to the community. In this way we are producing satisfactory or useful results. This is an important strategy introduced by the project.

We should not expect patients to come to our health post. We take the necessary inputs including the chart with us when we go to see patients. We are operating by combining our efforts and skills.

What does the cooperation between people or groups working together look like?

It is found in good condition. Health leaders accomplish their task successfully. Thanks to JSI people are coming and working together to achieve a common goal. In past only development groups were involved. health leaders have attended formal schooling / education.

Because of this reason they can carry out an action successfully especially one requiring care or skill. They were given training by JSI to enhance their capacity. They have all the information or awareness about child health.

But they don't give treatment / medical aid to patients. They have adequate information in mind because they can read books. They provide us with the necessary information about the general wellbeing of the children in their respective locality or neighborhood. They make things easy or easier for us to do. I think they need to be strengthened.

How do you evaluate the commitment or dedication and level of knowledge of HEWs to give service to the community?

Their dedication to duty is not adequate. Most of the staff is complaining because of small amount of remuneration for the services provided. With a small income workers are not able to meet the cost of living. They have lost hope. It is not possible to say that they take up much time and energy to carry out their duty. Everybody has ill feelings caused by small salary.

But We have no capacity gap. Gashe Zemed is coming here from ICCM. Other people are also coming. He makes things easy for us to share experience with other people. There is nothing they cannot do to build our capacity. The chart displaying detailed information has been updated.

As you have mentioned earlier there was a carefully designed plan of action to implement ICCM despite the challenge posed by coronavirus pandemic. In your opinion which of these strategies has failed to bring about basic change?

All strategies were having good effect and proved beneficial.

What are the high-level benefits that are attributable to this support/IR?

Treating child illness successfully is one of the high-level benefits. Secondly, the number of pregnant mothers who come to the health post to get treatment has increased significantly. Mothers are noticing there is no shortage of inputs. Everybody must have the opportunity or right to use the service. This way we can reduce mother and child morbidity and mortality significantly.

What kind of challenges do you face to go to the community and offer the necessary ICCM service?

Since last year more than 1000 people have arrived from other places and become residents of this neighborhood. But the resettlement is against the law. Accordingly, with a small number of workers / four HEWs / it is too difficult to give service to a large number of people.

Have you devised any system to assist the new arrivals?

Yes, we have made some attempts to assist them. They were forced to leave their place. They have many kids with them. The children are highly malnourished. Their general wellbeing /health / is not good. Many of these children

are not immunized. Because of these reason, they get measles. Owing to these facts and circumstance, we have exerted the utmost effort to assist the people/ new comers/.

What other benefits did the community obtain because of the intervention?

Different health groups were formed to assist us to successfully accomplish our task. Our intervention area / kebele is very big. Because of this reason, not all people can be reached by four HEWs. Alternatively, the health groups will make job less hardship. In my view, the groups should be strengthened to lesson our hardship and reach as many people as possible.

The support given by JSI is commendable. The assistance is found to be extremely important to give better service to the community. JSI has provided the health post training and material assistance to reinforce our capacity. It is difficult to exhaustively write down the assistance given by JSI. Before the intervention by JSI, we had only one room in which we used to give service to the community. The room was very small in size.

But, now the number of rooms has grown to four. Lots of people including community members, religious leaders, school principals and teachers, kebele leaders, goot health leaders / coordinators, development groups, and community leaders have attended training programs to improve the quality of service being given to the public. We are working in close collaboration with the aforementioned groups of people to make our service better in quality.

This project is now underway . Do you think it is feasible or capable of being achieved if the project is implemented across the country?

I think the goot leaders group is an excellent example that deserves to be imitated all over the country. Goot leaders are to be counted on. Therefore, these groups should be formed and strengthened in the whole country.

Unless we have a structure/ organize people into whole, we cannot bring the desired result. We the necessary structure, we can easily mobilize the community for action.

Which of the activities you have been implementing until now do you think are easy or not requiring much effort, and can be adopted elsewhere across the country?

I think ECHI can bear fruit if it is implemented in all parts of the country. It is also advisable if the information is accessed through a computer network. In this way we can ease workers' burden.

What about the application of medical care? which of the medical services can be easily imitated in other parts of the country?

The necessary inputs must be available and wage paid to a worker must be improved to provide appropriate medical help to children. trainings must be given to stakeholders to refresh their memory with a piece of information.

In this way we can remove burden or difficulty from the health center. The community can easily get service. Services given to newly born must be strengthened everywhere.

How do you integrate ICCM in your usual pattern of activity?

We have included ICCM in our routine. We make our plan of action. The health post is always open for clients seeking treatment. Two of the HEWs will be engaged in fieldwork and the remaining /two/HEWs / will remain in the office to undertake their task. HEWs who go for a fieldwork take ICCM with them. They give treatment to children affected by illness and education to adults.

How did you incorporate treatment given to newly born?

We make a plan to treat newly born and children under-five. We have quarterly, by-annual and annual action plan. We carry out our task as per the plan of action.

What types of activities are included in your plan of action?

Provision of vitamins and de-worming to children, nutritional screening, food demonstration, child treatment, and growth monitoring are included in our plan of action. Nutritional screening of children under-five and pregnant and lactating mothers is done on a monthly basis.

Do you have anything think is important to tell us that we have not asked you?

No. I discussed and explained what I know.

Thank you

Area Identification Amhara region

Name of facility: HP

Date of discussion 15/6/22

Start time: 1:40

End time: 3:20

Can you please describe the demand generation/SBCC activities you are doing on ICCM/ newborn health?

In the past the treatment was not given to new born here. Next we have organized awareness creation program for kebele leaders. Then the kebele leadrs started disseminating information for the community and reached all catchments and used places where people gathered together (Idir).

Originally, places where many people gathered recurrently were identified.Then, have provided the community with information about new born child health care service underway at the health post. After that we made religious leaders, and the community at large familiarized with the program by using the available structure / Women's Development Army / WDA/ at all level. We have communicated information to members of the community to bring their children to the health post to get them ICCM service. Then, the community has started to make use of the service. And we have found out that members of the community have great deal of interest to acquire the service.

Please tell me about the strategies you used to make the community aware of the program?

We have already established a task force that is performing at kebele level. Members of the group / task force/ are having a meeting at the kebele administration office every week.

Then, we go to the people attending a meeting and give them information about the launch of neonate health care in the health post.

We conducted an assessment on the number of new born died without accessing health care service. We informed people attending the gathering about the finding of the assessment. We familiarize them with the subject concerning the number of children dying because of lack of health care service in the past.

Following immediately after the introductory information, they are required to travel to different places within the kebele to publicize to the community about the start of neonate health care.

Could you please describe other strategies you have been using to make the community familiarize with the program? Any other method you have been using to give information to the community?

We have been using WDA, youth league, religious institutions and Community Based Organizations / CBOs/ such as Idir that are found at village level.

Then, kebele leaders who are higher in rank or responsibility went to the people attending the meeting and made them familiarize with the start of new born health care service in the neighborhood.

During the monthly meeting of WDAs we used to discuss things that could be done by the health post. The primary responsibility of WDAs is to mobilize the community to bring their children to health post to acquire health care service.

They also mobilize and give strong advice to pregnant mothers to attend prenatal care in the health post. In addition, they urge them to take part in pregnant mothers ‘conference and deliver their child in the health facility.

WDAs pass on important information to the community. They have provided us with the means to achieve the desired result. We give them training to WDAs and review their success and weakness every month.

Even though WDA is not working actively we have organized training program for members of WDAs. The trainees were told to move to different places in the village, gather data pertaining to newborn and finally let the health extension worker know.

We let them know that new born affected by illness should be brought to the health post to get health care service.

You have mentioned that WDA is not as active as it was before? Why?

The women who are in lead or directing others to follow have been offering service for more than eleven years now. They are given no incentive to encourage them to action.

Neither the kebele nor the woreda health office gives them incentive in cash or in kind to motivate them to carry out their duty. Then COVID-19 emerged and became another obstacle to conduct a meeting with them.

After that, members of WDAs have stopped coming together. We are unable to bring them together because of the pandemic. Owing to the above circumstances, we are unable to make the best use of their time and knowledge. We were planning to change members of the group who worked for long time on several occasions. But, we weren’t able to gather members altogether.

You have mentioned that you were planning to replace some of WDAs. What barriers prevented you from making the replacement?

It was impossible to bring members of the group together to make substitution. Usually, we find them inside the church attending religious service. But, after attending the church program they go back home to discharge social

obligation. They have no enough time to attend the meeting. When this gap happened the kebele leaders should have given us some assistance. But, it doesn't give us any kind of support because there is high staff turnover. The outgoing and incoming staffs don't pay serious attention to our problem. They are too preoccupied to notice what is going on as far as our activities are concerned.

You told me "COVID-19 has an impact" why?

It was not possible or allowed to gather many people at the same place and time because of coronavirus pandemic. And, after a particular period of time, members of WDAs have become reluctant to attend a meeting, review their performance and submit a report.

However, they have continued to assist us whenever we go to the villages. They go from house to house with HEWs and give them the necessary support. But, they are unenthusiastic to attend a meeting.

How is the engagement of communities and WDA networks in the implementation of integrated community-based case management of common childhood/serious neonatal illnesses?

The communities are happy by the ICCM and neonatal health care service they got from the health post.. Accordingly, the communities who are aware of the services are making effective use of the service provided by the health post. We inform the provision of the service and to bring the new born for any health problem for those who had no information when we go house to house for postnatal service delivery.

Mothers don't want to go to any health facility to get treatment before she performs ceremony baptism. The process is creating a lot of problems to give postnatal care. However, we go to the woman's house to give her postnatal care. and observe the general condition of the mother and the baby.

What do you do to help the woman gain access to postnatal care who is unwilling to come to the health post before she performs ceremony of baptism?

What we do is to teach the community to gain adequate know about serious signs of neonatal health problem that indicate the presence of a disease in the child's body and bring him / her to health post to get medical help.

The woman should be mindful of the fact that the newborn can be affected by any illness just two or three days after he / she was born. Therefore, we told them they don't get a problem when they come to the health post within ten days after giving birth. If they didn't accept this we advise them to inform HEWS by a telephone.

Did you observe any change on the health seeking behavior to that effect?

Yes, there are lots of changes. When their newborn gets sick, the mothers call and inform us by a telephone about the condition of their neonate.

In this way, there were a number of children who were completely cured. The mothers managed to quickly transmit the information to other households in their neighborhood.

Do others take part or participate in the activity of creating awareness? / Apart from WDAs/?

Recently we have established village health leaders group. Members of the group play intermediary role or carry message between the health post and WDAs.

Who initiated formation of the group?

The group was established in Megabit, 2013 E.C. Members of the group were given training by JSI. They are part of government structure. But, their role is to provide a link between WDAs and HEWs/ health post/.

Village health leaders were given some money for tea and coffee during the training. But, members of WDAs were displeased with the payment made to village health leaders.

WDAs claimed that they are entitled to get the money because of many-years of service they have been giving to the community. Thus, we tried hard to resolve the problem together with the kebele manager. We went to each member of WDAs to tell them the reality. We told them that village health leaders are their assistants. We have informed WDAs that village health leaders will not take their place or position. They act if our statements were true. But, they pretended to be interested in our idea.

How does their feeling or response affect your activity/service delivery?

WDAs are not able to submit a report to the health post because they are not able to read and write. Thanks to village health leaders, we can get any information without difficulty now. It is easier for us all to gather information from 12 village health leaders not from 28 members of WDAs.

What type of information do they give you?

They bring every Information relating to all the 16 health extension package. They keep all household data in their district / neighborhood and submit a report on a monthly basis.

They keep the necessary information about the number of toilets, men and women joined in a marriage recently, pregnant women who have started to attend prenatal care, women who delivered a baby at the health facility and started to attend postnatal care, and newborn who are inoculated.

Earlier you have mentioned that members of WDA were having grievance because of the money paid to village health leaders to drink tea and coffee during the training. How did their grievance affect your duty?

It has eroded their morale to carry out their task. We have exerted the utmost effort to convince them to change their mind.

How did you fill the gap?

What we did was just to try to convince them to accept our advice to keep giving service to the community. Additionally, we tried to fill the place of those women with many years of service with other women. But, the new ones haven't started working.

Why?

Because we are not able to get together with the women by arrangement. The kebele leadership has not given us any support to meet with the newly elected women. We wrote them a letter to do the job. Then the kebele leader told them to come. But they did not come.

In your opinion what should be done to persuade them to begin to carry out their task?

Their grievance is caused by lack of financial incentive. My recommendation is to make coffee and tea, invite them and discuss the problem. They must be accountable to the kebele leadership and their performance must be reviewed by same.

It is only HEWs who ask village health leaders to submit performance report every month. The assistant assigned by the health center does not watch over their activity. It will be good if the woreda health office arranges a meeting every quarter to make them feel interested to do their job.

How do you see the contribution of village health leaders to improve ICCM/newborn health care service?

Before village health leaders joined the health post, it was not common to review activities associated with the provision of medical help to children in general and newborn in particular.

The quality of our working effectiveness began to decrease. Then we organized a one-day meeting in Debremarkos in which we discussed the problem. We decided to make the best use of the existing structures to bring about the desired change.

Next, we familiarized the kebele leaders with the new initiative/ plan. We discussed the plan with religious leaders. Finally, the number of people coming to the health post has increased.

Formerly, we used to give little attention to postnatal care. Currently we collect enough information about the general wellbeing of children in operational site.

How do you describe any other challenge you might have faced to build of knowledge of the community and familiarize the service with them?

Presently, we are not able to come across WDAs. We have not provided WDAs with the necessary information about our service the way we did for village health leaders.

We were planning to organize a conference on newborn health. But, we were not successful. We need the assistant of the kebele leaders to bring the people together.

We have no right or power to enforce rules or give orders to gather all the kebele residential. The kebele leaders don't turn down our request. But, they give different reasons not to make the request practical. They give focus to their regular activity. We only meet Kebele leaders on Sunday. They have other responsibilities on other days. Besides they were ordered to do other emergency activities on that single day/Sunday/ from the woreda.

Did do you get the support system ,mentorship, on-site coaching methods, supervision, technical support and PHCU level PRCMM helpful to enhance your skills over the traditional off-site training and woreda level review meetings?

JSI is owner of this support. Before JSI began to implement the support, provision of health service was business as usual for us all. We have made an assessment of our working effectiveness after which the findings were discussed at a review meeting in the city of Debremarkos with JSI.

We discussed the scale of the problem. We were well-informed about the magnitude of the problem hampering provision of health services for the community.

This is how the project has given us support to achieve a goal. We ought to improve the level of awareness of the community to achieve our objectives. We were also required to know the scale of the problem to do our job successfully.

The reason why we decided to find out the scale of the problem was to address child morbidity and mortality. Our primary objective was not merely to give medical care to whoever comes to the health post. It was also to identify the cause of their death. We are feeling regret to see new born dying in a week after they are born.

So, we discussed the problem at the health center. Then the organization has continued to do so and ICCM focal persons at the health center tried to find out the existing gaps to provide appropriate service for the community.

How do you describe your efforts to provide ICCM/new born health care?

The way in which we did our job was good despite some gaps. But, we would have done more had it not been for the aforementioned challenges. Emotionally we are very close to mothers related to emotion.

We advise them to be careful of their health when they come to the health post to get vaccine and attend mothers' conference. Demanding job has been done by religious leaders to achieve the desired result.

In past we used to give focus to mothers and children affected by illness. We came across two or three people who were having a disease. The mother used to believe that her child is affected by minor illness. But currently, we find the situation to be different.

How do you think the COVID-19 pandemic and/or COVID-19 response measures affect the uptake and/or delivery of ICCM services?

Protecting ourselves from corona virus pandemic was a question of life and death for us all. Therefore, at that time we paid no attention to our regular activity.

We used a face mask and sanitizer wherever we went to carry out our task. We used to offer advice to mothers to practice social and physical distancing to avoid infection by the virus. We have tried as much as possible to educate the community about the mode of transmission of COVID-19 from one person to another.

We used to get the usual kind of support after the emergence of COVId-19. But we were supplied a face mask and sanitizer by the health center. We were informed to pay equal attention to all kinds of activities.

What were the challenges you encountered on the delivery of ICCM/neonatal health care to community during COVID-19?

At that time building the level of knowledge of the community was out of question. It was strange even to be considered. Going from one household to the other was extremely tiresome.

It was not possible to use the existing structures to do our job. We were able to gain access to only a small number of family units. Everybody thought that they would be taken to place of isolation if they were seen coughing.

Because of this reason, members of the community would stay in their home even if they were affected by sickness.

As you have mentioned earlier the community did not come to the health post because of COVID-19. It was HEWs who used to go to the community to pass on information. Apart from a facemask and sanitizer support how do you describe the strength of the support you got from the project /PHCU to address barriers to deliver ICCM/PSBI during COVID-19?

After the onset of coronavirus pandemic HEWs were given instruction to increase the frequency of house-to-house visit. Accordingly, we passed on information to the community about the mode of transmission of the virus from one person to the other and its prevention. We offered them not to go to another place without covering their face with a mask and using sanitizer.

How do you describe your strength and devotion to deliver ICCM/PSBI at the time of coronavirus pandemic?

We were having workload. There were many people in the area who have been repatriated from Arab countries.

Therefore, we were required to make an assessment about the general physical wellbeing of the people who were sent back home. We would give information to the health center about these people.

We used to gather information about people suspected of being infected by the virus. The work was boring because of being repetitive or unvaried.

What are the high-level benefits that are attributable to this support/IR?

The support was very beneficial. But the community has now started to pay attention to the pandemic.

What do you think should have been done differently to do and achieve better result?

A. we would have done a lot of things if we managed to bring set of circumstances caused by the onset of COVID-19 in a short time. We should have designed a system to make the existing structures start to carry out their task soon.

WDAs were not working anything for long time following the emergence of coronavirus pandemic. But we were not able to bring the structures/ the groups back to work immediately.

We let the structures scatter in different directions in careless manner. The kebele administrators failed to design a strategy to make the structures continue to carry out their duty.

How much did the technical support you have been offered by the project or the health center during the last one year build your capacity or skill?

Not all issues about which we have learned previously can be remembered. Also we may not put some ideas into practice because of lack of concern. Therefore, it is vital for them to point out or mark the errors we have made. It is also important for them to show appreciation for all the good things we done.

They are also required to observe the quality of service being offered to patients. A fair assessment of our performance will help us improve our skill to carry out our activity.

What difference did you observe between the previous capacity building trainings you were given before and on-site coaching methods/ a follow up support you have been getting since last year?

Currently the case being examined or investigated has increased. we give treatment to patients by using the booklet containing information about the people using our service.

In past health workers were coming here from the health to give us professional advice or support. But, the support has come to an end for an extended period of time. Then, we have continued to give service to the community by using the old methods.

Most of cases investigated by our health post are about children who are from two to fifty-nine months old. On one occasion only a small number of newborn / two months old/ were brought to the health post to get their case examined. We have found out that majority of the children dying are those who were born very recently or who are two or less than two months old.

On the other hand, we lacked the confidence needed to give medical aid to the newly born. we attribute the success achieved in a rise in the number of cases being investigated by our health post to the support we have been given.

During the training everybody assumes that he/ she has properly understood the information being communicated. But, we may face trouble to put the knowledge acquired into practice.

Thus, we ask them by a telephone to give us clarification about the issue. We have found the system very important. I think if these activities are reviewed on a quarterly basis, they will renew everybody’s energy.

Similarly, they will enhance our capacity to do a job good enough to meet a requirement. In addition, our capacity will be improved if people from the health center who have attended training program are assigned here to give us support by looking at the cases being investigated.

How do you think they can assist you?

We will show them the file containing list of patients who have been given treatment. Then, we will inform them about the type of illness against which treatment was given, a drug or other medication prescribed by a HEWs and date of appointment etc.

They will rectify imperfection / deficiency / if any/. They will give us advice to make our service better in quality. We cannot acquire adequate knowledge when we attend training en masse.

Who gave you the support? How often did they give you the support?

Gashe Zemed / the project’s focal person/ has given us the support. He is coming here regularly to oversee how the task is done. As soon as he arrives here, he wants us to tell him about the problems we encountered when we accomplished our task.

Then, he requests us to show him list of names of patients who were given treatment. After that he asks us to tell him about the items we used to treat patients.

Next he raises some issues purely hypothetical and gives us an order to deal with it. He tells us not to carry the book for official records when we go to the community. He is telling us to use a piece of paper to record the information in writing.

You have mentioned that the quality of service declines when it is given in the community. What is the reason for the decline in the quality of service?

When we go to the community, we may forget to give treatment to some children. We may also forget to carry some tools used for treatment with us. But, when the children are brought here, they can get complete service with nothing let out. It is difficult to properly use weighing scale in the community.

You said that you lacked confidence to examine or investigate a case. How did you come to acquire confidence?

Incidentally, the children to whom we gave treatment were cured. That condition gave us hope or confidence to keep on giving treatment to a number of children.

Can you please describe about the confusion you have encountered?

Currently, a dose of drug is injected into the body of newborn between zero and two months old for two days. Formerly, the drug was administered for seven days. We were scared to administer the drug / gentamicin for seven consecutive days. A reduction in the number of days during which the drug is given to the child has built our confidence.

Did value or appreciate the support you were given highly?

Yes, we did.

What factors made you feel happy?

A review of our performance should be made regularly. Health workers should come to the health post regularly and give us a particular situation or event to take a look at. This issue will help us build the level of our knowledge to improve our working effectiveness.

How do you compare the support you have obtained currently and previously in order to discover similarities and differences between them?

The support they gave us previously paid little attention to quality of service. Its main focus was quantity but not quality. Now it easy for a supervisor to go to the community and find out quality of service being rendered. They can ask the community to ensure that the report submitted by HEWS is correct and credible. Therefore, the support we are getting now has improved the quality of service and capacity of the workers.

In your opinion hhat are the gaps that need to be addressed to improve the general standard of service given to the community?

I suggest the presence of important entities such as religious leaders and WDGs in a meeting as good idea. It is also important if people in charge of the entire operation find a way to make the job of carrying a kit to the community easy or easier.

For example, we use a bag to carry different items when we go to the community. we do not have appropriate item to lay the baby down and measure his/ her weight. I also suggest to buy sheets of paper for writing instead of removing individual paper from a document as good idea. We also would like them to give us pens and pencils for writing.

It will be good if they arrange a meeting at a woreda level to make us feel more energetic or renew our energy. The booklet we are using now is updated with more recent information than was formerly available. Therefore, we need to be given a training to make us familiar with the new information.

How ECHIS implementation helps you with ICCM service delivery

We are working on ECHIS now. We are not doing anything about ICCM using our mobile phone. I hope we be able to give good quality service to the community when we start using electronic community health information system/ ECHIS /.

Let us talk about child vaccination. The fact that we have written down the names of the children, it will put the children eligible to get treatment in a list. If the mother fails to bring her child within two days as per the appointment, the mobile phone will show us yellow color. But if the children are eligible to get treatment on this day, the mobile phone will show us green color. When the mobile phone gives out a red color, it means that the children in question have not received any treatment.

If the child is ordered to use a particular drug or penta on this day and if penta three is not available in the storage, we will observe a change of color after two or three days on the mobile phone. Thus, we will consider the child as a defaulter. We can observe similar situations If a pregnant woman who has attended the initial postnatal care but failed to attend postnatal care for the second time. The mobile phone will tell me whether the woman has delivered her baby or stopped coming to the health facility to attend postnatal care. Therefore, the information will lead me in the right direction to provide service for community members being considered.

How do you think the assistance would facilitate the implementation of ICCM if it was implemented?

It will make danger sign visible. In addition, if I jot down mistaken figure, it will not let me carry on my job. It will show me a red color. I assume the system will help us provide the community with good quality service. Furthermore, I might have an appointment to meet people who need service. But, I may not remember the date of appointment if it is written on paper. So, the new system put in place helps us to carry out our task with ease. It is useful to prescribe appropriate drug or other medication for a patient. For example, a person might have been affected by pneumonia or an illness causing coughing. Also, he might have high body temperature. I may be required to order use of paracetamol by the patient. But if I recommend any other treatment / or drug to the patient instead of paracetamol, the system in place will not allow me to do so. It will let me know the type of drug I must prescribe for the person affected by illness. In this way, quality of medical care shall not be compromised.

How do you describe the advantage of ECHIS to retrieve or manipulate data?

For example if a woman wants to get family planning service in the health post, it takes long time to find all information from the file contained in the shelf. Firstly, I must get information from the woman about her physical location. Then I have to look into the content. But, the information about the woman can be easily accessed or

retrieved through a computer network. We can save our time and energy by using a computer program that searches for specific instructions and returns a record of events.

You have told me that ICCM service delivery has not been made practical. What do you think needs to strengthened to improve service delivery pertaining to ECHIS and ICCM?

Firstly, all data in a hard copy must be stored on a computer disk. But, the information must be available in Amharic language. we don't have a good command of English language. Because of this reason, we may report the information to other people in an inaccurate or misleading way. When we start using electronic health information system, we will be able to see or understand everything easily.

Describe the main issues faced by the health system to identify and treat neonatal infections and to manage ICCM in the community?

There was no problem to identify the infection. The booklet will help us recognize the illness. But, we were facing a problem to dilute gentamicin delivered to the health post by the health center. The condition remained a source of misunderstanding for some time. But later, they have showed us how to dilute or make the drug thinner. Then, we started to give diluted or thinned gentamicin to our patients. I think the health post must consider of providing diluted gentamicin to the health post to make our job easier. In past they used to give us diluted gentamicin. We were giving a measured quantity of thinned gentamicin to the patient in accordance with the guideline. But currently they give us the powder after which we make it thin by using distilled water.

Why is it difficult for you to dilute the powder?

Usually, we are unable to remember to determine the ratio of the water to the powder to be diluted.

Did you face any other challenge to identify disease or sickness affecting newborn?

We did not face any problem. We use a timer to find out the process of taking air into the lungs and pushing it out again.

What other problems did you face to deliver the necessary service / treatment for children under-five including neonates/less than 2 month/ ?

We want to see the existing structures strengthened. They will make the frequency of our journey to the community less. They will make our job easier. WDAs will help us build the level of awareness of the community. I hope we will make them stronger if the kebele leaders and the woreda administration assist us.

Is there any other input you may require?

A. we need appropriate facility to measure to weigh / find out the weight of children. at one time the thermometer was not working. Likewise, we were given a new thermometer.

Is there any skill gap?

We are human beings. We have heavy workload. We don’t read the guidelines thoroughly. Usually, the knowledge we gain from a training is not in agreement with practice. Anyway, we need a training to refresh our energy or feel more energetic to efficiently do our job.

The training on ICCM was lasting for only a short time. In past we used to attend training on ICCM for six days. After that, we have not attended any training. We need a minimum of ten days to acquire basic knowledge about ICCM. We investigate a small number of cases when are trained by skilled professional at the health center.

The number of trainees is big. I don’t think I am mistaken if I say the training was organized merely to make available for others to see. They must show us the practical application of the skill gained from the training. It is also important to use hospitals to transmit knowledge for the trainees.

How do you think the COVID-19 pandemic and/or COVID-19 response measures such as the state of emergency and curfew affect the uptake and/or delivery of iCCM services?

It was not having any effect.

How did the conflict in some parts of the region, which are found near to you, affect delivery of services?

The community was mentally disturbed by the conflict. People used to give more attention to the conflict. Even we were psychologically unstable. We were not able to go to the community and talk to them. However, there was no warfare between opposing forces in our neighborhood.

How did restriction of movement of people affect distribution / supply of inputs?

It did have no effect. We used to bring the necessary inputs to the health post whenever we wanted to.

How did COVID-19 prevented mothers from acquiring income to bring their children to health facility to get treated?

The community is not engaged in the activity of buying and selling goods. Agriculture is the main stay of the community at large. Therefore, I don’t think coronavirus pandemic was having any impact to that end. But, mothers were having difficult time to get delivery service, antenatal and postnatal care. They didn’t want to come to health facility because they were scared of infection by the pandemic.

Therefore, home delivery was becoming a norm. additionally, the community thought that service delivered by the health post decreased significantly.

They thought that anybody who showed act or sound of coughing was prohibited from seeking service at the health post. They believed that an act of coughing was a sign of infection by the virus.

Why did health workers refuse mothers, children and any other patient seeking treatment from coming to the health post?

Because they believed /thought that the community would be infected by COVID-19. Additionally, health workers lacked devotion to duty because of coronavirus pandemic.

Were mothers interested to bring their children to the health post during COVID-19?

They were less interested. They preferred to staying home to coming to health facility. We were searching for people with an illness causing coughing. We told these people to go to health facility to get service. But, they did not want to.

What did you do thereafter?

We informed the community that anybody who was showing an act of coughing could come to the health post at any time he wanted to. We told them to save the lives of their family by seeking help at health facility. Some people showed willingness and the others didn't.

What any changes did you observe as the result of the implementation of the COVID-19 adaptive ICCM implementation strategies?

We used to go to the community following the onset of COVID-19. But, our focus was coronavirus pandemic. ICCM was a secondary importance for us all. We did not take any kit with us. We were provided with all essential support to give other kinds of treatment to the community but not ICCM. We were given advice to keep our selves save from COVID-19.

Did you any job to identify ICCM during COVId-19? Did you take any action?

No.

What was the reason for lack of any kind of support at that time?

We would have done a lot of things if they managed to provide us with the necessary support. Lack of devotion and support on the part of our superiors were to blame for low performance.

What are the high-level benefits that are attributable to this support/IR?

We found the short-term trainings very useful for bus. The support we obtained from the health center is producing a good or advantageous effect. They used to come to the health post to show us how to investigate a particular case.

They took a look at every case and pointed out or marked errors. They offered us advice not to make an arbitrary decision based solely on personal wishes.

Which one of these assistances and encouragements did you find very important?

We possess the necessary skill to do a job. But, we can perform better if we organize the community and resources to be ready for action. Mobilizing the community is not a one-time activity.

We should keep on preparing people including WDGs for action to ensure sustainability of provision of the service. Anyway, a review of HEWs performance conducted on a regular basis was found very important. It provided us with the means or opportunity to put emphasis on mobilization of the community.

What about the support given to build your skill?

The support has improved our ability to do our job well.

Please explain to us the feasibility of this support/ IR for national scale-up?

I think the short-term training is very useful. I assume we can work together with the experts assigned by the health center to give support solely on the implementation of ICCM. We can make a call to the experts and obtain their service or advice very easily. We communicate with the experts whenever we refer anybody to the health center to get help.

They are also coming to our health post to find out the number of cases investigated by HEWS. But, we must get training opportunity to improve our working effectiveness. But, the training need not last long.

Two-day training is enough for us. The purpose of the training is just to refresh our energy. We have already acquired the knowledge to carry out our task.

We should keep on mobilizing the community to own the service. The community must be educated to get treatment which is given for free. We have the necessary facilities in place. But, the existing structures at all levels should be made functional.

Other regions can use our structures as a model to implement similar activities. It is possible to do the job at a health post level. WDGs have strong feeling about mothers and children.

Because of this reason, they can produce a desired result. But, members of WDGs must be given incentive that encourages them to action.

In my opinion the community must come here to get treatment. But, we are required to invest a lot of energy and time to go to the community to give treatment.

Besides, quality of services will be highly compromised. It is not comfortable to give treatment in the persons'/ child's residence. Making a trip to the village is time consuming and causing fatigue.

How the activities/efforts are embedded in your routines/work streams? What implementation strategies are incorporated with the health post annual work plan?

ICCM is part of our usual activity. It is included in our plan of action. We often strive to increase the percentage of children in the target area who are reached by medical service. We try hard to achieve our target and provide good quality service to the community. To attain our goal, we go to the community to give postnatal care to mothers. We identify pregnant mothers to inform them attend prenatal care and deliver their baby in the health post.

We also advise them to attend postnatal care. We go to the community and do assessment of the health of mother and the newly born. We give them treatment if they are affected by illness. We put everything into a package when we go to the community.

How did COVID-19 affect your routine?

It was having a great deal of effect. We were required to go to the community at short intervals to find out people infected by COVID-19. We were frightened by the spreading of the disease rapidly.

Your main emphasis was the prevention of the spreading of coronavirus pandemic. How did this condition affect delivery of ICCM service?

We give little attention to ICCM. Our concern was to give information to the community about the mode of transmission of the pandemic from one person to the other. As a result the number of people who seeks health care service decreased during COVID pandemic.

However, nobody has died because of lack of medical care. If people were not interested to come to the health post, they would go the health center to get medical help. They also went to private health facilities to get treatment.

As you mentioned you faced difficulty to prepare Gentamicin. Did you face any problem to offer gentamicin to the patient?

No, we did not. But, one must have confidence in his / her ability to administer the drug. At one time, we went to the community to give postnatal care. Then, someone told us that a woman has delivered twins in a hospital.

We know that we referred to the health center to get delivery service. When we went to the woman’s house to visit her, we were told that one of the twins has died. The one who was alive was seriously sick. We asked the woman to carry the baby and go to the health post with us. But, she has refused to.

Then we asked her neighbor to lend her “Ankelba” to carry her baby on her back. Then we managed to bring the woman and the newborn to the health post. We examined the baby and give her treatment. Then, we told her to

bring her child to the health post for consecutive seven days. Finally, the baby was cured. And now the parents and their neig

Area Identification Amhara region

Name of facility: HP

Date of discussion 16/6/22

Start time: 9:05AM

End time: 10: 30AM

Can you please describe the demand generation/SBCC activities you are doing on ICCM/ newborn health?

We have been given a number of training programs to build our skill and improve the wellbeing of newly born who are less than two-months old and children under-five. But after sometime, there were some gaps in providing the necessary support to newborn less than two months old. We appointed the mother for postnatal follow up but we didn't register the baby. Very recently we have attached informative item about the newly born baby during postnatal follow up assessing the health condition of the baby.

Additionally, we just used to refer the neonate to the health center when they got sick and brought to our health post to get medical care. Accordingly, Gashe Zemed looked into the file in detail after which he found out lots of gaps pertaining to information about young child less than 2 months old..

Gashe Zemed was giving particular /serious consideration to our health post. He followed our health post every day. In this year we treated 5 children for sepsis and a good number have been treated against local bacterial infection. Also, considerable numbers of newborn who are less than two months old have been treated against pneumonia. But last year we didn't do same. Presently, we assess and attach descriptive word or phrase about the health condition of the newborn by using the guide booklet. We make every attempt to be as correct as possible following the guide whether the child affected by illness or not during postnatal follow up.

What did you do to improve the level of awareness of members of the community about ICCM/neonatal health care service?

HEW, Kebele chair man ,kebele leader let the community in the kebele know about and acknowledge the work done for child health service by the health post.

What are the activities you did to let them know about the service?

assigned to different "Gott" and went to churches during religious service to pass on information to attendees. Firstly, we let the kebele leadership know about

For example, the community contributed birr 700.00 to make a banner to display information pertaining child health care service which is given in the health post. We displayed the banner in the health post. After that we educated and offered the community advice not to take every sick child to the health center before they seek medical care in the health post. We told them the availability of each required medication in the health post and about the training we have taken to treat sick baby .

We told them as we will refer the child to health center if the case is beyond our capacity to treat so not allowed to go to health center before visiting us. You will waste your time and energy when you go to the health center for similar treatment to the health post.

In addition, after birth/ during postnatal care the women are offered advice to bring the new born to health post if affected by illness. Next, all the women began to consult HEWs.

What are the strategies used to raise awareness?

This year because of different campaigns we have planned and organized series of journey to every household. We used to work in the health post for a very limited number of days. Almost all we have been going house to house in the community for the whole year. This creates good opportunity to get each member of the household so we talked to pregnant and lactating mothers and assess the new born baby and register for further follow up and give appointment for postnatal follow up.

What did you do to aware them about ICCM and neonatal health care when you visited every household?

We gave special attention to all new born baby between zero and two months old. Mothers should never take new born babies to health center before visited us because our newborn coverage was less. Other children above 2 months old and under-five are brought here to acquire essential service irrespective of the type of illness. Mothers only took their under five children to the health center when they didn't get u(when the health post is closed).

We have taken part in the training of ICCM and CBNC together however there was gap in service provision for neonates.

What did you write on the banner?

There were a number of statements on the banner giving people information pertaining to child health and illness. It was stated on the banner that neonates below two months old and children under-five can acquire medical services from the health post. It was also mentioned that vaccination, prenatal, postnatal care and other 17 package services were mentioned.

Why did you go to see every household?

For example we gave vaccine to children against polio twice. Then MAUWC screening from 6 month up to 5 years old, assessment of COVID infected individual and then COVID vaccine. All these activities were done house to house. Next we carried out provision of medication house to house to prevent people against trachoma / eye disease. Today we have launched a campaign against corona virus pandemic.. Owing to these facts and circumstances, we have got a chance to get and communicate with the community. When we go to provide all the above services we carry other medication and supplements to treat a child who is sick. The child who has severe disease is referred to health center.

How is the engagement of communities/WDA networks in the implementation of ICCM/PSBI?

At the beginning members of WDAs were given training along with members of the kebele leadership. It is clear for every one that WDAs were getting weaker following the reform within the government. They are not working actively following our day to day activities. Recently, one Wash-Wash project started to implement some activities in this area. The project has given training for 25 members of WDAs with some incentives.

The training was having significant content related to seventeen health extension package. We meet them every month to discuss about the health of the community. Gash Zemed gave one-day training to members of WDAs. Newborn was the training's focus / main emphasis including main causes of death for new born.

We have told the trainees that HEWs are capable of applying medical care to cure diseases irrespective of severity of same. We informed them that nobody is allowed to seek medical help at the health center without obtaining reference from the health post. Those who are near to the health center only take the new born to get health care.

How do you assess the degree of their involvement after the training?

They are making a great deal of participation. There is a woman who is a traditional healer for children in our community. After the training WDAs motivate or persuade the women to get their child treated in the health post rather than taking to a traditional healer. They follow if there is a woman who planned to get treatment from traditional healer in secret. A case in point is a woman/ a member of WDAs/ and her name is Meaza who persuades members of the community to bring their child to the health post by reasoning, pleading or coaxing when they planned to take the baby to traditional healer. And then she gives the information to us to treat the baby before they go to there.

What about other WDAs participation?

But not all WDAS members are involved in busy activity. Not all civil servants are hardworking let alone peasants(WDAs). However, majority of WDAs members are tending to work industriously. Some of them are using mobile phone.

We don’t involve the men in the activity. But they build a toilet. Agricultural sector uses male participants. We involve the women in accordance with government strategy. We have invested a lot of resources to give them training. However, if any one of them decides to leave the group, we take somebody that replaces her.

Did you involve any other entity in the activity?

Yes, we did. Gash Zemed We caused school principal, the kebele manager and religious leaders to participate in one day training.

What did school principals and kebele leaders do in order to achieve the desired result?

I cannot say anything concerning the role they played in achieving the goal. However, we made them familiar with the services offered by the health post and to be assertive and use any opportunity to transfer the message to the community.

Describe the main challenge faced by the health system to identify and treat neonatal infections in the community. What are the critical factors affecting the delivery of iCCM to clients?

They complain about supply shortage of drugs on many occasions. They always want to make sure that the health post has enough drugs in the storage. We confirmed to them the availability of supplies.

At one time there was scarcity of gentamicin. But, we have no shortage of Gentamicin and any other medicine now. We have enough Genta, amoxicillin, Zinc and other in our storage.

What kind of challenge did you face when you tried hard to change the behavior of the community to make effective use of the service provided?

We have not encountered any challenge worth mentioning. But, many people live in the surrounding area/ near the health center. They don’t want to come to our health posts, Angote Degera, because it is located a great distance away from their village. The health center is near for their residential place.

How does the traditional healing practice affect your activities after you aware the community?

We have no problem to that effect. But traditional healing practice has become a source of income for the woman. A good numbers of people are going to the woman from various directions to get cured from their illness. A lot of people are queuing while waiting for service. If we go today we can get 400 - 500 children.

I have informed a physician to undertake small-scale study concerning the service offered by the so-called traditional healer.

To our surprise, the woman cures or treats illness. We are seeing children affected by different types of illness such as back pain, wasted child, a child who has difficulty to walk and others gets cured and able to walk. Thus, it is difficult to forbid her from practicing the job. She only applies liquid from plant part on the child’s head and body

to cure the illness not by mouth. I myself went to her house and saw the number of children. The type of cases for example hydrocephalus and other sever cases that I saw there made me feel horror or disgust. Her treatment has no effect in our work.

After the onset of COVID-19, what kind of support did you obtain either from the project or the health center to address barriers in implementing ICCM /PSBI?

We have obtained continuing support despite problems. They looked at our registration book. They evaluated our performance. They acquired information about the number of children born every week.

Ato Zemed from the JSI was visiting us together with the project staff. Also Tigist and some other health workers from the health center and woreda health office used to come to our health post establish that our performance was correct. Sometimes we may make mistake when provide health care service and write down information on the book.

They checked the registration book and asked us why we classified as Sever disease, Pneumonia, Sepsis and the like and also about the treatment given. So, they offer us advice to take reasonable care to avoid mistakes. If we did not do any mistake, they used to express a feeling of gratitude.

How do you describe the strength of your efforts to address barriers to deliver ICCM/PSBI during COVID-19?

We used to carry different types of drugs with us when we went to house to house. When we went to the woman immediately or soon after birth for postnatal follow up, we gave serious attention to the newborn. We asked her about the general wellbeing of the child.

We advise her to take the utmost care to avoid all risks affecting her child. We tell the woman to bring the child to the health post if he/ she refuse to suck breast milk immediately.

But, not possible to give regular focus/attention on each activity of ICCM/new born health care because we have a difficult responsibility / duty and work load.

One day when we go house to house, we came across a baby who was affected by illness. The child refused to get breast-fed and had difficulty to breath. Later, we found out that the child was affected by serious illness. Then, we gave Gentamicin and referred him to the health center. After that, the child was again referred and admitted to hospital. The child was cured and discharged from hospital in two days. The family acknowledged our works and witnessed that our child gets cured because of your visit. If you didn't refer, the baby would die.

On one occasion, I was alone in the health post. My workmates were not around. I went to a woman house for postnatal visit. When I examined the new born baby, she did not breast-feed and urinate. The mother did not give serious attention to the problem hoping to start feeding tomorrow.

The baby was crying accompanied by intense emotion. After that I referred the child to the health center. Next the baby was referred to hospital. The child was cured after short time. Because of this the mother says the baby the child of Yezenash. (the name of the HEW who refered the baby is Yezenash).

What things do you think should have been done differently to improve ICCM/new born health care service provision?

Everything depends on our strength and commitment to duty not only the presence of support. People may say or comment a lot of things about the work done by HEWs but improvement and achievement depends on motivation and commitment of HEWs. The owners of the activities are HEWs.

We are evaluated in the health center by our boss to make sure whether or not we have adhered to the guideline when classifying and treating child cases. We exchanged the registration book to each other and evaluated each activity done according to the guide line. Health workers from the health center draw comparisons between achievements gained by different health posts.

But, we are facing a number of difficulties to be overcome. Thanks God for letting us stay alive. The presence of workload is a challenge not to do what we plan and want to do. When we are busy we forget planned activities and lose motivation. For example last time One-WASH began to implement some activities associated with toilet. And the community is challenging HEWs activity. When we tell the community the activities to be done, they act as they know it before and nothing is new. They were not willing to prepare the toilet. Because of this, some gaps have been created when we started to involve in One –WASH project. At that time we just called the villagers and asked them about the number of children born, and affected by sickness. We didn't go house to house to visit the new born in person. We only went to house when WDAs tell us the presence of sick child. I am telling you the truth how and what we did.

The responsibilities of HEWs are becoming demanding or requiring a lot of time, energy and attention.

We did not have enough time to go to the villages to visit them. Majority of the women in this neighborhood deliver their child either in the health center or hospital. Only a small number of women give birth to a baby in their home.

Yes you are busy and have so many responsibilities. You have been doing different activities regarding ICCM/new born health care. So what things do you think should have been done differently to improve ICCM/new born health care service provision?

All HEWs took the training either once or twice. Besides the training there is a guide.. So, no need to give training except for those HEWs who are new.

As I told you before HEWs and professional from health center evaluates our performance in HC having the registration book. But last time the working-effectiveness and performance of HEWs was evaluated at zonal level. Regional and zonal officers were in attendance of the meeting. We bring all our documents to a place where performance evaluation takes place. On this performance evaluation Gash Zemed prepared and displayed on a computer how each health post performed ICCM/PSBI. By doing this, they identified health posts that did in a better way and not(that had gaps). Some health posts may perform better than others. I think it is good if they keep on organizing the event. It is important to organize this type of event on a quarterly basis to make us feel more energetic and exchange information.

Did you get the mentorship and on-site coaching methods, supervision, technical support, PHCU level PRCMM, etc. helpful to enhance your skills when compare with the previous off-site training and woreda level review meetings?

We have found the training given by Ato Zmede very useful. He comes to the place where we are working and studies problems caused by lack of capacity. For example let's say 25 mothers gave birth and registered for postnatal care. From these we might examine and classified the health condition of three new born babies only. Gash Zemed asked us why the other 22 new born babies not visited. Thus, he can easily identify the shortfall / underperformance. This condition shows a failure to meet a goal or requirement.

He pays serious attention to the cause for failing to conduct postnatal follow up and examine the health condition for the remaining 22 new born babies. He questions HEWs whether the problem was caused by negligence or workload or any other reason. He tries hard to discover the real cause why we haven't succeeded in conducting postnatal follow up for all mothers who gave birth.

I think Ato Zemede came here in June 2022. At that time, we were having only one or two new born babies who were treated and registered in our file. But when a review meeting was held after two or three months, it was found out that everybody has given postnatal care and assessed health condition of the new born for about ten mothers. Within one year approximately for 200 mothers that I did postnatal follow up and examination for their new born babies. We are seeing a great deal of improvement in the delivery of postnatal and new born care.

They give us a serious of practical tasks to judge our knowledge or ability. For example, they bring any child affected by an illness such as pneumonia or severe pneumonia and instruct us to give him/ her treatment.

After that we will explain to him how we have treated the child. If we make any mistake, he will inform us to rectify it. The system helps us to improve our skill and to find out the cause of illness and order the right medication.

Have you accepted the system in its entirety?

Yes we have. I have found it very useful. The support system helps to learn from our mistake. It shows us our strength and weakness in doing ICCM and new born treatment. Gash Zemed showed us how to use and follow the guide booklet correctly when we made a mistake. We don't forget the correction that we have got through this way.

How does ECHIS implementation help you with ICCM service delivery?

We haven't started implementation of ECHIS yet. But, they promised to start it soon. They said it is very helpful. ECHIS will help us keep the necessary information about the newborn.

We keep information about the newborn. But, was not given any training on this issue. So, I cannot say anything in response to your question.

How does the information contained about family planning, postnatal and antenatal care contribute to ICCM service delivery and for the treatment of PSBI?

It helps to access the new born information easily. The computer reminds us mothers who have appointment for postnatal visit and children who have appointment for vaccination. It shows red light who missed and default the service and yellow to remind us their appointment date.

Because of ECHIS we are certain about the delivery date and the risk factors associated with her pregnancy and birth of her child.

We register health information about the woman who gave birth and her baby. The computer asks presence or absence of danger sign for the new born. Based on the information filled for both, mother and new born, it reminds us the appointment date to visit them.

I think it is a modern system. It belongs the present period. I think the information stored in the mobile phone is credible because it is loaded by a specialist. We can make a mistake when we write down information on a paper. But information stored in a computer can be retrieved or manipulated very easily. I think it will let you know what type of treatment you need to order for a patient.

How is ECHIS helpful to make appointment with a patient and identify a defaulter?

We have a lot of work to do. The woman may not come to the health post at the appointed date and time. The mobile phone will let you know the passing of a period of time during which the woman failed to make an appearance at the health facility. Non-defaulters are shown by green or yellow color. But defaulters are shown by a red color. The same thing is true for children who have vaccination follow up.

ECHIS is very helpful unless your mobile phone apparatus is stolen. You can go to any place without sheets of paper and collect your data using your mobile phone. But, you cannot perform any other job because it takes time to fill and finalize the information. It is very slow. This is a weak point in using a mobile phone.

It is too difficult for HEWs to use a mobile phone to gather and manipulate data by using electronic equipment. As I have mentioned to you earlier, HEWs are required to complete a lot of task every day. Because of this reason a data clerk must be hired to do the job alone. Anyway, ECHIS is extremely important to collect reliable information and to detect clients who have appointment and defaulter easily. Finding these clients from hard paper is time taking and tiresome. In addition the quality of the data is more better when registered electronically because don't allow to do mistakes and not to miss any information.

If ICCM is module is loaded in to ECHIS it will save our energy and time from carrying unwanted or useless material to every household. It is beneficial to store all essential data pertaining to the child in our mobile phone apparatus. I think the phone will tell you the type of medication to be given to the child if ICCM chart integrate into the system. It makes easier to hold good quality data in the mobile phone. The data are stored in the mobile phone by specialists.

We are not required to write down anything. The mobile phone will tell us the type of treatment to be given to the child. It will inform to refer the child or the mother to a specialist.

What are the critical factors affecting the delivery of ICCM to clients?

The main challenge facing the HEWs is workload. The community lives in 16 villages. They are scattered in different directions. Only three HEWs are working for the health post. Some places are mountainous / hilly.

In some places we are required to cross a river. So, it is too difficult for the HEWs to carry out our duty. Sometimes we go to a campaign to achieve a specific goal. Therefore, we are forced to spend the whole day at one village. On that day it is not possible to give medical care to a sick child. In addition when the health post is closed, many people will be forced to go to health center. The number of HEWs and the population are not proportional. No boundaries for our responsibility and accountability. All these factor have either direct or indirect influence on ICCM/neonatal health care service.

When go to field/ campaign, we draw up working schedule. We use all available opportunities to do our job. We have expressed our complaints many times. But there is no reply. Our monthly salary is not commensurate to our responsibilities.

Unlike HEWs, many civil servants have a lot of free time during which they have no obligation or work. But from all other activities I feel happy when I give treatment and vaccine to children and offer postnatal care. I have been engaged in the job for about 14 years.

What kind of challenge have you faced to identify and treat neonatal infections in the community?

I cannot mention any problem related to skill now. But, you will face a problem if you don't have the guide booklet. During the training program the trainers (Gash Zemed) declared that only a person with an evil eye tries to manage without the guide booklet.

The guide guides every activity. You don't make any mistake. Not only us but also nurses are using it to manage and treat cases. Our guide is written in Amharic so easy to understand but not the guide in HC, written in English. It provides you with detailed information who to and not to treat.

I forget the case but I called to one of the health professional working in HC to help me solve the problem. She called and told me to refer to a certain (exact) page inside the booklet. Finally, the problem was resolved.

We are now memorizing information contained in the booklet because we work always using it.

There was no lack of inputs. There was shortage of gentamicin for some time. But, it was made available shortly.

Did the state of emergency and the conflict affect your activities?

No, it didn't. But there were people who have been forced to leave their home. Accordingly, we have been informed to provide service to displaced persons. We have recorded their name, number of pregnant women and children were coming to our catchment. They have created no problem. We managed to give the necessary service from the health post. There were women who delivered their child in the health center. There is no problem that we faced to provide ICCM and new born health service because of them.

Didn't they bring workload?

No, they didn't

How do you think the COVID-19 pandemic and/or COVID-19 response measures affect the uptake and/or delivery of ICCM services?

At first movement of people was restricted because of the pandemic. People stopped coming to the health post because they were scared of infection by COVID-19. Anyway, it did not bring significant impact. HEWs used to go to the community to identify people infected by the pandemic.

We were education the community to keep somebody who is suspected of infected away from other people to prevent the spread of COVID-19. However, we have not encountered anyone who was infected by coronavirus pandemic.

A few days ago went to the community to give vaccine. But, the community did not feel happy to get vaccinated. When a campaign is launched to fight against polio or measles, our regular activities will be negatively affected. We spend a lot of time to give vaccine to the community.

How did COVID-19 affect the community in terms of lack of income to come to health facility?

It did not have any impact. The community was always working despite the pandemic. We haven't seen anybody who was infected by the virus. There was no outbreak of disease in the area.

But, many people were frightened to go to Bahrdar or Addis Ababa. They were not afraid to gather in public places for buying and selling goods. But, we informed the community to enforce isolation of people exposed to COVID-19.

We used to measure their body temperature when they arrived here.

How did CIVID-19 affect ICCN/CBNC?

Children were brought here in spite of the pandemic. There was no interruption as far as child treatment is concerned.

Explain to us any changes you observed as the result of the implementation of the COVID-19 adaptive ICCM implementation strategies?

We were using sanitizer and a face mask to prevent infection by coronavirus pandemic. But, the community believed that we were infected by COVID-19 when they saw us wearing a face mask.

We have communicated information to the community to come to health facility to get medical care despite the pandemic. We gave serious attention to people who were repatriated from Arab countries. They were tested to ascertain the presence of COVID-19 in their body. The community used to report the arrival of people from the Arab world.

How did you interact with WDAs after the emergence of COVID-19?

We did not arrange any meeting with WDAs for about two months because of COVID-19. But later, they were told to practice physical distancing whenever we organized a meeting. We were communicating with WDAs by a telephone.

In addition, we had to go to their abode / the house where they live to carry out our duty. We were able to gather important information from WDAs. Many of the groups' members are using mobile phone.

A good number of people were given treatment against local bacterial infection. Children were treated against severe pneumonia. This is an important impact of the intervention.

How did you manage to treat local bacterial infections and other illness successfully?

We managed to successfully carry out our duty after following the review meeting organized by Gashe Zemede. Strict evaluation was made at zonal level. All pertinent entities were invited to participate in the review meeting.

They have promised to make inputs available for us without any interruption. We were told to pay serious attention to child treatment and vaccination.

We have been informed to bring the ICCM file to a review meeting with us. They use the ICCM file to review / evaluate every activity in detail. Everybody evaluates the performance of her fellow worker. Then we discuss how to improve our weakness. That was how managed to bring about a basic change.

What type of support did you obtain after the evaluation?

They were coming to us to oversee our activity or the task being carried out. They used to collect information about the number of women who delivered a child at a particular time. They were asking about the number of women who received antenatal and postnatal care. They looked at our file to make sure that the job was done. We have to produce an acceptable reason for failing to carry out our duty/ if any/.

Please explain to us the feasibility of this support/ IR for national scale-up?

I recommend the provision of onsite coaching and mentor ship to HEWs as good idea. It helps to upgrade our skill. So scaling up this following/supporting mechanism is easy. It is possible to do the mentor ship by health professional from HC/woreda health office. When HEWs are well skilled, Newborn / less than two months old/ and children under-five across the country will gain access to ICCM and newborn health care services effectively without compromising the quality.

We have encountered no problem in dealing with a number of child related illness. But, there is a need to hire additional workforce to make things proportional and minimize complaints or resentment. To be frank, we are doing very satisfying job. We feel very happy when we see people cured from illness. It is possible to scale up

How do you incorporate ICCM and child vaccination in your routine?

When we go for field work, we identify mothers that should obtain postnatal care. we give this case priority treatment. We must make sure that both the mother and her child are healthy. We must visit the woman on several occasions / again and again.

We will inform her to call us if she and her child face a problem. a woman can go out of her home seven days after she delivered her child. But, she must take holy water before she goes out of her home. We take amoxacillin, ORS, gentamicin, one page from the guide booklet(Voiding) and thermometer with us whenever we go to the community.

We have annual and quarterly action plan which is developed by our supervisors. ICCM and newborn heath care service vaccination are among the main focus of our action plan.

The plan also includes the number of children below two months old to be treated against local bacterial infection and pneumonia.

Additionally, the plan contains services to be offered to children between zero and five years old. Then, we make weekly, monthly and quarterly plan of action. After that the plan of action will be posted on the chart.

How do you make sure that you are carrying out your duty according to the plan of action?

We look at the weekly plan of action. We also submit a report on a weekly basis. After that we conduct self-evaluation to find out activities that are not done. We also find out type of illness that affected the child.

What kind of support do you obtain from health centers pertaining to child treatment?

Some of them give exclusive attention/ treatment to children. Accordingly, our discussion gives exclusive attention to children. We look into the booklet to find out how we treated the child, the type of illness affecting the child and the number of children who have obtained medical help. Then they find out whether or not the right type of drug or treatment is recommended for the patient. They let us know whether or not we have used the booklet / chart properly.

How often do they make a visit here?

They come frequently. They used to evaluate it every month. But, we have one expert who is helping us regularly. Some of them are coming every week if they think we are not carrying out our duty appropriately.

Did you find the support useful?

Yes.

Why?

If I do the job alone, I can make a mistake.

Do you have anything think is important to tell us that I have not asked you?

We get very small amount of salary. I have joined the civil service fourteen years ago. But, I get Birr 4,200.00 net per month. Every month I am contributing Birr 4,00.00 for the national defense force. So, now I get a net income of birr 3,800.00 per month. Our salary is not appropriately proportionate to our responsibilities.

Area Identification Amhara region

Name of facility HP

Date of discussion 20/6/22

Start time: 11:10AM

End time: 12: 25PM

Can you please describe the demand generation/SBCC activities you are doing on ICCM/ newborn health?

We used to bring members of the community together during religious service in the church to pass on information about the initiation of newborn health care service delivery in the health post.

We urged them to bring their children who are below five years of age to the health post to get treatment. We have started to ICCM service delivery several years ago and neonatal health service recently. Therefore, we move toward every village / household to let the community know about the service.

What are the strategies used to raise awareness?

We have applied a number of strategies to give information to the community. We transmit information to our target population using different structures such as Women Development Armies/ WDAs/. Besides there is one to five and one to thirty groups. The group leader is responsible to aware members of the group using each opportunity. For example they transmit information during meeting and other informal means of communication such as coffee ceremony, when they go to fetch water and the like.

Members of WDAs organize an occasion / meeting during which time we teach the participants / members of the community/ about ICCM/neonatal health care service.

How is the engagement of communities in the implementation of ICCM of common childhood illnesses/possible serious bacterial infection (PSBI)?

As far as the engagement of the community is concerned, everybody is showing a feeling of interest to acquire knowledge / information about the issue being discussed.

Accordingly, they bring their children to health facility to get treated. Additionally, members of the community exchange information pertaining to the treatment given to a new born child and children under-five.

When we go to the community to conduct door to door visit, we carry the necessary things for children such as medicine, thermometer, registration paper and the like and provide treatment and care accordingly.

The communities share their experience to each other about the services they have been getting for their sick baby from the health post. The mother of a sick baby share to other people about good things about the services/treatment the baby got from HEWs.

Please describe the challenges you faced when you were trying hard to aware/teach the community about ICCM and neonatal infection?

Not all members of the community are coming to our health post to get treatment for their child. Majority of them get medical care at the health center. Because the health center and post are found in the same compound.

We have not encountered any challenge worth mentioning. However, some community members were not punctual. They were not arriving at the arranged time. Besides, they would like us to bring the meeting to an end as soon as possible. They don't want the meeting to go on for an extended period of time. They say they have a lot of work to do at home.

A lot of people didn't believe that a new born child needs medical care. They thought that giving medication (injection) for the newborn baby is not appropriate. In addition at an earlier time they would not take their children to health facility to get medical care until the mother and the newborn baby were baptized and accepted into the Christian faith.

But now because of the taught that we and other responsible bodies made the community understood that newborn baby needs treatment when sick. The communities are willing to get treatment even for a one day neonate.

How do you describe the strength of the support you got from the PHC and the project as well as your efforts to address barriers to deliver ICCM/PSBI during COVID-19?

COVID-19 did not affect our activities. The community did not accept the emergence of coronavirus pandemic as true. However, the community was showing signs of fear of being taken to place of isolation to prevent the spread of the disease.

Therefore, they were not willing to come to the health post immediately after they and their children became sick. Besides the kebele has a very large area. So, it was somewhat difficult to make the community gain access to the service offered by the health post. To address this gap Gash Zemed told us to built strong communication with WDAs. Accordingly we were communicating them about mothers who gave birth, sick child, pregnant mothers and other health related issues that requires our services and have given treatment and care for them.

Gashe Zemed went to each church and communicates a message at each village about neonatal services. Besides awareness creation, Gash Zemed and we gave treatment for sick neonate and child in their village.

Not all WDAs were willing to give time and resources to accomplish an activity.

Why is it difficult for members of the WDAs to attend a meeting?

They complain that they are given no financial incentive. They say it is better to us to look after our children than spending time here with you. They say they have grown tired of hearing the same kind of message again and again.

What kind of action did JSI take to strengthen WDAs?

They advised us to try hard to persuade them to actively involve in the activity. There are different groups within the WDGs which are referred to as one-to- five and one-to- thirsty. These groups have not received any special support but Gash Zemed advised us to build our relation sihp/communication with them. These groups of people can easily access information about mothers who gave birth and their newborn and then gave the information to us.

Now a day I heard a group called village health leaders but formation of this group has not been started yet. And I didn't know why the health center not started organizing this group.

How do you describe the strength of the support you got from the PHC and the project to address barriers to deliver iCCM/PSBI during COVID-19?

Gash Zemed used to come here and check names of service seekers that are recorded in our official register. He offers us advice and comment checking the treatment that we gave to a baby less than 2 month and under five children.

Mostly he strongly has advised us not to give any kind of treatment to a child without using the guide/ booklet. He showed us how to calculate the medicine according to age of the baby.

Besides checking the treatment that we gave, they provide us with a particular case scenario including the sign and symptoms of the disease involving possibilities and they were observing when we identified and treated the case of the given child. Gash Zemed come here once or twice. We got this type of support only from him.

How often do people from the health center come here to give you support?

Our health post is found in the same compound with the health center so they haven't given such type of support for us.

Please tell me clearly what he did when he came here?

He has investigated the booklet carefully. He will make sure that all information is properly handled. He confirms or establishes whether or not we diagnose or identify an illness in a child correctly. He checks whether or not we order use of the right medication for each child.

As you mentioned you have got support from JSI. Did do you get the support system helpful?

Our performance has shown a great deal of improvement after JSI stated to give us active help and encouragement. I can make a lot of mistakes if I do my job in a particular way by tradition without using the guideline/ booklet. But, the support I get from JSI provide me with the means to remove errors. We are required to be as precise as possible when we give medical care to patients because we are dealing with human life.

Please describe the advantage of the support you obtained according to their importance over the previous off site training?

It will help us reduce our mistakes to a minimum. It is also having value or benefit to go to the community frequently to find sick child or their particular problem as a subject of medical attention. Visiting the community door to door helps not to miss sick neonate and also gives us to implement the treatment soon. Providing the treatment immediately after we get the support helps us to provide the treatment in the right way.

Gash Zemed asked us the number of neonates that we visited. To have an answer for his question we visited each house and give appropriate care and treatment for neonates and under five children. This opportunity helps not to miss postnatal mother and to provide other health related services.

Can you mention about any other advantage of the support you have obtained from JSI/Gashe Zemed/ comparing with the previous type of training that you have taken?

They help us not to make the same mistake again and again. They advise not to ignore the guideline / booklet when we carry out our task. We might do simply without using the guide booklet thinking that we are familiar in identification and treatment of cases.

Are HEWs accepted the support given by JSI?

Yes, we are happy and pleased. We accepted it. The mentorship helps us fill a deficit of our capacity required to carry out our task efficiently.

Did you face any issue causing difficulties or unwanted extra effort because of the support?

There was none.

Was anyone from the health center / either ICCM focal person or any other health worker/ coming here to give you support?

No they didn't come alone. But they came together with Gash Zemed. They watched/ observed what Gash Zemed commented and showed over a task done by us. Gash Zemed orient the health worker to follow and assist us accomplish our task.

How did people from the health center assist you?

Health professionals in the health center don’t have many years experience. They are fresh so they may think that the HEWs are accustomed to carry out daily routine based on their experience. We (HEWs) have many years experience. We have worked as HEWs for many years. But, the health worker who is assigned to assist us may not have adequate exposure to events. Because of this they didn’t give support to us. And yet we haven’t asked them to support us.

What do you think ought to be done to deliver ICCM service and neonatal care differently?

I have no idea. But what I am certain is that we always strive to provide the community with appropriate services. I think no activity should have been done differently.

We are rigorous in ensuring that all rules should be obeyed to offer essential services for all children who are sick. But we are informed to register and keep the name of all neonates in writing and identify an illness if not to say no infection. However, it is too difficult for us to register all neonates who are not sick and we didn’t understand its benefit. We don’t have adequate time to carry out this task.

We need to be given specific activities to reach as many children as possible. We have too much work to do. The work has to be shared among HEWs. Each of us has to devote our time exclusively to perform particular job. We couldn’t focus on ICCM/neonatal health problem because many activities we have been assigned to be done in specific time limit. Providing ICCM/neonatal health service together with the health extension package doesn’t make to accomplish successful outcome and not possible to address all under five children. For example health professional in health center has specific duties and responsibilities.

How are health workers assigned to do a job at the health center?

Everyone is assigned to do a particular job. For example, a midwife is trained exclusively to help deliver babies and offer support and advice to pregnant women. But, we are assigned to offer every kind of service. This is difficult to bear. Likewise, the quality of service provided to the community will be compromised.

How ECHIS implementation helps you with ICCM/neonatal service delivery?

We haven’t started implementing ECHIS yet. We are still entering the data into it.

When did you start to record ECHIS in writing?

It was two or three years ago. The area covered by our service is large. Usually, they don’t assign the right person with the required skill to do the job. For example, I was in charge of doing the entire job for more than a year alone. I was not having any help or support from anybody. The remaining two HEWs were not available due to maternal leave and schooling. Therefore, it was extremely difficult for one person to finish entering the data into database. Additionally, the system requires training or the process of learning skill.

We need a lot of time to enter the information into database. The information network linking computers is not acting rapidly unlike writing in hands. Mostly we enter 3-4 households per day. It is not possible to enter more than ten household (maximum number) into database. Besides we are assigned to take part in different vaccination campaign. We completed entering the data into data base this week.

You finished entering the data. How ECHIS implementation helps you with ICCM/neonatal service delivery?

We can find information about each child in the database easily. We can find the child under the name of the household that the child belongs to.

Which one of the systems/ the previous or the current one/ did you find appropriate and easy to retrieve data?

It is not difficult to carry our tablet / personal computer/ in which the information is stored. Information put into a database cannot be spoiled easily. We cannot erase or remove the information from a computer file or disk easily. I hope we will successfully master the technology within or after a short time.

In my view it is easy for us to get back information from those recorded in writing. The number of households entered into the data base is too much /bulky/ so we couldn’t get the data of each individual quickly unlike to that of hand written file.

Why did you prefer to record your information in writing to putting it into a database?

It is because we are accustomed to do things in that way. We are not familiar with the tablet. We haven’t practiced providing services using the tablet.

Though you are not familiar do you think ECHIS is beneficial to show you a defaulter?

Yes it is. But, we haven’t started using it. Last time we made an attempt to apply the system. It has produced red, green and blue colors when we were dealing with antenatal care. It showed us an arrangement of services based on the appointment date. In such a way it indicates us something has to be done before something else can happen.

What are the colors showing?

For example red color for pregnant mother indicates that the mother is near to term or shows that the pregnant women didn’t come here.

What do you think needs to be done to strengthen ICCM service delivery?

We must be oriented with a new situation to make proper use of the tablet to store our data. We don’t have the skills required to use electronic data processor properly. But, our supervisors have assured us that training will certainly be given to us.

Describe the main issues faced by the health system to identify and treat neonatal infections in the community.

When a child is brought here, the first thing we are expected to do is to find out the weight of the child. But, we do not have appropriate weighing machine to find out the weight of children who are less than two months old.

Which one of the signs which indicate ill-health in a new born child have you found difficult to identify?

We take neonate and children's weight to calculate the appropriate medication. But we don't have a weight scale which is comfortable for new born baby. We prepared weight scale made from plastic basin. Baby's mother doesn't feel good when we place the bay on the plastic weight scale.

It is easy to identify an illness in a child but giving injection for new born baby requires confidence. Sometimes we frustrate to give injection to them.

Previously, we used to give Gentamicin which didn't require preparation/dilution. We were not required to dilute but now it needs to be diluted. I don't know how to dilute 40 and 80 mg Gentamycin. We call health worker from HC to show us how to prepare/dilute. Preparation of such types of Gentamycin is very confusing for all of us(three HEWs in the health post).

Did you face any other problem to diagnose an illness in child affected by an illness and to give him / her medical care?

Nothing

Did you face shortage of inputs to provide service to a new born child and children under-five?

There was scarcity of OTP. Children who are wasted / very thin and whose MaUwac is below 11.5 Kg. These children need to get nutritional feeding/plum peanut for three months. But because of shortage of Plum peanut we didn't provide for them.

What other Shortage of supplies and commodities did you face?

No. We take delivery of each item when deemed necessary. We request using RR timely from health center. 80 milligrams and 40 milligram of Gentamicin is included in the requisition form.

Do you think the community has a positive attitude toward the service?

The community is willing to use the service.

How did the state of emergency declared following the conflict in the northern parts of the country affect your activities?

There were a lot of people who have been forced to leave their home because of the war. Therefore, it was difficult to meet their requirements as far as the delivery of medical care is concerned such as preparation of

latrine. There was also a lack of food leading to sickness. For example breast feeding mother came because her child wasted due to lack of food. As I told you there was no plum peanut so we couldn't give plum peanut for the child.

Was the displacement of people causes barrier for ICCM/neonatal health service delivery?

Not much but shortage of plum peanut occurred because of the displacement. Number of plum peanut users increased when the population increased due to it.

How do you think the COVID-19 pandemic and response measures affect the uptake and delivery of ICCM services?

COVID-19 did not affect our activities. The community did not want to hear anything about COVID-19. They believed that the whole idea about COVID-19 was an invention of the politicians.

Members of the community showed no signs of fear about coronavirus pandemic. But at first many people were afraid of being infected by COVID-19.

For example, if a person was showing an act of coughing, he would not dare come to the health post because he / she was afraid of being put in a place of isolation if they were found to have been infected by COVID-19.

The meeting with WDAs not interrupted because of COVID.

How did you provide ICCM services to members of the community when they stopped coming to the health post?

We told them no one would inspect their condition or health against their will.

We go to every household and provide ICCM, neonatal, PNC, ANC and other services. During which the villagers were advised to come here when they are affected by ill health.

What are the high-level benefits that are attributable to this support/IR?

The support helped us fill our capacity gap. One day a child to whom I gave medication previously has come to the health post. Then he/ the supporter/ carried out official inquiry to the guideline /booklet and found out that the recommended drug was not proportional to the weight of the child. After that the child was ordered to use medication that was twice as small as the previous one. This was how their support provided us with the means to enhance our capacity.

Can you mention any other benefit of the support?

It is useful to increase our working effectiveness. We are able treat sick children because we become very qualified through the support learning from our mistakes. It helps to deliver appropriate treatment for sick children as per

our plan. The more we communicate with the community house to house, the greater the number of children that gain access to our service.

Please explain to us the feasibility of this support/ IR for national scale-up?

We found the assistance very useful. I appreciate and providing treatment to new born and under five children is easy. I suggest designing a support system for achieving similar objective as good idea. But, I don't think it is advisable to keep the name of all children in writing as. This might work for the Kebele where small number of people is living. We may expend or consume our registration book until none is left if we register all. In my view, it is reasonable to keep a record of children affected by an illness. This condition will help us save time and reduce physical and mental exhaustion. Assigned activities for HEWs are not countable. We are tired some in registering surplus information.

How are the activities/ efforts embedded in the health post routines/work streams?

Usually we go to see members of the community and spend time to watch over their well-being. We identify the number of women who delivered a baby.

In fact, we can find information from the health center. They let us know about the number of babies born by a tablet. We can share information to each other by a tablet.

What other information can you access through ECHIs?

They shared and let us know the type of family planning that the mother has used, number of follow up pregnant mother attended and which follow up she left, and others.

You have mentioned that the health center will share you such information so what kind of contribution does it have for ICCM delivery service?

It will help us visit the woman and provide her with essential support such as postnatal care and also to register and assess the health condition of the neonate.

How did you incorporate ICCM and neonatal care within your usual activity?

We take a lot of things with us when we go to the community. We take contraceptives, ICCM inputs such as ORS, amoxicillin, and Zinc. We also take a thermometer to measure body temperature.

What implementation strategies are incorporated in your annual work plan regarding ICCM/neonatal health care?

We also combine ICCM and neonatal care within our weekly, monthly and quarterly plan of action to give medical help to children who became sick of local bacterial infection, sever bacterial infection, diarrhea, severe pneumonia, and others.

We also gather information from WDAs about the number of women who delivered a child within a specific timeframe / period of time.

When there is significant difference between the plan and accomplishment, we examine the reason. Accordingly, we try hard to reach as many mothers as possible to achieve our plan.

What is the current problem you are facing to prepare Gentamicin?

We learned how to prepare or dilute gentamicin. Gashe Zemed and other health professionals from the health center have come here to show us how to dilute and calculate Gentamicin. But, the message can be forgotten easily. The booklet does not state clearly how we should dilute gentamicin using distilled water. The method of dilution and calculation should be prepared in a chart and posted to use easily looking on it.

What other challenges did you face to diagnose a new born child who is less than two months old?

There is no problem if we have confidence in our abilities to diagnose. Sometimes counting the breaths in neonate is challenging. They have very fast breathing. Because of this we are mistaken in classifying the disease in new born babies.

Sometimes we may assume that the child is having very little body fat (very thin). And we are inclined or tending to give an injection.

Do you have anything you think is important to tell us that we have not asked you?

They kept quiet.

End line Evaluation of the PSBI implementation research; Full Transcription for an In-Depth Interview with iCCM Focal Person

Questionnaire ID	09
Area Identification	Dembecha
Name of Woreda/Zone/Region	Dembecha WoHO/West Gojjam Zone/Amhara Region
Name of facility	Health Center
Name of moderator	
Name of a note taker	
Date of Interview	16/06/2022
Participant #	01
Audio File #	iCCM Focal_16.6.2022
Start time:	09:00 AM
End time:	10:31 AM
Transcriber/Translator	
Duration of IDI:	1 hr, 32 minutes

I. Fidelity

1. Can you please describe the demand generation/SBCC activities you are doing on iCCM/ newborn health? What are the strategies used to raise awareness? If none, please explain why.

Interviewer: Can you please describe the demand generation/SBCC activities you are doing on iCCM/ newborn health? What are the strategies used to raise awareness? If none, please explain why.

Respondent: I thank you so much for having me for the interview. First what we did was to organize training program for Health Extension Workers/ HEWs/ and ICCM focal person to boost their capacity, and your organization has also trained additional health care workers other than the iCCM/CBNC focal person. Why we did this is because I am not able to oversee the usual activity of HEWs because I am completely absorbed in performing my usual activity. In other words, I have an excessive workload. Therefore, training of an additional one person is to give focused assistance to HEWs using a checklist while they carry out the tasks done or occurring every day. Accordingly, the expert is now focusing more on the HEWs, and supporting and following the direct iCCM implementation by HEWs. Unless it is for the occasional interruption due to some campaign activities, this trained person is supporting the HEWs based on a checklist; other than the one health post, all the rest out of five HPs

we have been supported using a checklist. Previously however, most of the people did not know whether or not CBNC/iCCM was offered by the health posts. As a result, a lot of people used to come to the health center to get the service. We also used to ask them about their reason for coming to the health center, and most mentioned the lack of awareness about the service existing at the HP. So what we did afterward is communicate with the woreda and the health center, and an official letter to be issued especially to the woreda administrators. Then, we have provided awareness indicating about the fulfillment of materials and supplies, and readiness having trained health care providers at the HP, and hence the provision of the CBNC/iCCM service at the HP. We told them that we have done what is necessary to carry out our task. We made effective use of the available opportunities including religious places inside a church to pass on information to the community. We even did this through a campaign at the churches and other places together with the HEWs and also dividing the task among other health care workers to make the community familiar with the CBNC and iCCM service provided by the health posts. Afterward, members of the community stopped coming to the health center and avoiding the unnecessary travel and physical exhaustion after travelling long distance to the health center to get the service especially for the CBNC service, but they were still coming for the iCCM service since it was a bit difficult to manage at the HPs.

Interviewer. Why do you say it is difficult for the health posts to deliver ICCM service?

Respondent: Because supplies were short at the health posts for the iCCM service. Usually, there is a scarcity of zinc and amoxicillin. But our need for amoxicillin has been partially satisfied. Therefore, the community is / was coming to the health center because of scarcity of essential supplies such as amoxicillin and zinc at the health post. The iCCM service of course is also provided by the HP, but it was like the CBNC service, as a result, we have been more focused to the CBNC service than the iCCM.

Interviewer: What else have you done to increase the level of awareness of the community on iCCM service?

Respondent: HEWs have performed a lot of activities worth stating. There were conferences organized at the kebele level where the different leaders are available, and it was facilitated for the HEWs to disseminate information to the people who take part in the meeting. We used to support HEWs organize the conference. There are opportunities where we provided education for clients here coming to the health center that we provided every morning offering advice to use the services at the HPs which are close and easily available for the farmers without the need to come all the way here. To our delight, now everything is turning out well or having the intended result and most community members are using the services [iCCM service] at the HPs unless it is for some challenges we encounter in stock out of supplies and during campaigns that they come to the HC. Otherwise, it is in a good state and the community is gaining better knowledge.

Interviewer: Have you ever used a banner to advertise or publicly announce the availability of the iCCM service?

Respondent: We have not used a banner so far. As you know, we have severe budget constraints, and hence it is difficult to prepare a banner and advertise our service. To our dismay and to the dismay of others, we have no money to buy even printing paper. But, we exert the utmost effort to utilize every opportunity that arises.

a. How is the engagement of communities/WDA networks in the implementation of integrated community-based case management (iCCM) of common childhood illnesses/possible serious bacterial infection (PSBI)?

Interviewer. How is the engagement of the community and the women development agents' networks in the implementation of the iCCM service? What is the community level participation?

Respondent: it is good. This is because it does not incur them a lot of costs if they use the service at the HP, but if they come to the health center there is a wastage of time and cost as well. But, going to the health post involves very little time and effort. Besides, they receive the service for free. Consequently, the community is now feeling happy and encouraging us to continue because if this is at the health center, they are charged 7-8 birr to be issued a card but every kind of service at the HP is given free of charge. Unless the community sometimes being completely absorbed in the situation of the country, they have good engagement.

Interviewer: Are they willing to bring their children to get treatment?

Respondent: The community is willing to bring their children to get treatment since it also saves them from the transport cost; to your surprise, there is a kebele which they charge them as much as twenty birr for a single trip to come here which is normally the price charged to go to Dembecha health center [farthest]. This is an exorbitant price or unreasonably high. So since it has saved them from such things, they are happy with the service availability at the HP.

Interviewer: What about the contribution of the WDAs and how are they participating in your awareness creation activities?

Respondent: Honestly speaking there are no Women's Development Groups/ WDGs/ in our neighborhood. They aren't functional at all. Therefore, we have put a different structure in place. The recently formed structure is called village health leaders which are established in the anticipation of a specific entity that can focus and support the health-related activities. it is with the intention that they can somehow replace or compensate for the WDAs which we can say are no more existed.

Interviewer: what are these village health leaders and why did you establish them?

Respondent: It is because the officials at the woreda level did not pay adequate attention to the community level health related activities. It is then the follow up of what to do next. Regarding the village health leaders, we have assumed that we can find at least one person per one village who will be willing to join as village health leader, then we train her. The number might depend on the different village

[Gote] population size; if it is large population we select more village health leaders. What the criteria says is that three people will be selected from one village [Gote], then these three people will be trained. It also requires that these people must be able to write, read and accepted by the community. After we train prospective members of the group, they will be assigned to educate the community about neonatal health care, nutrition, Maternal and Child Health/MCH/ together with HEWs and render the HEWs with support. As we know, the HEWs activities are becoming broad in scope and becoming difficult to manage, which is why we are heading towards establishing this new system. But for the WDAs roles, you can say they are already disintegrated.

Interviewer: Does this mean they are going to replace the WDAs?

Respondent: they will replace WDGs to some degree but not completely. Village health leaders are rather additional to WDGs. They are not going to replace them since the WDAs are selected or the interest of the national level. The VHLs will play a role at least facilitating and balancing the health sector activities since the WDAs are not there.

Interviewer: Have you seen any change already from using the village health leaders?

Respondent: First, we were trained at the woreda level including the HEWs and our health care providers [at the HC], then we recruited the village health leaders and planning to provide them a training; we have already chosen members of the groups from among the community. Likewise, our next job is to give them training at a woreda level and allocate a task to everybody. But, because of the current situation in the country, and also related with the campaigns activity such as Polio, Trachoma and currently the COVID vaccine campaign made the progress difficult. I hope we will achieve a desired result if they are given training and job assigned to them without delay.

Interviewer: What is the work or planning involved in making the training ready? Please describe your state of readiness to make the training real?

Respondent: There is a training manual that was prepared at a woreda level which we are waiting for that. We expect them to organize the training program as soon as possible. Three people have been selected from individual kebeles to offer the training after which we made them known by the woreda heath office and the health center. We are expecting the woreda health office to give us inputs to offer the training to the village health leaders. What is expected of us is to choose prospective village health leaders from among several villages and make them known by the woreda health office.

Interviewer: Are their duties and responsibilities clearly defined? Is there any job description for the would-be village health leaders?

Respondent: There are two kinds of manuals or books giving instructions. The manual states that every village health leader will submit a report every week about the number of pregnant women, newborn child health, vaccine administration through campaigns, etc. Every village health leader and the HEW will

come together, write and submit the report to the woreda health office and the health center once each week.

Interviewer: what is the distinction between the roles and responsibilities of WDAs and those of village health leaders?

Respondent: There are different types of WDAs that are involved in supporting the agricultural sector, health sector, education sector, etc.

Interviewer: Ok if that is so, with the health development agents that is the HDAs. What is their [village health leaders] difference from the Health Development Agents/ HDAs/?

Respondent: it is not that significant.

Probe: What are some of the biggest challenges with SBCC activities for newborn care?

Interviewer: Please describe major challenges you have encountered to communicate information to the community and boost their awareness? Do you have any uncompleted activities owing to different reasons?

Respondent: a number of activities remain uncompleted and lag behind when we take part in different campaign, for instance, there were campaigns for polio, trachoma, and now there is also COVID. As a result, the health posts will be completely closed because of these campaigns. At that point, clients using the service will go back home without getting medical care or advise. After that the community will provide the absence of HEWs from duty as a reason not to go to the health post again. Every activity having a brief duration will take our attention away by appearing more important. The other challenge has to do with the lack of supplies. On one occasion it was difficult to find gentamicin in all pharmacies, vendors selling and dispensing medicine. There was zinc shortage for about three or four months. In these cases, our community does not trust you unless you work and show them the output, in order to do that however, we need adequate budget.

Interviewer: How did budget constraints prevent you to make your service known by the community? Since my question is related to the SBCC activities.

Respondent: for instance, we raised the use of banners earlier, so budget to prepare the banners to aware the public and give information to the community. We also need budget to incentivize the WDAs to motivate them to take a course of action or continue their work. At an earlier period, we used to pay incentives not only to members of WDAs but also kebele administrators. But it is no longer the case. Nobody is now willing to assist you without any incentive or something that motivates them to do a job.

Interviewer: I was going to ask you this earlier, who was paying the WDAs these incentives earlier?

Respondent: it is the woreda health office used to pay them in the form of periderm. In past members of WDAs were summoned three time a month for different reasons, now it is even available for the health

care providers; it has already stopped. Regarding this, majority of the kebele leadership are still engaging and they tell us they would rather prefer that their perdiem be deducted and given to the WDAs and incentivized since they have been a big part of their activities. However, the policy does not allow that; the law developed at a federal level says that WDAs should provide service to the community for free, on a voluntary basis or without financial reward. However, last time the Carter Foundation ordered us to provide WDGs with financial reward in return for what they have done. But, the regional government does not allow us to effect the payment. This condition is creating unfavorable results. I think the government should consider revising the policy that forbids paying financial reward to WDAs. We are experiencing a rising inflation. Members of WDGs deserve incentive because they are unable to meet the rising cost of living.

2. Could How do you describe the strength of the support you got from the PHC and the project as well as your efforts to address barriers to delivering iCCM/PSBI during COVID-19?
   Probe: What was changed?

   Probe: Was the support you got from the project and PHC helpful? What could have been done differently?

Interviewer: how strong and effective was the assistance given to the health post by the health center and the organization that has supported the project? Especially provided in COVID era and considering the pandemic.

Respondent: The health posts have made effective use of the support given by the health center and that of the project which has provided support in different forms—I think this has to continue in this manner. What makes you amazed is that previously we were using a guideline that was out-of-date or no longer current. But recently we were given a revised version of the guideline in a soft copy to deliver ICCM service.

Interviewer: please describe how has the project been providing you support?

Respondent: it has mostly been supporting us with observing practical aspects of our activity. For instance, I might be engaged to fully produce a complete outcome, and it has been supporting on what I am doing, and checks if I am making the correct diagnosis, treatment, etc. This is how it has been supporting me that is concerned with the technical aspects of the activity and providing recommendations. It is also a big deal from the project to train health care providers who can support the HPs. So in general the technical and training aspect of the support from the project was good. We must find a way of expressing our gratitude to the organization for all it did.

Interviewer: what about the support from the HC to the HP, what was it like?

Respondent: regarding the support from the HC to the HP, I don’t think it resulted in having a satisfactory outcome for the reason that we are frequently occupied in other activity. As I have mentioned to you earlier, after the iCCM trained health care provider returned we developed the checklist and commenced

support at the community and Gote level by integrating him with the Kebele assigned supporters, but as we always start the implementation, we were constantly being blocked due to other competing priorities. Therefore, intervention by other activities has made our progress difficult. However, the HEWs have been well exposed to the iCCM/CBNC training and acquired detailed knowledge. For this reason, if you ask a HEW and compare them with health care providers at the HC level, you might not find a significant difference. This is because the HEWs have taken part in CBNC/iCCM training more often than anybody else. JSI gives extensive training to HEWS about CBNC/iCCM, and there were also other partners in the area who were active in providing HEWs with different supports. However, we cannot claim with absolute certainty about the involvement of the health center to build the capacity of HEWs.

Interviewer: Do you think your participation in a campaign is the only reason that has prevented from capacitating HEWs?

Respondent: We have no other problem worth mentioning. It is advisable for us all to go to the health post and gather information about the problem they are facing and how they are progressing. I don't think there are others reasons for the health workers here at the HC to go and support the HEWs other than the over-engagement in the different campaigns.

Interviewer: Do they have transportation problem to go to the health posts and comeback here?

Respondent: they may be a problem of transportation. But it just one of the kebele which is far from here, the rest are relatively reachable but still availing the transportation access could serve as a facilitating factor to make the support at ease; this is because there is an apparent difference who someone is told to go on his own and when it is availed for you. There are such gaps but when we consider the professional ethical issues, I think it is related only with the extra engagement in the campaigns which they still could have supported.

Interviewer: What can we say about the frequency of support you made to the HPs, and How often did you go to the health posts within the last twelve months to give them support they required?

Respondent: in the past many experts drawn from different units used to go to the health posts to give them assistance. But, within the current budget year, we did not give them any kind of assistance. What we did was just to assign an expert to provide them with the support that has relevance to CBNC. Earlier the focal persons for Mother and Child Care/ MCH/, under-five children, the pharmacy, Outpatient Department / OPD/ and the health extension program coordinator used to impart to convey information or knowledge to the health posts. This way of proceeding to achieve the desired goal has already been discontinued. But, experts are still going to the health post alone to give them support. We no longer go for integrated supportive supervision. Owing to the above facts and circumstances, at the present time it is difficult to know how the health posts are operating.

Interviewer: is there a support at individual level? It is because you mentioned there is no integrated support?

Respondent: It was that person who used to support them. He was immediately trained, and also has been jointly going with you for some time. So he has been supporting and communicating some of the HPs using a checklist.

Interviewer: is iCCM service included in the support he was making?

Respondent: No, he did not give any ICCM support to the health posts because he has not attended any training. As a result, he was focusing only on CBNC support.

Interviewer: What was also your problem not to go to support the health posts?

Respondent: As you told you, there is no one else here. I am in charge of two units here, and if I go to there, the activities will be affected during my absence here. I am handling CBNC, ICCM and vaccination. It would have been good if two persons are assigned to manage the two sections.

Interviewer: Did you make any plan of action to deal with the problem you have mentioned earlier, in order to resolve these challenges?

Respondent: there was nothing I can mention to you. There are the assigned HP supporters which continued the support, but there was no major decision that we passed on this problem.

Interviewer: What things do you think should have been done differently to keep on providing the support to the health posts?

Respondent: what it says as a standard to equip a given health post is that the health post should at least be visited once in every quarter—it is expected from the HC, but we didn't do that. Similarly, it is also expected that the woreda visits the HC once in every quarter, but that is also not the case. So when this is not done, it means it blocks everything. There is this thing which seems a curse that we always engage when there is support and stops when the support stops too; this has been a trend but what would have been great to do was that if we were able to make the support using a checklist. Ofcsourse we raise the issue in the different meetings about the lack of support, but overall our supports tends to be weak.

Interviewer: the reason for this is still the one you told me before, or is there any other?

Respondent: the reason for this is we lack a facilitator to give attention. As an institute, it needs someone who can coordinate and leads it properly. Besides, the chain of communication for the woreda support has broken down. Formerly, every quarter. people from the woreda health office used to make a visit to the health center to assist us. They even checked the cleanliness of the health posts. When this stops, our leaders also stopped. We always raise and discuss on why we don't support the health posts using a checklist. If you see now the HPs status—which you may have seen them already, they don't look like a health post. We were expected to support them but we didn't do so, this is because the whole chain of command from the woreda to the lower level has been disrupted.

Interviewer: can you describe the reason behind the disruption in the chain of command from the woreda to the lower level?

Respondent: I have no idea about it. Previously, we were planning to do a lot of work. But, we accomplished nothing to achieve a goal or desired state.

Interviewer: How much did the war between opposing forces in the country and the emergence of COVID-19 affect your plan? Do you think these may have contributed for the loose engagement of the leadership?

Respondent: I don't think the conflict and coronavirus pandemic were and are having any impact as far as provision of any support to the health posts is concerned. They might have affected other activities. The war and COVID-19 cannot serve as acceptable reason or excuse for the health center not to support the health posts. I Believe lack of commitment of the leadership is to blame for not giving assistance to the health posts. But, we cannot deny the impact of coronavirus pandemic on a number of activities.

Interviewer: can you mention something about the support given to the health posts by other entities to identify and treat on newborn health care?

Respondent: Currently, only JSI gives the entire support to run the program. In addition, the woreda health office has given assistance to the health posts that is too small to be important. They provided information to the members of the leadership about the provision of CBNC/iCCM service by the health post. Usually JSI is organizing a number of training sessions for HEWs. It provides them with the means or technical support to efficiently carry out their routine. The woreda health office makes the awareness especially for the core leaders, they are familiarizing the leaders about the health care services given by the health posts despite the fact that WDAs are currently not functional. Other than these two bodies, I don't know about any other entity that supports the program.

Interviewer: do you have a system of support in which you evaluate the performance and the progress, is there any support you implemented in this manner for the HEWs?

Respondent: Yes, we have. By the way, we evaluate our performances every month. We evaluate the HPs based on the number of cases it treated for diarrhea or pneumonia under CBNC or iCCM, and other types of cases per health post., then we evaluate the reason for the low performance. After that we take a new direction to improve our working effectiveness. We enforce or compel our employees to organizational rules to improve their competence.

Interviewer: do you take any action afterward? If you do, what type of action?

Respondent: the action is that we put out future direction. But, some problems may arise and will cause a serious delay in our action or progress, for instance, if a HP didn't treat a diarrheal disease within two months, it is not considered as existed. So we set future directions to follow and monitor to avoid such problems occurring in the HPs. However, we are a bit weak in conducting the proper follow up. By the way there are also other partners providing support, including JSI managing to give training to

health workers and HEWs to boost their capacity to deal with any type of inconsistency and providing awareness. This is how we evaluating ourselves.

3. Do you think the support system is helpful (mentorship and on-site coaching methods, supervision, technical support, PHCU level PRCMM, etc.) to enhance your skills [that is HEWs' skills] over the traditional off-site training and woreda level review meetings?

Interviewer: How do you evaluate or describe the benefits gained from the trainings / both conventional and unconventional/ to increase the capacity of health workers, and also including the support you provided in improving the skill related to the iCCM service delivery? How did it

Respondent: their [the HEWs] skill has improved a lot and as I told you, it is also inseparable from the health care workers working here at the HC since they update them in different ways. In relation this, to your surprise the guideline you see here is a recent iCCM guide but no one is trained on it here even if they are trained on CBNC; no one here is trained on iCCM at the HC level. And hence, JSI has tried to familiarize us with the new guide and provided us with a momentary orientation on the new guideline. Then we provided the HEWs a follow-up orientation on the specific changes of the new guideline compared with the old; whether it is the treatment or the follow-up of cases which differ now. I am happy with the JSI providing the HEWs with different awareness at different times; the HEWs are provided with different trainings taken as far as to Debremarkos by JSI. You can see that they have better knowledge than we have as a result.

Interviewer: Tell me about the skills that have been improved as a result of this support by JSI [training]?

Respondent: one must have confidence in one's ability to do a job, this is the first. Health workers will be able to make a plan to implement CBNC or ICCM only when they have self-assurance in their ability to succeed. Accordingly, the training has provided them with the means to have the confidence to implement the different activities from the meeting and other exposures by JSI. The second issue has to do with the issue of completeness which enables the data to have quality with all the required information filled—I think it has improved them in this respect. The reason for this is also from the regular follow-up and monitoring which makes a difference from the regular identification of gaps and recommendations provided. I think it has also increased their knowledge in this manner. Then it has also contributed to providing the required skill for making the proper care and administering drugs by the HEWs.

Interviewer: Which type of support do you think has improved performance, and discharge their duty efficiently?

Respondent: it is the one JSI has done. One of these is the training and also the support it provided them by coming here onsite. Especially the one which it provides them here has also a lot of components.

Interviewer: Which one is more significant?

Respondent: it is the one it provides them here. This is because, when it is the training they are just using words but when they come here, it is practical and shows them while they are doing it themselves. This is

from the information I have been told that I am telling you. It is also the fact that people tend to understand more when you show them practically.

Probe: for acceptability of the strategies **(for HEWs only)**. How could the support system be improved?

Interviewer: how much do you think this method of support has gained acceptance by the HEWs?

Respondent: We all have different attitudes or points of view about a particular issue. For example, I may say also that you exhausted me from the questions you are asking me but if we are committed to our profession, we will be feeling pleasure. As a result, the majority of health workers are showing pleasure, if we feel the issue from our heart, we will all be happy. As a result, most of them are happy, and we get great enjoyment and pleasure when a child affected by illness is completely cured. So most of them are happy with the practical support they are getting.

Interviewer: Do you think the support should be given in a different way? Do you have anything to recommend on how the support should have been provided differently, that is general on the different supports provided to improve skill?

Respondent: when I start from the JSI support, it is good and should continue providing training to health workers to make them feel more knowledgeable. People always some refreshment after sometime so they should continue refreshing the staff. Especially the support through onsite presence at the HPs should continue which has importance in rectifying imperfections. Still on JSI, I say it has to do more to improve the capacity of HEWs especially in providing updates; the health workers have already been provided with most recent information about CBNC and ICCM but if there are still updates to be provided and attention be granted for training. What I am saying is the guideline has already been revised and updated but there is no training on that. But I understand there might be some people who may be trained at the woreda level. Again when I come to the woreda, it is a bit weak as I mentioned it earlier, and I suggest if the woreda strengthens the continued chain of support; usually, people from the woreda health office come to the health center if it has to do with different campaigns but they have not come for other activities. The health center is expected to increase the capacity of HEWs using different ways. But it is good if they come and review the different data, understand the situation on the ground and make the proper support as well. The health center should primarily need to know what to do to capacitate and strengthen the HEWs through checklist-based supervision that should be made every quarter; the HC has to develop a standard checklist and conduct quarterly supervision by selecting people from the different units to support the HPs. I think the HEWs will be strengthened if this is done.

4. How eCHIS implementation helps you with iCCM service delivery?

Probe for advantages:

- case identification? quality of iCCM case management? retrieval of client records? data quality? client appointment scheduling and/or defaulter tracing?
- **Ask why?**

Interviewer: How eCHIS implementation helps you with iCCM service delivery?

Respondent: I did not take part in any kind of training on eCHIS. As a result, I am afraid I may not give you detailed information about it. But from the layman's perspective, I can say that eCHIS is extremely important. Suppose let's talk over about a pregnant woman. If we know the date when she became pregnant with a child, then we can easily calculate the date when she will deliver her child. After the woman delivered her child, she is required to attend postnatal care. she must get the newborn child vaccinated. Generally, eCHIS will make it possible for us to identify the number of newborns in the respective localities and the vaccination status of a child since it allows us to determine if the newborn is vaccinated or not against certain disease types. So I think the eCHIS has a lot of significance.

Interviewer: Do you think eCHIS is helpful to identify a case?

Respondent: It may be helpful to some extent. I think eCHIS may recognize a newborn and signal whether that child is vaccinated or not. There is also a disease that might occur if that child is not vaccinated and hence, it might be used to indicate those probable diseases.

Interviewer: Do you think eCHIS is helpful to provide a good quality service to clients?

Respondent: yes, it has a lot of significance, especially for the quality issue. This is because, suppose a particular HEW may claim that she managed to get vaccinated certain number of new born children in her catchment area. She may also possess a document or a book that bears full information about the children. eCHIS will tell the HEWs whether or not that child is vaccinated. Secondly, it gives us information about the population living in some locality, for instance, the number of pregnant women in some village etc. This is how we can ensure quality of service provided to the community by the health facility. This condition will give rise to accountability. If they health workers are required to justify action, it means they will efficiently discharge their duty.

Interviewer: Does it have any benefit to gain access to or retrieve client data without any difficulty?

Respondent: it may be difficult to enter our data from CHIS to eCHIS. this is because after entering every information, information about age, sex might be missed when entering the eCHIS data. The other problem connected with ECHIS is that if I go away to other place, the collected information about my history are not updated with the most recent information. Because of this reason, they are a bit challenged to make the eCHIS functional.

Interviewer: If we include ICCM within ECHIS, do you think it will help us identify cases or ensure the quality of ICCM service?

Respondent: I don't think so. But I have not much awareness about it since I am not trained. But if it is integrated in the eCHIS I dint think it will completely help to ensure the quality or completeness. But I might be saying this because I don't have in-depth knowledge.

Interviewer: What I am saying is, if the module or the whole of the iCCM manual is installed in the eCHIS?

Respondent: Oh, if that is the case of course it is going to be great. But still our success depends on the availability of continuous power supply. What the eCHIS uses of the tablet, but there is a question and uncertainty if we can always use the tablet always for power interruption. Of course, the workers have a power bank but I don't think it is going to totally avoid the challenge. Therefore, I cannot claim with absolute certainty that the application will solve our problem in its entirety. All our problems will be solved only when we have a continuous supply of power and our mobile phone becomes fully operational.

Interviewer: Can you trace a defaulter or make a schedule using eCHIS?

Respondent: yes, we can. For example, if a particular woman starts to attend antenatal care, it will show you whether or not she has continued to do so as per the appointed time for PNC. The system does not allow a mere passage of a newborn child unvaccinated which will flag red if not vaccinated.

- **Probe for areas of strengthening?**

Interviewer: what do you think should be done to strengthen eCHIS?

Respondent: Firstly, the CHIS should first be entered into the eCHIS taking the utmost care. That it is in order to make the eCHIS functional we must carefully enter the CHIS into the eCHIS. I also feel happy if the two also go side by side or are implemented in parallel; both the manual system and the eCHIS since there might be some connection problems that might be encountered with the electronic system. The second is the challenge with the power supply but I know we might not bring any solution if we spend time talking about it.

5. How was the introduction of the iCCM/CBNC module into eCHIS affected the iCCM/CBNC service delivery?
   FOR LUME WOREDA ONLY extra

II. Implementation challenges

6. Describe the main issues faced by the health system to identify and treat neonatal infections in the community. What are the critical factors affecting the delivery of iCCM to clients?
   Probe:
   - lack of competence of HEWs, shortage of supplies and commodities; weak support system; low community demand?

Interviewer: Describe the main issues faced by the health system to identify and treat neonatal infections in the community. What are the critical challenges that have been encountered in the health system to provide the **iCCM** service delivery?

Respondent: the first challenge is related to the shortage of supplies and medicines. Especially the shortage of drugs has been a serious challenge for us to provide the iCCM/CBNC service. The second challenge is inadequate allocation of budget. As a result, if you have seen the condition of the health posts, they are in bad condition and the situations the health posts are in will make every client feel disappointed. I don't think they deserve the name. How can they heal a person affected by illness? The rooms are full of dust. Adequate allocation or lack of budget is to blame for the existing problem. The health posts don't have a guard to protect their property from thieves. The problems facing the health post you have visited today are showing the tips of the iceberg. I am sure you have noticed what the roofs look like other health posts have neither a toilet nor a fence to enclose their area and act as a barrier. Thirdly, they don't get any kind of professional support; neither the zonal nor the woreda health offices nor the health center is having a feeling of concerned to provide the health posts with professional support. the other challenge that is becoming serious for the health program implementation is the engagement of the community with different emergent tasks and the absence when we go to their homes during the house-to-house visit, most of the community are out in the field. For example, recently there was a COVID campaign but we could not find the community in their residence. They were busy preparing land for crops or performing any other activity. We were able to find no one in the village to treat people affected by trachoma. When we come to the iCCM / CBNC, the health posts remain closed due to different reasons and the engagement of the HEWs. In some health posts only one HEW is assigned to run the activity. There are health posts who does not have a HEW and some who used to work as three reduced to one. There is no way to meet the requirements of the community by a single HEW. This is so serious issue.

Probe:

- Regional/national state of emergency and conflict in the northern part of the country?
- How has that changed during COVID?

Interviewer: How has the situation in the country related to the war which broke out in northern part of the county affected the newborn health care service? Even if it didn't directly take place here, is there any indirect impact on the iCCM service delivery?

Respondent: whether is related to the conflict or COVID-19, it will have both direct or indirect effect on the delivery of community services. if I mention about the war, nobody was concerned about the health of the community. Everybody's main concern, let alone other leaders, even the leaders in the health sector was concerned was about collecting cereals and money from the community for the war. Even, a number of health workers were sent to the war front to give care to the injured fighters and

civilians from our health center. All essential services required as a woreda were closed since the main attention was the war and the war only; the health posts were closed since the HEWs were participating in collecting food items from the community to the war. Therefore, the war was having disastrous effect as far the provision of medical care to the community is concerned.

7. How do you think the COVID-19 pandemic and/or COVID-19 response measures affect the uptake and/or delivery of iCCM services?

   Probe:

   - Has COVID-19 affected your daily routines; your work on newborns; the community in terms of livelihood and vulnerability for newborn care-seeking
   - How has that changed over time?

Interviewer: How long did the impact emanating from the war lasted?

Respondent: It lasted for about three or four months or until the fighting stopped. If we also have to talk about the COVID-19 impact, it was having disastrous effect on our routine. For instance, there was the COVID vaccine at some point, and they didn't trust it at all and as a result no one was coming to the health post including for the iCCM because they thought that they would be given vaccine against COVID-19 without their will or desire. The community claimed furthermore that coronavirus pandemic was a political issue. As a result, the pandemic was having an adverse impact on every activity including the delivery of ICCM service. Parents were not willing to get their child vaccinated. There was a problem from the government side attributing for this challenge since we were advocating a connotation of like if someone is not vaccinated against COVID, to have implication in getting the services which lead the community to believe that if they go to the health center they would forcibly vaccinate them against COVID. This has resulted in decreasing the performance of iCCM, CBNC, ANC for mothers and other services. Last time there was trachoma campaign and someone in the community even mentioned we were giving them COVID in the form of tablet, the challenge with COVID was stretched to this extent and COVID was the second reason that impacted the iCCM service.

Interviewer: ok how did the COVID-19 pandemic impacted your day to day routines? And, also how the routines of the HEWs were affected in relation to the iCCM service delivery?

Respondent: as I mentioned earlier, the COVID-19 pandemic had a bigger impact on the community not to come to the services because it was communicated that they will be vaccinated for COVID if they go come to the health facilities. As a result, the health posts were closed, and the HEWs were also not working. It was having a powerful effect. A lot of things were said to the public about coronavirus pandemic that contradicted what the government has said. Only a small number of people were used to come to health facility at night after their condition became worse. Nobody was going to the health post to get treatment or any other kind of service.

Interviewer: How did you manage to bring about change in the situation with the COVID impact?

Respondent: The beliefs that affect community's attitude about coronavirus pandemic and other related issues still continue.

Interviewer: why was the community showing a feeling of anxiety or apprehension about vaccination against COVID-19?

Respondent: The community used to fully accept coronavirus pandemic as a political issue. The community residing in this neighborhood believed that the Tigrians are having the art of using alleged magical power to produce lethal drug. The community assumed that the enemy is planning to use the drug intended to cause death because they could not succeed in the war. The onset of COVID-19 and the war declared by the enemy happened at same time by chance in a surprising way. Secondly, at the beginning the health workers were not certain about the effectiveness of the vaccine. No date of expiry was written on the object containing the vaccine. Many people claim that it takes 10-15 years to conduct research and produce an effective remedy for an illness. So, well-informed community members were under suspicious circumstances. Thirdly, we have started to observe minor side effects of the drug. After they were administered, some people became sick of diarrhea which they claimed as a symptom of the harmful effects of the vaccine. I myself was sick of rheumatism and fever. But when you think about other medicine so far been provided, but since it is related with a political issue it is somewhat exaggerated.

Interviewer: Did you take any action to reverse the mindset and way of thinking of the community about the vaccine? In order to ease the impact on the services including iCCM.

Respondent: We have exerted strenuous effort to provide the community with the means or information to develop a positive attitude toward the vaccine. Every morning we used to convey a message to the community about the presence or absence of illness or injuries. COVID-19 was one of our agendas. We were telling the people who were in attendance of a meeting about the effectiveness of the vaccine. We worked hard to pass on knowledge to community during religious service inside the churchs' compound. What made us disappointed was that the community started to consider COVID-19 as contradicting to their religion. Let me tell a story about a priest. He asked me some questions why we did vaccinate every person against coronavirus. I tried to explain to the priest about the reasons. Then he argued that COVID-19 is 666 or relating to Satan worship. I tried to convince him that the government does not intervene in religious affairs. But I was not able to change the priest's attitude. He said that we are administering the vaccine on the left hand which implies it is the 666 that you are injecting. I tried to convince him otherwise, but he said it is written in the bible. Then I asked if he can show me stipulated in the bible which we went out together, but what he showed me was not related to his claim. It says in the bible, if someone is put a mark on his left or right arm then it is symbolization and affiliation of the 666 Satanism worship. However, I told

the priest that it is not putting a mark that we do it is rather a vaccine that we are administering to protect against COVID, and the scar will also disappear after 2 to 3 days. Anyway, we have tried our best to convince presenting ourselves at churches but it is not completely changed despite our efforts.

III. Adoption and reach/effectiveness

8. Explain to us any changes you observed as the result of the implementation of the COVID-19 adaptive iCCM implementation strategies? – What impact have the strategies had?
   - What are the particular features of the strategies that made a difference?

   Probe:
   - for a support system and linkages, motivation, and competence of HEWs, community**,** and awareness creation?
   - What do you think are the reasons for non-significant changes?

IV. Maintenance and sustainability

9. What are the high-level benefits that are attributable to this support/IR?

Interviewer: What are the high-level benefits that are attributable to this support/IR?

Respondent: I think the results obtained from the support were great. Well the community at some point has stopped the service due to COVID but then, we managed to persuade the community to resume using the service after a temporary halt. Generally, we found the support provided was very useful.

Interviewer: Tell me about your main achievements or benefits attributed to support?

Respondent: The success achieved to boost the capacity of HEWs is worth mentioning. More importantly, the assistance has provided us with the means to improve the capacity of HEWs. The second most notable is also related to benefits in terms of the acquiring quality data.

10. Please explain to us the feasibility of this support/ IR for national scale-up?

    Probe:
    - What features could easily be integrated into the existing system? Which not?

Interviewer: Please explain to us the feasibility of this support/ IR for national scale-up?

Respondent: I think the activities implemented by JSI are all feasible or capable of being achieved at a national level. This is because our community is experiencing a lot of problems some of which need such as CBNC and iCCM follow up which they have been making. We attribute the change of attitude on the part of the community to JSI's all- round support. Therefore, the support given by JSI to build the level of knowledge of the community can be cited as a feasible strategy. However, I cannot dare to say JSI alone could be feasible for national scale up.

Interviewer: Ok you mentioned JSI but out of the activities JSI initiated and implemented, which do you think will be scaled up at national level? And that can easily be integrated into the existing health system?

Respondent: the awareness creation that has been implemented at the community and commencement of the iCCM service at health post level is attributed to the JSI support. the awareness created at the community level will the future sustainability of the service at the health posts. The second, to our satisfaction and to the satisfaction of all other stakeholders is that the quality of data gathered by HEWs has improved. I think their system of data registration including the completeness of every in the register can be taken as excellent example that deserves to be scaled up elsewhere in the country. For example, JSI makes sure that all the necessary data are filled and checks whether or not HEWs offer treatment to their clients as per the chart booklet showing detailed information. However, the continuity of the activity depends on the woreda health office's strength to make a regular support to the health centers and health posts, otherwise it is hard to say the activity will continue if these are not met.

Interviewer: which support do you think will continue to sustain on its own?

Respondent: it is the iCCM service that is being provided by the HEWs will sustain but it still depends on availability of support. If they are not supported, this will also not continue.

Interviewer: what favorable conditions were put in place to keep on these activities going?

Respondent: They should keep on providing support for the health post and health center in order to keep implementing ICCM and CBNC continuously.

Interviewer: Why is it so obligatory to give them support?

Respondent: it is not clear for me. Nowadays we don't see a lot of people showing persistent and hard-working effort to carry out a task. Civil servants or employees in a government organizations are too reluctant to give us the required service.

Interviewer: do you think the capacity gap of HEWs has been fully addressed to carry out their routine successfully?

Respondent: yes I do. I think there is lack of sense of ownership of the project. HEWs and the leadership seem to demand or anticipate receiving something tangible in return for the jobs done. Lack of incentive was the main reason for members of WDAs to stop involving in any kind of activity. The smaller the amount of money paid or is due to be paid, the smaller the work done by a person. I think the whole issue revolves around getting financial reward.

11. How are the activities/efforts embedded in the PHC and woreda routines/work streams?
    - What implementation strategies are incorporated with the PHC and woreda annual work plan?

Interviewer: Please describe how you have incorporated ICCM into your regular activity such as plan of action, report, support etc.?

Respondent: it is not as strong as it should be. But, it is mandatory to incorporate ICCM and CBNC into our routine and the iCCM/CBNC has its own plan.

Interviewer: Does it have a separate plan for the iCCM separate for instance for under 2 month newborns?

Respondent: yes we do in the under 5 unit. We also have a review meeting every three months to evaluate the performance, for instance, the screening, SAM and MAM performance. We conduct supportive supervision using a standard checklist but it had some challenges as I told you. But we make a supportive supervision with a standard checklist having a separate section for the iCCM, CBNC, maternal health services. For instance, I have also visited majority of the health post in the last fiscal year using this standard checklist. Other than this, the services have their own plan also at woreda level including the iCCM and other services. We also evaluate our performances as a health center with the HEWs, WDAs and also we get evaluated at the woreda level.

Interviewer: do the HEWs also plan for the iCCM services?

Respondent: yes, they do. How they do the plan is first we receive the plans from the woreda and cascade it to the HPs.

Interviewer: How about the planning for the implementation of the different strategies like mentorship and others?

Respondent: yes, you are right. It is like what I said, if the support was strengthened it would be appropriate to plan for such things but I don't think it is necessary at this level. For me it would be appropriate to think about other strategies through a strengthened support but it is not the case now.

12. Do you have anything think is important to tell us that we have not asked you?

Respondent: what I finally want to say is if every concerned bodies are involved and supports the iCCM service. As you can witness, so far it has been only JSI that has been making the necessary support but it is also good if the woreda and other concerned bodies are also involved, and if they also don't leave the pressure on a single party.

Interviewer: Who are these concerned bodies you are referring?

Respondent: the woreda is concerned, zone, and even the health center is concerned with this. that is if these activities are strengthened and the activities started can commence.

Interviewer: Thank you very much for your time.

THE END

End line Evaluation of the PSBI implementation research; Full Transcription for an In-Depth Interview with a Health Extension Worker

Questionnaire ID	05
Area Identification	Dembecha
Name of Woreda/Zone/Region	Dembecha WoHO/West Gojjam Zone/Amhara Region
Name of facility	Health Post
Name of moderator	
Name of a note taker	
Date of Interview	16/06/2022
Participant #	01
Audio File #	HP_HEW_16.6.2022
Start time:	02:00 PM
End time:	03:12 PM
Transcriber/Translator	
Duration of IDI:	1hr, 12 minutes

V. Fidelity

13. Can you please describe the demand generation/SBCC activities you are doing on iCCM/ newborn health? What are the strategies used to raise awareness? If none, please explain why.

Respondent: In order to promote the newborn health care service at the HP, we try to reach the community members and raise their awareness in different ways. Among the ways include using different community gatherings like religious gatherings, the church, and other opportunities that can enable us to meet the community. Based on the information we provide the community members usually come to the health post to utilize the service. But, most of the time the health posts are closed due to the workload from field activity, and there might be a shortage of service providers, and during such time they might not get the service as they need it. This situation of getting the health post closed when they come to receive the service makes them upset.] and return back. Other than that

we tell every service that we provide to the community members like the under two months and under 5 years' child health care services.

Interviewer: Can you elaborate a bit more? What strategies do you implement to reach the community and try to raise their awareness?

Respondent: We don't have defined strategies but we simply use every opportunity like conferences at which community gathering occurs and tell the community members about the services we provide. For instance, we provide the awareness creation on Sunday at church and we also use the meeting of the women's development armies which they make every month and tell about the services. In fact, the women's development army meeting is fading recently and we are trying to re-establish it and start the meeting.

Interviewer: How and when do the development armies conduct the meeting?

Respondent: the development army meets every 29th regularly. They bring over every happening in their own village and report to us, and then we also aware them of what we have and about activities we conduct.

Interviewer: how do the WDA members create awareness?

Respondent: They will pass information to their village member communities about the services that we provide at the health post telling them to go to the health post to receive the service for free rather than going to the health centers and incurring unnecessary cost, and then they send them to us.

Interviewer: You mentioned that you use gatherings at church and meetings of WDAs. Are there any other ways that you use to do the awareness creation?

Respondent: we also do the awareness creation during the house to house visits while we are conducting other activities, like vaccination and PNC during which we conduct follow-up visit after a mother has delivered. During the follow up we tell the mother about the consequent services that she can receive from the health post.

Interviewer: How do you get information on the delivery of a mother?

Respondent: through an Action card and recently from the online system using our tablet that the health service providers or the midwifery at the health center at which the mother delivers will sync the information about the mother. The online system that we access using our tablet will provide us with information on the consequent services that the mother should receive from us.

Interviewer: Are there any more ways that you use to conduct the awareness creation? You mentioned Church, meetings opportunities, is there any other method you utilized to provide awareness?

Respondent: No.

Interviewer: Is it you by yourself who delivers the education at church?

Respondent: There is no one else.

Interviewer: Have you ever used banners, posts, or brochures to disseminate information?

Respondent: There is a post at the health post which describes about all the services that we provide. Other than that we don't use any additional materials.

Interviewer: does the post at the health post specifically describe about newborn health services?

Respondent: No the post is general but not specific to the newborn health service.

Interviewer: Have you ever planned to use banners, posts or brochures?

Respondent: No. Even if we want to there is flip chart. May be there is a banner having a list of the services posted outside. Other than that, there is nothing else.

Interviewer: Does the banner outside include the newborn health care service?

Respondent: No, it is not there.

Interviewer: Have you may be planned to do it, and any challenge you faced?

Respondent: No

Interviewer: You told me that the health posts are sometimes closed while they were supposed to be open. Can you tell me the reason for closing?

Respondent: For instance, here in this health post as you can see I am alone, and when there are campaigns like for the carter, we stay on field for ten consecutive days. So the health post will be closed during campaigns and other activities that we conduct in house to house basis. As there are no sufficient service providers in the health post that can conduct the field and the health facility services simultaneously, it is a must to close the health posts. In fact, this closing of health post affects the health service utilization of the community members as they become upset when they get the health post closed. The service provision would improve more if there are 3 to 4 service providers in the health post.

a. How is the engagement of communities/WDA networks in the implementation of integrated community-based case management (iCCM) of common childhood illnesses/possible serious bacterial infection (PSBI)?

Respondent: Recently most community members prefer to receive services from the health centers rather than health posts since they don't pay for the services and it's free due to the health insurance they have. They are mostly inclined to the services at the HC than us.

Interviewer: Why do you think is that?

Respondent: The community members usually have limited trust in the services at health posts.

Interviewer: Why is the trust of the community toward your service is limited?

Respondent: They usually do not believe that they will get good service at health posts just like the services they get at the Hospitals and HCs, that is even if we tell them that they can. For instance, regarding lab test, if we do perform RDT test for a child who is febrile and the lab result also turns out negative and then if we want to administer medicine based on that result, they might question why is it this

test only, and then we refer them to a HC to have a full lab test and give stool samples et cetera. Since complete investigation is not done here and they incline to that.

Interviewer: What did you do to change their attitudes towards the quality of the services that you provide so that they will be more interested to receive the services from you rather than from the health centers or hospitals?

Respondent: I usually tell them to receive newborn health services from the health post in meetings. Even a command post was organized and conducted awareness creation about newborn health care services from the health post.

Interviewer: What should be done more to raise the awareness of the community members so that they become interested to receive the service from the health post?

Respondent: Improving the capacity of the health post in terms of manpower, so that they would always get the service whenever they needed it and upgrading the laboratory tests for better service provision would help. In addition, as you mentioned earlier, using awareness creation materials like banners and posting it at places that most community members can see. Using pictorial posters that can briefly and easily disseminate the services that the health post provides will increase the chance of utilization of the service from the health posts.

Interviewer: What kind of support do you need from other partners?

Respondent: Recruiting additional service providers and preparation of banners and pictorial posters.

Interviewer: How about the support from WDA? What was their support and role in the newborn health care service utilization and improvement?

Respondent: They pass information about the service to their neighbors and other community members in gatherings including the church. They advise mothers whose newborn child shows symptoms of diseases to take their children to the health post. But be it the WDAs or the 1 to 5 organization is becoming loose that it needs reestablishment and strengthening again. In the effort to strengthen it, even yesterday an official was discussing with me about the need for strengthening the WDA and the 1 to 5 network because they are useful in supporting us in the awareness creation activities. Currently, the responsibilities of the WDA is being pushed towards us and I am urging for reestablishing or strengthening the groups but the WDAs are important for everyone and I alone could not reach all the society member nor have sufficient information about each and every household. However, the presence of the WDAs will immensely support us since they know each and every household support us in facilitating information exchange as they can have updated information about the households. So, we are now in the process of re-establishing and strengthening the loosened WDA network.

Interviewer: What is the reason for the loosening of the WDAs?

Respondent: Among the reasons include lack of motivation and the feelings of fatigue among the WDA members; the absence of some motivational payments that they used to get earlier when they are involved in campaigns and when this was also fading away, they also start refrain. When we also recruit married women, they are telling us that we are jeopardizing their marriage; in relation to this, I have once organized a ceremony with the aim of replacing weak WDAs, but I was truly sad for two or three mothers because they were told to go and pushed away by their husbands. And this is challenging us a great deal.

Interview: Why do husbands show lack of interest for their wives' membership in WDA?

Respondent: they do not want their wives to waste their time on social activities but rather they are demanding their wives to serve their family in full time.

Interviewer: How does the participation of a woman in WDA affect the condition of their husbands or their family?

Respondent: The activities in the WDA does not affect the husband or the family that much. But the husbands usually moan about the time that their wives spend for the WDA activities mentioning some reasons that are not significant. The husbands usually say to their wives that their wives should at least look after the house as no one is available in the house since he is staying in the farm and the children at school. Due to this complaint from husbands most women are losing interest to be a member of WDA. When we also use prostitutes, they are becoming bored; we also use prostitutes in larger number but they are getting bored. We are also finding it hard to replace them after the prostitutes leave because most available ones are married. When we have no option, we might use the married ones, but it is going to be disrupted. However, the prostitutes during their involvement help us a lot, and they mostly come to us.

Interviewer: What is their acceptance in the community?

Respondent: it is good, and they also do well.

Interviewer: is there a unique reason that force you to recruit prostitutes as WDA member or is it just common in most places?

Respondent: It is like this everywhere and obvious. It is a common understanding that most married women do not have the desire to come and serve here. We sometimes select an unmarried woman who just completed her education but such a woman does not stay as a member in the WDA for more than a year as she most probably becomes married or will depart in search of a job. Once she became married she immediately drop her membership. The activities also become delayed while trying to familiarize the ones and when there is so much replacing.

Interviewer: In your opinion, what do you think is the solution that can enable a woman to sustainably stay as a member of WDA?

Respondent: They need to be incentivized or compensated for the time they waste for their community engagement, like the campaign for Carter. Regarding carter for instance, there used to be at least

some compensation. We are also witnessing these days that it is difficult to waste time for nothing, hence it is at least good to cover their daily expenses. Previously, when there was some payment for some activity, the women used to compete to be a member. Even the complaint from husbands to their wives would stop if the husbands come to know their wives will get some incentives for their involvement. It has been the norm before and such things used to be made, but now everyone is mocking them that they are wasting their time for nothing, and wondering what they could get in return not minding about the health benefit they could get too.

Interviewer: is there any other form of social organization you use other than the WDAs to implement the newborn health care services?

Respondent: No there is not. But there is what we have been trained as "village health leaders" which we have not yet provided them training. We wait for that, and it was informed training will be provided but it is delayed. But if this was available, a village health leader would have strengthened it by having two WDAs under it, and they submit reports to us. we are waiting for that but still, if there is nothing again like as an incentive, it won't be effective.

Probe: What are some of the biggest challenges with SBCC activities for newborn care?

Respondent: The over workload is the main challenge. The task is difficult that it demands traveling through geographically difficult areas.

Interviewer: How about the location of the health post? Is that center to its catchment areas?

Respondent: Except for the two kebeles that tend to use their nearby health centers the location of this health post is almost centered.

14. Could How do you describe the strength of the support you got from the PHC and the project as well as your efforts to address barriers to delivering iCCM/PSBI during COVID-19?

    Probe: What was changed?

Respondent: It is good that the staff from the office, the health center, and some projects visit us and fill the existing gap that they observed. Due to workload, we continuously do routine tasks that we sometimes forget some procedures and techniques, so they help us in recalling things. Other than that they don't provide me a solution for major problems I tell them.

Interviewer: What were the supports that you received from the health center and the project staffs?

Respondent: They help me in guiding how to treat or medicate a patient by looking at the chart booklets and recordings I completed for a newborn I care for and identify the gaps in the process and provide me technical support. That is both from the HC and the project.

Interviewer: What else do they support and how often do they visit you?

Respondent: Regarding the frequency of the visit it is quarterly.

Interviewer: Who are the ones who visit you quarterly?

Respondent: The health center staffs visits quarterly and the project staffs in between like in every three months.

Interviewer: What other support? For instance in resource and others that you received?

Respondent: it is the health center that provide us with supplies; we fill out the HPMRR request form and request, and then they provide us, and there is no problem regarding the supply, except for the gentamicin and zinc that once we run out and could not get additional supply as it was also stock out at the health center but now we got both.

Interviewer: Are there other kinds of support like evaluation and review meetings, training, and others?

Respondent: Yes, there are such supports.

Interviewer: If so please elaborate on all the supports that you received regarding iCCM from the project or the HC?

Respondent: regarding iCCM it has been very long, in general, the wereda calls us around three months ago, and gave us a rough training but they didn't provide us a formal training. However, we have had some revisions at the woreda as well as the health center which means a sort of experience sharing having the register and chart booklets by which one HP exchanges with the other, and we discuss on the gaps. They support us in this manner.

Interviewer: How about review meeting and other activities?

Respondent: we have once went to Kossober for a review meeting. It was the project which facilitated it.

Interviewer: How about training? Is there any training you received on iCCM?

Respondent: No, except for the first training that we took long ago, more than a year or two.

Interviewer: How about an evaluation meeting and other activities at the HC?

Respondent: At the health center there was few but regarding iCCM it has become loosen recently that no evaluation meeting about it is being conducted.

Interviewer: How about earlier? How was the follow up?

Respondent: Earlier they used to visit us monthly and provide us refresher training frequently. But recently, I think it is also due to the current situation in the country. Now it is not like before, and the focus on iCCM performance is not that strong when we evaluate our performance during the monthly meeting.

Interviewer: What is that you said about the current situation in the country?

Respondent: it is just the situation in the country that is the call to war for existence (yehelewena Zemecha) which we have also been wondering around. We really didn't think we would stay this long.

Interviewer: Tell me, what is the influence it had brought? How did the current situation in the country affect your activity?

Respondent: Well, if we for instance look at the government structure there is being tied up and over-engagement with emerging tasks, and when such activities arise we interrupt this activity and move to the emergent task. So we would rather focus on these tasks and have never evaluated the iCCM performance.

Interviewer: How long have this been interrupted, how long since you stopped the evaluation?

Respondent: it has been very long, and we have never evaluated the iCCM performance in this year.

Interviewer: what were your role in the “march for existence call for the war” that had an impact?

Respondent: there is money, red peeper/chilli, flour and we have also been collecting 25,000 ETB per day, that is every professional also having its own assignment. Even at the woreda they were also engaged in preparing roasted wheat, barley flower, Enand other food items for the war. In general, things were a bit messed up, and the activities were coming one after the other.

Interviewer: So when was the time that you think were being provided with best support?

Respondent: Around 2008 EC before six years.

Interviewer: Did you receive training in the last one year on iCCM ?

Respondent: No. but we conduct review meeting on newborn health from Zero to two months both at the health center and Kossober.

Interviewer: Was there any mentorship provided for you on iCCM from health center focal?

Respondent: No, there was not that much support from the iCCM focal. Regarding iCCM service, we are working using our earlier knowledge and except for newborn health care, the iCCM is so much delayed. We have not even got the update on iCCM, for instance, TB has been integrated and I was even the one who resisted when they bring the revised chart booklet on iCCM without any training or orientation. It was actually simple to use since it was in Amharic and brought it back to use after some resistance.

Interviewer: So how do you manage the identifying, screening, treating, and referring the child without receiving training on the booklet?

Respondent: the support for iCCM is too late and loose. In fact, they might try to supervise it on a quarterly basis but the support they provide is insignificant.

Probe: Was the support you got from the project and PHC helpful? What could have been done differently?

Respondent: the support is in general helpful that the supervisors remind us the skills and techniques that we forgot due to routine activities that we were involved in for a long time. So the support they provide is good as we correct our mistakes as per their feedback.

Interviewer: You told me that they do not conduct the visit frequently as it should be. So what should be done to solve this problem? What do you suggest to help you provide the service more efficiently? What is your suggestion to be corrected?

Respondent: The focal should make supportive supervision regularly at least on monthly basis. This helps us to timely fill the gap that we have in the service provision and even thanking those who performed well based on a frequent visit is helpful for the improvement of the service. The reason for our decrement of performance and capacity is the workload that we usually carry. We are usually forced

to be involved in the activities of all sectors, not just only health so if they come to support us on our profession in every month, and a frequent visit from the supervisors is necessary.

Interviewer: What else can be improved and corrected?

Respondent: The other is adding more professionals. Other than this, as I mentioned it earlier is a banner and pictorial advert that if we could display them in different places, it will be strengthened more.

Interviewer: What is the exact number of times that you received support this year? How often did they exactly do that from the health center?

Respondent: There are supporters of course but the question is, are all supporters considered as supporters

Interviewer: Please explain to me what are the challenges are concerning this? Why do you say that?

Respondent: the supporter might come and tell you he has not got the iCCM training when we ask him something about it. there are always supporters to the HPs assigned by the HC but they are not all the same, and might tell you he does not know about iCCM or trained on it when you ask him.

Interviewer: But how about the iCCM focal who are allocated for this cluster by the HC?

Respondent: But how many focals are available? Here is there are 5 HPs, and hence only one HP might get that iCCM trained supporter, and it could not reach and support every HP. Well had he been going to every HPs and making a separate support on iCCM only, it would be great.

Interviewer: ok if that is so, how many times have you been supported by iCCM trained focal in the year?

Respondent: I did not get a visit from the iCCM focal this year.

Interviewer: So, how did you get support on iCCM from the health center this year?

Respondent: normally the supporters during their field activities asks you things, and we support each other on general technical issues

Interviewer: How about similar support on how you are providing medication for newborn health care looking at the iCCM chart booklet et cetera, was there such support?

Respondent: No there was not much about this.

Interviewer: How about support from projects on iCCM? Was there any support provided from a project on iCCM this year?

Respondent: There was no any on iCCM that I was supported here.

15. Do you think the support system is helpful (mentorship and on-site coaching methods, supervision, technical support, PHCU level PRCMM, etc.) to enhance your skills [that is HEWs' skills] over the traditional off-site training and woreda level review meetings?

Respondent: regarding skill, I have gained on newborn health care that is on infection which they have showed us—we used to skip that section in the earlier times, so the project helped us notice that gap and showed us how.

Interviewer: When did you see it and also how did you they show you?

Respondent: that, the project helped us hold an event for experience sharing among health posts organized at the health center by exchanging our registers among us; one of the identified gaps was also detected from us, and we have discussed about it. so the project supported us in this manner.

Interviewer: was it a form of training or what? And where was the place?

Respondent: it is a kind of review meeting and training yes, that is aimed at filling the gaps on service provision. It was conducted at Anjene health center at which all the five health posts have gone there having their register and chart booklet, and evaluated our performances, how we implemented the activities and learn from one another by exchanging our registers. The issues in the meeting include evaluation of our performances, then we identify the gaps by exchanging our documents with little support from the health center.

Interviewer: so this is what you remember, the one-time support you got that improved your skill?

Respondent: JSI used to support newborn health care from Zero to two Months strongly. If I am not mistaken it was Mr. Kebebew that used to support us; we have been receiving consistent support until the last four months before it was interrupted.

Interviewer: how helpful was the support in terms of improving your skill?

Respondent: **i**t enabled us to properly provide the newborn health care/medication; it improved us in correctly administering drugs.

Probe: for acceptability of the strategies **(for HEWs only)**. How could the support system be improved?

Respondent: it is very good because it allows us to recheck ourselves, and to ask myself why I missed things.

Interviewer: Those benefits you told me you got on the skill, is it acquired when you go to the HC or is it from what they showed here onsite?

Respondent: it is at the HC from the meeting we had.

Interviewer: Do you have any suggestions and recommendations to improve the support in improving your skill?

Respondent: if training is provided and performance reviewed. In addition to this, if we also don't read and update ourselves it is still going to be the same. I myself would like to read if I were to have time but currently, I am tight. There is a need for reading for improving your service provision like treating patients but I don't have sufficient time for that because the workload does not leave me any time to read.

Interviewer: did you say that you haven't received training on ICCM?

Respondent: I took training on ICCM once around 2007 or 2008 during which good attention was given from the government.

16. How eCHIS implementation helps you with iCCM service delivery?

Probe for advantages:

- case identification? quality of iCCM case management? retrieval of client records? data quality? client appointment scheduling and/or defaulter tracing?
- **Ask why?**

Probe for areas of strengthening?

Respondent: We didn't start working on ICCM using eCHIS and we also did not receive the training yet, and we also did not provide service using this system. However, the system brings up anything to our attention that we could have missed while providing care for a newborn, for instance, while we are caring for a child the eCHIS might send us message what to do. The eCHIS updates us on every needed information, even while feeding information you press Ok, it asks you if you have taken a training, or if have the medicine, and it also stops you when you make some mistake. This is very best. And if we can start the implementation sooner, I think we can score a good achievement. But it also has some risk; holding the tablet and moving house to house will be risky and we also worry that they might take it from us and usually don't bring it with us as there are some service providers who already were stolen. I usually do not hold it with me but if I do for any reason I put is inside an ordinary plastic bag (Zembil) to hide it while traveling. But the system is so helpful that it recalls you the activities you may forget.

Interviewer: so do you feel that you are at risk using the equipment?

Respondent: yes, very much that even my relatives can't be trusted

Interviewer: what are the activities that you are using the eCHIS for?

Respondent: we did not start to use the system100%, and the plan is also to finlize updating it within 15 days, that is from the CHIS to the eCHIS; the CHIS is a source for the eCHIS. Hence, every service will be fed in the system, and what we are currelty providing os family planning, PNC, ANC3, ANC4, but we refer the ANC1 to midwiferies. The service is not that successful but there is the initial activity.

Interviewer: How about delivery?

Respondent: yes, the delivery is included that after delivery we receive action card from the system. We refer the ANC 1 to midwifery and they resend us the action card as feedback.

Interviewer: You mentioned about the delivery ANC and other services but is there any indirect way that you have used these services to the iCCM advantage?

Respondent: Not yet.

Interviewer: For instance, do you use the system for case identification?

Respondent: No it does not have the case definition for every disease for iCCM. It is not yet installed. But it is available for the other already installed services. But regarding the ICCM we are planning for the future.

Interviewer: Can you search and select patients that you already register from the system?

Respondent: Yes. For instance, the family planning clients list is available in the system. The system was loaded at Debre Markos on my tablet but the earlier mobile, Lenovo, was inefficient that the application is already lost. Recently I changed the mobile that now I use Samsung. So you can select the head of the household from the system that it will give you information about the household like the number of household members at ease.

Interviewer: How about the significance of the system in data quality?

Respondent: it helps keep the quality of data very much for instance if you enter data that is not valid, it will immediately erase it and flags red. The data checks consistency that if you first entered the member of a household under a household head, and if you enter a different name not registered later, it flags red. The system has significant importance in keeping the quality of the data that it won't let you enter what you did not first feed it.

Interviewer: Is there any other way that you witnessed the importance of the system in keeping the quality of data? Like anything that it flags red when you enter invalid data?

Respondent: it will not allow you to proceed if you commit any error at some step. For instance, if you consider family planning after you enter data that she is pregnant then it will ask you about her last date of menstruation. And if the date is her 8th day it will stop you. When you plan to provide some method of FP like depo or other, It will check her weight and prevent you from providing her the service if the method is not compatible with her weight.

Interviewer: How about notifying appointments and defaulters?

Respondent: Regarding the appointment the system sends notification in terms of color like black yellow and red etc. and if it shows red it means the deadline is passed so you have to follow the notification strictly so that you won't miss an appointment for specific service. Except the risk of losing the tablet, the system will simplify your load by reminding you all you need that there is no need to use other manual methods.

Interviewer: Anyways, you are not using it to indicate you anything for the iCCM service? Is it not from delivery that your follow-up for the newborn starts and if so how do you make the visits, how would you be notified for a new delivery and identify a newborn?

Respondent: An action card will be sent to us from the HC, and we also communicate through phone. For those mothers delivered at the health center, the midwiferies will send us and then we make the follow up visit for PNC that it if possible since I told you we have shortage of health care providers, but we have to visit the newborn as much as possible. We also communicate the mother, and tell her to bring the newborn if he gets sick and also counsel her on the vaccination schedule, on how to breast feed, and keep him warm etc. Other than that, it does not tell us about the newborn sickness but it does tell us about the availability of a newborn.

Interviewer: so if the eCHIS informs about the availability of a newborn, so why are you not using it to plan for the PNC? Is it possible to do that?

Respondent: Yes, it is possible but there might be still things remaining; it is not yet installed, we are not trained.

Interviewer: So in order to fully implement what do you think should be done? How can the system implementation be strengthened to help the ICCM service by the system?

Respondent: What we want to be integrated in the eCHIS includes for instance, sepsis, and if Sepsis is integrated, all the danger signs should be incorporated and generally if all the services are incorporated in the system that is if everything in the chart booklet is included. So you can read the chart booklet so that all the possible dangers, diseases and symptoms are included in the system, we can easily run through pages, read and provide the care.

Interviewer: so you are saying if all the CHIS converted to the eCHIS?

Respondent: Yes.

Interviewer: How would it help with anything if it is made like that?

Respondent: If we want to prescribe drugs, rather than carrying the chart booklet it would be easy if the chart booklet can be loaded into the system so that we get all the information on the system easily.

17. How was the introduction of the iCCM/CBNC module into eCHIS affected the iCCM/CBNC service delivery?
    FOR LUME WOREDA ONLY extra

VI. Implementation challenges

18. Describe the main issues faced by the health system to identify and treat neonatal infections in the community. What are the critical factors affecting the delivery of iCCM to clients?

Probe:

- lack of competence of HEWs, shortage of supplies and commodities; weak support system; low community demand?

Probe:

- Regional/national state of emergency and conflict in the northern part of the country?
- How has that changed during COVID?

Interviewer: Could you please describe the challenges and bottlenecks that affect the service provision of the newborn child health service in general? It can be with regard to health system, community, resources, attitude towards the service, and other.

Respondent: the first thing is that we are disgusted with being HEWs, one because there is the issue of transfer which even if it is done, it won't be fair; you will be transferred to a better place if you have someone from the inside and if you don't, you will be thrown to a remote location. For instance, if we see my case I was away for education reason but when I return back I was told to come here and since I am poor, I have come here and struggling. Beyond the remoteness, now I am assigned here

alone that the 2 HEWs who were assigned here with me are transferred to other kebeles due to shortage of service providers. The salary is too low that it discourages us to perform better. The workload on HEWs is too high that we are working being stretched beyond our capacity that easing this condition by recruiting additional HEWs is necessary. It would be better if we can get updates and the payment should be revised and improved. I, being level 3 and with higher experience may get less salary than a fresh HEW, and hence the civil service should revise its system of salary increment. Other than that there are no as such significant challenges. But when we consider the community, the community will be happy and will get better service if we the service providers are happy. So if the salary is adjusted we would provide better services and if not things are discouraging.

Interviewer: Are there problems that are related to supplies?

Respondent: regarding the supply, the health center supplies us with the drugs it has, we receive the supplies that we need through the HPMRR request form. Thus there is no that much problem with regard to supplies.

Interviewer: How about the support from the health center?

Respondent: The support should be strengthened that it is currently very loose, especially in our clusters. There is a need to be motivated and energized that currently I am observing very loose initiation among the service providers at every level. There must be a limit in assigning number of maximum clients by a HEW currently I observe tendencies of leaving a task that should be covered with two HEWs to one and the task that should be covered by three to two and so on. So if this over-allocation of tasks to HEWs is improved we would perform better.

19. How do you think the COVID-19 pandemic and/or COVID-19 response measures affect the uptake and/or delivery of iCCM services?

Probe:

- Has COVID-19 affected your daily routines; your work on newborns; the community in terms of livelihood and vulnerability for newborn care-seeking
- How has that changed over time?

Interviewer: Was there a challenge related to the state of emergency that limited your service provision on newborn health? For instance, the state of emergencies due to COVI-19 or the war?

Respondent: Not much. But we were restricted to limit our meetings. Especially the response to COVID-19 was so intense that I myself was so afraid of it, and because of that meetings including with the women's development armies were interrupted. The other challenge related to COVID-19 was its effect on our referral of TB patients to health centers. During the time of the outbreak, the TB patients that we referred were not accepting our referral fearing that they will be stigmatized and or will be sent tan

o isolation center. For this fear of stigmatization and isolation, patients were not even interested to visit the health facilities they get sick.

Interviewer: Are these TB cases children?

Respondent: No they are the adults. Our meetings were interrupted.

Interviewer: How about now? Is the meeting resumed?

Respondent: We are struggling to re-establish and strengthen the women's development army, and currently it can be said that the structure is already loosened and become dysfunctional, and we are also confused. May be the village health leaders I mentioned. I hope could bring us something good if we are able to strengthen and implement it.

Interviewer: Why did it became loosened and dysfunctional?

Respondent: the WDAs network is loose from developing boredom, and hence we are working on replacing those bored WDAs, and if we can also manage to commence and engage the village health leaders in the activities, I think we can see something different because one village health leader would be having two WDAs under them to implement the activities which encompass up to 90 households which also contains the WDAs, 1- 5 network, and if they can at least engage on holidays and send to us, I think we can get something great. But we have yet started it and have not fully implemented it.

Interviewer: How about the war? How does it affect your service?

Respondent: There is no significant problem related to the war it was the COVID-19 that we were afraid of.

20. Explain to us any changes you observed as the result of the implementation of the COVID-19 adaptive iCCM implementation strategies? – What impact have the strategies had?
    - What are the particular features of the strategies that made a difference?

    Probe:
    - for a support system and linkages, motivation, and competence of HEWs, community**,** and awareness creation?
    - What do you think are the reasons for non-significant changes?

Respondent: Regarding COVID-19, there was not that much problem due to COVID but we were being supplied with materials and supplies like face masks, and sanitizers for the prevention. However, it did not pose any major challenge, and there was no activity-limited due to COVID.

Interviewer: COVID-19 was novel for significantly affecting a lot of activities in different areas? So, what do you think is the reason that it does not affect your activities?

Respondent: In fact, we were afraid of COVID-19 at the beginning of its outbreak but later, as there were no cases of COVID-19 found in our areas, we continued our normal activities.

Interviewer: Were not there any activities affected due to COVID-19? Was there any activity that was limited due to the outbreak as a professional to engage in your normal routines?

Respondent: We were afraid of conducting house-to-house visits and discussing with households. We were also afraid of meeting an outsider, like from Addis Ababa or strangers from other far places, that we were distancing ourselves from such strangers. During the household visit for the newborn health service, we were afraid and we tried to keep our distance and even we had a feeling that it is better not to do it at all.

Interviewer: How about the community, were there free to interact as health professionals and bring forward their newborns for medication?

Respondent: Yes, when are also telling them about COVID-19, they might say nothing is also known about us the professionals. Even if they don't tell it to your face, you can see it in their eyes that they are suspicious of us.

Interviewer: How does this attitude of the community then change over time?

Respondent: Thanks God now there is better situation, and also there has never been anyone who got sick, recovered, died in the society or among relatives. With regard to this, it is good and now they think COVID does not exist anymore.

Interviewer: Was there any action that you the service provider took to change the attitude of the community members? And was there any support from other health facilities regarding changing the attitude of the society toward COVID-19?

Respondent: We were raising the awareness of the community members on a household to household level, especially on the ways of prevention of the transmission of the virus. I think it was a year from now that we were conducting intensified activities regarding the creation of awareness on the prevention methods of the transmission of the virus. We told the households about things that they can do by themselves that can help prevent the transmission of the disease that significant number of them were implementing it. Among the actions that most households implement to prevent the transmission include the preparation of handwashing facilities near their houses.

Interviewer: Who provided you the support for these activities?

Respondent: We and other support providers, who were recruited from the community members, were trained by the health center.

Interviewer: Were there unique projects or interventions implemented to tackle the obstacles, which may happen due to COVID-19, in the service provision activities of the new born health service?

Respondent: No such unique intervention was implemented.

VII. Maintenance and sustainability

21. What are the high-level benefits that are attributable to this support/IR?

Respondent: What I think has benefited me the most is in the technical capacity areas that I improved and helped me fill my capacity gap in providing newborn health care.

Interviewer: What were your gaps?

Respondent: in terms of identification (disease), and making proper diagnosis and investigation in newborn health care which I had some gaps. So they helped me fill that.

Interviewer: What was the specific support you received that helped you fill this gap?

Respondent: They use dolls to represent a child and clearly elaborate on ways of examining a child including where and how to touch the part of the body like the chest of the child during a physical examination and make a child become stable through a proper approach. So they demonstrated to me that and filled my technical gaps.

Interviewer: What else?

Respondent: there is nothing else I remember.

Please explain to us the feasibility of this support/ IR for national scale-up?

Probe:

- What features could easily be integrated into the existing system? Which not?

Respondent: in order to sustain the activities that is still having the support, is to avail a separate room for the iCCM service. I have thought about using the other room available here but there is a limitation in capacity. We can also observe the fence which is not optimal but overall a place intended for newborn health care service should be clean.

Interviewer: I am asking again, is there specific support or activity that you think could easily be integrated into the existing system with no significant support, and also you say, this is a difficult one and won't be integrated unless it gets support in relation to the newborn health care service?

Respondent: all what I can say is that all the initiated activities should sustain.

Interviewer: Can it sustain without any support?

Respondent: Yes it will continue.

Interviewer: Which activity is it among those supports you were provided or activities you implemented, which would sustain even if there is no support?

Respondent: it is the newborn health care service, I will sustain it even if the workload and being here and there might hinder to some extent. But otherwise, it has to be sustained.

Interviewer: For instance, can you do the identification and medication by yourself with no support?

Respondent: Yes, I can do it.

Interviewer: But what do you say should be changed or support you need to sustain this activity?

Respondent: I need thermometer but I can request and also a weight scale. If these materials and supplies are availed, I can continue providing the service.

Interviewer: What I focused on your activities is because you mentioned early that there is no support provided to you from others? So, does this lack of receiving support will affect your continuation of the service?

Respondent: The support will continue.

Interviewer: Do you think that the support will continue without problem? And which support should continue?

Respondent: I only need technical support in case I sometimes forget some technical issues due to workloads, and they make some evaluations and measure the performances. So it is good if the support still continues.

22. How are the activities/efforts embedded in the PHC and woreda routines/work streams?
    - What implementation strategies are incorporated with the PHC and woreda annual work plan?

Interviewer: How are the iCCM activities embedded in your routine activities at the HPs? Are they included in the house-to-house visits and also here at the HP?

Respondent: frankly speaking we don't make a house to house visit for the purpose of iCCM service, and it is mostly here at the HP that we provide the service. Even if we go there, we will not have all the materials for the iCCM service because

Interviewer: why is that?

Interviewer: Why didn't you hold the necessary materials?

Respondent: If you consider under 5, there is vaccination, deworming, vitamin A and having the equipment like weighing scale and others, and hence we don't carry all the materials all the way there and provide the full services. Thus what we do is just provide the service here if they come to the health post since all materials are complete, and we also don't carry the weight scale and the medicines. Hence, I cannot say that the iCCM service is being provided successfully to a household level, as far as I am concerned.

Interviewer: Do you check at least for the presence of some disease on a child as part of identification while you conduct any house to house based activity, like vaccination or other?

Respondent: Yes, for instance, while we do the awareness creation at the church we tell them about the symptoms that a child can show for a disease.

Interviewer: Here I am asking whether you do some physical checking for identification while you come across for instance, a child under two months, while you are providing some house-to-house service?

Respondent: Yes, we always check for a child for identification whenever we come across a child while we are doing any house to house service. I always check a child or mother, and at any house-to-house visit, my attention is on the mother or the child, especially the children so I will check the child's health status. If for example, I come to see a child which is thin while conducting any house-to-house service, I will ask the caregiver for the status of the child. I will ask her for details about the child so that I enquire whether the child was sick or showing some symptoms of illness and I will then conduct some physical checking to understand the health condition of the child.

Interviewer: What other activity, which is related to newborn child health service, do you do while you are doing any routine activity like including the iCCM service in performance evaluation meetings or others? for instance, is the iCCM service included in the reports, performance evaluation meetings?

Respondent: Yes it is included.

Interviewer: Can we say the health center has also included it in the report?

Respondent: Yes, the report on ICCM is included in the report that we provide to the health center. For instance, we report Diarrhea, OTP, and Pneumonia

Interviewer: is it for the newborns?

Respondent: it is in the under 5. There is no separate I included for the under 2 month children. It is out of thought that it could be included within the under 5, we were actually told to do so.

Interviewer: How about the health center in including the newborn health service in their planning and reporting, or in the performance evaluation?

Respondent: It fluctuates depending on the intensity of the support, if it is low it also slows down accordingly. If the intensity of support from the project becomes high, it also becomes intensified, we were once highly engaged when Mr. Kebede engaged us, but if he slows down, it also slows down at the health center and HPs; I can't say we will do it the same way.

Interviewer: Is the service included in the checklists of supportive supervision at wereda and higher levels?

Respondent: They try but it is not that successful.

23. Do you have anything think is important to tell us that we have not asked you?

Respondent: yes, this is my personal opinion. But the other conditions to sustain are fulfilling thermometers, measuring weights, and especially the measuring weights, I am using that one you see over there, it is a modified one.

Interviewer: What is that, please explain it since it is audio?

Respondent: The current scale that I use, the hanging scale that can weigh up to 25 kg, is not safe to use, and the caregivers usually feel that their child will fail and become harmed. So, it would be better if the type of the scale is changed to digital with a safe tray to lie the child and measure. We also conduct house-to-house service provisions the leftover of the disposable plastic bags from us could bring about environmental pollution. So, it would be better if we can be provided with a standard bag to hold all the materials required for the iCCM service and the disposals. We are currently using festal, which is not safe because it easily tears and the materials including the disposal will fall to the ground, and collecting those is something shameful. So, if we have a bag that can hold all the field supplies safely it would encourage us more.

Interviewer: What else do you recommend similar with this, since now you are addressing the question I was going to ask you at last?

Respondent: ok if this is so, what I also want is a uniform for the activities just like what is provided in the agriculture sector; they provide them with quality shoes, umbrellas, and other goods considering also the inflation or the high living cost. If we even wanted to have better quality shoes, we don't

have the means. So because of this, it might be difficult to engage especially during the rainy seasons.

Interviewer: Ok is there anything you would like to add at last, that were all my questions but if you have anything to add that you think should be addressed you can tell me now.

Respondent: Ok thank you. I have mentioned already what I want to be improved, and if you can support the woreda, they are saying recently that the budget has been utilized for different reasons while we are still donating our salaries to the call on war for existence. If the uniform demand for HEWs is fulfilled, and also the weighting scale be availed, it would be great.

Interviewer: Ok, I thank you very much for your time.

THE END

End line Evaluation of the PSBI implementation research; Full Transcription for an In-Depth Interview with a Health extension worker

IDI_Daba HC_Daba HP_HEW_17.6.2022

Questionnaire ID	10
Area Identification	Daba
Name of Woreda/Zone/Region	Daba HP/West Gojjam Zone/Amhara Region
Name of facility	Health Post
Name of moderator	
Name of a note taker	
Date of Interview	17/06/2022
Participant #	01
Audio File #	_HEW_17.6.2022
Start time:	09:00 AM
End time:	10:20 AM
Transcriber/Translator	
Duration of IDI:	1hr, 7 minutes

VIII. Fidelity

24. Can you please describe the demand generation/SBCC activities you are doing on iCCM/ newborn health? What are the strategies used to raise awareness? If none, please explain why.

Respondent: I am Seble Ayalew [Name changed for confidentiality reason] and I work here at Daba health post. Regarding the Newborn Health service, first of all in order to raise the awareness of the community we use different meetings like religious places, and tell the services that are being provided for children under 5 at the health post for free. In addition to this, we also inform the community members that we provide malaria test for free for those members of the community that are above age of 5. We use every different incidents or purposely conduct activities and inform the community that we provide health services for children under 5 for free.

Interviewer: What are these different incidents that you use to raise the awareness of the community? Please elaborate it in detail all the activities.

Respondent: When I say different incidents, for instance, we sometimes went out to fields for service provision, or go to churches or move house to house. During this time, though most of the community members

have become beneficiaries of CBHI, we rarely come across households who are not yet become member of the CBHI. We sometimes find such households with their sick child and unable to visit health facilities as they have no money for the service that they simply wait hoping that the child will be well on their own. During this time, we inform these households that we can treat a child who contracted pneumonia with cough, diarrhea and other for free and advise them not to let their sick child stay at home.

Interviewer: Ok you mentioned that the incidents include church gathering and during house to house visit. What else?

Respondent: During meetings. For instance, there are weekly meetings by the kebele task forces that we usually attend, unless we have other duty like campaign, that we use this meeting to disseminate the information to the community leaders in the meeting about the service.

Interviewer: What type of meeting is that?

Respondent: Evaluation of services by different sectors that is conducted weekly with the kebele leaders that we use this meeting to pass the information about the service. In addition to this, there is also a monthly meeting among development groups, every 12th day of the month that we also use this meeting to communicate about the services. We use this meeting to inform the women development members that we provide the under 5 health services in the health post for free.

Interviewer: Are there any other ways that you use to do the awareness creation about the service to introduce the services to the community? For instance, use of posters and banners that are posted at a place that the community members can see?

Respondent: At the health post there is a poster, which you can see later, that shows the types of services that are being provided for free.

Interviewer: What information are indicated on the poster?

Respondent: The banner shows all the packages, which is not the child health specific but it is part of, that are being provided for free at the health post.

Interviewer: Are there any activities that you planned but did not accomplish regarding the awareness creation?

Respondent: No.

Interviewer: How successful were these activities that you conducted to raise the awareness about the service?

Respondent: There is a tendency by most of the households preferring to use health centers whenever their member gets sick, especially those households who are CBHI beneficiaries. This is because they receive the service for free as long as they are CBHI beneficiaries and our health post is in the health center and non-remote area that they can simply access the health center and rush for it. Only few households, whom we found during house to house visit, with sick child and few other who are non-beneficiaries and who have no money, receive the service from us. Except this the probability seeking our service for a sick child is low.

Interviewer: What are the reasons that they do not prefer to seek the service from the health post?

Respondent: Those households for whom we treat their children tell us that we treated their children well that it is not that they are not satisfied with our service. They also know that we provide similar treatment like the health center. But the health post is sometimes closed as we will not be found at the health post not for other reason but to conduct field services in the Gots/villages. Especially this year, two of us, HEWs of this health post were attending school that only one HEW was providing the service that she couldn't always open the health post. Due to these reasons our performance on child service provision is low.

Interviewer: As I can see that your health post is near to health center. Does this affect the service-seeking behavior of the community from this health post?

Respondent: Yes, it prohibits us to serve more children as the mothers tend to go to the health center because it is near but not due to a lack of satisfaction from our service. And even if the cases are beyond our level we refer them to the health center.

Interviewer: What are the differences in the services between the health post and the health center? Is that because the service at the health post is free?

Respondent: Not only because it is free that most of the beneficiaries prefer injectable. What I mean is, for instance when we ask mothers whether they get their children treated or not, they answer us by complaining that they were given drugs in oral solution or tablet while they were interested in injection. Most households think that injections will relief them faster than other methods for both adults and children. The households know that injections, the method that they mostly prefer, are not normally provided at health post. They even know that there are no medicines to be taken in the form of liquid or diluted form, with the exception of ORS, at the health post. They know that the medicines at the health post are mostly to be taken in tablet form, which are to be swallowed with clean water or breast milk. Other than that we tell them that there is gentamycin for children under 2. Due to this reason most households rush to health center seeking medicines in the form of injections.

Interviewer: Are there any support that you received from health centers or other projects to conduct these activities of awareness creation?

Respondent: If you are asking about the support in this year, the other HEW can provide you more information than me as I was at school this year. She told me that there were various supports from stakeholders. And she is here that you can ask her.

Interviewer: [Switched to the second HEW that was fully serving this year] Ok. In case you can add more, let me start my question with you from questions that I asked earlier. What are the activities that you have conducted to promote the service and raise the awareness of the community about the service?

Respondent: In order to raise the awareness of the community, I passed information about the service through influential leaders of the community, especially in the areas that I couldn't reach. In addition to this, whenever I go out and meet the community I will pass the message on the service that I tell the services are being provided at the health post for free.

Interviewer: Can you tell example on where you go out? Where are the places you go? On what way do you pass the information?

Respondent: For example, when I go out for immunization that we provide permanently per month and when I do house to house visit based services.

Interviewer: Can you tell the reasons why most households do not seek the service from you? If so, what did you do to change this attitude of the households?

Respondent: Regarding their attitude, they do not think that a child will be cured by the treatments at a health post and everybody knows for the presence of this attitude. The nearness of the health center is also the other pushing factor that they tend to go to the health center.

Interviewer: Why don't they think that a child will be cured by the treatments at a health post?

Respondent: In my opinion, it is due to the lack of trust on the services provided by the HEWs.

Interviewer: Why did they lack trust on the services provided by the HEWs?

Respondent: In fact, even in earlier days all the households usually seek such services from the health centers but not from health posts.

Interviewer: Why not from health post? Can you tell reasons other than lack of trust?

Respondent: No.

Interviewer: How do the influential leaders pass your messages?

Respondent: There is regular monthly meeting that they use this event to pass the message about the presence of the health service at the health post.

Interviewer: What supports did you receive from health center or a project for the activities you conducted related to the awareness creation? But before this, in addition to passing the information orally through events, have you ever used posters, fliers or banners for the awareness creation?

Respondent: No. We did not use such method. But, regarding the support, I received training recently and I have been receiving support before training from ato Zemed and even after training Ato zemed provided me strong support. Other than that the health center usually provides me support.

Interviewer: What are the types of support that you received? Please explain.

Respondent: By providing a case and showing on how to assign, treat after that by providing feedback on the gaps.

Interviewer: You are telling me about the support on treatment, which we will cover later in this discussion. But here I want you to tell me the support that you received on the awareness creation activities. Is there any support on the awareness creation activities?

Respondent: The health center has provided us the banner that we post here in the health post compound. Moreover, I use the materials of the training that the health center provided me to brief the mothers and community members about the service.

Interviewer: Who briefs to the mothers?

Respondent: Me.

Interviewer: What are the materials that you mentioned?

Respondent: For example, the MUAC measuring tool, the height measuring board and the weight measuring scales .

a. How is the engagement of communities/WDA networks in the implementation of integrated community-based case management (iCCM) of common childhood illnesses/possible serious bacterial infection (PSBI)?

Respondent: The WDA, which can't be said that it is functional, and as the health post was mostly closed this year, because the two HEWs that work with me were at school, the activity was very low. Regarding the WDAs, there are still few that help me in mobilizing pregnant women to come to the service but it can't be said WDAs are currently functional.

Interviewer: Why are these WDAs can't be said currently functional? What is the reason for not being functional?

Respondent: When they are asked why, they respond that they are simply wasting their time for nothing as they do not get any benefit out of it. AS the WDAs relate each of their activities to some benefit and as they are not getting any from being WDA, they say that we better drop it out. The wereda health office told them that there is no budget allocated for WDAs in any way and hearing this and considering the difficulty of the task they prefer dropping it out.

Interviewer: Was there any benefit that they used to get before?

Respondent: I don't know before I joined the service but after I joined there is no any benefit that they used to get.

Interviewer: What other reason, other than the benefit, do the WDAs mention for their dropout of the service?

Respondent: Some of the WDAs say that they don't have time but the main reason is not getting any benefit from the activity. We, as HEW, can't pay them anything but we have tried to tell them that it is a voluntary act that they should do without payment, which they don't want to listen and do not believe.

Interviewer: How do you rate the support from the WDAs?

Respondent: Not significant that even they hardly attend meetings after tedious provoking done to them.

Interviewer: How then did you fill the gap that occur due to the absence of support from the WDAs?

Respondent: By optimizing the support that I can get from the few WDAs that are willing to work voluntarily.

Interviewer: So, that means there are few WDAs that are still supporting you. How could you sustain their support?

Respondent: The health center has once provided them “Safa” [a bowel for washing cloths] as an incentive. But there are WDAs who leave after receiving the incentive and there are few that continued.

Interviewer: Was that a kind of reward for their accomplishment?

Respondent: It was intended to get their extended support for the future too by motivating them through incentive provision.

Interviewer: Did all WDAs receive the incentive?

Respondent: No it was provided for those who have been actively supporting.

Probe: What are some of the biggest challenges with SBCC activities for newborn care?.

Interviewer: What are some of the biggest challenges with SBCC activities for newborn care? Do you think that the awareness creation activity that you conducted was sufficient?

Respondent: I don’t think that the promotion and awareness creation activity conducted was sufficient as we did not use materials like banner and phone. The challenge that I faced is that, I have been working alone for 7 to 8 months in the kebele which is large in area with 7 churches. Even it was difficult to cover the immunization service. But now, as the two HEWs joined me I think we will perform better.

Interviewer: So, did this situation affect your awareness creation activity too?

Respondent: Yes, I believe that the activity that I conducted regarding the awareness creation is not enough.

Interviewer: What other challenges did you face that affected your awareness creation activity?

Respondent: The awareness raising activity needs a lot of work on the community that we should raise their awareness repeatedly to raise their awareness as the community hardly understand. And this can be achieved only through our effort.

Interviewer: What are the factors that made the community hard to understand? What do think you can be done in the future to resolve this problem of understanding?

Respondent: just lack of awareness.

Interviewer: What type or problems do exist? Are there any misconceptions that you heard about?

Respondent: The community members say that taking children for medication, at least before they are baptized, is immoral, and against religion and hence, they prefer to seek traditional medicine, which they use some ointments that they believe prevent or cure the child from disease. Mothers usually believe that all children may get sick unless they use this traditional treatment.

Interviewer: What did you do to make them change this attitude of the mothers?

Respondent: In this village, I come across a woman who use the traditional ointment for her child that she even refused to show her sick child for me. Then I, with some other elderlies went her home and advised her to bring the child to the health post for treatment that later the child was cured after treatment at the health post and in fact, she used both the modern and traditional treatments.

Interviewer: What did you do to prevent such misconceptions with other community members?

Respondent: I advised her and other to seek health services at health facilities rather than the traditional one.

25. Could How do you describe the strength of the support you got from the PHC and the project as well as your efforts to address barriers to delivering iCCM/PSBI during COVID-19?
    Probe: What was changed?

Respondent: In general, I developed confidence on my capacity to treat children by myself. I took training and after that I was sure that I am able to handle the treatment of sick children by myself and they supported me for this.

Interviewer: What are the changes that you brought about due to the support that you received?

Respondent: Earlier, as I was new, I was not able to treat a child by myself that I used to tell mother to go to health center. Here, I am telling you my experience but after the training and the support I become able to provide the service.

Interviewer: What additional thing did you get that you were not able to do before and become able to do after the support?

Respondent: Became sure that I am able to treat sick children.

Interviewer: How about identification and referring?

Respondent: I won't face difficulty on this too.

Interviewer: Is this the skill that you acquire after support?

Respondent: Yes. In fact, the skill that you acquire in class training is different from on practice that the skill through practice and the support you get during practice will make you better.

Interviewer: Please tell me the support that you received in detail. What are the types of support you received from the health center or others?

Respondent: As I told you, after the training all the HEWs were gathered at the health center. They provide us with case scenarios for each of us and they verify our work on the case based on the chart that they identify the ones who did right and who did not. Based on this they fill the gaps that we have. Other than that I received direction to strengthen the WDA, which was being fading out, and out of the 42 we tried to redeploy some of them.

Interviewer: How did you manage the redeployments of the WDAs?

Respondent: I conduct house to house visit to communicate the existing WDAs, using the list at the health post that I gave them calling letter, which is prepared by the health center, for a meeting that some of them have come to the meeting.

Interviewer: What other supports, including the PSBI and on ICCM, have you been receiving and how often were they providing you the support?

Respondent: Though I can't recall the frequency, there are times that he came twice per week, in which he visited all the health posts. Other than that he has been supporting us a lot of times.

Interviewer: What does he do during his visit?

Respondent: He asks us for the challenges we face and he provides the necessary supports needed. He observes our treatment and identify our gaps during service provision and provide feedback on the rights and wrongs. It is like on job training that helps me to stand alone on my own.

Interviewer: How about support from health center? What are the types of support that it provided you?

Respondent: Especially, currently, it can be said that the health centers are working together with us. For instance, they are working on the eCHIS with moving in the community with us, that is to record all the households which have been communicated to be completed up to 15th of this month. Especially this week they are working on house to house visiting to complete listing of the household heads by accompanying us all the time.

Interviewer: What other support did you receive that it can be a kind of review and evaluation or coaching or guiding during real time implementation?

Respondent: Yes, they also do that. At least they will inquire for the reason of low performance and after they identify the reason they will suggest some method that can improve our performance. Every month, after we submit our report there is evaluation that they review our report to identify our gaps and provide feedback that can fill the gap.

Interviewer: How do you evaluate the support? Was that helpful? If so how do you describe its importance?

Respondent: Yes, I consider it as important and helpful.

Interviewer: Do you have suggestion that can improve the support that which was supposed to be done to significantly improve your skill and service provision?

Respondent: As I told you earlier it would be helpful if banner is prepared and used for promotion that it should be strengthened.

Interviewer: How about regarding inputs? What are the supports you received to be sufficiently supplied with inputs?

Respondent: Regarding input, most of reagents needed were stocked out for a while but later Ato Zemed provided us that all the health posts received the reagents from him and filled the gap.

Interviewer: What else, what was the way that you regularly get the inputs?

Respondent: There was no significant problem regarding supplies that we used to receive upon request.

Interviewer: How often do you request for the supplies?

Respondent: Monthly and if we run out we request bimonthly.

Interviewer: So, regarding supplies, you have nothing that you suggest to be improved. is that right?

Respondent: The support should continue to improve the service.

Interviewer: Are there times that you couldn't be able to receive sufficient support?

Respondent: Sometimes there is less and other times there is much support that I suggest it to be uniform throughout the times so that we can improve our performance.

Interviewer; How about now? How is the supply provision?

Respondent: Currently it is good.

Interviewer: How about on iCCM? When was the last time you received support on iCCM?

Respondent: They visited me in recent days that they observed my activity of treatment and commented that I am doing well.

Probe: Was the support you got from the project and PHC helpful? What could have been done differently?

26. Do you think the support system is helpful (mentorship and on-site coaching methods, supervision, technical support, PHCU level PRCMM, etc.) to enhance your skills [that is HEWs' skills] over the traditional off-site training and woreda level review meetings?

Probe: for acceptability of the strategies **(for HEWs only)**. How could the support system be improved?

Respondent: I consider it as good and I took training once that earlier only experienced HEWs were supporting us.

Interviewer: Why did you consider it as good? In what aspect did you consider it as good?

Respondent: The support helped me identify my gaps and fill it that I now become confident in my treatment of children. Other than this, the review meeting that we conduct improved my skill because I receive feedbacks on my gaps.

Interviewer: Which support, the training or the mentoring on the job, contributed more for your improvement on treating children?

Respondent: Both were helpful but the on job mentoring or support was more helpful as it bases my gaps based on my actual activity.

Interviewer: Which one, the training or the mentoring on the job, was more receptive?

Respondent: Both.

Interviewer: Is there anything that should be improved?

Respondent: No.

27. How eCHIS implementation helps you with iCCM service delivery?

Probe for advantages:

- case identification? quality of iCCM case management? retrieval of client records? data quality? client appointment scheduling and/or defaulter tracing?
- **Ask why?**
- **Probe for areas of strengthening?**

Respondent: With the phone there is the treatment chart but we all are not using it for the treatment but for immunization, FP, ANC and PNC.

Interviewer: What is the benefit of using eCHIS?

Respondent: Regarding use of eCHIS, I don't see any change from the previous but first this system is time taking. When you fill the data to the system, while the mother is sitting and waiting for the service, there is a delay which is created due to server problem. Other than that, the system may become better after completely entering all the data for all services that we may completely use the system by dropping the hardcopy method.

Interviewer: Didn't you completely enter all the data to the system yet?

Respondent: There is few left.

Interviewer: You mentioned that there is a delay during data imputation. Why is that?

Respondent: I think it is due to the weakness of the network and connection that the data are not transferred faster. When you fill the questions in the tablet the filled data does not easily transferred to the server.

Interviewer: What are the strengths, other than the mentioned weakness, do you observe with the system? For example, with respect to case identification, case management, data quality or other?

Respondent: Regarding data quality, yes it ensures better data quality as long as you properly enter data and the data will be well documented and secured.

Interviewer: How about in finding lost clients? Can you tell me more on how the system is helpful, with other aspects too? Please tell me just like telling to someone that doesn't know about the system.

Respondent: once we start to enter information on pregnant woman, it will notify us the consequent appointments by shading some color, which is so helpful not to forget the appointment. It notifies us the date of appointment that we use that notification to find the pregnant woman on the day, which is good. The same is true for the appointments of immunization that it notifies us for the date of specific immunization for a specific child.

Interviewer: But, you are telling me that you are not implementing the system for iCCM. Is that right?

Respondent: Yes, I did not yet use the system for treatment of children that I am using it for other services.

Interviewer: What are the services that you are using the system for?

Respondent: FP, ANC, Immunization and a lot more.

Interviewer; How about delivery?

Respondent: Yes, it also holds delivery that the health center will send us the information for a woman that delivered immediately after delivery and we use this for the subsequent follow up /PNC and immunization/ service.

Interviewer: Is there any way that you can use the system for the iCCM service delivery?

Respondent: It is possible but I did not yet use the system for iCCM.

Interviewer: How is it possible?

Respondent: If a child comes for treatment, rather than the hard copy that I currently use, I can use the tablet to enter information in the tablet.

Interviewer: Based on the gaps that you observed with eCHIS, is there anything that should be strengthened or improved regarding the eCHIS? What should be done in the future to improve the utilization of the system?

Respondent: I have no that much different idea that it is ok as it is now that it should continue as it is.

Interviewer: For instance, you told me that there is a delay during data entry, do you have something to suggest on this?

Respondent: I think this is due to the location of the area that we are using.

28. How was the introduction of the iCCM/CBNC module into eCHIS affected the iCCM/CBNC service delivery?
FOR LUME WOREDA ONLY extra

IX. Implementation challenges

29. Describe the main issues faced by the health system to identify and treat neonatal infections in the community. What are the critical factors affecting the delivery of iCCM to clients?
Probe:
    - lack of competence of HEWs, shortage of supplies and commodities; weak support system; low community demand?

Respondent: The main problem is the geographic condition of our area, for instance Chuye Got, that it is difficult for a woman to easily bring her child to the service that she is discouraged to utilize the service.

Interviewer: Is that due to distance or the difficulty of geographic condition?

Respondent: It due to both that the distance and the difficulty of the geographic condition.

Interviewer; What can be done to resolve this problem? How about the location of the health post? Is it not at the center?

Respondent: No, it is not at the center that I don't think the health post is located at a nucleus location.

Interviewer: What else? What challenges, other than the geographic condition, did you face this year?

Respondent: There is no other challenge.

Interviewer: How about challenges regarding support?

Respondent: As I told you earlier the support should be uniform throughout. Mostly, the support is provided at the end of budget year that it should be improved and the support should be provided regularly and consistently throughout the year.

Interviewer: Which support, from project or health center, is being conducted irregularly?

Respondent: It is from all but the support from the health center is relatively better as it is near that they are mostly accessible for support. The support from the project is rare and irregular that they should make it more frequent and regular.

Interviewer: How about challenges related to outreach services? Is there a problem of supplies and resources?

Probe:

- Regional/national state of emergency and conflict in the northern part of the country?

Respondent: The conflict has hindered us to meet and work together with the community leaders. The weekly meeting that was being conducted among command posts with men and adults was interrupted as most of them have responded to the "Hilwna Zemecha" (War for existence) call by the government that most men have joined the battle. During the conflict, some community members were also

discouraging normal activities, like polio immunization campaign, saying “There is no use doing such activities while our country is collapsing”.

Interviewer: Who were discouraging and saying this?

Respondent: It is the community that for instance, when we were administering polio immunization campaign they were not interested to immunize their children just by relating it with the conflict.

Interviewer: How about for children treatment?

Respondent: Regarding the treatment, there was no that much problem as the conflict was not happening in our area.

Interviewer: How about the state of emergency? Did it affect the iCCCM?

Respondent: The ANC and PNC service was affected that some mothers were not properly attending the ANC and PNC, which implies the same for iCCCM.

Interviewer; Does the restriction hinder mothers to come to the service or you to provide outreach service?

Respondent: No, it did not in our kebele.

How has that changed during COVID?

How do you think the COVID-19 pandemic and/or COVID-19 response measures affect the uptake and/or delivery of iCCM services?

Probe:

- Has COVID-19 affected your daily routines; your work on newborns; the community in terms of livelihood and vulnerability for newborn care-seeking
- How has that changed over time?

Respondent: The COVID-19 vaccination administration was difficult as there were few community members who contracted COVID-19 among the vaccinated ones that most community members were keeping themselves away from health services.

Interviewer: Here I want you to tell me the impact of COVID-19 outbreak on the ICCCM service provision. How does the occurrence of the outbreak affect the service?

Respondent: There was no interruption of the iCCCM uptake due to the outbreak but it is after COVID-19 vaccination that the community members started to stay away from any other service including the COVID-19 vaccination. The reason is the fear of contracting COVID-19 as few members of the community contracted COVID-19 after receiving vaccination of COVID-19.

Interviewer: How about you, the service providers? Did the outbreak hinder you to move to the community and provide your day to day services to the community?

Respondent: In fact, there was a fear among us as few members of the community contracted COVID-19 after receiving vaccination of COVID-19 that we were afraid that the community will be against us due to the incidence. We were afraid that the community members will consider us the reason for the contracting of the virus of the few members that we vaccinated for COVI-19. But, though we were

afraid of such happenings, there was no such reaction from the community that they forgot the condition shortly.

Interviewer: So you were not moving to the community fearing that they will react for the situation?

Respondent: What I mean is there was only feeling of fear within us due to the incidence but I am not saying that we stopped or hindered our normal activities.

Interviewer: How was the reaction of the community just at the start of the outbreak?

Respondent: there was no that much influence other than being told to wear facemasks.

X. Adoption and reach/effectiveness

30. Explain to us any changes you observed as the result of the implementation of the COVID-19 adaptive iCCM implementation strategies? – What impact have the strategies had?

Respondent: The direction from higher official which forced us to provide the child treatment while we are conducting any other house to house based services was helpful to introduce the service. This direction of provision of the service while we are conducting house to house based services later helped mothers to seek the service at the health post that they are currently seeking the service here at the health post.

- What are the particular features of the strategies that made a difference?

Respondent: It was due to the awareness created about the provision of the service at health facility including the health post and the health center and the improvement of my skill due to the support that I have been receiving.

Probe:

- for a support system and linkages, motivation, and competence of HEWs, community, and awareness creation?

Respondent: Yes, providing the service at health facility was helpful. For instance, while I was providing the service on the household to household basis, carrying the materials needed for the service was challenging. Due to this, unlike the full treatment that I am now conducting accessing all the necessary materials here at HP, the treatment that I was doing in the house to house basis was not complete as I was not carrying all the necessary materials during the visit.

Interviewer; How about the linkage? Related to earlier, is there any improvement observed?

Respondent: At least when I send mothers after filling the referral form, the mothers are treated properly without facing challenge at the health center. Due to this, I think our linkage is improved.

What do you think are the reasons for non-significant changes?

Respondent: Currently the magnitude or number of heath care services at the health center and at the health post is not the same, which is because the tendency of the users to be served by the health center rather than the health post. Most service seekers directly go to the health center by bypassing the health post because the health center and health post are near each other. Thus, we have to strengthen

our service provision so that at least those children that can be treated at health post can be treated at health post rather than passing us and get treated at the health center.

XI. Maintenance and sustainability

31. What are the high-level benefits that are attributable to this support/IR?

Respondent: What I put first is the treatment of children. Other than this, the skill of communication and working with people is second.

32. Please explain to us the feasibility of this support/ IR for national scale-up?
    Probe:
    - What features could easily be integrated into the existing system? Which not?

Respondent: As the activities are a lot, I will be happy if I could not continue all.

Interviewer: Here it is not about being happy or not happy to continue. What I mean is, sometimes there are some activities started with a help of a project or a support but could not be sustained after the support or project is stopped and there are some activities that can be sustained. So, in your case related to iCCCM, are there any activities that you started with the support and that you can integrate and sustain even if the support is stopped.

Respondent: The treatment of children will continue that it should not be stopped. Moreover, the use of eCHIS and use of the tablet will also continue.

Interviewers: Are there condition that can enable you sustain the child treatment activity? If so what are the conditions that can enable you sustain the service? Can you proceed providing the child health service on your own? How are you going to sustain it?

Respondent: I can request and receive the necessary supplies from the health center that can help me provide the service.

Interviewer: What other things do you need to sustain the service, like for identification, support from the community, and others?

Respondent: As I mentioned to you earlier, if the banner is prepared and used for promotion, with a help of stakeholders, it would be helpful for improving the utilization of the service.

Interviewer: Other than the promotion, can you access every supply needed for the service like the charts and other testing materials?

Respondent: Yes, I have the materials needed for the service like a thermometer and others.

33. How are the activities/efforts embedded in the PHC and woreda routines/work streams?
    - What implementation strategies are incorporated with the PHC and woreda annual work plan?

Respondent: What we do to integrate the service is, we always use a detached page of the main iCCM register and the chart booklet, and record activities using pencil and erasing errors that occurs when we are in the community.

Interviewer: Do you usually do this identification?

Respondent: Yes.

Interviewer: Did you incorporate it in your plan?

Respondent: Yes.

Interviewer: How do you include it in your plan? Is it specific to newborn health and did you include it at HP level?

Respondent: you can see it posted there.

34. Do you have anything think is important to tell us that we have not asked you?

Interviewer: Do you have anything that you would like to add at the end?

Respondent: That is enough.

THE END

End line Evaluation of the PSBI implementation research; Full Transcription for an In-Depth Interview with a Health Center Head

Questionnaire ID	03
Area Identification	Dembecha
Name of Woreda/Zone/Region	Dembecha WoHO/West Gojjam Zone/Amhara Region
Name of facility	Health Center
Name of moderator	
Name of a note taker	
Date of Interview	17/06/2022
Participant #	01
Audio File #	Health Center_Head_17.6.2022
Start time:	10:30 AM
End time:	12:19 AM
Transcriber/Translator	
Duration of IDI:	1hr, 49 minutes

I. Fidelity

Can you please describe the demand generation/SBCC activities you are doing on iCCM/ newborn health? What are the strategies used to raise awareness? If none, please explain why. **Probe:** What are some of the biggest challenges with SBCC activities for newborn care?

Interviewer: ok thank you. My first question is about the demand generation and SBCC activities you implemented to create awareness, in order for the community to know about the iCCM services provided at the HPs? What are some of the activities you implemented?

Respondent: ok thank you. When we see the awareness creation activities, it was last year that we started to fully implement them. Beginning from the month of August 2013, we prepared a banner listing the type of services at the HP level and read them out loud at meetings, in order to help all, who read and don't read understand about it. We aware them of the iCCM/CBNC services being provided at the HPs and that they don't have to suffer coming to us at the HC, so we reached an agreement with the community about this. We make the awareness creation through the kebele leaders' support, gathering the community for us, and now if you go to each HP the type of services is listed, and it is also informed that there will be a referral if it becomes beyond the HP capacity.

Interviewer: Ok so you implemented this using a banner but you used the kebele leaders?

Respondent: generally, all the leaders, and also it was in the presence of the religious leaders, influential community figures, and WDAs that the awareness creation was made. This is because we might not include or all invited people might not come, so by making awareness creation for these people, it will surge and reach the rest of the community. We also verify the delivery of the information when we go for the house-to-house visit asking them to mention the services provided at the HPs. So this is the extent to which we have been implementing it.

Interviewer: how effective were you in terms of the effort you exerted to aware the community of the services?

Respondent: there might be some gap despite the effort. Most had awareness but they didn't have trust, and at first they were taking most of the newborns to traditional medicine. This is because we were observing them doing that. It is after that they come to us. When they are first sick they don't bring them to us or the HP, it is after that they bring them to us. It had a bit change from the previous and there are somethings remaining still, this is because when we still visit some mothers we notice that they are still visiting the traditional healers. Many children used to die because of this but now we manage to stop that, and there are no more deaths now. When we compare it with the previous time, there is much improvement. We also want to thank the organization [JSI], they helped us a lot in terms of providing training for our professionals. For instance, there were health professionals who does not know how to provide the treatment at the HPs, and in consultation with Gash Abebe [the JSI staff, name changed], we trained all the HEWs in the five health posts. Now if there is a need to treat, they won't be biased and can provide the service. **6:27**

Interviewer: you told me there is change but there are still somethings remaining. What is that you think is not done from your side or the reason for the community still seeking the traditional medicine?

Respondent: well, this is even a challenge at the woreda level. I also think if we can merge the modern and traditional medicine together. For instance, there is sometimes swelling and bursting in the beast areas of the newborns which science says it subsides on its own, but they say traditional medicine cures it. They have the awareness about where this service is provided but they relate it with the spiritual aspect. But when we advise them properly, they tend to come and change when they see other neonates cured. As an example, there was a newborn who we came a cross when we were visiting the village, and there was a depression on his fontanel and inflamed which clinically means he developed sepsis. When we reach at the place, we observed she had put some herbs on his head, and she told us the reason relating it with spiritual reasons, and then we told her that it can be healed through medication, she then mentioned they told us about it. so she had the awareness but she didn't understood it well. But when she bringsw the newborn and we put him on gentamycin she saw the changes and even vowed to inform others to bring their children. There is a traditional healer at Yechereka who is challenging us very much, and the community here also go there in mass,

I even raised the issue in a meeting if we can bring this woman to the modern medicine linking here with institutes like the Universities.

Interviewer: But have you noticed the community are getting cure from her, from the traditional healer?

Respondent: they say they are getting cure but there are diseases that are not cured

Interviewer: what are some of the activities you did on the community to avoid their dependency on the traditional medicine?

Respondent: we have provided education for the community at churches but I don't think this is adequate as well. A lot is expected. Since there was a focus on the conflict and since we had also professional we send out to the war front, hence we are only treating those seeking treatment here. We can't say the education we provided is also adequate, there is a still a lot more to do, and it will be something we will strengthen in the future.

Interviewer: is there anything that you say is not implemented in the SBCC activities? Or challenges?

Respondent: the challenges in the SBCC activity are some of the places we didn't address due to the difficult geographical location; you might go to these places but it is difficult to return. for instance there was as an area called Chube which we didn't address and other places. So we have some gap in the outreach service which we want to address in the future.

Interviewer: what is it that you need to address these areas?

Respondent: as I told you, our attention was on the war and we didn't give much attention for the community activity. We also have shortage of health workers, for instance we were very challenged when the HEWs were away for education reason. Since there were such challenges it was difficult for us, but now everyone is available and it will be possible to implement everything in the future. The other challenge we have is the supply shortage, for instance, there is no gentamycin to treat sepsis, especially the 20 mg, so most come to the HC since it is not available at the HP. It is mostly the 20 mg that is needed to be availed at the HP since there might be a skill challenge to make the proper dilution for the other types. There is also a shortage in the dispersible Amoxicillin, and ORS-Zinc is also shortage.

a. How is the engagement of communities/WDA networks in the implementation of integrated community-based case management (iCCM) of common childhood illnesses/possible serious bacterial infection (PSBI)?

Respondent: the communities' contribution is good but normally they are not helping much due to the current situation in the country. For instance, if someone is sick in their area they will give us information, the tendency of the community to provide information when there are outbreaks has grown like when there is illness among children, etc. so it is good in terms of providing information.

Interviewer: How about the contribution of the WDAs to the iCCM service delivery? What was their contribution?

Respondent: it is beginning from the mother's pregnancy that the WDAs start the follow-up. They have a meeting every month and when they come for that meeting, they bring information about sick people in their community—which children are sick, which mothers are sick, etc. The HEWs also ask the WDAs, and then if there is a delivery or sick newborns in the community the WDAs provide that information, and accordingly, the HEW will make the assessment based on this information.

Interviewer: What is the current functionality of the WDAs, is it to the extent that you have just mentioned that they are engaged? How is it?

Respondent: they are not fully functional. At Daba catchment, out of the total WDAs only 20-30% of them are fully engaged, which applies to all those I mentioned earlier. But the rest are not engaged, and they have been in the activity for too long and not been replaced as per the recommendation every five years but they have worked for more than this, and because of this, they are not that engaged. But we tried to improve this from our efforts, and held a discussion with them. They have related it with benefit issues, they told us they have been giving everything for the work, but let alone an incentive, we have not been even recognized in words, and they mentioned that would have been enough. In respect to that, we have bought all our WDAs a bowl to serve them wash their clothes, and after we gave them the bowl they have shown an improvement and they have started their regular meeting in the form of "EKUB" *[a form of social association where they meet at a given time interval for collecting money from the members, draw a lottery and provide the winner for the collected money, and waits to do the same in the next round until all members are drawn].*

Interviewer: Is there any other additional association other than the WDAs?

Respondent: there is something we started in recent time, but it is not fully started which is called "village health leaders", we have already selected the members but we are waiting for budget and preparing to provide training to commence the activity. If this does not work we also use the Men Development Armies especially when there are campaigns it is them that we use—they are normally established by the administration and we use them integrating with the WDAs.

Interviewer: Do you use them for the iCCM service delivery?

Respondent: Men are even better to use for this service since they possess cell phones; we call them and ask if there are any deliveries or sick newborns in the community. Even if they are small in number we use them especially for those areas remotely located. Once they call and informed us about the occurrence of scabies outbreak in the community which we then went to the site and intervened.

35. Could How do you describe the strength of the support you got from the PHC and the project as well as your efforts to address barriers to delivering iCCM/PSBI during COVID-19?
    Probe: What was changed?

    Probe: Was the support you got from the project and PHC helpful? What could have been done differently?

Interviewer: How do you describe the strength of the support you got both from the project and your support to the HEW you implemented with the project?

Respondent: well the organization supported us a lot. Gash Abebe [JSI staff] makes the maximum support, I want to thank him and he used to call even in the weekends, and there was a condition where we support all the HPs in person. There is an integrated supportive supervision every week provided for the HPs, not just on the iCCM service but integrated. A supporter goes to the HPs and even to the villages to see how they have performed. and there was also neonatal care-specific support conducted every two months using a checklist Gash Abebe gave us to evaluate how they are providing the treatment for the newborns including the diagnosis and support provided on spot on the corrective measures. We discussed with the supporters about the significance of the support and activity because of the alarming neonatal death rate in the country, and then we started exchanging of weekly reports on iCCM—but now it is not being implemented after Gash Abebe stopped coming like before, and we ask questions when we encounter deaths of neonates, we also ask how much they treated and we also see the newborn in person through a random visit for ensuring the provision of the service. You have also slowed down with your support, we can do everything on ourselves but sometimes we need support for awakening, this has been the norm as humans.

Interviewer: Has there been any other support from you side and the project regarding the iCCM service?

Respondent: sometimes there may be some member of the community who might refuse and become hesitant to receive the services, and in this case, there is a trend to go to the community and provide awareness creation for this group of community. We also investigate the reasons behind and try to sort out the rumors.

Interviewer: How about training and other supports?

Respondent: regarding the training, the project trained all the HEWs in terms of identifying the type of disease, for instance, when we even evaluate among us we can see that they become competent and doing it neatly.

Interviewer: How about the off site evaluation here, and designing tailored onsite support etc? are there some kind of support for the HEWs?

Respondent: Very well. Regarding that, it is also good, especially on the evaluation of the activities, we are mainly focused on mothers and newborns even before that, the HEWs come every month with their reports and we make the most evaluation on mothers and child care; why she didn't do it, and if zero we ask if it is really that because newborns are scarce, and we even go down to the community and check for the validity of the report. When they also come here, they come with their charts and then we give them a case, and then describe an ideal newborn coming with a specific case, and then has a specific sign and symptom, and then how would she identify the disease and how she treats it. and if

she manifests a specific gap, then we provide her support and even go down to the HP to make onsite support.

Interviewer: what are some of the changes that you have got from making these supports?

Respondent: we have got many changes, the first is normally there were those who remains in their house and eventually dying, and we managed to stop that. The second is, there was a lot of suffering from different things like transportation and distance when they were coming to us, they used to think only vaccination is provided at the HPs and didn't know about the health care service, so after we told them the service availability at the HP, they stopped coming. Hence, it implies that neonatal death has decreased and addressed those newborns which we could not reach. So these must be strengthened and I think you as a project must give the responsibility to the government to sustain since it is a project's nature to end at some point. So now the community has got the awareness and know about the service, and from the HEWs perspective, the support makes them gain confidence to provide the service; there is rhe chart booklet and they are treating now. But before the training they don't consider themselves as a professional.

Interviewer: Is it only the training or the other supports you made that boosted their confidence?

Respondent: the training is the main thing, but after the training all might not have equal understating, so we provide them routine supportive supervision and told them people need not suffer with transport and others, and that they need to provide the service. If they encountered any difficulty they communicate with us through phone and we immediately go there in person and provide them support. We also encourage and coach them using ac al cases in the community. So it is both our support and the training that contributed for their change.

Interviewer: What else do you have to tell that changed due to the support?

Respondent: the awareness level of the community has also increased after the time the project supported us. those who can read the banners also get amazed that the listed services are being provided at the HPs.

36. Do you think the support system is helpful (mentorship and on-site coaching methods, supervision, technical support, PHCU level PRCMM, etc.) to enhance your skills [that is HEWs' skills] over the traditional off-site training and woreda level review meetings?

Probe: for acceptability of the strategies **(for HEWs only)**. How could the support system be improved?

Interviewer: Now let me ask you about the support, that is the onsite support, mentorship you provided, in enhancing skill of the HEWs, especially compared with the conventional offsite traninig and others?

Respondent: I think the onsite support is better than the offsite training because there is no fear since they know us. We also discuss the actual case; for instance, when they come here, we take them in the under 5 OPD and encourage them to treat, diagnose, etc. If they were working with new faces, they would even forget what they know. Hence, coupled with the training, the onsite mentorship has helped

them to become more competent. As I mentioned, we also try to improve their competency using case scenarios.

Interviewer: I am also referring to the importance of supportive supervision and other supports for skill improvement?

Respondent: regarding the supportive supervision, we do it every quarter, but the mentorship is implemented after identifying their gaps and those HPs and HEWs with the gap, and try to make tailored support. So this has improved their competence level.

Interviewer: what skill improvement have you observed because of these supports?

Respondent: le us start from the awareness level; previously they don't have the intention of providing the service and after the support they started providing it. The competence level among the HEWs was also not equal so we made them exchange experiences, and they also had gaps in properly utilizing the chart booklet; if a child comes coughing, they would just give him Amoxa. But after the training, and our follow-up and support, they are now properly diagnosing, and asking every detail for the complains, and properly using the chart booklet. There was also some knowledge gap especially those newly assigned had no idea about the iCCM service provision but because of the support they are now in a better condition. Previously there was fear to administer injection especially Genta, but now they had no challenge, after the support, and all the 11 HEWs can now administer the injection.

Interviewer: what was the acceptability of the support by the HEWs?

Respondent: they have no problem accepting the support or to the support system, but they might raise their challenges like the religious reasons from the community

Interviewer: Is there anything that you if the support could have been provided in a different way?

Respondent: what I think would have good is, well the provision of the training for the HEWs is good but it is better if a review meeting or similar kind could be arranged to refresh the HEWs, and if such things are arranged I think the motivation of people to engage in the activities will also improve. With regard to improving skill, well science is dynamic and hence if there are updating of the staffs when there are such things, not just through in the form of training but it could be in a different way. Other than this, it is also good if there is a strong follow up including from your side since it is slowing down a bit now.

37. How eCHIS implementation helps you with iCCM service delivery?

Probe for advantages:

- case identification? quality of iCCM case management? retrieval of client records? data quality? client appointment scheduling and/or defaulter tracing?
- **Ask why?**

Interviewer: How does the eCHIS implementation helped you with the iCCM service delivery?

Respondent: regarding that, well it is good a point you raised. Normally we had a problem as a woreda regarding the eCHIS implementation, and we have been receiving a lot of blame on that. This is because most

people are bored; going to each household and record every child born is boring. However, eCHIS with all its problem is very helpful for the neonatal health care service delivery. For instance, any child born is registered in the system and the mother will also be given a PNC, then you start the newborn's status after he is registered; if it is about the pregnant mother, it makes you forecast the date of delivery and you make your visit on the specific date, and if it is the newborn and you registered him, then it indicates you the day he is supposed to take a vaccination. So it helped us a lot for such things. The other is, when you go there looking for the register, you also get that child—we make updates every year, and when you go looking for that, you can also make updates if there are additional children, and you can also get the chance to record challenges if encountered. So the introduction of eCHIS has been helpful for us, we were also recording all households to bring it all into the eCHIS which will make it easy for us; to know how many children we have, those sick ones, and to also enquire the WDAs as "there is this number of children in your village, are there any who is sick?". We have shown a good recording in the eCHIS in the last two months and the HEWs have also provided treatment to neonates using that.

Interviewer: does it have an advantage in the case identification?

Respondent: No it has not started yet. it is not in the software yet, and the management has not been started yet such as the indication to provide this or that for the treatment has not started. But I think there is a suggestion to change the software, maybe we will have a chance then.

Interviewer: How about easily accessing client records?

Respondent: A card number is not recorded and if this was done, it would have been helpful. What it is helping with now is in determining the population number and the number of service users. MRN is not being recorded and if this was recorded it would have been helpful. This is also being implemented at the HP only and there is no linkage with the health center, but if there was linkage it would have been easy to do that. It is only the activities that are being implemented at HP sank to the HC through the focal at the HC but for the MRN, they are just using their own code to identify them in the eCHIS at the HP. But here there is a different client code given in the individual folder, so the eCHIS is not implemented at the HC and is done only at the HP. Client data retrieval can easily be made at the HP using their specific code in the eCHIS—it will give the person when you feed the specific code.

Interviewer: How about the eCHIS advantage in ensuring data quality?

Respondent: it is very helpful. Previously it is all flooding of numbers, for instance, they used to report which they didn't do or missing to report something implemented that is under and over reporting. But now they just say sink for activity they did and the focal person then receives the report, and also follows their progress of the HEWs and their implementation. We also ask them why when they are sending an exaggerated report, and because of this it is changing everyone to make a valid report.

Interviewer: How about with regard to making a schedule or following defaulters etc?

Respondent: It flags red light, for instance when a child vaccinated now is not vaccinated again in the next schedule, it will flag red. We also follow the implementation for each service from the server when they sink it, and if she didn't provide for instance ANC, EPI, we will follow her if she didn't do it on the schedule and it also flags red. It also has an action card section found in the eCHIS, putting the un implemented activity if she didn't not do it. so we inform her to visit those people she didn't manage and she will be reminded.

Interviewer: is it possible to implement and have this advantages if the iCCM services is integrated in the eCHIS?

Respondent: yes it is possible to implement. Because is the application is installed there will be anything challenging, it will be much easier. Currently how they are implementing the iCCM service when they are making the house to house visit is by having two detached pages of the main register. But if it was installed, it will be easily implemented and conducive to carrying it around through the tablet. So it is better to provide health care if it is integrated into the eCHIS.

Interviewer: How do you see the advantage in the case management and quality?

Respondent: the management and providing the treatment is done on spot. When a HEW goes out for a house-to-house house visit, they bring along all those required things with them within their bag.

Interviewer: so would it also important to ensure the data quality and retrieve the data records just like you mentioned for the others, if the iCCM service were to be integrated in the eCHIS?

Respondent: yes it would. If it is integrated in the system.

Interviewer: For instance, is there anything that you are challenged from using the manual CHIS, that you think would be improved if it is integrated in the eCHIS?

Respondent: Yes, for instance, at one point they were sending a report as a newborn being treated for sepsis, which they didn't do. But if we were using this system, we could easily detect and control it. There was case we verified not treated but sent to us in the weekly report by the HEWs. This is mainly resulted from recording in personal note pad of the daily activities by HEWs and not in the register, but if they were able to record it in the eCHIS, it would have alleviated such data quality issues. She might also start a treatment for a newborn but would not complete and it would enable us to follow those things if it is integrated in the eCHIS.

- **Probe for areas of strengthening?**

Interviewer: what are some of the things that you suggest should be strengthen about the eCHIS from your implementation so far, as well as other improvements.

Respondent: one thing I think should be improved to strengthen the eCHIS, one is the tablet sometimes makes a difficulty in sinking and problem with the server. And sometimes it gets stuck and mal-functions. Most tablets get stuck and as a result we have 3 to 4 tablets which are out of use and stored. The other challenge, we have serious shortage of electric power supply. We of course a power bank but

could not charge it since we have no power for a period. Hence if we can get support for solar power instead. We have been challenged the eCHIS campaign to register all households because of electric power supply shortage. If we also get additional power bank at least we can get power for two or three weeks.

38. How was the introduction of the iCCM/CBNC module into eCHIS affected the iCCM/CBNC service delivery?
FOR LUME WOREDA ONLY extra

II. Implementation challenges

39. Describe the main issues faced by the health system to identify and treat neonatal infections in the community. What are the critical factors affecting the delivery of iCCM to clients?

Probe:

- lack of competence of HEWs, shortage of supplies and commodities; weak support system; low community demand?

Respondent: we have many challenges and I mentioned some earlier. One of our biggest challenges currently is the skill gap filling, since we are also having newly assigned HEWs. So we might be expected to provide a kind of training both from our and your side. But with regard to the policy aspect, I think there is a gap from the government side, and I sometimes wonder how long should we have to depend on partner support. I have this thought, why is the government not having a specific design of policy on this and start implementing it. The other big challenge regarding neonatal care is the supply shortage. We have made a request but we could not have the required supplies for the iCCM service for children. The government should give due attention specially for child care services, and if it arranges favorable conditions to get these supplies for us. The other is in terms of the HPs structure itself, and the lack of standard rooms to provide the services, if you have seen our under 5 OPD, it has a very small working space which is difficult to provide the required activities such as to measure weigh and heights, and as a treatment center we also don't adequate room to provide the service.

Interviewer: what else?

Respondent: I have no more.

Probe:

- Regional/national state of emergency and conflict in the northern part of the country?
- How has that changed during COVID?

Interviewer: How about the conflict in the northern part of the region, how did it affect the iCCM service delivery?

Respondent: the situation in the country has affected not only the iCCM service but all the services. it was also only our health facility that was providing service at the time, the others were closed, and all the focus at the time was on **"the March for Existence"**, and the community at the time does not give you the attention and was only thinking about the time when they will be caught, which their

concern was all about. The chance people came to us to seek medical attention was very minimal because they believe death laid up on by God was a blessing. Most people at the time was rather favoring their religious practices—praying and everything. Now the situation has settled but the pressure at the time was very high. If such things continue the challenge is going to be immense. The people beginning from the leaders not only the community were thinking when these institute was going to be robbed, and the health professionals has not been in the routine activity, they rather were dealing with dismantling and assembling of weaponries. It had a difficult impact on the health care service in general at the time, but thanks to God we have gotten through it.

Interviewer: How about the impact with the state of emergency?

Respondent: that too had specially decrease the case flow in relation with the mask use. It was circulated that no one should enter the health facilities without putting on any mask. The community refusing to buy it was going back. But I tried to solve the challenge by placing a face mask from the health center resources to provide those having no mask, and I was specially sympathizing with children coming for health care, so I tried to avail for those. But those who were not observed and remained at the gate with the guards were going back. They were then disseminating the information that the HC has prohibited service if we don't have a mouth covering, and missing to make a visit. I may not have mentioned this earlier, but the WDAs remained diffused after meeting were said to be banner due to COVID. The WDAs before this was functional and strengthened which about 75% were fully functional but after meeting was banned they could not come together again.

Interviewer: what was the difficulty for them to come together again?

Respondent: they had a lot of reasons, some say why would we call them back once we told them to go, and some of them were also bored from working for a long time. But it is still being worked on, and there is also a gap from our side to have a specific method to bring them back. What we did was also send a request for some of them to return back through some fliers and we also didn't do the same for all, so we have a plan to do it in the future.

40. How do you think the COVID-19 pandemic and/or COVID-19 response measures affect the uptake and/or delivery of iCCM services?

Probe:

- Has COVID-19 affected your daily routines; your work on newborns; the community in terms of livelihood and vulnerability for newborn care-seeking
- How has that changed over time?

Respondent: after the occurrence of COVID, most professionals had no attention to the work, and I would say it was stopped, and most were staying ideal in the health facilities. And from the request to wear a face mask, clients stopped coming. Concerning the health professional, they were very scared and fearful to attend to any patients because it was not possible what they might be having, but we have passed that moment.

Interviewer: Was there any challenge to going to the community, especially for the HEWs to make the house-to-house visit?

Respondent: the community does not have acceptance since they were having their face masks on when the HEWs were moving around. Some also suggest not to come to their houses because they might pass the virus. Some also asked when we were out in the community "what are you doing here when it has been declared for everyone to stay at home?". And said you may be bringing it to us. Hence, the HEWs having these reasons were not going to the community, and most children as a result were hurt at the time, and I also remember one child severely debilitated and referred.

Interviewer: How did it change over time?

Respondent: after the prevailing fear, there was the introduction of the use of sanitizers, keeping distance, then there comes the vaccination, and when people also understand the level of burden in the country, then it started to be familiar and now it is in a better condition.

Interviewer: How is it in the current time?

Respondent: Now regarding COVID, there is no problem including the professionals, since there is also the vaccine. I think it is the severity and burden in the country that calm everything. now there is no challenge to giving the services due to COVID. Now there is no one wearing face masks for COVID reasons because the professionals think they are vaccinated and judging the severity also we have become relaxed.

III. Adoption and reach/effectiveness

41. Explain to us any changes you observed as the result of the implementation of the COVID-19 adaptive iCCM implementation strategies? – What impact have the strategies had?

Respondent: we have implemented many activities despite COVID challenge in the last year, and after we got support, the burden become understood, and after we were vaccinated, everyone was doing their normal activities; holding back the fear and frustration, they were able to provide the service going in every household.

Interviewer: if you can directly indicate the changes in the iCCM service delivery obtained as a result of the implementation strategies and supports provided in the one year?

Respondent: one of the changes is in the early identification of newborns for treatment. They used to come after becoming severely ill but in the last year, we were able to early identify and detect sick children by actively looking for these children in every household, and making them start early medication, including on way of prevention of sepsis. The support from the project was also good that also included weekends and including support in the supplies; they have once availed gentamycin. When we also go for the CBNC purpose, we treated other cases like malnourished children.

- What are the particular features of the strategies that made a difference?

Probe:

- for a support system and linkages, motivation, and competence of HEWs, community, and awareness creation?

Interviewer: have your support system brought changes like improving linkage, etc?

Respondent: yes for instance, our support previously used to be based on mere checklist support and checkpoint marks but the current support is a detailed one on the number of newborns identified, number delivered, etc. for instance in the iCCM/CBNC all newborns whether sick or healthy are registered. Previously only those newborns who got sick after delivery were registered but after the training, all delivered babies are registered

Interviewer: What was the contribution of the support in terms of motivating the HEWs?

Respondent: Yes, they were motivated, for instance, we use some sort of mechanism to create a sense of competition between the HEWs based on the report they make, like providing a mobile card for the best performer. So it was effective to encourage others to do the same—this is what we did on our side. From the project side, it gave us training, and also supported us with a budget to make a review meeting at woreda level as well.

Interviewer: can we say that the support had significance in terms of creating community awareness, and with the WDAs functionality?

Respondent: Maybe it differs from kebele to kebele. There are some kebeles doing very well and working even better than the HEWs in some cases. But this is also dependent on our effort and the understanding level of the community. We have exerted similar efforts but there has been a difference in the understanding level of some communities.

Interviewer: what do you think is the strategy or support that made a difference in the iCCM service delivery?

Respondent: initially they were directly assigned on their duties after school, but the support hierarchy and strategy which we followed to provide the supportive supervision to the HEWs, after the training for the HEWs was provided by the project to fill on the identified knowledge gaps of the HEWs, I think has made a difference. The other is the participation of the community to aware about the availability of the services, making it part of the supportive supervision has also made a difference. Previously the community didn't know anything about the iCCM service, they only know vaccination and contraceptive pills are provided at the HPs. After we had the community discussion and creating this stage, the community has shown an improvement and I think this strategy has also proven effective to bring out this changes. The last thing is the commitment of the project and the constant enquiries have helped us produce a good result.

- What do you think are the reasons for non-significant changes?

Interviewer: what are the things you say, despite the efforts have not really produced a good result?

Respondent: I think it is the communities' awareness level that there is still a gap. As I mentioned it varies among the different community groups, but the achievement is still not to the level we anticipated it.

Interviewer: have you identified the areas that are of the community awareness gap to work on?

Respondent: it is what I told earlier, despite the efforts the communities still seeks medication from the traditional medicine. Since this is an awareness gap, I say if we can work on this, and I dint think this is something to be solved by the health center only, it requires the involvement of all parties including the WoHO and the project.

Interviewer: what do you think is the background reason for the community to still want to visit the traditional medicine?

Respondent: one of the reason is that she [traditional healer] is found to be providing the cure, and the other is the service inadequacy in terms of service interruption and shortage of supplies, and sometime the lack of awareness expecting a prompt change in the health condition after treatment by the community is also another factor—they want to see the change as soon as they brought him on spot, if that is not the case they take him to the traditional healer, and there is also a gap by the HEWs in informing the expected progress. So I say if we can work on this in the future.

IV. Maintenance and sustainability

42. What are the high-level benefits that are attributable to this support/IR?

Respondent: the filling of our gap is one thing because from now on we can sustain the activity. So I think our skill and knowledge gap is a major issue that is filled. Even the arrival of the supply support has also helped to maintain the services.

43. Please explain to us the feasibility of this support/ IR for national scale-up?

Probe:

- What features could easily be integrated into the existing system? Which not?

Respondent: well regarding the scalability, the challenge we mentioned needs first to be improved, if so, it will be very helpful. Our support will be sustained from now on because it has already been integrated in the HP-HC linkage which we develop for support; iCCM has been integrated in the checklist and support is being provided accordingly, so it will be sustained from now on and we will continue this way. The other is the gap with the HEW has been fully fulfilled and since we also discuss everything integrated in the checklist, and evaluate the performance, I think they will not have a challenge to implementing the activities.

Interviewer: what do the HEWs have fulfilled that could make them sustain the activities?

Respondent: one of the challenges they had was with regard to their skill and knowledge, and that have been fulfilled. The other is the supply issue, and the minimum supplies required to provide the services are available at the HC level, so we have the iCCM/CBNC supplies and when they require, they can request and get them. The other is that community awareness compared to the previous time is also in good status so as to sustain the services.

Interviewer: what about those support systems or activities that may not be sustained?

Respondent: if we still do not follow and provide awareness creation to the community, things might go back. If something is not done regarding the traditional medicine practice, it will also be a threat.

Interviewer: If you can see your response in terms of the activities that you have been implementing that may not continue and might require still support?

Respondent: Normally we don't think there are activities that are not going to be sustained.

44. How are the activities/efforts embedded in the PHC and woreda routines/work streams?
    - What implementation strategies are incorporated with the PHC and woreda annual work plan?

Respondent: we have normally integrated it into the routine activities; it is integrated into the weekly and monthly reports and the performance is evaluated to determine the progress level. For instance, when we integrate it into the weekly report, we include the number of births in the week, how many of them were born at HF, and we also categorize them based on their risk level for sepsis since they could be exposed to local bacterial infection. Then we make a specific follow-up. When we also meet at the woreda level, neonatal care is the priority activity that we first discuss.

Interviewer: have you put in plan some of the iCCM activities and strategies? and what are some these plans?

Respondent: yes, they are put in planning and used to measure the progress in the performance; they are divided into days, weeks, and months and even distributed even among the HEWs, and we let them know about the expected performance from each.

45. Do you have anything think is important to tell us that we have not asked you?

Respondent: finally, I want to thank the project for the support it implemented even going down to remote places and looking for newborns, which I want to thank them for they have been doing this for the country. The other which I think is worth incorporating in the iCCM service delivery is the supplies which we are sometimes challenged to avail at the HPs and so if it is possible, it is good if we get the support. I also heard some areas which does not have the project support are not implementing the same as here, so the government should give due attention to providing and sustaining the activities regarding neonatal care.

Interviewer: Thank you very much for providing me with a lot of information

THE END

End line Evaluation of the PSBI implementation research; Full Transcription for an In-Depth Interview with a Health Center Head

Questionnaire ID	01
Area Identification	Dembecha
Name of Woreda/Zone/Region	Dembecha WoHO/West Gojjam Zone/Amhara Region
Name of facility	Health Center
Name of moderator	
Name of a note taker	
Date of Interview	10/06/2022
Participant #	01
Audio File #	HC_Head_10.6.2022
Start time:	09:00 AM
End time:	10:40 AM
Transcriber/Translator	
Duration of IDI:	1hr, 41 minutes

Introduction and Consent

Interviewer: We thank you very much again. As we previously tried to introduce the objective of our presence today, it is in relation to the iCCM service delivery that you have been supporting the HEWs to identify and treat newborn illness in the community when it is not possible to refer to health facilities. For the last one year, you have also been doing this activity with the project support from JSI-L10K. Hence we have come to collect the information regarding the effectiveness of these activities, and the challenges encountered, which could also serve as an input for future planning. It will take some time, so are you willing to participate?

Respondent: Yes, I am willing. My name is Abebe Kebede [name deliberately changed]. I am the Dembecha Health center head, and I am willing to participate in the interview.

Interviewer: Ok thank you very much. So I have my interview question order and we will go accordingly.

V. Fidelity

46. Can you please describe the demand generation/SBCC activities you are doing on iCCM/ newborn health? What are the strategies used to raise awareness? If none, please explain why.

Respondent: even if the service on newborn health care has long provided at Kebele level, the information on the availability of the service in the community was very low. 02:23 …kewesde behuala.. In the last one year, the JSI-L10K has taken note of the gaps specially to treat newborns, and initially it was the awareness creation activity for the community that there is this condition the newborn health care service could also be provided by the HEWs, and that the supplies are also available and if things become beyond the HEWs capacity, there is also the condition arranged for them to refer to health facilities. These have been implemented in every Kebele and churches using the men development armies, and women development armies, and since this woreda is also one of the pilot sites we have communicated with the community engagement people available in the other form of social organization called "VHL" or "Village Health Leaders". And hence, the service provision has long been started at the HP level by the HEWs even before the one year. The service was available even before that but it was weak and not like the way it is provided now. The community-level awareness was low and the HEWs would have preferred to refer to the health centers rather than treat the cases by themselves. However, beginning last year after we discussed the issue with the HEWs, they started providing the treatment and still are.

Interviewer: you have mentioned that the service before last year was a bit weak and it improved afterward, could you tell me something you did differently to create awareness in the community?

Respondent: Thank you. as I told you, in the past even though the service was provided, the community does not know it existed. They have no awareness that the newborn health care service was provided by the HEWs. So what was done by the JSI-L10K is the conduct of the awareness creation forum held in the presence of the kebele leaders, key community figures considered to influence the community and communicate key messages, us from the HFs, HEWs, WoHO, and even people from zones. So the kebele leaders had a bigger role to mobilize and aware the community. After the JSI-L10K implemented this, we went back to the community and utilized different meetings during "EDIR", and at Churches to provide awareness on the availability of newborn care service delivery by the HEWs without going far to the health facilities, without any waiting and payments they have to make at the HFs and aware them that the services at HPs rather are provided for free. So awareness creation at the kebele level and introducing the service to gain recognition by the community was implemented last year.

Interviewer: What was the project's support that helped you to implement these activities?

Respondent: the first project support was in the conduct of the mobilization, which had a great contribution in making the service known at the kebele level, motivating the HEWs, and helping us also to engage at that level. It also supported in allocating a budget for the mobilization. Since there were people assigned, a follow-up activity was also put in place to see if the community received proper treatment or not.

Interviewer: Are there any other methods you implemented to aware the community in addition to the awareness creation activity using churches and public places?

Respondent: another method yes there are, for instance the pregnant mothers' conference is regularly held in every month. These mothers after their delivery are targets since there may be illnesses among newborns, so on the way the treatment is provided. So I said, the advantage of this service is one it is not far from their localities and the second is that the service is provided for free. The environment is also one they are accustomed to and with no major challenges—so this is one of the method that we have used to tell and inform the community about the advantage of the service. In addition to this, we have also the "VHL" social organization which we used them to communicate information in every village about the provision of the newborn health care service by the HEWs—this made it east since they have also taken the training.

Interviewer: is it the village leaders you said?

Respondent: there are the so-called "village health leaders". This is a new social organization in our area in addition to the WDAs, which serves as a bridge between the HEWs and WDAs. So we use them to pass information.

Interviewer: What was your reason to establish a new organization in the form of "village health leaders"? what was your base to establish the new social organization?

Respondent: it was JSI-L10K's initiative by the way after studying the situation in collaboration with the MoH. As we all know, there is a bit decline in the activities by WDAs nationally, so after studying this repeatedly on what to do, there was a need for additional people that links the WDAs and HEWs selected from every village based on their educational readiness, age, and acceptance or respect by the community. It has been around a year since this has commenced in our woreda and so, these people have education, capable of moving around at ease in-terms of their age and also have the respect of the community. These people have already been selected and engaged in the activities, and they are also more close to the community than the WDAs, and since they also greatly contribute in the reporting the messages were made to be communicated through them.

Interviewer: what is that you think you have got specially from participating the VHLs?

Respondent: what we think is special or different is that the selection of these people were made with greater care, they have the community's acceptance, they are stable and have the educational readiness to make the reports. They are also models in their own villages, and so they give you the cleanest and quality data on latrine conditions, newborns, pregnant mothers, vaccine defaulters since they are available in every village along with the WDAs. They have a very well organized data, can read, write and provide complete information. They are also very good in terms of mobilizing the community. However, when I say this, I am not referring that the WDAs are excluded, they also coordinate and

hold a regular monthly meeting with the HEWs. The current condition as it is now, I think has a good advantage.

Interviewer: whom do they report to—the VHLs?

Respondent: what they do is the compile the reports from the WDAs, include theirs and send to the HPs. Then the HEWs organize the reports and send it to us.

Interviewer: was it JSI-L10K who provided them the training?

Respondent: Yes, it was from JSI-L10K unless it is not different on how it came through the JSI-L10K—the JSI-L10K or MoH. Especially the woreda level training about budget which was a six-day training was provided by JSI-L10K.

a. How is the engagement of communities/WDA networks in the implementation of integrated community-based case management (iCCM) of common childhood illnesses/possible serious bacterial infection (PSBI)?

Respondent: meantime during the implementation, we have encountered some challenges that is soon after the VHLs took the training a perception was conceived among the WDAs as if they were completely replaced by the VHLs. Later we discussed with them in the presence of the Kebele leaders, informing them that the VHLs are only here to establish a linkage between them (WDAs) and the HEWs, and not with the intention to replace them. There has also been activity in replacing those WDAs who have served for a long time—six or seven years and replacing with the new ones, strengthening those doing well, and replacing those weakly performing with the appropriate praise for their services. I may not know the condition in your areas but here the overall activity by WDAs is slowing down and not like before.

Interviewer: What is the reason for the slowing down in the performance of the WDAs?

Respondent: the basic reason that the WDAs repeatedly raise is that previously they used to get some incentives when they engage in activities such as campaigns, but after the government interrupted this, they also show less interest in showing up here. The intensity with the HEWs in making the regular meetings is also not like in the earlier days. So it is in two ways, one is the lack of incentives and the other is the weak linkage with the HEWs—fatigued and developing boredom from working for some 15 or 16 years, and getting tied with their social issues like giving birth and health issues. These are the issues that are repeatedly raised and also we identified as basic factors.

Interviewer: How do you see the overall engagement of the WDAs in the SBCC activities and awareness creation?

Respondent: the engagement they have is numerous, and being specific to newborn health it includes the mobilization during vaccination, they provide information on newborn health care service availability by HEWs when there are illnesses. Sometimes there might be issues that go beyond them and that will be addressed by the leaders; for some parts of the community who are not responsive despite repetitive mobilization they are discussing them with the leaders, us, and the HEWs. Generally, the motivation of the VHLs is also very good. Initially, it was only in the Dembecha cluster that was first

initiated but now it expanded in all the 6 health centers; they have taken the ToT training, the selection has been made, and now training of the people and assigning them in the activities is what remains.

Interviewer: Was there any solution that has been taken to the challenges that you have mentioned so far? Any support that was provided to alleviate the gaps, especially on the motivation aspect?

Respondent: the action that was taken as a solution is that the WDAs and VHLs were made to discuss and raise their issues in the presence of the HEWs, kebele, and woreda leaders, and what was taken as the biggest solution is replacing those bored WDAs with the new ones, and encouraging and recognizing those who are performing well at the kebele, PHCU and WoHO level. That is what we discussed. **15:30**

Interviewer: what else? Was there any capacity-building activity implemented?

Respondent: Yes, especially the WDAs were made to take a 52 hours long training under the HEWs level and were certified last year; those who were tested and passed the exam were certified. Such motivational mechanisms were tried but it is still a bit difficult to say the previous performance level had been restored.

Interviewer: have you support in order to implement these activities or were you alone in this?

Respondent: well we had the JSI-L10K support in the previous time, the WoHO support, and since there is the expected HC-HP linkage, every health professional is assigned to support the HPs, and hence we are engaged in the activities at the level expected in collaboration with the HEWs.

Probe: What are some of the biggest challenges with SBCC activities for newborn care?

Interviewer: well you have mentioned different challenges, especially in relation to the HEWs, but what are some of the challenges you encountered with the SBCC activities for newborn care?

Respondent: specific to our cluster and in relation to the HEWs residence being near to their workstation there was some time-wasting caused due to frequent visits to their homes, which we discussed with them to remain and work from their workstation. There are also some others who don't have homes in the kebele or have difficulty living in the kebele which is a challenge we are still facing. Because of the delay to arrive in the working hours by these HEWs some clients may not wait at the HP when they see it is closed and might just go to the HCs since this is an illness and doesn't give time. In addition to this, there are some HEWs who are having health issues from serving for a long time and struggling to properly work now. Since our area is one in which many HEWs who have served a lot in other places want to make a switch; our site is having those HEWs who are aged, have health issues, or who are busy with their social issues. I don't know how this could be improved in the future but this is an area which we are planning to strengthen.

Interviewer: How about the challenge with the demand creation and SBCC activities?

Respondent: regarding the awareness creation, I don't see any challenge that we encountered. Unless for some gaps observed when we go to the practical implementation after we created the awareness, there was no challenge in the awareness creation. Maybe there was some concern in trusting the HEWs. They had no full belief that the HEWs could really treat their children and for that reason, they skip the HEWs and come to us. Hence, newborns come here for treatment that could have been treated at the HPs. We also discuss during our PHCU meeting; that we single out the newborns who were sick in their respective kebeles and treated. For instance, if it is at Godo Ber Kebele we discuss newborns from 0-2 months who were sick and those who were treated, including the reasons why they were not treated at the HPs and sent to us. So there was no challenge in the awareness creation activity, but after the awareness creation, there were other gaps in maintaining punctuality by the HEWs and community perception lacking full trust in the HEWs to treat their children and coming to us to get the services rather than going to the HEWs.

Interviewer: what is the reason? Why are they leaving the HEWs and coming to you? What is this associated with, why couldn't they use the services at the HPs instead?

Respondent: there are people who think it is better to get treatment at the private health facilities instead of the public health facilities right? Just like that, there are also people who think they would get better treatment if they go directly to the health centers rather than the health posts, which is thought by most.

Interviewer: Have you tried to employ any other methods like banner or poster for the SBCC activities regarding neonatal care by HEWs? Or if anything you want to utilize but could not due to different reasons?

Respondent: we have posted a banner at the health posts listing the different available services provided at the health post level. It is obvious that people visit the kebeles for different reasons so we have placed these banners depicting about the availability and provision of these services in all health posts.

Interviewer: What was it about, the banner you displayed?

Respondent: there is the neonatal treatment service, and other services are also available to make it comprehensive.

Interviewer: can we generally say the community perception has completely changed because of the activities and seen with respect to the objective you set out?

Respondent: All I can say is that it has relatively changed seen with respect to the past. However, it is difficult to say it has completely changed. As I was saying, there are variations in the community perception; some might think it is better to go to the health centers than the HPs, and even there are preferences for the different HEWs. Hence, it would be hard to dare say it has changed 100%.

47. Could How do you describe the strength of the support you got from the PHC and the project as well as your efforts to address barriers to delivering iCCM/PSBI during COVID-19?

Respondent: the support was intensive and provided in a way to capacitate the HEWs skill. People from JSI-L10K visit the HPs, see neonates who have been treated, evaluates whether the treatment provided matches with the service provision and they also provide us feedback after their visit. They raise issues if they are present during our PCHU meeting. They also trained two health professionals from the HC to support the HPs every month. So generally the support was good.

Interviewer: Were there any aspects or forms of the support to regard it as strong, it could be in terms of building skill to provide the treatment at HP level or any other different support?

Respondent: as I tried to mention early on, there was a lot of support to commence the medication service beginning from the registration, availing drugs that are difficult to get like gentamycin. They even brought the HEWs and us together for an experience-sharing session by bringing the registration books; the HEWs switch places and see the age, the administered dose, the classification, and what has been missed such as the dose, or the route of administration. Then we go to evaluate the performance, so the support was being provided to this extent and was strong.

Interviewer: what about the support provided to alleviate COVID-19-related barriers and maintain the services?

Respondent: in relation to COVID-19, well within our community we were all worried especially at the beginning. However, as it becomes familiar it has not been much of a challenge, and there was no one who refrained from coming because of this, they would not keep away even if we say so. What was being done at the HPs, in spite of this though, is sanitizers and face masks were available and we also provided education for people coming to the HPs about the protective measures. COVID-19 vaccination has also been provided in three rounds and not only that, we also been providing education on COVID-19 protective measures using every opportunity and when we also go to the community together with the HEWs. But COVID-19 is not that a big challenge now for our community, we are living within a community that still thinks COVID-19 does not exist. So they don't stay away from getting the services because of COVID-19.

Interviewer: Ok the current situation might be the way you stated, but going back to when it first occurred what were some of the main challenges encountered and how did you address them?

Respondent: since we have been evaluating the challenges we have seen that we did not face a significant challenge with regard to service delivery. Back then what we evaluated was that most of the services—except for chronic disease and TB, the others such as vaccination, and delivery services were in good status. Even in terms of number, they have not shown a decrement. This is because we were implementing in a way that the services should be maintained, that is since we were telling the community to get vaccinated and seek medication for their sick children cognizant of the COVID-19 challenges. It was may be in the first one or two weeks that the community was worried and didn't come to get the services, but afterward, it increased. However, our OPD per capita has extremely decreased that is frankly speaking; ART defaulters increased, and those being treated for TB had the

intention of defaulting. We however didn't see major challenges in the delivery, postnatal, and vaccination services.

Interviewer: So how did you evaluate the performance related to the iCCM service delivery? How did you compare the impact whether it is related to the before COVID or after?

Respondent: how we evaluate them is we compare the numbers in the specific months before and after COVID-19 pandemic occurred. Then we evaluate the performance improvement after the COVID-19 awareness creation activity was implemented. This is done for all services including iCCM.

Interviewer: so are you saying there was much challenges encountered due to COVID-19 and most were pre-existing?

Respondent: Yes, there was much change die to COVID-19.

Probe: What was changed?

Interviewer: talking about the support provided still, what are the changes registered as a result of the support provided in the last one year. You can compare and tell me what was before the one year and after the support in the last one year?

Respondent: regarding the support by the HC and the project, it was provided in an integrated manner using supporters from our facility, and hence the supporters develops the tendency to be motivated and provide support with knowledge when they are refreshed with knowledge and other from the joint activity. However, the challenge from our side was we had a high case load—the Hospital was not available and recently opened, and because of that it was challenging to go out and make a field support. Other than this, the assigned kebele supporters are happy to be involved in the activity. So, JSI has been supporting us to this extent and the two professionals JSI trained from our HC has a good contribution in terms of regularly supporting the HPs in every month and correcting the gaps and improving their performance.

Probe: Was the support you got from the project and PHC helpful? What could have been done differently?

Respondent: I don't think the strategy by itself had a problem but there might be some gaps in terms of the implementation. There is a frequently raised issue with a lack of budget to cover the perdiem costs for field-level engagement, and vehicles and motorcycles' inaccessibility are some of the implementation challenges. Unless for these challenges the strategy is relevant but there are gaps in the implementation.

Interviewer: if you can elaborate on the implementation gaps a bit more? And if there are other challenges as well?

Respondent: we have one motorcycle that is currently allocated and one ambulance which is not adequate to cover all the 8 kebeles we have—6 rural and 2 urban kebeles, and the kebeles are also sparsely distributed. For instance, if they were to go to these kebeles at the same time, it won't be possible. Sometimes they use public transportation but their cost spent on this is not reimbursed. So these

are some of the challenges hindering the full implementation. If these challenges were managed, we could have established an even stronger linkage with the HPs and be able to support the HPs any time if vehicles were availed. The use of public transport also has time delays, and exhaustion and even the HEWs might not be available during arrival. Hence, in order to effectively make the support vehicles need to be availed or the public transportation cost spent by the supporters needs to be reimbursed.

Interviewer: Can you mention some of the gaps that resulted or something you planned but could not achieve as a result of the challenges you mentioned?

Respondent: what we planned but didn't achieve is the CHIS which is implemented both manually and electronically. We are behind our plan which we should have finalized the household registration, family member registration, and we should have started providing the service but there are some HPs with the gap. Other services are also not implemented as per our plan such as the family planning, ANC4, delivery even is relatively better at 76% but it didn't reach our target at 80%. So these gaps have occurred in relation to the challenges.

Interviewer: are these related to the iCCM service delivery?

Respondent: Yes, for instance, the condition for the newborns delivered here to be optimally followed and registered for PNC and treated is not conducive. Newborns might come when they are sick but what it should have been, all those newborns delivered should be recorded in the PNC register and their condition should be followed. So I think it has an effect in this regard.

48. Do you think the support system is helpful (mentorship and on-site coaching methods, supervision, technical support, PHCU level PRCMM, etc.) to enhance your skills [that is HEWs' skills] over the traditional off-site training and woreda level review meetings?

Probe: for acceptability of the strategies **(for HEWs only)**. How could the support system be improved?

Respondent: as it is known the HEWs have been taking a lot of training but the extent to which they understood it is not satisfactory. So what was done considering is first they were made to take an offsite training to get refreshed and then support was provided by people coming from the JSI-L10K at the health posts in the form of the on-the-job mentorship. In addition, when we are also conducting our PHCU meetings the HEWs are made to exchange the register they have been working and provide comments to one another. There is the classification, the dose, and there is the symptom and there was a detail discussion about each which was highly important to build their skill. So I think this is the most basic and better than the one provided in halls since the demonstration is about the practically implemented activities. Gash Abera *[name deliberately changed and he is referring to one of the JSI L10K staff supporting the HEWs]* was also frequently supporting them even during our absence. He evaluates the performance and skill of the HEWs investigating the relevance of the

treatment they provided from the register. Hence, I believe now the HEWs have got the required knowledge and skill to provide the service compared to their previous status.

Interviewer: Ok that is good. Is there anything that you suggest should be improved about the support?

Respondent: I think it is better if there is a mechanism to encourage and recognize those HEWs who have a better performance than others. It will be good to consider a mechanism to improve their skill since it is possible through motivation and keeping their morale up. I am talking about this from past experiences observing the performance of some HEWs who could not still improve and for such kind of performance gaps, I think it is good to consider different strategies to build the HEWs' skills.

Interviewer: Ok you said the support compared to the conventional off-site training is good. So How did you see the acceptability of this support by the HEWs?

Respondent: well the difference between the offsite training and the on-the-job training might be about the incentive issue. People might be delighted with the incentive during the offsite training but might not feel comfortable when we are doing it onsite only and since there is no payment they might not be happy. However, what I can be sure about is the onsite is much better in terms of delivering knowledge.

Interviewer: is there any specific observation that you can tell us they have improved due to the support [onsite]?

Respondent: one of the things improved is the gentamycin injection. Previously they used to be very fearful to provide gentamycin injections but this year I believe they have got the skill and are now capable of administering the injection. During our meeting, we have also come to know that they are capable of administering the specific dose including the dose preparation. In addition to the JSI-L10K support, we have also provided them training here in collaboration with our pharmacy professionals on the dose preparation and on how to reduce the dose, and administration. Hence, I believe they have got a special knowledge regarding this, on top of what they know and have already been doing.

49. How eCHIS implementation helps you with iCCM service delivery?

Respondent: neonates are one of the things which the electronic system aggregates, they are included inside. Some of the services included in this are family planning, and EPI—children are also included in the EPI since there is a specific section about child care. So when they are providing the medication they feed in about it since it also asks about the progress; whether he is dead, recovered, or lost to follow. This means, there is an action card in which if they don't follow the progress properly, the system flags red which alarms them to take follow-up action. So I think this will help them make a follow-up about the whereabouts of the child till the end.

Probe for advantages:

- case identification? quality of iCCM case management? retrieval of client records? data quality? client appointment scheduling and/or defaulter tracing?
- **Ask why?**

Interviewer: what else, would it have importance in the case identification?

Respondent: For the case identification since it is already registered, they use it to target children for vaccination, and they also address related issues during the vaccination and communicate about sick children who were sick and not treated at the HPs—this I think will also help them evaluate themselves.

Interviewer: what about the retrieval of client records and information?

Respondent: it has numerous advantages in this regard, there is no manual recording for instance, and if this is also properly implemented there is a chance for us to retrieve reports using their user name, without asking them. There is an aggregated number for the different services implemented which we can easily retrieve.

Interviewer: Is there any aggregated report for the iCCM service delivered too?

Respondent: Yes, there is also about child care. If we go into the system, it can show us the neonates treated in a kebele. Maybe it might not show us about the progress and others, but it can show us the number.

Interviewer: would it also have importance in ensuring the quality of case management or the data?

Respondent: I am not sure about the quality I may have forgotten about that since I didn't see it.

Interviewer: how about for the defaulter tracing or scheduling?

Respondent: regarding that, it can make a schedule for the days the HEWs have to make a visit and if they don't go on that date it flags red and shows an alarm but when after they assess and feed the system, it turns green.

- **Probe for areas of strengthening?**

Respondent: the first will be for the HEWs to handle this well and feed the system (eCHIS) properly. The other is if it (eCHIS) also incorporates a feature to show the progress as well. The red flag is just for the schedule when it is missed. But it would be better to show the progress of the child after the treatment, whether recovered or not. So I think it is good to consider such things in terms of data quality and management and incorporate them.

50. How was the introduction of the iCCM/CBNC module into eCHIS affected the iCCM/CBNC service delivery? **FOR LUME WOREDA ONLY extra**

VI. Implementation challenges

51. Describe the main issues faced by the health system to identify and treat neonatal infections in the community. What are the critical factors affecting the delivery of iCCM to clients?
    Probe:
    - lack of competence of HEWs, shortage of supplies and commodities; weak support system; low community demand?

Respondent: when we come to the health system challenges which we also frequently raised is the women's development army which has been challenging us to make it functional. Of course, there are these

community engagement wings known as “village health leaders” that has recently been introduced, and I say if they are well strengthened by collaborating with the HEWs, and since they are also the ones bringing back things from the HEWs the supportive supervision should be much strengthened with them. They were once engaged as a pilot and active, but if the tempo of supportive supervision and meetings declines they will also come out of the system and leave the activities. If a conducive environment is facilitated for us to meet with them at least in every quarter, all the services including iCCM will improve.

Interviewer: What was the reason you could not do this on your own?

Respondent: the reason why we couldn’t invite them over and evaluate their performances is that there was no concerned body who could support us with a budget to undertake the activity. Since these people are farmers and depend on their daily activity for their livelihood, we should at least cover their daily expenses if we are going to invite them to come here. Around the time the project commenced, we were able to meet and discuss with them 2 to 3 times with the support of the JSI-L10K and the ministry of health. But it could not sustain and, was interrupted afterward. The government could not sustain it. It is at the kebele level that we meet them now but it has a lot of interruptions and is not the same as the evaluation we make here. There is a chance to exchange and share experiences among the six HPs available when the meeting is conducted here.

Interviewer: Any other challenge?

Respondent: Another challenge that I consider personally is the HEWs boredom. Most of the HEWs under my cluster have worked for about 15 or 26 years, and some of them are having health issues, or being tied up in their social matters like giving birth, and as a result, there is no home-to-home visit currently made as in the previous times. Hence, it is if the HEWs are strengthened and some sort of mapping to indicate where the HEWs could be going from now on. So if these two basic challenges are solved the others I think won’t be difficult.

Interviewer: Ok there was also something you mentioned about an incentive earlier?

Respondent: yes, for the WDAs. It is what they have been accustomed to by the way in the earlier days when there are the Carter Centers’ campaigns like deworming, and vitamin supplementation, and when there are such campaigns they used to participate and there was a payment that they used to be paid together with the HEWs. It is after this incentive ceased that they also stopped supporting the HEWs. It was mainly during campaigns that they get paid when they move around along with others.

Interviewer: but what do you think is the main challenge existent in the health system that is a factor for the effective identification and treatment of neonates in the community by HEWs?

Respondent: Ok even if this challenge has relatively improved, there was a lack of belief and trust by the community that the neonatal care could be provided at the HP level, and prefer to go to hospitals and health centers instead. This community perception might be solved by about 70% but it still

exists, and we are expecting the change to come gradually. The other is the HP should be opened to provide 24 hours' service but the HEWs might come here or for some reason, the HP might be closed by the time they arrive at the HPs and assume as if it is closed all the time and rather come to the HC next time they seek similar service. They also rapidly communicate and disseminate the information that the HP is always closed and does not give service to the community. But if the HEWs were more than two, the HP will be opened and service will also improve once the community gets used to this. The other challenge is the disproportionate number between households and the HEWs; this is a serious challenge in our area. I think the revised assumption also states 330 households to 1 HEW, but if we calculate this in our context it is around 700 households per one HEW each for the total 19 HEWs we have. This is causing a lot of burden for the HEWs since they are not only engaged in this. so I suggest that the household to HEW number becomes proportional.

Interviewer: What is the impact HP's closure and the disproportionate household to HEW number had on the iCCM service delivery?

Respondent: it goes along with the others. for instance, a HEW with 700 households and 330 will not address them both equally. The one with the proportional number will have more chance to cover them and reach, be it for EPI, or iCCM it will be more efficient.

Interviewer: My other question is, was there any underlining reason or anything they observed contributing to the community's perception to seek the services at the facilities other than the HP? Fending them off?

Respondent: one thing still is if the HPs be opened and provide service at all times. If they see a child who got cured at the HP, they will stop coming to us, for that the HEWs capacity should be built. If this is so, it will signal a message in the community that if they too bring their children, they will be cured. If tailored training is provided for the HEWs, they will improve their skill and at the same time, the tendency of the community to seek the service will also be improved.

Interviewer: how about the competency of the HEWs, can we mention this as one of the implementation challenges?

Respondent: yes, we can mention that as a challenge because they choose among the HEWs and associate safety with some HEWs and leave out some others which indicates the perceived difference in the knowledge and skill of these HEWs. Not only this, we also observe variation in the performance of the HEWs and gaps when we call them here for review meeting despite repeated training. The gap observed in the registration implies the inevitable occurrence of some gap in the actual service delivery, such as route of administration, dose preparation, and cleanness.

Interviewer: How about the supply issue?

Respondent: it was very challenging, especially some supplies that were not available in the market, for instance, the 10mg gentamycin was difficult for us to get. This is where we also want to thank JSI-L10K which supported us in searching for this drug and avail it for us. We could not find this from PFSA or other

partners but since it was also possible to prepare it in a different way, we were working with a consensus with them as well. Well supply is a challenge as it is known, but since we were collaborating with the WoHO, other HFs, and the JSI-110K to address the shortage, it was not a big problem for us.

Interviewer: well you have mentioned different challenges, especially in relation to the HEWs, but what are some of the challenges you encountered with the SBCC activities for newborn care?

Respondent: specific to our cluster and in relation to the HEWs residence being near to their workstation there was some time-wasting caused due to frequent visits to their homes, which we discussed with them to remain and work from their workstation. There are also some others who don't have homes in the kebele or have difficulty living in the kebele which is a challenge we are still facing. Because of the delay to arrive in the working hours by these HEWs some clients may not wait at the HP when they see it is closed and might just go to the HCs since this is an illness and doesn't give time. In addition to this, there are some HEWs who are having health issues from serving for a long time and struggling to properly work now. Since our area is one in which many HEWs who have served a lot in other places want to make a switch; our site is having those HEWs who are aged, have health issues, or who are busy with their social issues. I don't know how this could be improved in the future but this is an area which we are planning to strengthen. ***Also Repeated Above under SBCC but Inserted here again for the Answer is more an implementation challenge than Specific to SBCC***

Probe:

- Regional/national state of emergency and conflict in the northern part of the country?

Respondent: the state of emergency impact due to COVID-19 may be reflected at the HF level and the HEWs staying in their homes thinking there won't find anyone to give the service to since it was being communicated for the community to limit their activities and remain in their houses if possible as a prevention measures. Other than this we were communicating with the HEWs and activities such as EPI, emergencies were conducted. So it might have some impact, especially for some services that needed to be reminded but there was no major influence on our service delivery in general—this is what I remember about the iCCM service delivery as well.

Interviewer: what about the conflict in the northern part of the country?

Respondent: which I think had a bigger impact is this one. Beginning from the onset of the war, and when it was told that they are approaching (soldiers from the other side) most people including us instead of focusing on the activities, become frustrated that their facilities will also face the same fate of being demolished like the rest. We can say that we were not working for almost 2 months because of this except after things starts to settle and we discussed commencing the services. There was also no accountability or responsibility at the time, we were not asking for any reports or activities implemented. The war also affected us psychologically.

- How has that changed during COVID?

52. How do you think the COVID-19 pandemic and/or COVID-19 response measures affect the uptake and/or delivery of iCCM services?

Probe:

- Has COVID-19 affected your daily routines; your work on newborns; the community in terms of livelihood and vulnerability for newborn care-seeking
- How has that changed over time?

Respondent: when we look at COVID-19 impact on the iCCM delivery from the community perspective since the newborns we are treating for iCCM are not in the position to apply the COVD prevention measures and fear of the presence of COVID-19 at the time, they might abstain from visiting the facilities early when their newborns are sick unless their illness gets serious. This is what I think and believe personally. We have also understood mothers were seeking treatment for their newborns after an elapsed delay due to COVID, for instance, after their newborns were already dehydrated due to diarrhea. There was this trend in seeking treatment for neonates after complications both at Kebele level and here. Since the severity of the newborns was determined by their parents' evaluation, I think there were some delays which we also discussed previously as a unit. But when we generally see the trend in the number of newborns treated before and after the COVID-19 pandemic, there was some difference but it was not a significant one.

Interviewer: What was the impact from the health professionals' side because of the occurrence of the COVID-19 pandemic?

Respondent: some HEWs in relation with the COVID-19 and since the symptoms were similar with pneumonia and other diseases, there was fear among the HEWs when they see such similar symptoms and sending clients to HCs even if it is occasional. In this regard, we have also encountered 3 to 4 cases here that needed to be treated at the HPs, assuming that it could be COVID-19 without really appreciating the sign and symptoms. As a result, there was some push to send clients to HCs, hospitals, and private health facilities in relation to COVID in the previous times, that is before 6 months and beyond. Currently, however, COVID is not the community's problem that is frankly speaking. The focus still may be from the health sector and the government side but when we see the community, they consider it is the government spreading fear and believe it was not even there in the first place, since they are also telling us the same thing now when we go for vaccination.

Interviewer: If you can also explain the impact in terms of executing the daily-to-day activity by the health professionals and providing the iCCM service?

Respondent: it is almost the same. Maybe at the beginning, we were also scared to come to the HC and it was only because it was an obligation for us to come. But in the meantime, we have to come to understand that we could prevent it, and our fear starts going away, and there was a bit of belief both by us and

the HEW that if we even contract it there is a chance for survival. When we look at the current situation, we have got the vaccination and if we contract we believe that the morbidity and severity are going to be mild. So it is also similar to the HEWs, there was fear and limited activity at the beginning and later things start to improve. Regarding the community, they have now concluded that it is not existing. In the beginning, however, the community was not coming out and the HEWs also stayed in their residence. Currently, there is a better condition, there is also increased flow compared with the previous time.

Interviewer: can we say the COVID-19 impact mentioned could directly be related to the iCCM service delivery?

Respondent: yes, at some point there were also some people who said the HEWs are going to bring the virus to them in relation to the urban HEWs. Who said they prefer if the HEWs don't come to their houses because they are going to expose them to the virus and because they have kids.

Interviewer: So this is directly associated with children?

Respondent: yes.

Interviewer: Is it mainly those who had kids who say this?

Respondent: yes. It is the HEWs and generally anyone coming from the urban areas, including us. They don't like anyone coming from the urban areas, they think anyone from the urban area is having COVID.

Interviewer: How about now?

Respondent: now it has changed. COVID currently is not the community's basic problem, our community instead is suffering from the inflation and the cost of the fertilizer, etc. There is not much concern about being exposed to or infected by COVID.

Interviewer: What was the impact COVID-19 brought to the community that challenged the identification and treatment of neonates?

Respondent: I would not say that the community utilized the prevention and control measures of COVID in terms of handwashing, wearing a face mask, etc. We had strict policies not allowing clients to enter the premises but it was difficult dealing with them because they might put it off after entering. The health professionals on the hand had adherence to the prevention measures.

Interviewer: was it not difficult for the community to apply the measures in any way and were they complying with your instructions?

Respondent: they will not be allowed to enter if they don't have a face mask on. However, there were no compliance challenges from the difficulty of affording the face mask prices since we ourselves provided the guards to sell the face masks with 5 to 10 birr, it is cheap. We didn't tell them to bring the face masks, we just availed the masks to the guards to sell to the clients. It was not the price that was a problem for them, it was the difficulty in the utilization.

VII. Adoption and reach/effectiveness

53. Explain to us any changes you observed as the result of the implementation of the COVID-19 adaptive iCCM implementation strategies? – What impact have the strategies had?

Respondent: we are going back to even before COVID-19 regarding this. Our community's practice to take their children to health facilities was not that satisfactory, they used to take them elsewhere or they will just keep them in their house with the belief that they will just get better without any medical attention. Many have died as a result of this. However, the current change in the community's belief that it is possible to get a cure by seeking health care is immense. It was also JSI-L10K who advocated the provision of this service in the community, and its contribution in this regard is huge. Now, most community member knows that there is a health care service for neonates at the health posts provided by the HEWs. Who introduced the provision of this service is the JSI-L10K using the community agents (Village health leaders) I mentioned early before and after the COVID-19 pandemic informing them that children who are sick should remain home and that they can be treated and cured. It also provided the awareness if they are not treated at the HPs, there is also a way to refer them to HCs or the hospitals. It was like this in the earlier times in the community, they believe that a newborn who is sick is not going to be cured and to their best, they might take him to a traditional healer; in traditional medicine, there is an ointment they put on. But it was through the JSI-L10K that awareness was created using the religious leaders, WDAs, kebele leaders, and other people. And the activity has also come along with the occurrence of the COVID-19 pandemic.

What are the particular features of the strategies that made a difference?

Respondent: well with regard to describing the JSI-L10K's contribution (project), it goes beyond and enabled us to have fences for all our health posts. It taught us how we engage with the community to mobilize resources and construct a fence. As a result, we were able to build a fence for all our 6 HPs by the time JSI-L10K was active.

Interviewer: But what was that you implemented to motivate and engage the community in the activities?

Respondent: the first is to make the kebele leader work with you because he is the leader for that kebele, and if he orders and mobilizes the other WDAs, it can be implemented. That's why this approach was used, and he aware the community of the importance of the HP and the consequence if the HP is not available and the challenge they will be facing to get a health care service for their children once the HP is no available. Then when we go there, we will get all the community gathered in one place, and the next time they bring each one wood for the fence, and the next time they dug the ground. JSI-L10K has invested a lot on the HEWs, I think if the HEWs in this area are compared with other HEWs, there will be much difference in terms of knowledge; they have taken a lot of training and a lot of activities have been implemented over the years, and I think a lot has changed which we might not

quantify at this moment. With regard to skill, they have worked on the HEWs and WDAs, and they have taught a lot. They have also provided training on mentoring for HC staff. Their supportive supervision was also different, for instance, if they plan to go to the HPs at 8:30 am in the morning, they will be there at 8:30 am which is different from the government system which a plan for 2:00 am may be made at 10:00 am, so they have also taught us about commitment. When we make it more specific to iCCM service, they have shown many parts of the community who believes that neonatal care is not provided at the HPs to know that it actually is provided. They have fulfilled may supplies and many newbords have a treatment as a result. They have also coached the HEWs on how to identify disease in newborns and treat them. The performance has also improved cumulatively from all HPs in the catchment, and generally they have also taught on how to effectively approach the HEWs and implement the activities, that is what I think personally that is on mentorship. Even when we are holding our PHCU meeting, they have shown us in how to identify gaps and boldly depict the gaps. So I think JSI has done a lot of activities specially in this woreda.

Interviewer: You mentioned boldly about the mentorship. how often do you make the mentorship to support them?

Respondent: there is a monthly mentorship, also weekly mentorship and to the minimum, it might be in a fortnight. It might be difficult to implement it this year due to the different campaigns we are having but whenever people from here go there for other activities we also make them assess the iCCM activities.

Interviewer: You mentioned a lot JSI-L10K. How about your support? Is it in collaboration or individually you go to support them? And how frequent?

Respondent: we go for mentoring both separately and jointly when the JSI-L10K is present. The project used to make every month for the 6 rural HPs we have. Mostly it is an integrated activity and the checklist used also incorporates all—our checklist and the project are aligned in one.

Probe:

- for a support system and linkages, motivation, and competence of HEWs, community**,** and awareness creation?

Interviewer: ok what about the changes in relation to the support system and in improving the linkage overall in order to provide the iCCM service?

Respondent: the iCCM service performance is evaluated using the dashboard as one of the KPIs. One of the measuring indicators among the 18 KPI measurements is also neonatal care. I have had the figure as well, for instance, the last year's performance was 37% but after JSI-L10K has come around it become 58%; it has made many newborns get this service both at HC and Kebele level (community level). It has also been made for many people to be aware and bring their newborns for medication. In addition to community awareness activity, the HEWs were also supported in correctly identifying

the type of disease for the newborns, that is capacitating the HEWs to build their skills in correctly identifying the disease types based on the specific sign and symptoms and treating accordingly. I think JSI-L10K has worked well on this and its contribution was commendable in terms of performance and quality of service as well. I believe the improvement from 37-58% is quite a big achievement.

Interviewer: How about the linkage you had with the HEWs has it improved because of the support?

Respondent: our linkage as I told you, the JSI-L10K has provided the mentorship training and they also make visits every month. The Kebele supporters also make visits and we have also incorporated this in the checklist hence one of the things we ask the HEWs when we go there is for the number of neonates she treated from 0-2 months, and if treated, how they were treated and with what is tried to be incorporated in the checklist to help them assess this in an integrated fashion. So our linkage is strengthened to this extent, but previously it was only the available services people observe during supervision. In consideration, it is now made to be included in the checklist.

Interviewer: what about change in the motivation of the HEWs, have you seen any change due to the support?

Respondent: what we do to motivate them with Gash Kebede *[name deliberately changed and the person is one of the JSI-L10K staff]* is we select the best performers first, and loud their performance with applause mentioning that Adanech has managed to treat 2 newborns correctly. We try to motivate them this way, and for those least performers, we mention their least performance and suggest the areas they need to improve being humble enough not to also hurt their morale. This I think is one of the motivation mechanisms in addition to the provision of some kind of reward, that is praising their performance where people are gathered. Then after, it also opens a room to exchange experiences among the HEWs on how each has been implemented; there was a change we observed from this practice, some who weren't providing the service have commenced, etc. For instance, there was one HC I remembered that has been providing the service for months, then we exchanged the registration of the HEWs, praised those who performed well, and suggested improvements for those not doing well, then within the same week they returned they managed to treat 3 or 4.

Interviewer: How do you do this? is it by inviting them over here?

Respondent: Yes. It was during our PHCU meeting. The first thing we do before we go to the performance evaluation is made them exchange their registers to provide comments among themselves. Then we recognize the best performers identified by their names.

Interviewer: was this activity supported by the project?

Respondent: Yes. It was the JSI-L10K that supported us to implement and get used to this practice. not only this, but they also supported us on how our support system, on how we approach and motivate people. Previously, we were only focused on the performance figures and asking for the reasons only, but the support taught us how we approach and could motivate people.

Interviewer: What was the change it brought to the functionality of the WDAs and discussions?

Respondent: the project support had its own contribution, people from JSI-L10K were present when we were holding our community meeting with the WDAs, VHLs, Kebele leaders, etc and directions were also communicated from the JSI about the implementation. Then after, we held a discussion with the WDAs and VHLs, and what we found out regarding their attitude and reflection was quite different from the first meeting. They had the desire to engage in the activity.

Interviewer: What does VHL mean?

Respondent: it means village health leaders.

Interviewer: Oh the one you have been saying "Gote leaders"

Respondent: Yes.

Interviewer: Ok please continue.

Respondent: So since there was a prevailing thought that they were replaced, it was left for us to bring these two together to clear the issue in a meeting. Hence one of the commendable activities JSI-L10K did integrate with us is this one since the project staffs were also present in some kebeles. It allowed us to introduce the role and responsibility of the VHL and WDAs, also what it means by "VHL". It was also the JSI-L10K that strengthened the WDAs in collaboration and recommended replacing those bored WDAs.

- What do you think are the reasons for non-significant changes?

Respondent: what I think didt bring about a change as per the effort exerted is the WDAs functionality and engagement.

Interviewer: what do you think is the reason behind it?

Respondent: what I think is a basic problem for this is that we have not been categorizing the performance of the HEWs as A, B, and C which is expected to be made in every quarter using the criteria sent from the MoH. The WDAs issue is related to the interruption of the incentive they used to get, the government didn't accept this yet but it is a very important issue. We have raised the issue in different stages but they are saying they could not afford it and for other reasons which seem ideal. The other is the decline in the momentum from our side of pursuing the activity implementation whenever the support from JSI-L10K seems to decrease. There is a trend in engaging highly in the activities when JSI support is active and becoming weak when they are not around. It has been a while since the JSI support has interrupted and I think they have finished their project time.

Interviewer: which activities are you not continuing or interrupted due to their absence?

Respondent: the mentorship activities are not done as before, and also since the campaign has also become many there seems to be a lack of attention from our side unlike before.

Interviewer: What are the activities that you must need their presence? Are there no activities that you can do on your own?

Respondent: it is for the monitoring and evaluation activities that we need them, not for any other. It is more strong when the activities are evaluated in their presence and the degree to which it is evaluated is different. It might not be that strong with us since we have a lot of other duties.

VIII. Maintenance and sustainability

54. What are the high-level benefits that are attributable to this support/IR?

The benefits have been mentioned in the above section in relation to the changes and strategies

55. Please explain to us the feasibility of this support/ IR for national scale-up?

Probe:

- What features could easily be integrated into the existing system? Which not?

Respondent: regarding the scalabilty of the support to other place, well it different from the government system and what makes the JSI-L10K implementation different from the government is that the monitoring and support supervision is very good. It monitors properly and makes a review. Since the activities are not reviewed and evaluated there is a chance the poor and strong performances might not be differentiated and under looked all together. But if the government strengthens this, there is a chance to sustain the activities; there is a need to stick with schedules set to conduct review meetings say quarterly, and a ground for accountability for non-performance and recognizing good performances should be established. I think it is doable if these are implemented. It is always the practice from my experience that activities are well implemented when partners are active and wanes when the partners become inactive. The government might just have the idea of sustaining it but the activities in conducting supportive supervisions using a checklist, review meetings and efforts in creating accountability are not implemented in a similar manner when the partners were available.

Interviewer: Now that you have been working with the JSI-L10K for the last one year yes. Now if you can tell which activities do you think will sustain and which might not from now on?

Respondent: ok if the JSI is no more available, the activities will sustain but it will be a bit slow. It is good to look at it in two ways, one when Gas Kebede [JSI staff] was present and now after his absence is different. We have our checklist, and the HEWs will come for the PCHU meeting and we will evaluate their performances. But it might not be as strong as it was during the project implementation, however, activities we have started implementing will continue. It must be understood that I am not implying that all things won’t continue after the project. There are activities we commenced which the communities also have been introduced to, and if the community seeks the service at the HPs, it is

not like the HEWs will send them back because JSI is not available. We will also avail the supplies. The difference maybe in terms of making regular mentoring to measure their skill level. And since science is dynamic, I think there might be a slowing down in making regular updates to them.

Interviewer: Why would it slow down and who should also be making the mentorship in your opinions?

Respondent: why it slows down is for instance if we are busy with other things today but JSI want to make a visit, then they meet the HEWs on their own, and if this is their schedule then they evaluate their performance and ask reasons for nonperformance, so this is one thing. We are tied up with many activities especially this year we have been going from one campaign to another, and hence we have not been engaged with the routine activities but iCCM is not alone in this, it works for all other activities.

Interviewer: So one of the reason is the extra engagement in the campaigns. What else could have been there as a reason not to sustain?

Respondent: the other is there must a responsible person who makes the trained mentors accountable for the activities. What I mean is the number of mentorship they make, who they met, and the findings they obtained—JSI used to evaluate in this manner and but this all needs hall to do that.

Interviewer: the trained mentors here with you at the HC, are they specifically assigned to support the iCCM only?

Respondent: Yes they are specifically assigned to support the iCCM service only, they have their own checklist and they used to support the HEWs on monthly basis.

Interviewer: is it interrupted now?

Respondent: No it is not. but they would not find the HEWs if they go to the HPs because they are engaged in a campaign.

Interviewer: so there is a separate checklist for iCCM?

Respondent: Yes. But only for the mentorship purpose. But the checklist for evaluating the performance and assessing other activities is an integrated one, but it is just for the mentoring that they have a separate checklist.

Interviewer: Ok even if there are challenges you mentioned, which activities do you think could still be scaled up at the national level and can easily be integrated into the existing health system?

Respondent: what I think is easy to implement is the treatment service at the HP since JSI-L10K introduced it to the community and has now been familiarized, so it will continue. The other is fulfilling the supplies from our side, the store man has already retained the list of the required drugs for the iCCM service, so the budget will be requested when they are stocked out and the HEWs will also request through HPMRR and they will get the drugs, so being able to do this is a big deal. The HEWs have now been accustomed to the iCCM service which they have been afraid of doing in the past, so this is not going to stop unless there is poor flow because they are not going to send them back if there is a demand. So these are three activities I think will continue. Similarly, the mentorship is also going to continue

since we have trained staffs and once they return from the campaign, we will send them for support. Since we also have the platforms to evaluate the performance in our PCHU meeting, I think this will also sustain.

Interviewer: what about the activities which might not be sustained?

Respondent: it is not that I think it will not sustain but what I think will slow down is related to the WDAs. Since these are the ones supporting the HEWs, and since the household to HEW number is not proportional, it is the WDAs that are helping the HEWs with this in mobilizing the community, alerting them when there are deliveries, and PNCs, so I think it might be difficult.

56. How are the activities/efforts embedded in the PHC and woreda routines/work streams?

Respondent: the first thing required to provide the service is training so the HEWs are trained on iCCM so it means the condition for providing the service have been fulfilled. The supplies needed for the service will also be availed by the HC and in order to ensure the implementation there HC-HP linkage will be used in which one supporter for a kebele will be assigned, it has also a checklist. When it is at kebele level most of what the checklist asks is about neonatal care, which includes about the disease classification, type of medication provided, diagnosis etc, so competent people are assigned to support this. Beyond this, there is also a quarterly supportive supervision integrated with all units and one of this is about the iCCM service delivery. So I think this has already been adapted.

- What implementation strategies are incorporated with the PHC and woreda annual work plan?

Respondent: the planned activities incorporated at the HP level include medication with ORS, and ZINC. I may have forgotten some but the treatment of diseases such as diarrhea and pneumonia have been incorporated, they have their own targets and performance which are evaluated on a quarterly basis at the kebele level, including the number referred.

57. Do you have anything think is important to tell us that we have not asked you?

Interviewer: Finally, if you have anything to add or recommend or suggest

Respondent: I won’t have much to say since we touched on most issues. But maybe what I suggest if it could be worked on is around the community engagement in strengthening the WDAs. I would suggest if JSI-L10K could do something about the WDAs in collaboration with the government, it would mean a big deal for us in the health sector. This is what I think should greatly be considered, and if we can work on this core issue the other implementations will accordingly be accomplished in line with this. Since JSI-L10K has previously worked a lot previously and if it can also work on this one as well. Maybe with regard to HC-HP linkage, and if there is a support to reimburse and cover the cost of the people when they are engaged in the activity. This is all I have thank you.

Respondent: Thank you very much for your time. You have given us a lot of information.

THE END

End line Evaluation of the PSBI implementation research; Full Transcription for an In-Depth Interview with a WoHO Child Health Officer

Questionnaire ID	06
Area Identification	Dembecha
Name of Woreda/Zone/Region	Dembecha WoHO/West Gojjam Zone/Amhara Region
Name of facility	Woreda Health Office
Name of moderator	
Name of a note taker	
Date of Interview	16/06/2022
Participant #	01
Audio File #	WoHO_Child Health Officer_21.6.2022
Start time:	11:00 AM
End time:	12:31 PM
Transcriber/Translator	
Duration of IDI:	1hr, 31 minutes

IX. Fidelity

58. Can you please describe the demand generation/SBCC activities you are doing on iCCM/ newborn health? What are the strategies used to raise awareness? If none, please explain why.

Respondent: Well, what the first situation was the community does not know that the newborn health care services were provided at the HPs, and unless it is for the services the HEWs provide on hygiene and others at the household level, the services were not known to them. However, after the consultative meeting we held at Debremarkos, it has become very strengthened, then what followed in the HEWs presence and also with the support from us is the awareness creation made for the community on the commencing along other services they provide of the newborn health care services by the HEWs mentioning that there are partners who support them, adequate medicines availed and that the services are also provided for free. After the awareness creation was made, then we discussed on what to do for children and mothers to come for the services which we discussed with the community at every available opportunity. That is what we implemented but before this was done, the activity was not that much.

Interviewer: if you can elaborate a bit more on how you implemented it, and the methods you utilized?

Respondent: Ok, after our discussion was held, public gatherings and general public meetings, churches were used, and also mothers when they come for vaccination service, were also advised that it is not just for vaccination that they should bring their newborns but when they feel sick too, and that the HEWs provides this medication service and can cure them, and including about the free service provision. Also, the advantage of avoiding long-distance travel was made aware; we, for instance, have one HP called "Addis Zemen" which is very distant—about 50KMs away from here but by the time we went to make a visit after the Markos consultative meeting, we found out that she had treated 8 sepsis cases, and this change was attributed to this service. When we also implement these activities, the leaders at the kebele level have also implemented a lot of activities in terms of renovating the HPs and making it comfortable; in creating awareness to the community about the newborn health care services, for instance, in places where we could not be present, they have been providing awareness about the commencement of the service, and it was after they did this and the other activities that a lot of changes were registered. When we also compare the performance from our regular monthly performance evaluation, there was a significant change.

Interviewer: How about in utilizing other methods like displays etc about the iCCM service?

Respondent: yes, a banner was prepared from the internal budget of the health center and posted at the HPs listing the type of services they provide, the time they are available, about the free provision of the service, including the address of the health care providers. These were all done at the time.

Interviewer: Was there any specific support you received to be able to do that, and how much did you also support the HPs?

Respondent: in order to implement this activity, there was an assigned person for this from the project Mr. Abebe [JSI project staff, the name changed] he visited all the HPs and revisited them three or two times; we have 31 rural kebeles and there was no kebele that he didn't visit three or two times, about 50% of them were visited twice and those located in close proximity were visited three times including myself. We also made the support having the assigned supporters from the HC, but you know unless it is for the slowing down as the supports also decrease in intensity at times, Mr. Abebe also used to receive reports every week, and we also receive the reports. Our report exchange also used to be on weekly bases.

Interviewer: You have mentioned earlier about a consultative meeting held at Debremarkos, if you can tell me more about it, what was it about?

Respondent: the consultative meeting was held in the attendance of the kebele leaders, all the HEWs, kebele supporters from the HCs, and it was communicated that this service has to be known and expanded, that it is also deadly—when an infant below 2 months of age dies, it is not even properly declared as death when it is actually possible to cure it. The service is provided for free with the support of partners that is outweighing the advantage for the community. As I mentioned earlier, we have a HP

located far from here about 50KMs and a mother used to carry and come all the way here, and the service has avoided this challenge and for this, we would like to thank JSI. However, regarding the continuity from our side, we didn't maintain as before due to different emerging tasks, but if we were able to maintain it, we could have treated a lot of children and saved a lot of lives but what was done is undeniably commendable.

Interviewer: so why you could not sustain the activities is due to emerging tasks?

Respondent: yes, it is due to the emerging tasks, and also the lack of partners to adequately make the performance evaluations just like other activities; it could be in every quarter or every six months since change is anticipated from being able to evaluate our performances.

a. How is the engagement of communities/WDA networks in the implementation of integrated community-based case management (iCCM) of common childhood illnesses/possible serious bacterial infection (PSBI)?

Interviewer: How about the participation of the community and the WDAs? How did they help and support you in the newborn health care service provision?

Respondent: Ok, the community participation especially on this, for instance, there is a HP called Gajira HP which the kebele spent around 60,000 ETB to renovate the HP including the inside structure; they have painted the walls, cemented the floors, built a fence which they didn't have before. They had their self-motivation to contribute something to HP and the money was collected from their self-initiation also by printing a receipt and collecting this 60,000 ETB. Other HPs have also made similar contributions, 50,000 ETB or 20, 000ETB depending on their size, and especially those remote kebeles were interested to do this since they don't have access, but the kebeles located near have different options to visit health facilities since there is adequate transport service, and other which they can move during nights or day time. Regarding the WDAs…

Interviewer: If you can be specific to the newborn health care service?

Respondent: ok the WDAs regarding the newborn health service provides information about sick newborns in the community, they also bring them, and communicate through phone.

Interviewer: Ok, how do you describe their support and their current contribution compared with the past also?

Respondent: Yes, when you compare it with the past it is diminishing. For instance, if you take one HP, there are 39 expected WDAs but not all of them are involved and maybe 2 or 3 might be properly engaged, so their role is diminishing from time to time. I was just telling you about the previous trend earlier, but currently, very few of them are engaged, and we need to strengthen it. If they are strengthened, not only this we could achieve many other activities. The newborn health issue for them means dealing with their neighbors since there are newborns; they know who delivered in the community and who is having a sick newborn, etc. They even send them to us—they would tell to them to go and check themselves when they are not even sick.

Interviewer: are you referring this to those currently engaged?

Respondent: Yes, it is for those currently active. If there are 39 of them in a given kebele, all of them won't be functional.

Interviewer: what do you think is their reason for not being completely engaged?

Respondent: they related it to financial issues, for instance, previously some organizations used to support them with umbrellas, bags, etc. they also used to call them sometimes for review meetings to the towns, but those things have stopped now due to policy changes, which it is not allowed to pay them anymore, and even if some organization want to call them somewhere and make payments to them, it won't be allowed since the activity is one which they implement in their kebeles', it is not possible to pay them money. You know these people are farmers, and I presume you also know how they live, and because of this they would say why abandon their household chores for this. So it is diminishing because of this.

Interviewer: is it not really not allowed to pay them for an organization if, for instance, it plans and wants to provide them training here?

Respondent: No, it is not possible. If there are no updates, the previous status was that since the WDAs are working in their own kebeles, and serving the community who selected them, they have to provide the service for free. They also relate this with budgetary issues.

Interviewer: Ok to make it clear, for instance, an organization may invite over people from HC to come here and provide them training, and there is also what the organization provides to the participants in the form of perdiem, are you saying that if in a similar manner it also calls the WDAs here and provides them training, that there is nothing it gives them and prohibited to pay them anything?

Respondent: No, it is not for training when they come here but it is when it is at the Kebeles where they live and work. So the thinking is that, if it is at the kebele, the WDAs are selected by the community and they have to serve them for free, and it is not allowed to make any payments in such cases.

Probe: What are some of the biggest challenges with SBCC activities for newborn care?

Respondent: one, there was shortage of materials at the time in relation to the SBCC activities. For instance, there were no materials to give education for the community, nowadays there is no challenge in having community who reads, and because of this, relatively better awareness is created if for a mother—some material like a leaflet—is read out for her by her student child and when he explains it to her than being addressed by some leader in public. So if this is considered in the future, it will have impact.

Interviewer: What else?

Respondent: the other is using audio messages. This is for instance for families who can't read at all. In relation to this, there was once a communication of message about the service using an amplifier that come along when vaccination was being provided about the child health, the danger signs and if there is a

tool that explains about the type of services provided for newborn health, it will help the community learn more than the leaflet.

Interviewer: You mentioned tool, what type of tool for instance? You mentioned speakers…

Respondent: it is around that and similar to that, for instance, regarding the vaccination service, there used to an audio communication using amplifiers of the type service describing about those who are to be vaccinated just after delivery, and those to be vaccinated after the 42nd day and such. Hence, if there is a similar mode of communication on newborn health care as well, it will help them understand more, than the leaflets since there might be people who can't read, considering also these people are farmers.

Interviewer: what do you need to do that?

Respondent: there is finance required in order to buy the amplifiers. But generally, regarding newborn health services, there are a lot of activities yet to be implemented. The activities commence by some partners but stop as soon as they phase out.

Interviewer: What is the challenge you have in order to sustain the activities by yourself?

Respondent: it is the shortage of budget. You don't have the finance to organize meetings and evaluate the performances just like the other programs. When you separately see the loss due to a sepsis case; you can imagine mothers before that have conceived their infants for nine months, suffered a lot, and when after 2 or 3 months she may have to experience some bad outcome, you can imagine the suffering, grief, and psychological impact she might experience. But it is possible to avoid these all if we can work on this service.

59. Could How do you describe the strength of the support you got from the PHC and the project as well as your efforts to address barriers to delivering iCCM/PSBI during COVID-19?
    Probe: What was changed?

Interviewer: Ok thank you. The other thing I want you to explain to me is the strength of the support that was provided from the HC, project or you to give the service by the HPs? How strong was the support?

Respondent: it was helpful for us at the time. As I told you earlier, Gash. Kebede [JSI staff, the name changed] has supported all the HPs; those far from here were supported two times and HPs that are closely located three times. So this is one of the support, and there is also our joint support as external people from other places come; there is our support, support from assigned supporters from HC, and also the support from the HEW coordinator, all these supports are available. So the support at the time has improved the service, and we were also comparing the service provided at the time

using numbers which a lot of newborns were treated and cured and a lot of mothers have also given their gratitude. I don't think there is anything worth this.

Interviewer: Ok if you can tell specifically the degree of support that was provided by the HCs to the HEWs since eventually it is them who are expected to give this service? Then you will explain your support.

Respondent: the support given by the HCs might not be specifically for the newborn service alone, but they might do it integrated while going out for the other services. It is also similar with us here, and there was no a separate support made targeting only the newborn health services, that I didn't do personally and also as a department. But when I go out for other services, I might as well see this service; I will observe the registration and check the number of sepsis cases in that week, or within these two days or in the month. It is also included in the supportive supervision conducted every quarter incorporated in the supervision checklist, and the activities are evaluated and supported accordingly. That was the support provided.

Interviewer: regarding the checklist inclusion of this service, is it possible by everyone to support using the checklist or is it specific to you only?

Respondent: regarding the iCCM service, it is available and included in the checklist. So we pay visit to the HCs every quarter, and the service is also incorporated in the checklist so every officer will go out and assess the HCs for iCCM service as well.

Interviewer: Do the HCs provide a form of support called PRCMM?

Respondent: yes, they had that support at the time but not it is halted. I will be telling you what was done since I have to be frank about it.

Interviewer: Ok what was implemented at the time, back then?

Respondent: at the time, the number of newborns treated in a week used to be cross-evaluated, and also in every fortnight, the HPs used to bring their registers to the HCs to share the experience with one another for gaps that one might make while providing newborn health care service since one HEW could learn from the others in the making. Hence, every HEW will come with their register, and be given a case scenario or might work on a real case if there is any, and then they discuss the gaps they made and provided with a recommendation to correctly treat using the treatment guideline. It is undertaken in the manner that I will have your registration and you will have mine, that was for the support provided in every fortnight.

Interviewer: so you are telling me the past experience?

Respondent: No I am telling you what was present in the near past, we have been doing that until very recent time but now it is interrupted and we didn't sustain it par with the previous intensity.

Interviewer: What else? What other type of support they provided? Was there a form of onsite support at the HPs provided by the HCs?

Respondent: yes, there was an onsite support provided by the HCs at the HPs by looking at their registers; whether they are following the treatment guideline to give the newborn health care, and also with regard to drug management and proper request of the drugs because of the impact on mothers to deprive them of the service and going back without the service due to stock out of the drugs, and since the mothers might also communicate undesired messages to the community about the unavailability of the services. Hence, the HCs make observation of their drug storage condition, management and proper requesting. If they are also not providing care according to the treatment guideline, they demonstrate them onsite.

Interviewer: Ok, what about the effort from the HEWs to implement and provide the iCCM service?

Respondent: the effort was, well initially the available registers were the old one but after the markos consultative meeting we have received the new one. The only difference with the HC is that it was the Amharic version whereas it is the English available at the HCs. It was instructed support to be provided for the HEWs by the under 5 health care service providers, and it was also mentioned at the time that they had gaps and they were not treating using the treatment guideline and also they were using the old. Then the new register was provided to them, and the health service providers working on under 5 from the HCs also started supporting them going to the HPs, training was also provided to the under 5 health care providers to be able to support the HEWs.

Probe: Was the support you got from the project and PHC helpful?

Interviewer: How do you see the support in general, about the strength of the support? Was it helpful, or did it help you?

Respondent: the support has helped but it had no continuity. However, had we been making the support jointly with the same intensity every week, and we might also make specific support for the newborn health or sepsis from here, but if that was continued with the same intensity, a lot could have been achieved.

Interviewer: the reason for that you told me is related to emerging tasks?

Respondent: yes there is an emerging task, there are budgetary challenges, vehicle challenges, etc, if for instance, I wanted to go to some HP, it is something possible since there is a transportation challenge. So such things may limit the continuation of the activities.

Probe: What could have been done differently?

Interviewer: what is that you think could have been done differently about the support?

Respondent: what I think could have been done better is, if we were able to be part of the support from the start and jointly sustained the activities just like the degree of intensity observed at the beginning, then the professionals would have also owned it and made it a trend. This is one, the other is the lack of full working hours opened for the services at the HPs mainly related to the current situation in the country, especially in the farthest HPs. The other is, that we have been receiving commendable

support from Gash Kebede [JSI staff—name also changed] for a long time by giving due attention probably better than us but at some point, there have been some gaps and interruptions.

Interviewer: what was the reason for the occurrence of gaps and interruptions?

Respondent: one of the reasons is being overloaded by emerging tasks, and the second is budgetary issues which presents you with a challenge to organize meetings to evaluate performances; the number of HEWs is too much, and when you plan for Sepsis, you also need to call the under-fives, etc and because of that we could not cascade it.

60. Do you think the support system is helpful (mentorship and on-site coaching methods, supervision, technical support, PHCU level PRCMM, etc.) to enhance your skills [that is HEWs' skills] over the traditional off-site training and woreda level review meetings?

Interviewer: generally, the activities that you have been doing like the review meeting—I don't know if you ever manage to do that, but also including the onsite supports, the PRCMM you mentioned to me, and when you also compare these supports in general with the conventional type of supports like the offsite training, how do you think your support have contributed in improving their skill, and how much it contributed?

Respondent: in relation to improving their skill, it helped them a great deal. Well you know one of the services which the HEWs are known for is newborn health care, and unless it is for the newly assigned ones, if you take one senior professional working on under 5 at the health center and a health extension worker for newborn health care you won't find major difference in terms of identification and following the chart booklet to treat a newborn unless it is for the English and Amharic version of the booklets, so a marvelous work has been done in this regard. And in the future, if there is any partner that can support this, the onsite support can definitely help. The onsite support helped since they also already reported about the gaps they had at the meeting and since it has been provided to them several times now they are doing their work similar with the health centers unless for the new ones maybe.

Interviewer: what changes have you observed due to the onsite support for instance any specific skill the HEWs developed? What specific skill is there that you say has developed?

Respondent: the skills developed with regard to the number of those treated newborns have increased over the year, and the other is in terms of being self-aware and implementing the activities—there is a better performance in terms of these things.

Probe: for acceptability of the strategies **(for HEWs only)**. How could the support system be improved?

Interviewer: How were the HEWs receptive of the support provided to them?

Respondent: they were very receptive and happy to get the support, one HEW even mentioned once that she felt like she was in a school that day when Gash Kebede [JSI staff] came to support her—yes it is like a

school if it reminded her what she forgot and filled the gaps she had on the treatment guidelines, it is like learning in her house. They have got a lot from the onsite support and if there is more attention on newborn health care.

Interviewer: that was my next question, how do you suggest can things be further improved? What should be done to improve newborn health services?

Respondent: well after every discussion what we rest on is budget, so if there is a budget and the activities can be reviewed every quarter. As you know, there are also no partners who are making separate support for newborn health care while there is a lot to be done, and I recommend that something is done about that.

Interviewer: But if there is anything you suggest about improving the skill-related issues?

Respondent: the other is that there are always new professionals being assigned, sometimes all new ones may be assigned at once in one place, and if there is training to be given to them. I know they learn these things during their preservice school but if we can manage to provide them training.

Interviewer: ok, now these activities were being implemented to support the newborn health care improvement, but have there been any activity implemented considering COVID and its possible impact in hindering the newborn health care service delivery? Any intervention on newborn health improvement but also considering COVID?

Respondent: well what was implemented considering the occurrence of COVID is that we used to give education in meetings, at health centers when people come for medical attention even if the flow at the time was minimal, we used to tell them to come and bring their newborns for treatment applying the protective measures. We tell them not to refrain from coming for fear of the pandemic.

Interviewer: Who was implementing this activities?

Respondent: it was done by the HEWs, also by us and even if to a small extent also by the WDAs, including the leaders. The services not only at the HP, it was also interrupted at the HCs too for more than 2 weeks, and it was also a time where the professionals were also scared of providing the services. However, as we get accustomed to it, things start to improve. We used to give awareness about the services including the child health care services and that it is still being provided and not interrupted at the HFs, while also providing them education on the protective measures like on application of face masks etc. They [the community] were advised and consulted with the health professionals for any health issues they might have.

Interviewer: Was there changes to your support strategies considering the occurrence of the pandemic? That is tailored to COVID? Anything changed for the HEWs support system?

Respondent: there was some things they changed [the HEWs], which they started to make the house-to-house visits, and the visit was also a bit more considering the pandemic. They used to tell them not to keep

away from coming and about the availability of the services. Our visit was also increased in frequency at the time since we also need to provide education to the community.

61. How eCHIS implementation helps you with iCCM service delivery?

Probe for advantages:

- case identification? quality of iCCM case management? retrieval of client records? data quality? client appointment scheduling and/or defaulter tracing?
- **Ask why?**

Respondent: regarding the eCHIS, it integrated not only the iCCM service but also other services such as house-to-house registration, delivery, family planning, and vaccination, and it incorporates all activities. But there are still hardcopies that is yet to be implemented in the future, and even if the system is under pilot stage and a lot remains to be done, we can however observe that the system is good and achievable but they have not finished recording all the households yet. But if they able to continue with it, it will have significance to avoid the junk paperwork you see for the services, except for the iCCM service registration and chart booklets or the treatment guideline which I think might not change, all the services will be integrated and will have importance to also provide the newborn health service; for instance, if a mother delivers it means the newborn will come and identified or labeled using the register. But this is yet to be implemented and on progress.

Interviewer: How does it help in the case identification? Can it help with that?

Respondent: for instance, if a mother delivers, the HEWs will know and visit the mother. If the WDA knows about it and if there is an illness she will refer her to the HP. The HEWs, even if everything is normal, will provide her education on at least breastfeeding, that is why it is advocated that every newborn should come and be identified. If it is known that a mother has delivered…

Interviewer: How do you know that a mother delivered using eCHIS?

Respondent: for instance, everyone in a household is registered in the eCHIS and when there is a new birth, it will be updated which means the newborn is available in the system.

Interviewer: Ok how would it help in ensuring the quality of case management if the iCCM service is integrated in the eCHIS?

Respondent: well it is my thought that it might help, this is because I could not say anything for sure on something that has never been tested. But I think it would help if it is integrated since it is an online system which might have importance for drug stock out etc. Since it is interlinked from higher to lower levels, it might indicate something. But still, it might be difficult for me to state its advantage or disadvantages at this stage of the implementation.

Interviewer: How about for easily accessing client's records?

Respondent: it is very helpful for that. What this means is for instance there is a pregnant mother and then she delivers, then after the 42nd day of her delivery, there is something the system notifies her [the

HEW], which means it is important for PNC and also for iCCM because it is flagging her in the system, which she can be reminded to check for newborn health as well.

Interviewer: What about for data quality?

Respondent: it is also similar to the data quality as well. But maybe if we also forward this issue to the officials' attention, and if partners are available and you after this assessment can also work out on this, I think it will be more appealing.

Interviewer: regarding scheduling or defaulter tracing etc, can it help if iCCM is integrated in the eCHIS?

Respondent: well what it is generally thought about as I told you earlier is to incorporate all activities in the system, and the newborn health care is also included in this, and hence since it is included, I think it will help, that is why it is also the software is developed this way.

- **Probe for areas of strengthening?**

Interviewer: How can the system be strengthened more? So far from the challenges maybe you heard from the HEWs or something you observed etc?

Respondent: what the HEWs raise is that they say it is troublesome and exhausting to use. The reason for this is related to the challenge of lack of easy familiarity, and the technical inability of the HEWs to easily manipulate the instrument. I have seen them use it when they were recording and I don't think the technique is that difficult unless it is for fear of engaging in the activities by the HEWs. But what I heard them saying was that the technique to operate has been difficult for them.

62. How was the introduction of the iCCM/CBNC module into eCHIS affected the iCCM/CBNC service delivery?
FOR LUME WOREDA ONLY extra

X. Implementation challenges

63. Describe the main issues faced by the health system to identify and treat neonatal infections in the community. What are the critical factors affecting the delivery of iCCM to clients?
Probe:
- lack of competence of HEWs, shortage of supplies and commodities; weak support system; low community demand?

Respondent: ok we can raise many challenges on the implementation. The health care service that we provide to the newborns here is similar to what we could provide at the hospital; maybe we might provide them IV at the hospital and here IM which may be the difference. Hence, if it is required to continue the program, benefit the community, and considered eradicating neonatal death, first, a senior professional should be assigned to work with them; this is important for those newly assigned HEWs which even becomes difficult for them to insert needles in the arms of infants relatively thinner. I think there might be something considered in this regard in the future. This is one, the second is the budget for supplies needed to consider the HPs, for instance, the supplies that are availed for the HPs are purchased from the HCs budget, and there is no budget allocated for the HPs supplies. The

HC is not complaining and is supplying the HPs from their goodwill considering it is still serving its catchment population. But the supplies provided to the HPs are utilized for free, and if the HPs manage to treat many newborns the HC might be bankrupted. However, if we need to expand the service, it should come in the name of the HPs directly and a way for them to utilize them on their own to provide the health care service. The other is that there is a need to have a meeting for the HEWs to evaluate their performance at least every quarter since there are HEWs who have a lot of gaps and need support. The other is also the renovation of the HPs and the need for better-conditioned HPs which are clean to provide the different services and undertake different procedures like injections which should be made in a clean environment.

Interviewer: OK what about the community awareness level about the iCCM service? Can we say it's fulfilled 100%? Is there anything that remains?

Respondent: there is what lacking from the HEWs side and also from the community. From the HEW side they have to spend their full time providing the stationary service at the HPs. For instance, if there are two HEWs, one has to stay at the HP and make the services open while the other makes the field visit. What remains from the community is that, well you know there is an obvious shortage of supplies and when the community didn't get the services for this reason, they might implicate it as if there is no service provided at the HP. So there is a deficit from both sides; the HEWs in terms of not using their time effectively and the community with lack of acquiring the expected level of awareness to the extent we provided them.

Interviewer: Ok this is about lack of awareness. Is there a sort of making comparisons between the quality of service between the HP and the HCs and inclining to one?

Respondent: yes, they make comparisons and say if they go to the HC they think drugs in large quantity; it is human nature and reflected by all of us, that when there is a service given to us for free and when there is another one we pay for, we usually think those services incurring costs have better qualities. So a lot is expected from them and from us as well.

Interviewer: What else for choosing the HPs over the HCs, any other reasons?

Respondent: There is no other reason, it is only the gap in opening the services for 24 hours and waiting for the community by the HEWs, and maybe if there is a shortage of supply and for this reason, they are sent back. The other which I forget to recall is the transportation challenge, for instance for a mother, residing in one of the distant HP, a referral is needed to treat her newborn, ambulance might not come for her. This was actually started right after the consultative meeting but because of the current country situation about 3 or 4 of our ambulances were sent to the war front, and one of them had an accident and was left out there. So currently we don't have ambulance access and hence if we are not able to refer a mother when she requires an ambulance for her near newborn they might get disappointed. In the future, there are things that we need to do. I think now some of

our ambulances that were under service are not out and ready which we also need to inform our superiors.

Interviewer: How about the skill and competency level of the HEWs? Is there any gap concerning that?

Respondent: most are doing a marvelous job to a level almost similar to the HCs but there are skill gaps among the newly assigned HEWs, and also in relation to individual variations in the understanding level of the HEWs. In terms of knowledge, well it is also relative.

Interviewer: what about the intensity of the support, you early on mentioned that it is not like before?

Respondent: yes, well what is to be noted here is that this year we have, most of the time, been engaged in conducting campaigns, such as there was polio, COVID vaccine which we have given in three rounds and also azithromycin. Hence, we have spent most of the time in routine activities as before. The HEWs are still available in the community but the HPs in most of the time were closed because of the campaigns.

Probe:

- Regional/national state of emergency and conflict in the northern part of the country?

Interviewer: what kind of influence did the conflict in the northern part of the region have on newborn health service delivery?

Respondent: well one of the challenges as I told you is related to the supply shortage that is associated with the war, for instance, one health center has contributed at least 50,000 or 60,000 apiece for the war, and as a result, they have now no finance, especially the rural health centers don't have any finance since there are also other factors such as the exempted services, free service for the needy ones, and because of these the HCs are not profiting.

Interviewer: What is that you mentioned 60,000?

Respondent: it is the medicines they contributed and sent for the war, which they could have supplied to the HPs to support the newborn health care service and due to this challenge the HCs are not sending anything to the HPs.

Interviewer: what kind of medicines that are related to newborn health care are sent to the war?

Respondent: I am referring to the support which they could have made in terms of the budget, and it was not to say the medicines intended for newborn care were sent to the war. Regarding the ambulance challenge, it is what I stated early on. Other than this everything is stable and at peace here since by chance this area is not where the war is taking place unless for displaced mothers who are residing here due to the war. And, the war also did not have a direct influence on the provision of newborn health care services.

Interviewer: was there any challenge to treat newborns of internally displaced people?

Respondent: they treat them at the health centers for free whether it is for the mothers or the newborns.

Interviewer: any impact because of the IDP situation or to provide them the iCCM service?

Respondent: the influence it created to us is in relation to the transportation challenge to reach the community; there is an ambulance shortage and had it been with the three ambulances as before, it would have been great but now the available ambulance we have is not adequate to serve around 175,000 populations we have.

Interviewer: But this is about the general challenge, my question however was related to the IDPs. Any challenge to provide the iCCM service to the IDPs?

Respondent: So far there is no one from the IDPs who complained about not getting the service [iCCM service]. We have been informed by the woreda administration through an official letter to provide the services for the IDPs for free, accordingly, they are also getting the services for free and there is no problem.

Interviewer: you have the extra budget for that?

Respondent: Yes, but even if we don't have the budget, the woreda administration has subsidized by allocating an additional budget.

Interviewer: What about the state of emergency and movement restriction etc, was there any influence it has caused for the newborn health care service delivery?

Respondent: there was nothing.

- How has that changed during COVID?

64. How do you think the COVID-19 pandemic and/or COVID-19 response measures affect the uptake and/or delivery of iCCM services?

Probe:

- Has COVID-19 affected your daily routines; your work on newborns; the community in terms of livelihood and vulnerability for newborn care-seeking

Interviewer: ok now if you can tell me elaborating more from the beginning to the end about the conditions which the occurrence of COVID pandemic or the response measures affected the iCCM service delivery or uptakes? Beginning from the health professionals, the community side etc.

Respondent: at the time there was fear, we ourselves were also not engaged in our routine day-to-day activities—there is no point in lying about it. I myself for instance in a cluster that had about eight HPs and other than maybe coming to the office I have not really been engaged in anything. It has a lot of influence on all of us at the time but as time passes by, we become familiarized with the situation. Even if the number we have been hearing from the different media outlets, especially in other countries was high, the severity relatively seen in our country was insignificant—even if there were people who died because of it, and especially the number of cases in this region was also low. In relation to this development, people then gradually start to engage in their routine activities. However, at the beginning especially in the first month, activities including the field level activities

were completely halted, and it was only to the office that we used to come—this was even done because we were in the health sector, other sectors, however, have totally stopped.

Interviewer: Ok what about challenge when the HEWs want to go to the community to do the house-to-house visits or any activity in the community?

Respondent: it was actually the same in the first month and there was a gap at the time. In the first month, even the HCs were also not somehow closed and not giving service. But when I explain the influence in general starting with us at the bureau and for the activities I used to execute during the normal days, I didn’t do anything or anything to support the newborn health care service because of fear of contracting the virus.

Interviewer: For instance, which activities you didn’t do, which you used to be doing?

Respondent: if I give you an example, I have not visited a HP and assessed the status of the performance by observing their register and treatment guidelines, which I would have on normal days. It was also the same for the community challenged to come to the HPs for fear of contracting the virus. They had a lot of fear at the time since it was communicated that the virus transmits through breathing or contact with surfaces but gradually they started to make improvements as we have also been providing them education and awareness to apply the protective measures to come for the services.

Interviewer: Ok what about the influence to use the iCCM service with regard to applying the COVID protective measures and coming to the HPs?

Respondent: Yes, there was an influence, especially in terms of using the face masks, the community complains that it suffocates them or bruises them, and some also hypothesize that why they are told to wear the face masks by us is to intentionally put the virus inside the face mask and deliberately expose them to it. But gradually they get used to it and have been wearing and obligated to apply the face masks to enter the HPs, and a handwashing station was also availed at the HPs to wash their hands, however, as soon as they leave the HP, they take their face masks off.

- How has that changed over time?

Interviewer: How did the situation with COVID change over time? And, what is the current status?

Respondent: how it changed is that when they see the people in the towns, and those who have children living in the towns providing them awareness about the protective measures like covering their nose and mouth with their cloth if they like, etc, and when we also aware them, they start to become accustomed.

Interviewer: Was there a sort of problem in affording the cost of these protective materials and avoiding coming for the services?

Respondent: there was not a challenge mentionable to this extent. It was around 10 ETB that was used to be sold at the time.

Interviewer: what is the current situation? Is it burdening the services?

Respondent: the COVID case is still being identified but the community does not believe it though. They will tell you COVID is not major challenge, it is rather the high living cost that is challenging their survival and not COVID, if you ask them. This is what they were telling us during our vaccine campaign.

Interviewer: How is COVID influencing you not to properly provide the iCCM service in the community, in the current time?

Respondent: currently there is nothing that COVID is influencing us not to provide the newborn health care services. The community, at least 50%, has awareness about the disease, and they are applying the protective measures. They put on face masks when they are visiting the HCs or HPs but as soon as they leave they take it off and put it in their pockets.

XI. Adoption and reach/effectiveness

65. Explain to us any changes you observed as the result of the implementation of the COVID-19 adaptive iCCM implementation strategies? – What impact have the strategies had?
    - What are the particular features of the strategies that made a difference?

    Probe:
    - for a support system and linkages, motivation, and competence of HEWs, community**,** and awareness creation?
    - What do you think are the reasons for non-significant changes?

Respondent: there were changes, for instance, the first activity that Gash Kebede [JSI assigned project staff] did was provide awareness for the community gathered for the mosquito bed nets distribution, which he told them about the existence of the COVID, and hence the need to protect themselves, and while protecting themselves that they have to bring their newborns to get the health care service. He told them that they don't have to stay at home for fear of the virus knowing that they have a sick child in their hand. He added the services will be provided and the HPs are also open since the health professionals are also wearing face masks so that they don't have to worry about contracting the virus. He communicated this in a strong manner to the community and after that, there were some changes observed.

Interviewer: my intention was actually for you to tell me about the general changes observed such as the supports you provided, the HC provided as a general strategy, and the changes observed as a result of these supports. If you can tell in this manner, what are the changes attributed to these supports?

Respondent: ok, the changes were that one the number of patients increased, the reporting system improved, for instance, before four months ago we started a weekly reporting for Sepsis, also for nutritional screening which commenced with Gash Kebebe [JSI staff], and we managed to sustain this to date, just like the time we started and still we have a weekly reporting that is every Thursday which the woreda reports to the zone and the zone to the region. This is going in a strengthened manner and now we also ask why we don't see Sepsis included in the report; anticipating whether it is related to drug stock out, or whether they were not available, etc. the other is it also makes the HEWs be

recognized, as clinicians in the community; you well know that every health care worker who wears a white gown is considered as Physicians and so they were addressed as Doctors since they were providing them drugs, and also because they were able to give medication and cure them that they were recognized as such. The other is also even in improving the HPs' physical conditions which were in bad shape, but it was after the support that the fences were constructed, their walls painted and banners were also prepared with a list of the type of services even if the WDAs didn't have any part in this.

Interviewer: Ok, among the supports you mentioned earlier, which do you think have made a difference and the most impact?

Respondent: one of the support was the one in which the HEWs bring their registers to the HCs every two weeks, and the manner in which they were able to identify and work on their own gaps has made a difference and brought a change.

Interviewer: what type of change did it bring?

Respondent: it improved their skill. If I am able to learn from my mistake from the experience of others that is a big benefit, and as a testimony, we also didn't see any gaps in providing newborn health care based on the treatment guideline by them when we go to the HPs, except for the new ones. This I think is a big change, and it is the same delivery of care with the physicians that they also use the same treatment guide except for the HEWs' guideline being written in Amharic, that is the only difference. If a mother in a given kebele can get health care service for her newborn for free is a big change. Another change is with the community having the willingness and ownership to consider the HP as his and contributing to the renovation of the HPs is also another change. The treatment guide is also a shortage in other woredas but we have got the support in getting the revised guidelines while there are some that do not have any. The reporting of Sepsis on weekly bases which I mentioned is also another change that we have taken experience after the commencement of this project which we have been doing every month before.

Interviewer: Are all the supports you mentioned related, I mean to which support type does the change in the reporting and the guideline availability, belong to?

Respondent: they are related to your support [JSI support], that is after the project implementation for one year. If you have not supported us with the guidelines we would have the same status with those woredas that still don't have it. Those woredas that are not supported are still working with the old guideline so what I want to say is that it has brought change to our quality.

Interviewer: is this change because they are supported more frequently or is it due to the training or what?

Respondent: it is due to the training and also from the regular support which is expected when you are frequently supervised, which in a similar manner might also go back if there is no regular support.

Interviewer: Is there anything you can tell me about the advantage of the support in improving your linkage with the HCs or the HCs with the HPs and also in increasing the motivation of the health care workers?

Respondent: the support level has increased especially on newborn health care service in the past year was implemented with due attention. Unless it is for the challenges with transportation and the current issue that I was mentioning but the activities with regard to the newborn health services were being implemented with a lot of attention in the last year, which is incorporated in the checklist and the weekly report. It was because the service has gained attention that the service has shown improvement.

Interviewer: Can we say the community awareness has improved with regard to newborn health services?

Respondent: yes, we can say but for the community not to fully rely on the HEWs to treat their newborns, there are still some gaps in the shortage of supplies seen at times and still not completely resolved.

XII. Maintenance and sustainability

66. What are the high-level benefits that are attributable to this support/IR?

Respondent: among the benefits the project gave us, we can mention a lot. One of these is the awareness creation in the community and especially the participation of the kebele leaders in the awareness creation effort has brought a change. It is not the same when a kebele leader tells the community something than when I am making it in terms of acceptance. He is the one who has the acceptance. The training was also given to the kebele leaders; they were trained at Markos as well as by us after we get back from training, so this is another change. The other is, it also filled the skill gaps of the HEWs; if I am not repeating this Gash Kebede [JSI project staff] has also supported on-site and filled their gaps intensively, so such supports from the project has helped us to give focus for the activities. it enabled fill their gaps and I suggest if the project continues in the manner it was provided, I think we can save a lot of newborns. The participation of the kebele leaders, and also the community engagement in renovating the HPs considering it as part of their property, are some of the benefits we got from the project.

Interviewer: So these are the high-level benefits that you have got from the support?

Respondent: yes, they are.

67. Please explain to us the feasibility of this support/ IR for national scale-up?
    Probe:
    - What features could easily be integrated into the existing system? Which not?

Respondent: well what I consider to be scaled up is the introduction of the services to the community –which this project has done a lot for us in this respect. The other is the preparation of the banner by listing the type of services provided by the HEWs, which I think is good if scaled up that is the experience in using banners for awareness creation purposes should be practiced. Another aspect is the engagement of the community to contribute to the construction of fences for the HPs and

renovation considering the facility as their own is also good if scaled up, and if they even further believe in the significance of the HP to them, they might even construct additional blocks if they are convinced.

Interviewer: now what I want to tell me is about the activities that are possible to be easily integrated into the existing health system without requiring any special support from any partner?

Respondent: frankly speaking there is no activity that the government alone could implement. It might gradually be implemented but if we need to sustain it for the long term there is a need for this project to continue the support or it could be other partners. The newborn health care service is not something that you implement on its own like other activities without the collaboration of other parties since it requires supplies, budget, transport, and the government or the health sector alone will not be able to sustain it by itself. The cost of the drugs is increasing and if one day the HC becomes bankrupt; it might refuse to supply the drugs to the HPs. So we need collaboration if we really need to reduce neonatal death and benefit the community, and the need for partner involvement is a must.

Interviewer: If you can tell me why you need the partners for sustaining the activities? For which activities do you require them?

Respondent: one of these is the supplies for providing the care, our HCs are in deficit in the name of the health insurance. Our farmers are also paying 300 ETB apiece for the health insurance but they are still paying 30-40,000 thousand ETB for referral purposes going as far as Black Lion hospital to get the services. So we need to be supported through supplies. The other is the support to review our performances through a review meeting, this is especially important for those newly assigned professionals.

Interviewer: For instance, you mentioned the PRCMM support at the HCs. Do you think the support is something that can easily be integrated into the existing system?

Respondent: It still needs support. For instance, we used to go into the different clusters at the HCs, and when they are making this activity that is every two weeks. However, due to the different competing priorities and activity load, I could not be available as before If there is specific support just like Gash Kebede [JSI staff].

Interviewer: Ok, so if the support from different partners is required to this extent to integrate the supports into the existing system. How do you think it is possible to sustain the activities without any partner support since projects might not always be around?

Respondent: you are right project does not last forever, but it will show the way to some extent. But what I am saying is that the activity might continue just like other activities but it might not have quality.

Interviewer: What are the activities you are implementing or strategies targeting the sustainability of the activities with regard to the newborn health care services?

Respondent: there is no separate activity we did for this service only. But we are trying for all services we are providing to the community to have quality and continuity. However, when we gather the community for different reasons, we provide them education, and even when they are coming to the woreda for different reasons as well—even if we don't provide them the education separately for the newborn health services but along with the other services about caring for the HPs as their own and to bring their newborns for treatment, etc. This is how we are trying to ensure sustainability, other than this, we don't have a different sustainability plan for it alone.

68. How are the activities/efforts embedded in the PHC and woreda routines/work streams?
    - What implementation strategies are incorporated with the PHC and woreda annual work plan?

Respondent: iCCM is integrated into our routine activities, and one of these is in the plan of course. There is an annual plan in which the iCCM service is included and evaluated on quarterly bases when we meet along with the other activities. We also receive the report every month. So it starts from the plan overall. It is also evaluated when we go out for supportive supervision as I told you earlier also included in the checklist, there is a separate section for iCCM/CBNC in the checklist. This is the integration in routine activities.

Interviewer: What are the activities included in the routine activities for the iCCM service?

Respondent: the plan has Sepsis, vitamins, deworming, and mass screening—even if this is not directly related to the newborns.

Interviewer: What about the plan for the implementation strategies and activities for the iCCM service?

Respondent: we make a supportive supervision every quarter for all services including the iCCM service. Other than this there is program-specific support which means I, for instance, go to the HPs, I will make separate support looking at the registers, I will look at the number of care provided for the month or quarter depending on the time.

69. Do you have anything think is important to tell us that we have not asked you?

Interviewer: Lastly if you have anything to say or want to add not addressed in our discussion.

Respondent: lastly, well maybe I said it repeatedly but there is a lot of work that is to be implemented on the iCCM service, that has not been touched yet. So we have to collaborate to do these activities, and I know this is going to be repeated but we are still going to raise the budget issue, and if the supplies are fulfilled. As I mentioned to you early on, if there is also a senior health professional with better qualifications than the HEWs assigned the HPs who they consult with since they are providing treatment, and hence if there is a senior professional—those with special training and who can be assigned at the HPs permanently to support the HEWs. I also think the activity should not be left to the government only, but if we can get the partners' support and achieve better results in the future to provide quality newborn health services and reduce neonatal death in the community.

Interviewer: I thank you very much.

THE END

End line Evaluation of the PSBI implementation research; Full Transcription for an In-Depth Interview with a Health Center Head

Questionnaire ID	02
Area Identification	Dembecha
Name of Woreda/Zone/Region	Dembecha WoHO/West Gojjam Zone/Amhara Region
Name of facility	Health Post
Name of moderator	
Name of a note taker	
Date of Interview	16/06/2022
Participant #	01
Audio File #	HP_HEW_16.6.2022
Start time:	09:00 AM
End time:	10:20 AM
Transcriber/Translator	
Duration of IDI:	1hr, 20 minutes

XIII. Fidelity

70. Can you please describe the demand generation/SBCC activities you are doing on iCCM/ newborn health? What are the strategies used to raise awareness? If none, please explain why.

Respondent: we have implemented a lot of activity in this regard. One of the activities is that we have called the WDAs and provided awareness, also provided awareness for the kebele leaders, and religious leaders at churches. In addition, we have prepared a banner listing the services we provide and posted it at our HPs. After we came back from the training the first thing we did was provide aware the WDAs, and we made it be passed to the 1-5 network, since it is thought it could be passed on to the community through the 1-5 network. Then after that, it was the leaders in which we were both available, set a schedule and provided the training where the leaders were present—the training objective is to make them mobilize for us. In addition to this, we have also provided awareness at churches for the faith-full about the neonatal care we provide at the health post. It is actually previously known that we provide health care for under 5 but now we were specific on neonatal care from 0-2months. In addition, as I said early, we have prepared a banner and displayed it with the kebele budget listing the 17 services that we provide including the iCCM/CBNC service.

Interviewer: if you can tell me how you were providing the awareness and mobilization with the kebele? If you can tell how you do this in detail, and the acceptance from the community?

Respondent: the first was the kebele leaders, and then in collaboration with the kebele leaders we aware the WDAs and then we make a home to home visit which the WDAs aware the community and also through the 1-5 network which they addressed the people and neighbors close to them. Then we all went to churches and provided awareness in shifts and also at vaccination centers, well the vaccination centers were what we utilized the most.

Interviewer: how do you find the WDAs to provide the awareness? How did aware the leaders first? Is it through a one to one meeting?

Respondent: No we gathered them at once, we and about 24 leaders met first and informed them that we are trying to aware the community and discussed on how best the community could get the awareness. Then it was agreed that it should be made through the WDAs, which we then gathered about 27 WDAs and made to reach the 1-5 network. Other than this, we were present in every vaccination center and there was not a time we send them back without providing awareness for mothers.

Interviewer: How do you do it at the vaccination sites? Is it through oral communication?

Respondent: Yes, we tell them verbally, and not with some sort of leaflet. Other than this, there are also children who come sick and since we always have the kits in our bags we treat them on spot, so these people will also serve as a proof and testimony of the service when we meet during the vaccination, who tell us and everyone that their children got better because of the treatment we gave them.

Interviewer: what else have you used to provide awareness? You said banner were used, how did you manage to prepare it and what was the support?

Respondent: it was the kebele that prepared the banner for us spending about 300 ETB, it was not us. But after it was availed, it was not easy to carry it around because it was heavy. Even if we couldn't take it to churches and other places, we did however manage to avail at meetings, and we read them out loud the listed services we provide which include the vaccination, family planning services, not only the health care service.

Interviewer: What else? For instance, have you used a banner to provide awareness for identified myths and miss conceptions circulating in the community about the service? Instead of just listing the services.

Respondent: well about that, there is what they call is "split-up of the head" [YERAS MEKEFEL], and they go to a monk to put on some herb. We have made most to give up this practice.

Interviewer: Can you tell me more about it? what it is about?

Respondent: well it is that, is it not the infants' fontanel open? Then they touch that fontanel and think that their head is split in half and will remain like that and die. We have actually identified many were using this practice but later we provided awareness and most mothers have now abandoned the practice, the kebele leaders were also present while we were providing the awareness. She [the traditional

healer] has now increased the price to 100 ETB, she puts on the herb for three days and received 1000 ETB for that. We have provided the awareness and now most mothers are not going. Other than this, most mothers also want to come to the HC than visit our HP, unless we make a home to home visit or for some villages near to the HP, most prefer to go to Yechereka HC or Dembecha because the place where the HP is located does not consider the distribution of the community. If they go to the HC it will take them only 30 minutes but if they wish to come to the HP it will be more than 30 minutes. The community residing near the asphalt road also prefers to use the nearest HC than coming to us. Hence, we also made an awareness creation to the community cognizant of the challenge, we told them that we can provide neonatal care at the house level, and now they call us when they detect illnesses or redness in the newborn's navel area.

Interviewer: Ok what the method you use to aware the community about the traditional healer, or about the house to house level treatment service you provide as an option?

Respondent: it is on the meetings we facilitated and the different stages. For instance, when we gather the kebele leaders, the WDAs and when reading the banners, we tell them a mother who just delivers and could come that we can come to her house and provide medication. Now there are not people who don't ask us if we have zinc or ORS since they understand we have those in our bags.

Interviewer: do you regularly meet? Do you get that chance to meet on a regular basis?

Respondent: Unless it is for the WDAs meeting, regarding the leaders it is on their own terms, which we then utilize that opportunity. There are also other meetings—which we used the opportunity to read out the banner, where the majority community meets for instance there is a community policing program which is a kind of festivity where cattle are also sacrificed on which every member of the community present themselves. In this program, there is an early phase in which we requested to have a schedule and presented ours. That is their own program and unless it is with the WDAs we don't have our own. Different from this is with the leaders but this is because the leaders meet more frequently. Regarding the general community, however, it is by utilizing the opportunity when others gather them for their own purpose, otherwise, we don't have the capacity nor the opportunity to gather or call the community for a meeting on our own.

a. How is the engagement of communities/WDA networks in the implementation of integrated community-based case management (iCCM) of common childhood illnesses/possible serious bacterial infection (PSBI)?

Respondent: the WDAs are our right hands. But most are now holding back because of the budget and high living costs (inflation), and because of that they move around with us as before since they are now being engaged in the daily labor. Unless for this challenges, they are our right hands.

Interviewer: are there any other reasons for their fewer engagement?

Respondent: Of course now they are inclined to their personal activities. previously, after you engage them for 2 or 3 days, there was some payment that you make to them, but now they spend the whole day, maybe for 2 or 3 days and she goes, but this woman works today for a meal that she needs tomorrow, it is a must. They are also having challenges in their marriage to hold back, being questioned about why they are engaged. Unless for these challenges, the WDAs are still in the right hands.

Interviewer: What is the impact this challenge has on your activities? that is because now they are not fully engaged, what is the impact on you?

Respondent: it has brought many challenge to us. for instance the respect you have when you are going in to the community with her and without her is quite different, they also don't give you attention if she is not with you; he might just leave you there standing and go to his business when you want to talk to him if the WDA is not present.

Interviewer: what is the impact in the iCCM service provision?

Respondent: regarding neonatal care, they might come looking for you if their child is sick to seek the treatment. They might even call us as I told you if their newborn is sick, let alone when we are available.

Interviewer: Ok so what I was asking about the WDAs contribution is in terms of their role to provide the iCCM service in the community?

Respondent: well previously the WDAs used to gather her 1-5 network and they used to meet at least during their coffee hours. But let alone making the 1-5 meeting, she herself is not being available the meeting. Previously when they also see her with us, they used to call her to tell her that their infants are sick but now when mothers see she completely cut her ties with us and that she is no longer moving with us, they tend to believe she is no more influential and that she left the role or no longer in the activity, then they directly go to the health centers or directly call us or come to us by themselves. But if they have consulted her, she would not refuse them. When we go to there, we are still using her even if it is forcibly with a police.

Interviewer: Now that they WDAs are not engaged in the activities, so how are you copping up and what ways you are using to overcome this?

Respondent: one of these is, when there are some invitations to send one or two WDAs by some bodies like woman's affair or agriculture sector, we select the WDAs who has been challenged the most to engage and kept away from us, so as to attract her again to come to us. there might be some WDAs who have been highly engaged but left us through time, so there is a condition where we send this WDA in order not to lose her. In addition to this, we also try to tell the WDAs that she is doing this for her own sake and that it will be her who they call for help when the child is sick. And we also tell them, that it will still be them who consoles the mother if the child dies too, also advising what to do for a newborn. Then they comply and try to engage, so there are also some who do these.

Interviewer: has the efforts in the SBCC and awareness creation made changes? What changes have you observed from the activities you implemented in the awareness creation on iCCM service and neonatal care?

Respondent: there are changes, for instance, there were no people who calls and informs about redness in the navel area of their infants, but they call and consult on whether they have to come or if we come to them. Even if it is limited but there is some improvement from the previous time.

Interviewer: How was it before?

Respondent: previously, there used to be infants we used to lose during our second PNC visit after visiting them first time but thanks to God, it has been a while since a newborn died on me since being reassigned to this kebele. I encountered two deaths in my previous kebele which I worked for two years, but now it has been two years since I started working here but I have not encountered any death. But there were infants we used to lose in the previous times. Even in other kebeles, you might hear an infant die after mothers called the HEWs and reaching to HF, but not just mere death, so there is an improvement.

Interviewer: so you have told me the changes, but was there any support you got regarding the SBCC activity implementation?

Respondent: the HC which supported us in observing the registration correctness and the implementation, and also the JSI-L10K. Especially the HC went down even to the village (Gote) level, especially Helen [iCCM focal from the HC] have worked with me for 3 to4 days and she has informed them that there is nothing special they get if they come to her, and that if it is beyond my capacity I will refer them to her. So she made the mobilization and supported us. TAKE IT UNDER 2 SECTION

Interviewer: so this is a general support right. But what was the specific support you got regarding the SBCC activities?

Respondent: even if we give the HC the money, it is the HC that prepared the banner using the list and displayed.

Probe: What are some of the **biggest challenges** with SBCC activities for newborn care?

Respondent: we disnt face any challenge when we were implementing the awareness creation activity. We also think we have done our best and there is nothing left to do regarding the awareness creation.

71. Could How do you describe the strength of the support you got from the PHC and the project as well as your efforts to address barriers to delivering iCCM/PSBI during COVID-19?

Respondent: well we had a strong support especially from Gash *Kebede [name changed, referring to one of the JSI-L1OK project staff].* I don't know why he has now been away since the month of TAHSAS, but he used to be here every day and checks on every you treat. We are nurses but his intention was to make us physicians [with laughter and admiration], he is also aged and does not lack anything to make the home-to-home visit and to support the mobilization. Regarding the HC's support, I can't

select out since they are part of us in the implementation, and there is nothing I can point out that they have not done.

Interviewer: what else? What other supports were you getting and the strength?

Respondent: regarding the HC, as I told you there is no supporter who does not provide awareness about the iCCM/CBNC service to mothers. In addition to this, the iCCM/CBMC focal also has been supporting us, especially staying for 3 to 5 days with us going in to each household and including on vaccination. I also call her for consultation and provides me guidance on how to treat the neonates, or she might tell me to bring them. So I cannot this or that about them since they are part of us.

Interviewer: How do they make the support, what is the approach in which they make the support?

Respondent: they come in person with the iCCM/CBNC chart booklet, and check those treated, that is from the HC. They also call us in every month, all the HEWs from every kebele, which we come with our registers and evaluate every gap we have, including the responsible one for the gaps etc. this is on monthly basis and we bring the iCCM register, and evaluate our performance.

Interviewer: How about the onsite support?

Respondent: it is a similar evaluation but it is made separately with each HEW. But here they share our experiences and during the onsite support they identify the gaps, and inform us of the reasons why we have not achieved it. Had it not been now for the different campaigns, we used to record all newborns in PNC but now we are limited to just recording those sick ones only. So they have been supporting us to this extent.

Interviewer: You mentioned about COVID-19. How has it been any challenge for you to implement the iCCM services?

Respondent: there is nothing that comes in the way of the iCCM service. But it was a challenge in the previous times when it was most feared, and it was at that time there the WDAs had diffused and remain that way still, that is around 2020 when movement and everything were restricted, and could not fix it still.

Interviewer: Why was it diffused?

Respondent: it was for the COVID reason that a meeting was banned. We used to have our religious gathering [TSEWA] every month which was banned at the time and it remained diffused after we stopped gathering. Even if we try to restore it now, we couldn't. now we try to meet the WDAs in their houses and sometimes we gather them, but we could not pursue with our monthly gathering, it was strong had a contribution and punishment for not complying.

Interviewer: Why has it become difficult to re-establish that?

Respondent: I don't know; I guess it may have to do with the inflations I think. As I told you before, inflation has got worse after that time forward. When we ask them, they also tell us about the high living cost and

reply to us about how they are supposed to raise their children if they are staying with us, then you don't have an answer but return back when they tell you this.

Interviewer: is there a form of joint support done in an integrated way?

Respondent: Yes, once in every quarter they come here and then they give us feedback.

Interviewer: is there a form of support based at the HC targeting improvement in your skill etc?

Respondent: at the HC, we bring that register, and then after evaluating ourselves and looking at our gaps, we finally get training and return back. JSI has given us an orientation when we were starting but there was no such support we got from the Woreda or at the woreda level. But JSI, I think has provided an orientation twice.

Interviewer: What kind of orientation or training was it about?

Respondent: at the beginning after they conducted an assessment, they provided us training at Markos. They conducted an assessment on all children and then they provided us orientation about the gaps, on how we should be working, I think for 2 or 3 days, and then we commenced the activity after returning back. They also trained professionals with gaps and new ones by JSI.

Interviewer: What else? For instance you mentioned the HC provided you training, what is about?

Respondent: it is about the iCCM CBNC in which we all bring our register and chart booklet, and then our performance such as case identification, treatment will be evaluated, and finally a case will be provided to us on how to provide treatment such as malaria or pneumonia and then, they will fill the gap we create while providing treatment for the case and they send us back.

Probe: What was changed?

Interviewer: how did it help you, and what things changed because of the support?

Respondent: it made us build our self confidence. We were very afraid to provide drug for an infant less than 2 moths, I used to be scared, and say what if he dies after I provide him the drug. Now I can treat a child below 2 months with full confidence, and I am very confident that now the infant I treated will be cured. I think it is gentamycin that we are very much eager to administer but we have not encountered that as of now but I have taken the training on the administration. In addition, we take out also satisfaction from the treated child and gain the respect of the community. iCCM/CBNC has gained us a lot of acceptance from the community, so it enabled acquire this. we were able to build our confidence to provide the treatment because of the HC support not just from the training, if it was just the training I don't think a single one of us would have been capable of providing the treatment. As soon as we received the training, the HC comes as soon as possible and push us to implement it telling us we forget it unless we do it now.

Interviewer: what about the support in terms of supply?

Respondent: there is Genta, Amoxa, and also ORS and Zinc—these are all fulfilled by the HC. Well regarding the iCCM service delivery, a stock out of drugs not even for a single is allowed by the HC, and if so you will be accountable.

Interviewer: What about in terms of fulfilling the COVID-19 prevention measures to implement the iCCM service?

Respondent: they used to give us sanitizers and face masks.

Probe: Was the support you got from the project and PHC helpful?

Probe: What could have been done differently?

Respondent: I don't have anything to say about that. Everything was supported.

Interviewer: it could be anything that you suggest or say if this was done this way it would have been better etc? it could be for the project or the HC

Respondent: No I don't have anything to say.

72. Do you think the support system is helpful (mentorship and on-site coaching methods, supervision, technical support, PHCU level PRCMM, etc.) to enhance your skills [that is HEWs' skills] over the traditional off-site training and woreda level review meetings?

Respondent: As I told you earlier, not only skill, it has capacitated us to provide the treatment with full confidence.

Interviewer: when you compare it with the other type of training that you have taken offsite, somewhere, and the support provided here by the PHCU and the project how do you see the importance in terms of building your skill?

Respondent: I have previously been on iCCM before, but it was not the HC that was supporting me, there was a person called Mr. Abebe [name changed, staff from JSI], that was supporting me at the time. It was from Alma-L10K, and that person used to support me every quarter. But the activity now and then was not the same, now the HP is working as one entity of the health center but previously it was occasionally when the cases come that was implemented. So the addition of the HC in the support is a good thing, it makes you feel as part of the HC and now it is much better. It is that difficult to compare the difference when only the project was supporting us and after the HC is integrated.

Interviewer: what makes the addition of this HC being incorporated in the support different now?

Respondent: you make a common task now. Previously it was only the responsibility of the HEWs only but now it is a shared task and responsibility between the HC and HP. Hence, if it fails we both are responsible, as a result we are supporting each other. The collaboration is also more helpful for us since the knowledge we have is limited.

Interviewer: How aboutim terms of skill that you have gained, anything that you say have built and improved from the support? What have you got?

Respondent: the skill I acquired from the training was not uniform but now in terms of speed and also with regard to ordering the medication from the chart booklet I have gained so much skill. But previously because of fear I used to make errors, now I may have some clerical errors I might make on the registers but now there is no activity that I don't do because of fear, I do all the activities with confidence. Previously there was nothing we know about medication, we don't even give paracetamol, but as a result of this support, we are now even providing paracetamol for adults.

Interviewer: Are you telling the iCCM support has contributed to this?

Respondent: yes, it was also brought in at the time we started providing the neonatal care, we were told as well to provide for adults whose body temperature is above normal.

Probe: for acceptability of the strategies **(for HEWs only)**. How could the support system be improved?

Respondent: previously we didn't like it when they support us because of our fear and rushing of things when they come. We also tend to reject them, and say is it going to be like this after every time we take a training. Our assumption was just to get the training there and come back, we were not told that we were going to treat. And we were complaining how we could be able to provide treatment with this knowledge we had, and hence we tend to resist them. But now it is not like that, and we really enjoy it when they are supporting us. if we have any gaps we either call them or they come and support us.

Interviewer: Do you think this has improved your linkage with the HC and your working relationship?

Respondent: Of course yes, previously it was only in two or three weeks that we meet but now we are like staff working in the same place. When you see everyone they all seem like working here, we are one.

Interviewer: How did it strengthen your linkage?

Respondent: it is because we support each other since we have a shared responsibility. And when the support from the HC, one health professional is assigned to support one Kebele.

Interviewer: what do you suggest should be improved?

Respondent: well it is only the location of our HP, which is found in a dangerous area. It is even dangerous for us to work there. There is not a single HEW who works alone in the HP because no one will come for you if you even scream for help, hence, the house-to-house outweighs the activity we did at the HP. I have been startled twice working at the HP when approached by a demented person; the HP is located near a holy water site where such people come for healing. This person came straight to the inside of the HP but managed to escape, so I say if the location is reconsidered. I would not say it should be relocated up to the periphery but down there where majority community lives.

73. How eCHIS implementation helps you with iCCM service delivery?

Probe for advantages:

- case identification? quality of iCCM case management? retrieval of client records? data quality? client appointment scheduling and/or defaulter tracing?

Respondent: so far the eCHIS has not helped us with anything in the iCCM service delivery.

Interviewer: What does the eCHIS incorporate as it is and that has some helped you in the iCCM service delivery? What does it have for instance?

Respondent: I don't know if it is me who didn't see it but there is nothing I have seen so far for the iCCM service delivery. But it is good to record the other services even of the device is not good; the tablet is not something you move around at ease. The tablet is so dull; it makes you wait a long time after you touch it to move to the next action, at least after 10-15 seconds which is difficult to swiftly fill in data. Either you have to record with a hard copy, go home and take time until late hours, otherwise it will not record it for you.

Interviewer: for what services are you currently using it?

Respondent: to organize the WDAs, health insurance, PNC, delivery, etc. Well, the delivery makes us make a follow-up for the newborn through PNC but usually, the delivery comes through an action card or it flags red. But I have never used it for iCCM.

Interviewer: could it be used for iCCM purposes?

Respondent: yes, because the application is available. For instance, after the newborn is delivered, you can go inside the app and use it to provide the treatment.

Interviewer: So why didn't you use it?

Respondent: because we didn't take the training and I have not also seen it. but it has delivery, PNC, vaccination services that I focus. For the iCCM, it provides me some indication because after the mother delivers it indicates to me the PNC and when I go for the PNC, the newborn might have some illness like redness in the navel, pneumonia, skin lesions, etc. so it makes me check that, even if I don't treat him using the tablet, I will otherwise treat him going in there physically when I make the visit for PNC but I don't feed that in the tablet for the iCCM, but it is possible to do since we have been using the tablet for EPI and others.

Interviewer: Is it possible to identify cases using the eCHIS?

Respondent: Yes, there is a section for it that asks about weight, presence of local bacterial infection, and redness in the navel. I will treat him accordingly but I never use the tablet for the diagnosis or the treatment, but it is there and asks you about it. As soon as you finalize feeding in about the mother, it immediately asks you about the newborn; does the newborn has a name, his weight, does he have a local infection, is there redness or pus in the navel, and if you answer NO to it, then it will congratulate and allows you pass to the next. But if you say yes there is redness, it will guide you to refer to HC but it does not guide you to provide any sort of treatment. But it is available in the manual iCCM within the chart booklet so you treat him accordingly.

Interviewer: what is the benefit of the eCHIS in maintaining the quality, and how is it helping you?

Respondent: well it corrects you, for instance, if there is anything you skip without filling like his age, then it will stop you and flags red.

Interviewer: how about with regard to case management, does it help with that?

Respondent: if you don't make the right diagnosis, it will inform you something is missing and but if you make the right diagnosis it will say congratulation and give you an appointment. For instance, if I feed in a lower weight, then it says it is malnourished and recommends to refer him.

- **Ask why?**
- **Probe for areas of strengthening?**

Respondent: I have not taken the training and hence I don't have anything to suggest on the areas of strengthening the eCHIS.

74. How was the introduction of the iCCM/CBNC module into eCHIS affected the iCCM/CBNC service delivery?
    FOR LUME WOREDA ONLY extra

XIV. Implementation challenges

75. Describe the main issues faced by the health system to identify and treat neonatal infections in the community. What are the critical factors affecting the delivery of iCCM to clients?
    Probe:
    - lack of competence of HEWs, shortage of supplies and commodities; weak support system; low community demand?

Respondent: what I see as a challenge is the lack of motivsation of the HEWs by the government. It is not like the other professionals, and if there is anyone who had to live deprived of anything is the HEW. From all the professionals working at the kebele, be it with promotion or for anything, if there is anyone who had to work deprived of anything is the HEW, and as a result of that, most of them are demoralized. We also don't have a good reception from everyone starting from the WoHO this is because we don't satisfy them that much because of load of tasks we have. Recently we have been in the trachoma campaign and seeing the bad face of reception from the farmers that is like fire, either they don't have a proper education but just with the little knowledge they have, it is like they put on us on fire. We are pushed aside from the community, and the higher government bodies are also pushing us back, in the meantime, they are making us hold on to the live fire.

Interviewer: Why is the community pushing you away?

Respondent: they are accepting anything that is being done, be it the fertilizer issue, the war and everything that is going in the region, they are not receptive of us because of that. Especially when we are engaged in the COVID-19 vaccination, they tell us there is nothing we can do since we are sent from the higher bodies, they question us if we have verified the vaccines. I have never thought I would be asked like that from a farmer, but he told me that Tedros Adhanom is the Tigrayans' leader so what he is

sending to sterile the Amhara region so I won't take it. I didn't answer him anything since I never thought about it. they also tell their problem is the lack of fertilizers suggest it would have been if they change the budget for this to the purchase of fertilizers.

Interviewer: But are these factors have also been a hindrance to provide the iCCM service to the community?

Respondent: since the iCCm service stands for itself there is no challenge, and it was even this service that gave us the opportunity to implement other activities like COVID, Trachoma. We use it to convince them; we say to them like you are taking you little ones for treatment, you also get treated, where are you getting these drugs, is it not America that is sending these drugs too and it is when we tell them this that they tend to be convinced.

Interviewer: any other challenge on the iCCM service delivery, supply issue, community perception, the support system etc?

Respondent: No we don't have a supply shortage, and the community is also very positive towards the iCCM service delivery. They look for you and come to get the services.

Interviewer: How about the conflict in the northern part of the region?

Respondent: no it didn't have any influence on the service. We rather have been especially following pregnant mothers whose husbands went to the war front by going in to their houses. The impact is on us, not on the service delivery.

Probe:

- Regional/national state of emergency and conflict in the northern part of the country?

Interviewer: what about the state of emergency?

Respondent: the restriction of movement has impacted us not to work as we wish to, as in the previous time. For instance, in the previous time if a mother is pregnant or a newborn is sick we might ask the mother to come along with us and return back after getting the newborn treated. Previously if a mother wants to take a family planning but the husband refuses, you either convince him or you take the mother forcibly, but we could not do that at the time, fearing if the husband hit us with his stick. There was police enforcement or order at the time.

Interviewer: was there impact related to the neonatal care provision (iCCM service)?

Respondent: There was nothing regarding the neonatal care.

Probe: How has that changed during COVID?

Interviewer: How has changed with the COVID? I mean the problems you mentioned, how did they change because of the occurrence of COVID, did they get worse or no impact?

Respondent: there was nothing, there was no change.

76. How do you think the COVID-19 pandemic and/or COVID-19 response measures affect the uptake and/or delivery of iCCM services?

    Probe:

- Has COVID-19 affected your daily routines; your work on newborns; the community in terms of livelihood and vulnerability for newborn care-seeking
- How has that changed over time?

Respondent: I don't think COVID had any impact in the iCCM service delivery, but I think it was at the HCs there was an impact since there were limited health workers at the HC. They used to complain they could not get any one at the HC. Out of this, since we were going to each household together with police, agriculture and other sectors, it was conducive to provide the iCCM service. It was at the HC the gap was seen since there was limited professions, mothers were even delivering in their homes and they were telling us they could not find any and the ambulance also could not come for them, so they delivered in their home. The iCCM service at kebele level however had no problem due to COVID, it was actually an opportunity and many newborns were treated because we were going to each household.

Interviewer: were the community willing to interact with you?

Respondent: it was just two months that COVID was attention in the community. But after that the community believed it didn't exist, and it was a fabrication of the government to extend the election time.

XV. Adoption and reach/effectiveness

77. Explain to us any changes you observed as the result of the implementation of the COVID-19 adaptive iCCM implementation strategies? – What impact have the strategies had?
    - What are the particular features of the strategies that made a difference?

    Probe:
    - for a support system and linkages, motivation, and competence of HEWs, community, and awareness creation?
    - What do you think are the reasons for non-significant changes?

Respondent: the change is that newborns now don't go to the HCs for no reasons; they either come to the HP or they might call and tell you to come to their homes. For instance, I treated two newborn cases just from a phone call; one local bacterial infection and the other redness in the navel area. I am not saying I don't treat others but this is just from a phone call.

Interviewer: what are the changes from your side, that is owed to the support?

Respondent: at some point there was a time we thought the activity was the project's only and hold back a bit but later the project recommenced and started the implementation as a new; previously we used to only record the mothers who deliver in the PNC register but we don't record the newborns in the iCCM register. But now we register all the newborns in the iCCM/CBNC register.

XVI. Maintenance and sustainability

78. What are the high-level benefits that are attributable to this support/IR?

Respondent: it was the support from the HC that ranks first, then the JSI's support.

Interviewer: what I mean is from the support, those benefits you regard highly of?

Respondent: for me all type of supports is equal but the support at the HC is a form of training using cases, but the support here at the HP is just the observation of the chart booklet, the register etc but at the HC, you will be assigned a specific case from each type, be trained, and even if it is not on the actual case, it is after you provide the treatment that you return back.

Interviewer: what makes the support at the HC special?

Respondent: what makes it special is the task of the HC and us is shared one; we are both responsible, and if for instance if I fail it will be the HC that is first held accountable, then I will be next.

79. Please explain to us the feasibility of this support/ IR for national scale-up?

Interviewer: Ok now what are some of the supports that are feasible and can easily be scaled up at the national level? Among the supports that you have been receiving, which are most likely to be scaled up?

Respondent: the iCCM service could easily be sustained by us only even without the involvement of the HC.

Interviewer: what do you think is the reason?

Respondent: it is because the HC made us competent. The HC will go about everything strongly that may be others addressed it lightly. So the support from the HC is good.

Interviewer: do you think the support you have been getting will continue?

Respondent: yes, it can sustain. We are still being supported even now, and they have not stopped because the project ceased, they are still doing it.

Probe:

- What features could easily be integrated into the existing system? Which not?

Interviewer: what are the high level benefit that you got with regard to sustaining the activity?

Respondent: we have exerted a lot of effort in terms of sustaining the activity, but since there is a high load of activity it has been difficult to sustain. There are a lot of field level activities like campaign, and if you are engaged in the field activity you will not be effective since it makes you hold on to one and drop the other activities. But despite all the load, we would not just pass by if we encounter a sick child, that is something we sustained about.

Interviewer: what is it that it gave you to do that? Did it improve your commitment, motivation?

Respondent: it created awareness, motivation and compassion with regard to iCCM service. Now I will not just ignore if I see a sick newborn, I might skip just others but not in case of a sick child. It is a big deal, when you see a newborn you treated get better.

Interviewer: what is that you think you can easily sustain from now one without major support.

Respondent: I think it will be it [the iCCM service]. I don't think I will stop giving the house to house care for the newborns despite all the challenge in the field activity and others.

Interviewer: what do you think the type of supports that may not be integrated or sustained in the existing system?

Respondent: may be what I see struggling is the activity regarding PNC that requires to register all those delivered may not continue from the workload. It requires that all those delivered in the kebele should be registered, for instance, if there are five births in the kebele, you have to go there, register them and return back.

Interviewer: why is difficult to do?

Respondent: it depends on where you stay on the day and the repetitive task you are being assigned. It is only due to the work load.

80. How are the activities/efforts embedded in the PHC and woreda routines/work streams?
    - What implementation strategies are incorporated with the PHC and woreda annual work plan?

Respondent: we are integrating the iCCM in every activity we are doing; we are integrating it in the house to house visit, in the HP stationed service. In addition to that, it is also included in the reporting, first it says live births in the kebele and then, there is a report for local bacteria, pneumonia, and diarrhea which are all available in the report. It is not just the neonatal care; it categorizes the diseases. The HC is also receiving the reports the same way we collect them. There are not places where the iCCM is not integrated.

Interviewer: do you also observe the integration of the iCCM when they come here at different times for support?

Respondent: yes, we do. When they come for supervision they observe the iCCM service and cross check the report with the tally and register. We have also plan for pneumonia, sepsis, local bacteria, diarrhea, which is categorized by quarter, and in number.

81. Do you have anything you think is important to tell us that we have not asked you?

Respondent: I don't have much to say but just if the government recognizes that the HEWs are the most disadvantage and sought some solution for us.

Interviewer: Thank you, we have finalized our question.

THE END

End line Evaluation of the PSBI implementation research; Full Transcription for an In-Depth Interview with a Health Center Head

Questionnaire ID	04
Area Identification	Dembecha
Name of Woreda/Zone/Region	Dembecha WoHO/West Gojjam Zone/Amhara Region
Name of facility	Health Center
Name of moderator	
Name of a note taker	
Date of Interview	20/06/2022
Participant #	01
Audio File #	HP_HEW_20.6.2022
Start time:	09:00 AM
End time:	10:10 AM
Transcriber/Translator	
Duration of IDI:	1hr, 10 minutes

XVII. Fidelity

82. Can you please describe the demand generation/SBCC activities you are doing on iCCM/ newborn health? What are the strategies used to raise awareness? If none, please explain why.

Respondent: Ok thank you, I am Yezeleka health center head. Regarding the newborn health service, we have got different support and training from JSI L10K. Based on this, it was possible to implement different awareness creation activities for the community to provide the service at the HP level. Among these activities, the first is listing the services provided at the HP including the newborn health service through a banner that was developed from the contribution of the community as well. In addition to this, it is also important to maintain the standard and cleanness of the HP while providing the newborn health care service, we have painted the walls, availed chairs and such activities were possible to do, and hence we have also seen changes as a result of this. We have seen better newborn health care service provision in the last year, and we have also seen that this performance was very satisfactory which we achieved more than 100%; it was a year where sepsis medication and others were provided. As a challenge maybe we have a supply shortage.

Interviewer: Ok but before the challenges, were there any other type of activities other than the banner for the SBCC activities?

Respondent: other than the banner, we have provided awareness at churches about the provision of newborn health services at the HPs; the HEWs have provided education for the faithful at churches that the newborn health care service provision is available with them, and if they cannot manage it that there is also a referral to the HC. Frankly speaking, this has resulted in a lot of changes.

Interviewer: By whom was it provided?

Respondent: by the HEWs, and our professionals who are supported from our HC. Frankly speaking, it has brought a gradual but good improvement, and service was provided in a greater degree.

Interviewer: was there an awareness creation provided only by the HEWs or other bodies?

Respondent: it was provided more by the HEWs at churches, and later, it was also introduced to the WDAs, which they have contributed even if it is not that satisfactory; it is not like before that they are implementing at this time, and there was some activity implemented on them. These are the ones who provide the awareness, at churches with the religious leaders that is in general terms and not to go into the details.

Interviewer: Please tell in detail?

Respondent: Ok as I told the HEWs take the lion's share in the awareness creation, and supporting bodies and kebele leaders have also played a bigger role. The reason is that they have discussed with the HEWs coming down to the HPs about the implementation of the expected activities; the kebele leaders are also more influential than the HEWs and accepted by the community. Hence it was possible to create awareness in the community about the provision of newborn health care services at the HPs by communicating with the Kebele leaders. So that is it and how it was implemented.

a. How is the engagement of communities/WDA networks in the implementation of integrated community-based case management (iCCM) of common childhood illnesses/possible serious bacterial infection (PSBI)?

Interviewer: How about the engagement of the community and the WDAs network in implementing the iCCM service?

Respondent: Ok, as I said earlier the engagement of the WDAs is not like before. I think there is some boredom from working for a long time and there have been some benefit issues in the former time. I think it is because of these reasons that now they are providing any support and keeping away from the services. I personally tried to discuss these with them at Asteboch HP, but it was not fruitful and mentioned that they are considered as irrelevant, and the final conclusion also had to do with the incentives they used to get in the previous times. However, they had also some help in countering of some myths and misconceptions arising from the community. Other than this, the WDAs in terms of providing support has a very limited role.

Interviewer: why do you think that they are having a limited role?

Respondent: one thing is from boredom developed from working a long time and the other I think is related with the benefits issue they have been getting before; in the earlier times, some things line Umbrella and others used to be provided to them, but it is not being made now.

Interviewer: why is not being made now, why did it stop?

Respondent: I think it is a budget deficit. They used to get these things from some NGOs and I don't think they were getting these from the government side. But I think there is something initiated at the woreda level to strengthen the WDAs.

Interviewer: what was the role of those limited WDAs in supporting the iCCM service delivery?

Respondent: it is with respect to providing awareness to the community. Since the WDAs had some newborns living in their neighbors it is in terms of providing awareness to their neighbors. But it is not that significant, they might be 10% of them doing that to the maximum. Frankly speaking, it is mostly the HEWs doing most of the activities at churches and other places, and also our supporters—those supporters from the HC, and for one Kebele there is one supporter assigned. Other than these, the WDAs' role is insignificant.

Interviewer: Are there some ways that you have implemented to fill the void left due to the WDAs lack of engagement?

Respondent: In order to solve the challenge, it was not only about the iCCM service that the WDAs was intended for, it also helps in the other activities. And hence, in order to solve these gaps, as I told you I tried to select two HPs and work on them but it didn't turn out to be successful, and I didn't find it something to be solved by the HC alone, and hence I also held a closed discussion with the kebele leaders for 2 or 3 days but it was still challenging. So when it becomes this challenging, we also started to work with those willing until something from the WoHO is communicated about it. But this was the extent to which we went but it didn't turn out successful.

Interviewer: What was the participation of the community in the iCCM service delivery?

Respondent: the community participation is improving from time to time, especially after JSI supported the iCCM service delivery, it has become good. That is in terms of renovating the HPs, owning them as their own, and bringing their newborns to the HPs to get health care service; as I told they just don't come directly here, they first visit the HP.

Interviewer: what was it that you did to have this improvement?

Respondent: it is the promotion work that we did, and also the filling of the skill gap the HEWs used to have. The other is also because since now the HEWs acquired adequate skill they can provide medication service wherever they go based on the knowledge and instruction they had. And this is the reason why we had an increased newborn number who are treated.

Interviewer: is there any other form of organization that you have utilized other than the WDAs to improve the iCCM service delivery?

Respondent: what we call as organization or maybe it is not actually an organization but there is what we use to execute other activities, for instance, there might be an outreach for a campaign like for Polio, Cholera, Azithromycin, and such things, which we also integrated other activities including iCCM service. Other organizations in the kebele such as the Men development armies have helped a lot better than the WDAs; the MDAs are much better than the WDAs in making promotions. They have helped a lot in promoting things, linking, and sending the community to the HPs.

Interviewer: Have you implemented something different to engage the MDAs?

Respondent: To tell you the truth, I usually organize and chair meetings for the surrounding kebele, bigger meetings with the so-called big core leaders consisting of the Kebele leaders and organization (party) leaders, which we hold a discussion with them, and we make them communicate this to the WDAs. This is the system I have been using so far. And also to use any opportunities they got to communicate about the availability of newborn health care services at the HPs. This is the organization we have been using.

Interviewer: what else, is there anything called a village organization, or something like it?

Respondent: Yes, there is. Kebele is organized into villages, and in one village there is one leader, and this leader it the representative for this village (Gote). WDAs are also available in each village, and it was these WDAs who used to work for us.

Interviewer: Are the Gote (village) leaders engaged now, do you utilize them now?

Respondent: yes, they are working.

Interviewer: How are you using them and what makes them different from the WDAs?

Respondent: I use them in any kind of situation. And what I consider the closest and works better than the WDAs are the village health leaders, and if you ask me how, for instance, if there are different campaigns it will be them who we directly communicate with, and if there are people we want in the community regarding iCCM service it will be them that we provide information. One thing is that they have cell phones nearby and also for meetings and other issues, they are better than the WDAs.

Interviewer: Any preparatory work you did or support you got to engage them in the activities?

Respondent: we didn't get any support, and it is with the core leaders that we communicate. As I said it, it is not only iCCM but our biggest challenge was with the implementation of the package, and in order to implement this, we work with the core leaders, and the core leaders also know the village health leaders and this was how we were linked. We also go to the village level (Gote), for instance, there is a village (Gote) called Genbo, and if there are activities at the village, I myself call and inform the VHL that there are HEWs and supports who will be coming and to work with them. When we also want to go to the other village (Gote), it is the WDAs that take us there and then link with the village health leaders to work with us. This is how we are using them (VHLs) for the activities.

Probe: What are some of the biggest challenges with SBCC activities for newborn care?

Respondent: the doubt in trusting the HEWs to provide the newborn care service has been resolved from the awareness creation activity. But our challenge is the shortage of the HEWs, and as a result of the shortage HPs might stay closed for a day, and when the communities come for the services they find it closed and complain that we call them to come but it is closed.

83. How do you describe the strength of the support you got from the PHC and the project as well as your efforts to address barriers to delivering iCCM/PSBI during COVID-19?
    Probe: What was changed?

Respondent: regarding the support as for our HC, even if we don't go out to make a specific support, but we do that when we making an integrated supportive supervision. But what surprises me the most is the support from Gash Abebe [JSI assigned project staff], just like his own in making regular follow up and guidance to implement the activities, and his support was immense. However, as for our HC there was also support, but it was not just specific for iCCM that we were supporting, it was integrated when we go out including for the other services; it has its own focal person, and it trained them for us and when the HEWs were having any difficult he supports them. That is what the support looks like. In addition to this, we also used to support remotely through phone communication.

Interviewer: How often do you make the support, and also the type of support provided? Your support as well as the project's?

Respondent: Our support was on skill capacitation, it was called as PCRMM where all the HEWs come here, and provided a practical session training using the actual health care service provision in the under 5 OPD, observing while the professional provides care, and also observing their register practically, and we also observe the HEWs being engaged, so this is how we have been providing training for the HEWs. We either call them here or show them on-spot at their HPs to show them the proper implementation.

Interviewer: How different do you think is this approach you called PCRMM from the other conventional support systems?

Respondent: PCRMM means, a practical observation made by the HEWs, while a newborn is being cared for, or they might also practice by providing care for the newborn, and which in the meantime, they take lessons and get an education.

Interviewer: How often do you provide them this, and what is the consideration for it?

Respondent: so far it has been conducted three times, and the purpose is to assess their knowledge and used to evaluate how good they are providing newborn care at the HPs. The other support is made onsite by looking at the register and providing guidance on the proper implementation.

Interviewer: Do you use a checklist for this? and how do you provide them feedback?

Respondent: Yes, we use a checklist. But we don't give them formal written feedback unless we make it through a phone call, and orally.

Interviewer: How about the project support?

Respondent: the project is just like I told you, daily he receives a report and gives us feedback. He also supports them going in person to the HPs, and gives us back feedback about their strengths and weakness, and about the area of support they need. He also provides them with onsite training.

Interviewer: How was your coordination with the project?

Respondent: we work in coordination with the project, they inform us ahead they are coming and give us different directions and we also try to implement to our best the tasks they give us.

Probe: Was the support you got from the project and PHC helpful? What could have been done differently?

Interviewer: After you started working on the project and implementing activities, what changes have been achieved?

Respondent: the changes as you can see is, I can say the neonatal death has come to zero, and also the rate of flow of cases for newborn care has also decreased since they were being treated at the HPs. The community was also not coming to the health facilities because of doubt if they could get a cure from the health facilities, now this awareness has improved and they are now coming to the HFs and being treated. So we managed to minimize neonatal death, and suffering of the newborn and also lowered the HC burden after the commencement of the project implementation and the provision of awareness creation in the community.

Interviewer: Do you think there was a chance the support could have been provided differently or in a different way?

Respondent: what I think would have been better is in terms of strengthening the WDAs, and if the project has been implemented also in terms of strengthening the WDAs a different result would have been achieved. The project has done a lot and benefited from the implementation but it would have been great to include the WDAs in the implementation, and this is just because women are more close to children, and if still the project works on WDAs we can achieve even better outcomes.

84. Do you think the support system is helpful (mentorship and on-site coaching methods, supervision, technical support, PHCU level PRCMM, etc.) to enhance your skills [that is HEWs' skills] over the traditional off-site training and woreda level review meetings?

Respondent: both the onsite support and off-site training are important, but the onsite training is best, but there might be professionals who complain about the lack of benefits in the onsite training. Since we also need to speak frankly. The other is the disadvantage we have; we could not go and address every site due to the shortage of professionals we have and because of this, it has become difficult for us to train all. However, onsite training has its own advantages because it is what you see practically. As

per our HC, we have also tried to discuss the disadvantage stating that it should not always focus on benefits and others.

Interviewer: which one are you recommending?

Respondent: it should not be much, if it is too much for instance if all are trained offsite and if you are not going to practice it and show it to others, it won't have value. But if you have 10 professionals, 4 or 5 of them will be trained and, I think it is better if the trained ones could capacitate the rest.

Interviewer: What is the skill that you think the HEWs have got from the onsite support?

Respondent: They have got a great deal of skill, you know they have never been providing care, they used to just refer clients to the HC. But now they can provide care, they know the specific doses to provide, and they have confidence now. So being able to save a life, gaining knowledge, and saving times are the benefits. It is because they got skills they are able to do this, and because they are able to give care at the HP they have decreased the patient flow coming to the HC, and are also able to save lives.

Probe: for acceptability of the strategies **(for HEWs only)**. How could the support system be improved?

Interviewer: How receptive were the HEWs to your support system, how do they accept the strategies?

Respondent: as per our cluster, since the HEWs have not worked for a long time here, they are ready to receive any type of support. You see them being eager to grasp knowledge.

Interviewer: Earlier you told me that it is only the integrated support system during supportive supervision, don't you have a separate type of support like mentorship?

Respondent: We have that kind of support even if it is not that strong. For instance, after our 3 or 4 professionals were trained on iCCM/CBNC they were made to support 3 or 4 HPs alone, since the Gash Abebe also tells us the gaps and areas of support, we have made that support. However, as I told you earlier it is about 31,975 populations that this HC is serving and the available professionals are very limited, as a result of this problem we were being challenged to make separate support. But as I told you, we have tried to support four or five HPs just after the professionals were trained based on the feedback we receive from Gash Abebe [JSI project staff]. This is what we did and since this year things were also difficult due to the campaign, it was difficult to make separate support.

Interviewer: You also told me earlier that the PCRMM was interrupted for some months now. What is the reason?

Respondent: it is because we thought their skill and knowledge gaps were solved, and so there is no condition to call and conduct PCRMM every time, and hence because of this it was interrupted for 3 to 4 months since they have observed everything and we thought they were competent.

Interviewer: Is there a way you think that the support could have been implemented differently in terms of improving their skill?

Respondent: I think the support so far is adequate, and there is nothing I suggest to do differently except strengthening the WDAs in training which I mentioned already.

85. How eCHIS implementation helps you with iCCM service delivery?

Probe for advantages:

- case identification? quality of iCCM case management? retrieval of client records? data quality? client appointment scheduling and/or defaulter tracing?
- **Ask why?**

Respondent: regarding the eCHIS contribution to support newborn health care delivery, I have not seen any tangible. It is yet to be done that, we are still recording all households and are not done yet.

Interviewer: Ok assume it is integrated into the eCHIS and what could be the contribution then?

Respondent: it will be very helpful, for instance, it will be helpful to remind those things likely to be missed like what to things to feed in for newborn seeking care, and since it has the chief complaint and gives you many things which are important to take proper care. So it is more important than the manual system. The second is, that it saves you the expense you spend on stationery and others for the manual, and also to have a valid age recording once it is archived for the newborn; a mother might come and tell you the newborn is 9 months when he is 1year old already. It is also quick.

Interviewer: Would it be possible to use it for case identification?

Respondent: it will help, because you do the physical examination since it does that for you on its own like if he is below that or this, and it also out the option if it has fast breathing, respiratory rate, etc, and you also follow and put them accordingly. So it helps to conclude whether the newborn is sick or not.

Interviewer: How about the case management?

Respondent: it does help the case management since it directs you what to give for things happening etc.

Interviewer: can it also ensure the data quality in the iCCM service delivery?

Respondent: Yes, it ensures, for instance, as I mentioned it earlier if there is wrong age told, it will be correct since it is already registered, and possible to determine the current age, it does improve the quality. It also corrects errors in sex recording, as well as improves the quality of the case management since it guides you to administer this and that.

Interviewer: How about easy retrieval of data?

Respondent: yes, it has a bigger advantage instead of collecting data manually, you can easily obtain from the monthly report including those treated or sick newborns, and since it is possible to generate this and send it to the HC, I think it is easy to implement.

Interviewer: could it help in the scheduling and defaulter tracing?

Respondent: it will also avoid those things. You appoint her to come on some specific day, and since it also has an action card which we can trace her and also phone number recording option. It has many advantages in terms of data quality, and quality in case management, so the integration I think will have importance.

- **Probe for areas of strengthening?**

Interviewer: What are the areas that the system should be strengthened, and how do you think this should be strengthened?

Respondent: I have not actually seen it thoroughly but the report still has not been generated and if the report is generated. The second is, if once registered I don't think it makes updates, for instance, if once age is recorded I think it is you who makes the update, I don't think it makes the updates. These are some of the things, there may be more if seen thoroughly.

86. How was the introduction of the iCCM/CBNC module into eCHIS affected the iCCM/CBNC service delivery?
    FOR LUME WOREDA ONLY extra

XVIII. Implementation challenges

87. Describe the main issues faced by the health system to identify and treat neonatal infections in the community. What are the critical factors affecting the delivery of iCCM to clients?
    Probe:
    - lack of competence of HEWs, shortage of supplies and commodities; weak support system; low community demand?

Respondent: one of the implementation challenges is a shortage of the HEWs, and coupled with this, there are distant places like Addis Zemen which are not possible to address by the HEWs alone, and hence one Midwifery or Nurse should be assigned with the HEWs. If this is done, all activities including newborn health care could be provided; you can imagine how people living in these areas could suffer to come here if the services are not available there. So let us reach them out where they are, and if the relevant professionals are also assigned.

Interviewer: the other professionals you proposed, what is it for? To provide which services?

Respondent: one, if they are above the HEWs qualification, to properly provide the iCCM/CBNC service at the HPs, and the second is to provide labor and delivery service; if there is no labor service, it is difficult for the iCCM/CBNC or to provide delivery service; if they run of options they deliver at home, or they might deliver at the HPs by the HEWs. So with respect to such things, I think if the two are fulfilled we can bring about a significant change. The second operation challenge I consider is a shortage of supplies and interruption, gentamycin was scarce for some time including Ampicillin, and so these two challenges have been one of the biggest hindrances to providing newborn health care services. So these are the biggest challenges, which are shortage of supplies, lack of assignment of relevant professionals in the right place, or the distance the HPs have from the HC.

Interviewer: What about the competency of the HEWs, can I be a challenge?

Respondent: there might be some gap but we can say we have fulfilled 90% of it.

Interviewer: How about the motivation and interest of the community to seek the service at HP?

Respondent: we can not say the communities interest is 100%, one of the reasons is the HPs being closed when the HEWs have field duties, the second is the supply interruption, and the other is the HPs status being below the standard; if you have seen at Kendabo Nebersa HP, it does not look like HP and it is physiologically difficult to get medication there; the physical space of the room is very narrow, it

does not have its own separate room, chairs, data are not orderly laced, it does not have proper painting and the floor properly made. When you are coming to get health care, it should also be ready and in good condition, and mothers will also have good intentions to come and get the services. Let me give you an example, if you have seen the Kendabo HP it does not have a shelf to store the CHIS files, and even if you wish to have one, the room does not have adequate space, it also does not have a window, so I also think the HPs being below standard also contributes for the challenge for the proper provision of the newborn health services.

Interviewer: What is the support you are getting from other bodies?

Respondent: Like from the Woreda, yes we have been getting some support from the Woreda Child health office but it was not that strong and they usually don't come regularly.

Probe:

- Regional/national state of emergency and conflict in the northern part of the country?
- How has that changed during COVID?

Interviewer: what about the conflict in the northern part of the region, how has it affected the newborn health care service provision?

Respondent: A I said it, the impact the conflict has brought is not that significant and we also didn't encounter any supply shortage due to the conflict, but maybe we have been sharing some of our drugs to nearby HFs. There was also an impact in the professionals since we send out some of our professionals to the war front, and due to a stability issue the service at Addis Zemen HP was closed which had a lot of burdens; the HPs were closed for a week or sending one professional from our side mean a lot of burden to us.

Interviewer: How about the impact from the community side because of the war?

Respondent: No there was not much influence on the services.

Interviewer: What was the influence of the state of emergency declared for different reasons such as COVID?

Respondent: that had some influence and the performance was also low. But as per our cluster, we didn't really stop providing service and instead told them to put on their face mask and use the service, and the influence we think might be about 5% since we have also evaluated it at the time. The Yezeleka catchment flow at the time was also different from the rest facilities, people were also asking us the reasons but it was just that we were telling them to put on and come to use the services by being present at churches, etc. Sanitizers were also availed at the gates, and they were using face masks.

88. How do you think the COVID-19 pandemic and/or COVID-19 response measures affect the uptake and/or delivery of iCCM services?

    Probe:

    - Has COVID-19 affected your daily routines; your work on newborns; the community in terms of livelihood and vulnerability for newborn care-seeking
    - How has that changed over time?

Respondent: one of the bad influences that COVID brought was some people were scared to come because it was thought, and circulated by some presumably educated people, is that health facilities are the places where COVID could be contracted, and some people who developed pneumonia also didn't come because of fear that it might be related to COVID. And so we think it may have brought some 5% influence as I mentioned earlier. But we rather think that the occurrence of COVID has helped us in preventing other diseases, and mobilizing, resources like sanitizers face masks, and prevented us from exposure to other diseases. We also exerted effort in terms of creating awareness for the community on the prevention measures but since the COVID impact was observed much in the area, they didn't trouble us to use the services.

Interviewer: Ok you are telling me it had no influence, were the HEWs not challenged to go at the household level and provide the care?

Respondent: as I told you, we were not challenged not the community, as I mentioned it already unless for the probable 5% influence it may have on the community, they were not troubled, and it didn't make any influence on us.

XIX. Adoption and reach/effectiveness

89. Explain to us any changes you observed as the result of the implementation of the COVID-19 adaptive iCCM implementation strategies? – What impact have the strategies had?
    - What are the particular features of the strategies that made a difference?

    Probe:
    - for a support system and linkages, motivation, and competence of HEWs, community**,** and awareness creation?
    - What do you think are the reasons for non-significant changes?

Respondent: the support strategy which I think was good is our PRCMM, and it has brought a big change since they were able to observe the activities practically and they were implementing it. After we provided the PRCMM, we were also seeing a big change in their registration and hence we consider this as something significant. It is not just the registration quality, but also the administration, management, and categorization, and on such things, they had a good improvement. As a result, the flow for newborn health care services at the HC also decreased since they were able to get the service at the HP, so we think this is due to our support. The other good achievement is the mobilization of the community to renovate the HPs, we have got a good change. Let me give you an example, for instance, at Asteboch HP, the HP is properly painted, chairs have been arranged and the banner was also prepared and posted, so there was some angle the community considered the HPs as HPs. We also made constructions; areas that used to be playgrounds for mouths also fixed and built the trust of mothers to get the newborn health care services with delight. So we did a marvelous job in terms of mobilizing the community. These all were possible to achieve after the commencement of the iCCM/CBNC program implementation.

Interviewer: Have you applied and followed a different strategy in order to improve the iCCM service while targeting gaps identified due to COVID?

Respondent: No we have not implemented anything like that.

Interviewer: How did it improve your linkage and also did it improve the motivation of the HEWs?

Respondent: what we think improved is that the iCCM has also served us as one of the criteria to evaluate the performance of the HEWs, and to create a competition among them, so because of that they have given due attention to the implementation and hence, we think we have improved the iCCM service or put in place some strategy.

Interviewer: How ab in improving the competency of the HEWs?

Respondent: that too. We have improved in terms of looking at their registers and evaluating their competency level through regular support, so we have also tried to improve that.

Interviewer: What is the impact of the support in changing the community's awareness of the iCCM service?

Respondent: What we think improved is the change in the awareness about the service provision at the HPs than the HC. It is a time we have witnessed this.

XX. Maintenance and sustainability

90. What are the high-level benefits that are attributable to this support/IR?

Respondent: what we say we are benefited from this support is the improvement we have made in regard to changing the HEWs' skill. The second benefit is managing to decrease child death. The third is managing to minimize the flow to the HCs and the fourth is also alleviating the suffering of the community.

Interviewer: Do you have evidence about the decrement in child death?

Respondent: No we don't have evidence, but we are not seeing any child death since we have the information and since it is also reported by the HEWs. There is an improvement in child death this year. 1:02:50

91. Please explain to us the feasibility of this support/ IR for national scale-up?

Probe:

- What features could easily be integrated into the existing system? Which not?

Respondent: among the activities I mentioned PRCMM is one of the activities that will sustain and should sustain in order to be scaled up for others. as for our case if there are no new people coming it will sustain, we just need to make sure the activity conduct by evaluating every three months by observing their registers and performance.

Interviewer: How could is sustain?

Respondent: the iCCM assigned focal will make a regular support and if it has to continue there must be a report exchange every week or two weeks.

Interviewer: ok what are the conditions that are fulfilled for this activity to sustain itself?

Respondent: the first is there is awareness created in the community and the second is the HEWs skill have been filled. so there is no reason that it is not going to continue. In addition, there are also 3 iCCM trained professionals at the HCs who will sustain the activities. But if it is aspired to strongly maintain the activities the WDAs need to be strengthened. However, it will sustain if such is not also made since the HEWs have already been trained and are also implementing it now. In addition, since it is also considered as the national KPI and has the support and follow-up at the HC, there is a good probability that it continues. Both the HC and the HPs have got the capacity to implement and sustain this.

Interviewer: What are the activities that may not sustain or are difficult to sustain?

Respondent: there is nothing that won't continue.

92. How are the activities/efforts embedded in the PHC and woreda routines/work streams?
    - What implementation strategies are incorporated with the PHC and woreda annual work plan?

Respondent: as for our HC it is integrated into our supportive supervision including parameters on the number she provided care, how she provided care, etc are incorporated in the supportive supervision checklist. The second is, that it is included in the monthly report and evaluated, and the iCCM focal also presents these reports. The other is when the HEWs hold their monthly meeting iCCM service is the main talking point and agenda.

Interviewer: What are the implementation strategies that you have incorporated into your annual plan?

Respondent: regarding the iCCM, we didn't specifically mention the iCCM support frequency but since it is integrated into the checklist, it is also supported along with the regular HP support we make every week.

93. Do you have anything think is important to tell us that we have not asked you?

Respondent: what I want to add is if JSI could support us with ultrasound for mothers' care since they are the first thing to consider talking about newborn care, and also if the WDAs are also strengthened.

Interviewer: Ok thank you very much.

THE END

End line Evaluation of the PSBI implementation research; Full Transcription for an In-Depth Interview with Health Extension Worker

Questionnaire ID	08
Area Identification	Dembecha
Name of Woreda/Zone/Region	Dembecha WoHO/West Gojjam Zone/Amhara Region
Name of facility	Health Post
Name of moderator	
Name of a note taker	
Date of Interview	20/06/2022
Participant #	01
Audio File #	HP_HEW_20.6.2022
Start time:	10:00 AM
End time:	11:19 AM
Transcriber/Translator	
Duration of IDI:	1hr, 19 minutes

XXI. Fidelity

94. Can you please describe the demand generation/SBCC activities you are doing on iCCM/ newborn health? What are the strategies used to raise awareness? If none, please explain why.

Respondent: Ok, initially after I came back getting the training, I gathered the leaders at the command post and provided them awareness. Then we divided the task among the leaders including me at churches and we provide awareness for the community by even demonstrating the drugs and comparing the one from the HC and the HP, and describing the similarity. We also did the same at schools creating awareness for students. Other than this there is nothing else.

Interviewer: so when you say leaders is it the kebel leaders? And how did you manage to communicate the leaders?

Respondent: yes, it is the kebele leaders which I gather them in one place—we have our regular command post meeting every Saturday. An official letter was also written to the kebele leader which I gave that to him. I told them that the newborn health care is going to be provided as anew—the service of course used to be provide before, but I mentioned to them it will be provided more strengthened and that there is also partner who will be supporting us in this.

Interviewer: What did you want them to do for you?

Respondent: it is to aware the community through them about the service provision here at the HP rather that going to the HCs, and about the medicines available here and at the HCs being the same and the services here also include all under 5 children.

Interviewer: How they also get the community to communicate these messages? What are the ways by which they get in contact with the community for awareness creation?

Respondent: it is mostly at the churches which we usually do it in collaboration that is with the leaders and the health care providers that provided the awareness creation for the community. It was one of the organization member's child that came first to get the health care service.

Interviewer: what organization? You mean the kebele?

Respondent: yes, there was a person who used to work in the kebele here, and his child was sick and he brought her here to get treatment, and then the awareness has started to raise in the community.

Interviewer: Has the organization member's child working here in the kebele contributed to the service provision with regard to the awareness creation?

Respondent: yes, it has. After he got the service here, his wife also told the community that their child was cured at the HP, and explained to them it is similar to medicine that they were provided at the HP with what she once was provided when she visited the HC and told them that it will be a similar service they will get if they go to the HC as well. She has told this to many people and it contributed a lot in motivating them.

Interviewer: Ok, so you have told me so far about the use of the kebele leaders to aware the community for you, what else?

Respondent: the other is at the churches using even recordings together with the leaders to communicate information about the services being provided at the HP, about the medicines here at the HP and the HC being similar, and further communicating why they should waste their time waiting at the HCs and spend costs when the service at the HP is provided for free.

Interviewer: What else?

Respondent: We also used banners.

Interviewer: Tell me more about the banner in detail from the beginning?

Respondent: first when we were trained on newborn health care there was a section about beautifying the HPs. Immediately after I get back from the training, I collected a contribution of 10 ETB apiece from the community, which I used it to paint the HP and also made a partition of the single room we had to two. Then, the HC asked to prepare a banner and then we paid 270 ETB for it from the 10-birr contribution.

Interviewer: What information does the banner has concerning newborn health services?

Respondent: about the service we provide for under 5 children, about the screening service for children, care on children's intestinal parasitic disease, screening and referral of pregnant mothers to health center,

HIV screening and referral and also weight measurement for children. There are at least 17 services listed in the banner.

Interviewer: where are the sites you posted them?

Respondent: it is only here that it is posted. I have not posted it anywhere.

Interviewer: what supports have been made to you related to the awareness creation activity?

Respondent: it was first Gas Kebede who supported us to introduce the service to the community, to go to schools etc, and who provided us first this assignment by gathering us all at the HC. We have also taken the training at Markos. We have the support of many concerned bodies to provide the newborn health care service including this project, the HC and others.

Interviewer: How does the training seem like especially the contribution it has for you to implement the awareness creation activities?

Respondent: well, we could not have painted the HP or prepared the banners if we had not taken the training in the first place. It would have been the same everything like before if it was not for the training. So it motivated us to implement the activities.

Interviewer: Ok, you have mentioned these activities you implemented, and when you see the significance, how effective was in terms of achieving your targets?

Respondent: it was very helpful. Since the HP was also a new one, they would not have come for the health care service. They tell us they didn't know about it before, so it was very beneficial. They are able to come here, and now they have got total belief to get the services here. Previously if I come across someone heading to a HC, and if I try to change his mind to come to the HP instead, he would say there is no injection at the HP and refuse. But now, they have got the total belief to rely and get the services here.

Interviewer: What injection? What do you mean by that?

Respondent: well most of the time what we provide them is drugs in tablet form, and if they don't get injection even at health center level, they would even go to private clinics to get an injection since they don't believe the tablet can give their children a cure.

Interviewer: so, does that mean you are also providing injections? How did this belief improve?

Respondent: yes, we also provide injection for diseases of children which requires administration of injection like Gentamycin. Otherwise we don't provide for any cases. We convince them by telling them that both the tablets and the injections are similarly effective.

a. How is the engagement of communities/WDA networks in the implementation of integrated community-based case management (iCCM) of common childhood illnesses/possible serious bacterial infection (PSBI)?

Respondent: the community is happy but regarding the WDAs, they will not be willing; they are not engaged in the activities, and when we also ask them, they say that they have exhausted themselves a lot for the

activities before and give you such reasons. As for my kebele, they will not be willing. However, the community is delighted to get the services close to them here without going far, but there are some people who still go to the HC—those who find it easy and relatively located near to their residence.

Interviewer: you mentioned that the community is happy but why is the WDAs not happy to engage in the activities? what is their reason?

Respondent: they don't come when we call them unless we go to their homes to get some information. There are very of them, that is only 4 out of the 14 are willing.

Interviewer: what do you think is their reason?

Respondent: they tell me that they have done everything in the past better than us and now they are exhausted, even if I don't know how they did it in the past since I am new here. I have worked here for one year, but I am not saying I am new for the activity since I have worked in other site.

Interviewer: so what do you anticipate about their current reason to refuse to engage that is for those 10 HEWs and what was it before and then?

Respondent: There is nothing I know.

Interviewer: And also, if there is anything you did to engage the 4 in comparison with the remaining 10 who are not engaged about their reasons?

Respondent: by the way they don't tell you about their reasons, and we have also once invited them to come for a meeting through a written letter of invitation having the kebele leaders signature and stamp that is to force them to come but they didn't come to the meeting, they said they will accept any punishment for not coming—one of them told the member of the men development army sent to deliver the letter, which she told him she is not coming and she has no business here. And, only the 4 came but since we could not do anything about it, we sent them back.

Interviewer: maybe if you happen to know any factor behind the 4 WDAs that made them come while the majority were not willing?

Respondent: I didn't ask them about their reasons since I called them for a meeting purpose. We waited—along with the kebele leader who also called them—for the others to come and later we sent them back home. But in the meantime, I was able to tell these 4 to inform me when there are home deliveries and other information in their village, and now one of them is helping me a lot when I go there for vaccination which she mobilizes the community for me. She is doing a commendable work.

Interviewer: what other supports are making the other 4 in terms of the newborn health care service?

Respondent: they mobilize the community. When there is a new delivery, I mostly know it if it is delivered the facility, but when there is home delivery they inform me that there is a delivery and recommend me to come and see. So I go there and check on the mothers and the newborn for any health issue. It is only the 4 that are involved.

Interviewer: SO if only 4 are involved, how are you filling the void left by the remaining 10 WDAs?

Respondent: well if it is the newborns, I usually find them when I visit mothers before their 42 days of birth, and when I also go there for other assignments. Most of them also know about the services but I also aware them to come to the HPs whenever their newborn is sick, and not to go to the HCs before visiting the HPs first. I tell them that I can also refer them to the HCs if it is necessary through an ambulance. They also come to the HPs to get vaccination services after the 42 days of delivery and I ask them where they are taking their children when they get sick, and also explain to them the service availability here and to bring them first here at Kendamo HP when they become ill, but I tell them to come to office rather than Kendamo since it is new and they don't know it by that name. So I just tell them to bring their children to office—the name which they commonly call the Kebele with. Other than this about the WDAs we have even forced them to engage but there is nothing.

Interviewer: Did you discuss about the WDAs challenge as a cluster with the HC and the kebele?

Respondent: not only with the Kebele it has also been discussed at the woreda level, and they are well aware that they [the WDAs] are not engaged. I even told them the same thing when they were interviewing me just like you are doing it now. They [woreda] have also recently passed a direction to register a list of the WDAs by excluding those not engaged, and replacing them with new ones, and then to prepare to give them a training to establish as anew which they have already provided us with a paper form for the registration.

Interviewer: What other social organization other than the WDAs have you used?

Respondent: it is the men development armies. It is them who mobilizes the community for us when there are campaigns, and who goes to the house-to-house visit with us, and also for the newborn health care services which they have come to know more about it since we meet every Saturday. So they mobilize the community on newborn health care and also provides awareness to receive the services at the HP.

Interviewer: How did they become so helpful, tell me more about it?

Respondent: primarily they were with the leaders when we provided the awareness. And, after that they have been alerting me when there are deliveries in the community, and they also call me since they have my number.

Interviewer: what is your work relation with them? How did you manage to get them so involved?

Respondent: when there are meetings I always get myself availed. Even if I don't have much to do, I will be present to help them with their activities. if they have some activity in the community I will also go with them doing their activities so that they help me later too. In the meantime, I also execute my activities while being with them. Since I help them they help me too.

Interviewer: so there is a sense of collaboration among you, that is what you are telling me right?

Respondent: Yes.

Interviewer: do you think this is something different from the reset of the HPs, regarding the participation of the men development armies?

Respondent: when I was working at addis zemen HP the WDAs was better from here but the men's involvement was the same with this one. But I hear from most HPs that the men's development army are helping them better than the WDAs, this is also what I am hearing from most of my friends who are HEWs.

Interviewer: ok what about the reason for the engagement of the men development armies? Why did you think the men development armies have come closer and the WDAs keep away from the activities? you might tell me the experience from other places?

Respondent: there is nothing special done to involve the male development army but they tell me it is because the WDAs has worked for a long time that they are not willing to engage now.

Probe: What are some of the biggest challenges with SBCC activities for newborn care?

Respondent: I don't think there is anything unachieved from my plans.

Interviewer: is there any other form of organization that you have used for the SBCC activities?

Respondent: yes, there is. There are the village health leaders but we have not worked on them yet. We have already selected them and their list is ready, but they are yet to take training.

Interviewer: What other activities remain to engage them?

Respondent: there is nothing that remains unless for our busy schedule. We have selected them at the churches, and it is also said that they will take the training at the health center or health post. So it is only providing the training we remain with, and unless for the interruption due to campaigns that are coming amid this like the COVID vaccine campaign.

95. Could How do you describe the strength of the support you got from the PHC and the project as well as your efforts to address barriers to delivering iCCM/PSBI during COVID-19?
Probe: What was changed?

Respondent: the support especially Gash Kebede used to support us very much. He first came to the HP and showed us practically how to administer drugs. He also called all of us the HEWs at the HC which we have brought our iCCM register, the register for the under 2 months' children treatment register, and he showed us everything. The iCCM focal from the HC and people from the woreda also joined him, and they supported us together. In addition, there are two health care providers who are trained at the HC who support me at times when I encounter difficulty in providing care, I call them and they also come in person and go with me to the household level where the sick child is present. When other supports also come for other purposes, they also ask me how I am doing on newborn health care. Especially, Gask Kebede has nothing but to support us.

Interviewer: Is Gash Kebede the project assigned staff?

Respondent: yes.

Interviewer: How often do they come, including from the HC to support you?

Respondent: regarding the HC, they might not come specifically to support newborn health care, but they do so when they come for other purposes. The frequency of visits is dependent on the availability of different campaigns, which sometimes might not even be possible to come for a month but if they are available, they might come weekly or every three days since there are permanently assigned supporters. Gash Kebede [JSI staff] came three times to support me at the HP and he also called us [the HEWs] at the HC three times; he demonstrated us very well.

Interviewer: Ok you have got these supports in the manner you mentioned. How did they contribute to your achievement? What changes were you able to make because of the support you got on newborn health care?

Respondent: what I was able to change is that we were able to provide for children under two months of age but now we can. He used to come when I was working at Addis Zemen HP, Gash Kebede used to come there, and children less than two months of age also used to come but we didn't know how to provide care for them so we tell them to go to the HC; children less than 2 months used to come with different sickness such as with redness in the umbilicus, etc, which we send them straight to the HC but after Gash Kebede *[project]* and the HC demonstrated us on how to do it, we were able to care for even a child less than 3 days let alone 2 months. It was confusing at first, even if we have taken the training for longer days previously it was a long time ago which we were provided while we were in preschool and we could not remember it. However, the project a lot of knowledge and helped us.

Interviewer: Can you name those things you were able to treat?

Respondent: there was nothing we treat here and we just refer them regardless of which cases. But after Gash Kebede came and showed us how, we were able to provide care for every type of disease. Now we can treat cases for children less than 2 months even in their own homes.

Interviewer: what other supports have you got, for instance, in terms of supply and others?

Respondent: it is with supply. There was a gentamycin shortage which we should have requested, but we never requested whether it is available at the HC or not before. As I told you before since we don't know what to do with it but when Gash Kebede came he told us to request the Gentamycin and others.

Interviewer: How is Gentamycin administered?

Respondent: it is through an injection.

Interviewer: Was it the injection you refused to administer?

Respondent: yes

Interviewer: So is it the skill you developed as a change to later provide the injection from the support?

Respondent: yes.

Interviewer: What other support was there from the project and the HC?

Respondent: there is no other support. We just request the drugs using a requesting form from the HC.

Probe: Was the support you got from the project and PHC helpful? What could have been done differently?

Interviewer: Ok how do you see the support from the HC and the project, was it helpful?

Respondent: I say it was very helpful.

Interviewer: Why was it helpful?

Respondent: I have known many things I didn't know before. The person who comes from the project has exerted a special effort for us to acquire many things; he also helped to take training for about eight days. We also become able on making proper disease classification which we used to do in it arbitrarily before, including the correct prescription of drug dosages which we don't fill every information on the form—we just wright as Amoxicillin 250mg, for some amount per day, but now we are writing the complete information and he even made us post it here on the wall.

Interviewer: Tell me about the training. How helpful was it and what could have been done differently?

Respondent: the training to treat all children below 5 years of age and the conditions when to refer them to HCs, when to treat them at the HP not only the under 2 months also for under 5, and also the drugs we administer which we have known from school but it reminded us all these.

Interviewer: Ok do you have any opinion about the training?

Respondent: No, it was adequate.

Interviewer: Ok, what is your opinion about the support in general from the HC and the project? What could have been done differently?

Respondent: the support has nothing to be corrected but if we were two in number here we could have provided a different support, and there would not any fear to go the most distant places and for the house to house visit since it is a forest and very risky to go by myself. Because of this I only go to the houses which are relatively near, or for some critical cases, and also if I am accompanied by someone which the kebele leader assigns for me, only then I might dare to cross the forest.

Interviewer: is there any danger to move around?

Respondent: it is very bushy. There is one HP which is relatively far and no one dares to go there. Actually, this is my home village I might not fear that much but they tell me others would not have come when they see me there and ask me how made it to come alone—I sometimes take the risk and go by myself when I get tired of asking for the leaders' company.

96. Do you think the support system is helpful (mentorship and on-site coaching methods, supervision, technical support, PHCU level PRCMM, etc.) to enhance your skills [that is HEWs' skills] over the traditional off-site training and woreda level review meetings?

Interviewer: the question I am going to ask is about the support you have been receiving here and also at the HC, and the support you got to like the training provided offsite, which do you think has improved your skill more?

Respondent: the one which I think is better is the training we got at Kosober for seven or eight days which provided me with a lot of knowledge. The onsite which they did here is also good and made corrective actions but I have got a lot of things and learned in a manner which I understood in the training.

Interviewer: When you were telling me earlier, you also mentioned that you were trained first somewhere but the onsite support helped you to kick start and capacitate you to give the newborn care which we were not able to do?

Respondent: when we were in preschool we have taken some training but that has already disappeared, later when they were supporting us here, they interrupted and gave us a similar training in the meantime. Regarding this training, I got a lot of things and was able to immediately start working and this is not comparable with the training I received in preschool.

Interviewer: so there were the supports that you have been provided and when we compare this training with the onsite support here which one is more helpful to develop your skill?

Respondent: it is still the training that was provided for longer days.

Interviewer: What makes the training helpful compared with the onsite support?

Respondent: they have of course demonstrated me on how to make disease classification and treat children less than 2 months of age but the training I took has every component on how to treat children starting from zero to 5 years of age. We have practiced on the provision of treatment using the chart booklets, and on disease classification; we even went to a health center during the training and learned how to measure temperature and counting of the heartbeat which they demonstrated to us on a real child who has just been born. This is how they trained us, and due to those approaches, I was able to acquire many things.

Interviewer: Do you have any opinion on an improvement that should be made on the onsite support?

Respondent: what I want to say is that the training is much better than the onsite support, but I am not referring to it as not good. It was them of course who showed us how to classify disease in the first place, but I am just saying the training was better. The project support was specific to children less than 2 months of age, and if they come to support the under 2 months of health care, they only deal with that and don't widen it to the under 5.

Probe: for acceptability of the strategies **(for HEWs only)**. How could the support system be improved?

Respondent: I have accepted the support very well.

Interviewer: Anything else you want to tell on why it is acceptable by you or any improvement?

Respondent: it is very nice generally, but one thing they don't come more often [the project]. The support should not be interrupted by the occurrence of different campaigns, well if they can't make it, there is the HC of courses that could provide us support. For instance, when the project was actively supporting us they used to come once per month but it would have been great if they could have come every week, for instance, Gash Kebede came only three times since my assignment here even though he makes good support when he comes.

Interviewer: So you are saying the support should be more frequent and should not be interrupted along with campaigns.

Respondent: yes.

97. How eCHIS implementation helps you with iCCM service delivery?

Probe for advantages:

- case identification? quality of iCCM case management? retrieval of client records? data quality? client appointment scheduling and/or defaulter tracing?

Respondent: the eCHIS notifies us if there is a new birth within the same day of delivery; the HC registers and syncs it for us, which we then know which mother has delivered and visits her.

Interviewer: How about in the identification of cases for iCCM?

Respondent: We have not started using it for the identification of cases yet. It is only for registration [household level] and vaccination service we have used it so far. We have not used for the newborn health service but it is helpful to know about a newly born child. If for instance, this was through a paper-based one, I will have to go myself and see if any mother gave birth, or the WDAs need to inform me about the delivery in the community otherwise, it will be after 3 or 4 days that I will know, however, using the eCHIS I would know in the same day of delivery since the HCs syncs it.

Interviewer: what other services are integrated in the eCHIS other than the newborn health care?

Respondent: we use it to provide family planning, for ANC1, ANC4, PNC follow-ups, we can also use it to organize the 1-5. The tablet is also used to send reports to the HC; for instance, if there is a mother PNC conducted we use it to send the report for this mother.

Interviewer: How about the significance of the eCHIS in ensuring quality?

Respondent: if this was in the paper I may lose it but since it is in the tablet, they might have a backup and retrieve it. If I record a mother wrongly in a paper form I will have to erase it but on the tablet, it is possible to replace her or delete her.

Interviewer: Would it be helpful in recording? Easy retrieval etc.

Respondent: For instance, using the paper-based system, how many pages should we have to turn to find one record. But in the eCHIS if write the house head's name, it will list down everyone in the family.

Interviewer: What about scheduling, defaulter tracing?

Respondent: yes, it traces that for us.

Interviewer: How?

Respondent: it indicates us when it is their schedule and flags when it reaches their deadline, by changing the different colors.

Interviewer: Ok if we were able to use fully the eCHIS for the iCCM service, would it enable us to effectively identify cases of neonates?

Respondent: if the iCCM services is installed in the system I think it is doable, why because when we are going to the house to house level for the iCCM service we are using part of the register detaching from the main registration which we use it to record the disease classification and others, then we transfer it here to the main register. But if this is available in the tablet, we can record everything in it.

Interviewer: again how would it contribute for the data quality if iCCM was integrated in eCHIS?

Respondent: it will not allow us to go to the next step if we make an error. But if it is the paper I am using, I might write it wrongly, and will not take me back. I think it will be good if it is integrated.

Interviewer: can we also easily identify or retrieve records if iCCM was integrated?

Respondent: Yes, it can because there is an identifier. For instance, in order to retrieve a newborn in given Gote, I will go to that Gote first and then when I write her family name, she will be displayed.

Interviewer: do you think it will help you correct errors you normally make in the paper based iCCM, if this was integrated in the eCHIS mainly in scheduling or default tracing?

Respondent: I think so, since we are seeing it helps for the other services but we have not seen it being used in the iCCM yet.

- **Ask why?**
- **Probe for areas of strengthening?**

Respondent: Ok what I think should be strengthened is that the tablet is not fast and usually takes time and delayed to skip through pages. So, if there is better tablet and be replaced.

98. How was the introduction of the iCCM/CBNC module into eCHIS affected the iCCM/CBNC service delivery?
FOR LUME WOREDA ONLY extra

XXII. Implementation challenges

99. Describe the main issues faced by the health system to identify and treat neonatal infections in the community. What are the critical factors affecting the delivery of iCCM to clients?
Probe:
    - lack of competence of HEWs, shortage of supplies and commodities; weak support system; low community demand?

Probe:

- Regional/national state of emergency and conflict in the northern part of the country?
- How has that changed during COVID?

Respondent: one of the challenges is that our kebele is very wide in the area and it is not possible to cover with just one HEW, we have to be two.

Interviewer: what else, you mentioned the lack of frequency of support, what else?

Respondent: the other is from the HP being located in the kebele office is creating discomfort among mothers to take family planning service. If you have noticed earlier, there was a farmer who is here for another purpose at the kebele, but the mother was not comfortable coming in and using the family planning service. All people, who come for fertilizers or to get new IDs, and those for family planning services also wait in the same place outside, so it is different from what I used to work in another place, and the HP should be relocated to some other place.

Interviewer: What other challenge do you have?

Respondent: I have no other challenge except this, in fact, Gash kebede [JSI project staff] has praised me as one of the good performers whom he was pleased by the improvement I made.

Interviewer: Ok now tell me about the influence the conflict or the war brought on the newborn health care service?

Respondent: It didn't bring any influence on the health care service provision, but they say when we were in the community for the COVID vaccine campaign that COVID is not their main concern, it is rather the war that it is their concern.

Interviewer: may be, anything related to the war that may have had influence in the newborn health care service provision? Any attention diversion, and the interruption of services if any?

Respondent: there was nothing interrupted, and it rather has helped me in that I used to go and move along with the leaders when they were going to the community to collect contributions for the war cause which I used to it my advantage in providing care for newborns as well as the mothers. It did not inflict any suffering on me.

Interviewer: what about the state of emergency, movement restriction and its impact when it was declared and active? Did it have any impact on the services?

Respondent: No there was nothing.

Interviewer: For instance, in limiting the community's movement or restricting them to come for the services?

Respondent: No. It didn't have any influence.

100. How do you think the COVID-19 pandemic and/or COVID-19 response measures affect the uptake and/or delivery of iCCM services?

Probe:

- Has COVID-19 affected your daily routines; your work on newborns; the community in terms of livelihood and vulnerability for newborn care-seeking
- How has that changed over time?

Respondent: by the time COVID occurred, I was working at Addis Zemen HP and there was no impact. The community also has Zeleka HC near them and they were going to this HC and using the services. Since they have only these HC and the community has nowhere to go, there was nothing interrupted and they were using the services. We were also going to the house-to-house level and creating awareness on COVID prevention and control measures. Since we were two at the time, we used to work in turns that is when I am in the field engaged with the kebele leader, she stays at the HP, and when she is in the field and I engage at the HP. So there was nothing that diminished in performance.

Interviewer: I mean there were a lot of reports on COVID impacts around the time it occurred, and what was the situation with you during that time, and the influence it had on your day-to-day routine activity?

Respondent: We used to move around a lot at the time COVID occurred; people from the woredas also used to come more frequently to support us and make inquiries of our performances; they used to ask the community if we have provided them education in our absence. There was a lot of people that was scared of COVID of courses but we continued to provide newborn health care service while we also provide awareness on COVID, so the performance didn't decrease. The community also didn't avoid us.

Interviewer: so are you saying there was no any influence on the newborn health care service due to COVID?

Respondent: there was none.

Interviewer: Was there no any kind of fear on COVID by the people and also from you? What was it like?

Respondent: people were scared of COVID of course but we told them about the prevention and control measures, we availed them handwashing facilities at the doors, and we also informed them to bring their children if they get sick and not to remain in their homes. They were just only afraid of going to the towns, and other than this, there was no challenge to come.

Interviewer: Why were they afraid to go to the towns?

Respondent: They were afraid to the towns and they don't take their sick ones to the hospitals because they think they would snatch their newborns from them for COVID reasons. They used to fear that if their kids have flu they would take them away at the hospitals thinking that it is COVID.

Interviewer: Any challenge related with fulfilling the protective measures from the community side and you?

Respondent: they used to bring us from the health center; face masks and sanitizers, and it was even forbidden to work without applying those measures; we used to be told that if any HEWs are found working without a face mask is going to be fired.

Interviewer: so can we say there was no change of any kind while working with the COVID from the beginning till now?

Respondent: well the community was very fearful at the beginning but now there is a tendency of forgetting about it. And, when we also went to the community to provide them with the COVID vaccine, they ask "what is it doing here" when it should have come earlier.

Interviewer: what is the current situation with COVID after you have been reassigned here?

Respondent: No they don't have fear for COVID. They might have had fear when it occurred I am not sure but now their heart is on the war. As I told you, they say "did I say COVID killed me, it is the war that is killing us".

Interviewer: does it have any influence on the newborn health service that is their heart being on the war?

Respondent: No, only when they relate it with COVID, otherwise there is none. When you tell me to protect themselves from COVID that they tell you COVID didn't kill them, rather it is the war that is killing them—they might give you such response.

XXIII. Adoption and reach/effectiveness

101. Explain to us any changes you observed as the result of the implementation of the COVID-19 adaptive iCCM implementation strategies? – What impact have the strategies had?

Respondent: as I told you, the different supports have brought numerous changes. The support o has helped us to treat those children below 2 months, and the time we start to worry get worried is after the support. It is also after the support we started to stay and work at the HP but before that we used to close the HP and focus on the house to house level activities engaging in the other packages. Currently however, even if we go out to implement other packages, there is no time we didn't also check on the under 2 months' children; back then I was not identifying and registering cases of newborns unless it is for the post-natal care.

- What are the particular features of the strategies that made a difference?

Interviewer: Which among the different supports is one you think has made a difference?

Respondent: it is the support I got to provide care for under 2 month children.

Interviewer: tell me about the specific support that made you able to provide the under 2 month children treatment?

Respondent: previously it was only when children above 2 months of age came that we use the chart booklet. But after they showed us, I have become able to do treat the under 2 months as well.

Interviewer: is it the support they provided you here?

Respondent: Yes

Probe:

- for a support system and linkages, motivation, and competence of HEWs, community**,** and awareness creation?
- What do you think are the reasons for non-significant changes?

Interviewer: how about the significance in improving your linkage with the HC, has the support contributed fir this?

Respondent: it is also one aspect to consider, even if they also used to support us before except for the under 2 months. It contributed for the linkage but it was also there before, and it is good and they used to come on daily bases when I was there.

Interviewer: how about the contribution of the support for changes related to improving your motivation to work in the community?

Respondent: yes, it helped. This is because I consider the reason why this person [JSI staff] is trying to help us at this age of his is because he wants to save children which motivated me even if I already have the motivation to provide care, but the motivation from the previous time to provide care has increased more from the support.

Interviewer: what is it that you say have not made much changes despite the efforts being exerted?

Respondent: there is nothing I say like that but when we commence the implementation first, it was communicated to trace those newborns with severe cases but it was not possible to find them, like those who have had convulsions, and with the difficulty of breastfeeding. However, it was tried but it was not possible to find those kinds of cases.

Interviewer: do you think you had adequate support regarding the skill to identify those skills?

Respondent: I believe it was adequate, and the reason they were not found is that there was no case.

XXIV. Maintenance and sustainability

102. What are the high-level benefits that are attributable to this support/IR?

Interviewer: what is that you have benefited highly from the supports?

Respondent: it it being able to provide care for the under 2 months' children. ***[not probed further because it has already been mentioned above following this same answer]***

103. Please explain to us the feasibility of this support/ IR for national scale-up?

Probe:

- What features could easily be integrated into the existing system? Which not?

Respondent: the newborn health care services can sustain without any further support since they have coached us well. It is also a service that we should have provided even before the project support since it was one of our tasks to treat children—it has to do with our negligence and we also did not ask for support. Anyways, the newborn health care service is going to be maintained regardless of the availability of support unless there is a shortage in drug supply since we have the guideline and register. I believe we can provide the service.

Interviewer: Do you think this is scalable at the country level?

Respondent: Yes I do because we were first trained when we were at college, it is our blame for taking.

Interviewer: what about the features of the support that you think is difficult to integrate in the existing system?

Respondent: if it is something that we started working on, and face difficulty in the meantime we can ask for support from other experienced HEWs and maintain the activity. However, if this is new and something someone told you to do it only, there might be a chance of discontinuation. Otherwise, if it is something that we already have been working, how come we interrupt it. We can do it, and I am not also going to cease working because some project that used to support me is not more coming.

Interviewer: Do you also think the support from the HC will also continue afterwards? From now on?

Respondent: I think they will continue supporting us while they come for other activities, and since they have still been supporting us integrating it with the checklist, I think they will continue doing that. I don't think they also decide to stop because Gash Kebede has stopped coming.

104. How are the activities/efforts embedded in the PHC and woreda routines/work streams?

- What implementation strategies are incorporated with the PHC and woreda annual work plan?

Respondent: It is included in the report and also we register them in the registration. We make a report to them every week on Friday.

Interviewer: What are the things that you report and to whom do you report every week?

Respondent: it is Sepsis which we make the formal report every month, but for the weekly report they call us and collect the report; they ask for how many newborns we visited even if they are not sick and the number we provided care for the sick ones out of this total. We report them from the register. The paper-based report is made on monthly bases.

Interviewer: what else, has it been embedded in your routine house to house level visits?

Respondent: Yes, Gash Kebede [JSI staff] gave us a page detached from the main register to use during our house to house visit. We also have our pencils we bought here, and this is how we integrate and work.

Interviewer: Have you integrated in your plan and is it evaluated?

Respondent: yes it is incorporated within the plan, and we are evaluated based on the plan during our quarterly evaluation at the woreda; I have been personally evaluated and I recall being asked why the Sepsis cases decreased and also the local bacterial infection, and if it is because I am not going to the house to house level, etc.

Interviewer: so you are saying it is embedded in this manner.

Respondent: yes.

105. Do you have anything think is important to tell us that we have not asked you?

Interviewer: If you finally want to say anything that we didn't discuss or something you want to add.

Respondent: I believe I have said all that is to be said, and I don't have anything to add.

Interviewer: Ok thank you very much for your time.

THE END

End line Evaluation of the PSBI implementation research; Full Transcription for an In-Depth Interview with iCCM Focal Person

Questionnaire ID	07
Area Identification	Dembecha
Name of Woreda/Zone/Region	Dembecha WoHO/West Gojjam Zone/Amhara Region
Name of facility	Health Center
Name of moderator	
Name of a note taker	
Date of Interview	10/06/2022
Participant #	01
Audio File #	_iCCM Focal_10.6.2022
Start time:	02:00 PM
End time:	09:12 PM
Transcriber/Translator	
Duration of IDI:	2 hrs, 12 minutes

XXV. Fidelity

106. Can you please describe the demand generation/SBCC activities you are doing on iCCM/ newborn health? What are the strategies used to raise awareness? If none, please explain why.

Respondent: well regarding the newborn health care service, there are activities that are being implemented by the government. In addition to this, there is a project implementation by the JSI L10K especially with regard to providing training for our HEWs; there were HEWs that were being newly assigned and the existing HEWs which the project also provided them a refresher training. The training has been helpful for the activities that we implemented afterward, and the JSI L10K training provision has made it possible for us to make onsite support for the HEWs. Other than this what we did was on the services for diseases that are known to be curable but fatal such as Tuberculosis, Dysentery, and uncomplicated malnutrition which are mostly perceived by the community to be provided normally at hospitals and health centers. However, after the HEWs were trained about the services, awareness was created about the provision of the services by the HEWs. The community had no adequate awareness before about the services but after the training, it was communicated especially to the Kebele leaders and the WDAs to create awareness in the community; there are community leaders or the WDAs selected by the community itself and these leaders were

communicated to create awareness in the community. Not limited to these social organizations alone, we also provided awareness using places where the community gathers most like EDIR, churches, or religious places where effort was made to aware create about the service provision at the HP level with the utmost ease to the community. Other than this, there are political organizations that we also used the opportunity to aware them in a brief manner, using tailored approaches and in a language common to them. We told them about the service is available for free, and that they don't have to go anywhere else, so we have communicated adequate information which suffices to get these services at HP. Based on these, we also think we are successful in our effort, for instance, we have 6 rural HPs in our catchment, and the services in all the HPs are being provided without any interruptions and at any time. We also go there for support which we make every month, and when we go there, we observe that they are providing care for newborn cases of diarrhea. When we try to evaluate how they are performing after the training, we have witnessed their care provision was good and most people were also coming to them to get the services—most were doing well and progressing according to their plan. Maybe with regard to Sepsis health care provision, there were some gaps in understanding but in terms of the other services like Tuberculosis, diarrhea, uncomplicated malnutrition, malaria, and trachoma, they were provided in a coordinated manner and all the evidence we reviewed including the field observations indicate that the community was also utilizing them.

Interviewer: Ok, maybe on the provision of care on Sepsis you mentioned, what is that you faced? Why was it low in performance? Was there any challenge related to the awareness creation activity?

Respondent: Yes, with regard to the health care on Sepsis, the HEWs don't have any gap but the community had some perception, for instance, they might say they won't take their newborns to a medical care before he touches holly water [baptized], and such beliefs might be reflected by the community. And, there have been efforts to create awareness using religious leaders but we have not been that effective. There were however some who came to get treatment and got cured, and it was these newborns that we were also using as a testimony to aware the community, but still, there are huge gaps that we need to work on. Hence, I don't think the utilization of the Sepsis care is as anticipated.

Interviewer: is it the time convenience or what is it with the holy water issue you mentioned? If you can elaborate more?

Respondent: As you might know, with the Orthodox Christian religion if, for instance, a newborn stops breastfeeding after two or three days of his birth—we think this might be due to probably Sepsis right? but the mother would say how could we—both him and her—go to a medication without being blessed by holy water and it is putting out a temptation to the Gods; this is not a good belief of course, but because of this religious shielding, they tend to remain home instead of seeking medical care. Mostly when we go to a house-to-house level in the community we also encountered

newborns with difficulty of breastfeeding and not taken for medical care, and this makes us realize to do more. What we did as a solution was that we tried to showcase those treated and cured as a testimony to convince others to come to the services, but we are still behind in achieving the expected result and the community practice in terms of coming to get the services in this aspect has been a bit low for us for reasons I mentioned before.

Interviewer: How many days do they stay without being baptized in this area?

Respondent: at least 2 weeks; that is after she delivered, she waits for 10-15 days considering both Sexes, and it is after this time and she had the holy water that she goes to the health care facilities. And so, there are such cases.

Interviewer: so my thinking is that they don't come for any illness not only for Sepsis unless she had holy water, is that it?

Respondent: yes, but they come after that. So why we tried to use those treated and cured newborns within this time is to convince them that it has no relation and implication to their religion and that it is curable.

a. How is the engagement of communities/WDA networks in the implementation of integrated community-based case management (iCCM) of common childhood illnesses/possible serious bacterial infection (PSBI)?

Respondent: the WDA network is available in every kebele, and they have a monthly meeting. Under the WDA there are also the 1-5 network leaders. What we normally do using the WDA is that we give them training during their monthly meeting on different issues, and one of these is about the iCCM service; providing insight on what it means about the newborn health care service in order to increase their understating and awareness. We also aware them to look around their neighbors to see if there are sick children and to refer them to us if any. We provide this training taking a long time about 2 days maximum which they come and go every day for the training. We make the training venue to be center for everyone and considering the different villages which are also relatively far. So based on this they participate in the training, and as I told you when they come for this monthly training day, there is also a monthly performance evaluation conducted along with the training, so concerning the evaluation, they present their performance which we start from the number of clients they referred and other evaluations based on the different assignments we give them, for instance, the type of gaps they observed in their village or if there were pregnant women in their village, we look at the number of ANC visits they managed to do for her, and encouraging them on their performance. sometimes, we also invite the kebele leaders to encourage the WDAs during this meeting to improve their performances.

Interviewer: What is the frequency of the training?

Respondent: there is a regular training which they take every month based on specific titles and focus areas but sometimes there may be some competing interests like if it is COVID campaign, Polio campaign and other activities implemented in terms of TT surgery, and if there are such emerging priorities and programs we also provide their training integrated with the regular program. So we have the regular meetings and training, and what we did for the regular meetings not to interrupt we also aligned it with their monthly religious gathering for the holy chalice practice [TSIWA] like Holy Chalice in the name of St. Mary or St. Gabriel, etc. So we do this with the aim of strengthening their interaction, and as a means to bring them together and increase the time they spend together. So during this day, they don't engage in any other activity and respect the day, and based on that they present their reports and we also provide them the training.

Interviewer: Have you seen any change from implementing this activity? Was there any change and have you observed anything related to the iCCM service delivery?

Respondent: Yes there have been changes, for instance, we educate them on those regarded as danger signs to a level suited for WDAs, and as a result, WDAs have been produced who can properly explain the danger signs when they occur in the community, and when they observe such signs they also immediately refer them to HPs or HCs; because we have created the awareness in the danger signs and also other services to the WDAs, not the community behavior in terms of visiting the health care facilities for medical care have improved, which has not existed before; since they don't have the awareness many newborns used to die, and when they also come to this facility it was after children were severely malnourished or severely affected which we used to struggle to manage, but nowadays most of the children are able to be treated and cured at the HPs. We also treat those cases that are beyond their capacity here, and as a result, the community is building trust in the HEWs and the provision of medical care at the HPs. Previously, since it was only health education that the HEWs used to give to them, the community's trust in the HEWs was not that strong but now when they observe that they are also providing them medical care, and when they observe children get cured their trust on the HEWs has increased and it is becoming a family like relation which the community consult every issue and seeking advice when their newborns get sick. This is also what we observed practically when we go out for supervision which they were properly discussing the progress of the health condition of their child and what they ought to do, etc. So we think that the awareness level of the WDAs is in good condition and as a result, the health-seeking behavior of the community in terms of visiting health care facilities has increased.

Interviewer: is it only the WDAs that you have used or any other association that you have used other than the WDAs?

Respondent: it is the WDAs that are helping us to a greater extent in our activities at the moment, other than this, there are also the men and young associations but the role of the WDAs is much greater than the

rest since most of our activities centers women. However, we also engage men to have the influence of husbands which we make them attend sometimes when conferences are organized or in similar events, but the rest don't have much influence as the WDAs so we use them as required such as the religious leaders depending on different circumstances.

Interviewer: is there a form of social organization called village health leaders?

Respondent: Yes, there is a village health leader. It is a link and serves us as a bridge between the WDAs and the HEWs; it collects every health-related information in that specific village; they plan activities jointly with the WDAs; it executes all health-related activities in that village in a coordinated manner. These village health leaders have also taken the training within the last year and started the implementation. Similarly, these WDAs also register and archive all information about each household, so they consider the newborn health care services, under 5 and all other services as part of their plan, and participate in the activities serving as a bridge between the WDAs and HEWs to deliver the different services, so it means the village health leaders also indeed participate in the activities.

Interviewer: How do you see their role in the iCCM service delivery? Is there anything that they brought as an association? What is their special role in the activities?

Respondent: well what was the challenge seen with the WDAs is that there was some boredom among the WDAs from working for a long time, and even though the WDAs had the community's acceptance—since they originate from them, there were however very few of them who were able to read and write, and also able to receive a plan and demonstrate proper implementation. Especially in terms of withstanding the influences coming from their husbands, they were not up for it. So since the role was based on their will as a volunteer, they were showing boredom due to their long years of service but after the introduction of the village health leaders, it was like having additional support for specific village activities which also imply that there is a concerned person for the village. It was the WDAs who in the previous times were concerned about the villages who might be available in 3 or 2 numbers per village, and there was no anyone responsible to organize them before, and there were challenges in this regard which were manifested with variable performances among the different WDAs and also among the 1-5 association leaders even within the village [gote]. However, after the introduction of the village health leaders, there is ownership of the village [Gote] working as one village [Gote], and the VHLs also happen to serve as a helping hand for the WDAs since they can write and read, and since they have also better educational background and understanding than the WDAs, hence they support the WDAs in the villages with report compilation, and in coordinating of the WDAs themselves found in that specific village.

Interviewer: if you can focus on the newborn health service and the change in relation to this obtained as a result of the VHLs?

Respondent: in terms of the iCCM service delivery, for instance, the flow of newborns that have been referred to the HPs has improved since the VHL introduction as per our observation. For instance, there used to kebeles that had very iCCM performance among the 6 kebeles we have in the catchment, however, after they arrived especially in terms of providing counseling about the newborns, and referring them to the HPs, they had a good performance, and this is also because they were well trained about these components also on the specific danger signs which capacitated them to make proper referral upon their return to the community from the training. When we also evaluate what these village health leaders implemented from their reports as well as during our supportive supervision, I think they had a good implementation in terms of socializing with the community and managing the referral of cases from the villages to the HPs. For instance, in some villages the referral of newborn cases to seek medication at the HP was not good before the VHLs participated but now since they now know each household and recorded them well, they have been supporting the community during their challenges, and helping them to get medication by the HEWs at the HPs. And, since they had also a good understanding I think they have brought a commendable change compared with what we had before.

Interviewer: Who supported you to realize the establishment of this social organization that is the VHL?

Respondent: well who supported us with everything is the JSI L10K. They have first provided the ToT training for trainers and then facilitated for the trainers to recruit the village health leaders from the different kebeles [Yegote Tena Meri] in collaboration with the HEWs and leaders in the Kebele. JSI L10K also had a bigger role in the successful organization of the training that is coordinating the proper training conduct according to the schedule, etc. in addition to this, the WoHO and Zone HO had also a contribution but it is the JSI L10K who had the lion share helping us to acquire the current achievements, and we really thank the project for that.

Interviewer: Is there any other approach that you have also used to aware the community about the iCCM services, other than the WDAs and VHL that you have told me? What SBCC activities have you implemented?

Respondent: it is at the churches and providing education where people gather and inform about the advantage of the services and the consequence if they don't use the services.

Interviewer: For instance, using banners and posters and other similar SBCC activities?

Respondent: well regarding the type of services provided at the HPs, they are listed out on a poster and displayed at the HPs. I am afraid these are the only activities we implemented.

Interviewer: For instance, you might identify some perceptions and rumors in the community in which you use a banner or poster to aware the community about it? that is rather than just listing the services at the HPs. Was there anything like that you utilized?

Respondent: No there is not.

Interviewer: Maybe, was it your interest to use such methods but you had a limitation, or what was the reason that you didn't use those?

Respondent: regarding the perception in the community there is a variety of them, for instance, in our area, there are cultural harmful practices that are being practiced for newborns. You know there is such saying about the newborns head that is the fontanel remains open and as a result, most newborns are taken to a traditional healer which is located at a place called "Yeckereka", which she treats them using by putting on some herbs on heads of the newborns—the traditional healer is an elderly woman with religious devotions [EMAHOY]. They also say that they got healed afterward, but most newborns as a result of this are dying out of hypothermia and Sepsis which is the common reason they are dying, and especially there is the tendency of visiting the traditional healer among those firstborns or pregravid. We have discussed the issue as a health center, and we have informed the existence of such practices in our catchment for the health care providers and the HEWs, and by chance, this issue was also raised among participants of WDAs while we were training them; they mentioned and explained us the existence of this practice. Despite our efforts, this practice has been widespread in the community, and this woman also costs them a lot which at first was about 50 ETB but gradually increased to 1000 ETB which she receives from them. Our observation is that most children are dying from hypothermia exposing them naked when she puts off all their clothes to put on the herbs on their bodies. We tried to convince the community using these facts but it has not worked out well.

Interviewer: Ok what is that you have diagnosed from the medical aspect in your investigation about this complaint from the community? What is confirmed about what they are saying about the opening of the head in newborns from your side?

Respondent: Ehhhh [laughter], well the children are distinguishable when they come after visiting the traditional healer, they make a wrapping around their heads and they also gasp. They become anemic and usually severely ill when they reach here. What we got as information from interviewing some people, is they say the fontanel does not have a pulse, and it also remains open. They say such things. So we try to tell them the fontanel might take time up to 2 years to mature and about the anterior and posterior fontanels as well. And that, since it is a natural occurrence it is not worrisome, and sometimes during delivery, if there are injuries it might not be pulsatile. We also tell them these things can be confirmed by checking their heartbeat and that, infants after delivery should not be naked and kept warm with proper clothing, etc. So we usually tell them those, and there are people who brought their infants for medical care, but overall these are some of the challenges we faced with regard to the community perception.

Interviewer: Are there any other similar challenges you faced in terms of making behavior changes in the community?

Respondent: these are all the major challenges we faced that we identified regarded as major hindrances to providing the services.

Interviewer: what about the functionality of the WDAs, is it playing the role it should as expected in terms of the SBCC activities?

Respondent: most of them are helping us, especially after the introduction of the VHLs they have been motivated and supporting the activities. The WDAs are supporting us with iCCM activities, especially with regard to the under 2 months children's health care in the community and providing awareness for the prevailing community perception. The WDAs compared with the past are helping us in alleviating the challenges related to the community perception and sending community members to seek medical attention which was poor before and their understanding is showing improvement from time to time, even though there are still some gaps that need some training and the influence of husbands is also challenging us.

Interviewer: What is it about the influence from the husbands?

Respondent: well the WDA rounds and moves within the village, and the husband otherwise wants her to incline to her household chores, also because the service is provided for free and does not have any benefit. There are some women who withstand this and continued with the activities and serving their communities, who continued providing education, engaging in children's vaccination, identifying those danger signs in newborns that could lead them to death, and referring them to health care facilities, so there are still such WDAs who are implementing the activities despite the influence of husbands, and the husbands or men's participation in the social organization is not that promising since they left all the responsibility for the WDAs. Similarly, the role of the religious leaders in advising their godchildren to go to the health care facilities is still not satisfactory, but a lot is expected from them and yet it is immature, and a lot of expected to do in this regard.

Interviewer: What are some of the issues that prevented you from doing what you plan to implement?

Respondent: as per our plan and if we have adequate time, I think it is good if we can get the religious leaders and discuss the newborn health care services I think we can achieve a good result if we do that. Also, with other social organizations if we can gather them at the HC level and discuss the community perceptions prevailing regarding the newborn health issue I mentioned earlier. So in order to correct the community perceptions, I think it will be good to have an opportunity to meet with them which would bring the anticipated result.

Interviewer: what is preventing you from doing that?

Respondent: In order to organize a meeting and such activities, there is a budget required and as you know there is also a limited budget at the HC level and impossible to organize such meetings but at the HP level I mentioned to you there are religious gatherings like for the holy chalice which we invite the religious leaders to bless the meeting with which we also explain to them about the services, and give some

assignments. However, in order to evaluate what they did and the activity status, there are limitations as I mentioned with the budget shortage which makes it difficult to have such discussion about the performance status of the assignments with the religious leaders. In addition to this, there are also those known as community key figures who are influential in their own villages, and if we can also discuss with these people, to aware their community and pass strong messages using the different opportunities they get to their community which could be Ekub, Edir, etc, could play a vital role in correcting the wrong perceptions reflected by the community on the services. Maybe to ensure the quality of the services, I think it is nice if there is a platform which the HEWs can also meet and discuss, for instance, every quarter which they can meet to learn experiences from each other in their newborn health care service provision, and I think both the health care service for under 2 months and 59 months will also improve if this platform is facilitated for them. Currently what we do is there is the monthly PHCU meeting which we meet every month and the newborn health care service is one of our activities which is divided among the HPs to evaluate which has provided the standard care, even further divided among the specific HEWs to determine which has treated better and which has gaps including the assessment, classification and treatment aspects of the care and all steps related to her appointment/schedule setting and drug administration. We also make support in person at the HPs to see how they are implementing the activities, however, it will be strengthened more and better outcomes will be obtained if meetings of such kind are also organized.

Probe: What are some of the biggest challenges with SBCC activities for newborn care?

107. Could How do you describe the strength of the support you got from the PHC and the project as well as your efforts to address barriers to delivering iCCM/PSBI during COVID-19?

Respondent: well generally the support from JSI is immense in many aspects, including the training for our health professionals which is unthinkable during the COVID era, and holding on to the challenges it has managed to provide knowledge for the professionals and also produced a health force which could support the HEWs through the provision of training. It was only a few of us who used to support the HEWs but JSI trained additional new health professionals who could support the HEWs to be able to scale up the services in all the kebeles, and it created us the opportunity to divide the kebeles adequately among us and eased us the challenge. Hence, it tried to have a coordinated effort to have the availability of the services at the HCs and HPs, which I think was successful. Generally, we at the HCs as I said also held monthly, and also annual, and quarter performance evaluations which we support, and similarly, the WoHOs also have their own. So we support the HPs every month to see how well they performed, and we also support iCCM every month. Every quarter there is also integrated supportive supervision comprehensive for every service, and the WoHO also has similar

supportive supervision which they make every quarter. There is also an officer at the WoHO who supports maternal and child health services. In general, it is my observation that the support level has improved from the previous times after the project implementation started.

Interviewer: if you can compare and tell me the difference prior to the project and after the project?

Respondent: for instance, before the project we had no regular support on iCCM every month, it was only maybe once in every quarter or per annum, and the support was not intended to deliver knowledge to the HEWs or in-depth one, rather it is just for the sake of doing it or if we are given a direction to support well the hygiene and sanitation activity, we just do that only and leave. So I don't think the HEWs were being supported but after the project came to the realization the iCCM service was properly being supported and doing so also helps us to have the chance to properly identify the gaps, adequate time to work on the HEWs knowledge and provide solution on challenges related to supply and other challenges. For instance, we used to find drugs available on shelves long after their expiry date passed, so we provide support on the availability of the supplies and if not we provide problem-solving suggestions on spot, and we also facilitate conditions to withdraw the drugs, and we also check the registration if there is a gap in the quality of care, or of the assessment or classification was correctly made or if the relevant child was referred who should be referred or if an appointment was set correctly and if the child had the right treatment on that day, etc, which we coordinate all these, took our time and jointly made the proper support. But before this, iCCM was not our focus, and we used to go for support taking the iCCM service as one of the activities but the priority was for other emerging activities. The improvement in our support also improved the competition among the HEWs to provide better health care services, in addition, Sepsis care used to be feared among the HEWs to provide, and they used to struggle to provide this care due to the supply and skill-related challenges they had but we tried to show them the proper care provision based on the standard treatment guideline, and now those kebeles who were regarded as silent have also improved in their knowledge and skill from the support they have received including the support from the JSI which they have trained our professionals at the beginning. Especially on the newborn health care service as I told you I have stayed for a long time in this HC and when I compare it, I recall those silent kebeles that don't treat any cases; it is not because the cases were not there but because they lacked the proper knowledge and skill to do that, and they used to refer to the HCs instead of treating them at the HPs, but after we coached them on how to administer gentamycin guided by the standard treatment guideline, their knowledge and skill started to evolve. We also communicate by phone when they face challenges which they call us, and the conduct of such support strengthened the HEWs to provide the services. However, the base for all these changes is the JSI L10K, which is impossible to do with the government budget since there is a limitation to

making review meetings and providing training, so it is because of the JSI support that the HEWs were able to develop the skill and acquire the knowledge to provide the services.

Interviewer: What was the specific support that was provided to you which enabled you to provide those supports? The training was one, what else?

Respondent: the project as I told you has supported us with training, and the second is it has been supporting us with supportive supervision, and in the provision of some supplies. We were also trained on mentorship by JSI which enabled us to provide similar support to the HEWs. I consider the support from JSI as the main factor for the changes obtained, the WoHO also makes supportive supervision of course but most of the support in the newborn health service is made by the JSI. Initially, we didn't have any idea or the skill on how to approach the HEWs to make the proper support but after we took the training we are able to mentor the HEWs properly and also have the skill to make the monthly and quarterly review meetings.

Interviewer: How about the logistic issue and cost of your daily expense etc., who makes those support?

Respondent: well supportive supervision is made for free.

Interviewer: I brought up something else ha?

Respondent: hahahahaha

Probe: What was changed?

Probe: Was the support you got from the project and PHC helpful? What could have been done differently?

Interviewer: was the support you got helpful? And can we say that it was one that made you meet the intended objective?

Respondent: Yes, we can say that. The project support has given us great capacity. Our profession is related to saving the lives of children and hence in this regard if the project has enabled us to get the training, helped us acquire the relevant knowledge to also support the HEWs, and if the leaders' participation also increased because of the support, what more can we ask for.

Interviewer: maybe if there are activities or supports which you say could have been done differently, that could have resulted in much better performance?

Respondent: well what I say is if there are regular review meetings at the HC level and if there is refresher training for the HEWs and also for supporters from the HC. I also say if we can improve the participation of the religious leaders, and those community figures who are influential in the community—if there is the chance we get to meet these people and discuss the different issues we will achieve a good result.

Interviewer: what kind of meetings can elaborate on the type of meeting you are referring to?

Respondent: what I was referring to as a meeting is, for instance, it could be quarterly in the presence of the HEWs, HP supporters from the HC, the WoHO, and if possible the WDAs to evaluate our performances, and if there is a stage where we can discuss about the achievements from our joint

effort. It will make it possible to everyone to take responsibility, usually, we do this when we go to the specific kebeles but that is just for that specific Kebele but if our HEWs could participate in a meeting where everyone is present, they can really get the understanding on how attention has been given by the government, the partners, and others for the activities.

108. Do you think the support system is helpful (mentorship and on-site coaching methods, supervision, technical support, PHCU level PRCMM, etc.) to enhance your skills [that is HEWs' skills] over the traditional off-site training and woreda level review meetings?

Respondent: well previously the training could be provided once at the woreda level or somewhere else and based on that, these trained people will be working from the single knowledge they got and they also don't have much monitoring and the majority of them are also HEWs who take the training. They are the responsible ones and will be implementing the activities to their best, and when they also face challenges they are not well supported unless it is for the quarter or six-month supervision they might get once every year which we also get a lot of gaps when we go there during these supervisions, for instance, you might get gaps related to drug their administration like gaps in the dose, duration of the administration, or they might miss some of the danger signs and manifest gaps in the disease classification. Currently, however, the HEWs have taken a refresher training and all new and existing HEWs are all trained but had it been in the previous time, they might tell them to continue and the training to come later, but now everyone is trained and the supporters from the HC are also trained on how to provide mentorship to the HEWs, so adequate knowledge has been obtained for the HEWs from the training and is well introduced with chart booklet at the HPs; for instance the chart booklet at the HC is IMNCI and the HEWs' is iCCM and since the iCCM is written in the Amharic language, the supporters used to make improper support since they were not well acquainted with the iCCM booklet in the previous time but since they are now well trained on the iCCM chart booklet, they are making better support to the HEWs. Hence, they will be on the same page when the HC supporters go to support the HEWs, and they observe every case based on the chart booklet. Previously the HEWs had challenges in making correct disease classification; prescribing correct drug dose and duration; analyzing and verifying the danger signs and the cases. Of course, the chart booklet is written in Amharic and clear, but they had some challenges in wrongly interpreting it and lack of focus to investigate more and clarify the cases, especially they had difficulty regarding the provision of care on Sepsis cases which they were challenged to administer specifically Gentamycin. They, of course, have been administering vaccines but when it comes to iCCM service and to administering Gentamycin they manifest fear related to some skill gaps they had. In relation to this, the HEWs when they identify Sepsis cases, they don't register them but directly refer them to us. However, when we demonstrate to them how to administer Gentamycin using artificial dolls in the skill lab that JSI built us, they have shown an improvement and it became

easy for them gradually. The quality has also improved when we evaluate the performance but previously there was a repetition of the same gaps since the frequency of our visits was very long.

Interviewer: If you can explain what you mean by quality as a testimony of the improvement or change before and after the intervention?

Respondent: for instance, what I mean by Quality is the drug administration time interval as stipulated in the standard treatment booklet, and the other is the correctness of the assessment and the classification—whether the two match or not. And, hence the current administration of drugs provided in a way that does not inflict any harm or safely to the patients and also aligned with the standard time interval, I think has quality. Previously, there was misclassification of the diseases, and gaps in identifying the main health complaints associated with the disease that is missing to detection were highly observed in the previous times. And, we think there are improvements regarding these gaps. Previously, however, challenges existed regarding pneumonia and diarrheal case investigation mostly, for instance, regarding heartbeat determination it is stipulated to calculate within 1 minute but they might just do it for the first 15 seconds, etc., And, also there are gaps in focusing and being preoccupied on other major disease types unrelated to the disease type at hand, for instance, missing to detect difficulty of newborns breastfeeding being preoccupied and inclining to their own imagination of the disease, and missing to detect the correct disease in the patients. Such gaps were prevailing in the previous time but it has been fixed and improved.

Probe: for acceptability of the strategies **(for HEWs only)**. How could the support system be improved?

Respondent: the HEWs are very happy and they have a good acceptance of the type of support we provided them, and most of them are receptive and happy about the challenges we identify and try to solve. Most of them also recommend that we make a more frequent visit, and some of them also call us when they encounter any challenges related to understanding the chart booklet, and we provide them virtual support on what to do. Their request is for us not to interrupt the regular monthly visit, but in the meantime, they also call us for support. We have even made ad hoc supports through motorcycles and other means.

Interviewer: Ok if you have any suggestion on how you could have also provided better support than this? something different from so far?

Respondent: well regarding our support, there are times we skip the regular visit that we planned and agreed to do at the HPs on a monthly basis which sometimes may be interrupted due to different competing priorities, and this has to be strengthened in the future. The other is also regarding the practice which the HEW make here at the HC, we have previously shown them on how to administer Gentamycin as I told you earlier, but these things cannot be complete and there are issues that need this type of support, and so if there is the condition for the HEWs to come, and refresh their skills and fill the gaps.

Interviewer: What is that holding you back from doing it yourself? What are your challenges?

Respondent: well it is just the time constraint. The HEWs are usually overburdened with a load of tasks. We have actually had the idea to invite them here and make them refresh on the whole component of the chart booklet, it is possible to do at the HC and no problem since we have also trained health workers and the complete package here with us. It is one due to the time constraint of the HEWs and it is also because we have not really pushed for it relying on the support we are providing them onsite at the health posts, so I consider this as a gap from our side as well.

Interviewer: Is there any other suggestion that you think could have been done differently and improved just like this one? Regarding the support system.

Respondent: the support we are getting or made from the WoHO is also not that good. There is an assigned expert on maternal and child health at the Woreda health office but the support is not that good and is not that frequent. The HEWs are also not receptive to the type of support since it assumes a kind of check and balance, and controlling. So I suggest that the support from the WoHO is also well strengthened. The Woreda also makes support at the HC level at times when there are challenges with supply or budget allocation. However, it lacks technical strength and is not a kind that builds technical or professional capacity. For instance, there is a kind of specific support in the area of service I am working, they just trust me that I can do it and there is no more than this from the district office; they don't see in detail about the IMNCI service that is available at the HC and evaluate whether I am performing well or not.

Interviewer: How about things that should have been done differently from the project side?

Respondent: regarding the project, what I say is if it can continue providing us with regular support. There is both the iCCM and IMNCI service, and for instance on IMNCI at the HC if they can observe in detail our performances and provide us support according to the identified gaps. Of course, we are mostly supported through training, but if this is not limited to that and support us also in person through checklist-based onsite support. The frequency at some time might become intensive and at another time it might become low, but the intensity makes the HEWs and everyone motivated and to engage in the activities. So I recommend that the support is regular and not interrupted by sticking with the set schedule. In addition to this, it is also good if the regular supportive supervision and monitoring we are making to the HPs are maintained as regularly as possible. I think the HEWs have potential if they are well strengthened and it will also improve the chance to get the community's trust. If the other issues I mentioned like the review meeting and others are also strengthened, I think we can get better achievements.

Interviewer: you mentioned about the project support to be based on a checklist, is it not how it is done so far?

Respondent: No, the support is made through a checklist of course, but my point was on the regularity or frequency of visits which is not regular. So I am suggesting if it is improved to be regula.

Interviewer:

109. How eCHIS implementation helps you with iCCM service delivery?

Probe for advantages:

- case identification? quality of iCCM case management? retrieval of client records? data quality? client appointment scheduling and/or defaulter tracing?
- **Ask why?**

Respondent: ok regarding the eCHIS implementation I don't have much understating.

Interviewer: Why don't you have the understanding about it?

Respondent: well I have not taken the training on eCHIS which I may have some highlight but O don't know well about which services are integrated in the eCHIS. However, generally, I know that maternal health service and other services are integrated.

Interviewer: is the training provided at the HC level and how it is cascaded to the HPs?

Respondent: well I know the training was provided at the HEW level and I know also the midwifery professionals have taken it but those working on other services including us here working in under 5 there is no one who has taken the training. That is why I was not sure about the integrated services in the eCHIS.

Interviewer: Ok you can tell me to the level you know about it?

Respondent: Ok what I know is that they are using for recording the households and family members, maternal health service is completely provided through that, and the services by the WDAs and 1-5 network is also made through the eCHIS. The services provided in the eCHIS can also be made manually as well as electronically through the system, for instance, I have seen it used more for the maternal health service, for instance, it provides them swift information about a mother who delivered at the facility and that the mother should get PNC service which enables the HEWs to react quickly to provide the service. In this regard, the previous linkage and communication between the HC and the HEWs was not strong, for instance, when the HC sends the mother they might give her some paper and in the meantime the paper might be lost and the follow up might not happen. Currently however, the eCHIS has strengthened the linkage between the HC and the HPs, and since it also provides her information and put it in the action box of those unimplemented activities for the HEWs, it creates the urgency among the HEWs to go and execute the task and I see that it helped them for these matters. Previously, if it is a PNC there was a card that was sent along from the HC to the HP which might also be lost in the meantime but now the card sending has stopped and it is no more available at the HP.

Interviewer: what has the abolishing of the card utilization and replacing it with the eCHIS has contributed?

Respondent: well the cards are given to the clients and they might not give it to the intended HEWs, and the message might not be delivered. But in case of the eCHIS, it immediately sends notification to the HEWs as soon as the mother's delivers, which the system sync the information, and the HEW at the

HP can see who is in line to receive the PNC service, and it will make it clear for her who she should be providing the service. So she makes prioritization and provides the service accordingly.

Interviewer: do you think it has significance for the case identification?

Respondent: I think there is a chance to do that since I think it is also available in the maternal health service module to assess about newborns who developed illness during their house to house visit. So I think it would help with that since every information about that specific house hold is also available in our hands, I think it will help.

Interviewer: How about the eCHIS contribution in scheduling and defaulter tracing etc?

Respondent: it helps with that. For instance, when there are defaulter and such specially when we treat cases of uncomplicated malnutrition at the HC level, there might be defaulting and not showing up on the date of schedule, so since we have that in hand and we are easily notified, I think it can help us trace the defaulter and to ask the cause for that.

Interviewer: How about the iCCM service quality?

Respondent: yes, it helps with that as well since we check every part of the questionnaire and avoiding the general enquiry at once we are usually accustomed, but making us tune and focus on details of the questions, so I think it helps to prioritize and provide the service accordingly.

Interviewer: How about the data recording? It is not possible to get every information at household level or the client information at kebele level?

Respondent: in the eCHIS, yes. I think the HEW will have information recorded about each household including when there are new births.

- **Probe for areas of strengthening?**

Interviewer: I am reserving myself on the following point since you mentioned you don't know much about the eCHIS, but I was going to ask you on how it should be strengthened, and since you are also the iCCM focal, issues that you think could have helped you if integrated in the system, if there is any though you have related to this?

Respondent: No, I don't have any since I have not got the training.

110. How was the introduction of the iCCM/CBNC module into eCHIS affected the iCCM/CBNC service delivery?
FOR LUME WOREDA ONLY extra

XXVI. Implementation challenges

111. Describe the main issues faced by the health system to identify and treat neonatal infections in the community. What are the critical factors affecting the delivery of iCCM to clients?
Probe:
- lack of competence of HEWs, shortage of supplies and commodities; weak support system; low community demand?

Respondent: generally the challenge on the health system is that there are different perceptions in the community that should be avoided and the activities should be strengthened to improve the practices among the community to visit the health care facilities more than the cultural and traditional medicines practices most community are inclined to such as the challenges in avoiding medical care for newborns before they are first exposed to a holly water and their visitations to seek traditional and cultural medicines etc. there must be a means whereby the community gets uninterrupted awareness, and if the health system works intensively on provision of health education to the community, I think we might see improvements. The other issue is related to the medicate care provision specially related to the HEWs gaps if there is a coordinated effort to solve these gaps. Also, regarding the mentorship program which should be done strengthened in a manner it is evaluated regularly to identify the gaps and assess whether the services are provided keeping the quality.

Interviewer: This activity you mentioned on the mentorship is expected from you right?

Respondent: yes

Interviewer: So what are you recommending about it to be strengthened?

Respondent: in order to strengthen it, there must be a regular evaluation of the activity at HC level.

Interviewer: are you not doing that?

Respondent: Yes of course, we are doing that, it is just that I am recommending generally on all.

Interviewer: what I want to tell me is about the challenges which are regarded as major challenges in the health system which is hindering the iCCM service delivery?

Respondent: it is right, so the first is the community perception. This is one, the second is the problem with regular refresher training provisions for the professionals. The other is elated with the supply shortage which we sometimes are mostly challenged with stock out of gentamycin, and also Amoxicillin and Zinc which these supplies sometimes get missing for a long time. Who provides us with these supplies to us the PFSA but mostly these things are a challenge, if the supplies are supplied to use regularly and timely, I think it will be good to provide the uninterrupted services.

Interviewer: How about the situation with doing what is expected of you etc? any challenge?

Respondent: we usually receive the reports from the HP and we want them to show the gaps., and based on that we receive the supplies directly from PFSA and most of the time since the iCCM supplies do not come through program support they are usually availed by buying them from the market, the service, of course, is exempted and provided for free to the community. Usually, gentamycin is not availed through program support so we buy and avail it for the service, but when we request PFSA there are many occasions that they tell us it is stock out, especially the 20 mg used for the Sepsis care; of course they tell us it is also a problem at national level but there are many occasions we don't get them. Hence we try to purchase from private suppliers etc, but it is a challenge at country level. The supportive supervision should also go in line with a known schedule and in uninterrupted

manner that is including higher level structure which is not clear for them, for instance, there is no clear guidance on the frequency of support that WoHO should make to HCs unlike ours, which is clearly stipulated in the policy about the frequency of visit that we should make including what supports to make to the HPs. We can't ask them why they didn't come here because of this. The last point is about a motivation mechanism and recognition issue. Regarding this issue there are integrated services that are provided at the HP level but we don't have any means of evaluating the performances to rank the performance among the HPs. How we are measuring currently is using the number of care they provided and others and it is us who are also setting up the weight by allocating some scores for specific activities but it has to have a uniform scheme that should be used throughout to evaluate all the HPs; for instance, when the HCs are evaluated they have their own measuring criteria like KPI and others but there is no such thing used to evaluate the performance of the HEWs. For instance, if we want to reward a good performance on iCCM we have to have some standard for measuring their performance since we might end up demotivating them in the process. The standard is also good not only for recognition also to identify poor performances and provide advice and support as well. As a practice we have budget limitation as a health center and hence we only provide them some certificates but that may not be enough and if it is coupled with better recognition means which will motivate others as well. This recognition could be made at the woreda or at zonal level. The criteria we are using is only ours and is not standard and some even may ask why but this is just our criteria we used to rank them as first, second etc, and hence we are asking for a uniform measuring criteria that can be used for the entirety.

Interviewer: if among the points that we raised, if you can point out one which is the most critical one, which one would be?

Respondent: well, it is going to be the gap with the community perception which I consider as a major constraint since others have been addressed through training and others.

Interviewer: what if you are asked to mention the second critical challenge?

Respondent: if to some extent there is a refresher training since all have taken the basic training, which is a continuous one. The next will be mentoring and then comes the measuring criteria and if the recognition and reward mechanism is in place.

Probe:

- Regional/national state of emergency and conflict in the northern part of the country?
- How has that changed during COVID?

Respondent: well the emergency of state did not have much influence to deliver the services unless it is to some associated risk and fear to come at night to get the services from the community side associated with loose law order and enforcement. But concerning us, there is no much influence it caused us. Similarly, with the conflict, it may have taken away the attention of the community but there was no

direct influence to deliver the health care services that they still were coming and getting the medical care. May be it is because the war didn't take place in our area and it was only those security forces that went to the war front, so there was no special influence it inflicted on us. Also, regarding the COVID impact, it was seen at the beginning when it occurred first time in our country that the community has also refrained from coming to the HFs but later, it has shown an improvement, also from the awareness creation about the services availability and to use the services while taking the proper precaution measures without any fear that we tried to explain to the community. We also tried to educate the community in streets coordinated by the WoHO and also taking turns in our catchment at the Kebeles, we tried to provide them awareness about the pandemic. There was a different kind of fear that surged here in the community from reports happening in the town but gradually things start to improve and we tried to fix. Regarding the conflict effect, it was only with the security force and it didn't make any effect on the service delivery. The state of emergency was a challenge may be for services provided at night for security related fear by the community to come to the HFs. The HEWs also used to close the HPs and get away, but from the discussion we had with them it was immediately solved, and this didn't last long also and only for a brief time this was encountered.

112. How do you think the COVID-19 pandemic and/or COVID-19 response measures affect the uptake and/or delivery of iCCM services?

Probe:

- Has COVID-19 affected your daily routines; your work on newborns; the community in terms of livelihood and vulnerability for newborn care-seeking
- How has that changed over time?

Interviewer: I am still going to take you back to COVID again, may be from the community side. What was the challenge specially you faced to provide the iCCM services?

Respondent: yes, there was some perceptions and thinking from the community side the HEWs might expose their children to the disease and describing the HEWs mostly having the disease since it is thought they stay with patients. So there were those thoughts from the community, and because of this fear they were not willing to come to HFs and stayed in their homes but we were immediately able to create awareness in the community, come to a consensus with them and were able to immediately restore things to normal. Concerning the current situation, well the COVID pandemic is still there, and unless for some negligence in the community, we have already communicated with the existing social structure and kebele leaders about the service availability despite the pandemic, to avoid the previous thoughts about the COVID pandemic and to come to get the services—to be communicated with the community, and as a result they are currently coming to the HPs and using the services.

Interviewer: was there any strategy which the project implemented considering the COVID pandemic, for the iCCM services not to interrupt?

Respondent: well the direction we received was to apply the precaution measures and to provide the services accordingly. So we have been implementing that, and the interest from our WoHO was also to apply the precaution measures while we conduct every activity such as during vital sign takings, while we use thermometers etc. there was also adequate PPE materials including face masks, also sanitizes fulfilled for the professionals.

Interviewer: How did it also tested the community that is from the requests asked of them to fulfill like the mandatory mask use etc?

Respondent: from the community perspective it was not that much requested of them to fulfill since they have been using from the HC resources like the sanitizers. But since the community is one residing in the rural area, there was no good awareness about the prevention measures and it was after we provided the vaccine that there was a bit awakening. Otherwise, it was only those visiting the HCs that were forced to wear related with the No-Mask-No-Service motto at the time, but they were not wearing any face masks when they visit the HPs for the iCCM and other services.

Interviewer: Any challenge in affording the cost and similar problems you observed from the community that may have hindered the service delivery?

Respondent: it is just the lack of awareness that the disease is not going to caught them, and not related to affording the cost since there were options and available resource availed in the market to also use cloth-type face masks. But there were challenges in properly using these PPEs even though there were efforts from the HEWs side, there was a significant gap form the community to adhere to the prevention measures.

Interviewer: Ok, despite the gaps in proper utilization of the prevention measures from the community, what was there specially hindering the iCCM service delivery during the COVID era? It could be from the client or the professionals side.

Respondent: we have given due attention for the newborn screening since there was the prevailing though that COVID was highly spreading, and the screening for the newborns were critical and have given greater attention in distinguishing between COVID, Pneumonia and other diseases since we have given the HEWs training about the screening. Since they were engaged in the community COVID screening for adults in their hose to house visits, they have also made similar attention for the newborn COVID screening as well. However, at the begging of the academic all services including maternal and newborn health services were stopped since they were not coming for fear of the pandemic. And, the professionals including the HEWs were also challenged to fully engage in the activities for similar reason but later from the awareness creation and utilization of the prevention measures, the services start to improve and the community also start showing for the services.

Interviewer: Well you know diseases among children are usually acute, and was there any trend you observed in increment of sick children as a result of their lack of visitation?

Respondent: well at the beginning when it was highly rumored about the risks associated with the pandemic, there was a tendency of keeping their children for risk of exposure in their homes, but as the cases become severe, for instance, children who were diarrheic used to come at night or at times they think there is no one in the health center. But later after the vaccination and we provided them the awareness that we also provide the services along with other services, the community starts to ease up and gradually began to use the services.

XXVII. Adoption and reach/effectiveness

113. Explain to us any changes you observed as the result of the implementation of the COVID-19 adaptive iCCM implementation strategies? – What impact have the strategies had?

Probe:

- for a support system and linkages, motivation, and competence of HEWs, community*,* and awareness creation?
- What do you think are the reasons for non-significant changes?

Respondent: whether it is before or after the pandemic it is known that the impact regarding newborn health was prominent especially when we see the under 1 child in general, there is a significant death. And, when the pandemic is coupled it makes the situation much complicated which is obvious to imagine since the death toll is going to be exacerbated due to the pandemic coupling effect. So what the project approached to implement considering the influence from the pandemic to deliver the iCCM services was, one in maintaining the existing service delivery—keeping the quality and not to be affected due to the pandemic through the provision of consequent training and supportive supervision which I think the project has been supporting, and the other is with the regard to supporting the HEWs to adopt different services delivery approaches, and also supporting them with different supplies.

Interviewer: What service delivery approach for the HEWs?

Respondent: for instance, to use sanitizers, put on face masks and to keep their physical distances in order to protect themselves and the community while they are delivering the services. This was delivered to the HEWs by the project through improved awareness and providing education, and also availing the different medical supplies required to provide the services without interruption and helping the quality not to be compromised. So the project had a contribution in helping us withstand both the possible interruption of the services due to supply shortage and also in preventing the COVID pandemic, and as a result of this, it enabled us to quickly adapt with the situation and provide the care with an improved quality for a lot of newborns. Other than this, it also helped the community to gain awareness through the HEWs by using different banners and leaflets. Messages were also communicated to the general public on the prevention measures using megaphones, and the

messages were also communicated in a way that the services do not mean they are going to be interrupted because of the occurrence of the COVID pandemic.

Interviewer: Ok now you have provided the awareness creation and others but was there any challenge related to transportation and others at the time to come to the HFs caused due to COVID?

Respondent: As I told you earlier, the HCs at the early stage of the pandemic were closed, and clients coming long distances incur costs for nothing not getting the services at the HCs so what we were communicated for the community specially for the newborn health services was that not to come to the HCs since they can get the services at their nearest HPs. So we see mothers at the HC who have come from long distance we provide them awareness, and also at the HPs since we already made agreement with the HEWs from our meeting to provide awareness about the services availability at the HPs. Yes, it was true clients used to come from long distances also because the services at the HPs were also smoothly provided but later from our training to the HEWs and the awareness creation, conditions have improved gradually and the services for newborn health care also started to be fully provided at the HPs, and the community also gradually made the improvement to use the services at the HPs, and in this regard there has been much improvement.

Interviewer: What about the significant of the support with regard to improving your linkage with the HEWs?

Respondent: as I told earlier we have already the guidance about the support including the frequency of support to the HPs even if we don’t always keep the schedule. However, in relation to the iCCM service, the provision of the training especially has enabled us to make regular monthly support to the HPs, that is also separately for the iCCM service alone without coupling with any other task which has helped to identify gaps related to this service only. It also enabled the HEWs and us to work more closely and in coordinated manner, and also the WoHO to support the activities which enabled the improvement of the performances as a result of the project support. Previously however, there was no serious evaluation of the performance and the gaps used to repeat since there was not specific support like the project’s, and we were passing through one gap to another without solving any gap prior.

Interviewer: which support type was the most significant of all which enabled you achieve these results you are describing?

Respondent: it is mostly the mentoring and the integrated supervision. The mentoring has realized solving of the skill related challenges at ease, and the supervision especially has enabled us solve the supply and operational challenges. The review meeting also helped us with the awareness related challenges in the community and among the HEWs, also in solving the challenges related to the service delivery and performance issues among the HEWs. And, all these things were facilitated for us by the JSI L10K.

Interviewer: what about in improving the motivation of the HEWs and also the competency?

Respondent: it has improved it a great deal, and as I told the regarding the competency well our service frankly speaking had no quality, especially the service for under 2 months was filled with fear, for instance, there was no any conviction to administer gentamycin and was filled with dread. We were doing providing the services to the community with full confidence and quality, but after the project arrival training was provided to us and that has helped to improve our skill and knowledge to adequately provide the services. The projects support to make strengthened follow up and supervision to the HPs has built our capacity and skill. The consequence of the strengthened support also improved our HEWs motivation to exert more and develop the interest to save more lives.

- What are the particular features of the strategies that made a difference?

Interviewer: what is the most impactful component of the project strategy that made a difference in your opinion?

Respondent: as for me, it is the support that is provided at the HEW level to improve their skill [PCMM], and if you ask me for my second, it is the mentoring support that helped to maintain the quality of service and its continuity.

XXVIII. Maintenance and sustainability

114. What are the high-level benefits that are attributable to this support/IR?

Respondent: it is the increase in the quality of care and also the reduction of neonatal death especially those under 2 months and us being able to easily save children, I think I would be happy if it is attributed to the support or to the project.

115. Please explain to us the feasibility of this support/ IR for national scale-up?

Probe:

- What features could easily be integrated into the existing system? Which not?

Respondent: regarding my opining on the scalability, I think it is possible to scale up to others and if so, it can also easily be integrated in the existing health system. Well the HEWs are available in all health system since there is already the chance to solve the awareness related gaps among the HEWs and supply related challenges since the system is also already established in order to avail supplies and others at the HP level. We have a structure now with availability of health professions who are capacitated to support and provide mentorship in every HCs, and if we can further support them through training and others things, it suffices. There is also an existing established system regarding the HC-HP linkage and policy, and if we can also strengthen I think we can get better achievement. In addition to this, there is also the social structures which are available everywhere, and if we can support them I think we can achieve much more with less effort.

Interviewer: Ok, what about focusing on the support systems that were introduced after the introduction of the project, and which do you think among those activities would be sustained?

Respondent: I think the iCCM service that is being delivered at the HP level will continue but if the project support may cease, I think the activities that are being implemented around the WDAs may stop too, this is

because so far it has been working on supporting the review of performances at the community level so may be those may cease if the project is no more exist. Other than this, the clinical mentoring might also discontinue if the WoHO could not sustain and make regular support, and if the HC and WoHO don't work in coordination and sustain this, it might not be maintained. May be the iCCM service at the HP level, it is being provided as a routine service since the project has already introduced us well to the activities, that is if we can still support it with supply, and fulfil other requirements.

Interviewer: Why do you think is that the clinical mentoring might not continue?

Respondent: it is related with the lack of follow up which I presume might not be made after the project phases out. This is my observation from experience; it is the usual practice that when a project that introduced some activities leaves, there is less tendency of the health system to build on that and sustain it. There may also be turn over and others, which might force the activities to pause.

Interviewer: What is the status of capacity building and training provided in your cluster?

Respondent: as a Dembecha cluster, we have adequate man power who can resume the services both from the professionals and also the HEWs who are adequately trained. And, all might not leave at the same time but the threat is if they are not replaced when they do leave.

116. How are the activities/efforts embedded in the PHC and woreda routines/work streams?

- What implementation strategies are incorporated with the PHC and woreda annual work plan?

Respondent: for instance, we have plan for the activities and a follow up and checking mechanism for the activity performances. We also exchange report every week, and we ensure the activity implementation by availing the required supplies based on the number of care they provided for the newborns. We also interview clients and check the provision of services for newborn health care services during our support, and hence these are ways we ensure the continuity and that is also integrated in our routine service.

Interviewer: what activities are integrated in your plan?

Respondent: on the services provided for instance, there is health care service for Tuberculosis, Diarrhea, uncomplicated malnutrition, Sepsis, local bacterial infection which are divided among the HPs as plans. So one HP knows its own plan in the number of Pneumonia or other diseases per month, per quarter or per year. If for instance, a HEW does not have a report of Sepsis care per month we ask why during our review meeting. There are also other activities related to iCCM service such as number of WDAs they provided awareness creation which are not integrated in their plan. Regarding the supply, it is incorporated in the main HC plan and not divided among the HPs.

Interviewer: Are the HEWs well evaluated and is it also part of their career evaluation?

Respondent: yes, for instance, the HEWs discuss about their performance and what they performed prior to reporting at the HP. What we are now mainly focusing as an institution is on Sepsis health care

provision which we have been given a major task to achieve. So they [the HEWs] are also focusing on Sepsis care and evaluating themselves, and we also use this performance to evaluate them and to measure their performances. So it is part of our activities—the iCCM service and we also much attention to the under 5 service. The WoHO has given us a plan to be cascaded down to the HP level, and the iCCM performance is also presented during our quarterly and annual review meeting through the HPs. The WoHO also provides support once every quarter for the HCs, and the iCCM is considered as one part of the child health care service. It is also integrated during the woreda based plan, and we also allocated supplies required to provide the iCCM service which is purchased along with medical supplies.

Interviewer: what about the integration of the iCCM service in the different support system, in the regular support system even in the absence of an iCCM focal, the tendency to be supported in the system of the HC?

Respondent: it is also available in this as well. For instance, the iCCM service is integrated in the integrated supportive supervision checklist along with the other health services. May be if someone who is not trained on iCCM is not available it might be missed so we make sure someone trained on iCCM is available within the team which is a must, so as to enable the detail observation of the iCCM section at the HPs. May be what is to take away from our support is that we have some interruption in making all the planned regular quarterly supervision due to workover load here at the HC. But when we make the supervision there are detail aspects that we observe on the iCCM service such as the number of care provided, classification proper conduct through register observation, plan versus achievement, and also a section where we raise points on the supply issue including the quality. So it is part of our regular activity.

Interviewer: what is that you identify as something as a good achievement or best practice and something that could also be scaled up at national level?

Respondent: may be the mentoring, and the regular support we provide to the HEWs. Also, the integrated supportive supervision that is well supported by a checklist, and the support we provide to the HEWs in terms of capacitating their skill such as those training we provided on the injection etc, and also in the awareness creation on some community perceptions. I think these are areas which I think should be scaled up to other areas.

Interviewer: if may be we single out the mentoring, what is it that makes it different to be regarded as something scalable, different from others?

Respondent: regarding the mentorship is enabled to identify and fill the skill they have, which I think makes it different in my opinion.

117. Do you have anything think is important to tell us that we have not asked you?

Respondent: I would recommend is that if the training for the HEWs is strengthened since science is dynamic, and if there is a continuous update for the HEWs aligned with the emerging facts from recent studies. If

there are also regular review meetings and interventions concerning the perceptions emanating from the community that is based on a strategy from a thorough investigation.

Interviewer: Thank you very much for your time, I have finished all my questions. If may be Eyerus has something to add or ask. If no, thank you very much again.

THE END

End line Evaluation of the PSBI implementation research; Full Transcription for an In-Depth Interview with iCCM Focal Person

Questionnaire ID	11
Area Identification	Dembecha
Name of Woreda/Zone/Region	Dembecha WoHO/West Gojjam Zone/Amhara Region
Name of facility	Health Center
Name of moderator	
Name of a note taker	
Date of Interview	15/06/2022
Participant #	01
Audio File #	HC_Head_15.6.2022
Start time:	09:00 AM
End time:	10:53 AM
Transcriber/Translator	
Duration of IDI:	1 hr, 53 minutes

The interviewer explained to the interviewee in detail the purpose of the research

Q. Can you please describe the demand generation/SBCC activities you are doing on iCCM/ newborn health? What are the strategies used to raise awareness? If none, please explain why.

A. I thank you so much for having me for the interview. First what we did was to organize training program for Health Extension Workers/ HEWs/ and ICCM focal person to boost their capacity.

Your organization has supported the training program financially. I am not able to oversee the usual activity of HEWs because I am completely absorbed in performing my usual activity.

In other words, I have excessive workload. Therefore, we managed to give training to an experienced and qualified person to give assistance to HEWs using a checklist while they carry out the tasks done or occurring every day. Accordingly, the expert has to oversee, manage and direct the implementation of ICCM by HEWs.

At the present time most of the health workers take part in a campaign to achieve a specific goal. Likewise, the campaign is having a strong effect on other activities.

But, the expert provides four health posts with essential support in compliance with the fixed standard /checklist. Only one health post did not get support. However, most of the people did not know whether or not CBNC was offered by the health posts.

As a result, a lot of people used to come to the health center to get the service. However, we were questioning them about their reason for coming to the health center.

Afterward, well-informed members of the community started to get the service at the health post. We discussed the problem with the woreda health office and the health center to send a message to the administrators of the respective settlements.

Thus, we let them know about the provision of CBNC in all health posts by means of the letter. We told them that we have done what is necessary to carry out our task.

We informed them that no an insufficient supply of inputs and shortage of manpower to offer CBNC. We made effective use of the available opportunities including religious service inside a church to pass on information to the community.

We campaigned to make the community familiar with the CBNC provided by the health posts. We carried out the task along with the HEWS. Afterward, members of the community have stopped coming to the health center to get the service.

The people were weak with mental and physical exhaustion after travelling long distance to the health center. We are feeling pleasure to see the problem of the community solved. The expert has been exerting strenuous effort to look after the activity.

However, the community is still coming to the health center to get ICCM service. HEWs May not it find easy to deliver ICCM service.

Q. Why do you say it is difficult for the health posts to deliver ICCM service?

A. Because inputs are in short supply to the health posts. Usually there is scarcity of zinc and amoxicillin. But our need for amoxicillin has been partially satisfied.

Therefore, the community is / was coming to the health center because of scarcity of essential elements such as amoxicillin and zinc in the health post.

Q. What else have you done to increase the level of awareness of the community?

A. HEWs have performed a lot of activities worth stating. They have been organizing women's conference at a kebele level. Administrators of the respective neighborhood are also in attendance of the conference.

HEWs use the occasion to disseminate information to the people who take part in the meeting. We used to support HEWs organize the conference. Every morning we offer an advice to people who receive medical treatment to obtain CBNC at the health posts.

To our delight, everything is turning out well or having the intended result. But, we are facing some minor problems at a particular time/ when we take part in a campaign or inputs are in short supply/.

Q. Have you ever used a banner to advertise or publicly announce availability of CBNC?

A. We have not used a banner so far. We have severe budget constraints to buy a banner and advertise our service. To our dismay and to the dismay of others, we have no money to buy sheets of paper. But, we exert the utmost effort to meet the needs of the community.

Q. How is the engagement of communities/WDA networks in the implementation of integrated community-based case management (iCCM) of common childhood illnesses/possible serious bacterial infection (PSBI)?

A. community engagement in the implementation of the project is encouraging. It gives us hope and confidence. Coming to the health center involves a lot of effort, money and time.

But, going to the health center involves very little time and effort. Besides, they receive the service for free. Consequently, the community is now feeling pleasure.

The health center charges 7-8 birr to be issued a card. But every kind of service is given free of charge in the health post. Currently the community is completely absorbed in thinking about the deteriorating security situation of our country.

However, the community is very much engaged in the execution of the program despite a feeling of frustration.

The community is willing to get treated its child. The drivers charge as much as twenty birr for a single trip from Solomon kebele to Dembecha town / the health center. This is an exorbitant price or unreasonably high.

Honestly speaking there are no Women's Development Groups/ WDGs/ in our neighborhood. They aren't functional at all.

Therefore, we have put a different structure in place. The recently formed structure is called village health leaders groups.

Q. why did you establish village health leaders group?

A. It is because the officials at the woreda level did not pay adequate attention to the well-being of the community. We have assumed that we can find at least one person who will be willing to join village health leaders team.

We are planning to give training to every potential member of village health leaders group. The law says that three people will be selected from one neighborhood /district to join village health leaders team.

The would-be member of village health leaders' team must be able to read and write.

After we train prospective members of the group, they will be assigned to teach the community about treatment of neonatal infection, nutrition, Maternal and Child Health/MCH/ together with HEWs.

As we know HEWs are usually fully occupied in dealing with an illness or a disease that affects the good health of the public at large.

Q. Are village health leaders going to take the place of WDGs?

A. they will replace WDGs to a degree but not completely. Village health leaders are rather additional to WDGs.

However, WDGs are more desirable at a national level than some other groups. But, WDGs are having failed to be cooperative and enthusiastic.

Q. Have village health leader groups started functioning?

A. We have already chosen members of the groups from among the community. Likewise, our next job is to give them training at a woreda level and allocate a task to everybody.

But, the warfare between government and rebel forces within and without the region as well as coronavirus pandemic made progress difficult. I hope we will achieve a desired result if they are given training and job assigned to them without delay.

Q. What is the work or planning involved in making the training ready? Please describe your state of readiness to make the training real?

A. There is a training manual that was prepared at a woreda level. We expect them to organize the training program as soon as possible.

Three people have been selected from individual kebeles to offer the training after which we made them known by the woreda heath office and the health center.

We are expecting the woreda health office to give us inputs to offer the training to the would-be village health leaders. What is expected of us is to choose prospective village health leaders from among several and make them known by woreda health office.

Q. Are their duties and responsibilities clearly defined? Is there any job description for the would-be village health leaders?

A. There are two kinds of manuals or books giving instructions. The manual states that every village health leader will submit a report every week about the number of pregnant women, new born child, vaccination/ inoculation etc.

Every village health leader and the HEW will come together, write and submit the report to the woreda health office and the health center once each week.

Q. what is the distinction between the roles and responsibilities of WDGs and those of village health leaders?

A. There are different types of WDGs that are involved in supporting the agricultural sector, health sector, education sector etc.

Q. What is the difference between the roles and responsibilities of Health Development Groups/ HDGs/ and those of WDGs?

A. Their difference is marginal / negligible.

Q. please describes major challenges you have encountered to communicate information to the community and boost their awareness? Do you have any uncompleted activity owing to different reasons?

A. a number of activities remain uncompleted when we take part in a campaign to treat trachoma, and prevent the spread of COVId-19. The health posts will be completely closed because of the campaign. At that juncture/ point, clients using the service will go back home without getting medical care or advise.

After that the community will provide the absence of HEWs from duty as a reason not to go to the health post again.

Every activity having limited duration will take attention away from us by appearing more important. The other challenge has to do with lack or scarcity of inputs. On one occasion it was difficult to find gentamicin in all shops/ pharmacies selling and dispensing medicine. There was zinc shortage for about three or four months. Our community does not trust you unless you produce tangible evidence to support your claim. But, we need adequate budget to produce evidence that is able to be perceived through the senses.

Q. How did budget constraints prevent you to make your service known by the community?

A. if we have budget we will a public sign will give information to the community. We need budget to give incentive to WDGs to motivate them to take a course of action or continue doing their work. At an earlier period, we used to pay incentives not only to members of WDGs but also kebele administrators. But it is no longer the case. Nobody is now willing to assist you without any incentive or something that motivates him to do a job.

Q. who used to pay incentive to WDGs and the woreda administrators?

A. The woreda health office used to pay them a periderm. In past members of WDGs were summoned three time a month to debate an issue or to do a job. But, the majority of members of the kebele leadership are still willing to take part in a campaign to achieve a desired goal. They are ready to give their daily payment or allowance to members of WDGs. But the law prevents them from giving their per diem to other people. The law at a federal level also says that WDGs should provide service to the community for free / on voluntary basis or without financial reward. However, last time the Carter Foundation ordered us to provide WDGs with financial reward in return for what they have done. But, the regional government does not allow us to effect the payment. This condition is creating unfavorable results. I think the government should consider of revising the rule that forbids paying financial reward to WDGs. We are experiencing a rising inflation. Members of WDGs deserve incentive because they are unable to meet the rising cost of living.

Q. how strong and effective was the assistance given to the health post by the health center and the organization that has supported the project?

A. The health posts have made effective use of the support given by the health center and that of the organization that provided money to help fund the project. I think the organization should keep on giving assistance to the health posts. What makes you amazed is that previously we were using a guideline which was out-of-date or no longer current. But recently we were given updated version of a guideline in a soft copy to deliver ICCM service.

Q. please describe in what the organization has given you an assistance?

A. The organization must be convinced of our working effectiveness in order to get all forms of assistances from the organization that supports the project. Likewise, we have to produce tangible evidence to support our claim. We are required to diagnose or identify illness in a patient and give him/ her medical care. it wants to see a complete performance. That was how the benefactor has provided us with technical / practical assistance. We must find a way of expressing our gratitude to the organization for all it did. But, I don't think the support offered by the health center to the health post will have a satisfactory outcome for the reason that we are frequently fully occupied in other activity. As I have mentioned to you earlier, we have prepared a checklist after the guy arrived here. We have assigned the guy / fellow and his aide to go to a village and do what was ordered and planned. Therefore, intervention by other activities has made our progress difficult. The HEWs can give you detailed information about ICCM and CBNS. It is HEWs that take part in a training program about CBNC more than anybody else. I have also attended a training program about CBNC. If you ask any other health worker about CBNC, they will not be able to give you detailed information. JSI gives extensive training to HEWS about CBNC. There were also other agencies in the area who were active in providing HEWs with essential training. However, we cannot claim with absolute certainty about the involvement of the health center to build the capacity of HEWs.

Q. Do you thank your participation in a campaign is the only reason that has prevented from capacitating HEWs?

A. We have no other problem worth mentioning. It is advisable for us all to go to the health post and gather information about the problem they are facing and how they are progressing. There are no many cases to be examined or investigated by the health center. Therefore, the health workers have ample time to travel to the health posts and provide them with appropriate support.

Q. Do they have transportation problem to go to the health posts and comeback here?

A. they may face a problem of transportation. One of the health posts is located relatively a great distance away from here/ the health center. But, if we are sincere, we can assign an expert from every department who gives the necessary support to HEWs to carry out their routine successfully.

Q. How often did you go to the health posts within the last twelve months to give them support they required?

A. in past many experts drawn from different units used to go to the health posts to give them assistance. But, within the current budget year, we did not give them any kind of assistance. What we did was just to assign an expert to provide them with an assistance that has relevance to CBNC. Earlier the focal persons for Mother and Child Care/ MCH/, children under-five, the pharmacy, Outpatient Department / OPD/ and health extension program coordinator used to impart or convey information or knowledge to the health posts. This way of proceeding to achieve a desired goal has already discontinued. But, experts are still going to the health post alone to give them support. We no longer go together. Owing to the above facts and circumstances, at the present time it is difficult to know how the health posts are operating.

Q. Have you ever given any ICCM support on individual basis?

A. It was the guy who went to the health post alone or without any other person. He used to visit certain numbers of health posts. But he did not give any ICCM support to the health posts because he has not attended any training on same.

Q. Did you have any problem not to go to the health posts?

A. We have shortage of manpower. I am in charge of two units/ departments. I am afraid some activities may stop occurring in my absence from here.

I am handling CBNC, ICCM and vaccination. It would have been good if two persons are assigned to manage two sections.

Q. Did you make any plan of action to deal with the problem you have mentioned earlier?

A. None.

Q. What things do you think should have been differently to keep on providing assistance to the health posts?

A. The law says a group of experts drawn from various departments must visit the health posts on a quarterly basis using a checklist with a required level of quality in order to make them capable of performing their usual activity.

But, we haven't followed the law because of a number of reasons. In a similar fashion the woreda health office is required to assist the health posts. But, that did not happen.

In addition, everybody is tending to rely on other entities for help. Generally, we lack strength or determination to help the health posts solve their problem. The job requires a well-trained facilitator. Besides, the lines or systems that join onto one another are broken. Formerly, every quarter.

people from the woreda health office used to make a visit to the health facilities to assist them. They checked the cleanliness of the health posts. I always discuss the problem with other people. You have personally observed the situation which the health posts are in.

Q. can you describe the reason why the interconnecting lines between the woreda health office and the health post not properly attached.

A. I have no idea about it. Previously, we were planning to do a lot of work. But, we accomplished nothing to achieve a goal or desired state.

Q. How much did the war between opposing forces in the country and the emergence of CIVID-19 affect your plan?

A. I don't think the conflict and coronavirus pandemic were and are having any impact as far as provision of any support to the health posts is concerned. They might have affected other activities. The war and COVID-19 cannot serve as acceptable reason or excuse for the health center not to support the health posts. I Believe lack of commitment of the leadership is to blame for not giving assistance to the health posts. But, we cannot deny the impact of coronavirus pandemic on a number of activities.

Q. can you mention something about the support given to the health posts by other entities to identify and treat neonatal infection?

A. Currently, only JSI gives the entire support to run the program. In addition, the woreda health office has given assistance to the health posts that is too small to be important.

They provided information to the members of the leadership about the provision of CBNC by the health post. Usually JSI is organizing a number of training sessions for HEWs.

it provides them with the means or technical support to efficiently carry out their routine. The woreda health office makes the main leaders of WDGs familiarize with the types of medical care given by the health posts despite the fact that WDGs are currently not functional.

I don’t know about any other entity that supports the program.

Q. do you have a system in their working methods in which HEWs invited or made to participate in a review meeting?

A. yes we have. We organize a review meeting every month. We examine the number of patients who were treated against diarrhea, CBNC, pneumonia, ICCM and any other type of illness.

Health facilities with very low performance rate give reasons for the variance. After that we take a new direction to improve our working effectiveness.

We enforce or compel our employees to organizational rules to improve their competence / to do their task well to achieve a desired result.

Q. do you take any action? If you do, what type of action?

A. we put a definite goal or purpose. But, some problems will cause a serious delay in our action or progress.

For example, we cannot claim that any HEW is busy doing her job unless she provides treatment for two patients per day affected by a diarrheal disease. the same rule applies to CBNC. Therefore, it is possible to assume that the health post was not open on that specific date.

JSI managed to give training to health workers and HEWs to boost their capacity to deal with any type of inconsistency.

Q. How do you evaluate or describe the benefits gained from the trainings / both conventional and unconventional/ to increase the capacity of health workers?

A. the training has provided the health workers with the means to improve their working effectiveness. Nobody was trained in the delivery of ICCM service. But there were some health workers who were trained how to treat CBNC.

ICCM is a new intervention for which health workers must be trained. But, some of us were given a momentary / very brief training to run the activity. They have made some changes to the previous guideline/ treatments.

We let the HEWs know or understand whether there is any change regarding follow up or supervision of activity etc. I am happy with the JSI for organizing training programs for HEWs. They are taken as far as to Debremarkos to attend a training program to acquire skill.

Q. Tell me about the capacity gaps addressed through trainings?

A. one must have confidence in one's ability to do a job. Health workers will be able to make a plan to implement CBNC or ICCM only when they have self-assurance in their ability to succeed.

Accordingly, the training has provided them with the means to have confidence in their abilities to put an instruction into effect. The second issue has to do with completeness / inclusiveness.

Likewise, the training has enabled them to make their data complete and inclusive. It also provided them with the skill or knowledge to recommend the correct type of drug to cure disease.

Q. Which type of support/s do you think has / have helped them improve their performance and discharge their duty efficiently?

A. JSI has done very commendable job to improve the capacity of health workers. to accomplish a task successfully. an expert from JSI used to come here to make information available for those who want to use it. When the expert gives a training to health workers, he is using words.

But when he gives them training at the health center, he is communicating tangible information that can be perceived by touch. He is showing lot of things to the trainees.

Q. which one of the two methods of providing training to the health workers do you think is desirable?

A. the one given at the health center desirable. My answer is based on the information obtained from the health workers. The actual application of a method is can produce more fruits than a theory or abstract thought.

Q. how much do you think this method of offering a training gain the approval of HEWS?

A. We all have different attitude or point of view about a particular issue. For example, I may see say I feel mental and physical exhaustion when I reply to the list of questions used to gather information in your research.

But, we will be feeling pleasure if we have an attitude of esteem or admiration for our work. As a result, the majority of health workers are showing pleasure. They accept that a child belongs to a society. We get great enjoyment and pleasure when the child affected by illness is completely cured.

Q. Do you think the support should be given in a different way? Do think the sponsor should come up with an updated version of training system in order to make it more realistic?

A. Many thanks for the support provided by JSI. It should continue providing training to health workers to make them feel more knowledgeable. The support offered by JSI will provide us with the means to rectify imperfection. But, JSI has to do more to improve the capacity of HEWs. The health workers have already been provided with most recent information about CBNC and ICCM.

It is desirable for the woreda health office to strengthen its connection with the health facilities. Usually people from the woreda health office are coming to the health center to persuade us to take part in a different mission such as vaccination campaign. That is the only reason for them to come here.

The health center is expected to increase the capacity of HEWs using different ways. It should organize a training program for HEWs using a checklist whose level of quality or excellence is accepted as a norm.

Q. How ECHIS implementation helps you with iCCM service delivery?

A. I did not take part in kind of training On ECHIS. As a result, I am afraid I may not give you detailed information about it. But from the layman's perspective, I can say that ECHIS is extremely important. Suppose let talk over about pregnant woman.

If we know the date when she became pregnant with a child, then we can easily calculate the date when she will deliver her child. After the woman delivered her child, she is required to attend postnatal care. she must get the newborn child vaccinated.

Generally, ECHIS will make it possible for us to identify the number of new born children in the respective localities and to inoculate every child against disease.

Q. Do you think ECHIS is helpful to identify a case or illness in a patient?

A. It may be helpful to some extent. ECHIS may recognize a newborn child and to be able to say he/she is not vaccinated. if the child is not vaccinated, we cannot protect him/ her against disease. Therefore, ECHIS is showing or suggesting that something exists or is true.

Q. Do you think ECHIS is helpful to provide a good quality service to clients?

A. yes it is. Suppose a particular HEW may claim that she managed to get vaccinated certain number of new born children in her catchment area. She may also possess a document or a book that bears full information about the children.

ECHIS will tell the HEWs whether or not my child got vaccinated. Secondly, it gives us information that describes where the building is or where the child lives. This is how we can ensure quality of service provided to the community by the health facility.

This condition will give rise to accountability. If they health workers are required to justify action, it means they will efficiently discharge their duty.

Q. does it have any benefit to gain access to or retrieve our data without any difficulty?

A. it may be difficult to enter our data on a computer disk. But after we enter our data about ECHIS into database, we can save the same from being lost. we can also get the information back very easily. It contains a lot of information about vaccine, the practice or principles of cleanliness etc. However, it is slightly difficult to enter data into file in a form that is suitable for storage in a computer.

The other problem connected with ECHIS is that if I go away from a place, the collected facts about my life story are not updated with the most recent information. Because of this reason, they are not able to make ECHIS functional.

Q. why don't you write the information on a card?

A. we do write it. But, some people mix things up to make them hard or harder to understand. Let us assume that I perform my routine in this room.

Let us also assume that today you have spent the entire day accomplishing some activities in the same room I am used to carrying out my usual activity.

May be you are a recent arrival. But if I am not going to update the information you have entered into database, then all the collected facts will be lost.

Q. If we include ICCM within ECHIS, do you think it will help us identify illness affecting a particular person or ensure quality of ICCM?

A. I don't think so. But I have no much awareness about it.

Q. What do you think if the chart displaying detailed information is uploaded?

A. that is oaky. But our success depends on the availability of continuous power supply. The question is whether or not we can use our mobile phone continuously. Will there be a constant supply of electricity? Certainly, the workers have a power bank.

Therefore, I cannot claim with absolute certainty that the application will solve our problem in its entirety. All our problems will be solved only when we have continuous supply of power and our mobile phone becomes fully operational.

Q. can you trace a defaulter using ECHIS?

A. yes, we can. For example, if a particular woman starts to attend antenatal care, it will show you whether or not she has continued to do so as per the appointed time. The system does not allow a passage for a new born child who is not vaccinated. it will out a red light.

Q. what do you think should be done to strengthen ECHIS?

A. Firstly, we need to update ECHIS with the most recent information than was previously available by taking reasonable care to avoid risk. We must put ECHIS data into a database in order to make it fully functional. I think it is good to make one category of ECHIS be or run parallel to that of another.

Q. Describe the main issues faced by the health system to identify and treat neonatal infections in the community. What are the critical factors affecting the **delivery of iCCM** to clients?

A. firstly there is severe supply / medicines/shortage. The second challenge is inadequate allocation of budget. The situations the health posts are in will make everybody feel disappointed. I don't think they deserve the name.

How can they heal a person affected by illness? The rooms are full of dust. In adequate allocation or lack of budget is to blame for the existing problem. The health posts don't have a guard to protect their property from thief. The problems facing the health post you have visited today are showing the tips of the iceberg. They are the small observable part of a much larger state of affairs.

I am sure you have noticed what the roofs look like. other health posts have neither a toilet nor a fence to enclose their area and act as a barrier. Thirdly, they don't get any kind of professional support.

Neither the zonal nor the woreda health offices nor the health center are having a feeling of concern to provide the health posts with professional support. Sometimes the health posts are not made familiar with incidents that are available only during one season or at specific times of the year.

For example, during some occasions we were not able to find anyone in the village to communicate information to the community about COVID-19. They were busy preparing land for crops or performing any other activity. We were able to find no one in the village to treat people affected by trachoma.

The same logic applies to ICCM / CBNC as well. Usually the health posts remain closed. In some health posts only one HEW is assigned to run the activity. There is no way to meet the requirements of the community by a single HEW.

Q. was the war between opposing forces in the country having any impact on the delivery of ICCM service to the community?

A. Both the conflict and COVID-19 are having direct or indirect effect on the delivery of community services. Nobody was concerned about the health of the community during the war.

Everybody's main concern including that of the health workers and officials was to avert the danger. Our concern was how to mobilize resources in kind and in cash for the war effort. Additionally, a number of health workers were sent to the war front to give care to the injured fighters and civilians.

The health facilities were not in use or operating during the war. HEWs were busy collecting bottled water and food from the community to assist the war effort. Therefore, the war was having disastrous effect as far the provision of medical care to the community is concerned.10242

Q. How long did the problem last?

A. It lasted for about three or four months or until the fighting stopped. COVID-19 was having disastrous effect on our routine. We started to inoculate new born children as soon as coronavirus pandemic was declared as a national threat. But, people stopped coming to the health facilities because they thought that they would be given vaccine against COVID-19 without their will or desire.

The community claimed furthermore that coronavirus pandemic was a political issue. As a result, the pandemic was having an adverse impact on every activity including the delivery of ICCM service.

Parents were not willing to get their child vaccinated. Similarly, we were not able to identify illness in the child unless he/ she got vaccinated. there was a widespread problem across the region as far as COVID-19 was concerned. There was public campaigning that tended to stop the public from going to health facility.

The community was hardly getting any kind of including vaccination or neonatal or postnatal care or CBNC. Last time there was public campaigning against trachoma. But, at that moment we were surprised to hear that the health workers were administering a pill against COVID-19. They did not want to accept our words as true.

Q. How do you think the COVID-19 pandemic and/or COVID-19 response measures affect the uptake and/or delivery of iCCM services?

A. It was having a powerful effect. A lot of things were said to the public about coronavirus pandemic that contradicted what the government has said.

Only a small number of people were used to come to health facility at night after their condition / illness became worse. Nobody was going to the health post to get treatment or any other kind of service.

Q. How did you manage to bring about a positive attitude to change?

A. The beliefs that affect community's attitude about coronavirus pandemic and other related issues still continue.

Q. why was the community showing a feeling of anxiety or apprehension about vaccination against COVID-19?

A. The community used to fully accept coronavirus pandemic as a political issue. The community residing in this neighborhood believed that the Tigrians are having the art of using alleged magical power to produce lethal/ deadly drug.

The community assumed that the enemy is planning to use the drug intended to cause death if it loses the war. The onset of COVID-19 and the war declared by the enemy happened at same time by chance in a surprising way.

Secondly, at the beginning the health workers were not certain about the effectiveness of the vaccine. No date of expiry was written on the object containing the vaccine. Many people claim that it takes 10-15 years to conduct a research and produce an effective remedy for an illness.

So, well-informed community members were under suspicious circumstance. Thirdly, we have started to observe minor side effect of the drug. After they were inoculated some people became sick of diarrhea which they claimed as a symptom of harmful effects of the drug. I myself was sick of rheumatism and diarrhea.

Q. did you take any action to reverse the mindset / way of thinking of the community about the vaccine?

A. We have exerted strenuous effort to provide the community with the means or information to develop a positive attitude toward the drug. Every morning we used to convey a message to the community about the presence or absence of illness or injuries. COVID-19 was one of our agendas. We were telling the people who were in attendance of the meeting about the effectiveness of the vaccine.

We worked hard to pass on knowledge to community during religious service inside the churchs' compound. What made us disappointed was that the community started to consider COVID-19 as part of their religion.

Let me tell a story about a priest. He asked me some questions why we did inoculate every person against coronavirus pandemic. I tried to explain to the priest about the reasons. Then he argued that COVID-19 is 666 or relating to Satan worship. I tried to convince him that the government does not intervene in religious affairs.

But I was not able to change the priest's attitude. He said that he would quote something from the bible to support his argument. I agreed to his idea. The bible says to mark the skin of one's left hand and right hands with a tattoo or permanent design, then the tattoo amounts to 666 or Satanism /the worship of Satan.

However, I told the priest that we don't form a tattoo but protect somebody against coronavirus pandemic. I reiterated that the sign formed during the inoculation will disappear shortly. Anyway, we have succeeded in changing the attitude of the community for better despite some challenges.

Q. What are the high-level benefits that are attributable to this support/IR?

A. I think the support enabled us achieve a desired result. The community stopped to get medical care after the onset of COVID-19. Then, we managed to persuade the community to resume using the service after a temporary

halt. But, the temporary halt made the community neglect everything. Generally, we found the assistance very useful. More importantly, the assistance has provided us with the means to improve the capacity of HEWs. secondly, the assistance has provided us with the knowledge to gather good quality data. Thirdly, the support increased the strength and depth of our success.

Q. Tell me about your main achievements

A. The success achieved to boost the capacity of HEWs is worth mentioning.

Q. Please explain to us the feasibility of this support/ IR for national scale-up?

A. I think the activities implemented by JSI are all feasible or capable of being achieved at a national level. Our community experiences a lot of problems some of which need such as CBNC swift interventions. We attribute the change of attitude on the part of the community to JSI's all- round support.

Therefore, the support given by JSI to build the level of knowledge of the community can be cited as a feasible strategy. Secondly, to our satisfaction and to the satisfaction of all other stakeholders, the quality of data gathered by HEWs has improved.

I think their system of data registration can be taken as excellent example that deserves to be imitated elsewhere in the country. For example, JSI may consider of implementing a particular activity together with the health post.

JSI may critically investigate data registration methods. It makes sure that all the necessary conditions are met. JSI will check whether or not HEWs offer treatment to their clients as per the chart showing detailed information.

Q. Do think HEWs have the requisite capacity to keep the project going?

A. It is questionable whether the HEWs to keep the activities going if JSI withdraws its budgetary and technical support. But, it is possible to make the project continue to exist with the full support of the health center and the woreda health office. But the series of chains that are connected in one way or the other to offer support to a fellow member of a group or organization are broken.

Q. WHICH of these activities do you think will keep on going without any assistance from outside?

A. I think ICCM and CBNC will keep on going or continue to exist without no one's support.

Q. what favorable conditions were put in place to keep on these activities going?

A. They should keep on providing support for the health post and health center in order to keep implementing ICCM and CBNC continuously.

Q. it is obligatory to give them support?

A. it is not clear for me. Nowadays we don’t see a lot of people showing persistent and hard-working effort to carry out a task. Civil servants or employees in a government organizations are too reluctant to give us the required service from

Q. do you think the capacity gap of HEWs has been fully addressed to carry out their routine successfully?

A. yes I do. I think there is lack of sense of ownership of the project. HEWs and the leadership seem to demand or anticipate receiving something tangible in return for the jobs done.

lack of incentive was the main reason for members of WDGs to stop involving in any kind of activity. The smaller the amount of money paid or is due to be paid, the smaller the work done by a person. I think the whole issue revolves around getting financial reward.

Q. Please describe how you have incorporated ICCM into your regular activity such as plan of action, report, support etc.?

A. it is not as strong as it should be. But, it is mandatory to incorporate ICCM and CBNC into our routine. Firstly, we make our plan of action for ICCM.

Q. Do you have a plan for a new born child less than two months old and for children under-five?

A. yes we do. we also have a review meeting every three months. We conduct supportive supervision.

End line evaluation of the PSBI implementation research (IR), the qualitative component

I	Identification	
1	Questionnaire ID	**Lume-Daka Bora PHCU-Director-IDI-L&T**
2	Area Identification	
3	Name of Woreda/Zone/Region	**Woreda**
4	Name of facility	**PHCU**
5	Name of moderator	
6	Name of a note taker	
7	Date of discussion	**13/06/22**
8	Participant #	**1**
9	Audio File #	**1**
10	Start time:	**_8__:_30_**
11	End time:	**_9_:_11____**
12	Transcriber	

Key: I: interviewer

R: responder

I: HI, my name is Temam and my colleague is Lalisa. We are from the JSI L10K project. We are here to gather information on the ICCM project for end evaluation. I thank you for your willingness to do this interview. Can you please introduce yourself and let us proceed to the next question?

R: ok, thank you, I am the director of Daka Bora PHCU. It is a health facility under Lume woreda. My profession is BSc. nurse.

I: Can you please describe the demand generation/SBCC activities you are doing on iCCM/ newborn health? What are the strategies used to raise awareness?

R: frankly speaking, iCCM is the service that saved the lives of plenty of neonates. Even though diseases are not prevalent here, the JSI L10K project had contributed a lot. A wide range of activities was accomplished. I like to thank the project very much. On top of saving tremendous lives, it provided plenty of pieces of training in different administrative structures. Starting from the PHCU director up to the kebele level HEWs were trained by this project. Then awareness creation was done using community one to five linkages and WDA. The HEWs provided adequate information about iCCM by going home to home. By maintaining each of the communicable and non-communicable diseases, they were informing them what advantages the iCCM does have. The HEWs did very effective activities regarding the hygiene and sanitation of neonates. Hence we are providing all-around awareness on our side.

00:02:56

I: did you try to use religious leaders, pregnant women conferences, elders, schools, and the like for awareness creation?

R: the HEWs provided awareness by gathering the WDA. The HEWs as well as our HC professionals gathered the WDA leaders and disseminate the information to them. They then transferred the awareness to their members. We were evaluating their performance and checked their effectiveness. That was how we provided support for HEWs. The easiest way to disseminate the information is using school children. On our side, awareness creation at the school level is satisfactory. We were conducting reforms home to home, at school, and others to disseminate the information.

I: What are some of the biggest challenges with SBCC activities for newborn care?

R: there is no significant reason that challenged us for information dissemination about iCCM. Regarding skill gaps with the HEWs, both we and the project is providing different kinds of pieces of training. We, based on our educational background, were providing support for the HEWs at the HP level. A simple challenge observed is on the tablet. Sometimes it doesn't synchronize or fails to function. We inform the issue to the woreda and higher administrative hierarchies.

I: How do you describe the strength of the support you got from the PHC and the project as well as your efforts to address barriers to delivering iCCM/PSBI during COVID-19?

R: the support provided was not limited to iCCM only, rather it had contributed a lot to the general health care service. Especially regarding the pregnant mother conference and care for mothers was the most astonishing activity. The project did awareness creation and fulfilled skill gaps. It enabled us to prepare an action plan and work on our shortcomings. It had shown us a variety of directions to act upon. Hence, I can mention a lot of strong activities performed by the project. The project enabled us to work on the tangible data about the number of available households and the associated population practically. This helped us to increase our performance and service delivery. The once used to be low; is now effectively fitted. Because we are doing well by now, I think it will be better than what is currently observed.

00:09:50

I: what do you suggest should be done differently?

R: I suggest the training should be provided at any possible level. It has been provided, but better to increase it more than this. The supportive supervision, on the project side, shall be continuous. The other is the failure of the tablet to synchronize should be solved.

I: Did you get the support system helpful (mentorship and on-site coaching methods, supervision, technical support, PHCU level PRCMM, etc.) to enhance your skills?

R: the activities undergoing are directly related to skills. It is about mentorship. The activity increases the skill to a higher percentage. Because they are up to date and focus on the effectiveness. Especially at this time of COVID; when that was complicated by another disease called monkeypox.

I: does the support increase your acceptance in the community?

00:12:44

R: the onsite coaching support contributed a lot. Because we are using it as an example to apply to our daily activities. It has shown us a variety of techniques. Even though the support is from NGOs, the support was directly fitting our governmental activities. It was according to the way we can address all the children in our catchment.

I: how do you compare the onsite coaching with the offsite training?

R: demonstrative the procedures on-site for the HEWs are unforgettable. The activity was prone to be forgotten, in the previous times. This one is unforgettable because it is directly applicable. Training off-site can be forgotten, but onsite training is given at the real place and is memorable.

I: How does eCHIS implementation help you with iCCM service delivery?

R: eCHIS has a great advantage. It shows practical and quality pieces of evidence. I am reliable on that because it is advanced. It is a paper-free task. It has helped us a lot. It had shown us what we have and what we don't. In the previous times, by the name of eligibility, we were given tasks that were not real. But in the eCHIS, we identified our real target and applying on it. had there no eCHIS, there couldn't be iCCM.

I: how advantageous is it in case identification and management, prescription, quality data retrieval, and follow-up?

00:16:10

R: it guides in case identification and treatment. We were provided training on how to operate the eCHIS. About HEWs application, referral application, and focal person application were provided. For example, regarding referral cases, there is a guideline for the focal person at the HC. The way referred cases can be identified and action cards should be managed as indicated. It does the same for HEWs as well. The application has a module in it in which it can show diseases, case management, household leaders listing, and a variety of information. Hence, all information required can be accessed easily. Hence it is the tool by which we can easily manage activities. It can simplify diseases and associated medicine.

I: How was the introduction of the iCCM/CBNC module into eCHIS affected the iCCM/CBNC service delivery?

00:19:26

R: the activity was shifted from the paperwork to electronics and became practically shown. As long as the disease and its management are clarified, it is easy for us to decide. It explains every problem in the process.

I: How does it help in follow-up and default tracing?

R: for example, if one mother came to us as a referral case, it shows us on the tablet. The action card displayed the information for the mother on the referral and also provide feedback for the HP regarding the status of the mother. The HEWs can send cases beyond their capacity as a referral to HC or decide to treat at the HP level. Hence, the CBNC module integrated with eCHIS has a great advantage.

I: how it helps in the identification and registration of sick newborns?

R: the newborn is registered in tier own folder. In the process, it can tell how healthy the neonate is.

I: how about data retrieval?

R: it is simple to trace back the data. At the time you click on it, it shows the aggregate data. It can show the number of children treated up to now.

I: How does it help in keeping quality data?

R: it had contributed a lot regarding data quality. Because the once recorded data is saved safely. We can miss the steps while recording data on hard paper. But this one cannot miss the steps. It doesn't allow you to escape any step. It shows the mistake by red color. It is error-free.

I: How does this help regarding client satisfaction?

00:23:38

R: the farmers were not familiar with tablet service for several years during iCCM service. The community knew the service and are satisfied.

I: what do you suggest to be improved regarding eCHIS and CBNC module integration?

R: the tablet sometimes does not allow to delete information once entered. We are consulting experts at the woreda level for this issue. At the time a given household leader has left the area, it doesn't delete; rather goes on

increasing the household number. So does the WDA information. The once entered listing was not able to update. Otherwise, there is no significant issue that I can suggest to be improved. It is good as it is.

I: Describe the main issues faced by the health system to identify and treat neonatal infections in the community. What are the critical factors affecting the delivery of iCCM to clients?

00:26:08

R: there is a challenge with topography and distance. That restricted us from identifying case and providing treatment. Shortage of manpower is another issue. We have 7 HPs, but the HEWs are only 10. This restricts us from addressing the community effectively. The HEWs are not able to stay in the community to provide service day and night; due to a lack of residence in the vicinity of the HP. lack of transportation also affects the service.

I: How does lack of competence with the HEWs restrict the service provision?

R: all of the HEWs are not equally competent. Because they differ in skill and experience. For example, level 4 and 3 HEWs are different in skill. The HC professionals are also first degree or diploma level. Hence they do have different skills. The others differ regarding exposure. What we want from you is that diversified training should be provided; either on the job or off the job.

I: how does this affect the iCCM service?

R: it caused the inability of providing quality and adequate service. The most challenging one is the lack of medicine. The medicine is not available even at the HC level, let alone HP. we are not able to buy some of the medicine from the market. That can cause complaints from the community and hence affect the iCCM service. For the time being, irrespective of the shortage of training, the HEWs are doing their best.

I: How does the low demand from the community affected the iCCM service?

R: I don’t think there is low demand from the community. The issue of health is not refused by anyone.

I: how about a security issue?

R: there is no security problem in this area that can affect eh service.

I: how about COVID?

R: it is a national problem. It collapsed everything.

I: How does it create a problem?

00:30:40

R: one problem is fear from the community. There was a time when the community was resisting seeing the health professionals. The health professionals were not able to treat mothers and neonates due to this reason. Even they used to run away; when they see us wearing a face mask. Currently, because they got awareness through different directions, including media, they are not that resistant. We are also smoothly coming closer together with the HEWs, to provide awareness.

I: How does this affect the routine activities?

R: it had affected a lot. For example, the activities we used to provide home to home were interrupted. The community was not worrying about other disease-causing agents; rather pay attention to COVID due to the fear of death by the virus. That had greatly affected our performance dropped.

I: How were the neonates harmed due to this situation?

R: because it was not possible to go home to home, they were not getting health care services. But we used different strategies like a loud speaker, home-to-home visits, and media to create awareness. We have gradually convinced them.

I: how does it affect the livelihood?

R: it had affected a lot. There was no religious activity practicing, no gathering for ‘idir’, and any movement was stacked.

I: what strategy did you use to withstand the impacts of COVID and restart the service?

00:33:56

R: we paid strong attention to awareness creation in the community. Together with HEWs and the health development army, we moved home to home and provided awareness about how to prevent COVID. Keeping a distance, wearing a mask, washing hands, and putting flats soaked by chemicals at the entry of their house gate. The community started to apply it and the vaccine arrived. Currently, there is no worry.

I: what was done to motivate the HEWs?

R: because the community who got awareness started to seek health care service, they started to provide the service.

I: What are the high-level benefits that are attributable to this support/IR?

R: it has helped to provide quality service for the children. The service has become standardized. Both the HEWs and health professionals are similarly providing the service. It boosted their trust in the HEWs service. The trust they have in us is the same for the HEWs as well. As you know, the community likes to compare the health professionals in their skills. That is solved by the iCCM service provided at this time. Now every HEWs worker is providing the same kind of treatment. It also helped in reducing neonatal death.

00:38:45

I: Please explain to us the feasibility of this support/ IR for national scale-up?

R: definitely. We only saw its advantage not disadvantage. Had it been done at the national level, it can save the lives of a tremendous number of the neonate from dying. It is possible to scale up by extending the training to the national level. It is better to increase the attention paid to this service and make its agenda for the government.

I: what are the enabling factors to scale up and what are the obstacles hindering?

R: insecurity can be an obstacle to not scaling up. The other obstacle is the shortage of transportation. Lack of residence for the HEWs in the vicinity of HP is another obstacle. Lack of training can also be an obstacle.

I: How are the activities/efforts embedded in the PHC and woreda routines/work streams?

R: yes we can continue the activity; what was new in the system and how to become effective in the iCCM service, otherwise the service was there. Hence if it is incorporated into the routine activity, it is very interesting.

00:42:30

I: how do you support it with planning?

R: we are focusing on the available children. The plan and performance go on like that.

I: do you evaluate it at the PMT level?

R: yes, but we check it as a general service, not specific to iCCM. Because LQAS is done randomly, if a child's disease is selected randomly, we consider it.

I: Do you have anything think is important to tell us that we have not asked you?

R: I don’t have much to add. I like to inform you to add additional helpful functions to the tablet. it is better to strengthen the supportive supervision. I suggest you focus on additional communicable diseases included in the tablet.

I thank you for your time and valuable information.

End line evaluation of the PSBI implementation research (IR), the qualitative component

I	Identification	
1	Questionnaire ID	Lume-Daka Bora PHCU-Haro Yohannes-HEW- IDI-L&T
2	Area Identification	
3	Name of Woreda/Zone/Region	**Lume Woreda**
4	Name of facility	**PHCU**
5	Name of moderator	
6	Name of a note taker	
7	Date of discussion	**14/06/22**
8	Participant #	**1**
9	Audio File #	**1**
10	Start time:	**_8_:_30_**
11	End time:	**_9_:_21_**
12	Transcriber	

Key: I: interviewer

R: responder

I: Hi, my name is Temam and my colleague is Lalisa. We are from the JSI L10K project. We are here to gather information on the ICCM project for end evaluation. I thank you for your willingness to do this interview. Can you please introduce yourself and let us proceed to the next question?

R: Yes, I am willing to participate in this interview. I am Daka Bora PHCU, Hora Yohannes Kebele HEW.

I: Can you please describe the demand generation/SBCC activities you are doing on iCCM/ newborn health? What are the strategies used to raise awareness?

R: we were provided training and started providing iCCM service in the community. We were conducting identification of children less than five years of age.

I: How did the community know about the service?

R: using 'Afosha', the traditional money contribution by the women, and different conferences, we were telling them. We were telling them that those children who are sick can get treatment with us. We also tell them about the service while we move home to home for PNC service.

I: How did you use WDA in the awareness creation?

00:02:43

R: the WDA helps us a lot. They tell us about the women who delivered, immediately. Even though we are not able to provide PNC service in two to three days, we try to provide the service in three weeks.

I: How did the community engage in the awareness creation?

R: they participate to some extent; even though the number of children in the kebele is very few. The majority of them take their children to HC. They tell each other about the iCCM service among themselves.

I: How do you describe the strength of the support you got from the PHC and the project as well as your efforts to address barriers to delivering iCCM/PSBI during COVID-19?

R: they provided us with training.

I: How does the training help you in the iCCM service?

00:05:17

R: we were not participating in the treatment of under-five children. It was after we got the training, that we started to provide the service. It increased our knowledge.

I: how about other support?

R: there is supportive supervision every week from the HC. They check our performance and give corrections regarding our mistakes.

I: How does the material supply or support provide helped you to provide the iCCM service?

R: I was in education during the COVID pandemic. There is no significant challenge then after.

I: what kind of changes happened after the support?

R: after we got the training and knowledge change happened. Then we started providing treatment services; which we were not doing. It enabled us to treat the children.

00:09:19

I: Was the support you got from the project and PHC helpful?

R: yes, it was helpful. Even though there is no significant number of children who felt sick and came to us.

I: What could have been done differently?

R: support should be there to enable the HEWs to deal with the variety of packages. Because we might forget them. Material support is required. For example, weight balance or medicine should be fulfilled. In the absence of medicine, if we tell them to come and take the service and are not able to provide medicine, it becomes an obstacle the second time we tell them to come.

I: what kind of medicine is lacking?

R: they prefer syrup over the tablet. They don't consider tablets as useful as syrup. They want to divert their attention toward HC.

I: why syrup is preferred over tablets?

00:11:51

R: they argue by saying children cannot chew it. The syrup is also easy to give them. But we tell them that the medicine is similar and can be given by diluting in the water.

I: how quality is the water?

R: it is obvious that the drinking water source is surface water. We tell them to boil the water or buy bottled water.

I: is it not possible to provide the syrup?

R: I have never seen the medicine in the form of syrup. But it is good if it is available readymade.

I: Did you get the support system helpful (mentorship and on-site coaching methods, supervision, technical support, PHCU level PRCMM, etc.) to enhance your skills?

R: it is helpful to remind us what we forgot. It enhances us to continuously provide the service. it also improved our competence.

00:13:39

I: how do you evaluate the difference between onsite coaching and off-site training?

R: there is not much difference. It is good if we move to other place and get the training. But the onsite coaching is helpful by showing things practically.

I: How was the review meeting helpful?

R: review meeting is for the evaluation of activities performed. To see how much is done and what is left. It is better to improve our progress; to provide continuous service.

I: are the onsite coaching, mentorship, and other technical supports acceptable?

R: yes, but it is better if they are provided being together with other HEWs. Because one is different from the other in understanding, it is better to be together and share the experience.

00:17:13

I: how do you suggest should the support be improved?

R: I suggest the support should be continuous and supplies are fulfilled. So that the community access the service in their vicinity.

I: How eCHIS implementation help you with iCCM service delivery?

R: we see every part of the children. The eCHIS help us in identification. It corrects the mistake committed. This one is very helpful. It also assists in prescribing the medicine. It shows the dosage and kind of medicine that should be ordered. It has helped me a lot.

I: How does it help in data retrieval?

R: it doesn't show you the number of services we provided. We get that information only from the register. It only shows those on follow-up.

I: how about its support regarding data quality?

R: I can say it is of better quality. It has the data about all the households in the kebele. It tells the number of children and pregnant women available in the community. it has a great advantage in keeping the quality of services.

00:21:04

I: how about an appointment and default tracing?

R: at the time appointment is passed, it shows red color.

I: what do you think should be strengthened in the eCHIS service?

R: at the time I am busy and provided vaccination without the tablet, it cannot record the data in the tablet later on. It also cannot retrieve the data about the children who got service. When a lot of children come for

vaccination, it makes me busy recording each child on the tablet. Then I decide it to record later after I complete the service. But I am not able to record the data of these children on the tablet at the end of the day.

I: How was the introduction of the iCCM/CBNC module into eCHIS affected the iCCM/CBNC service delivery?

00:25:43

R: it makes our service faster and guides us in every step.

I: How does it help the identification and registration of sick newborns?

R: it helps to treat the neonate while we provide PNC for the mothers.

I: How does it help in clinical and referral decision support?

R: I had sent one referral case up to now. At the time I sent it, there is nothing that remains on the tablet. Only those I treated and appointed can be seen on the tablet.

I: how do you know the status of the baby sent as a referral?

R: there was no feedback provided for me after I sent the baby.

I: how do you identify the satisfaction of clients with the tablet?

R: the majority of them prefer the HC. For the children I treated, I revisit them and they showed me a good response.

I: do you have any suggestions to be improved regarding the module?

R: it is better if it shows me the number of children I treated. It doesn’t remove the name of pregnant women after delivery. It stays where it is on the tablet. So do those children who completed the vaccines.

00:32:07

I: Describe the main issues faced by the health system to identify and treat neonatal infections in the community. What are the critical factors affecting the delivery of iCCM to clients?

R: the community is not willing to bring their children to us. It is because the HP infrastructure is not in a good condition for them to access the service. Even though they got the awareness, they prefer HC over the HP. they are also not bringing their children on the 45th day for initiation of vaccination. They are still thinking traditionally. They sometimes wait for baptizing before vaccination. They can be changed if we strongly provide education. But I am the only one in that kebele and get busy with a variety of activities.

00:35:31

I: what are the other issues you face in the community?

R: while we move for community service like health insurance, the HP might be closed, that can be a reason for them to bypass the HP to HC. We were told to open HP twice a week, but we can be busy in the field the whole week.

I: How does the HEW's lack of competence affect the iCCM service?

R: I can provide service for the one I can do and send as a referral case to the HC for the other beyond my capacity.

I: How does the shortage of supply and support affect the iCCM service?

R: supply problem was there formerly; now it is solved; so does the support.

I: How does low demand from the community affected the iCCM service?

R: they have the demand but due to the closure of HP, they might not access the service.

00:39:44

I: how does COVID affect the iCCM service?

R: I was not around during the pandemic, currently it is not that challenging.

I: don’t you have any information on how it was during the lockdown?

R: it was serious. No one was able to come closer. It was terrible to provide service at that time.

I: hence it affected your work on newborns?

R: it might be at that time.

I: How does the COVID affect the livelihood of the community?

R: the society was not able to go to a religious organization. It was very serious at the time. But I was not around at that time.

00:42:34

I: you don’t know the strategies used to adapt the COVID to provide the service?

R: yes

I: What are the high-level benefits that are attributable to this support/IR?

R: it enabled the sick babies to get treatment in their vicinity. Such kind of service was not there and it gives us the chance to develop skills. The community was not aware of the service we provided, but they realized that the service is being provided and they started to get the service.

I: Please explain to us the feasibility of this support/ IR for national scale-up?

R: yes it is possible. Because there is HEW everywhere. At the time children are sick, she can provide treatment at HP. The availability of health professionals everywhere is also an asset.

I: what do you think could be an obstacle not to be scaled up?

R: manpower with adequate training has to be there. Availability of resources and medicine has to be ensured.

I: How are the activities/efforts embedded in the PHC and woreda routines/work streams?

R: it is better to support by planning. But there is a limitation regarding outcomes. But if follow-up has been applied the performance could increase.

I: is it possible to plan for the iCCM?

R: yes, by counting how many services were provided.

I: what are the components of iCCM that were used for planning?

R: no anwer

I: have you ever planned for iCCM?

R: yes, together with the general activities. The planning for iCCM is done based on the diseases like pneumonia, diarrhea, and the like.

I: how do you evaluate your plan with performance?

R: the plan is a plan and performance is also a performance.

I: Do you have anything think is important to tell us that we have not asked you?

R: I don’t have anything to add

I: thank you for your time and valuable information.

End line evaluation of the PSBI implementation research (IR), the qualitative component

I	Identification	
1	Questionnaire ID	Lume-Daka Bora PHCU-Vice Director- IDI-L&T
2	Area Identification	
3	Name of Woreda/Zone/Region	**Lume Woreda**
4	Name of facility	**PHCU**
5	Name of moderator	
6	Name of a note taker	
7	Date of discussion	**14/06/22**
8	Participant #	**1**
9	Audio File #	**1**
10	Start time:	**_3_:_30_**
11	End time:	**_4_:_28_**
12	Transcriber	

Key: I: interviewer

R: responder

I: Hi, my name is Temam and my colleague is Lalisa. We are from the JSI L10K project. We are here to gather information on the ICCM project for end evaluation. I thank you for your willingness to do this interview. Can you please introduce yourself and let us proceed to the next question?

R: Yes, I am willing to participate in this interview. I am vice director at Daka Bora PHCU.

I: Can you please describe the demand generation/SBCC activities you are doing on iCCM/ newborn health? What are the strategies used to raise awareness?

R: the majority of the HEWs were taken the training. Few of them, who were on study leave did not take it. To create awareness, we used the pregnant women conference and different platforms. They knew that they can bring their baby to the hospital or HC while sick. That had harmed the children very much. After the initiation of the project, we started informing the community that the HEWs can give the service at the HP level. That was how iCCM service started at the HP level and the community was utilizing the service.

00:02:55

I: How did you use the WDA, kebele structured, students, and religious leaders for awareness creation.

R: as I told you before, the one I said about different platforms was about using the different meetings conducted in the community. They were surprised when we told them about the service at the HP. and we tell them the iCCM service starts at the HP and goes on to the HC and then the hospital. We told them that they can also treat us just like one of us.

00:04:22

R: they used to raise a variety of issues and we responded by saying the HEWs can provide treatment. At the time problem happen, that can be reported to the respective bodies.

I: What are some of the biggest challenges with SBCC activities for newborn care?

R: not only at the HP level, but we also face the challenge at the HC level. We are not getting the new medicine included. We used to provide Cotrimoxazole and that one is not allowed by this time. Because it is shifted to prophylaxis. Ciprofloxacin was used in place of it and this is not easily accessible on the market. There is no challenge with the awareness. But at the time they go to HP and did not get the medicine, it is a challenge. Shortage of manpower, two are expected and only one is available in the HP by now. A single person is hard to provide service to all that need or might face closure of the HP at the time something happens to her. Hence, most of the time, the community complains that the HEW is not available at HP. we are providing solutions for that.

00:08:14

I: How do you describe the strength of the support you got from the PHC and the project as well as your efforts to address barriers to delivering iCCM/PSBI during COVID-19?

R: all of the professionals at the HC are providing support to every HP every week. During the campaign, we give them an additional task. They are expected to assess iCCM service on children side by side.

I: Was the support you got from the project and PHC helpful?

R: this is unquestionable. The clients wouldn’t have come if the service was not important. We are checking in the tablet as well as the registers for the services provided. Saving the lives of children in a tight situation infers its importance.

I: if it is helpful, how do you explain the changes that happened due to that?

R: the community is not exposed to unnecessary costs. The HP is found very far from HC, so it is hard for the community to come to the HC. The child can be harmed on the way to the HC. I can say there is a change. The training provided enabled both the HP and HC professionals to provide standardized services.

R: What could have been done differently to improve the support provision?

I: as I told you before, there is a big challenge with supplies. We are the ones who support HP by investing money. At the time we buy and give the medicine for free service at HP, it is a big challenge. In the future, it might create big trouble for the HC. You can imagine that a cost of a single glove is 44 birr from 12 birr. We are providing it for free to the HP; so does the medicine. It is better to increase the manpower in the HP. there is a big burden in the HP that one could not handle. On top of the variety of campaigns, huge regular activities are done.

00:15:02

I: how about support regarding supervision and the like?

R: if we mention all of the challenges, they are tremendous. For example, the problem of transportation is there. At the time I got one motorbike, I was able to cover all the seven HPs in one day. I am using a very old motor now. We are struggling with it to provide support. We are using a single motor among ourselves to accomplish a lot of activities. There are areas which are very far from here. That can take about 3-4 hours on foot. I suggest this should be fulfilled.

I: Did you get the support system helpful (mentorship and on-site coaching methods, supervision, technical support, PHCU level PRCMM, etc.) to enhance your skills?

R: the HEWs are getting the training; so does those of us who are in management. But the health professionals who supervise the HEWs did not get a single training. Their support does not match that of the HEWs. The HEWs were already developed experience in iCCM. They were supposed to get more knowledge than the HEWs.

I: does the onsite coaching and mentorship contribute to the improvement of HEWs' skills?

R: this is obvious; that anyone can see. It has changed a lot. They were providing only FP service. Because they got different training, just like the other professionals, they can provide iCCM service. I don't think this could have happened in the absence of the training. There might be one who did not apply the training or is about to apply. We will gradually solve it.

00:19:24

I: how do you see the onsite coaching and mentorship over the offsite training and mentorship?

R: in the absence of this support, the change wouldn't have to come. It wouldn't have been better if they are gathered at one place and got the training. But we are not able to do that. The HEWs had taken a lot of training.

I: which one do you prefer on-site coaching and off-site training?

R: I think on-site coaching is most preferable.

I: what do you think should be done to improve the support?

R: it is better to provide training for supportive supervisors. Some supervisors are directly from a college or university. Sometimes they are not able to answer questions from HEWs.

00:23:15

I: How does eCHIS implementation help you with iCCM service delivery?

R: We are about to collect demographic data and not complete yet.

I: How does the eCHIS help in case identification and management?

R: they can provide service at the HP using the tablet. They also use it for case identification and management while moving home to home. Hence I can say it has a huge advantage. Even though it is not supported by the laboratory, it can identify cases and prescribe medicine. Hence it is guiding.

I: how about data retrieval?

R: it has to follow up mechanisms. At the time it is passed, it shows by color. Hence it is good to remind the appointments and make the client come for service.

I: How does it help in default tracing?

R: the different appointments can be given. Three, four, or seven days appointments can be given. We were not doing that.

I: How does it help in data quality?

00:26:07

R: it is unquestionable. The data quality can only be solved by this mechanism.

I: do you have suggestions for the improvement of eCHIS?

R: not at all

I: How was the introduction of the iCCM/CBNC module into eCHIS affected the iCCM/CBNC service delivery?

R: it helped to provide treatment wherever the child is located. In addition, it is guiding the case identification, management, and prescription.

I: How does it help in the identification and registration of sick newborns?

R: they were providing ANC service for the mother, at the time she deliver, they know it and provide an assessment for the mother and her newborn child.

I: how do you trace the client satisfaction?

R: the community does not keep quiet about the service we provide. At the time they are not satisfied, they reflect it by any means they encounter. Otherwise, we don't know about the feedback regarding satisfaction. Maybe the HEWs know how it functions regarding client satisfaction.

I: how advantageous was it for clinical and referral decision support?

R: there are under five focal people who took the training. But the tablet provided was not functional then after. The one with the HEWs is up to the standard and ours is not. Even though we are doing well, the issue of the tablet is a challenge. I suggest it should be corrected.

00:30:07

R: the module guides step by step. It shows what should be done in each step. At the time it is beyond her level, it guides her to send as a referral. It also shows what should be done before sending the child as a referral.

I: does the module guide about the appointment, the dose of medicine, and the follow-up?

R: yes, I saw the previous one and it has such a guide. It is easy to use.

I: Describe the main issues faced by the health system to identify and treat neonatal infections in the community. What are the critical factors affecting the delivery of iCCM to clients?

R: the biggest issue is the distance between the HPs. The HEWs live in Modjo or another area. The child might be sick in between and may lack the treatment. It is better to avail residents of the HEWs.

I: How does lack of competence among HEWs affect the service?

R: there might be an individual difference regarding skills and knowledge. Because the HEWs repeatedly received training, I think they are in a good condition.

I: do you think the training they received is adequate?

R: it is not possible to say it is adequate. Because it is prone to be forgotten; unless refreshed. I think it would be better if it is given again; it strengthens them more.

00:35:09

I: How does the shortage of supply affect the iCCM service?

R: as I told you before, what is in the guideline and the medicine we have at hand is not matching. It guides to order Cipro, but it is not amiable; even at the HC level. It is becoming an obstacle to providing adequate service. It will be very challenging in the future. We purchase the medicine and provide it for free to the HPs.

I: how about a security issue?

R: there is no security problem here.

I: how about COVID?

R: because we are creating awareness and providing the vaccine, I think we will complete it by today.

I: How do you think the COVID-19 pandemic and/or COVID-19 response measures affect the uptake and/or delivery of iCCM services?

R: 00:37:12

R: not only on iCCM but the general service was also affected by the COVID pandemic. People were not able to come for service at the time. They were restricting sick children at home. The community is not comfortable with the home-to-home service by the HEWs. They were suspected due to their contact with a variety of people. They used to hide their children from the HEWs. This time, because the vaccine is being provided, there is no significant challenge regarding COVID.

I: How does the pandemic affect the livelihood of the community?

R: after treatment using traditional medicine, the community brings the child to the health facility being severely ill.

I: how were the COVID-19 adaptive iCCM implementation strategies?

R: they used to avoid the health professionals, by suspecting them. But after awareness is provided together with the vaccine, they became free of the fear. The health professionals, by taking the vaccine, started to save the lives of mothers and their children. They were providing awareness on any platform they encountered. They were also creating awareness by going home to home.

I: what was changed after the adaptive iCCM implementation?

R: we were convinced of those who claim COVID is not existing. We told them the preventive strategies and let them bring their babies. Because one should not forget that vaccination does not protect 100%.

00:43:20

R: they were not listening to us in the previous times. But now they are willing to bring their children for services. There is a big difference by now.

I: how about the motivation of the HEWs?

R: the feedback from the HEWs was not welcoming. They were considered as if they were carrying the COVID with them. That was changed by now and the community is arguing not to take the vaccine; claiming that it is not existing. Because they didn't see one who was a victim of the virus. But now there is a big change in accepting the HEWs and utilizing the services they provided. The HEWs can visit their children for service at home.

I: What are the high-level benefits that are attributable to this support/IR?

R: the first one is reducing the load on the HC. the second one is the timely treatment of children. The service was made up to the standard. The community was saved from unnecessary costs. The HEWs boosted their confidence to provide treatment. It also reduced child death. The use of technology had also been supported a lot. I am afraid not to retract.

00:50:20

I: is it possible to scale up the service to the national level?

R: yes, I don't think one can resist the service.

I: what are the enabling factors to scale up and what limit from scaling up?

R: there might be challenges at a different place. We were challenged to apply the service in the community. There was a shortage of tablets and old tablets were challenging to synchronize. Lack of support and training can also be a challenge.

I: what are enabling factors in Lume woreda, which can be available elsewhere?

R: availability of HC and HP everywhere is enabling factor. Unless the manpower limits the service. The crucial one is the health professionals.

I: How are the activities/efforts embedded in the PHC and woreda routines/work streams?

R: we are giving the service side by side.

I: was it supported by planning?

R: not for 2014 E.C. it was there while planning for under-five children.

I: is the iCCM service planned separately?

R: not in that way.

I: how do you evaluate the plan and outcome of the iCCM service?

R: all the HEWs are submitting their annual plan. Based on the plan, we evaluate their performance.

I: How does the iCCM service included in the woreda base plan?

R: we daily report to woreda by collecting from the HP.

I: Do you have anything think is important to tell us that we have not asked you?

R: there is nothing new I want to add. It is better to provide residents with HEWs. They are not able to provide service after 5 PM. Training should be provided for the health professionals at the HC; just like what was done for the HEWs. Transportation service has to be arranged to facilitate the supervision. The other is supply fulfillment.

End line evaluation of the PSBI implementation research (IR), the qualitative component

I	Identification	
1	Questionnaire ID	Lume-Ejere PHCU-Director- IDI-L&T
2	Area Identification	
3	Name of Woreda/Zone/Region	**Lume Woreda**
4	Name of facility	**PHCU**
5	Name of moderator	
6	Name of a note taker	
7	Date of discussion	**15/06/22**
8	Participant #	**1**
9	Audio File #	**1**
10	Start time:	**_8_:_30_**
11	End time:	**_9_:_38_**
12	Transcriber	

Key: I: interviewer

R: responder

I: Hi, my name is Temam and my colleague is Lalisa. We are from the JSI L10K project. We are here to gather information on the ICCM project for end evaluation. I thank you for your willingness to do this interview. Can you please introduce yourself and let us proceed to the next question?

R: Yes, I am willing to participate in this interview. I am director at Ejere PHCU. It is one of the five PHCU at Lume woreda.

I: Can you please describe the demand generation/SBCC activities you are doing on iCCM/ newborn health? What are the strategies used to raise awareness?

R: we were trained by JSI about the iCCM and started to discuss it together with those who took the training after we came back. We formed a consensus among ourselves. Then the professional who moves to the community started to create the awareness. At any community gathering, about iCCM service and severe neonate diseases were informed. What it means, how they can get service at HP, and the like were communicated. At every 'Afosha', the local money contribution mechanism, the signs of the disease, and where the service can be accessed are told. The diseases in the children like diarrhea, pneumonia, pain, and headaches can be treated within the community. How and where rashes on the children can be treated was told to them. The awareness creation was continuous and still exists.

00:04:01

I: how did the WDA, kebele structures, and schools used for awareness creation?

R: pregnant mothers' conference was mostly used for information dissemination. In addition, WDA meetings, conducted monthly, are also used for awareness creation. Then after, PNC was done for mothers and the neonate. Any health effect seen on the neonate like umbilicus or any other can be treated at a health facility and they should take them health facility. The HEWs were providing awareness regarding this service while they were moving home to home. We were disseminating information in such a way to all of the 8 kebele in our catchment. There is WDA conducted monthly. They discuss health, hygiene, and sanitation. They have money-saving mechanisms for health treatment. This is a well-known and regular program in our PHCU. HEWs, kebele manager and agricultural extension worker is also participating. We use it for disseminating information about the iCCM service. Supervisors also disseminate information while they move to the community for supervision; because it is included as one of the points of supervision. There are one to five linkages in the neighborhood, they are also used for information dissemination. There is a book about the health of the community. They use it for discussion and hence the issue of child health was also discussed in line.

I: did you use school children for information dissemination?

R: yes, we used school clubs in the school for awareness creation. We mostly communicate about communicable diseases like HIV and STI. In addition to that, we tell them about hygiene and child health service.

00:08:40

I: What are some of the biggest challenges with SBCC activities for newborn care?

R: because there are regular meetings in the community, rather than vesting the responsibility only on the HEWs, PHCU professionals should also participate in the awareness creation. Transportation facility is very crucial to providing awareness creation. Motorbike is the most suitable transportation which should be fulfilled. Manpower is challenging and the busy task of the HEWs for other purposes can be a challenge.

I: How do you describe the strength of the support you got from the PHC and the project as well as your efforts to address barriers to delivering iCCM/PSBI during COVID-19?

00:11:16

R: the big activity performed during this support was, the improvement of skill gaps. Especially the training provided for iCCM/CBNC was very important. Starting from assessment up to case management, providing different manuals for the HP, they played a great role, to be honest. Supplies like weight balance are also distributed. They printed brochures for the HEWs to use during awareness creation. I can say the skill gaps with the HEWs were fruitfully solved. They provided tablets with effective chargers and enabled them to easily deliver

health care services. They even promised to provide transportation facilities like a motorbike. For example, they provided us with two motorbikes for HPs.

I: What are the changes happened due to the support provided?

R: problem with knowledge was solved. The gaps that used to be seen among the professionals were solved. iCCM and CBNC were realized at the HC level. Health care service was made to be according to the chart booklet standard. The HP was not using a chart booklet for health care services. They didn't apply the correct disease classification method. But now they started to apply it accurately.

I: can you give us directions on the way the support should be improved?

R: I told you about the support provided by L10K. I suggest the follow-up at the HP should be continuous.

00:16:12

I: Did you get the support system helpful (mentorship and on-site coaching methods, supervision, technical support, PHCU level PRCMM, etc.) to enhance your skills?

R: yes, we were given orientation about collaboration with other HC. They told us to separately conduct a review meeting for each HP. you do with one HP this month and for another the other month. Rather than conducting a general review meeting by collecting all the HEWs from every HP, we started doing that on one HP.

I: how do you compare the mentorship and on-site coaching with the offsite training?

R: the onsite coaching is very important to practically show the activities. You can see the registers, and treatment mechanisms and fulfill the gaps. It is refreshing and recreational when you practically show the gaps. During onsite coaching, the usual service delivery is done for the clients. Then it is used for experience sharing. Starting from counseling by the HEWs, how she is using the chart booklet, how she is taking history and disease classification, and the way she manages the case can be practically shown. This was unforgettable, contrary to the offsite training.

00:21:01

I: does the onsite coaching in the presence of clients increase the acceptance of the HEWs?

R: our measurement for client satisfaction is the number of service seekers. It is increasing.

I: what do you suggest for the improvement of the support?

R: it should be continuous. Supplies should be fulfilled, especially regarding duplication of references.

I: how do you think the onsite coaching improves the skill of HEWs?

R: it had contributed a lot to the improvement of their skills. The iCCM service provision was increased based on the standard.

I: How eCHIS implementation help you with iCCM service delivery?

R: integration of module in the eCHIS was done by this year. The utilization of technology by HEWs was increased. It improved the quality of services. From the quantity-focused service, we shifted to quality service for all. It also improved eCHIS utilization.

I: does it simply case identification?

R: the integrated module was very easy to use. It helps the HEWs as well as the clients. It helped to provide appropriate service for the identified disease. It is guiding by itself.

00:26:25

I: how did it improve appointment, data quality, and retrieval?

R: its benefit is tremendous. It allows you to provide proper treatment. It is also used to give appointments and follow up. It has a reminder for the fixed appointments; so that the HEWs can go to the mother or let the mother come to the HP.

I: How was the introduction of the iCCM/CBNC module into eCHIS affected the iCCM/CBNC service delivery?

R: as I told you before, it helped to provide appropriate diagnosis and hence to provide standard treatment. It is about a life-saving issue. Apart from strengthening the skills of HEWs, it helps the community get appropriate service. It saves time in providing service. Other than using pen and paper, it is now done with a single click. It increases the quality of service.

I: How does it support the referral cases?

R: yes, the module has a lot of services in it. It has lists and addresses of the community in the catchment. It strengthen the relationship between the HC and HP. it allowed communication between the under-five focal person and the HEWs. It has a referral linkage in it. It simplified the data recording system. The one we used to suffer to identify the number of activities performed by the HEWs, is now easily obtained from eCHIS. The PHCU focal person can open the tablet and see how much is done by each HEW. We can also measure the quality of service in line with that. It facilitated the relationship between HP and HC. It enabled an easy shift of the service according to the availability of HEWs. It is guaranteed for data storage.

00:32:15

I: tell me about follow up

R: the module has an action card, and we use it to check the once treated child. If the treatment provided is showing a recovery, we let the child continue taking the medicine. If not, we decide on another action. Including the revision of treatment or sending as a referral case.

I: How is it possible to remind the appointment?

R: it has three colors. The green color is for the upcoming appointment and the red is for the passed appointment. It has the addresses of the child including the contact number. This was not available in the previous manual service.

I: how do you see the client's satisfaction with this service?

00:35:51

R: we have six criteria to measure patient satisfaction. We use a community score card every quarter. It is done by the social parliament and sent to us. I say it is more improved in this service. By providing treatment using technology, the clients realize something better is going on. At the time we measure BP and show them, the community understands something.

I: how do you explain the advantage of data retrieval?

R: iCCM dashboard is not displayed for the HEWs. It is only possible for the focal person or for those who can see it in the central server. Previously, even woreda was not able to see that. It is recently that they are authorized. Hence, the HEWs are not able to see their performance on iCCM. It was thought to be revised, but not done yet.

I: how about its advantage regarding data quality?

R: what is recorded on the tablet is also recorded on the register. They used to send the report without recording it in the register. This time, we see three records, one is registered, and the other is a dashboard and their report. We compare them while evaluating.

I: do you have something to be improved in the module?

R: the dashboard should be possible for the HEWs. In addition, the mechanism by which we can comparatively grade the HPs should be available.

00:40:18

I: Describe the main issues faced by the health system to identify and treat neonatal infections in the community. What are the critical factors affecting the delivery of iCCM to clients?

R: the dilemma about the treatment of children at health post is now solved. The technology helped us from moving with hard copies during home-to-home service. We can use our mobile and provide treatment; so that they can get the medicine from HC. The problem was that the client does not bring a child to HC for accessing the medicine. The HP should follow up and provide a solution for that. For a few HPs, home to a home visit and not being able to follow up is a problem. Being busy with other works or meetings and closing the HP due to that reason can decrease the iCCM service delivery at HP. for the iCCM service to be delivered, the HP should be open every working hour. Because the community doesn't know that it is closed and at the time they see the HP closed, they think as if the service is not available.

I: How does a shortage of supplies limit the service?

R: the shortage of Gentamycin was a problem. Amoxicillin dispenser, injection and tablet service, and ORS should be available. Twenty mg Gentamycin was not available for this service; because EPSA did not avail it. We have only 80 mg. but, because EPSA availed them recently, the problem was solved.

I: How does the shortage of support limit the iCCM service?

00:45:02

R: Support for the HP is remaining; except for the HC. Last time, JSI was providing coaching support for 3 selected HPs. We were told to report any supply problems and we did it; especially for medicine and treatment devices. They gave us a weighting balance and Gentamycin 20mg. Even though it is not continuous, they supported us.

I: How does the shortage of support provision affected the service?

R: previously it has had a great impact on the iCCM service. Not identifying the disease correctly, not being able to manage cases properly, and not correctly prescribing medicine was the problem.

I: is this before the training?

R: yes. After the training they started using the module and chart booklet; hence there was no such kind of problems. Except for the discontinuity of support, they are providing it.

00:47:54

I: How does the low demand from the community affected the iCCM service?

R: yes their low awareness had decreased their service delivery; because they don't think they can provide treatment for the children at the HP level.

I: how about security issues?

R: there is no security problem here.

I: how about COVID?

R: the impact of COVID was very serious.

I: How do you think the COVID-19 pandemic and/or COVID-19 response measures affect the uptake and/or delivery of iCCM services?

00:49:16

R: the community was afraid of the pandemic because they think they are contaminated by the virus if they come to the HC. That had decreased our service. They were also not able to gather during the meeting for awareness creation. So does the awareness creation at school. This affected the awareness creation and hence our service.

I: what do the community do with their sick babies?

R: they started traditional medicine. They started using the leaves of plants for treatment. We were seeing this practically. They preferred it over the COVID contamination while taking health care services at a health facility, they think. This time, because it is not serious, they are not worrying about COVID.

I: can you tell me how the worries are solved?

00:51:23

R: it is because the real impact on individuals was not observed in the community. They used to see it in the media; otherwise, they saw no one who was a victim of COVID. Others politicize the disease; by saying it is the government that brought COVID. They claim it is false, that they didn't see it practically. You disconnected us from each other; even though you thought us to wash hands with soap.

I: you are saying the previous fear is resolved by now?

R: yes.

I: is there a change after COVID adaptation?

R: yes, the patient flow was increased. We vaccinate at least 40 children every Monday. At least 20-30 children get treatment at HC every day. I clerked about 93 adults yesterday. After the fear was over, the service was increased very much.

I: What was the motivation from the HEWs look like; when compared with the time the pandemic was serious?

00:54:37

R: they were using transportation and due to the fear of COVID, they preferred to stay home. The cost of transportation was very high at the time. But now, because everything is settled, they are performing well. Supportive supervision for HEWs was not possible at the time. Now, our relationship, including conducting meetings has become possible. The review meeting was also reinitiated.

I: How does community engagement change over time during the pandemic?

R: the community, after getting the awareness, they are disseminating the information to others that the service is available and safe. They tell them using examples of those who got treatment.

I: What were the strategies you used to reinitiate the service after the lockdown?

R: they broke the fear because they didn't see any harm from the disease. They were listening to the media about the disease and that helped to let them come to the health care facility. At the time they come here, the health professionals were providing awareness. But the community, because they didn't see anyone harmed, they started to come for the service. This time, no one refuse to come here due to the fear of COVID.

I: What are the high-level benefits that are attributable to this support/IR?

00:58:32

R: awareness was created and hence the number of clients were increased. It increased the quality of service. Facilitated the linkage. The poor awareness about iCCM/CBNC was solved. It enabled to have reliable data. The use of technology and skill of the HEWs was improved. It also facilitated the reading for HEWs.

I: Please explain to us the feasibility of this support/ IR for national scale-up?

R: the iCCM service has great advantages. It should not be undermined. Scaling up is very important; even to the level of developed countries, let alone Ethiopia.

I: what are the enabling factors for scale-up?

R: the module has everything in it and is very interesting. It is standardized to the continental and international levels. Hence it is similar everywhere. The module contains beyond iCCM. Scaling up helps the professionals, and the clients and solve the problem with data management.

I: what could be an obstacle to scaling up?

R: if the project stops until the government takeover it could retract. Until the HC owns the activity and overtakes the service, it could stack. Participation of stakeholders and availability of tablets is required.

01:02:04

I: How are the activities/efforts embedded in the PHC and woreda routines/work streams?

R: we have everything at hand. Hence we can continue the service. we can share our experience with those in need.

I: What was planning for iCCM look like?

R: iCCM is one of the family health issues. We can plan for it accordingly.

I: Do you have anything think is important to tell us that we have not asked you?

R: a lot of issues were raised. I thank you for coming to us and consulting us. It is for the benefit of our community.

I: thank you for your patience and valuable information.

End line evaluation of the PSBI implementation research (IR), the qualitative component

I	Identification	
1	Questionnaire ID	Lume-Ejere PHCU-Tiliti Garbi kebele-HEW-IDI-L&T
2	Area Identification	
3	Name of Woreda/Zone/Region	**Lume Woreda**
4	Name of facility	**PHCU**
5	Name of moderator	
6	Name of a note taker	
7	Date of discussion	**16/06/22**
8	Participant #	**1**
9	Audio File #	**1**
10	Start time:	**_2_:_30_**
11	End time:	**_3_:_27_**
12	Transcriber	

Key: I: interviewer

R: responder

I: Hi, my name is Temam and my colleague is Lalisa. We are from the JSI L10K project. We are here to gather information on the ICCM project for end evaluation. I thank you for your willingness to do this interview. Can you please introduce yourself and let us proceed to the next question?

R: Yes, I am willing to participate in this interview. I am Tiliti Garbi kebele HEW under Ejere PHCU.

I: Can you please describe the demand generation/SBCC activities you are doing on iCCM/ newborn health? What are the strategies used to raise awareness?

R: regarding awareness creation, we oriented the 13 groups under the HP that we can treat children less than five years of age. We told them to disseminate the information among their group members. In addition to that, we used different platforms and 'Afosha', the local women's money contribution system, repeatedly. We informed them to come for the iCCM service. we tell them about the children who were treated in the HP; as an example.

I: how did the WDA and the community participate in the awareness creation?

00:02:07

R: the WDA were organized since 2005 E.C. they are not only engaged in the iCCM but all the 18 packages delivered by the HEW. Even though iCCM is not one of the packages, because it is our duty, we provide the WDA

activities. They create awareness and bring lists of children. The advice mothers of the children to take their babies to the HP at any time they feel sick.

I: did you participate in the religious leaders or elders in the community?

R: yes, even though we are not able to provide the information by ourselves at a religious places, we made the religious leaders disseminate the information. We also used Aba Gada and elders in the community. We tell them to disseminate the information at ‘Afosha’ and other gatherings.

I: Is there any challenge with SBCC activities for newborn care?

R: yes, regarding medicine there is a challenge. It is sometimes not available. For example, at the time we treat diarrhea, we might miss Zinc. It is not possible to provide only ORS. We refer the child to HC due to this. We also lack Gentamycin for under two months of children treatment, currently. This is another serious challenge. At the time children come to us; we are forced to send them to HC.

I: How does this affect the awareness creation?

R: we told the community to bring their mild and severely diseased children to us. Because we are not able to treat these children, we send them to HC. But the community prefers HP over the HC. After we told them that we can treat and lack medicine for treatment, it is an obstacle. We were supposed to send only those above the HP level.

00:04:56

I: How do you describe the strength of the support you got from the PHC and the project as well as your efforts to address barriers to delivering iCCM/PSBI during COVID-19?

R: above all, the new strategy, assisted by technology like tablets delivered from the project, is very interesting. After registration by recording the name, and age, it performs identification. Then it guides you to the treatment and prescription by itself. It also has a new chart booklet that is also very interesting.

I: How does the renewed chart booklet help you?

R: at the time we visit a mother after delivery at home, we used to call it PNC. But now, because we are also checking the neonate, it is called assessment. There are a lot of issues revised in the chart booklet.

00:06:27

I: what additional benefits did you get from the support?

R: in addition to what I said before, at the time mother bypassed this HP and accessed treatment at HC, and the feedback about treatment comes back to us. Including names and the kind of treatment. The other issue is that,

because we are loaded with activities, at the time the appointment approaches, it reminds us of red color. Hence it can necessarily remind us, even though we are busy with other activities.

00:07:32

I: do you suggest something to improve, regarding the support provision?

R: supportive supervision is loose and irregular, most of the time. We are only dependent on our endeavor. No one comes to us for support regarding under-five children. I saw them only once or twice when they come and provide supportive supervision from the woreda level. I prefer if the under-five focal person at HC, who was trained on it, shall come and support. We might commit a mistake. That can increase our performance and skill. Because the electronic applications need repeated practice, I suggest the project provide us refreshment training or review meetings; at least quarterly. Because it helps us to share the experience.

I: is there no review meeting conducted?

R: no review meeting was conducted. It was done only last September.

I: Did you get the support system helpful (mentorship and on-site coaching methods, supervision, technical support, PHCU level PRCMM, etc.) to enhance your skills.

R: mentorship was provided only once, in September, just after the review meeting. Then after everything stopped. There was no supervision.

I: How does that affect your skill?

R: it doesn't affect as such. We might forget some of them. At the time they come for supervision, there might be measurable points that we can remember in our way of reporting to our supervisors. It also gives a chance for experience sharing. Mentorship should be conducted at least quarterly.

00:10:11

I: there was no on-site coaching provided for you?

R: not at all.

I: how about off-site training?

R: not at all

R: which one do you prefer on-site coaching and off-site training?

R: I prefer if the iCCM is provided in the form of training. It is better to gather us together, use our tablets individually and see the treatment practically. During the mentorship, they see registers and evaluate our performance. That is not preferable over training. Because training allows to practice.

I: did you take iCCM training?

R: yes, I took it.

I: so you are preferring off-site training over mentorship?

R: yes, because the one providing mentorship covers a lot of kebele. It is tiresome and boring. But during the training, because it is pre-planed, you can see things stepwise. During the mentorship, they might not cover all of the HP. it is tiresome due to walking long distances. The contact with a variety of individuals and interviews might be tiresome and boring. It is not necessary; unlike training. During the training, the trainer gives the contents as planned.

00:12:50

I: How eCHIS implementation help you with iCCM service delivery?

R: everything is found on the tablet. At the time you start treating a child, when you want to measure weight, it tells you whether the weight is adequate or not. So does the temperature. During identification, it can show it easily. What kind of disease a child is affected with can be easily identified. At the time identification is complete, it can show what kind of medicine should be provided, including its dose. At the time a referral decision is required, it pop-ups in the referral writing place. It also shows the medicine should be provided before referral. The other advantage is that it reminds the appointment. At the time appointment is reached, it shows by red color. If the appointment is yet to come, it shows by a yellow card. There are about four colors to remind appointment. At the time appointment is passed, it shows the red color and the child should be visited. The status of the child should be known by any means. The integration of the chart booklet with the eCHIS is also interesting. At the time we send the referral case to the HC, the acceptance of the child at the HC comes back as feedback to us. The information about the treatment, medicine provided, and time is sent to us as feedback.

00:15:10

I: how about retrieving data or data quality?

R: it is not 100% quality. At the time treatment is provided. The child has to be registered in the under-five register and on the tablet. The one under two months is registered in under two-month register and do so for the other above two months. The other is registration on the cards, but we didn't use them. That could compromise the quality. Not recorded in the tally sheet is also another shortcoming.

I: how do you trace defaulters?

R: the tablet shows red and we visit the child at home. Then we see the status of the child and fill in the tablet accordingly. then we inform the WDA to follow the child and inform us.

I: How was the introduction of the iCCM/CBNC module into eCHIS affected the iCCM/CBNC service delivery?

R: it has helped us a lot for iCCM service. At the time we want to conduct a home-to-home visit, we don't need the chart booklet in hard copy. It is found in the tablet and at the time we start treatment, everything is found in it and guides us very well. For example, if I want to measure breathing, it can do that and show me the ranges with the meaning. Hence, it is easy to use and treat. We used to carry the chart booklet with us; not it is found on the tablet. We only carry MOAC and medicines.

I: what are its advantages in identification and registration of sick newborns; clinical and referral decision support; follow-up of sick child treatment and/or defaulter tracing?

00:18:55

R: at the time I record information about the child, it shows the identification. After identification, it decides on a referral case or medicine prescription. It tells how the first dose of medicine should be ordered. It also shows the duration in which the child should be sent. It shows the date of appointment and two days for pneumonia. Then you complete the treatment in such a way. The appointment is silver at the beginning; then turns green. It shows yellow when the appointment is reached and red when it is passed. Red means it is an emergency for searching the child. Regarding referral cases, it asks for the availability of necessary medicine. If you say no, it orders you to provide the first dose of the medicine and allows you to send it as a referral. Otherwise, it doesn't decide whether to send or not by itself. But you can treat the child as long as you have the necessary things.

I: is sending or not sending as a referral dependent on the availability of medicine?

R: one is the availability of medicine and the other is the complexity of the disease. Very severe diseases are not treated with us.

I: who decides the seventy of the disease, is that you or the tablet?>

R: it is the tablet.

I: how is it advantageous regarding client satisfaction?

R: the community is satisfied very. Because they don't like moving long distances; they are delighted by the service provided at HP. they prefer HP over the HC. At the time they come to the HP and have their babies cured by the

service provided, they are very happy. Sometimes they exaggeratedly talk about the HP over the HC. This had helped us a lot in the success of our service.

00:23:36

I: do you suggest something should be improved?

R: I see only the advantages.

I: Describe the main issues faced by the health system to identify and treat neonatal infections in the community. What are the critical factors affecting the delivery of iCCM to clients?

R: there is no significant challenge that we faced in the community; while providing the service. I have never faced any challenge, personally. But sometimes remembering and carrying the medicine with us is sometimes a challenge. But there are no significant critics that were forwarded from the community; that can be an obstacle for the iCCM service. At the time we encountered a sick baby and provide treatment, we might have no medicine at hand. Then we tell them to come to the HP and get the medicine. They come and take it.

I: How does your competence become a challenge in providing the service?

R: there is not at all

I: how about the shortage of supplies?

R: we didn't have a weighting scale but we were provided a bucket with a spring balance, recently. Even though it is not significant, sometimes the medicine supply is short. I don't think it is due to the HC, rather it is from the suppliers. ORS and Zinc are repeatedly lacking. Gentamycin supply shortage did not get a solution up to now.

00:27:11

I: how about lack of support?

R: there is no significant challenge that happened due to lack of support from the project or any other body. Even though the project reinitiated this service, we used to provide it; even though it was irregular. It is because the service was decreasing, that the training was provided and the service was restarted. We are not new to the service and so does the community. Hence, there is no challenge.

I: does the low demand from the community affected the service?

R: no. the community was accessing this service. Because we face a shortage of medicine, at the time we send them to HP after diagnosis, they started to bypass us. But after we renewed the training, they came back.

I: how about a security issue?

R: there is no single security problem.

I: how about COVID?

00:28:37

R: at the time of COVID and then after, there was no time when the service was interrupted; nor does it change anything. The existing service during the pandemic is still there after that. It doesn't affect the service anyway.

I: The pandemic has affected the livelihood; how do the COVID-19 pandemic and/or COVID-19 response measures affect the uptake and/or delivery of iCCM services?

R: as I told you before, there was no time at which we closed the HP due to the COVID. We were providing all kinds of services at the time. The only service interrupted was the pregnant women's conference. Even that was done because there might be contact between pregnant mothers and they are sensitive. We told them at the last conference and exchanged our contact numbers to follow them up individually.

I: Has COVID-19 affected your daily routines; your work on newborns; the community in terms of livelihood and vulnerability for newborn care-seeking?

R: what happened during the pandemic was, that they were not bringing their neonate to HP. we are the one who goes to them for treatment. Otherwise, those above 45 days were coming for vaccination service, FP, and other services.

00:31:18

I: what does the pandemic look like by now?

R: we know the people who were sick, admitted, or died due to the disease. We are the ones who fear more than the community. Otherwise, the community almost forgot about COVID. They are saying you are the one who speaks about COVID; otherwise there is no COVID. Maybe it is because it doesn't affect them significantly. Now seeing the one affected by COVID or who died due to COVID could be the reason for the community not to fear.

I: was it not informed in the media?

R: it might be because they expected to see the diseased one over the simple information.

I: what were the COVID-19 adaptive iCCM implementation strategies?

R: there was no significant difference regarding the support provided. But the linkage between the HP and HC was interrupted. We were supported by the director of Ejere PHCU. The kebele administration was not collaborating with us the team. But now it is changed and supportive supervision was provided every week. Maybe because of

busy tasks at the HC, the health professionals were not providing supportive supervision by the time of the COVID pandemic.

I: is there no change about accessing the health facility by the community?

R: they were coming to health facilities by the time. Even they prefer HP over HC by the time. At the time they were attacked by cough, they didn't want to go to HC, due to the fear of being admitted as a COVID suspect. They said to let you do your best for me; otherwise, they refuse to go to HC.

00:35:22

I: how was the motivation of the HEWs at the time; as compared to the present?

R: it is almost the same for me. At that time, we aimed to save lives and so do at present. We aim to accomplish the responsibility vested on us. Because we focus on prevention than cure. We were supporting them on prevention mechanisms of any kind of disease. We were busy more by the time than the present. Because we were expected to move home to home for awareness creation. We were challenged a lot. We limited the number of money collectors on 'Idir', the traditional money-saving mechanism. At the time five and more people are found sitting together, and we were blamed if we didn't give them awareness. Even though it was challenging, we were successful in creating awareness and saving the community.

00:36:46

I: how about community engagement and awareness creation?

R: there was a challenge in every direction. The community was not comfortable letting us touch their children. Even we also suspected ourselves. It is only because they don't have options than the HP that they came to us. Rather than moving long-distance, they prefer us. Otherwise, they don't like governmental health facilities. They prefer a private one. Because they fear not being isolated at the HC, in case cough is suspected. They chose us irrespective of the fear of the pandemic. Hence, there was no time that our service was interrupted and the community didn't bring their children. There was no child affected by diseases due to the fear of COVID.

I: What are the high-level benefits that are attributable to this support/IR?

R: it saved the time we used to write on paper. It simplified our activities. It assisted follow-up at the HP and the one from HC. Because it shows colored reminders.

I: how about the benefit regarding child health?

00:40:13

R: there was no treatment effort we made that became unsuccessful. The majority, more than 100 children, I treated after the training provided were successfully cured. Their feedback was motivating; there was no single complaint regarding the service. At the time I see the baby cured, it initiates me for more. I like it if adequate medicine is available and serves more children. The only occasion heartbreaking was that 28 day old neonate was passed due to lack of Gentamycin. She was attacked by severe disease; had I gotten gentamycin, I could have given her at least the first dose and saved her life. Otherwise, all the services I used to provide were very successful. At the time I was told about the child, I went to her home and took her to HC. After she got treatment at the HC and went back home, she died before completing the prescribed medicine. Beyond that, I can say 99% of the services were accomplished.

I: How does the project support help you to boost your acceptance in the community?

R: we are living in the community. We have a close relationship and are always showing them a well-coming face. That kind of service provided in a good relationship attracted them a lot. They are not comfortable with the bureaucracy at HC. They simply come here, and we sit together and discuss the issue and provide service.

I: does the availability of service with the tablet increase your acceptance in the community?

R: I cannot say it is increased or decreased. It is as usual for me. Before and after the tablet, our acceptance in the community is almost similar. Sometimes, they say, you are using technology and your service is becoming advanced.

00:43:29

R: they are happy when you take fingerprints of children above two years. That can make them feel different and happy. But that doesn’t change the number of individuals accessing the service. Those who used to access the service are still coming. No one is coming outside of the catchment.

I: it was said using the tablet increased the satisfaction of the community over reading hard copies for treatment. Does this holds the same with you?

R: as I told you before, the tablet saves time. We used to record all information about the child and go to identification using the chart booklet. Now the tablet has everything in it. Hence they are satisfied with the table service.

I: Please explain to us the feasibility of this support/ IR for national scale-up?

00:45:01

R: yes it is possible to scale up. The community, especially those in the countryside, prefer to come to HP over the HC. There are a lot of poor people. Services HC, starting from record room up to final treatment, is by payment. But every service is free here. At the time they get free service and their babies are cured, they are delighted more. Children can be harmed by the time they take to prepare themselves for services provided at the HC. Due to lack of transportation or money, until they make themselves ready for the service provided at other government or private health facilities, the child might be harmed. At the time the HP is applied to the country level, home-to-home health care service can be possible. Because the software simplified everything, service provision can be simple. At the time it is beyond our level, we can provide advice and send it to the higher level; by convincing for the reason.

I: what are other factors that assist to scale up?

R: the presence of HEWs at the national level is one reason. It is possible to capacitate them and provide the service to save the lives of children.

00:49:28

I: what could be an obstacle to scaling at the national level?

R: I don’t think there is an obstacle to scaling up. The rural community prefers HP over HC. The HEWs should have a residence near the HP just to provide timely service. And all the requirements for her have to be fulfilled. But that cannot be done by this project. Lack of access to clean drinking water can be an obstacle. Because ORS is diluted by clean water.

I: How are the activities/efforts embedded in the PHC and woreda routines/work streams?

R: even though it is not adequate, we are doing our best. Most of the time, what we did and what was recorded are not balancing. What we did is greater than what we recorded. The other is that we are not able to retrieve the information of children who got treatment. It should be corrected.

I: what do your woreda, HC, and HP collaborative planning look like?

00:53:17

R: during our review meeting every month at the woreda level, iCCM service is also evaluated. We plan and evaluate our performance accordingly.

I: How was the planning for iCCM?

R: our planning for diarrhea and iCCM is done by conversion factor targeting under-five children out of the total population. That is how we plan and report our performance monthly. Our performance is up and down. Three is a

time at which pneumonia increases and other times diarrhea increases. There is a time at which it is 100% and other times less than 50%.

I: are there reasons that can retract the iCCM service?

R: no reason can retract the service. The only reason could be the lack of attention paid to iCCM service. Most of the time, due to lack of attention, we prefer to send cases as referral cases.

I: Do you have anything think is important to tell us that we have not asked you?

R: regarding iCCM service, lack of at least a quarterly review meeting should be provided. We were provided about 4 days of training. We told them it was not adequate, but responded it is only for the pilot study. The detailed training will be provided, they promised. But full package training was not given to us for a year long. I suggest that should be fulfilled. Retrieving data of the children treated should be made possible. For the referral feedback, the action card, once seen, is not possible to be deleted. At the time the child is accepted and confirmed, the action card remains. That should be deleted.

End line evaluation of the PSBI implementation research (IR), the qualitative component

I	Identification	
1	Questionnaire ID	Lume-Ejere PHCU-Vice director- IDI-L&T
2	Area Identification	
3	Name of Woreda/Zone/Region	**Lume Woreda**
4	Name of facility	**PHCU**
5	Name of moderator	
6	Name of a note taker	
7	Date of discussion	**15/06/22**
8	Participant #	**1**
9	Audio File #	**1**
10	Start time:	**_2_:_30_**
11	End time:	**_3_:_27_**
12	Transcriber	

Key: I: interviewer

R: responder

I: Hi, my name is Temam and my colleague is Lalisa. We are from the JSI L10K project. We are here to gather information on the ICCM project for end evaluation. I thank you for your willingness to do this interview. Can you please introduce yourself and let us proceed to the next question?

R: Yes, I am willing to participate in this interview. I am the vice director and HEW coordinator at Ejere PHCU.

I: Can you please describe the demand generation/SBCC activities you are doing on iCCM/ newborn health? What are the strategies used to raise awareness?

R: I think we did a great job to create awareness in our PHCU catchment. We used 'Afosha', local money contribution by women, different platforms, and pregnant women conference for creating awareness. A variety of works were done on these alternatives. Timely access to health care services was not common in our community. To change this attitude, the HEWs and health professionals of our PHCU played a great role. In addition to this, while we were rounding for the ODF activity, we provided awareness by going home to home.

I: How did you use the WDA and available administrative structures?

R: we made the HEWs gather the leaders of WDA to provide information about iCCM.

I: What are some of the biggest challenges with SBCC activities for newborn care?

00:05:52

R: Because it is about behavioral change, it challenges to some extent. Because the majority of our community is not educated, it is hard to bring behavioral change. Let alone the community, it is hard to make a behavioral change in an individual. It needs patience and time to bring behavioral change. Walking long distances and whether the condition can be challenging. There might be resistance from the community.

I: How do you describe the strength of the support you got from the PHC and the project as well as your efforts to address barriers to delivering iCCM/PSBI during COVID-19?

R: as to my understanding it has a great advantage as compared to the previous one. By including the iCCM/CBNC in the checklist, we provided support for the HEWs. The number of children who are getting treatment at the HP is far greater than the previous one. It is increasing from time to time. It is supported by software and a separate tablet. The tablet guides the treatment steps. It helped to save data and act as a base for assessment and sequential investigation.

I: what do you suggest to improve the support?

R: there is the diagnosis that was not included in the tablet; I suggest including them.

I: What are the diagnoses that are not included?

R: for example, dermatology was not included. I suggest all the under-five diseases should be included in it. I think the guideline is developed by GP and medical doctors. I was the guideline and it orders only Amoxicillin repeatedly. I think they are the first-line drugs. It should be followed by second and third-line drugs if the child does not recover. It takes a process for the child to be free of the disease. They need patience, regardless of the pain from the disease. I suggest moving to the second-line drug for the baby to feel better immediately.

I: Did you get the support system helpful (mentorship and on-site coaching methods, supervision, technical support, PHCU level PRCMM, etc.) to enhance your skills?

00:15:15

R: the HEWs were not giving treatment regarding iCCM. They were restricted only to the 18 HEW packages. But after iCCM/CBNC, they started providing treatment. They got training and mentorship support to develop their skill.

I: How does the onsite coaching and mentorship assist the skill development over the traditional off-site training?

R: I can say mentorship and on-site coaching face to face is the most intelligent way of skill development; as compared to the offsite training. At the time training is provided collectively, one might or might not understand the contents. Individual face-to-face coaching can give the chance for identifying the knowledge gaps. It boosts confidence to 100%.

I: how about a review meeting?

R: we conduct review meetings monthly. There was no time when the iCCM/CBNC and eCHIS issues were not raised. It gives chance for gathering all the HEWs for discussion. Issues are identified and solutions are provided. Experience sharing is also possible.

I: How does that contribute to the acceptance from the community side?

R: the community is coming closer by now. They are started to consult the HEWs for any issues. They are also bringing their children to the health facility. Trust in the HEWs is increased; even though there is a challenge.

00:21:28

I: what kind of challenge?

R: Ejere catchment community is promising, but not applying yet.

I: what do you think is the reason behind it?

R: the mistrust is still there. Change takes time.

I: what do you suggest for mentorship and onsite coaching support to improve them more?

R: I suggest behavioral change should take place at the national level. If that has happened, I think everything becomes smooth. I also suggest the PO medication for injection. Because second-generation HEWs are coming to provide this service at the kebele level.

I: How eCHIS implementation help you with iCCM service delivery?

R: eCHIS is the best; out of the HIS available. It identifies, treats, and manages every case of iCCM by itself. It has a good feature together with the CBNC module.

I: How does it identify and manage the cases?

R: you ask for the problem with the child, for example, under two years old. Then it could be watery diarrhea, pneumonia, malaria, or malnutrition. You ask the mother and record on the tablet. Finally, it has colors by which it tells the decision. Red is for referral cases. That is how it goes on.

I: how about data management?

R: the biggest challenge with eCHIS is that it doesn't show the data about how many treatments were conducted. For example, after EPI or pneumonia treatment, the data is found only in registers. It cannot be accessible from eCHIS.

I: how about data quality?

R: paperwork can be affected by a false report. ECHIS tells only what was done. it is of the best quality. It also contributes to quality reports.

00:27:30

I: how about an appointment and default tracing?

R: it gives an appointment by itself. The maximum appointment data is 14 days. It shows by the color of the appointments. It turns red when the appointment approaches. It has red, yellow, green and blue colors. It turns red when the appointment is passed. That is good to remind the appointment.

I how advantageous is it as compared to the previous one?

R: in the previous one, it could be forgotten; unless the mother comes by herself.

I: what do you think was changed in the eCHIS for iCCM service as compared to the previous one?

R: it has brought behavioral change. It has contributed a lot to data quality. It has also improved the knowledge of HEWs.

I: what kind of behavioral change has happened in the community?

R: It increased the trust of the HEWs. The technology facilitated service delivery and the assistance from the tablet increased the satisfaction on the client side.

00:30:44

I: you told me that retrieving data is a problem, can you add more that should be improved regarding eCHIS?

R: it is getting improved gradually. I hope all will be solved in such a way.

I: How was the introduction of the iCCM/CBNC module into eCHIS affected the iCCM/CBNC service delivery?

R: after delivery, PNC follow-up is done by the HEWs, assisted by a tablet. The eCHIS facilitates feedback about the service provided. That is how they know PNC service. They also use a phone to inform about a mother who went home after delivery.

I: how about clinical and referral management?

R: referral cases are those which are complex or randomly seen by the PHCU professionals. The case is sent to under five OPD as a referral.

I: how about feedback after referral?

R: the feedback is also done using eCHIS. For example, for a mother on ANC1, it shows the services she got at MCH like VDR, HIV test, hemoglobin, hepatitis, TT, and iron-folic services. After BP and other necessary steps are complete, at the time you click on synchronize, it sends to HEWs.

00:35:56

I: is it possible to measure patient satisfaction with the tablet?

R: yes it is possible to measure. We used to send written papers to HC, for referral cases. But now, with a single click, you can synchronize it. That mother can get the service at any time she wanted. That is one satisfaction, for me.

I: how do you compare the integrated CBNC module with the previous one?

R: customer satisfaction is realized. The second one is the use of technology. And the third one is that it contributed a lot to knowledge. The guideline was standardized. The tablet assisted in closely refer the manual for updates.

I: is it possible to improve the service even more than this?

R: I like it if it is improved. Few tablets are not easy to synchronize with HP themselves. It takes 30-60 minutes to synchronize.

00:39:13

I: Describe the main issues faced by the health system to identify and treat neonatal infections in the community. What are the critical factors affecting the **delivery of iCCM** to clients?

R: I don’t think there is no challenge in this regard. It is possible to improve the skill gaps through training and review meetings.

I: How does the shortage of supplies affect the service?

R: we are providing Amoxa, Gentamycin, ORS, Zinc, and TTC. We faced a shortage of Zinc and ORS. But because we are buying them from the health care financing, there is no significant challenge related to that.

I: how about a shortage of support?

R: we are collaborating with woreda and JSI for supportive supervision. There is no significant challenge related to a shortage of support.

I: what about low demand from the community?

R: it is found in a good condition; hence there is no significant challenge related to that.

I: what about insecurity?

R: there is no security problem here.

I: how about COVID?

R: it is not only the problem of this area, it is a national and international problem. I was not around during the pandemic. I came here five months after the pandemic is gone.

I: is the pandemic not existing by this time?

R: I am comparing when it was serious and the current time. There was big chaos by that time. People were afraid to come closer. People were afraid of health professionals; because they are prone to infection.

00:43:47

I: hence this was hindering them from accessing the health care service?

R: yes, they suspect to be infected, if they go to the health facility.

I: what happened to the iCCM service at that time?

R: the community was providing traditional treatments for their children. Except for a few of them, who decide to bring their children to the health facility, not letting their child die at home due to lack of treatment.

I: then how did they start coming to the service?

R: it is by the awareness created after that. Media, HEWs, and administrative bodies had created the awareness. They were told while they come for vaccination.

I: what strategies did you use to resolve the created gap?

R: we started health education; this was the biggest intervention. They used any platforms to disseminate the information. They also moved home to home and used influential people in the community.

I: how did the motivation of HEWs initiate?

R: I think you should not ask me this question; because I was not around at the time. I was one of the COVID victims.

I: How was the feeling?

R: I don't wish it even for my enemy. It kills psychologically. I was admitted for one month.

I: How was the service delivery improved then after?

R: we discussed with the HEWs and enabled the community to use masks and sanitizer while accessing service. we used religious leaders and elders to create awareness in the community. That is how we initiated the service delivery. I can say iCCM/CBNC is found on a good status; from being stacked during the pandemic. We also used WDA and pregnant women conferences so that they can bring their babies to the health facility.

00:49:58

I: What are the high-level benefits that are attributable to this support/IR?

R: it had greatly contributed to data quality and knowledge. It eased the treatment and made it paper-free. It is also made to reduce the cost of treatment. It also reduced the death of neonates.

I: How does it reduce the death of neonates?

R: it facilitates the PNC service by synchronizing it with the HP immediately after delivery. That is how maternal and neonate death is reduced.

I: How does it improve the quality of service?

R: there is no argument with the HEWs on the reported activities; just like what was done before. There might be an argument on whether the report was sent or not sent. This technology is not deceived or deleted.

I: Please explain to us the feasibility of this support/ IR for national scale-up?

R: yes it is possible by experience sharing from us. There are similar guidelines and treatment standards at the national level. The HC center found here uses similar technology to the one in Debre Birhan or elsewhere. So do

the HPs. But the service was dependent on once knowledge in the previous times. It solves that kind of problem and standardizes the service.

I: what could be an obstacle to scaling up?

R: one could be a security issue and the other is inflation.

I: How does the electric power and network?

R: it is unquestionable. It is a problem everywhere.

00:57:12

I: How are the activities/efforts embedded in the PHC and woreda routines/work streams?

R: the annual iCCM service is included in the plan; both at HP and HC levels. Under-five Pneumonia, a very severe disease of a neonate, sepsis, and local bacteria infection is included in the plan.

I: do you think the service could retract in the absence of this project?

R: no

I: why?

R: the base was made by creating awareness in the community. They know that they can come to the HP or HC to get service. Maybe a shortage of medicine can be an obstacle.

I: Do you have anything think is important to tell us that we have not asked you?

R: it is better to correct the problem with data retrieval. It is better to produce a dashboard for the HC and HP level. It is better to decide and compare the performance of HEWs. It is also better to provide incentives for the most performing professionals.

I: thank you for your time and valuable information.

End line evaluation of the PSBI implementation research (IR), the qualitative component

I	Identification	
1	Questionnaire ID	Lume-Tade-Bola Buta Kebele-HEW- IDI
2	Area Identification	
3	Name of Woreda/Zone/Region	**Lume Woreda**
4	Name of facility	**HP**
5	Name of moderator	
6	Name of a note taker	
7	Date of discussion	**09/06/22**
8	Participant #	**1**
9	Audio File #	**1**
10	Start time:	**_8_:_30_**
11	End time:	**_10_:_38_**
12	Transcriber	

Key: I: interviewer
R: responder

I: Hi, my name is Temam and my colleague is Lalisa. We are from the JSI L10K project. We are here to gather information on the ICCM project for end evaluation. I thank you for your willingness to do this interview. Can you please introduce yourself and let us proceed to the next question?

R: I am HEW at Bola Buta HP

I: Can you please describe the demand generation/SBCC activities you are doing on iCCM/ newborn health?

R: regarding iCCM/CBNC, after we took the training, we started to treat neonates, infants, and mothers on PNC. At the time the neonate, as well as infants, come to our HP, we tell the mothers about diarrhea, pneumonia, very severe diseases, and local bacterial infections. We also use religious leaders, kebele administration, and using our good relationship with the HC, we used to create awareness. We also provide awareness by going home to home. Generally, before the tablet and after that, we were doing awareness creation; even though it has some shortcomings.

00:04:00

I: What are the strategies used to raise awareness?

R: we are doing it depending on the CBNC module. We take the WDA together with us while moving into the community. At home, we use any favorable language understandable to them. We tried to obey their traditional

practices and culture. For example, a given mother can feel shy; we then try to make her free, before starting the service. We also used 'Afosha', the local women's money-saving mechanism. We also used the available platforms and school students to disseminate the information. We used pictures to show them the signs of diseases in the children.

I: How does the community participate in the awareness creation?

00:05:50

R: we used the pregnant women conference, which is conducted once a month. We strongly disseminated the information. At school, we told them to inform their parents, that when such a kind of sign is observed on the children, let them come to us. Inform the pregnant women to come to us. We inform the husband; because mothers sometimes use traditional medicine and make the disease complicated.

I: What are some of the biggest challenges with SBCC activities for newborn care?

R: there is no significant challenge; but sometimes, mothers, due to lack of awareness, put something on their umbilicus. That might lead to local bacterial infection. The other is, that at the time a child feels sick, they bypass and go to another health care facility. The other challenge is the lack of adequate materials we take with us during home visits. For example, we might not have amoxicillin to give the babies. There was hesitation in our skill to treat the children. The other challenge is that HP is not balanced with the community we are serving. At the time we convinced the mothers to use the service, due to the challenge of topography and distance, their children may die before they reach us or other health facilities.

00:10:22

I: How did you try to convince the community and get rid of the hesitation?

R: the first one is by fulfilling the necessary medicine for the HC. The other one is by strongly paying attention to our ability of treatment, guided by the module. At the time a mother sees her baby cured, she disseminates the information to the community and lets them come. She tell them that I went there and helped me with the treatment of my baby. If they see such kind of signs on their baby, she tells them to go to the HP. Making the HP open all the time can increase the satisfaction in the community.

00:11:39

I: how did the WDA help you in the awareness creation?

R: the WDA and one to 10 linkage are interrupted. It is challenging to gather them for discussion. We used the pregnant mothers' forum for information dissemination. We give them our numbers and plan together on the issues we have to do together. Regarding the packages, including the child's health. We use them to disseminate

information about iCCM. The big issue is to work closely together with them. We feel the harm of a given child just like one of our children. So harms a mother. We feel as if she is our mother or sister.

I: How do you describe the strength of the support you got from the PHC and the project as well as your efforts to address barriers to delivering iCCM/PSBI during COVID-19?

R: the support we were provided was at least feedback based on our iCCM service. That is already documented. At the time they come for supervision, they check every critical issue and provide us with their feedback. They reminded us of a lot of issues regarding health care treatment for the children. They listed our gaps and provided us with the correction. We followed it and improved our performance. There was a fear during the COVID pandemic. We were not confident to treat the children. But we applied the prevention measures and told them to bring their children by using the preventive measures. We were improving our shortages gradually. Because rural mothers don't trust that the pandemic exists. But we tell them the prevention mechanisms like wearing masks and washing hands. We tell them how they can prevent their children as well. Hence, we were creating awareness and providing service side by side; without stopping the service.

00:16:20

I: What was changed?

R: the change was that there was no medicine available in the HC, but they made it available. Gentamycin, vitamin K, Zink, and Amoxicillin were not available; but were fulfilled later on. They provided us with different manuals. The manual we used to treat iCCM before the training and the one we started using after the training was quite different. They provided us the soft and hard copies of these manuals. From the previous paperwork, they enabled us to provide digital service on a tablet. A mother doesn't waste her time waiting for the service at this time. That was how we copies the services on the register after the treatment. There is a big difference between reading and filling the register and using the tablet to copy it on the register. That was the advantage of the support.

00:18:15

I: Was the support you got from the project and PHC helpful?

R: perfectly. On is its easy handling while providing service for the mother with a sick child. It improved the trust of mothers; and respectfulness. At the time I turn the pages in hard copy, she might be fed up and lose attention to tell me the correct history. The tablet enabled me to communicate face to face and collect the correct history. I can also get time to reconfirm her words by making her repeat them. Hence, I get adequate time to ask problems of the child and herself. They provided us with a document understandable in our language; Afan Oromo.

I: comparatively, what are the other differences you observed in the previous and the new manual?

R: amoxicillin dosage was reduced from 10 to five days. Registration was somewhat difficult in the previous manual. Now it was shortened and with the language, we can understand.

I: are you referring to the CBNC manual?

R: yes

I: what were the supports that enabled you to withstand the impact of COVID and continue the iCCM service?

R: the first support was to create awareness so that they can get health care services; by taking COVID preventive measures.

I: What could have been done differently?

00:23:50

R: masks and sanitizers should have been availed to the adequate level by the time. If mothers come to us for services in large numbers, we don't have adequate rooms for them. We could have served them by keeping their distance. We don't have water sources at the HP. But these did not significantly affect our iCCM service.

I: why do you think it doesn't affect the service significantly?

R: because the HC is proving us step by step. Mask sanitizer and soap were distributed during the COVID time. Because it is not much serious at this time, we don't feel the shortage. But still, it should be availed.

I: Did you get the support system helpful (mentorship and on-site coaching methods, supervision, technical support, PHCU level PRCMM, etc.) to enhance your skills?

R: yes it has helped us perfectly. As a human being, one can have a shortcoming. We might miss the steps that should be provided for the child on treatment. They showed us our gaps to be corrected. Shortages like medicine, MOAC, thermometer, and weight scale were fulfilled. Gaps and challenges were sufficed by the support. It is when we identify the gaps and report as well as when there is supportive supervision that we can tell and access the shortages. Rather than limiting our service at the HP, when we go home and consult the mothers about their child's health status we can attract them more. The support from the project initiates the supportive supervision from HC and makes them stronger. The support helped us to provide with up to date information and manuals that can boost our skills. It helped us to prepare a plan collaboratively.

00:28:22

I: does the support increase your acceptance in the community?

R: yes. We were not able to provide treatment for the infants; even though we were educated at the college level. It is after we got the training, that we started treatment. That helped the community not to pay the extra cost and that she can easily get service for her baby at the HP. That has increased our skills and improved the trust of mothers. At the time she sees her child is cured in the hands of the HEWs she can be happy. That makes her disseminate the information; so that the others can utilize the service. No reason to hinder us save the children from dying.

I: what makes the onsite coaching and mentorship different from the traditional off-site training and woreda level review meetings?

R: the onsite coaching and mentorship are practically shown us the activities. Seeing while it is done is quite different from asking from a distance. At the time they evaluate and show me my gaps; I understand my gaps and try to correct them. That helps me to see the gaps that I used to be thinking of as correct. The onsite coaching helps me to understand my gaps. The review meeting is used only to compare the activities accomplished in terms of number. That doesn't help me as such.

I: can you tell me the gaps they identified and corrected for you?

R: for example, breathing is measured only for pneumonia and escaped for another kind of disease like diarrhea. But I measured breathing for diarrhea and they corrected me then after.

I: are the strategies acceptable commonly for the HEWs?

R: I don't think there is anyone who is not comfortable with the support strategies. Because someone working can commit a mistake. It helps to identify and correct that kind of mistake. At the time someone mistakenly treats children and harms them; it is considered as if the child is killed. Hence, correcting the mistake that can save one life should not be resisted by anyone.

I: what do you suggest as an improvement?

00:36:30

R: it is better if adequate reference materials are available; that we can refer to during our spare time. That is how one can improve the skill and knowledge. It is hard to treat people arbitrarily. I also suggest incorporating updated materials into the tablet. It is better to provide an audio-visual lesson so that we and the mothers can see and

understand the issues related to the iCCM service. Rather than talking a lot to the mother, it is better to give it to them so that they can listen to it in their spare time.

I: How does eCHIS implementation help you with iCCM service delivery?

R: after we took the training and started providing iCCM service, the eCHIS application is guiding by itself. It assists in case identification and enables completing the information recording. Then it shows the prescription and appointment. It has separate categories for the mothers, children, and others. However, it cannot retrieve information about the children who were treated. It simply mixes the children in the main list found on the tablet. Hence, the contribution of eCHIS is very great. The previous service was time-consuming. Now eCHIS made it faster.

I: what do you think is the reason why you are not able to retrieve the data?

R: I don't know the reason, but it simply mixes the names of the children who got treatment. The name of the children returns to the original listing and I have to search by the name at the time I wanted to retrieve the information. At the time we wanted to assess the latrine utilization of a given household, you can get it. But not for the data of these children.

I: How does the eCHIS help you to assist in a follow-up and defaulters?

00:46:48

R: there is a pictorial as well as a written guideline while treating and following up. At the time I click on the reminder, I can see the children on appointment. I check the child again if the mother comes according to the appointment. I try to inform her about her appointment and ask about the status of her baby. Lastly, I will move to her and see them in person. The eCHIS is very helpful in that regard.

I: how about the advantage of eCHIS data quality?

R: the eCHIS shows red at the time we commit a mistake. Otherwise, it is guiding every step of case identification and treatment.

I: regarding the data quality, you used to record in the tally sheet, card, or register. But it was full of errors. How the eCHIS does help you make these records free of error?

R: there is a great difference. I can easily synchronize the data to the server so that it can be stored centrally. But while using registers, is time-consuming. It is paper-free and reporting can also be possible. It can show the figurative information on the services provided to a mother. It is not possible to include false information. The

mistake committed can move to the federal level. It only reports what we did; no room for mistakes regarding numbers. For example, for 5 mothers who took the long-acting FP, it only sends 5 and will not allow adding the wrong number. It shows when she took it when to remove and all the necessary information. If you add more numbers, it will not allow you to proceed. It guides you to the next step or limits you from proceeding in the wrong direction. We also take fingerprints of pregnant women and those who took FP; but not for the children.

00:53:20

I: can you tell us the points that should be modified in the use of eCHIS?

R: there is a place where it says any medicine; after prescription of the main one. For example, it mentions the dose of Amoxicillin and asks for additional any kind of medicine. It needs to be mentioned. The other drawback is that it orders you to take temperature using a thermometer; then asks for an additional temperature check. I wonder why it asks twice. I think that should be corrected. I don't have more to be included in the iCCM, but it is better to include additional diseases that can affect infants more than 2 months and less than 5 years. I don't think there are underweight or overweight options for the neonate less than 28 days. Gentamycin and amoxicillin are delivered when the expiry date approaches.

I: what is wrong with the 'additional medicine' option?

R: I wonder why it asks for 'additional medicine' after I ordered the main one; for example amoxicillin.

I: what do you have to be included in the eCHIS?

R: we use the eCHIS by reading what is found in the tablet. But it is better to include an additional descriptive video that we can use for the mothers. Because mothers might be terrified or burst into tears when I tell them about their babies' disease. Had she heard it from the video that explain the disease, she might be calm.

I: is it not described by a photograph?

R: yes there is. But I said it is better with the video. Because she is eagerly waiting for me until I tell her about the problems with her baby. That could have assisted me by convincing the mother and making her care about the medicine prescribed; by properly giving it to her baby.

01:02:18

I: How was the introduction of the iCCM/CBNC module into eCHIS affected the iCCM/CBNC service delivery?

R: CBNC iCCM starts from early ANC. It starts with that and finally aggregates with the child after delivery. At the time PNC is done, the very severe diseases on the neonate will be assessed for the baby. I think CBNC is good to see both mothers and their babies together.

I: do you have additional information on the way the case could be identified in the integrated CBNC module?

R: we should ask the mother for the identification of cases with the neonate. Hence we touch the mother while treating a baby. Hence it is easy to associate mothers with their babies. While treating the baby, mothers can also be diagnosed and get solutions for any possible problem. That simplifies the activities.

01:06:30

I: How was it before?

R: we only focus on the babies in the previous times. But after the CBNC module, we started to serve the mothers side by side.

I: how about clinical treatment and referral cases?

R: we used to use a chart booklet; but after the eCHIS/CBNC, it helped us to easily identify them. There is an option that asks for the case to be referred. For the diseases we can treat, we don't send it as a referral; but for the cases more than our capacity, we send it as a referral to the HC. We used to provide paper for referral cases, but now it is easily sent from our tablet. The tablet also helps us by determining cases that should be referred to the HC. But in the previous time, until we identify and determine the case to be referred, the child can be harmed by the disease.

I: how about follow-up and defaulters?

R: previously, after treating diarrhea, we check them after two days, whether it is recovered or not. But on the tablet, it can show the available appointment. It asks whether follow-up is needed or not. at the time you check yes, it records the appointment and reminds me of the date by color. At the time the appointment is passed, it shows red color.

01:10:50

I: what additional advantages are there with the follow-up?

R: the color change is a reminder of the appointment. At the time the date is passed, it asks for the fate of the child. Hence it is better than the one before. It has four colors green, yellow, silver, and red colors and has a meaning about the appointment.

I: is there something you can suggest to be included in the module to improve it?

R: I suggest having the hard and soft copy references that can improve our knowledge and skill.

I: is there any mechanism by which you can check the client satisfaction?

R: we only see their feeling personally; otherwise there is no mechanism by which we can check it on the tablet.

I: Describe the main issues faced by the health system to identify and treat neonatal infections in the community. What are the critical factors affecting the **delivery of iCCM** to clients?

01:14:50

R: there is no significant challenge we face in the community; while providing iCCM service. The main point is our smooth relationship with the community. At the time we build a smooth relationship, a mother can tell us about her private issues; let alone the child's problem. Hence it depends on the level of relationship. But if we use hard words and rough blame some communication, she might be discomforted and even don't want to come to us.

I: how about lack of competence or shortage of supplies that can determine your acceptance in the community?

R: there was no significant challenge that I faced. But there might be a time when a mother passed me because I was not able to provide good treatment. Maybe due to the medicine I prescribed or due to improper use by the mother, the baby might not recover and she moved to an alternative health facility. She might not come back again even though her baby is still feeling sick.

I: hence, do you believe that the HEWs have adequate competence for treating iCCM?

R: I don't think so.

I: it is obvious that there is no one complete, but in which area do they show a lack of competence?

R: If you don't have adequate knowledge for treatment, the mothers might not be happy and are not willing to come again. Mistrust develops then after. I think the short-term training provided might not be adequate. Because we are dealing with lives. On top of that, we are loaded and busy with other activities. that can compromise the service we provide.

I: how about a shortage of supplies?

R: gentamycin and zinc are frequently lacking.

01:20:07

I: support is not only medicine, how about the impact of a shortage of materials?

R: the weighing scale is not available. We use hanging on a spring balance. Mothers don't like that; afraid of putting their babies in the bucket. The last time a given bucket was given to us; even though it holds a baby, mothers are afraid to put babies in it. The thermometer is not functional most of the time. Out of 4 available thermometers, only one is functional. The batter may be dead or drop off and break; because we carry it

everywhere we go. It is better to have a reserve. The other is a lack of pictorial explanation of the diseases. We were given a reference manual only. It is better to get pictures describing the disease. We can post it in schools and public gatherings. The device we use to measure heartbeat is also lacking.

I: How does the shortage of support influence your iCCM service?

01:22:55

R: the support provided did strengthen our competence. There is no way it limited our capacity; except for the interruption in the last week. There is no influence on the iCCM service.

I: how about the low community demand?

R: mothers could not bring their sick baby at a time which may get complicated. For example, pneumonia can be turned into another kind of disease. Or the medicine provided may not cure the baby. This time, she might tell others that the medicine given to her is not curing.

I: What could be the reason for a mother not to come on time?

R: it may be due to distance, awareness, I might not be available, she might expect that the baby could recover, or due to my lack of competence. When the medicine is not according to the disease, the baby might not recover.

I: how about insecurity in the area; how does it affect the iCCM service?

R: insecurity might influence the service. Not only that, conditions, filling of rivers, or muddy roads can hinder to access health care services. The baby might be harmed while walking a long distance to the HP.

01:27:56

I: How has COVID affected the iCCM service?

R: it hindered the mothers from getting health care services. We were restricted from home-to-home visits and screening of the babies. Babies might be harmed because they are restricted at home while feeling sick; due to the fear of COVID. Had there been no COVID, mothers could have come to us and gotten solutions for their babies.

I: is there a baby affected due to lack of treatment during the pandemic?

R: there is no baby affected by COVID pandemic. No problem happened to them yet.

I: if no problem happened due to the COVID pandemic, it is only the fear that restricted the people at home?

R: yes

01:32:59

I: How does the COVID pandemic affect the livelihood of the community?

R: We were very terrified to go to the community for ourselves. The community suspects us for the COVID, by saying you are the one who brought COVID to us. Hence, the impact on the health service was very significant. This was common, even at the national level. Even though the service was decreased, there is no one affected due to this.

I: what were the COVID-19 adaptive iCCM implementation strategies?

R: by using masks for ourselves and by the mothers. Keeping their distance. Washing hands at the gate. We provided awareness about COVID. The iCCM training was provided after the COVID pandemic.

I: what strategies did you use to prevent COVID 19 contamination while a home visit?

R: we used sanitizer before touching the babies. We tell her the preventive mechanisms and the safe way of treatment.

I: how were the linkage mechanisms during the pandemic?

01:38:33

R: there were no suitable situations as such, but mothers do have our number and they can call us at any time they need our support. At the time they call us, we let her come, if possible, or go to her home. Then we use masks and sanitizers. Otherwise, there were no other mechanisms that we used as a linkage. There was no attention paid to assist the challenges of the pandemic. That was why our service decreased over time. But we tried to form a committee that can create awareness and assists in the prevention of the disease. That was almost after the COVID issue was calm.

I: What were the contributions of the community during the pandemic?

R: there were male and female groups, religious leaders, and elders who participated in awareness creation. We also used school and kebele structures that moved from home to home in awareness creation. Home to home, religious organizations, 'Afosha'- money contribution mechanisms by the women were used. We paid attention to pregnant women and children. We were aware they had to wear a mask while accessing health care services. we arranged a hand washing facility at the gate of HP.

I: How do the available structures were communicating for the service?

R: attention was paid from the woreda level and assisted us by organizing a committee that can be used for awareness creation; including ourselves.

01:44:30

I: What are the high-level benefits that are attributable to this support/IR?

R: it helped mothers to get health care services for their babies without any challenges like distance, lack of transportation, carrying the babies a long distance, without an appointment for the service. They were relieved from being undermined, mistreated, or ashamed while seeking health care services for their babies at the HP. They are getting free-of-change services. We are closer to them and know each other very well. The biggest benefit is saving the neonates from dying. We are proud of saving these children. We are proud of our skill of saving them and referring to complicated cases. At the time mothers develop trust in us, I am happy and stratified. I admire that I can do it. It also helped us to stand by for any kind of epidemic like measles, polio, or pneumonia. At the time one bloody diarrhea happens in one of the 14 villages of my catchment, the repetition might be considered an epidemic and we take immediate action at the specified site. We create prior information for the community like proper use of latrine, drinking water treatment, and the like. That is possible because we were capacitated to do that. That also helps to protect their neighboring and the other households.

01:48:06

I: how about resource utilization?

R: In medicine, for example, amoxicillin was used to expire due to lack of treatment. I feel it because it was obtained by investing money. But at the time I give it to the children and cured, I feel happy; because I see the future of the children. When the reverse happens, it demoralizes me because I consider them just like one of my children.

I: Please explain to us the feasibility of this support/ IR for national scale-up?

01:50:30

R: the project has a great contribution. When mothers suffer challenges in the hospital or HC, it is heartbreaking. But now they are relieved from such kinds of challenges. If attention was paid, and adequate knowledge is obtained, the service has a lot of advantages. But if we don't own it, there will be no change. That leads to false reporting. But if at least one or two are cured, or saved from dying, it is very satisfactory.

I: How is it possible to scale up to the national level?

R: I suggest training should be provided for the respective bodies. This was given only for three woredas in our area, but it should be extended to other places so that they can be benefited from the service. For example, my colleague did not take the training, but she can manage it under my supervision without taking the training just like me. At the time I am not around, she might not be as effective as I do. There might be others who don't know about this service; even among ourselves.

01:54:14

I: did you do visible activities up to now?

R: yes, even though it is not a hundred percent. For example, water, light, and other resources that can reduce our confidence. IV and other materials are not adequate for the service.

I: What should be integrated while scaling up?

R: there should be tablets distributed to all. Cards like nutrition, children, and mothers would be available. Refreshment training should be available; because training alone is exposed to being forgotten. The performances should be documented so that they can be used as an experience sharing. Like photographs and other materials should be available. One should be confident while providing the treatment. For example, during treatment, if the baby fainted, one should not panic; rather she should think about how to manage it. Interviews with the mothers should be documented for later use.

01:59:11

I: what do you think could not easily be integrated into iCCM, if scaled up to the national level?

R: there is nothing that cannot be integrated. But referral cases for the children, feedback about the child is not replied to quickly. We only check it from their mothers.

I: How are the activities/efforts embedded in the PHC and woreda routines/work streams?

R: we never hesitate to see the health status of children; when mothers complain; except for the serious issues that could hinder us. we provide priority for iCCM service.

I: do you think this commitment is also true for woreda level performance?

R: people are quite different. Lack of dalliance can lead to death or harm to the children.

I: how do you integrate iCCM planning with the other general health care service?

R: iCCM series was once forgotten, but is now initiated. It is still not that much active in remote areas.

I: do you apply microplanning?

R: yes, we plan for under-five children. That is how we plan in collaboration with HC and post it according to what you are seeing on the wall. But that same attention was not paid at the HC level as we do. There is a performance gap at the HC. We plan and evaluate the outcomes for the under-five children.

I: Do you have anything think is important to tell us that we have not asked you?

R: there is nothing I can add. I thank you for your suffering in the rain to come to this HP. I suggest refreshment training and supportive supervision just like what you are doing. It is forgettable and should not be. Rather it should be continued. Committed should be formed from woreda to the HP level that can do surveillance. Rather than wondering at the time problems happened; the committee should be proactive. iCCM committee should be formed.

End line evaluation of the PSBI implementation research (IR), the qualitative component

I	Identification	
1	Questionnaire ID	**Lume-Tade-Jogo Gudedo-HP-HEW -IDI-L&T**
2	Area Identification	
3	Name of Woreda/Zone/Region	**Lume Woreda**
4	Name of facility	**HP**
5	Name of moderator	
6	Name of a note taker	
7	Date of discussion	**10/06/22**
8	Participant #	**1**
9	Audio File #	**1**
10	Start time:	**_8_:_30 __and 9:22- 9:55**
11	End time:	**_9_:_21_**
12	Transcriber	

Key: I: interviewer

R: responder

I: Hi, my name is Temam and my colleague is Lalisa. We are from the JSI L10K project. We are here to gather information on the ICCM project for end evaluation. I thank you for your willingness to do this interview. Can you please introduce yourself and let us proceed to the next question?

R: I am HEW working in Jogo Gudedo HP.

I: Can you please describe the demand generation/SBCC activities you are doing on iCCM/ newborn health?

R: we have started the service with health education, family planning, and latrine utilization. We used to provide only vaccination for the children. We used to take their weight, provide vitamin A and deworming. We used to identify the malnutrition using MOAC. We used to distribute plump nuts to the children. We were not treating children and they didn't come to us for health care services. But after this project was launched, we started providing child health care services.

I: what did you do to improve the community awareness regarding the iCCM service?

00:03:54

R: first of all, we have to be convinced by the ability to provide the service. We were providing only FP and HE services. But when we begin the iCCM service, we have to be confident enough to do that. After the training was provided and become adequate to provide the service, we were expected to provide awareness for the

community. Because they knew that we were providing only FP and latrine utilization services. It is after we started the iCCM service that we began to create awareness for the community. Then the community understood about the service and started to bring their babies for treatment.

I: What were the strategies you used to create awareness?

00:05:01

R: we focus on the mothers; where we can closely provide the education. We also use ‘Idir’, the social organization used to support each other. And also used the WDA. We used any platforms we encountered for awareness creation. A home-to-home visit is also used for the creation of awareness. That is how we attracted the community to the iCCM service.

I: How did you use the WDA?

R: we inform them to tell the pregnant women to send them to us. We also community on phone for the women in labor. Those are how we communicate with them.

I: do you use influential people in the community, pregnant women conferences, and the like?

R: we used Aba Gada and religious leaders, as well as the elders, including the chairperson of ‘idir’ (a traditional money-saving mechanism)and the like, are used for information dissemination about the iCCM service.

00:07:41

I: What are some of the biggest challenges with SBCC activities for newborn care?

R: the community was aware of the previous service, that we only provide family planning and education services. They were complaining about the injection we provide; because they didn’t see us doing that at the beginning of the iCCM service. But we were provided awareness and changed their mind. That is how we began to provide gentamycin for the infants. We had been through a lot of challenges; because they knew we only provided FP service.

I: What were the activities performed to change their attitude?

R: because the PNC is conducted by us after delivery at HC. We started to treat neonates less than two months. When we closely investigate their neonate, the community started to accept us. At the time the neonate is hot or shows rashes and provided treatment, the others hear and disseminate the information that somebodies neonate is treated in our hand and got relief.

I: can you please tell us the services provided under iCCM?

R: we treat sick children. We also check their growth monitoring; where the height and weight are going in line. It is to decide the next step if it is not balancing. We also check them using MOAC and check their nutrition status. We also treat pneumonia and diarrhea.

I: does the community know about these services?

R: yes they know and get the service. if we are not able to treat their children, we refer them to HC.

00:12:00

I: can you please tell us how the community participated in the awareness creation?

R: there is a monthly pregnant women conference. In the beginning, they come to us on the 16th week and we send them to HC. Then because the 1000 days of child safety is included in it, we inform about the iCCM service on pregnant women conference.

I: can you please add some of the challenges you encountered while providing awareness in the community?

R: the mistrust of the service we started to provide was the challenge; until they got familiarized. Otherwise, there is no significant challenge.

I: How do you describe the strength of the support you got from the PHC and the project as well as your efforts to address barriers to delivering iCCM/PSBI during COVID-19?

00:14:20

R: it is after the training was provided by them, that we started providing the service. We were not providing such kind of treatment. Rather we used to provide vitamin A and conduction deworming. We simply used to send them to HC. But after the training provided, we were able to give adequate health care series regarding iCCM. Providing treatment for a given and seeing cured, boost our morale and initiate more.

I: what are the challenges of COVID on iCCM and how the support did help you to withstand the pandemic impact?

R: at that time, people were not moving and are not accessing health care services by the time COVID was serious. But we use face masks and sanitizer and go home to home to provide health care services for sick people. We were not leaving them at home being sick; rather we tell them to come out of their home and get treatment services by taking the necessary precautions. But the community was restricting themselves at home, even though they are feeling sick, irrespective of our advice. They fear that they can add even more diseases and fall into a complex situation; if they go to the health facility. Hence we were using a facemask and tried to provide education in the community by going home to home. We make them keep their distance when they come to the HP.

00:17:45

I: what were the supports provided by the project or the PHCU and how does that contribute to our skill improvement?

R: we were provided a mask and sanitizers

I: How does that help you to provide iCCM service; withstanding the COVID impact.

R: it helped us to provide the service by keeping our health. At the time refreshment is done, it is good to get an open mind and add more. There is no time that we stopped service due to the fear of COVID. We were also supporting the service by supervision.

I: is there any iCCM service that is affected or else improved by the pandemic?

R: except for the refrain from the service due to the fear of COVID, we were telling them to utilize the health care service, including cough due to TB.

I: do you think the support provided improved your iCCM service?

00:21:07

R: We were helped to prevent the infection due to lack of prevention. We in turn made the mothers take care of the pandemic and get the service.

I: do you think the iCCM service decreased during the pandemic?

R: we were telling the mothers to keep their distance and get the vaccination for their children. No service was interrupted or decreased. Nothing new happened during the pandemic. The services were continuous.

I: Did you get the support system helpful (mentorship and on-site coaching methods, supervision, technical support, PHCU level PRCMM, etc.) to enhance your skills?

R: at the time they come for supervision, they check registers and the way we are providing health care service for the children. In addition, they provide a case and see how we can manage it. Because there might be a limitation with us, they wanted to check our performance in providing treatment. Hence, they checked our identification mechanism, how we handled the cases, and the medicine prescribed.

00:24:26

I: does the community welcome you after the service you started to provide?

R: the resistance was at the beginning. But after we started providing service and after we created awareness, there is no resistance. The challenge was on giving gentamycin which should be diluted in water. They argue how a neonate is given diluted medicine. We are doing that due to the lack of diluent gentamycin. But we show them

that there is no difference when compared to the HC syrup with the one that should be diluted with water; as long as the water is clean. Another point is to give them in the morning and night; without missing the schedule.

I: How do the mentorship, on-site coaching, technical support, and supervision help you to improve your skill?

R: there are medium or server cases. Based on the training they provided to us, I can identify the cases. The ones I can treat will be done and those which I cannot are referred to the HC. I can do that based on the training provided to me.

I: can you add more skills you got from the support provided?

R: the training provided increased our skill on top of what we got before. For example, I knew the above 60 fast breathing of the children is severe pneumonia and it should be referred to the HC. hence, I can identify cases and provide appropriate medication.

00:29:02

I: how do you explain the difference between the onsite coaching and mentorship with the offsite training and review meetings?

R: off-site training is faster. But on-site coaching is good to see the practical implementation of the training. Fortunately, they might come at the time there is a client baby. They can see and evaluate the way we provide the treatment service. The haste offsite training is quite different from the onsite coaching and mentorship. The onsite coaching and mentorship are very helpful.

I: how do you evaluate the acceptability of the strategies like on-site coaching, mentoring, or supervision?

R: yes, they are acceptable. There are no worries than transferring information and knowledge. It is a kind of experience sharing.

I: can you mention what should be improved regarding the support?

R: I suggest continuing with the support.

I: at another HP, they suggested that audio-visual supporting material should be available. Do you suggest the same?

00:33:17

R: yes, I have a smartphone and I can use it if there is something I can refer to on my phone. For example, the picture of severe pneumonia is better if it is found in soft copies to be used on our mobile.

I: How does eCHIS implementation help you with iCCM service delivery?

R: eCHIS has helped us a lot. We have lists of all the children in our kebele. Before recording the child on the register, we identify the name of the child on the tablet. Then the eCHIS itself asks for the kind of problem with the child. It guides up to the prescription; and finally, the prescription is done by using the chart booklet. I can say it has a great contribution.

I: how do you perform the case identification and data quality using the eCHIS?

R: every issue is included in the eCHIS. For example, if you want to identify cases, it guides you to measure fast breathing and ask for the duration of sickness. But the way you gather the information determines the quality of the treatment. If you wrongly enter the information, it can lead you to the wrong type of disease.

I: How does it help you in providing quality case management?

00:37:26

R: it has helped me a lot regarding case identification. But one should be careful while using it. If you don't measure the correct fast breathing; the following result could be false. It has a great advantage if used appropriately. It is more helpful than paperwork.

I: how about retrieving the children who got service?

R: for the child who received treatment, if I want to get it back after two days, I have to search for it by name. It asks for information, about whether the child is cured or not.

I: I think this is for those who are under follow-up.

R: yes, it is for those who are under follow-up.

I: how about those who completed the treatment?

R: it is not possible to retrieve the data for those who completed the treatment.

00:39:14

I: how do you think will it be possible to retrieve it?

R: I don't know. I know only for those who are under follow-up.

I: how about data quality?

R: the data quality management is very different compared to the previous one. In the previous one, the paperwork was tedious. But now after case identification, treatment and prescription, the child is registered on the register. We record it for the neonates with less than 2 months and others for more than that. That is how we can get records of the children who received the treatment. Hence data quality is better with the eCHIS.

I: How does it improve the former mistakes used to be committed during the paperwork register. Even though we are I am not able to retrieve the information about the child who completed treatment, the others can do that.

I: in the previous times, there could be mistakes like recording errors, misidentification of diseases, sex exchange, and the like. How were these improved in eCHIS?

R: it is not possible to deceive the eCHIS. Because every record is centrally stored. But there is a chance to report false data, during the paperwork. That is why eCHIS is more quality.

I: how do you differentiate between the follow-up and the defaulters?

00:43:11

R: for the child who was treated with diarrhea or pneumonia, at the time I appointed for the next five days, the tablet can remind me about the appointment. Had it been on the card, it would have been hard to remember or search for the card on follow-up. Because I carry with me the tablet all the time, there is a great chance for me to open and see it; at least three times a day. That is how I can see the reminder.

I: what do you do for the defaulters?

R: even though the tablet shows the health status of the child in red color, it is a must to visit at home and check its status.

I: what could happen to the red color if the child is not coming for them till the end?

R: at the time you check the follow-up case and provide a solution in the tablet, the red color will disappear.

I: how do you think is it possible to improve the eCHIS service more?

R: it is not possible to retrieve data for the children treated in the past. That should be improved. The other problem is that the data cannot be retrieved for the specific zones in the kebele. There are three zones in the kebele and the data for a household leader for each woreda can be obtained but not according to male and female category. Rather it shows the male and female categories aggregated to the kebele level. It doesn’t also show the women between the age group of 15-45 according to their zonal category. The other is, that we are not able to obtain data for under 2 years and above. There is a format that asks us to fill the number according to the age mentioned. We are not able to get that on the tablet. If we want to do it, we are expected to search by their names under the household folder. At the time we are asked to report, we list them in their zone and search for the names of the children who receive the treatment. I suggest making it in an easy way using the eCHIS.

I: How was the introduction of the iCCM/CBNC module into eCHIS affected the iCCM/CBNC service delivery?

R: it is easy to read and understand the module and interactively apply them for iCCM service on the tablet.

00:50:31

I: How does it help you with the case and registration of sick babies? Including the referral and follow-up?

R: the manual had boosted our knowledge.

I: does it help you to identify and treat the cases?

R: yes

I: how about a prescription?

R: for example, if a given child is treated for pneumonia, prescription of medicine is done based on the weight and age. Because the weight and medicine prescribed should be proportional. Then an appointment will be given to the mother on the time she should come back again.

00:03:50

I: does the integration of the module standardize the iCCM service?

R: yes, we are only able to use it according to the standard set in the module.

I: does the community satisfied with the service you provide?

R: yes, they are happy when they see their children are cured.

I: How was the feedback from the referral cases?

R: after we send the child to the HC, some are provided with feedback while others are not. It is only from the mother that we can confirm whether she went to the HC or not. Those who are working on under-five children were taken the training recently. That is why they sometimes send the feedback and not the other time.

00:07:14

I: you told me that integration of CBNC in the eCHIS is advantageous. What changes did you observe on the side of clients; after you shift from the paperwork to the digital form?

R: at the time they see us serving their babies using the tablet, they are happy and understand that the government is paying attention to their children. They say living longer can make us see a lot of changes. Because we are taking fingerprints, they are happy at our service.

I: it there anything you suggest to be improved?

R: it is good as compared with the previous one. But the change has to be continuous.

I: Describe the main issues faced by the health system to identify and treat neonatal infections in the community.

R: the challenge in the community was the mistrust of our service. At the time we were recruited, we were told that 75% of our service is in the community while 25% is in the HP. We were not given the chance for treatment. There was a big challenge in saying what the contribution of HEW was. That was solved gradually.

I: is there a gap with the skill of HEW?

R: the aim of HEWs is prevention; not treatment. Due to the challenge in the community, the government shifted us towards the treatment; by giving training for us. We were only providing health education, vaccination, latrine utilization, and family planning. We were once used to be called the children of a latrine. We are happy when we see the cured children by our health care service. They were used to shut doors upon us; when we go to them for HE and latrine utilization. They say please shut the door on the children of a latrine. We had passed all these challenges and reached at the time of treatment provision. We will continue by strengthening our skills.

00:14:38

I: how was the shortage of support affected the iCCM service?

R: after the project, we can access the medicine from the HC. There was no shortage of medicine. There was no time at which we become short of medicine for the neonates. We don't have a functional refrigerator for keeping vaccines. We are accessing the vaccines from the HC twice a month; when there is a vaccination program. We take it, vaccinate the children and return it; because we don't have a refrigerator to keep the vaccine for the next day.

I: How does the low community demand affect the iCCM service?

R: there is no challenge to create awareness for the community, including distance. The majority of the community are getting health care service with us. but more than 50 households, which were found in Kiriti village, are not coming to us. They have access to transportation to Adama and move there. There are no single women who want to deliver at HC from that area. So does the children's vaccination. The kebele is very vast; we are not able to cover all. That is a challenge for us to provide good health care service.

00:21:48

I: is it due to awareness or due to distance, which made the community not willing to take the service at this HP?

R: it is hard to judge, if we call it due to awareness, others come from neighboring. Neither does the distance limited from the service.

I: How does the insecurity affect the iCCM service?

R: there is no security problem in this area. It was about three months since I came to this place; I am working in the community for iCCM service since then. I saw no single security issue that can hinder me from working.

I: How does the COVID 19 affect the iCCM service in the community?

R: there was no serious issue that happened regarding COVID. We provide service by using a face mask. There was a time when schools and colleges were closed and people were keeping their distance. We were not limited to giving service at that time. Even though we were burdened, we didn't stop providing the service. We were moving in the community, and at the time we face a sick individual, including cough, we send them to HC or treat them at HP; especially the children. We and other health professionals were creating awareness for the community by wearing our guan, by the time. We were helping mothers not to keep their sick babies at home. We did a lot of activities to make the families not suffer.

00:26:52

I: Has COVID-19 affected your daily routines?

R: no

I: how about your work on a newborn?

R: adults are allowed to wear a mask; but not neonates. Because they can be suffocated. But the family is not able to bring their sick babies to the HP for 2 to 3 months due to the fear of COVID. But we were supporting them to bring their children.

I: How has that changed over time?

R: the school started after a while. We used them to create awareness. We tell them to keep their distance and use a face mask. Still, now they are using sanitizer and we made them aware of the preventive measures.

I: how were the COVID-19 adaptive iCCM implementation strategies?

R: we were telling them to use preventive mechanisms and come to the health facility for health care services. We made the pregnant women deliver at health institutions by providing education. That is how we made them come to the HC as well; for those who used to resist to come even to the HC.

I: how did you provide the education, whom did you use to deliver the education, or what were the strategies to deliver the education?

00:31:09

R: regarding the HC every professional was campaigning by using a face mask, sanitizer, and guan. That was how we were moving in the community to provide education. The Red Cross was also coming to us from Mojo town and was providing the education. That was how we told them that they can get health care services at any health facility.

I: what were the motivation and competence of HEWs which enabled them to provide the iCCM service irrespective of the COVID pandemic?

R: the perception was that death follows every COVID infection. Because we were seeing what happened abroad. This was also felt among the health professionals. There was a suspect who was taken to Mojo town and the family was quarantined at home. Then returned home safely. That was a point when the community became well aware of the disease. It was realized that by using preventive measures, one can be safe. When the vaccine is available for that; everyone can be free of the fear. That was how the community became aware and started using the service.

I: what motivated you to provide the awareness?

R: because the wrong perception in the community can only be corrected by providing education. The community was restricted at home due to the fear of COVID; even though there could be another disease.

I: How were the community engagement and awareness creation?

R: Aba Gada and other community leaders were used to disseminate the information.

00:36:03

I: What do you think are the reasons for non-significant changes?

R: there is no child kept at home while feeling sick. We are only challenged by taking their weight by moving into the community.

I: What are the high-level benefits that are attributable to this support/IR?

R: saving the children from death is a big benefit. The community is saved from unnecessary costs and the service was free. The provision of health care for these infants had boosted our confidence and hence our acceptance in the community.

00:40:13

I: Please explain to us the feasibility of this support/ IR for national scale-up?

R: yes, it is possible to scale up.

I: how?

R: we had been through many ups and downs; yet we are not where we were by now. From not being able to provide treatment, we did after getting the training. If this is scaled up to other places, it can be more effective than ours.

I: what could be the obstacle to scaling up?

R: inability to provide treatment for the infants is a challenge. But if we can do that it is ok.

I: What could be helpful to scale up and what could be a challenge not to scale up?

R: HEW is found everywhere, as long as they are provided education and are adequate to provide the treatment, it is possible. They are women, they know the feeling for the babies. If they get the training, they can provide treatment for these babies. The insecurity in the country may be an obstacle to scaling up. It is when the area is peaceful that we can train and enable them for the service.

I: do you have something to add about the challenges and opportunities for scaling up?

00:45:15

R: the HEWs have to be committed to the service. In case there is a baby who is not able to come to the HP, she is expected to go home and treat. There is nothing that can hinder us from providing the treatment service. Collaboration between the education, health, and agricultural sectors is helpful to support each other by sharing the experience.

I: How are the activities/efforts embedded in the PHC and woreda routines/work streams?

R: we plan for the diseases like pneumonia or diarrhea based on the eligibility. We do for all of the services in such a way. That is how we are planning and evaluating our performance.

00:48:00

I: how do you make the iCCM service your daily activity?

R: at the time a child comes to us, we take their weight and MOAC; to provide the treatment. We do the same while moving into the community. We are not going to the community only for a single service. At the time we visit a given household, we provide all the 18 packages service. We know every child in each household; hence, we can give the treatments for each. At the time we get a child, we evaluate the health status based on the 18 packages. We are not limited to treating sick babies. At the time we visit 10 households, we provide all the 18 packages for each. We don’t give only one package and leave.

I thank you for your time and valuable information. You can add more if you think it was not mentioned.

R: I thank you too. I suggest the project provide refreshment training for us. It is better to fulfill materials like a weight scale, thermometer, and the like. I also suggest updating information that we can use on the tablet.

End line evaluation of the PSBI implementation research (IR), the qualitative component

I	Identification	
1	Questionnaire ID	Lume-Tade-PHCU-DIRECTOR- IDI-L&T
2	Area Identification	
3	Name of Woreda/Zone/Region	**Lume Woreda**
4	Name of facility	**PHCU**
5	Name of moderator	
6	Name of a note taker	
7	Date of discussion	**08/06/22**
8	Participant #	**1**
9	Audio File #	**1**
10	Start time:	**_8_:_30_**
11	End time:	**_10_:_30_**
12	Transcriber	

Key: I: interviewer

R: responder

I: Hi, my name is Temam and my colleague is Lalisa. We are from the JSI L10K project. We are here to gather information on the ICCM project for end evaluation. I thank you for your willingness to do this interview. Can you please introduce yourself and let us proceed to the next question?

R: Yes, I am willing to participate in this interview. I am Tade PHCU director. It has 7 satellite HPs that are providing health care services.

I: Can you please describe the demand generation/SBCC activities you are doing on iCCM/ newborn health?

R: iCCM/CBNC service was there, but due to some strategic obstacles it was weakened. JSI L10K had initiated it and was supported by technology in which iCCM service has been provided. PHCU director, vice director, under five focal persons, and others were trained to provide this service. After awareness was created in the community and behavioral change was done, the service was started. The iCCM service at this time was far greater than that of the previous one. This was evaluated once and twice and checked that it is far greater than the previous one. Focused supervision was done in collaboration with the woreda and HC supervisors.

00:04:58

R: hence the progress is found on a good status.

I: What are the strategies used to raise awareness?

R: at the beginning, we did training for the professionals and initiated them for the service. Then we used kebele administrative structures to create awareness about the service. They once thought the HEWs were not able to treat neonates and bypassed it. We told them that they can do. We used WDA and pregnant women conferences to disseminate the information. The necessary logistic for this service was partly delivered from this project and the others are accessed from the HC by health care financing.

I: What are other strategies you used for SBSS?

00:07:30

R: we used religious leaders, Aba Gada, including public figures, and women who participated in the training. They were told about the advantage and disadvantages of the service. Those women who have a better understanding are selected and together with the HEWs, they were provided awareness and training. They are made to initiate the community by creating awareness. So do the religious leaders and Aba Gada.

I: What are some of the biggest challenges with SBCC activities for newborn care?

R: the challenge is primarily about supplies. The community mostly doesn't trust the service by HEWs. They rather want to go to the Mojo town; bypassing the HP. We tried to provide awareness, but still, few are doing that. Partly because of the proximity to the Mojo Hospital. The other reason is the lack of confidence among our professionals to treat neonates. We found in the discussion panel that some of the professionals are in fear of treating the neonate. Because of lack of practice, due to few cases treated with them, the neonate treatment needs adequate exposure to the diseases. Hence, due to the fear, professionals are not confident enough to treat the neonate. It was a challenge until they develop confidence by the time. We are not also able to provide complete awareness.

I: What could be the reason for not providing the awareness completely?

R: the possible reason could be resistance from the community, lack of repeated supportive supervision, and lack of repeated assessment of the performance. For example, at the time information is disseminated, the impact of the action should be identified. Inadequacy of the information dissemination by the responsible individuals. Because it is not possible to force the community, complete compliance with the information provided cannot be possible.

00:12:50

I: do you think the community has the information about the iCCM service?

R: yes, they got information; even though it is not 100%. Especially the ones who are closer to the administrative structures in the community and influential people know it very well.

I: how did the WDA participate in the awareness creation?

R: they participate in community awareness creation and send the children to HP for this service. at the time we ask from where they heard the information, they tell us that the WDA member by name and that she told them to go there.

I: are these WDAs functional at this time?

R: yes

I: How do you describe the strength of the support you got from the PHC and the project as well as your efforts to address barriers to delivering iCCM/PSBI during COVID-19?

00:15:50

R: I see the support in three directions. One is training and awareness for the HC staff. The other is the supportive supervision we provide for the HP. The third one is support provided in collaboration with woreda, projects, and HC for child clinics. The other is the HMIS and PHCU vice director, who took training, goes down to the HP, and provides awareness on the way to use eCHIS. Logistic support was provided from the HC to the HP. There is no significant support we got from the woreda. We provide them by using our health care financing system.

I: How does the support help your effort to provide service irrespective of COVID 19?

R: the fear of COVID restricted the service utilization. We used a loud speaker to disseminate the information on the prevention mechanisms together with Red Cross and using an Ambulance. We told them that the health professionals are available at HP to serve you and you can safely utilize the health care service. That is how we boosted confidence in the community. We then told them how they can take care of themselves.

I: What was changed?

R: they started to come to the HP. They also started using face masks when accessing health care services at the HP and HC.

I: How was your readiness to provide the service at the time?

00:20:59

R: we arranged a hand washing facility both for staff and clients. We provide sanitizer for the clients and staff to use. We distributed the prevention materials to every HP in our catchment. We were given priority to mothers and their children.

I: can you please tell us the support provided by this project?

R: We were expecting materials support like medicine, but they are promising to do it; not yet provided. They gave us mentorship, supportive supervision, and training for capacity building.

I: What could have been done differently?

R: material supply should be available. We used to access the supply from NGOs. But now they are not supporting us. We decided to buy from our health care financing. We sell it with 25% profit at the HC. But we give it for free for the HP. We buy and provide it for free for the HP service. These can lead to collapse. Especially at this time when exempted services are increasing. FP, delivery, under child health care, HIV, TB, and others are provided for free. Sometimes the medicine expires before use. Essential drugs can be missed from the market and create a burden. This directly or indirectly affects the system. At the time we reported the issue, the federal officers told us that it needs to be studied and that solutions can be provided sooner. But not solved yet.

00:28:46

I: what changes did you bring?

R: we started focusing nutritional status of the children. Because malnutrition can affect their appetite and hence immunity. Hence, malnutrition causes disease, and disease can cause malnutrition. Hence we did a lot of activities regarding nutrition. The first one is screening children for malnutrition. The second screening was done on mothers. The third activity was farm garden; in collaboration with save the children and inter-sectoral collaboration. This was broadcasted by OBN. We created an educational forum for mothers, school children, and the general community about malnutrition. Hence, they can provide a balanced diet to their children.

I: do you think awareness is the root cause of malnutrition?

R: yes, because they have the resource. We demonstrated cooking, using purchased vegetables. But we were expected to produce in the garden and show the cooking as well. We cultivated about 8 varieties of fruits and vegetables and used them for the demonstration on the forum we had prepared. Hence we showed them how to produce and feed the children.

I: Did you get the support system helpful (mentorship and on-site coaching methods, supervision, technical support, PHCU level PRCMM, etc.) to enhance your skills?

R: previously, they used to provide service only depending on the college education. But now, by taking training, further supported by technology, they started providing the service. Before prodding the service, they were giving awareness. It guides and shows the quality of service by color. If an incorrect prescription is done, it doesn't allow to proceed. Hence, it shows her mistake and guides her step by step. If the service is not included in it, it doesn't

allow her to proceed; we are expected to suffice that part. I can say the integration done between iCCM/CBNC and eCHIS is very great.

00:34:48

I: do they give you supportive supervision and mentorship?

R: yes, they gave us and evaluated the outcomes. We got mentorship and supportive supervision twice. But it was not adequate. Because it was not timely and regular. But they were focusing on HP and took adequate time to deeply support the HEWs. The support provided by woreda was also not regular and adequate.

I: What were the gaps created due to that?

R: if that is not regular, people are ignorant due to loads of activities. A lot of activities are there, for example, campaigns, ODF, and others. At the time one gets busy, the regular task can be forgotten.

I: what was changed by the support provided?

R: the skill of HEW is improved. They were not able to provide treatment. Their attitude was changed. Their attitude towards treating the children was changed from not being able to treat them to have the skill. Previously, they simply ignored the rashes they used to see on babies. Then after, they started to check their attachment, position, and physical wellbeing. They started to perform identification using a chart booklet. But they were ignoring the signs and symptoms of babies by saying this could be simple and recover soon. They were not able to perform an assessment.

00:39:57

I: How does the support provide changed the attitude of the community?

R: we are reducing the negative attitude that the community hesitates about the skill of HEW. But we cannot completely solve it. Hence, the number of individuals bypassing the HP is decreasing. That is evident from the reports provided. The number of individuals who got the treatment is increasing from time to time.

I: What were the reasons not to bring a complete change?

R: the first reason is the attitude of the community. Our PHCU is found between Adama and Modjo towns. People prefer to go to either of the two. We cannot say it is due to a lack of awareness; or willingness to come to the HP. They are intermediate and is very hard to judge what is missing. Those who are educated and have the money want to bypass higher health facilities. Even, they prefer medical doctors over others. They started to prefer specialists in pediatrics. We are doing further awareness creation on these people.

00:42:48

I: How does eCHIS implementation help you with iCCM service delivery?

R: it has job aid. It guides and provides information on the service provided. It was standardized and improved the health care quality. It simplified the service and reduced the paperwork of HEWs. Because everything is done by the tablet. Hence it saves time.

I: How does it help in case identification?

R: the availability of job aids also helped to identify cases easily. It has also pictures and easy understanding. It used to take a longer time for the health professionals to arrive and assist the HEWs in case identification. But now, I can easily show to them my pictures. That boosted their confidence and treatment ability.

I: How does it differ from the manual one?

R: it is known that paperwork is sluggish and takes time for case identification. The tablet can easily identify and show the disease by picture. The community is interested in the service provided when a fingerprint is taken and assisted by such kind of technology.

00:46:46

I: how about the quality of case management?

R: after case identification, case management is also guided by the eCHIS. It hinders proceeding when wrong steps are done. It has an appointment and follow-up tracing. At the time appointment is reached, it aware of them by colors like yellow or red.

I: How does it help in retrieving patient records?

R: there is an alphabetical listing; you can easily search for the family folder by name. Hence, it is possible to record on the register as well as on the tablet side by side.

I: how about data quality?

R: yes it has data quality. Recently another module is added and training was provided. It shows the data quality by colors for the appointment. You can easily find your records on the tablet and see the data quality. Anyhow it has a good advantage over the manual one.

I: is it possible to perform LQAS?

R: it has information like registration, reporting format, and supportive supervision. It is also possible to check by the register, tally sheet, and reporting formats. Hence it is possible to check the data quality.

I: it is obvious that the manual work is full of errors; how was that improved in the eCHIS?

R: we have a plan to abandon manual work. Family folder coach will not be used in the future. We are planning to digitize every service. It is possible to check the performance and management appraisal. The included module has KPI, HEW, and supervisor at the HP level. To standardize HEWs, HP, and supervisors, there are 6 KPIs for supervisors, 10 KPIs for HEW, and 6 KPIs for HP. Hence it can evaluate the data quality and everything by itself. Hence, it has the perfect data quality.

00:51:30

I: How does default tracing possible with eCHIS?

R: at the time appointment is reached, it shows yellow and red for the missed appointment.

I: is it working offline?

R: yes, data recording is possible offline. But synchronization is expected every morning and evening.

I: what were the other advantages of the eCHIS?

R: the technology was challenging at the beginning; at the time it became a must to use it for HP health care service, they started practicing and now it is user-friendly. They are handling it just like they are using their smartphone. At the time there is training with other kebeles, our HEWs are faster than the others. Ada woreda complained about the fast pace of our HEWs; claiming to be trained separately. Hence it saves time and makes it fascinating. The other benefit is the quality of service. No data is missed and at the time information is needed; one can see it everywhere.

I: what do you think should be improved?

R: the module is becoming updated. We were consulted for the module updating before disclosure. The replacement of a very fast tablet is appreciable. But still few tablets are sluggish. It is demoralizing when compared with the faster one. It is better to avail the tablets to everyone in need. There is no significant problem with the internet. Updated SIM is provided to make it faster. The health net setup is installed and will be expected to be functional in the future.

00:57:48

I: How was the introduction of the iCCM/CBNC module into eCHIS affected the iCCM/CBNC service delivery?

R: at the time CBNC integrated, it made the tablet busy. But it was made better and is now faster. This time, we are only seeing the advantage, not the disadvantage. Hence, iCCM/CBNC is the health care service while the eCHIS is the digital part to facilitate the service.

I: how advantageous was it; for example, identification and registration of sick newborns?

R: it is almost similar to the one I said before. It is far better than the manual way of health care service delivery. One can see the reports being at the federal level when done with electronics. It enabled easy control of activities.

I: how about its advantage on clinical and referral decision support?

R: there is a system in it in which a referral system can be done. There is a little challenge in linking the HP tablet with that of HC. Because the one available at the HC is older. Hence, it challenges the referral system between the two. The health professionals at the HC are demoralized due to this. But the available system is very suitable.

I: how about the advantage of follow-up of sick child treatment and/or defaulter tracing?

R: the manual one is exposed to be forgotten. It is not suitable to take while moving for services. You can see the activities and referral cases being everywhere. The appointment can be seen everywhere. Hence it assisted fast decisions from everywhere. In general, all the activities are more advantageous than the manual.

001:02:33

I: does it have client satisfaction?

R: yes, for example, while we were using digital BP apparatus it is not suitable to read it. But after it is linked to the tablet, the measurements are clearly shown. That initiated the clients to take the reads and they are frequently coming to us for that service. During service initiation, a fingerprint will be taken. They are happy with it; because they are getting treatment using technology. During the treatment, because it shows the picture of children with the signs, especially those educated mothers, are happy when they read what is related to their child on the tablet.

01:05:45

I: does the application have mechanisms by which patient satisfaction?

R: we ask them for general satisfaction on the paper, not in the application.

I: what do you suggest to be improved about the integrated module?

R: the only improvement needed is the availability of a resource. Otherwise, the existing situation is very suitable.

I: Describe the main issues faced by the health system to identify and treat neonatal infections in the community. What are the critical factors affecting the **delivery of iCCM** to clients?

R: as I told you before, those who consider themselves advanced are undermining this service and they don't have trust in the service provided at the health post. For those who wanted to access the service, loads from the campaign and different activities become an obstacle. Because they close the HP and move for other services. Otherwise, the health seeding behavior was improved by now. Shortage of resources or manpower can cause a challenge for this service.

I: What are the critical factors, for example, regarding the competence of HEW's weak support system and the low demand from the community?

01:10:52

R: regarding the supply, if it continues in such a way, I fear that it could be closed. Because the free service provided at the HP can harm the purchased supplies from health care financing. The HEWs shall be exposed to the most complicated cases; because they can be challenged when such a case comes to them. Especially those cases who need treatment using ICU. If they get such exposure, they don't get frustrated when they encountered it in their real work. It is obvious that neonate treatment needs care and the HEWs may fear providing health care services because of their sensitive nature. The interruption of supportive supervision can cause to retract the service. Regular and scheduled supervision should be provided.

I: How does national security affect the iCCM service?

R: there might be a psychological impact; that is not possible to measure. Otherwise, there are no security issues in this area.

I: How has that changed during COVID?

R: there is no impact from COVID 19; the community is accessing health care services irrespective of COVID.

I: Has COVID-19 affected your daily routines?

R: the professionals were providing service using face masks and sanitizers. COVID is not an issue now. Clients are also using face masks while accessing the service. We were using a facemask including the clients while we used to provide the service during the serious COVID pandemic. This time, we all are reluctant regarding the pandemic.

I: What were the challenges by the time the COVID pandemic was serious?

01:19:56

R: there was a gap between professionals and clients. They were not able to move to seek service for the community or provide the service by the professials. This time the iCCM/CBNC is being provided as usual. Hence, there is no obstacle regarding the pandemic now.

I: what were the COVID-19 adaptive iCCM implementation strategies you used during the pandemic?

R: we did social mobilization and advocacy to initiate the people to make them come to the health facility. Those who came were disseminating the safety at the HC to the others. We used an ambulance to announce the prevention mechanisms. We told them that the health facilities are more strongly prepared than ever to serve. Performance was dropped and the case report was also dropped due to the fear of COVID. The strategy we used helped the clients to come and get the service. Acute respiratory tract infection was dropped due to the prevention measures. It had affected the iCCM services at the time; currently, it is not affecting. We are beginning to provide the the COVID vaccines because the community is adapted to the pandemic.

01:29:18

I: What are the high-level benefits that are attributable to this support/IR?

R: the benefits are obvious. It saves time and the financial needs of the community. That is why HP is opened in their vicinity. They used to bypass the service, but now getting adequate service in their vicinity. Bypassing the HP caused loads on the other health care facility and hence dropped their service quality. It gives the chance for the HEWs to build the capacity for the treatment of children. This increased acceptance of HEWs in the community. It lies a base for the upcoming second-generation HEWs program. In the absence of the project, if there were no health care services for the children, the endeavor to advance the HEWs program will be valueless. Hence the service provision at HP and HC level enabled to provide fast, quality, and up to the standard. The private health facilities provide above-capacity treatment. They give medicines directly; which should be provided after the first step. But we provide step-by-step treatment according to the standard. The treatment starts at the HP, goes to HC if not cured, and so on. The advantage is not being exposed to strong medicine and saving on the cost of treatment. For example, the support with a chart booklet is not available at a private health facility. Because it guides standard step-wise treatment, following the appropriate step is very important.

01:39:35

I: Please explain to us the feasibility of this support/ IR for national scale-up?

R: I like it if it is scaled up. We saw the benefits and it was scientifically proved to have benefited the community. The community also had seen the benefits and approved them. I suggest it be scaled up to the national level. The

resource and supply can be solved. Especially the remote areas can benefit more than others. Because they are not getting adequate service. It is also helpful in this high inflation challenge.

I: what do you think can be an obstacle not to being scaled up to the national level?

R: I suggest eCHIS should be integrated with the digital module to provide the iCCM service electronically. We were committed and tried to fulfill the necessary materials. But is not somewhat familiar to provide medicines to the HP for free. Supply should be provided in line with scale-up. Our topography and whether a condition is somewhat better.

I: How are the activities/efforts embedded in the PHC and woreda routines/work streams?

R: iCCM as part of family health. Under-five children's health is not a new idea and needs no new system. But it was the weekend and hence supported by the project. The project had initiated the usual governmental activities. I cannot discriminate between it as a project and the other as a governmental system; because the project supported the formal service. But this time, it got special attention and support. We have a plan starting from the woreda base plan for the iCCM service.

I: Do you have anything think is important to tell us that we have not asked you?

R: this system was paid attention to because it is thought that it can save children from dying. We have to collaborate and work on it. We have to formalize the service everywhere. To do so regular support have to be secured.

End line evaluation of the PSBI implementation research (IR), the qualitative component

I	Identification	
1	Questionnaire ID	**Lume-Tade-PHCU-Vice Director-IDI-L&T**
2	Area Identification	
3	Name of Woreda/Zone/Region	**Lume Woreda**
4	Name of facility	**PHCU**
5	Name of moderator	
6	Name of a note taker	
7	Date of discussion	**08/06/22**
8	Participant #	**1**
9	Audio File #	**1**
10	Start time:	**_2__:_30_**
11	End time:	**_5_:_00____**
12	Transcriber	

Key: I: interviewer

R: responder

I: HI, my name is Temam and my colleague is Lalisa. We are from the JSI L10K project. We are here to gather information on the ICCM project for end evaluation. I thank you for your willingness to do this interview. Can you please introduce yourself and let us proceed to the next question?

R: ok, thank you, I am the HEW coordinator and deputy director of Tade PHCU.

I: Can you please describe the demand generation/SBCC activities you are doing on iCCM/ newborn health? What are the strategies used to raise awareness?

R: iCCM and CBNC services were interrupted in the middle; sometimes before. The awareness was also compromised due to the interruption. It was not going as planned and was stacked for a few periods. It was after that, that a given organization came to us and conducted research on it. Then they started identifying the reason why it was interrupted. They consulted public figures of the community. They conducted a meeting for discussion on the issue together with them, at the woreda level. They asked a question about the reason why it was interrupted. Then they provided a short-term awareness creation. Because the service was interrupted, the community was taking their babies to a health center, bypassing the health post. After the awareness was provided for the HEWs, they went to the community for awareness creation. They started to discuss with the community on any platform they encountered; like 'Afosha', where the community gathers for the traditional money-saving system. It is after that, that the community got aware of the health care service provided for the

infants. A given child, if attacked by local bacteria or any hidden pathogen or malnourished, and got treatment from the HEW using available resources, the mother is happy.

00:03:57

She tells her neighbor about the service she was provided. That helps the others not to bypass the health post. That enables them to get faster service immediately. Because the service was free, a lot of treatments were conducted within a short period. We encourage such a kind of service to continue; rather than retract. The HEWs get satisfaction while providing such kind of service. By appreciating themselves for providing such kind of service, they get motivated for further knowledge and skill. Hence, after the assessment, the once discontinued medical supplies were re-distributed. It means the HC also started to pay attention to the service. That is how the service was started again. Because the community prefers to take their children to other health care facilities than the HP, the health of the child can be in danger, up to death; due to the time, it takes to the other health facility. Working on the safety of these children guarantees their future. The service is a continuation of PNC; the assessment starts from there. Whether the mother is breastfeeding properly, the way the neonate is sucking, and the like are done both for the mother and the neonate. They provide the service home to home as well as at the HP.

00:06:27

I: What are the strategies you used to create awareness?

R: as I told you before, awareness was provided at the woreda level. Then they came to the community and trained the women well. They used the gathering for the model women training for creating awareness. The training was phase by phase and we were using it carefully. At the time we move to the community, we used the pregnant mothers' conference to disseminate the information. I or any other health professional who attended the meeting seriously disseminated the information about the iCCM service. At the conference, we don't tell them only to care for their babies in the womb, but also on the way how to take care of their neonates after birth. We told them that the HEWs can provide health care services for their children and the medicine that they can gate from other are also available at the HP for free. In addition to this, at the time there is a meeting for other purposes, we create awareness on that platform. We prefer mothers over fathers because it is a mother who spends the majority of the time with the babies. Most of the fathers are farmers and they spend the day in the field and come back home at the night. It is only at this time that a father can identify his sick baby. Mothers are staying with their babies for the 24hrs. That is why we are focusing on the mothers. We also get the women when they gather for 'Afosha', the traditional money-saving mechanism for the women.

00:08:22

For the HEWs to improve their status, we have a review meeting every month. After they deliver their report, we evaluate their performance, for example, regarding iCCM. Who took care of the neonate, who seriously checked the very severe diseases, and the like will be evaluated. Before doing that, we have a checklist by which we can

evaluate them every day. Even though it was interrupted for a moment, we restarted it. How many neonates less than 2 months and more than two months were treated by each HEWs is checked. At the time we supervise them in such a way, they become alert and become serious about their activities. It makes them strive a lot to accomplish this task. That means, that if she didn't report a single service of iCCM, she knows that should not be repeated the next day. Hence, during the review meeting, we analyze their performance, summarize and compare among themselves at the end of every month. The best performer is used for experience sharing. Whether she used the chart booklet or did it randomly. At the time she shares her experience on spot, which is a good mechanism to improve their skill. Because the under-five children focal person at the HC was also trained about the iCCM service, he also shares his knowledge with the HEWs. That is how we are sharing experience and knowledge and ensuring the progress of the service.

00:10:31

I: can you please share with us some of the good performances by example?

R: ok, for example, Bola Buta kebele is performing well. There are about 3 kebeles who are performing well in our catchment. The HEW who was assigned to Bola Buta HP started a good job from the beginning and was sharing her experience.

I: What was her experience?

R: for example, identification of sick babies, and health care service provided were her experience. Whether she used the chart booklet or not. The way she used the chart booklet properly or not. That was the experience she shared with the others. Jogo and Adada kebele HPs are also providing health care services for most of the children.

I: I think it should be their initiation for the iCCM service, which made them perform well

R: yes, that is one of the reasons. At some HPs due to the inability to identify the kind of disease, children go back without getting adequate service. That is due to improper use of chart booklet. Because the chart booklet is easily guiding the service to be provided. It orders to give something for the kind of sickness matching. Hence, due to the lack of proper utilization of the chart booklet, there was a time at which the children were miss diagnosed. That was what we identified while we were supervising them using the checklist. It is after that, we decided to let them share their experience; rather than providing our knowledge. To create a spirit of competition among themselves. Hence, proper identification, diagnosis, and treatment mechanisms were focused on during service provision.

00:12:55

I: how did the WDA participate in the awareness creation?

R: yes we let them participate. But they cannot understand what local bacterial infection means. Our most utilized strategy is the pregnant women conference. Because they are mothers of children and the ones who bear children. The one who is pregnant, at the time she sees a health problem in her baby, is informed to bring it to the HP. For

the lactating mother, the proper way of breastfeeding, and the signs, and symptoms indicating the sick babies, are told to her, so that she can bring the baby to HP. Symptoms like resisting breastfeeding, if there is a rash on their body, bleeding umbilical cord, and stiff neck, are the symptoms we tell them. These symptoms are listed on family health guidelines. We provide a guideline for the mothers and let them see the symptoms. At the time they go home, they consult their educated children for more clarification. That is the strategy we use. We show them the symptoms on the family guideline and tell them to bring the babies at the time they see those symptoms indicated in it.

00:15:09

We tell them that the symptom is an indication of bacterial infection. We also tell them to get a reading from their educated children at least twice a day. The educated children can also identify the symptoms of the babies and associate them with the symptoms in the guideline. The family health guideline book has everything in it. Regarding family health, child health, breastfeeding, what could happen after delivery, and so forth.

I: What are some of the biggest challenges with SBCC activities for newborn care?

R: a variety of traditional practices could be there; that the community practice personally or in common. There is a tradition called 'Hamachisa', the ceremony by which the infant is given a name. The mothers don't want to move out of their homes before this ceremony. But this is not significant at this time. It is about to vanish. At the time we consult them closely, they are willing to get the service. Because health is something that should be given a priority. There is no single case reported concerning that ceremony. We also provide the service for the referral cases from the HPs and send them back. At the time we supervise the HEWs using the checklist, there was no single report regarding such a problem.

00:17:56

I: so we can say the community is well aware of the iCCM service?

R: yes, perfectly, they got adequate awareness by this time.

I: there is no single challenge regarding awareness creation?

R: yes, there is no challenge at all. As I told you before, the training provided for the mother to make them the best mother had played a great role. Fortunately, the training provision and iCCM service initiation overlapped at the time. That helped to easily communicate the information about iCCM. The HEWs also got it easy to start the service. Those who were not seeing the iCCM register for about five years will be surprised if you check the register by this time. It is filled with the children who got iCCM service. They can do identification of sick neonates and under-five children by this time. Above all, the support of eCHIS is undisputable; I have no word to explain its contribution.

I: How do you describe the strength of the support you got from the PHC and the project as well as your efforts to address barriers to delivering iCCM/PSBI during COVID-19?

00:20:14

R: the support provided from the project was very nice and strong. We got supportive supervision at least three times. It initiated us a lot. They provided support, conduct an assessment about the contribution of the support and reflect the result to us. That helped us to see our performance. That was incredible. There was feedback provided. Federal and Oromiya regional officers visited us to check whether the iCCM service was secured or not. More than the woreda officers and the others, the support provided by the project was incredible. It is a good job; that reminded us of the once-forgotten service. Regarding the second point, about COVID, the health professionals cannot retract from providing service due to the fear of it. Just like what the defense army does, the health professionals also strive to serve the community up to death. At the time every sector closed its door, the health sector was providing service regardless of the COVID. We were providing service during the COVID time and we are doing the same now. There is no time when COVID became an obstacle. Because we provided awareness for the community; so that they can keep their distance and access the service. The way they have to use the face mask was also communicated. We enabled them not to keep their sick babies at home; due to the fear of COVID. There was no time when children are kept at home due to the fear of COVID. It was only at the time of the lockdown; that the HEWs were not providing PNC service. Because the family was not coming closer; let alone the others. But we had been through that challenge and arrived today. Hence, the community is not staying away from the service due to the fear of COVID at this time. We sacrificed ourselves for the service and ensured the continuation of the service. Even we were providing vaccination services at that time; taking preventive measures.

00:24:20

I: What was changed?

R: the first change was the initiation of the iCCM register for the service. It was functionalized from where it was thrown to dust. The second change was on the HEWs. They started the once-forgotten service. You can see the registration filled by the services provided at this time. We don’t want to see sick people; rather we want to treat them before feeling sick. Assessment of the health status of neonates in a given kebele is a change by itself. Having a recorded health status of these neonates is a big change.

I: how about the change in the community?

R: attitude change was observed in the community. They were undermining the HEWs that they could not able to provide iCCM for neonates. But, after they saw the HEWs treating the neonates, they started to say why we travel a distance, and why we pay for the same service, while the service is in our vicinity. Hence, they developed a trust in them.

00:26:53

I: Was the support you got from the project and PHC helpful?

R: we see the support in two categories. One is supported by the checklist and the other is material support. There is no material support provided. But regarding support on the job, they touched every HPs, the way they are managing registers. To ensure whether the HEWs are simply reporting the numbers or they are critically managing them. You can call it to job short-term support or mentorship or technical support. It is a detailed kind of support that helped the HEWs to develop their skills. These are the biggest benefits we obtained from the project. These are good assets for the future; which lead to a change.

I: What was changed differently?

R: the medicines were expiring in the hands of HEWs; because they were not providing the service. This is one change. Regarding the HC, zinc was not available since last year, even at the national level. We discussed it at the management level because the service should not be compromised due to a lack of zinc. It is boring for the mothers to be sent to HC for that service. We decided to buy it from the health care financing budget, from where ever it could be accessed. Because the service provided was increasing from time to time and because the community consumes any accessible water sources, we decided to buy it. we were admired by the project supervisor; because we fulfilled the zinc which is not accessible in the other places he used to supervise. We are also providing supervision supported by a checklist. We check how many infants were treated, how many neonates were treated, and whether they used the chart booklet or not. There might be mistakes in their service; we check them, sit together and provide corrections point by point. Because it is not only their concern, rather it also is related to us.

00:32:39

I: what do you suggest to be done differently?

R: the biggest issue I want to raise, even though it cannot be realized, is to reduce loads of HEWs. Because, at the time they are planning for one activity, another one comes side way and diverts them from accomplishing their plan. Especially this year, it is a surprise that I have never encountered a year full of the campaign. For example, we are preparing for the COVID campaign. At the time a mother comes to the HP with her sick baby, the HEW either one of two of them is out of the HP. either it is better to reduce their load or better to add more professionals. For four professionals assigned to one HP, if three of them are out of the HP, one can stay and provide the service. When a mother comes to the HP for the service and sees it closed, she might not have the money to move to the HC. She has to go back home and seek money for transportation to the HC. The HEWs are accomplishing tasks that could not be possible for more than five individuals; being in two. Contrary to that, they are not appreciated; but rather blamed.

00:34:59

She is expected to accomplish the 18 packages. Which is not possible alone. Regarding the iCCM service, I suggest not to quite the support; rather continue and scaled up.

I: Did you get the support system helpful (mentorship and on-site coaching methods, supervision, technical support, PHCU level PRCMM, etc.) to enhance the skill of HEWs?

R: they came to us last week. the support provided to us was not simply like the one used to be done by using the checklist. Rather it was a mentorship that was practically showing every gap observed. They enabled us to have deep know-how on the iCCM service. In doing so, they covered all the 7 kebeles in our catchment. Then we sat together at the HC and saw the gaps they observed with the HEWs. They told us the things we should fulfill for the HPs and so does the fulfillment on the side of the HEWs. Regarding the prescription of drugs and diagnosis, for example, regarding identification, if wrongly identified, the diagnosis will be different. They provided mentorship on these issues and forwarded the feedback to us at the HC. So that we can follow up on the corrections. We then included the points in our checklist and started supervising the HEWs.

I: is there training provided for the HEWs?

R: yes, they had trained the HEWs very well. Not only the HEWs but we also attended the training. Not only limited to that, rather they provided two up-to-date chart booklets for each; after the trading.

00:40:12

I: was the updating of the chart booklet took place after the mentorship?

R: yes, it was done after the assessment conducted by them. One of the problems raised by the HEWs was the lack of an up-to-date chart booklet. They were using the 2016 chart booklet. They provided us with a three-page chart booklet as an alternative; until the original one become ready for service.

I: how was the mentorship, onsite coaching as well as technical support improved?

R: the biggest issue is daily supervision of the performance of HEWs regarding iCCM service. That helps them to pay attention and develop their skill.

I: you mean it should be dependent on their performance.

R: yes, it helps them to improve their skill as well as pay attention to their daily activities. It ensures the continuity of the service. Because, at the time the community started to access the service by changing their attitude, retracting can cause a disaster.

00:42:28

I: How does eCHIS implementation help you with iCCM service delivery?

R: eCHIS, for me, is a good asset in the iCCM service. At the time one is sitting while the other is working hard, it is painful. I used to say to my friends that there should be one mechanism by which the good performer should be

identified from the lazy one. It should provide me with what I deserve. That is what I saw in eCHIS. We are moving towards that. It shows what I did in numbers. Previously, one can perform more than I do and vice versa. But we get similar efficiency; like 80%. eCHIS has a big input on iCCM service. It has a great contribution to the health care system. One is the absence of missing records. For example, the service provided for a given child today shows the ways of identification, case management, and prescription. In case the child has an appointment, it shows in green color, one of the three indicative colors. If the appointment is yet to come, it shows gray color. When the appointment approaches, it turns yellow. At the time of appointment is passed, it turns red. This enabled us to provide the service without any missing; dropouts.

I: how about case identification?

R: it has everything in it including case identification. For example, if the child is known to have a common cold, you select it on the tablet. Then it asks for the duration of the cold. If it asks for the breathing system you measure and record it in it. You are not expected to see the chart booklet here. For example, if for neonate, the fast breathing system is 60 and above, it immediately tells you that the neonate is sick with severe pneumonia. The system, for the good performer, is a good asset. Because it is a technology-supported system.

00:47:01

I: how about data quality?

R: it can simply tell you the kind of medicine that should be provided then after. It hinders you to bypass the next step. Unless accomplished the sequential requirement. It doesn't allow you to provide treatment for the children that should not be. Rather it tells to send the child to the HC as a referral case. That is what we call the quality of service. At the time she sends the child, it directly links to the Tade HC. Because every site is linked to its respective HPs, it directly goes to the one linked to it. The HEW provides advice about the signs and symptoms observed in the child and tells the mother to take her to the HC for better service. This was communicated previously during the awareness creation. That is why a mother is not confused or hesitant about the service. hence, the service quality is unquestionable.

I: that is about service quality, how about data quality?

R: yes, it is perfectly keeping the data quality. It provides both service quality as well as data quality. From the beginning, the neonate is recorded in the family folder. Even though it doesn't give a name, because it is provided on the 45th day, it includes in the family folder by writing 'baby'. For example, my neonate is recorded in my family folder as 'Baby Tesfaye'. If you commit a mistake, the eCHIS tells you that the child is not recorded under the specified folder and claims for the correction. It forces to search for the neonate family and record accordingly. Hence, at the time wrong information is provided, it hinders proceeding.

I: how about the identification of sexes for the neonate.

R: it is possible, that only age and name are not identified before the 45th day. Rather it is recorded under the mother during PNC. That is how the application is created. At the time the mother is dispatched from the HC after delivery, they send an action card to the HEW. At the time the HEW allows the neonate to be included under the mother, the weight, Apgar score, sex, and every information will be retrieved. They simply synchronize the information and hence the record is entered into their eCHIS by date. Hence data quality can be seen here. Clean and error-free data will be obtained by eCHIS.

00:52:44

I: how about exchanging records for males and females?

R: there is no such mistake. Maybe in the GMP, the z score is different for males and females. If the male z score is needed, it brings that of the male and so does for the female. It cannot allow you to escape any step. At the time you escape sex, it forces you to fill it before proceeding to the next. The child of one cannot be given to the other. For example, if one is expected to be sent from Kolba, but sent from Bola, at the time she clicks on the action card, two issues are there. One is whether the mother is found in her list or not. At the time she clicks ok, if it is wrong, it orders to fill the household number for this mother. Then she has to deny the acceptance.

I: what is the language of instruction?

R: it is in three languages. Afan Oromo, English and Amharic. One can choose and favorite language. It is just like we can change the languages on our smartphones.

I: what do you think should be strengthened?

R: we were in discussion together with project leaders. We rose the idea about the individual level performance evaluation. We have to identify the well-performing from the other; to promote the good performers. There are issues in the checklist that are not applied by the HEWs; yet are expected to be filled during our supervision. Rather than simply writing no...no… no for these activities, it is better to omit them. For example, delivery is not done at the HP, but it is part of the evaluation checklist.

00:56:36

I: How was the introduction of the iCCM/CBNC module into eCHIS affected the iCCM/CBNC service delivery?

R: the introduction of the module into eCHIS is good input. At the time they move for a home visit, they are not worrying about carrying the chart booklet. It is found on their tablet. Hence they can decide the fate of the sick child they encountered. Whether it can be treated on spot or should be referred to the HC. The ability to diagnose the child on spot, rather than appointing them for the next day is a good input by itself. The other is, rather than opening the chart booklet to read, it is easy to check on the tablet. Because the community considers the one who is referring to the hard copy as poor knowledge. At the time they refer to the tablet, it is well coming for them; because the community considers that the service is assisted by technology. The biggest advantage of the

introduced module is to save time. It can help in identifying the case and hence quickly reach treatment. During PNC, it is not the mother who should come to the HP, rather it is the HEW who should go and provide the health care service. At that time, she can see the health status of the neonate, whether it has a feeding problem, local bacterial infection, stiff neck or umbilical cord bleeding, or some other else, using the table. She can refer to the module on the tablet and provide the necessary care and advice for the neonate, in an easy way.

01:00:33

I: how about the referral case management, appointment, or follow-up?

R: after assessment, we might provide an appointment. Then we check the improvement with the child. They can do that on phone or in person. If the child is not improved, they refer to the HC. The referral system is not simply random; rather it is included in the module. It has a note-writing place during the referral process. You can write the reason for the referral.

I: how about the feedback?

R: the big issue lies there. The HEWs are not able to retrieve information about the children who got treatment with them on the tablet. We told them to be improved. For example, if five children were treated, the information about these children is stored on the server; not in a separate file on the tablet. If the case was about EPI it includes under that or screening if the task was like that. But the children who were treated by iCCM should be available separately. This forced the HEWs to record on the hard copy as well as on the tablet. They need to have a backup. At the time the children had taken the necessary treatment and recovered, their data was removed from the follow-up and stored in their respective treatment listings. The number of PENTA provided can be retrieved; not for the number of children treated.

I: how about the referral cases?

R: we accept the referral case in two lines. One is paper-based and the other is from the tablet. The paper referral is simply to convince the mother that she needs to move to the HC for the service. After the treatment provided at the HC, the second follow-up is at HP not at the HC. For example, they send back the ANC service after ANC1; because the HEWs are the ones who continue the 2nd and 3rd ANC. Hence, at the time they click on the action card, the treatment information provided at the HC will be displayed on the tablet. That is how the follow-up proceeds.

I: How the client satisfaction should be obtained?

01:05:52

R: it is only at the time they meet with the HEWs; randomly. Otherwise, there is no place for client satisfaction on the tablet. I like it if this is included in the tablet. Because it is a good way to evaluate oneself performance.

I: What are the changes observed in the community's attitude, due to the service supported by eCHIS and the included module?

R: at the time they see the shift from paperwork to the tablet service, the community considers that there is an improvement in skill. That is a big change. Because fingerprint is taken from the mother and children above two years of age, they realize that their children are recognized before the eyes of government bodies. That they are worried about the safety of their children. We are seeing a big change by this time. We used to treat about 3 children in a year. But now, after we took the training and started providing iCCM service supported by tablet, we are providing an astonishing service. Our skill was improved greatly and so does the HEWs. Everything is recorded on the tablet. We are providing the vaccination service by tablet, the screening service is about to be included, and GMP. For the time being it is the iCCM service as well as the EPI service, which are being provided by the tablet. This has increased the acceptance of the service in the community.

01:11:04

I: what do you think should be improved?

R: the data should be retrieved from the eCHIS application. Because the HEWs should see what they did. It is quite different when checking the monitoring activity by oneself and when it is checked by another body. The second one is that there should be a place in the eCHIS application where the HEWs should write a note for themselves. Because it is a good input for research. The one I said before, that the service not provided at HP level is recorded as no, should be replaced with NA. That is applied only to the second generation. Because every service will be provided at the HP at that time. It is boring to record 'no' every time supervision is conducted.

I: What are the critical factors affecting the **delivery of iCCM** to clients?

R: there was a time when the HEW is challenged by the procedure. It was not due to the tablet but due to not reading the guideline properly. She chose the coughing baby as if it is living with TB patient family member. She was confused in the middle and consulted us for support. Then I told her to go back and revise the procedure. Finally found the mistake that she chose the option of the child living with a TB patient; wrongly.

I: is this due to a lack of skill or knowledge?

R: no, it should not be due to the skill gap; rather because they sometimes overlook the procedure. Otherwise, they took the training at least twice. The tablet they have at hand is very fast; a single touch can lead to the wrong place. She was expected to critically read the procedure and should take the history properly. Otherwise, they were practicing, at least for five days, during the training. There is no gap in their skill. If I say there is a gap, all the great deal of iCCM service could not be done.

I: but what are the major gaps observed in their skill?

R: the first gap is readiness. The other is patience for detailed references on the tablet. Sometimes they might forget the constants of their training. Because there could not be a common understanding among the HEWs. One can apply it without any push; while others may need pushing. To resolve these, it is better to provide at least

monthly orientation. we are the ones who should play a role in this regard. Hereafter, our review meeting should be done at the HP level, not at the HC. That is how one can share practical experience.

01:21:46

I: are all of them at level 4?

R: only one or two are remaining.

I: hence the newly coming HEWs cannot participate in the iCCM service?

R: no, anyone who can handle a smartphone can use the tablet and provide iCCM service. Nothing is new and challenging in it. Because they expect some orientations, we can fulfill that gap. I can share with you my experience, that I managed about three referral issues on behalf of the absent midwife professional. The supervisors identified it by checking the records done on that data in the table. They appreciated me a lot. Its content is exactly similar to the one in hard copy; except being included in the tablet. The newcomers can share the experience with the senior ones.

I: How was the supply shortage affected the iCCM service?

R: health care financing is used for the fulfillment of the supplies. We buy them from the budget allocated, in collaboration with the distributor called EPSA. Our big challenge is Gentamycin. It is not available. We have to dilute it and wastage happens. We cannot access the diluted supply. The other shortage weight scale. We hang the child in the case, bucket, or other available material; out of the standard. We repeatedly reported it, yet no one provided it. But it is very essential for iCCM service. It is hard to prescribe medicine for children without measuring their weight.

01:29:51

I: is there another issue that affected the iCCM service?

R: no, there is no single reason; except the one, I mentioned before. Regarding awareness creation, we have a plan to provide training for the community. Because the WDA is said to be restructured. Except for our commitment, there is nothing that can make us retract. We must save the children. The training provided in every direction, for example, ART and TOT, can also support them. Hence there is no challenge.

I: how about security issues?

R: this area is secured. There is no challenge regarding security. The northern part conflict could affect inflation. We used to buy a track full of medicine for 5000 birr. But now, we are not able to buy a bag of medicine with that amount of money. The cost is increasing while the demand is also increasing. In the absence of the community health insurance service, the community couldn't have come to HC for accessing the service. The HC may face

closure, in the absence of health insurance. Because they might prefer the private sector; if there is no difference in cost.

01:35:31

I: How do you think the COVID-19 pandemic and/or COVID-19 response measures affect the uptake and/or delivery of iCCM services?

R: using preventive measures like a face mask on both sides, we enabled the community to access the service. They are forced to wear the mast starting from the gate. That is the cause for the awareness created.

I: how was the iCCM service affected due to the pandemic?

R: no effect happened due to the pandemic.

I: it could be on the routine activity or service delivery; what is the impact of COVID on iCCM?

01:39:05

I: the gap was observed only when the lockdown was announced. That was the only time that the community was not able to come to the HC. The only service we used to provide at that time was delivery service. That has happened because it was spontaneous. Otherwise, we created awareness on how to prevent the disease and started providing services.

I: How does it interrupt the livelihood of the community?

R: that was obvious. Because body contact was dangerous, the children were not allowed to be touched. There was great trouble regarding access to health facilities; because the transportation cost was also doubled. But we had been through that obstacles and reached today safely.

I: Explain to us any changes you observed as the result of the implementation of the COVID-19 adaptive iCCM implementation strategies?

01:43:03

R: Currently, there is no fear of COVID. We are in the adaptation phase. But we had learned a lot from the post-pandemic. Because we had initiated the iCCM service from where it was forgotten, reinitiating again cannot be a challenge in this post-pandemic time.

I: what are the COVID-19 adaptive iCCM implementation strategies?

R: it was by creating awareness. It was after the lockdown, that the community started to move and get information about the service. We initiated the HEWs and they in turn used the available structures to disseminate the information. The HEW provide vaccination service for the vulnerable group of the society by using the list they have at hand. That is how the community became aware of the service initiation after the lockdown. The COVID 19

vaccination service also initiated the community to be confident about the disease. There is no new strategy developed to reinitiate the iCCM service. We were supported by the insignificant impact form the disease. Because the service can be accessed only by using a facemask. There was no service interrupted even during the lockdown.

I: hence the lack of COVID adaptive implementation strategy was due to the insignificant impact of the disease?

01:49:59

R: yes, the big issue was that only a few individuals were quarantined or dead in the town. Nothing was observed in the countryside, where the HP is found. The absence of health impact from COVID was a good opportunity for us to continue the service. Had there been a victim, the community could have resisted accessing the service. There was a time when my colleague mistakenly admitted a COVID suspect man and the clients wanted to be discharged due to the fear of it.

01:53:16

I: can you tell us the specific change in the iCCM service during the adaptation phase of COVID 19?

R: we are considering as if there is no COVID at this time. We have to give priority to health care services. we are giving priority to the children. Even the use of a mask is almost neglected. Hence, there is a practical change regarding the mothers taking their children to a health care facility; regardless of the COVID pandemic. They sometimes ask if the COVID is still existing. They are also giving priority to the health care service of their children. COVID is considered only by those who are educated. They are getting treatment for their children, they are accessing vaccination for their children, deworming is accessed and so does vitamin A for their children. For example, at Adada Dambala kebele, there is one child admitted for malnutrition at this time and accessing treatment. It indicates that they are developing trust in the HEWs and giving priority to their children.

01:57:01

I: What are the high-level benefits that are attributable to this support/IR?

R: after iCCM was initiated, our awareness increased. We started considering it as the main activity; from the once considered forgotten. So does the awareness of HEWs. At the time we are supervised for these activities, we understand that attention was given and strive to improve our performance. Had there no repeated campaign, we would have done even more than this. It also enabled us to pay more attention to the supply. At the time they come for supervision, they might see what is not available here, while it is in the other place. This can make us strive a lot to suffice them. JSI is the owner of the new iCCM service. It is facilitating the service by supporting eCHIS and integrating it with CBNC. The attitude of the community is changed. They realized that the HEWs can treat neonates and children. Hence, the safety of their children and saving children from dying is the benefit. The

mother who used to suffer by moving long-distance, paying the extra cost, and suffering in the queue at other places was provided the same service in their vicinity.

02:03:13

I: Please explain to us the feasibility of this support/ IR for national scale-up?

R: yes, it is possible to scale up, surely. The professionals have to accept the get prepared for the service. As long as they are ready for the service, it is possible to lay the base for iCCM service. Topography can be a challenge. The community may not accept it all at once. As long as the assessment is conducted and awareness is created well, there is no reason why it cannot be scaled up. At the time one child is treated and relieved from the disease, the others can understand the service.

I: how about harmful traditional practice?

R: that can be an obstacle, but closer awareness creation has to be done.

I: how about a resource?

R: access to a tablet might be challenging; because the high capacity one is very costly and it is hard to buy it in a larger quantity. the former tablet was very old and hard to use it. The Oromiya region had little contribution to the iCCM. The resources are directly distributed to the woreda. The region has to take part, at least by fulfilling the manpower.

I: is it easy to scale up to the regional level; while the region contribution is a little?

R: yes, the region knows about the iCCM service. But their control over the activities was minor. Otherwise, they can fulfill the manpower or the tablet or security issues.

02:10:18

I: How are the activities/efforts embedded in the PHC and woreda routines/work streams?

R: during iCCM supportive supervision, the federal, regional, and woreda family health teams are coming to us in collaboration. They randomly chose the HPs and supervise them. Then they come back to the HC and we discuss the issues. Hence, the chain is lined in that way, the JSI staffs join sometimes.

I: How does the woreda take over the activities and incorporate them into the annual plan for the evaluation of performances at the HC and HP level?

R: the HC plan and do the activities; so does the woreda. They use their checklist to supervise us and the rest of the activities; including iCCM. They are supervising the family health; iCCM is one of them. It is a must to supervise the performance; otherwise, the endeavor to provide iCCM service cannot be successful. I am the HEWs coordinator and together with HMIS focal person, because eCHIS is more responsible to him, are supervising the HEWs in

detail. We have an organized checklist that incorporated the iCCM service. We are given responsibilities and we have to address them to the end. Our focus is on the children who are going to replace us.

I: hence you are saying iCCM service is a planned activity?

R: definitely.

I: do you suspect it could retract in the absence of the support?

R: never, this is our regular activity. It should not be considered an activity that a given organization initiated for the first time. The JSI is here to restart the activities. Children could come for vaccination, assessment can be done at that time. PNC is provided for the mother and the neonate can be assessed at that time. Previously the professionals were trying to check only the severe diseases and local bacterial infections. They neglected the malnutrition. That is why the feeding problem issue was included in the tablet.

I: what do you think should be done not to lag?

R: strong supportive supervision should be done. The daily feedback collected can become a monthly report. At the time 10 services were provided, 10 reports, 10 registers, and 10 iCCM registers are expected.

I: PMT does not evaluate iCCM services in aggregate, rather it considers the services separately. Don't you think this can limit the quality of iCCM service?

R: the fear is real and correct. But the iCCM service can be reported to PMT.

I: was it done up to now?

R: no. but you showed me a good point and I can report it in such a way. I collect reports every Friday, from the professionals supervising the HP according to their schedule. We can evaluate the number of under two months who were treated, how many local bacterial infection was treated, how many very severe disease was treated, and how many feeding problems were treated. The number of above two months infants treated for diarrhea. This is done every Friday. I can guide them to supervise the HEWs accordingly. Then the monthly report can be done in the presence of HEWs. The under-five focal person should be in line with that; has to take the responsibility.

02:21:32

I: because it is part of the DHIS2, the reports of iCCM can be done. Every activity on DHIS2 has to be accomplished. That is how the efficacy of their performance can be seen and hence used for comparison. We can see the reports graphically and show the HEWs so that they can be motivated based on their performance. Hence there should be no fear of iCCM service retraction.

I: thank you for your time, do you have something to add at the end?

R: I can ensure that the activities of iCCM are a part of the regular health care service. it was stacked in the middle. But now, because it is restarted to a better level. There is no way that it can retract. Because we were not paying

attention to the activity. Now we can do that. It was stacked because the activity was not taken over from Save the children. Now we took over and started to improve the service. I like to thank you for your endeavor and came to us for consulting us. I like you scale it up to the national level. It should not be limited to us. Either the region or the federal should support the supplies. If inflation continues at this rate, the HC cannot survive. Those HC constructed 2000 E.C and afterward cannot survive, because they didn't develop a backup. Except there is compensation from the higher level, it is hard to survive. You can imagine a cost of a given packet of medicine and when that is provided to the clients for free. If there is no compensation. I recommend you focus on the supplies. The weight scale should be provided; if the standard should be followed.

I: Thank you for your time and valuable information.

I	Identification	
1	Questionnaire ID	T03
2	Area Identification	
3	Name of Woreda/Zone/Region	Lume wored
4	Name of facility	Health Post
5	Name of moderator	
6	Name of a note taker	
7	Date of discussion	11/06/2022
8	Participant #	one
9	Audio File #	07
10	Start time:	4:32
11	End time:	5:08
12	Transcriber	

Interviewer :- Can you please describe the demand generation/SBCC activities you are doing on iCCM/ newborn health? What are the strategies used to raise awareness?

How is the engagement of communities/WDA networks in the implementation of integrated community-based case management (iCCM) of common childhood illnesses/possible serious bacterial infection (PSBI)?

Respondent :- before JSI/L10K supports our attitude and skills were very low to treat sick infants of above two months and below two months . but after we took ICCM training we believe that we can treat sick infants well and demonstrated with evidences .then we inter to the communities to increase their awareness based on what we obtain chance on different conferences,on different meeting ,at school students ,at Health post, during home visits and every where we obtain the chance specially promoting on sick children under two months can treat at Health post and at home level by Health extension workers. likewise we actively requesting the health centers for sufficient drug supply to treatment sick infants at health posts.

We used strategies to increase awareness of the communities by kebele structure like 1 to 5 ,on pregnant women conferences ,religious leader specially at church and mosque , Aba Geda, during EPI Vaccination times, distribute my phone numbers for any question and consultations on iCCM Services .

During the Pregnant Women conference conducts we aware on the danger sign and symptoms of infants and mothers. we also educate them on child health, causes of child death, on how were infants becoming infected by pneumonia ,aware on mother and child hygiene after this activities we see many changes on Child Vaccination ,care and improved health seeking behaviors.

since this sites far distance from the town or biyo Health centers ,the communities have not had chance to go to there and the transportation prices is also very expensive to go biyo HC and mondjo town so they prefer to treat their infants .

Interviewer :- How do you describe the strength of the support you got from the PHC and the project as well as your efforts to address barriers to deliver iCCM/PSBI during COVID-19?

Respondent :- During COVID-19 started in our countries the people were very fright and the health services is interrupted but after all of us adapted the COVID-19 infection we start to work our activity routinely .

regarding to different support I would like to acknowledgment to JSI/L10K to give us this phone tablet to simplify our burden of activities in modern ways and the other is provide Training on integrated community-based case management of common childhood illnesses treatments supported by phone tablet to perform our activities in short method.

after we took training we simply treat the sick infants in the communities and refer to Health centers actively gate feedback from Health center in modern ways ,this phone tablet avoid to carrying paper based recording materials and this makes us to got special knowledge on our jobs .

Interviewer :- Did do you get the support system helpful (mentorship and on-site coaching methods, supervision, technical support, PHCU level PRCMM, etc.) to enhance your skills over the traditional off-site training and woreda level review meetings?

Respondent :- yes we got so much Supports like training on ICCM and CBNC ,refreshment training , mentorships related to how to treat infants by using phone tablets, how to identifies cases based on sign and symptoms, how to prescribe the drugs for sick infants .

JSI/L10K provides for us supporting manual to refer for additional references .

JSI/L10K coordinating with Wored Health office ,Regional Health Bureau ,Even with Federal ministry of Health supervised us on ICCM/CBNC two times and from JSI/L10K only they come to us many times for Mentorship,Coaching.

To say really before system supports I haven't had self confidence to treat infants even when the societies bring sick infants to health post I denied to treat ,only I had refer to health center,even if when the community bring moderate sick babies I fear to prescribes the drugs and no self confidences to treat sick infants . but after we got support from JSI/L10K like ICCM Training ,Supportive supervision and on job mentorships I have better self confidence and improved competences to treat sick infant and the people were also give us good respect on our recent knowledge and practices .

After system supports the daily communication and referral system between Health post and Health centers has been improved particularly on sick child treatments ..

JSI/L10K come up to house hold level to see services we delivered and they investigate us how we are working according to guideline and how to follow phone tablet steps to investigate a cases and to treat identified infants.

***In*terviewer :- How eCHIS implementation helps you with iCCM service delivery?**

Respondent :- eCHIS implementation helps us for example if one mother delivered at health centers and post natal started there and she referred to us by eCHIS, during home tome visit we investigate mother and her babies

particularly umbilical access , their weight ,their respiratory system simply by using eCHIS phone tablet and I can manage iCCM service at there .

With the guidance of phone tablet and Booklet charts I can identifies the cases, provide the correct drugs with their dose,time interval to took,item of the drugs for example if the tablet ask me the weight amount of the infants then relating to babies weight the tablet itself order me the amount of amoxicillin doses ,for such amount days , based on the case I have been filled the phone tablet answer for me that this disease categorized to this infection if pneumonia medium pneumonia , if pneumonia with bleed categorized to sever pneumonia this show that a phone tablet with booklet chart guide me to treat any bacterial infection. Even if I made a treatment error the eCHIS phone tablet correct me this also improve the qualities of our activities .

the other eCHIS benefit is if we move home to home we will fill the post natal follow up of mother and her child's appointed for next appointment the phone tablet is show me the appointment date if the appointment date is near future the tablet show to me by different colors, if the appointment date is near to date it show gray color ,if appointment dates left for two day it shows that yellow color,if the appointment date is passed the tablet shows that red color this alarming us to trace a defaulter from appointment dates this make us to do every activities in simple and good manners .

Interviewer :- How was the introduction of the iCCM/CBNC module into eCHIS affected the iCCM/CBNC service delivery?

Respondent :- iCCM/CBNC module has big in put for eCHIS services because for example if one infants faced with sever and complicated problems I refer iCCM/CBNC module and execute eCHIS phone tablets step by step it order me this infants should be refers to health centers with them case investigated and case severity .

This module guide me on children from two month up to five years and infants less than two months .

during we treat sick infants of zero up to two months we only guided on iCCM/CBNC module to identify the case and to make a decision for referral for further investigation .

iCCM/CBNC module categories who need treatment and follow ups at their home, who need treatment at health posts and for next appointments to be came to health posts.so the activities done by using iCCM/CBNC module improve our performance the communities also very happy with treatments of their infants by supported on eCHIS phone tablets.

Interviewer :- Describe the main issues faced by the health system to identify and treat neonatal infections in the community. What are the critical factors affecting the delivery of iCCM to clients?

Respondent :- our Health center has not had a problems what they had on their hands . but related to drugs we have a problems on the shortage of gentamyccine drugs.

As a factor during we move home to home visits some people were denied to show and to touch their infants relating to religion ,culture and habituation if we contacts with a such people we educate them and aware on iCCM services.

Interviewer :- How do you think the COVID-19 pandemic and/or COVID-19 response measures affect the uptake and/or delivery of iCCM services?

Respondent :- during COVID-19 time we stops our activities at home to home visits and child treatment at home because we fear COVID-19 infection , this minimizes our activities specially on integrated community-based case management of common childhood illnesses the community is not come to health post and they also fear us to came to their home for this iCCM Services were highly interrupted not only this their daily activities also highly affected.

Interviewer :- ***Explain to us any changes you observed as the result of the implementation of the COVID-19 adaptive iCCM implementation strategies? – What impact has the strategies had? What are the particular features of the strategies that made a difference?***

Respondent :- at present time the community is adapted with COVID-19 infection on how to obtain the services , without any fearing the communities come to health post and gate any services mostly child immunization ,treat sick childs.

all people aware on COVID-19 infection, prevention and control methods ,

During COVID-19 time no system supports but after adapt COVID-19 infection we see big changes from Woreda and health centers on Supportive supervision, coaching and mentorships on iCCM services deliveries.

The community participation and social mobilization on iCCM services delivery were increased and normalize as to before COVID-19 Pandemic happening .

Interviewer :-What are the high-level benefits that are attributable to this support/IR?

Respondent :- we obtain sufficient training on ICCM/CBNC ,eCHIS based services itself is very good benefits because we simply record,treat,refer and report by phone tablets ,Child death will also decrease.

our skill and competence were improved ,all communities were highly aware on child health and improved child health seeking behaviour .

Interviewer :- Please explain to us the feasibility/practicably of this support/ IR for national scale-up?

Respondent :- work done there by using phone tablets is a big changes for me, I expected that if this support is scale-up to other region they can obtain what we gate benefits from this supports easily by incorporate with the existing health extension packages.

Interviewer :-How are the activities/efforts embedded in the PHC and woreda routines/work streams?What implementation strategies are incorporated with the PHC and woreda annual work plan?

Respondent :- we prepare ICCM annual work plan based on our data we have on our hands integrating with health extension packages.

Interviewer :- Do you have anything think is important to tell us that we have not asked you?

Respondent :- we did not have finish our activities and we try to improve as much as possible our gaps .I would like to say JSI/L10K organization have to continues their supports for the future on fulfil human resources gaps, expand eCHIS technologies for all health extension packages.

Interviewer :- thank you very much for your time and response to my question

Respondents :- thank you too.

I	Identification	
1	Questionnaire ID	T05
2	Area Identification	HCs
3	Name of Woreda/Zone/Region	Lume wored/ East shoa Zone /Oromia Region
4	Name of facility	HCs
5	Name of moderator	
6	Name of a note taker	
7	Date of discussion	16/06/2022
8	Participant #	one
9	Audio File #	016
10	Start time:	2:31
11	End time:	3:58
12	Transcriber	

Interviewer : - Can you please describe the demand generation/SBCC activities you are doing on iCCM/ newborn health? What are the strategies used to raise awareness?

How is the engagement of communities/WDA networks in the implementation of integrated community-based case management (iCCM) of common childhood illnesses/possible serious bacterial infection (PSBI)?

Respondent :- for strengthening of demand creation on ICCM services. first HEWs and Health Care workers took ICCM training, after that we assigned to every Health post and kebele ,then we discuss with all kebele structures on benefits of ICCM services , the knowledge and capacities of Health extension Workers could treat sick infants at Health posts and to increase the communities beliefs and respection for them.

First We call the leader of Women developmental army and trained on ICCM then cooperation with kebele structure aware the community on HEWs could treat sick infants at health posts and promoting the services provision because they expects HEWs couldn't provide child treatments .

By using WDAs and kebele structure like Kebele Manager, kebele administrator ,one to five leader ,religion leader in church s , educate the people on at present time ICCM service is provided at health post level with its benefits of services.

After we make demand generation on iCCM services our infant treatment performances like pneumonia and diarrhea is on better improvements , before support infant pneumonia and diarrhea treatment performance were not more than 5%-7% but after we aware the communities our infant treatments were around 30%-50% at Health posts .

Related to Pregnant women conferences by monthly at every health post we have a meeting and cumulatively conducted at Health centers especially during child vaccination period and at school level we aware School director and Biology teacher then they aware students and different clubs in school ,because every students come from different community and when come back to their family they aware their family and communities on local bacteria infection on infants ;especially, at Bali Abo kebele the people asks us additional services because the residential site is far from HCs and far from the town .

Not only this our health center is Sever acute malnutrition treatment centers and we had different videos and pictures on different time we recorded and we demonstrate this Video during pregnant women conferences. by this year after we create awareness, we treat children on Sever acute malnutrition and these children recovered from a problem,they are very fatten and happy then refer back to health post.

Interviewer:- What are some of the biggest challenges about SBCC activities for newborn care?

Respondents :- after we mobilize the communities and start the service we had faced a some problems,from this some person is not belief ICCM services provided by HEWs, the other challenge is the community ask laboratory investigation for diarrhea and pneumonia if not gate the services they went to HCs.

The population residence were disperse and the HEWs couldn't reach for all House hold in short period of time this might be leads them for not timely provide the services .

Some people were no apply umbilical cord lubricating that provided from HCs they use like butter on infant umbilical and some people are couldn't show their infant to HEWs because cultural problems.

Interviewer:- How do you describe the strength of the support you got from the PHC and the project as well as your efforts to address barriers to deliver iCCM/PSBI during COVID-19?

Respondents :- to say truly before system supports by JSI/L10k the ICCM Activities were forgotten and neglected activities, we also only focused on child treatment at Health centers not at health posts. before three year this activities is total collapsed because no one focused on ICCM Activities but after JSI/L10k project come and provided training for HCWs focal person and HEWs ICCM Activities proved at health posts and our performances improved. for example; before JSI/L10k support pneumonia and diarrhea infection treatment performance were 8% -9% but after JSI/L10k support treatment coverage is 54% as the whole under our catchment areas.

Related to drug supply, during shortage happen we communicate with JSI/L10k and they actively phone call to Ethiopian pharmaceutical Supply Agency (EPSA) to avail drugs, like; ORS,Zinc and Gentamyccine after that some what the problems is solved. most of time They discuss With our Health workers and HEWs to strengthen ICCM Activities ,JSI/L10k provide a training repeatedly more than three time for HEWs and for focal person.

This support is helpful and very essential because it is focused on sick infant treatments at all health post, provided by all HEWs and focal person Health care workers at Health centers this make us to see ICCM Activities from initial and the communities were extremely aware on ICCM Activities provided in Health posts.

More than three time again and again they provides for us supportive supervision up to health post and interviewed with HEWs on their jobs, observes how to ICCM registration is used and asses the quality services.

Based on our performances we have seen big changes, before system support several health posts were not provide ICCM services, even during baseline assessments was conducted we identified that some health post stopped their ICCM services but after we got support we changed our attention to provide on ICCMs services and we decrease child death and improve ICCM performances.

Interviewer:- Did do you get the support system helpful (mentorship and on-site coaching methods, supervision, technical support, PHCU level PRCMM, etc.) to enhance HEWs' skills over the traditional off-site training and woreda level review meetings?

Respondents :- on supporting system for many times JSI/L10k gone up to HEWs provide mentorshps on how to HEWs provides ICCM services, referring the ICCM registration ,assessing on is there knowledge gaps or not? and then they inform us which health posts had a gaps and based on that we also work on it .

in generally our HEWs are at now time they are very alert. one time JSI/L10k coordinating with Oromia Health Bureau supervises three health post ,based on supervision they assess infant treatments from registration then trace up to home level, interviewed babies mother and they are very happy and satisfy to gate treatment at health post .

Practically mentorship ,on-site coaching, supervision, technical support has differences from off-site training and review meetings because mentorships is based on what HEWs had actual done on their registration if a gaps mentee demonstrates how to works and correct their gaps .

After we start ICCM services specially ,like Bali Abo kebele which is far distance from HCs 20 KM, the communities were ask us additional treatment services for all people and also their acceptance for HEWs were increase.

During technical support we have seen many gaps at HEWs level particularly on children screening who has sever pneumonia and moderate pneumonia ,on correct respiratory rate recording and measuring ,some time under one month and under five year registration interchangeable used but after extensive support were provided we have seen a changes.

Interviewer:- How eCHIS implementation helps you with iCCM service delivery?

Respondents :- eCHIS implementation helps a lot on iCCM services when HEWs inters sick infant assessment history to eCHIS Phone tablets it guide them by ordering do this as guidelines, eCHIS system is very strong and HEWs used as reference.but the problem is that eCHIS is not generate a reports, it is not show activities they

performed at the end of months. eCHIS shows appointment scheduling, they collect reports from hard copy registration .

eCHIS has quality on infant treatments, HEWs identifies babies problems when one mother brought her child to health posts then inter to eCHIS apps after that the eCHIS apps asks about the problems if she answer yes for question the phone tablets identifies the problems then asks for the next question if the child is under sever it referral to HCs, after that those children come to Health centers and they got with good quality treatment at HCs, for example Five children who treated malnutrition at Biyo HCs is come here by eCHIS referral systems.

eCHIS brought for us iCCM data quality , even if we couldn't do system assemble but we do many things after data or services inter to phone tablets ,services one mother to gate in next appointment date should be scheduled , this is alarms HEWs by color coding. For example if one children infected by pneumonia and enter data to phone tablets then it appoint the next service date in two or three days ,if this children was not came the phone tablet show red color at that time HEWs have to phone call to babies family or visit babies home and provide the services.

eCHIS on iCCM service data managements is difficult and need improvements if one HEWs perform child treatments for 20 children, we only obtain the data from registration not from phone tablets. When we see Under five,ANC1,ANC4 and EPI Services phone tablet show all services provided with their number and period but for iCCM services it only shows for appointment schedule dates.

Interviewer:- How was the introduction of the iCCM/CBNC module into eCHIS affected the iCCM/CBNC service delivery?

Respondents :- it is correct to incorporating iCCM/CBNC module into eCHIS especially for child treatments , before incorporation to eCHIS Health extension workers refer hard copy iCCM/CBNC module but at present time simply she refer from phone tablets even she can cites during child treatment .if HEWs used iCCM/CBNC module she can got good benefits because it improve her knowledge ,skill and competencies , thus incorporating iCCM/CBNC module into eCHIS make jobs in simple ways, in short period of times with correct ways and refer for every case she observed on the infants then provide correct treatments for infants.

For referral system iCCM/CBNC module is very good at earlier time all referral system integrate with MCH ,but after under five children enters to CBNC separately and focal person trained on referral around 16 children were referred from health post to HCs.

Referral linkage immediately sent referred person from health posts and action cards is prepared before child arrived to under five years focal person at HCs and then if referred child delayed Focal person phone call to the mothers,even when she went to modjo focal person call her and returns her back to biyo health centers .and provide treatments for children's.

Even though we didn't assess client satisfaction by using checklist but mother who carry their child and came to HCs ,particularly for Sever nutritional treatment are very happy when they treat their children free cost and see their well babies .

incorporating iCCM/CBNC module into eCHIS improve HEWs confidences and knowledge because it is states clearly for any disease on case identification,correct treatments for sick infants and for case referral, for next appointment date schedules, even to trace when they lost to follow ups.

Interviewer:- Describe the main issues faced by the health system to identify and treat neonatal infections in the community. What are the critical factors affecting the delivery of iCCM to clients?

Respondents :- on case identification and provide correct treatment we have seen factors from previous performance review meeting with HEWs from those challenges some person is not come to health centers immediately after cases referred. because some are far distance from biyo HCs and the rest are residents adjacent to modjo town,like arifata jogola, tefi Abo, Mumu shoki and Shera ,they prefer to go modjo town. under this HCs we have seven rural kebele but only three kebeles used this HCs effectively.

We had observe knowledge gaps ,all HEWs was not equally understands even on this module ,we have seen module utilization problems on case identification ,referral and correct treatments .

Related to shortage of drug supplies for many times we communicate with responsible body particularly on health care financing is faced with danger problems on drug supplies because for many time drug stock out at EPSA and we bought from private pharmacy with high costs by health care financing budget ,but we provide for sick children free of cost.

to assess Growth monitor program of children we need digital weight scales but at present we use buckets "baldi" and community denied to place their child's on it

Health worker assigned at woreda level coordinating with HMIS focal person is not provide continuously technical supports as planned and all community has awareness on iCCM services but on effective utilization and mobilization there is some gaps, several people asks HEWs laboratory request for their infants but when HEWs told to them at health post no lab services they directly self transfers to HCs.

Interviewer:- How do you think the COVID-19 pandemic and/or COVID-19 response measures affect the uptake and/or delivery of iCCM services?

Respondents :- at beginning time communities are very frighten COVID-19 pandemic and health care workers also fear to visit house holds level. Not only this many people were not came to health centers because of COVID-19 pandemic . at COVID-19 periods our HEWs are didn't do their jobs as usual and the societies also didn't expose their children to HEWs because they suspect that HEWs might be infected by COVID-19 infection.

Generally our OPD per capital were very decreased during COVID-19 infection detected in our country, at that time,the community went to private clinic because they perceived that at private clinic good precaution and no over crowded . treatment as generally including iCCM services extremely affected because at health post HEWs fear to touch with infants to identify case and they orally refer to HCs.

In shera kebele I had seen with tangible evidences is that one infant ill at home and his umbilical cord were changed to red and when we asked her she responded that she took her baby to private clinic because fear of COVID-19 infection in HCs and Hospitals but no changes on infants ,the other is many people directly go to pharmacy or came to HCs and complain about infant disease ask only drugs without present sick infants.

To say really at COVID-19 period not only community daily activities but also Health worker extremely fear the COVID-19 infection ,every person daily watch and hear from media the number infected,the number death .depending on this the community were not prefer to came HCs they choose stay at home.

Gradually the fear of COVID-19 infection is decreased by integration of Health care workers from HCs with HEWs aware the community ,apply COVID-19 infection prevention methods at all level,provision of COVID-19 vaccination ,decrease of COVID-19 infection cases and deaths.

Interviewers :- Explain to us any changes you observed as the result of the implementation of the COVID-19 adaptive iCCM implementation strategies? – What impact has the strategies had? What are the particular features of the strategies that made a difference?

Respondents :- at present time we have seen big changes on iCCM services deliveries. every community actively bring their sick children to health workers and show freely babies problems and also health care workers without any fear of COVID-19 treat sick children.

Previous time while COVID_19 started we didn't stop any supports but weaken supports at present time we strength system supports at health post by supervising HEWs activities , at community level and house to house level with coordination with HEWs observe what is done,what is not done and the problems still not solved at community level relate to iCCM service deliveries.

our HEWs highly motivated to provide iCCM Services and daily requesting supply and commodity by HPMRR to health centers ,community participation was need improvements but when compare with COVID-19 period there is a big changes WDAs actively participated,Pregnant women conferences were conducted in all Health posts,Every participants express their ideas freely without fear of COVID-19 and took education provided by HEWs .

Interviewer :- What are the high-level benefits that are attributable to this support/IR?

Respondents :- by JSI/L10k supports we got big benefits firstly improve neglected activities, iCCM activities were stopped in previous time but at present it gate attention ,provide training for HEWs and focal persons then communities were perfectly utilize iCCM service delivery at health posts and increase their awareness.

Child death were decrease because HEWs early reach sick infants and provide treatments for them and actively referral for sever illness.

Interviewer :- Please explain to us the feasibility/practicably of this support/ IR for national scale-up?

Respondents :- yes it can possible scale up for other regions ,nothing is not impossible in our catchment area some health post performances improved from zero treatment to 30-40 child s at presents, if all necessary supply and essential commodity is fulfill for HEWs and sufficient training were given for HEWs it can simply expanded for other region.

Community participation ,community mobilization and community awareness were very essential to know iCCM services provide at health post by HEWs.

all Heath care workers including HEWs should enters to the communities and work with them actively.

If number of HEWs changed from two to three at one health post to provide services in all days, it is good because we have seen many challenges on it , Health post house is very narrow to provide all services it also need addition class construction for HEWs living room and service provision class.

Interviewer :- How are the activities/efforts embedded in the PHC and woreda routines/work streams? What implementation strategies are incorporated with the PHC and woreda annual work plan?.

Respondents :- we supported by plan ,as general iCCM/CBNC incorporate in to our checklist. Our annual work plan is comprehensive for all disease ,for example in our check list if the the title talks about ANC or Communicable Disease the body of check list ask iCCM/CBNC services because all of this has relation ships with iCCM services.

We would like to thanks JSI/L10k projects to start and apply iCCM services in our woredas at now we are on good progress and we didn't backed for ever because this organization works for us best bench marks on iCCM service deliveries.

Interviewer :- Do you have anything think is important to tell us that we have not asked you?

Respondents :- nothing I have added.

Interviewer: thank you for your time and valuable ideas

Respondent: thank you too.

I	Identification	
1	Questionnaire ID	T04
2	Area Identification	
3	Name of Woreda/Zone/Region	Lume wored
4	Name of facility	HCs
5	Name of moderator	
6	Name of a note taker	
7	Date of discussion	12/06/2022
8	Participant #	one
9	Audio File #	08
10	Start time:	6:32
11	End time:	8:13
12	Transcriber	

Interviewer :- Can you please describe the demand generation/SBCC activities you are doing on iCCM/ newborn health? What are the strategies used to raise awareness?

How is the engagement of communities/WDA networks in the implementation of integrated community-based case management (iCCM) of common childhood illnesses/possible serious bacterial infection (PSBI)?

Respondent :- relate with ICCM/CBNC service delivery we are implementing for all rural kebele and seven Health post under Biyo Health centers by using phone tablets.

On communities awareness creation and demand generation before this projects supports, the awareness of the community were very low on Child health. but after we use phone tablets based treatment for sick infants and the societies were recognized and start to know the services deliveries.

at present time our communities were aware on sick infant treatment is given at health post and they also bring their sick infant to health posts.

On demand generation and to reach our societies first Health extension worker are trained on ICCM/CBNC service, after training we start to aware Women development army network who are fundamental for ICCM/CBNC service delivery at health posts .next to this at school level students and teachers are aware on common childhood illnesses because they come from the communities and when they back to their home they can inform their families,their neighbour and all the people live around them.

The others is Kebele structures like kebele administration ,kebele manager, religion leader demand generation.

Interviewer :- what the Challenges faced on demand generation/SBCC activities

Respondent :- before JSI/L10K supports and at beginning of this activities there is many challenges at health post level ,the community awareness were very low .

Interviewer :- How do you describe the strength of the support you got from the PHC and the project as well as your efforts to address barriers to deliver iCCM/PSBI during COVID-19?

Respondent :-related to support ,we have been obtain different supports from Woreda Health office, JSI/L10k most of supports is to strengthen our activities.

related to training focal person and all Health Extension workers were took a training on ICCM/CBNC. After they took training all activities were supervised by checklist on registration and record utilization ,phone tablet utilization during sick child treatments .

after we obtain system supports from JSI/L10k we have seen big changes on ICCM/CBNC services for under five years children s, JSI/L10k were provide for us contentious supports on phone tablet utilization with practical demonstration to treat sick infants at community level,strong supportive supervision on child treatment at health posts, mentoring HEWs on ICCM/CBNC Activities ,active referral linkage and feedback s between Health Centers and Health post were also improved, Sick infant treatment at Community and health post level provided by Health extension workers.

***Intervie*wer :- Since you are focal person ,what Did you say on supports that need to bee improvements ?**

Respondent :- related to supports things to be need changes, we prepare ICCM/CBNC check list for our selves to support Health post by Health workers who took training on ICCM/CBNC for example from MCH and from OPDs but those who support kebele and Health post other than were not took training on ICCM/CBNC ,therefore if those who support up to kebele and Health post level take ICCM/CBNC training will change the entire Activities more.

Interviewer :- Did do you get the support system helpful (mentorship and on-site coaching methods, supervision, technical support, PHCU level PRCMM, etc.) to enhance HEWs' skills over the traditional off-site training and woreda level review meetings?

Respondent :- related to Coaching and mentorships has big role to enhance the skill for Health care workers because this is the skill transfers from highly experienced to HEWs to improve the services with high qualities. specially during mentorships mentee go to the place where the HEWs works to demonstrate how to treat sick infants for Health Extension Workers provides the services like health post and community level mentor . on site coaching also has big benefits to improve our quality of services, specifically they coaches on eCHIS and Booklet chart utilization at health posts. this support make HEWs had full confidences and prevents from treatment error. traditional off-site training has the Comprehensive iCCM/CBNC activities and every HEWs might got theoretical information after off-site training she came back to her work place and changed to actual works which has technical aspects that is essential for continuous on job coach and mentorships to fulfill practical gaps.since this

support is continuous and not at once time the HEWs knowledge and skill also improved. related to review meeting ,it could be provide at woreda level and Health center level to access what is work done, how many activities achieved but technical part is not evaluated ,review meeting simply to compare the number of performances not technical parts so Coaching and mentorshipes is better.

after HEWs got ICCM/CBNC Training and continuous support from JSI/L10k the communities acceptance for Health Extension worker and ICCM/CBNC service delivery were improved at health posts.

Interviewer :-How eCHIS implementation helps you with iCCM service delivery?

Respondent :- eCHIS has big benefits for iCCM Service delivery after we start to used eCHIS HEWs has been provide the services based on actual data on her hands, catchment population numbers on her hands, the numbers of children on her hands in her kebeles. since they had list of all childrens they could monitor who is took full services and who is not took full services in her kebele.

on EPI HEWs provide her activities by using eCHIS phone tablets, this phone tablet is very alarming for any activities at any times, for example to trace drop out based on different color she simple categorized for those dropped out from vaccination .for example if one child took penta one and didn't took penta two the phone tablet shows that this children is not took penta two based on this every activities has been controlled in modern manners.

Not only this specially on House hold possess like latrine ,solid waste and other inter to the phone tablets then time to time follow and update the house hold data . related to iCCM Service delivery after using eCHIS first it guide HEWs itself , it ask HEWs thing they observed on sick infants and displayed by disease classification in the phone tablets. for example if the disease is diarrhoea eCHISs classified as diarrhea disease then order the drugs to be provided with correct doses, for numbers days with number of time interval .the other is for those who couldn't treat at health post and need referral to health center theeCHISs supported by phone tablet simply order for referral. when cases referred from health post to health centers referral linkages were reach health center quickly and health workers in health center ready to accept referral before referred cases arrive there and feed back were given by phone tablets.

on eCHISs phone tablets crucial things did not pass if we couldn't fill the circle boxs for example if phone tablet asks respiratory systems and we didn't fill it we couldn't pass to next question therefore eCHISs guides us in correct ways and prevent as from treatment error.

related to appointment programming for those on follow ups set date to be came back for next services if the appointment date near to follow up days it shows alarm color and if the appointment date passed action card is given to trace the child up to home and check child health status then up date .

eCHISs data quality has need improvements because the number of infant treatment given at health post by using eCHIS phone tablets is not shown on the tablets .

Interviewer :- How was the introduction of the iCCM/CBNC module into eCHIS affected the iCCM/CBNC service delivery?

Respondent :- we have been provide many activities by eCHIS from those iCCM/CBNC service delivery is the one, during one Health extension workers record her catchment area population under five years children were directly merged under iCCM/CBNC module. Related to defaulter tracing it has color coding based on color if red color is shown the appointment date is passed ,if yellow color is show the appointment date is near future and needs give attention .this helps to prevent the children from missed appointments and children status were know time to time.

in a days our Health Extension workers were sink their mobile morning and afternoon ,during sink their mobile every updated data were observed by color coding then they see and take action for newly update data s.on re referral system iCCM/CBNC module clearly guide for precondition for referrals and make decision for referrals.to identify cases and treat sick infants by using iCCM/CBNC module typically reach to find correct case and treat with correct treatments

Interviewer :- Describe the main issues faced by the health system to identify and treat neonatal infections in the community. What are the critical factors affecting the delivery of iCCM to clients?

Respondent :-the factors that faced us is Lenovo and HUAWEI Tablet smart phone for eCHIS are very weak and some time stops to works and this makes service deliver to late .

Health extension Workers those took training already on jobs but HEWs those not took training has been challenges for iCCM service deliveries. As much as possible we have been supporting free drugs for sick baby treatments at Health post but at present time we couldn't spend to supports on free drugs.

since our kebele is far distance from health center and the population settled disperse , the transportation were very challenge us to supervise, mentor and coaching at Health post and community levels.

only focal person has took a training on iCCM/CBNC at health center level and the rest health workers who are supervise and assigned to health post were not took iCCM/CBNC training, this prevent us leading by knowledge. Some peoples interest to be served at health post were low this might cause to minimize sick child treatment at health posts.

During rain season the Health Extension Workers might not provide the services home to home visit far away from Health post and some Health Extension Workers are lives in Modjo Town because living home is not constructed at Health post

Interviewer :-How do you think the COVID-19 pandemic and/or COVID-19 response measures affect the uptake and/or delivery of iCCM services?

Respondent :-during COVID-19 period every body has been fright of infection and the community also didn’t come to health facilities .at that time no one want to go health facilities to treat for every disease. Even; if one child infected by pneumonia the community perceived that child were infected by COVID-19 and then stay at home and treat by traditional medicine,there is restriction on movements ,transportation cost double ,because home to home visit is a problem and the societies were not need to treat sick infants services at home level and also they didn’t want to go health posts to treat sick babies based on the vulnerability of the children's are highly increase

.the social daily activities were highly affect but those interruption were gradually minimized and normalized social movement restriction were lifted and all communities were aware on COVID-19 and apply COVID-19 Prevention method.

Interviewer :- Explain to us any changes you observed as the result of the implementation of the COVID-19 adaptive iCCM implementation strategies? – What impact has the strategies had? What are the particular features of the strategies that made a difference?

Respondent :- on implementation of the COVID-19 adaptation we have seen big changes on ICCM services. All communities and health workers apply COVID-19 Prevention methods,COVID-19 fright very low . based on this firstly all communities come to us for care-seeking ,social mobilization and awareness creation on ICCM Service were improved,at school level awareness creation were improved .

during COVID-19 all Health worker were very tension and bothers at present time all HEWs has been fully motivate and provide services routinely on iCCM s

Interviewer :- What are the high-level benefits that are attributable to this support/IR?

Respondent :- benefits we obtain from this projects is large particularly at Health Post level services provided supported by phone Tablet which helps Health Extension workers as guide for sick infant treatments to improve their self confidence and minimize treatment error.

Defaulter were simply traced and back to services based on color coding ,referral linkage between health post and health center is improved and preparedness of health workers at health center increased ,active feedback with health post is good, communities were very happy to use eCHIS services provided, child death were minimized because sick babies treated in standard treatment in short period of times and active referral linkage between HP and HCs

Interviewer :- Please explain to us the feasibility/practicably of this support/ IR for national scale-up?

Respondent :- this support can expand to other region because this project supports has been bring for us to decrease Mother and child deaths this is strategies of the country had planed ,Train Health extension workers on eCHIS, provide phone tablet for all Health extension workers and provide free drugs to treat sick infants.

every body who works in every level need to committed to do iCCM services.

But my fear to prevent from scale-ups is phone tablet and drug supply ,training for scale-up all of these need huge budget ,geographical situation may has its own problem .

Interviewer :- How are the activities/efforts embedded in the PHC and woreda routines/work streams?What implementation strategies are incorporated with the PHC and woreda annual work plan?

Interviewer :- at biyo health center we have annual work plan on ICCM/CBNC as one initiatives ,all Health extension workers provide services for Sick infant treatment by eCHIS at health post daily and reports daily ,at health center level MCH and Under five OPD health workers has been daily follow eCHIS activities ,iCCM/CBNC incorporated to annual work plan by age dis-aggregation

Interviewer :- Do you have anything think is important to tell us that we have not asked you?

Interviewer :- I have already discussed in interview

Interviewer :- thank you very much for your time and response to my question

Respondents :- thank you too.

I	Identification	
1	Questionnaire ID	T06
2	Area Identification	
3	Name of Woreda/Zone/Region	Lume wored/East Shoa/Oromia
4	Name of facility	HCs
5	Name of moderator	
6	Name of a note taker	
7	Date of discussion	12/06/2022
8	Participant #	one
9	Audio File #	07
10	Start time:	4:32
11	End time:	5:08
12	Transcriber	

Interviewer: - Can you please describe the demand generation/SBCC activities you are doing on ICCM/ newborn health? What are the strategies used to raise awareness?

How is the engagement of communities/WDA networks in the implementation of integrated community-based case management (ICCM) of common childhood illnesses/possible serious bacterial infection (PSBI)?

Respondent :- During the basic ICCM training is given I was on rest , but after i trained recently ,the community does not bring their children under two to the health post because there is no enough drugs and there were no enough awareness ,but after we trained we give SBCC on how we enable ,trained that we can treat the sick baby and help them about how the mothers breast feed for their babies.

The involvements of WDA, during pregnant women conference are the critical way we used health education through the WDA leaders and they pass the information to 1-5 networks to bring the children to health post for child vaccination after 45 days of birth, how they exclusively breast feed, PNC follow up .The 1-5 network members then may bring them or call us.

Interviewer: What are some of the biggest challenges about SBCC activities for newborn care?

Respondent;-The challenges may arise when there were no drugs, syrup, the prefer to go to other health facility especially those who has low information, the other problem were the distance, infra structure since the kebele is wide and far apart and being the only one Health extension worker are the main challenges that limit the behavioral change out to bring for community because of being busy.

Interviewer: - How do you describe the strength of the support you got from the PHC and the project as well as your efforts to address barriers to deliver ICCM/PSBI during COVID-19?

Respondent ;-concerning the support given ,especially on the use of electronics phone tablets , it motivates my work during lonely in rural areas and since all data is captured and with me all the time ,the phone tab remind me with red signal for a child needs treatment or appointment reached and for example I remember that pregnant

whose appointment reached alarmed me with red signal and I tried lot and find the her to manage the case accordingly .

The other result of the program is, it helps the children to take full dose of vaccination, treatment of sick baby children , having the data on my hand is better than everything so that it couldn't be forgotten stolen .

The support given especially the awareness I got from the project from the project is also very helpful during the COVID 19 pandemic, because during that the everybody was in panic, so that the use of mask and other protecting way of COVID 19 helps me to ensure the service aimed as per the plan, but not enough behavioral change is brought on community about how to prevent and vaccinated for pandemic COVID 1, because they thought the as there is no a disease even the educated one gov't employee also deny to take the vaccination .

Interviewer: - Did do you get the support system helpful (mentorship and on-site coaching methods, supervision, technical support, PHCU level PRCMM, etc.) to enhance your skills over the traditional off-site training and woreda level review meetings?

Respondent:-, The technical support and mentorship enhances our skill , we didn't know how to fill the card , register before , especially the support given at job place the training they gave us there .

I didn't believe that I could treat a child less than two year at health post, but now am very confident to treat children with different cases.

The Mentorship, onsite coaching ,supervision and review meeting are the way of improving the performances of work , one helps the other and onsite coaching and mentorship may also equip well than others as of review meeting are of comparing one with other site performances and from planned one .

Lastly, I think these methods are better if done turn by turn, that is if review meeting held with other kebeles after on site coaching and mentorship is given primarily.

Interviewer: - How eCHIS implementation helps you with ICCM service delivery? What do you think can be changed or improved on Data quality on eCHIS implementation?

Respondent;- It has many advantages for example ,baseline data ,pregnant women , birth given in kebele , household data are on my hands , so that the tab can inform , pregnant women gave birth given from health center through feedback.

In Case identification and managing ,the tab itself categories the cases and alarm me the date of appointment and can show me if I miss diagnose and treat with recommended dosage .

Data quality can be checked by tab because It prevents registering other kebele's children**,** and gives signals as green yellow and red (appointment passed) for follow up to be reached , I didn't wait for red signal and will make decision being on yellow signal and I can assess the name of child treated and cured by Childs name.

I think the eCHIS is a good approach and helpful that doesn't need much improvement.

Interviewer: - How was the introduction of the ICCM/CBNC module into eCHIS affected the ICCM/CBNC service delivery?

Respondent;- Nowadays the services are given for under 2 children which was un learned before , pregnant women may also come to health post bow than before .

The tablet guides me to follow the expected procedure than manually assessed by myself because I may forget the important points.

If I refer the case to health center they follow the client from the far and may give us the feedback after the patient seen /diagnosed.

That defaulter can be searched, by using tabs information, we may check their satisfaction by different methods in the community, and they may come back if satisfied and may call us for anything they feel about

Being busy by a lot of services we delivered, it is difficult to address the ICCM activity with this lack of man power, beyond this nothing is needed to deliver the program.

Interviewer: - Describe the main issues faced by the health system to identify and treat neonatal infections in the community. What are the critical factors affecting the delivery of ICCM to clients?

Respondent:- We as HEWs didn't have skill to treat the children before this program is started at this kebele , but now I had full skill to treat cases ought to be managed at health post , because skill and logistics like of drugs and supportive supervision is exist.

The challenges may differ one from the other, some may think we as HEW can't treat the children because there may not exist enough drugs they prefer than syrups here at health post.

Interviewer:-How do you think the COVID-19 pandemic and/or COVID-19 response measures affect the uptake and/or delivery of ICCM services?

Respondent :- The occurrence of Pandemic COVID 19 , limits the services needs to be delivered to the community , peoples limits their movement to health post , and didn't tell if cough happened to a children , because they are ashamed of telling disease history for example it is difficult to differentiate the suspected TB patient s by cough history as they afraid of COVID 19 symptoms .

It also limits us to move for creating SBCC on ICCM and COVID 19 prevention methods from home to home during the epidemics. But I tried to implement COVID 19 prevention methods as of Vaccination against a disease, wearing face masks, physical distancing are the measures we used to deliver the services.

Interviewer: - ***Explain to us any changes you observed as the result of the implementation of the COVID-19 adaptive ICCM implementation strategies? – What impact has the strategies had? What are the particular features of the strategies that made a difference?***

Respondent :- I used to deliver the information on Faith based organization (iddir)about how to use protection methods and send any sick one to health post especially children with different cases even during panic time . The leaders including women development army are used to create awareness on how to be adaptive to the measures of COVID 19 and giving care for any one being sick especially pregnant women and children .

Not only the COVID 19 case that hinders the services of ICCM here in our health post , but the location of health post is also another factor because many of the catchment population far away from us and is nearby for other kebeles of under Biyo health center and which is assessable for cart transportation than coming here on foot .

Interviewer:-What are the high-level benefits that are attributable to this support/IR?

Respondent :-The big results of this program after started here is that , reduces the probability death of children at home because , our community's health seeking behaviour is low , so that the sick child may be stay at home and after being sever with disease will taken to health facility .But now they have enough information where to follow their pregnancy , give birth and follow PNC visit and the child immunization .

In addition to these treating children like this makes us very confident, we didn't think ourselves as a health professional before, so that we are happy now and treat the children we never touch them before.

The use of technology also improves our work, client satisfaction is also increased it reminds us appointment days, data quality and others are among good things of using the tabs.

Interviewer: - Please explain to us the feasibility/practicably of this support/ IR for national scale-up?

Respondent ; - Yes it is possible if it is scaled up as a national level , the death of children may reduced , and if the thought about HEW can't give curative services is given awareness for the community it is a better way of reaching a child from death .

I think if the accessibility of tablet for all HEW , nothing limits them from delivering the services .

Interviewer:-How are the activities/efforts embedded in the PHC and woreda routines/work streams? What implementation strategies are incorporated with the PHC and woreda annual work plan?

Respondents; - ICCM program is planned with annual work plan, we include the number of pregnant women and follow the the live birth, using the conversion factors to calculate women in child bearing age. Physically registering the pregnant women also will help us to decide the number of clients expected to deliver the services The baseline data will come from woreda health office with supplements.

Interviewer :- Do you have anything think is important to tell us that we have not asked you?

Respondent:- The limitations include ina accessibility of HP , cleanliness , shortage of drugs like gentamycine, syrups ,and medical equipments of like weight scale .

The community needs more awareness because they sometimes prefer taking the delivered child to health center than bringing to us for simple cases.

Interviewer: - Thank you very much for your time and response to my question

Respondents:-Thank you too!.

I	Identification	
1	Questionnaire ID	T07
2	Area Identification	
3	Name of Woreda/Zone/Region	Lume wored/East Shoa/Oromia Region
4	Name of facility	Health Posts
5	Name of moderator	
6	Name of a note taker	
7	Date of discussion	15/06/2022
8	Participant #	one
9	Audio File #	017
10	Start time:	5:04
11	End time:	6:08
12	Transcriber	

Interviewer :- Can you please describe the demand generation/SBCC activities you are doing on iCCM/ newborn health? What are the strategies used to raise awareness?

How is the engagement of communities/WDA networks in the implementation of integrated community-based case management (iCCM) of common childhood illnesses/possible serious bacterial infection (PSBI)?

Respondent:.concerning ICCM service delivery the main job is on under 5 the work is undergoing as per training given.

we aware the people during Pregnant Conferences and vaccination was undergone for pregnant women and after training is given for us by JSI/L10K the iCCM service had been given for under two year.

We promote our health posts on the treatment of under two children were given on pneumonia,diarrhea ,local bacterial infections .

In addition to this during CHD drugs like deworming and Vit A is being given for children's two times in a year in especial way we aware the community than before.

The ICCM activity is mainly focuses with awareness for Women in their networks as of WDA that are in good progress 7 ''gares''(groups) out of 9 in my kebele , even though it is on start ,frequent awareness is being given for group /''gare'' leaders in especial occasion like of Health conference being with health center staff.

In case of religion leaders we aware them on different meeting than regularly using them than of WDA .

At ‘’Gare’level a deep awareness had given for Gare leaders and WDA leaders on the existence of child Treatment and different drugs concerning ICCM, because they believe that there were no drugs at health post and they didn’t come to the health post and take the services.

Interviewer: What are some of the biggest challenges about SBCC activities for newborn care?

Respondent :- There were no many challenges at the moment because challenges were exist before , when there was no drugs at post and they assumed that HEWs will refer me to health center.

Now we had drugs even though it is not enough , we request Health center and they respond us as per their drug balance, they give us like of Gentamyccine for under two children and other for preventing infection during birth and those who took the services are being advocating about ICCM Treatment at HP.

Interviewer :- How do you describe the strength of the support you got from the PHC and the project as well as your efforts to address barriers to deliver iCCM/PSBI during COVID-19?

Respondent :- JSI/L10k Provide for us basic and constructive training on iCCM services.

They continuously provide for us on site coaching on how to I registered childrens history ,mentor me on my mistake on child treatments and they correct me by practical demonstration.

JSI/L10k might be phase out after some year so our HCs and Woreda Health office should be help us like this organization on supportive supervision at least monthly ,mentoring on child treatment .

Interviewer :- Did do you get the support system helpful (mentorship and on-site coaching methods, supervision, technical support, PHCU level PRCMM, etc.) to enhance your skills over the traditional off-site training and woreda level review meetings?

Respondent :- yes we got so much Supports like on site coaching ,refreshment training , supportive supervision ,mentorships from those support I have been aware on my works ,correct my mistakes related to how to treat infants.

On iCCM Activities I have got many benefits from supports because I have understood what I forgotten on child treatments , they support us by practical demonstration this help us to improve our knowledge and skill.

They provide supporting material like chart booklet that updated on child treatments and simple treat the children , for example if we took pneumonia i might be forget many things but chart booklet guide us to treat the childs. this is big things for me because child treatment at health post is on good progression .

If we compare present child treatment with previous child treatment at health post there is big difference in numbers.

At previous time we had interest to treat sick infant but we only provide referral to health center because we had a knowledge gaps on sick child treatments, but at present time we treat sick childrent perfectly.

We have seen difference between off-site training and on-site technical supports, off-site training is provided as general and took theoretical , on-site technical supports is practically demonstrated and changed to actual supports .

after we got support from JSI/L10K like ICCM Training ,Supportive supervision and on job mentorships my self confidence and competences improved to treat sick infant and the people were also give us good respect on our recent knowledge and practices.

Interviewer : -How eCHIS implementation helps you with iCCM service delivery?

Respondent :- eCHIS is good,it is phone tablet based work , Total population family registration had been done by it , treating children using eCHIS phone tablet based treatment is a very good way because it remembers me the step to follow and standardized.

treatment had been giving for all children using the same procedure and protocol when using phone tablet if don't using the phone tablet i asked the client information already i know/remembered ,thus why all cases will be addressed through our discussion ,

But we couldn't asses the number of cases addressed through the tablet ,but only can be found on registration book .

eCHIS helps in iCCM data quality because we has True data what registered on , it helps for appointment schedule by showing color codes, but unable to get the report back is still a problem.

Interviewer :- How was the introduction of the iCCM/CBNC module into eCHIS affected the iCCM/CBNC service delivery?

Respondent :- Even though The existence of this module in hard copy ,soft copy is very easy to read ,easily remind and asses in daily bases of my activity ,because of lack of time and its un accessibility it is difficulty to rehears the point in need .

The other speciality *introduction of the iCCM/CBNC module into eCHIS* remind for appointment program ,it has detail explanation color code like blue,yellow and red to trace default,Since we have their phone number we call them and they also might be call us for any help they want . the other thing is when we move from home to home we used to refer for any case we observe ,it also guide as to treat with standardized drugs for sick children

It is also good to get referral feed back through eCHIS because we got the feedback also through our phone tab not waiting for a piece of paper the whole a period of time.

Incorporating the iCCM/CBNC module into eCHIS phone tablets helps us easily to refer for clinically to treat children if we couldn't treat how to refer,for follow up children on treatment or on appointments and improve community satisfaction because we easily treat infants.

Interviewer :- Describe the main issues faced by the health system to identify and treat neonatal infections in the community. What are the critical factors affecting the delivery of iCCM to clients?

Respondent :- Absence of drugs supply in a place where demands of client is increasing is difficult because for many time out of stock here and at health center also.

Due to a COVID-19 infection there were a service hindered due to emergency state regulation to stay at home,difficulty with transportation and peoples also thought as if they may catch the COVID 19 infection.

Interviewer :- How do you think the COVID-19 pandemic and/or COVID-19 response measures affect the uptake and/or delivery of iCCM services?

Respondent :- at COVID-19 time it is difficult period because not only community we also highly fear the COVID-19 infection ,at that time we are very happy if the community is not came to us if they need some help we expected to help them at there.

Our daily activities were very weaken because at that time all people restric their dail movement and declared stay at home regulation ,no transportation to move from place to place.

the community were not brought their sick infant to health post because they perceived that we infected by COVID-19 at health posts and also we couldn't go home to home vise to identify sick infants at community.

To improve this problem we used to go home to home to address the children with problem using COVID 19 protocol (mask ,sanitizer) ,Gare(group leaders and Religius leaders involvement in the program helps us more.

Interviewer :- Explain to us any changes you observed as the result of the implementation of the COVID-19 adaptive iCCM implementation strategies? – What impact has the strategies had? What are the particular features of the strategies that made a difference?

Respondent :- related to COVID-19 we provide Vaccination for COVID 19 for the community , since the side effects with vaccine type Sinopharm is difficult , but after awareness is given almos all people had vaccinated.

Being we used COVID-19 Prevention method is helps us to improve the iCCM /CBNC service delivery and sick infant treatment at health post.

The main improvements of the ICCM services and child treatment at health post is increase mothers awareness ,they even call us when in need to do with ill child ,and there were also a time we went to home and check for the complain with PHCU Directer.

The patient satisfaction is better than before , not only them but also we are happy now using the eCHIS

Interviewer :-What are the high-level benefits that are attributable to this support/IR?

Respondent :- I took the big benefit is that all mother has been gate awareness on iCCM service delivery and they actively bring their sick infant on time to health post.

Decrease child deaths and child morbidity from possible bacterial infection .

Every child treatment , case identification and referral were depend on technology based and minimize hard copy ,improve service quality,increase our knowledge,skill and competencies

Interviewer :- Please explain to us the feasibility/practicably of this support/ IR for national scale-up?

Respondent :- Yes it can by using the phone tablet helps us Memories activity to be addressed and the community also will have confidence on HEW considering as we are modernized and educated more .

Having voluntarism and training is a very mandatory to use the phone tab and helping the community at large.

All HEWS and Focal person have to take a training on iCCM/CBNC services,all material should be available for this services.

If there is transportation problem ,no road to connect community with health post,residence disperse are the problems that prevent to scale up.

Interviewer :-How are the activities/efforts embedded in the PHC and woreda routines/work streams?What implementation strategies are incorporated with the PHC and woreda annual work plan?

Respondent :- our annual work plan is planed once coordination with health centers and after plan we start to do our activity.

Our annual work plan is planned at HCs and the break down to seven kebele.at present time we plan annual work plan based on what we have at hands in our eCHIS phone tablets .

Interviewer :- Do you have anything think is important to tell us that we have not asked you?

Respondent :-nothing I have.

Interviewer: Thank you for your time and valuable ideas

Respondent: thank you too.

I	Identification	
1	Questionnaire ID	T08
2	Area Identification	
3	Name of Woreda/Zone/Region	Lume wored/East Shoa/Oromia Region
4	Name of facility	HPs
5	Name of moderator	
6	Name of a note taker	
7	Date of discussion	17/06/2022
8	Participant #	one
9	Audio File #	020
10	Start time:	8:38
11	End time:	9:37
12	Transcriber	

Interviewer :- Can you please describe the demand generation/SBCC activities you are doing on iCCM/ newborn health? What are the strategies used to raise awareness?

How is the engagement of communities/WDA networks in the implementation of integrated community-based case management (iCCM) of common childhood illnesses/possible serious bacterial infection (PSBI)?

Respondent :-Yes ,First we made awareness to the community about the treatment we give for children under five and under 2 years using community facilitators , Garee(group),one to five network, Idir, WDA, and now they are happy and even expect from us if we provide delivery services for pregnant women is given here.

We focused on the social networks as of Idir (FBOs),pregnant women conference held every month at health post and aware about importance of early ANC follow up ,Delivery, PNC ,child diarrhea ,pneumonia, and other bacterial infections of children to create awareness than school based because our school is low grades up to 4 grades ,so that it is difficult to bring behaviour change and passing information by them .

We show to the people on the phone tablet , it helps us to justify disease category (treatable stage) (the yellow signal on tab) at Health post , referring to other health facility if red signal is seen on tab.the same procedure is done for mothers and may referred if the diseases seen is beyond our capacity and for other Lab. Investigation this make them to aware on treatment give by technology methods .

Interviewer: What are the challenges you face while raising SBCC awareness for newborn care?

Respondent;- challenges we faced is the residences of the community were distance from health post and difficult in infrastructure since the population density are disperse populated, we go from one administrative zone to other so that it takes us more time to address them

Interviewer :- How do you describe the strength of the support you got from the PHC and the project as well as your efforts to address barriers to deliver iCCM/PSBI during COVID-19?

Respondent ;- it gives us training , phone Tablet and other materials as of Chart booklet. we get detail awareness on iCCM service deliveries from this project .

The technology of using phone tab is better than using hard copy , it is easy to asses using username from everywhere and can be downloaded from dashboard even if unknowingly deleted or stolen .

This organization make us actively work with women themselves and they know services given here and even they are informed about schedule for child immunization .

The main advantage of the tab is, it shows you how to use, where to proceed ,not time consuming , it used correct and true data and can be assessed from everywhere than being only at heath post as of registration .

Interviewer :- Did do you get the support system helpful (mentorship and on-site coaching methods, supervision, technical support, PHCU level PRCMM, etc.) to enhance HEWs' skills over the traditional off-site training and woreda level review meetings?

Respondent ;- Woreda health staff had visited us on mentorship and they help us how can i improve my work , but it is very difficult to address all point because is bulky may take two days .

Now i have full confidence in front of our community because of the skill we get from the project in different way , even-though child treatment is difficult because a child can't tell you the sign and symptoms as you said . but now since i like the department ICCM a care of child ,I am a happy and passing an excellent moment with my community .

Interviewer :- How eCHIS implementation helps you with iCCM service delivery?

Respondents :- eCHIS helps us in registering all children's ,including their cases and shows cases with possible solution even to refer it to higher facility , it helps us by giving us the way we decide which drug is best ,if we include or fill the patient complain heard from the mother or a child ,chart booklet also shows drug dose.

using eCHIS phone tablet is very helpful in treating children based eCHIS phone tablet treatment is a very good way ,because it remembers me the step to follow and standardized treatment had been giving for all children using the same procedure and protocol when using phone tablet.

But the problem with the tablet is we can't asses the number of children addressed at the end of the month.

Interviewer :- How was the introduction of the iCCM/CBNC module into eCHIS affected the iCCM/CBNC service delivery?

Respondent ;- iCCM/CBNC module has good explanation for how to treat,order drugs,how to refer to HCs and how to follow during treating a child, it found in my phone tablets also ,if we follow the the procedure it stated before, it is helpful ,we didn't miss any thing and it shows us signals green ,yellow and Red to decide to refer and appointing the child with diarrhea and pneumonia when they come back and the colors shown us a signals has an apportionment days

it summarizes the recommended drugs with appropriate dosage for the list of disease symptoms we already filled from patient history.

Interviewer :- Describe the main issues faced by the health system to identify and treat neonatal infections in the community. What are the critical factors affecting the delivery of iCCM to clients?

Respondent;- at previous time we faced many problem especially shortage drugs but at present time all of the problem were solved.

We haven't digital weighting scale to measure child weighting,

Community awareness were not equal ,so some people were no accept our treatments.

At starting time the COVID-19 were highly affect us to identify and to treat sick infant in the community.

Interviewer :- How do you think the COVID-19 pandemic and/or COVID-19 response measures affect the uptake and/or delivery of iCCM services?

Respondent ;- during COVID -19 pandemic, there were a panic time , all health professional with the community also very afraid of the disease based on this they couldn't brought their sick child s to health posts and the HEWs also fear to treat sick infants.

Our community daily activities were very interrupted and they stay at home even when they sick they treated by traditional medicines.

You know community is resistive to use Face Mask , staying at home ,movement restriction were also existed .

But we tried to solve the problem by creating awareness on COVID 19 transmission way , by providing Water for frequent hand washing when the came here health post and this also improved knowledge s.

from the experiences they get how to prevent the diseases from the staying long time with the epidemic COVID 19 and the vaccination given time to time .

Interviewer :- Explain to us any changes you observed as the result of the implementation of the COVID-19 adaptive iCCM implementation strategies? – What impact has the strategies had? What are the particular features of the strategies that made a difference?

Respondent ;- we as a HEWs we didn't stay at home even during epidemic ,we used to educate the community at health post on sick child treatments.

without any fearing the communities come to health post and gate any services, like child immunization ,treatments for sick infants.

all people aware on COVID-19 infection, prevention and control methods ,

after COVID-19 infection adapted we got improved supports from Woreda and health centers on Supportive supervision, coaching and mentorships on iCCM services deliveries as usual.

The community awareness on iCCM services delivery were increased and routine as to before COVID-19 Pandemic periods .

At present time the community is awered about the COVID 19 and their participation on iCCM Activities were is increased .

Interviewer :-What are the high-level benefits that are attributable to this support/IR?

Respondent ;- Healthy fullness of our children is one of the benefits we gained, reducing death of children due to pneumonia , reducing transportation costs needed to treat the child to far away to hospital .

Quality of work is also better than before after the program started here and we gained also confidence of treating the complicated cases .

Interviewer :- Please explain to us the feasibility/practicably of this support/ IR for national scale-up?

Respondent ;- Yes, exactly , we also didn't expect the program will come here but as chance we get this program and we get great job with , and gives services in treating the children and we gate a confidence now and if this given for other zones and region good because there are the same HEW there also they could be a confidence for their community in treating children under 5 and under 2 year .

Nothing can back *feasibility* ,because if training is given for all HEWS and for focal Health Workers and if different stakeholders help them it is easy way to give services even to different region .

Interviewer :-How are the activities/efforts embedded in the PHC and woreda routines/work streams?What implementation strategies are incorporated with the PHC and woreda annual work plan?

Respondent;- in our annul work plan ,we already have baseline survey and we know how much under five and under two year children are there under our health post . So that it is clear to how much children is ought to be addressed by HEW in our HP .

Interviewer :- Do you have anything think is important to tell us that we have not asked you?

Respondent :- Nothing to add.

Interviewer: thank you for your time and valuable ideas

Respondent: thank you too.

I	Identification	
1	Questionnaire ID	T09
2	Area Identification	
3	Name of Woreda/Zone/Region	Lume wored/East Shoa Zone/Oromia region
4	Name of facility	Health Post
5	Name of moderator	
6	Name of a note taker	
7	Date of discussion	13/06/2022
8	Participant #	one
9	Audio File #	010
10	Start time:	2:45
11	End time:	3:50
12	Transcriber	

Interviewer :- Can you please describe the demand generation/SBCC activities you are doing on iCCM/ newborn health? What are the strategies used to raise awareness?

How is the engagement of communities/WDA networks in the implementation of integrated community-based case management (iCCM) of common childhood illnesses/possible serious bacterial infection (PSBI)?

Respondent :- even if ,we trained on Child health before ,we had a gaps on treat sick children ,to be served at health post if the community has sick childs we aware the community on iCCM services, we aware kebele structures; like ,kebele administrator ,kebele manager ,zone and one to five leaders on iCCM services provided at health posts.

We used pregnant women conference ,on this occasion lactating women also participated and we aware not only about birth preparedness but also we aware iCCM services deliveries.

We aware the society on different meeting ,WDAs leaders because they lives in the community more than us and transfer awareness for their networks.

Interviewer :- How do you describe the strength of the support you got from the PHC and the project as well as your efforts to address barriers to deliver iCCM/PSBI during COVID-19?

Respondent :- on infant treatments JSI/L10k provides for us on refreshment training, coaching us on how to treat sick infants,provide for us continuous supportive supervision, from HCs also iCCM services were integrated to routine activities .

The support we have got from top level is very essential, because if the JSI/L10k wouldn't supports us we couldn't see our gaps and this support make us to see ourselves gaps,they also correct us on our gaps and appreciate us on good things .

After we got different supports our sense of responsibilities on our works is increase make us to examine community problems while home to home visits and to focus on iCCM services in the societies.

regarding to different support I would ,like to thanks to JSI/L10K to give attention for this activities which is not works in other woreda ,provide different training to strengthening iCCM services .

Interviewer :- Did do you get the support system helpful (mentorship and on-site coaching methods, supervision, technical support, PHCU level PRCMM, etc.) to enhance your skills over the traditional off-site training and woreda level review meetings?

Respondent :-we have got much Supports like mentorships on this we see many things we forgotten ,remind us a things took in training that not work on it,they demonstrate us with evidences on how to treat sick infants by using phone tablets, how to identifies cases based on sign and symptoms, how to refer a cases to HCs, how to prescribe the drugs for sick infants ,how to archive our file and different documents this supporting improve our knowledge and skill.

For me mentorship and on-site coaching better than off-site training because during mentorship we sit side by side and detail discussion on some case,practically working with mentor how to perform our activity in health posts ,this improve our knowledges.

After we got support from top level we have seen many changes especially on awareness creation for the community on child treatments at health posts, we simply by using phone tablets we treat sick childs based history taken from mothers, it guide us what to given for sick childs, guide us what type of counseling is needed for so no challenges to treat children at health posts.

after we got support from JSI/L10K like technical supports the community were also give us good respect on our recent knowledge and practices ,their attitudes were also changed, if some problem is occur they consult with us and we provide the services for them,at previous time they didn't consult with us and they go to other health facilities without communicating with us.

At previous time our child treatments were very low ,in months we treat one childs at health posts,but after we got support system we got big changes, we treat up to five children .

Interviewer :- How eCHIS implementation helps you with iCCM service delivery?

Respondent :- eCHIS implementation and iCCM services has relation ships, we collect evidence of children residence in our catchments areas , eCHIS help on iCCM service quality because it has true children data in our kebele ,this is correlate with my plan and achievements.

using eCHIS phone tablet it asks me what I observed on sick childre and then enter to Phone tablets then if I said yes it guide me, if I made wrong it correct me , if I am on correct way it identify the case and place for me the cases

with correct treatments ,it appoints for cases who need appointment schedules ,if appointment date passed it remind me by red color showing and after that I provide the services for that childs.

eCHIS helps to treat sick child with correct drugs dose **,**frequencies to take a days and eCHIS is very good system for every person because make our burden of activities to easy with standardized treatments..

Interviewer :- How was the introduction of the iCCM/CBNC module into eCHIS affected the iCCM/CBNC service delivery?

Respondent :- introduction of the iCCM/CBNC module into eCHIS makes us to simply refer from Phone tablets during we move from house to house and avoid carrying hard copy .

For infants less than 45 days we register under delivered mother but after 45 days we register for child separately and follow separately for child ,during home to home visits for postnatal care we also check the status of mother and child by referring *iCCM/CBNC module in phone tablets.*

iCCM/CBNC module help us to identify the cases simply ,after we investigate child problem without any bother we simple refer the module and guide us simple to identify the cases.

In the module there is disease severity classification depend on our capacities and it indicate for those could be treated at health post and for those who couldn't be treated at health post ,then explain in the module how to refer to HCs based on their disease severity.

After we provide essential treatment for sick children and provide necessary drugs it display that do you want to follow this children if yes it displays appointment date and monitor children accordingly,not only appointment we also refer for tracing lost to follow up case from treatments.

the activities done by using iCCM/CBNC module improve our knowledge and skills and we also treat children perfectly and they were cured the communities also very happy on our treatments of their infants by supported on eCHIS phone tablets.

Interviewer :- Describe the main issues faced by the health system to identify and treat neonatal infections in the community. What are the critical factors affecting the delivery of iCCM to clients?

Respondent :- even if our community has awareness on sick child treatment in Health post ,some people were not took their sick children to health post and to health centers , they took to private clinic.

We have several treatment commodity some what but it needs additional other material to provide standardized treatment for children.

Interviewer :- How do you think the COVID-19 pandemic and/or COVID-19 response measures affect the uptake and/or delivery of iCCM services?

Respondent :- at starting period COVID-19 infection has been highly affect iCCM service deliveries , at that time the community were not only didn't brought their sick children to health post but also they didn't volunteers to touch their children even when we visit at their homes, Even We also not want to treat sick children.

At beginning COVID-19 infection has been affect our daily activities,we couldn't visit home to home activities .

During COVID-19 infection started in our country every body has been very fear,their work activities were highly affected,even on health workers big stigma and discrimination related to they are highly suspected to be infected by infection for this reason iCCM Services were highly interrupted .

After period of time all we explained problem has been changed up to said no COVID-19 infection in community.

Interviewer :- Explain to us any changes you observed as the result of the implementation of the COVID-19 adaptive iCCM implementation strategies? – What impact has the strategies had? What are the particular features of the strategies that made a difference?

Respondent :- at present time there is big changes , community were not fear COVID-19 infection , without any fearing the communities come to health post and gate any services like child immunization ,treat sick childs.

At present time no any thing bother us and the community ,thus every sick child's who could be treated at our level is gate any treatments services and who couldn't be treated our level is referred to health centers as usual.

During COVID-19 time our interest to support the community were low and community also didn't came to us ,even if when they came to us we fear them to serve them reversely they also fear us ,but at present time no such things from community and from us even our motivation is increased.

The community participation and social awareness on iCCM services delivery were increased and routine service were provided like before COVID-19 Pandemic occurrence .

Interviewer :-What are the high-level benefits that are attributable to this support/IR?

Respondent :- we get many benefits from these ,eCHIS Phone tablets to identify the case , treat sick children and monitor in simple ways, improved data quality, decrease child death because they gate treatment early and gate standardized treatment for children .

Interviewer :- Please explain to us the feasibility/practicably of this support/ IR for national scale-up?

Respondent :- yes ,if this support scale up for other region it is the best ,since HEWs work in all region this support also expanded by supporting like training for HEWswork .

Interviewer :-How are the activities/efforts embedded in the PHC and woreda routines/work streams?What implementation strategies are incorporated with the PHC and woreda annual work plan?

Respondent :- we prepare ICCM annual work plan incorporating with treatment under two month children,treatment under five month ,treatment child darrhea and with sepsis treatments.

Interviewer :- Do you have anything think is important to tell us that we have not asked you?

Respondent :- nothing to add.

Interviewer :- thank you very much for your time and response to my question

Respondents :- thank you too.

I	Identification	
1	Questionnaire ID	T10
2	Area Identification	
3	Name of Woreda/Zone/Region	Lume wored/East Shoa Zone/Oromia region
4	Name of facility	Health Post
5	Name of moderator	
6	Name of a note taker	
7	Date of discussion	12/06/2022
8	Participant #	one
9	Audio File #	012
10	Start time:	3:20
11	End time:	4:30
12	Transcriber	

Interviewer : - Can you please describe the demand generation/SBCC activities you are doing on iCCM/ newborn health? What are the strategies used to raise awareness?

How is the engagement of communities/WDA networks in the implementation of integrated community-based case management (iCCM) of common childhood illnesses/possible serious bacterial infection (PSBI)?

Respondent :- to generate community awareness we used every chances we got; like, Idir " afosha" places ,when we move house to house, additionally during mother came to health posts for vaccination we aware the community on children from zero to five year can treated at health post free of costs ,during post natal care we aware the mothers.

we participate religion leader , team leaders of women development army and discussion on iCCM service, if sick children presents surrounding in their societies they should took to health post and make as hot agenda in the community.

WDA has their own network like leader,secretary and members, team leader and secretary were talk about iCCM services time to time with us then after they got enough knowledge they meet with their networks and aware them by this method if one child treated in our health post and cured they took as exemplary to teach each others

.

Many people ask us that why you couldn't give us injectable drugs rather than tablets.

Interviewer : -How do you describe the strength of the support you got from the PHC and the project as well as your efforts to address barriers to deliver iCCM/PSBI during COVID-19?

Respondent :- strength JSI/L10k provide for us is different training,strengthen us on our jobs ,conducts constructive supportive supervise for us, reminding us on iCCM services .

Make us to give attention on iCCM services and actively communicate with HCs and woreda health offices.

Interviewer:- Did do you get the support system helpful (mentorship and on-site coaching methods, supervision, technical support, PHCU level PRCMM, etc.) to enhance your skills over the traditional off-site training and woreda level review meetings?

Respondents :-yes I have got supports like mentorshps on how to I utilizes phone tablets compering with monthly reports for ICCM services and they came here more than three time to conduct supportive supervision and they thanks me on my jobs, this is good appreciation and motivate me on my jobs, they supports improve my skill and my competencies.

We obtain on-site coaching,technical supports for many times from JSI/L10k ,during supporting us they observes best activities performances but from woreda health office told to them is no iCCM activity performances there.

We breaks negative perception of the community based on supports we got from JSI/L10k and we provide better quality services for the community, we provide services for the societies by using phone tablets this make us good quality services, active monitoring of their child by phone call or visit their home ,continuous supportive for them and communities awareness and mobilization were increased.

The thing that need improvement is continuous drug supply for iCCM services, time to time inform and supportive supervision from HCs and Woreda Health ofice,

Interviewer :- How eCHIS implementation helps you with iCCM service delivery?

Respondents :- eCHIS implementation helps a great on iCCM services ,it is like dictionary or guideline for example if I want to treat diarrhea and enters child assessment to the phone tablet it order me that provide this amount of drugs,provide this type of drugs and if I made a error eCHIS remind me and correct on it, by using eCHIS phone tablets to treat sick child is very good.

By using eCHIS phone tablet we simply identify cases, from me what expected is only fill the tablets after that phone tablets identify the case and guide me treat this disease by this drugs and ask me is you have such drugs? If I haven't the drugs eCHIS guide me where I have to refer the cases.thus if phone tablet fully implemented on works all activities were simply apply.

by using eCHIS phone tablets is not need such knowledge, we only enters child problems after that phone tablet identify the disease for example if I treat diarrhea it ask me is this diarrhea has blood or not placed on tablets after that give ORS or provide ORS with zinc or treat by A, level treatments or B ,level treatment with a correct dose and for a amount days and also eCHIS helps for appointment schedules, it guide us by asking do you want to monitor

this patient? if we reply yes it set the date we want to monitors and if the appointment date is passed it show red color, depending on this we trace the lost to follow up childs.

Interviewer:- How was the introduction of the iCCM/CBNC module into eCHIS affected the iCCM/CBNC service delivery?

Respondents :- introduction of the iCCM/CBNC module into eCHIS has big benefits, it prevent us to carry hard copy paper and refer simply from phone tablets, at rest time in our homes we could refer what we didn't understand ,we can teach the people from phone tablets referring this module,to do every activities by phone tablet it make us to do simply in short period of times.

iCCM/CBNC module has immense explanation for every disease, for example for sever case it explain with reason, provide treatment with reason, if not refer it asks for what reason.

introduction of the iCCM/CBNC module into eCHIS helps for child appointment dates ,it also asks the reason if the child is not came in exact date is that , is the child discontinuous the services? or address changes? or move other health facility? or need explain other reason.

This module has explanation with reason for referral to HCs ,for example;if the case is sever and couldn't treat at health posts, like bloody diarrhea it must refer to HCs .

iCCM/CBNC module has criteria to treat at health posts ,for example;if diarrhea or pneumonia and could treat by ORS with zinc or amoxicillin 250g per a day respectively and if beyond this drugs refer to HCs or by color coding if yellow it could treat at health post ,if red it need referral for childs.

Benefits of this module is it improve our knowledge's and confidence on treatments, prevent us from missed treatments ,make us to provide standardized treatment with correct dose,

Interviewer:- Describe the main issues faced by the health system to identify and treat neonatal infections in the community. What are the critical factors affecting the delivery of iCCM to clients?

Respondents :- Since Health Center and private clinic is near to us the community prefer health center and private clinic ,the community perceived that there is treatment difference between health post and HCs.

The people were not focused on preventive method like safe drinking water ,Personal hygiene and sanitation especially for children.Many person says OK for every counselling but practically no changes.

If we order on a drugs how to given for children some people are not follow our order, for example if we order syrups mix withe clean water, they didn't follow our orders and the children mightn't recover from disease.

We have full material to treat the children but some time we faced Zinc shortages and weak supports from top level.

Interviewer:- How do you think the COVID-19 pandemic and/or COVID-19 response measures affect the uptake and/or delivery of iCCM services?

Respondents :- at first time communities are very frighten COVID-19 infection and we also fear to visit home to home activities. many people were not came to health post because of COVID- 19 infection. at COVID-19 periods for three or four months we didn't do our jobs at that time we only refer to HCs for any cases and affects on iCCM

services deliveries. While COVID-19 started in our country we also absent from our jobs because stay at home were declared and community were also treat their sick infant by traditional medicine because of COVID-19 infection fears.

Generally COVID-19 infection affects the whole our daily activities ,the community were not brought their child to health posts and livelihood of the peoples in our catchment areas were extremely affected.

Step by step the fear of COVID-19 infection is reduced because the community were perceived that this is common cold no COVID-19 infection , we provide vaccination for community and use COVID-19 prevention methods.

Interviewers :- Explain to us any changes you observed as the result of the implementation of the COVID-19 adaptive iCCM implementation strategies? – What impact has the strategies had? What are the particular features of the strategies that made a difference?

Respondents :- at present time we have seen changes on iCCM services delivery.the community were actively bring their sick children to health post and show their babies without any fears. At present time system supports from HC and Woreda health offices were good ,we also highly motivated to provide iCCM Services after we adapt COVID-19 infection .

After COVID-19 adaptation We works our jobs as usual on community mobilization ,demand generation provide sick child treatments and community participation were improved at idir "afosha" places, and community meeting

Interviewer :- What are the high-level benefits that are attributable to this support/IR?

Respondents :- by JSI/ L10k supports we got large benefits first it make us to treat children under five years ,

Child death were decrease because we early got sick infants and provide treatments for them and if we couldn't treat active refer to HCs .since our service is free cost the mother were not exposed for unnecessary expenses

our community awareness were highly improved , continuous supportive supervision provided for us, since we used eCHIS we simple identify case,treat with standardize drugs ,appoint the date and trace lost to follow ups ,improve our activity quality,improve our knowledge and skills.

Interviewer :- Please explain to us the feasibility/practicably of this support/ IR for national scale-up?

Respondents :- yes it can possible scale up for other regions if they used eCHIS it can simple expand throughout HEWs ,because HEWs lives in the communities, works in the communities and know the communities problems.

It can feasibility if all necessary supply and essential commodity is fulfill for HEWs and sufficient training were given for HEWs and provide phone tablets ,communities were actively participate and aware on iCCM Service deliveries.

Interviewer :- How are the activities/efforts embedded in the PHC and woreda routines/work streams? What implementation strategies are incorporated with the PHC and woreda annual work plan?.

Respondents :- we incorporate iCCM/CBNC services to our annual work plans,all delivered children were followed under post natal cares and the iCCM/CBNC services also planned with PNC services.

Interviewer :- Do you have anything think is important to tell us that we have not asked you?

Respondents :- nothing I have added.

Interviewer: thank you for your time and valuable ideas

Respondent: thank you too.

I	Identification	
1	Questionnaire ID	T02
2	Area Identification	
3	Name of Woreda/Zone/Region	Lume wored
4	Name of facility	HCs
5	Name of moderator	
6	Name of a note taker	
7	Date of discussion	16/06/2022
8	Participant #	one
9	Audio File #	019
10	Start time:	3:23
11	End time:	4:59
12	Transcriber	

Interviewer: Can you please describe the demand generation/SBCC activities you are doing on iCCM/newborn health? What are the strategies used to raise awareness?

Respondent :- first of all our catchment area has seven rural kebele and two town kebeles. mostly ICCM activates were done with attention at Health center and at kebele level health posts by Health extension workers. simply at present time ICCM activates were on good position under our Health centers this is not mean that at previous time there were not work done on Child health's , after JSI organization has been provide training for Health Extension workers as much as possible better attention were given ,to do this activities the awareness should be given for the communities level as much as possible the social mobilization were given on sick children can be treated at health post by Health extension workers and Health center also provide medical commodity and drugs supply for ICCM purpose .

according that character we governing each others to improve ICCM ,Monthly we received activity reports,fortunately our site were governed by eCHIS and supported by electronic Tablets this is based on booklet chart in the tablets to treat sick children and we also provide big attention for this issues up to Heath post supports.

Previous time the socialites were directly came to health center for child treatments but now a time after the socialites were got good awareness the ICCM activities is in good progression at Health posts.

Interviewer:- How is the engagement of communities/WDA networks,Pregnant women conference,Iddir and Kebele structure in the implementation of integrated community-based case management (ICCM) of common childhood illnesses/possible serious bacterial infection (PSBI)?

Respondent :- to say rightfully at previous time no as such focused child health Education were given for Communities but after specific training and refreshment training were given for health workers all Health workers directly assigned to every kebeles. after this they coordinated with Women developmental Army, Kebele structure

gives awareness creation for the community on sick children can be treat at health posts by Health Extension workers , based on delegation strict follow up were conducted and the evidences were also support this activities.

After big attention were given for child health's, , Health education is given for the communities every person have to be treat their children at Health Post if their child were sick.

pregnant women conference were conducted at kebele levels two times in one months, on this circumstance all pregnant women should be attend their Anti Natal care(ANC) follow up at Health posts if any cases need to referral HEWs refer to Health Centers depending on the problems.

we also provide awareness and gives solution for any problems with Health Extension workers especially top level bodies gives attention for this activities and conduct mentorships.

All Health extension works provides service house to house visits and provide Health Education for the communities and conduct every follow ups, mostly Post Natal Care which is provided at Health Posts and at House hold level on this occasion health education and awareness creation activities were provided for a women s because all Health extension workers have had update data on her catchment area they know who deliver,when delivered ,what mothers and baby status and also they lives in that kebeles.after delivery post natal care were given at home to home based on this if there is the problems immediately provide counselling and order to take her children to health posts for farther treatments and for the rests communities health promotion were givens.

Interviewer:- What are some of the biggest challenges about SBCC activities for newborn care?

Respondent :- At previous time the communities were no have any awareness on sick Children were treat at Health posts, they perceive that Health Extension workers has been only provide family planning and house to house education , the other challenges is most communities were not believe child treatment drugs were available at Health Posts and the people complain to us is Health post is closed during they need services.

Interviewer:- How do you describe the strength of the support you got from the PHC and the project as well as your efforts to address barriers to deliver iCCM/PSBI during COVID-19?

Respondent :- for iCCM/CBNC activities JSI/L10k provide for us many supports ,the biggest supports is provide adequate and essential training for Health workers on how to work iCCM/CBNC activities and what required things for this activities.

relating to supports on iCCM/CBNC activities to make modernization they provides for us for all HEWs Tablet phone, ***strength of the support*** is, at previous time we used paper based recording and registration for any activities but at now time this activities is directly changed to paperless based on tablets phone, Tablet phone is provided for all Health Extension workers ,for two Health Extension workers two tablet phone and for One Health Extension workers One tablets phone is provided and activities were also mentor , JSI /L10k organization coordinating with Health Centers makes ICCM activities to got attention and for many times JSI/L10k organization

provide on jobs refreshment training and Mentorships for Health workers working on infant and children health it self is also bigger things not only our catchment area but also for the entire countries problem can solves.

Interviewer:- Was the support you got from the project is helpful?

Respondent :- this is no question, this is big support for us , even-though at previous time child health activities were involved in Health Extension Packages no attention is given for this activities but at present time especial attention were given for ICCM.

on job Mentor ,on job training this indicate that JSI/L10k organization gives attention for this activities .JSI/L10k organization also supports us how to standardize ICCM Service deliveries and provide correction on comments services at Health post levels.

Interviewer:- What was changed after you got supports?

Respondent :- after iCCM/CBNC activities were got attention we have to measure our job progression compare with before two and three years. based on this we see a big changes before two and three year not more than two or three children were treated at health posts ,but at present times after iCCM/CBNC activities were got attention.

many children under their catchment area were addressed and we also seen HEWs activities relating to their monthly reports, from this we observes there is immense changes, for example if we see pneumonia before three year and measured in numbers compare with this years there is big difference in number ,and also if we see relating to sepsis this also make us to compare and contrast previous year with this year activities , so we observe from this the achievements Number is increase by two times .

Interviewer:- Did do you get the support system helpful (mentorship and on-site coaching methods, supervision, technical support, PHCU level PRCMM, etc.) to enhance HEWs' skills over the traditional off-site training and woreda level review meetings?

Respondent :- yes ,JSI/L10K organization genuinely give attention for this activities ,provide mentor , awareness creation, provide training and prepare independent meeting to see improvement on ICCM Usefulness.; hence not only provide training but also to realize what is progration, what is changes for each health post level and to see what is difference compared with previous one.

After Health Extension worker took adequate training they have been working their jobs in full confidence because they supported by tablets phone. this prevent treatment error and make them to have enough confidence.

all this changes is the results of mentorship ,at now time every Health extension worker has improved their skills and Compitants knowledge.

Simply if sick children come to health post and HEWs treat without any fear if above their capacities they simply refer to Health center for further treatments .therefore this shows that one Health workers to have full confidence to treat infant and children

Interviewer:-Did do you tell me that the difference between on sit coaching ,mentoring with that of off site training which is relocation from work place and took training s?

Respondent :- off site training have its own benefits ; because all Health Extension workers came from different place this make them to share their best experience from each others not only took a training but also they learned from each other , what the challenges or the problems can face during their works and also they observe what solution planned for the challenges at their work places.

Interviewer:- How eCHIS implementation helps you with iCCM service delivery?

Respondent :- related to eCHIS the foremost is it should be best-known an electronic system and fundamental for our works, based on eCHIS we simply know what we have on our hands , at previous time the annual plan given from top down based on conversion factors and the number we have also given from top Level offices. based on this when we see our achievements from plans opposite to each others but after we start to use eCHIS we observe that at one Health post the actual house hold numbers, total population number, under five years,under two years have specific numbers according to their catchment area this is the actual data what we have at hands and used for decision making for what ever .

based on eCHIS activity means relating to ICCM services for example eCHIS Tablet phone used as treatment guide for sick infant.

directing by using eCHIS Tablet phone is the main services which is displayed on its platforms firstly who can I treat ,what can I treat then it also gives appointments date for the mother when she come back if she is not came on the appointment date it shows that a reminder signs.so eCHIS is simply to guiding us what we forgotten in modern methods .eCHIS prevents us from services error means if one infant sick by pneumonia it guides us what is services provided, what drugs given for,for how many days given,in a days for how many times given , how to follow up treatment progression is clearly placed in phone tablets. additionally eCHIS doesn't need special sciences for this reason it simply ask on phone tablets us that if one infants is sick it asks by what disease and then we answers for disease then simply go to up to treatment. if treatment is correct it lead us up to the end on correct ways .

if one HEWs engaged by others idea and order wrong drugs the eCHIS prevent from under dose or over doses drug provision, it also guide for how many times stayed with HEWs, after that what can proceed if cured from the disease he/she can discharged or if not how can referred to Health Centers in eCHIS simply located.

Interviewer:- How was the introduction of the iCCM/CBNC module into eCHIS affected the iCCM/CBNC service delivery?

Respondent :- to work as Health care workers you should be lead by guideline because every body what every he/she has skills and knowledge should be guided by a guideline because service delivery will be delivered according to sated standards. All ICCM Services is delivered by prepared module or chart booklets we understand

that what the module is depends on to know bacterial infection , for example what the sign and symptoms of sever local bacteria and sever pneumonia is look likes , when clients come with her baby ,we ask her when infection starts,for how long time sick,what the cause of the disease ,is he/she sick before, what is complain and take sick baby history and execute physical examination of the babies then refer to ***iCCM/CBNC*** module to treat based on we took examination history of the babies or to give the decision for referral to health centers.

The other benefits of the eCHIS is if one sick infants sent from one kebele by what modality sent, what baby is sick ,who sent and every thing of history of infants were sent to Health Center and after infant got treatments at Health Centers eCHIS Focal person is sent the infants results back to Health Posts on simple ways.

to provide iCCM/CBNC service based on iCCM/CBNC module is a basics for every activities and make our child health performances to standard and improve iCCM/CBNC services qualities and we also seen that all HEWs are used iCCM/CBNC module.

all Health Extension workers should be used iCCM/CBNC module because it helps from starting case detection up to discharges, how appointment date is recorded on phone tablets,why appointment card is given for Mothers and indicate the date she came back with her appointment card. iCCM services deliveries used by iCCM/CBNC module has had high qualities ,simply how to record ,treat ,discharge, refer to Health centers and save the time, avoid to carrying hard copy different recording documents. our communities were also very happy because we have been using eCHIS phone tablet and electronic methods.

Interviewer:- Describe the main issues faced by the health system to identify and treat neonatal infections in the community. What are the critical factors affecting the delivery of iCCM to clients?

Respondent :-To do iCCM service deliveries Human resource is very important. in many kebele we have only one health extension workers if she close health post and goes for house to house visit services the societies might not gate the services at health post and some people does not accept that Health extension workers could treat infants at health posts, so they directly go to Health centers or private clinic without health post referral systems.

The other challenges is through the year we are busy on different campaign this also interrupt iCCM service deliveries. to rich our goal drug supplies and commodities for iCCM services is big challenges for our catchment areas ,because we bought drugs by health center budget and distributed freely for iCCM treatment to do this we have budget shortage this also a big factors iCCM services delivery.

Interviewer:- How do you think the COVID-19 pandemic and/or COVID-19 response measures affect the uptake and/or delivery of iCCM services?

Respondent :- at starting time COVID-19 pandemic is not only problems on iCCM services but also big challenges for the entire activities because every people has awareness and fearing on the transmission of COVID-19 infection at that time communities were prefer to stay at their home than to be infected by COVID-19 infection from Health facilities. people were not came market places, Health facilities, even from rural to town because the rural people

perceived that this Disease is came from town and could affects town people and Health care provider was the most distributor to rural people

at health facilities the services were highly interrupted specially at EPI Department and daily activities and livelihood of the people were affected but gradually this fearing and disruption is changed because many effort have been done from top to down on COVID-19 prevention and controls methods including COVID-19 Vaccination.

Interviewer:- Explain to us any changes you observed as the result of the implementation of the COVID-19 adaptive iCCM implementation strategies? – What impact has the strategies had? What are the particular features of the strategies that made a difference?

Respondent :- to say truly the communities and all Health care workers have been taking COVID-19 Vaccination this make them to have full of confidences on COVID-19 infection transmission. in addition to this all of them has been applied COVID-19 Prevention Methods on their jobs. so at now time COVID-19 is not as major challenges on HEWs activities in the communities, to support Health post and to give iCCM Service.

We also measure iCCM activities performance at kebele level and we observed that the performance is twofold from previous time .a present time we proud of health care workers because they give them selves for the communities of they served specially on iCCM services Health workers work's highly motivate post COVID-19 pandemic.

Interviewer:- What are the high-level benefits that are attributable to this support/IR?

Respondent :-Big benefits we obtain from this support is working with in the communities to solve their problems itself is benefits and our community were highly aware on iCCM Services and Our Health extension worker also got sufficient training .

eCHIS services improve HEWs confidence and competencies on sick infant treatments and communities also increases the acceptances of Health extension workers especially on iCCM serveries deliveries which is decrease a child death .HEWs treat the infants in short period of time in modern system to provide standardized services .

Interviewer:- Please explain to us the feasibility/practicably of this support/ IR for national scale-up?

Respondent :- ideally I supports if this project/implementation research is expanded to other region because we see from this support how to one Health Extension Workers simply treat sick infants by using eCHIS at health post or communities level with full evidences, in short period of times and provide standardized services on iCCM service delivers .since our community in different region has the same structures like Health system delivery and Rural Health extension workers the best experiences we gate simply can expanded for other region by incorporating with existing Health extension packages.

Interviewer:- How are the activities/efforts embedded in the PHC and woreda routines/work streams?What implementation strategies are incorporated with the PHC and woreda annual work plan?

Respondent :- ICCM Activity is the main and fundamental activities at previous year we also incorporate ICCM activities in our annual plans but no attention given for it . by this year all ICCM activity is incorporated to annual plan with special attention on under two months children s

Interviewer:- Do you have anything think is important to tell us that we have not asked you?

Respondent :- to perform ICCM/CBNC activities commodity supply is very critical and the service follow up should be continuously supports from JSI/L10k organization and from other organizations up to this problem is solved at grass root levels .

Interviewer :- thank you very much for your time and response to my question

Respondents :- thank you too.

I	Identification	
1	Questionnaire ID	T01
2	Area Identification	
3	Name of Woreda/Zone/Region	Lume wored
4	Name of facility	HCs
5	Name of moderator	
6	Name of a note taker	
7	Date of discussion	14/06/2022
8	Participant #	one
9	Audio File #	015
10	Start time:	3:00_________
11	End time:	_4:_33______
12	Transcriber	

Interviewer :- Can you please describe the demand generation/SBCC activities you are doing on iCCM/ newborn health? What are the strategies used to raise awareness?

How is the engagement of communities/WDA networks in the implementation of integrated community-based case management (iCCM) of common childhood illnesses/possible serious bacterial infection (PSBI)?

Respondents :- after I and Health Extension worker took training on iCCM/CBNC , attention were given for this activities and we acquire a big changes. before JSI/L10k supports Communities awareness is low on sick infant treatment at health post and those peoples were gone private clinic and different hospital to treat their infants and they expenses to bought expensive drugs from at that place but the same drugs like ORS ,Gentamyccine is given at health post freely for sick infant and through the time the community itself recognize extra cost for the same drugs to treat their infants and after people know such things they start to treat sick infants at health post by HEWs.

at kebele level we have Women development army who are divided in to groups and one to five, pregnant women conference on this events we aware Pregnant Women s on ANC ,Deliveries ,PNC and EPI services provision in health posts in-addition to this the sign and symptoms of bacterial infection on infants and children's .

we also used community stakeholder like Aba Geda,Religion leaders specially on different health campaign .

Interviewer :- What are some of the biggest challenges about SBCC activities for newborn care?

Respondents :- at previous time the attitudes of people were very low on sick infant treatments at Health posts ,because they thought that HEWs couldn’t treat sick infants and not aware on service deliveries at health posts.

the others challenges were some people didn’t disclose their infant before two months ”hamachisa ”cultural practices which means one infant should named before two months from at births by ” wara kalu” before

nominate a names they didn’t show their infants to other person because they perceived that if an infants took any Vaccine and treatments before two months or before nomination by ” wara kalu” the infants will be infected by disease or can be deceased.

Interviewer :- How do you describe the strength of the support you got from the PHC and the project as well as your efforts to address barriers to deliver iCCM/PSBI during COVID-19?

Respondents :- on relate to support provided for us, firstly on capacity building for Health Extension workers and focal person to improve their skill and competencies on iCCM/PSBI is the strength of this projects. At present time JSI/L10k mobilize all health extension workers to provides treatment for sick infants at health posts by using updated ICCM /CBNC Module, which is prepared by afan oromo . if JSI/L10k were not provide supports, ICCM service deliver were already stopped the services ,thanks for JSI/L10k we have seen big changes on sick infant treatment and improved service qualities.

Before Project supports the communities go far away to treat their infants, this expose the societies for unnecessary expense but after obtained supports we also give attention for child health and the community also gate adequate services in rural kebele with free costs. we also suggest JSI/L10k organization on necessary support materials like drug supply to improve infant treatment more than at present performances and on jobs refreshment training were also very necessary because HEWs alert for their jobs .

Interviewer :- Did do you get the support system helpful (mentorship and on-site coaching methods, supervision, technical support, PHCU level PRCMM, etc.) to enhance HEWs’ skills over the traditional off-site training and woreda level review meetings?

Respondents :- yes we got supports ,like; mentorship and on-site coaching to improve the HEWs skills. for example HEWs might be made error while case identification , depending on steps of case assessments and treatments for identified cases ,so technical support were enhance HEWs skills and competencies. off-site training and Review meeting is presented by power points of work performances, from this many HEWs were not got the chance to ask a question and need extra elaboration but on mentorship and on-site coaching face to face discussion on registration and recording, without fear communicating to each others on different cases and obtain extra elaboration and demonstration on child treatment to improve the activities.

after we got supports like mentorship,coaching and supportive supervision we have seen big changes on sick child treatments ; for example before support ***systems,*** child Pneumonia treatment were very low. but at present after we got supports the treatment number is highly increase.

If we see before JSI/L10k supports at sites of Derar dembel Health post, which is far distance from HCs the people resident that kebele were treated at koka Health center, but after we got technical support child treatments like

pneumonia ,diarrhea and local bacterial infection treated by Health Extension Workers at Health post level, this is big changes on life saving .

Interviewer :- How eCHIS implementation helps you with iCCM service delivery?

Respondents :- eCHIS assist iCCM service delivery, during child treatment were performed HEWs treat what she could do, if child treatment need referral to health center she simply refer to HC by eCHIS Tablets, at HC focal person took training on iCCM received referral from Health post ,provide treatment for childs and sent actively feed backs to health posts , eCHIS supports on case identification because it guides steps to identifies the cases after that provide treatment drugs with correct doses give and interval time taken, even sever cases were also colored by red color indicates need emergency referral and treated at Health Centers.

eCHIS avoid false data and improves data qualities and prevents treatment error .eCHIS tablets helps for appointment schedule and monitoring of children on follow ups by color coding which are, gray color indicate on appointments ,green color indicates the appointment date is near future and red color is indicates appointment date is passed.

Interviewer :- How was the introduction of the iCCM/CBNC module into eCHIS affected the iCCM/CBNC service delivery?

Respondents :- iCCM/CBNC module has been brings for us big benefits ,every HEWs cites for infant treatments simply from iCCM/CBNC module tablets,this module helps us how to identify the cases,What types of counseling is given for the mothers ,How treat to clinically identified cases ,the types of drugs given for identified cases with exact doses .

the module helps to make a decision for referral based on color indication in the module to Health centers, the module also helps for appointment dates to come back for treatments ,for example; if the disease is pneumonia the children have to came back in three day ,if has sever diarrhoeal disease the children have to came in one days and iCCM/CBNC module states to write Families phone number to know the status of the child's and lost to follow up tracing .

Interviewer :- Describe the main issues faced by the health system to identify and treat neonatal infections in the community. What are the critical factors affecting the delivery of iCCM to clients?

Respondents :- we have seen several knowledge and skill problems between HEWs , as by nature human being has different characters HEWs ability on treatments were also different. Some HEWs are active and has ability to understand what they trained on iCCM/CBNC and the others are inactive.

Some kebeles adjacent to modjo towns and they got iCCM/CBNC service from a Modjo town ,they didn’t served at Health post of their Catchment areas .inadequacy of treatment drug supplies also as factors for iCCM/CBNC

service,at previous year we have been received daily reports of under five pneumonia treated, diarrheal disease treated,Local Bacterial infection treated based on HEWs reports we supervises them ,but by this year we are busy on different campaigns and didn't supervise them .

I*nterviewer :- How do you think the COVID-19 pandemic and/or COVID-19 response measures affect the uptake and/or delivery of iCCM services?*

Respondents :- During COVID-19 Pandemic , I am not here ,I am working in some Hospital but I know How the iCCM Services affected by COVID-19 Pandemic .from those ,one mother didn't took her sick infants because at that time she fear COVID-19 infection caught her and her babies at Health center. even the infant were under sever cases they took private clinic because they perceived that Hospital and Health center has many patients who might has COVID-19 Infection this expose them for unwanted expense.

Health care workers also fright to treat sick babies because the infants might contact with many person and the mother also reversely suspect health workers for COVID-19 Infection this scenario affects our daily activities. the Community living style highly affected because at that time no transportation ,high Fear of COVID1-19, movement restriction specifically at over crowded area but after period of time there has been a changes; because COVID-19 Vaccination were given for all of people, COVID-19 related death were decreased, Awareness on COVID-19 infection is decreases and the community perceived that there is no COVID-19 infection for at all.

Interviewer :- Explain to us any changes you observed as the result of the implementation of the COVID-19 adaptive iCCM implementation strategies? – What impact has the strategies had? What are the particular features of the strategies that made a difference?

Respondents :- already every body adapts about COVID-19 infection. Thus ;, routine iCCM service deliveries also provided for sick infants, all people brought their sick babies to Health post and health centers without fear of COVID-19 infection. Communities participation were also improved on iCCM Services,during COVID-19 infection started in our country all Health workers attention is on COVID-19 infection prevention and control but at present time our attention were on iCCM service deliveries like case identification and treatment on Pneumonia ,diarrhea for infant. System supports like supportive supervision ,Mentorship ,from Health centers were also improved on iCCM Services, at present time all HEWs Motivation for iCCM services were conditional ,because some time for several months Woreda Health office and Health centers team were give attention to bring up good performance we also recorded expected performances.

since all people perceived COVID-19 infection no harm and no agenda for all activities like Community participation ,Different community gathering and social mobilization on iCCM Service delivery regular and improved.

Interviewer :- What are the high-level benefits that are attributable to this support/IR?

Respondents :- iCCM Service delivery bring for us big benefits ,; like communities were not exposed for unwanted expenses. for example pneumonia, diarrhea is treated at health posts by HEWs ,child and mother death were decrease for example previous time to treat one infants communities might be need money if not gate the money the infants might be dead or to move long distance if no transportation and no money the infants might not gate treatment and this may lead to deaths but at present time all health post is provide iCCM service for free cost and community has been gate improved services.

utilization of technology depend eCHIS services also the best and standardize infant treatments.

***Interviewer :-* Please explain to us the feasibility/practicably of this support/ IR for national scale-up?**

Respondents :- If implementation research will expanded for other region it is useful because the communities residences may far distance from hospital and health centers so they want services near to their residences area, like our Woredas people got treatments at health posts the other area will need treatment and supports near to their living areas without moving far distances.

this support can be easily scale-up for other region because HEWs are working in the whole countries and The iCCM/CBNC service delivery already parts of their Health extension packages and integrated to it.

all responsible body and Health worker should be trained on iCCM/CBNC, particular attention should be given and technology supported eCHIS services should be very necessary because it guides as a guidelines and provide standardized treatments for sick infants .

***Interviewer :-* How are the activities/efforts embedded in the PHC and woreda routines/work streams?What implementation strategies are incorporated with the PHC and woreda annual work plan?**

Respondents :- iCCM service mean under five children services ,this has its own indicators like diarrhea, pneumonia,sepsis and has their conversion factors .

we break down Health centers annual work plan to bi annual,quarter,monthly and bi weekly then sent to kebeles and health posts.

iCCM service already in the annual work plans ,at previous year annual work plan is proposed based on annual woreda based plans conversion factors for example we plan under one year services from total annual Deliveries this is not consider actual data they have on at hands , but this year our plan is consider all house hold and total population who were registered by using eCHIS tablets classification,under five years children is registered, under three years children is registered,under one year children is registered by their kebele and zone catchment areas with their actual data what they have on their hands .

Interviewer :- Do you have anything think is important to tell us that we have not asked you?

Respondents :- the things I want to add is iCCM/CBNC services were good activities because it focus on child death minimization and we got many benefits from this projects ,every health care worker and community were highly mobilized ,iCCM services need continuous supports, refreshment training for Health workers and experience sharing between each Health extension workers if such things fulfill this projects will be sustained .

Interviewer :- thank you very much for your time and response to my question

Respondents :- thank you too.
